# Mitogenomes do not substantially improve phylogenetic resolution in a young non-model adaptive radiation of freshwater gastropods

**DOI:** 10.1186/s12862-024-02235-0

**Published:** 2024-04-08

**Authors:** Björn Stelbrink, Thomas von Rintelen, Ristiyanti M. Marwoto, Walter Salzburger

**Affiliations:** 1 Justus Liebig University Giessen, Giessen, Germany; 2 University of Basel, Basel, Switzerland; 3 Museum für Naturkunde – Leibniz Institute for Evolution and Biodiversity Science, Berlin, Germany; 4 Museum Zoologicum Bogoriense, Research Center for Biosystematics and Evolution, BRIN Gedung Widyasatwaloka, Cibinong, Indonesia

**Keywords:** Mitochondrial genomes, Species flock, Caenogastropoda, Ancient lakes, Indonesia, Sulawesi

## Abstract

**Background:**

Species flocks in ancient lakes, and particularly those arising from adaptive radiation, make up the bulk of overall taxonomic and morphological diversity in these insular ecosystems. For these mostly young species assemblages, classical mitochondrial barcoding markers have so far been key to disentangle interspecific relationships. However, with the rise and further development of next-generation sequencing (NGS) methods and mapping tools, genome-wide data have become an increasingly important source of information even for non-model groups.

**Results:**

Here, we provide, for the first time, a comprehensive mitogenome dataset of freshwater gastropods endemic to Sulawesi and thus of an ancient lake invertebrate species flock in general. We applied low-coverage whole-genome sequencing for a total of 78 individuals including 27 out of the 28 *Tylomelania* morphospecies from the Malili lake system as well as selected representatives from Lake Poso and adjacent catchments. Our aim was to assess whether mitogenomes considerably contribute to the phylogenetic resolution within this young species flock. Interestingly, we identified a high number of variable and parsimony-informative sites across the other ‘non-traditional’ mitochondrial loci. However, although the overall support was very high, the topology obtained was largely congruent with previously published single-locus phylogenies. Several clades remained unresolved and a large number of species was recovered polyphyletic, indicative of both rapid diversification and mitochondrial introgression.

**Conclusions:**

This once again illustrates that, despite the higher number of characters available, mitogenomes behave like a single locus and thus can only make a limited contribution to resolving species boundaries, particularly when introgression events are involved.

**Supplementary Information:**

The online version contains supplementary material available at 10.1186/s12862-024-02235-0.

## Background

Ever easier to obtain whole-genome sequence data have become a powerful source of information to address a diverse array of questions in ecology and evolutionary biology. Despite the increasing use of genome-wide data as a result of the ongoing advances in NGS technologies, traditional organellar DNA markers remain a key component in phylogeography, biodiversity and evolutionary studies, which is largely due to the moderate time and costs needed to generate such data and its straightforward compatibility and comparability to existing works, but also related to features such as gene orthology and a low recombination rate (e.g. [1, 2]). These arguments are of particular importance in the scientific exploration of species-rich (and non-model) groups such as gastropods, which comprise about 63,000 extant species [3]. So far, only a handful of reference genomes and comparatively few phylogenomic studies exist for this group (see e.g. [4–8] for an overview). However, the amount of genomic resources is also growing for gastropods [5]. In particular, there is a multitude of mitogenomes available, with many of them having been analysed to illuminate deeper evolutionary splits within particular gastropod lineages (e.g. [2, 9–12]). At the interspecific level, however, traditional mitochondrial barcoding markers (COX1 and 16S rRNA) still dominate the field of phylogenetics and phylogeography, typically in combination with a few fast-evolving nuclear markers.

The use of these ‘traditional’ markers is also a common strategy in the study of very young assemblages such as species flocks, that is, groups of closely-related species endemic to restricted geographic areas like islands or lakes (e.g. [13]). With respect to the latter, ancient lakes were long in the focus of evolutionary biologists owing to their extraordinary levels of freshwater biodiversity and endemism (e.g. [14–20]). Thereby, hypotheses arose whether ecological opportunity alone (e.g. [21–24]) or certain abiotic and/or biotic factors triggered the evolution of many of these freshwater species flocks. Among the main abiotic factors are basin changes and climate-driven lake-level fluctuations (e.g. [21, 25–27]), whereas key biotic factors include different reproduction modes (e.g. [28]), trophic specializations (e.g. [29–33]), and hybridization (e.g. [34–38]). Particularly the latter seems to drive biodiversity in several renowned adaptive radiations at various stages (e.g. [39–43]). However, virtually nothing is known about the extent of hybridization in non-model radiations, many of which account for much of the biodiversity in these isolated ecosystems that are currently under threat [19, 20, 44].

**Fig. 1:**
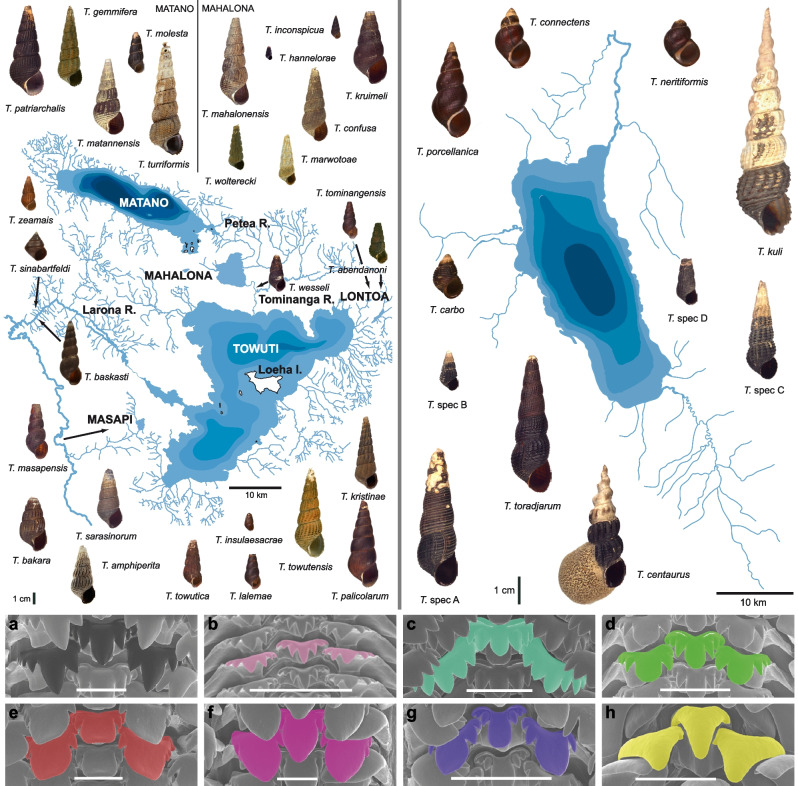
Shell diversity and radula morphs of *Tylomelania* morphospecies. Top-left: exemplary diversity of species endemic to the Malili lake system, top-right: exemplary diversity of species endemic to Lake Poso; bottom: exemplary radula diversity of **a** typical riverine form, *T. perfecta*; **b** *T. gemmifera* (Lake Matano), **c** *T. kruimeli* (Lake Mahalona), **d** *T. towutica* (Lake Towuti), **e** *T. sarasinorum* (Lake Towuti), **f** *T. matannensis* (Lake Matano), **g** *T. insulaesacrae* (Lake Mahalona), and **h** *T. carbo* (Lake Poso). Scale bar = 100 μm (modified from [45])

Understanding the relative roles of these factors and their impact on diversification not only requires a good biological understanding of the taxonomic target group but also precise knowledge of the environmental history of the study system. Both apply to the ancient Malili lake system in the central mountains and Lake Poso further north on Sulawesi (Indonesia), which harbour an extraordinary freshwater biodiversity (summarised in [19]). Remarkably, several of the well-studied species flocks in the ancient lakes of Sulawesi (gastropods, shrimps, crabs and telmatherinid fishes) show recurrent patterns, i.e. morphological distinctiveness, trophic/ecological specialization (substrate-specific feeding), two or more independent lake colonisations in several groups, high local degrees of endemism, and high rates of intralacustrine as well as lacustrine–riverine hybridization (see [19]). The most diverse and morphologically variable group in this system is the Sulawesi-endemic freshwater snail genus *Tylomelania*. It comprises 53 described morphologically and ecologically distinct species across the island, several of which show intraspecific variation in substrate-specific radula morphs [30, 45]; Fig. 1). Thus, the *Tylomelania* species flocks show phenotype-environment correlations sensu [46] characteristic for adaptive radiations, similar to the pharyngeal jaws in cichlid fishes (e.g. [33]).

Previous data indicate that ancestral populations of *Tylomelania* probably originated on the Australian margin and colonised Sulawesi about 5 million years ago (Ma), followed by rapid intra-island diversification and independent lake colonisation events [47]. Although age estimates for any of the Malili lake (and Lake Poso) radiations remain uncertain, available mtDNA data suggest that the different Malili lake clades of *Tylomelania* originated between 0.5–1.4 Ma [48]. Importantly, this time range falls within the age estimate for Lake Towuti (c. 1 million years, Myr), as revealed by the recently conducted Towuti Drilling Project [49]. In the available mtDNA-based phylogenies, the currently recognized morphospecies are recovered polyphyletic, with different haplotypes often clustering within different mtDNA clades. Although convergent evolution has been observed in other adaptive radiations (e.g. [50]), the observed non-monophyly of lake radiations might either be due to incomplete lineage sorting and/or mitochondrial introgressive hybridization within the lake endemics and between lacustrine and riverine species, which is not uncommon in rapid radiations (see e.g. [51, 52]). Signatures of mitochondrial introgression have been identified in *Tylomelania* [45] and telmatherinid fishes [34, 53]. However, it remains unknown whether such hybridization events also triggered diversification, particularly in the early phase of the different lake radiations (see e.g. [39]).

The generally young age of the *Tylomelania* radiation on Sulawesi and particularly in its lakes, the mismatch between available AFLP data and morphospecies, and the comparatively high degree of hybridization highlight the need for genome-wide data derived from NGS methods. However, only few genomic data have been published on this genus so far. These include a complete mitochondrial genome (mitogenome) of *T. sarasinorum* [54] as well as tissue-specific transcriptomic data of different populations of this species [55, 56]. *Tylomelania sarasinorum*, endemic to Lake Towuti, is of particular interest because of the existence of two populations showing different substrate-specific radula morphs, potentially indicative of early stages of incipient speciation. However, none of these previous genomic data contributed to a better understanding of colonisation processes, intralacustrine diversification patterns, interspecific relationships and levels of introgression in this genus.

In this study, we aimed at gaining a deeper insight into the *Tylomelania* snail adaptive radiation on Sulawesi, with a particular focus on the Malili lake system, from a mitogenomic perspective. To this end, we assembled mitogenome information of 77 individuals from Illumina shotgun sequencing data and assessed how promising mitogenomes are for resolving phylogenetic relationships within a young species assemblage of freshwater gastropods. More specifically, we were interested in (*i*) how variable such mitogenomes are across this species flock; (*ii*) whether or not mitogenomes provide more phylogenetic information compared to single genetic markers; and (*iii*) if other mitochondrial markers not commonly used in molluscan phylogenetic studies are equally informative at the inter- and intraspecific level.

## Methods

### Sequencing and read processing

A total of 78 *Tylomelania* specimens covering 27 out of the total of 28 morphospecies (except for *T. molesta*) described from the Malili lake system (see [45]) was examined (Lake Mahalona = 9 species, Lake Matano = 6 species, Lake Towuti = 11 species, Lake Lontoa = 2 species, and Lake Masapi = 1 species; note that some species co-occur in different lakes). The dataset was complemented with three additional morphospecies from the Lake Poso system and four riverine taxa (Additional file 1: Figure S1, Additional file 5: Table S1, and Additional file 6: Table S2). The material was collected between 1999–2020, and DNA was isolated using a CTAB protocol developed for molluscs [57]. Quality of the DNA was originally evaluated using an agarose gel, based on which samples were selected for Illumina sequencing. However, quantity and quality of the DNA and the DNA libraries was again assessed prior to sequencing with a Fragment Analyzer (Agilent) and a HS Large Fragment Kit and a HS NGS Fragment Kit 1–6000 bp, respectively, at the Genomics Facility Basel at the ETH Zurich Department of Biosystems Science and Engineering (D-BSSE).

For low-coverage whole-genome sequencing (lcWGS), we aimed to generate c. 15 Gb per library to obtain a rough coverage of 5X given an estimated genome size of 2.4–2.7 Gb based on preliminary Illumina MiSeq data (pers. obs.). DNA libraries were sequenced on an Illumina NovaSeq 6000 platform with a S2 Reagent Kit (300 cycles = 2 × 151 bp) at the D-BSSE. Raw reads were trimmed using Trimmomatic 0.39 ([58]; settings: leading = 3, trailing = 3, sliding window = 4:15, minlen = 50), and their quality was assessed before and after the trimming step with FastQC 0.11.8 (http://www.bioinformatics.babraham.ac.uk/projects/fastqc).

### Mitogenome mapping and annotation

For the mitogenome mapping step, we made use of the available annotated mitogenome of *T. sarasinorum* (GenBank acc. no. NC_030263, mitogenome length = 16,632 bp; [54]). Trimmed reads were mapped against this mitogenome using BWA-MEM 0.7.17 [59, 60]; the resulting sequence alignment map (SAM) files were converted into binary alignment map (BAM) files with SAMtools 1.7 ([61]; settings: removal of unmapped reads, MAPQ ≥ 25). Duplicated reads were discarded a posteriori using MarkDuplicates (Picard; https://broadinstitute.github.io/picard) as implemented in GATK 4.2.2 [62, 63].

BAM files were further visualized and processed in Geneious Prime 2023.1.2 (https://www.geneious.com) to (*i*) create consensus sequences of the individual BAM files (settings: highest quality = 60%, threshold for sequences without quality = 65%, call Sanger heterozygotes = 50%); (*ii*) transfer annotations from the reference sequence (NC_030263); (*iii*) to extract the 13 coding DNA sequences (CDS) and 2 rRNA genes for each sample for downstream phylogenetic analyses; the 22 tRNAs were not considered here, because both the mapping and annotation from the reference sequences failed for several tRNAs; and (*iv*) to align the gene-specific sequences using the MAFFT [64] plugin with default settings.

### Assessment of DNA quality

We subjected the mapped reads (before and after duplicate removal) to DamageProfiler 1.1 [65] to create so-called damage plots. Such plots, among other criteria, are typically used in ancient DNA studies to prove the authenticity of the nucleic acids (see e.g. [66, 67]). Here, we applied this step to assess whether the DNA of the older samples isolated more than 20 years ago has suffered noticeable DNA damage as reflected by an increased C to T and G to A base misincorporations towards the ends of the reads (e.g. [67]), potentially leading to conflicting phylogenetic inferences.

### Phylogenetic analyses and cophyloplots

Statistics for the individual multi-sequence alignments were assessed with AMAS [68]. Single-locus phylogenetic analyses were conducted using IQ-TREE 2.2.0 ([69, 70]; settings: GTR + Г for all partitions including codon partitioning for the CDS, 1,000 ultrafast bootstrap, UFBoot, replicates). The same strategy was applied to the multi-locus dataset (13 CDS and 2 rRNAs, i.e. a total of 41 partitions), which was extended with COX1 and 16S rRNA sequences of two sister species of *Tylomelania*, i.e. *Pseudopotamis semonis* (GenBank acc. nos. AY312049–AY312050) and *P. supralirata* (GenBank acc. nos. AY311944–AY311945). Thereby, the 12S rRNA and 16S rRNA datasets were aligned using MAFFT resulting in a final concatenated alignment length of 13,630 bp. In addition to the previous setting, the analysis was re-run without defining a substitution model *a priori*.

In order to compare single-locus tree topologies between the 15 genes, 14 cophyloplots were generated with the R package phytools 1.0–3 [71] for the R statistical environment 4.1.1 [72] and using the COX1 phylogeny as the reference.

### Mitochondrial gene representation in GenBank

We searched for the number of nucleotides available in GenBank (20 July 2023) for Caenogastropoda and Mollusca using the following search terms: ‘12S’, ‘16S’, ‘ATP6’, ‘ATP8’, ‘COI’/ ‘COX1’, ‘COII’/ ‘COX2’, ‘COIII’/ ‘COX3’, ‘CYTB’, ‘ND1’, ‘ND2’, ‘ND3’, ‘ND4’, ‘ND4L’, ‘ND5’, and ‘ND6’. To only count target fragments and not complete mitogenomes or other genomic resources, we limited the results to a maximum length of 2,000 bp for all mitochondrial genes.

## Results

### Read and mapping statistics

Although representatives of the genus *Tylomelania* have not yet been sequenced on a NGS platform on such a large taxonomic scale and based on DNA isolated in part from comparatively old material and using a standard mollusc-specific CTAB protocol, a comparatively large number of raw reads was generated for most of the samples, ranging from 161,954–137,791,566 (mean = 58,032,742, median = 44,376,141) read pairs, of which the majority (62.6–95.6%, mean = 94.2%, median = 94.6%) passed the trimming step. Of those read pairs, 326–816,369 (mean = 122,138, median = 89,125) were mapped against the mitogenome reference sequence with a mean coverage of 2.6–7,085.6 (mean = 1,031.8, median = 741.4). After duplicate removal, 137–561,131 (mean = 96,674, median = 68,902) and thus 42.0–87.9% (mean = 79.1%, median = 81.3%) of the original mapped read pairs remained, retaining a mean coverage of 1.0–4,926.6 (mean = 812.9, median = 589.7) across the entire mitogenome (see Additional file 5: Table S1 and Additional file 2: Figure S2 for details).

### DNA quality assessment

Overall, no DNA damage was observed among the 78 samples. However, given the low amount of reads and the poor coverage after the mitogenome mapping (Additional file 3: Figure S3 and Additional file 5: Table S1), *T. mahalonensis* 2001 from Lake Mahalona was discarded from subsequent analyses.

### Phylogenetic analyses and cophyloplots

**Fig. 2:**
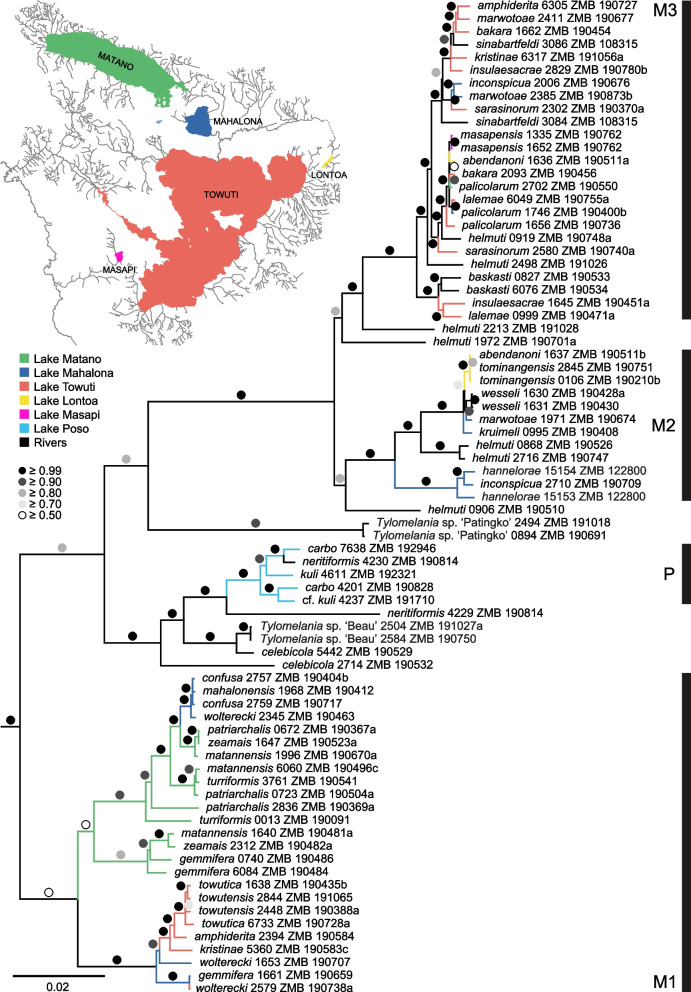
Multi-locus phylogeny based on 13 CDS and 2 rRNAs using IQ-TREE 2 and 1,000 UFBoot replicates. Branches of riverine species are coloured in black, branches of lacustrine species are colour-coded according to the Malili lakes shown in the inset map (Lake Poso not shown)

The two phylogenetic analyses with IQ-TREE2 – either based on the GTR + Г model or the ModelFinder option – resulted in very similar topologies; however, we hereafter only discuss the results obtained with the GTR + Г model. Accordingly, the multi-locus phylogeny revealed a highly supported ingroup (UFBoot = 100), including four independent lacustrine clades, one for Lake Poso (clade P, UFBoot = 100) as well as three for the Malili lake system (clades M1–M3; Fig. 2). Whereas clades M2 and M3 are well supported (UFBoot = 100), clade M1 only received moderate support (UFBoot = 56). Overall, however, the support is high to very high for the remaining nodes and in general considerably higher compared to a single-locus phylogeny based on COX1 (UFBoot > 90: multi-locus = 61 out of 76 nodes ≙ 80.3%, COX1 = 42 out of 76 nodes ≙ 55.3%).

Clade M1 consists only of lacustrine taxa occurring in the three main Malili lakes. The species belonging to Clade M2 either inhabit Lake Mahalona or Lake Lontoa, whereas clade M3 is the most diverse in terms of geographic distribution, including species from all five lakes of the Malili lake system. Within the latter two clades, also riverine samples are present. Taxonomic inconsistencies with respect to the position of particular morphospecies within and across the lakes exist in all three Malili clades. Moreover, some of the morphospecies occurring in the same lake are placed in different clades. This also includes *T. inconspicua* (placed in M2 and M3), which is, together with another species from Lake Mahalona – *T. hannelorae* (clade M2) – analysed here, for the first time, in a molecular phylogenetic context. According to a preliminary COX1 phylogeny, the specimen of *T. sarasinorum* used as the reference for the mitogenome mapping step (NC_030263; [54]) is closely related to *T. sinabartfeldi* 3084 (UFBoot = 88; data not shown).

**Fig. 3:**
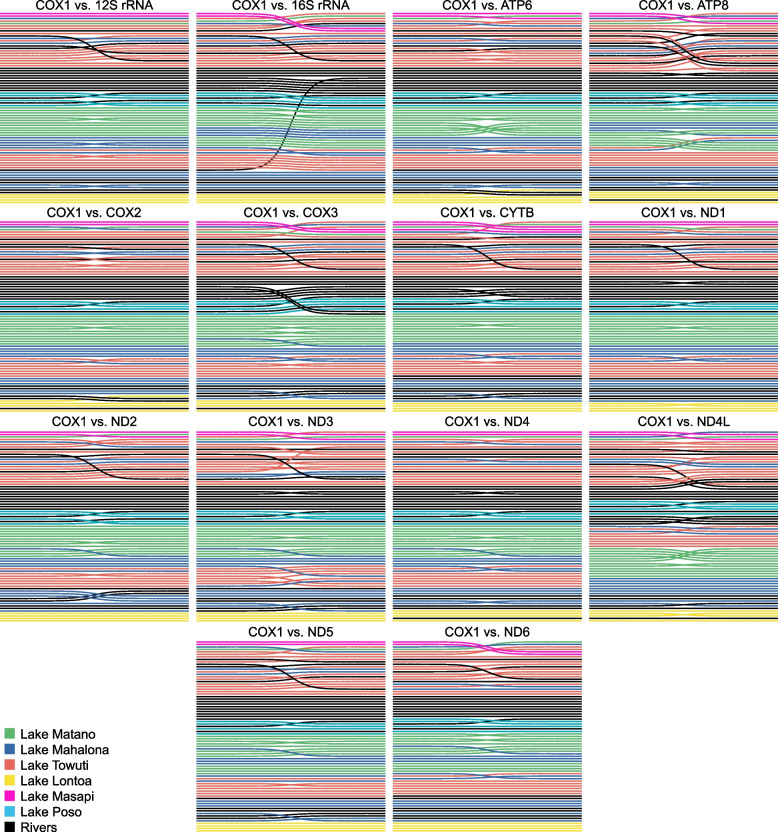
Simplified cophyloplots for the different mitochondrial loci analysed vs. the COX1 topology (left side of the plot). See Additional file 4: Figure S4 for the complete cophyloplots showing the individual topologies

The cophyloplots mainly revealed congruent topologies, however, with a larger amount of topological discrepancies between COX1 and, for example, 16S rRNA, ATP8, COX3, ND3, and ND4L (Fig. 3 and Additional file 4: Figure S4). The majority of these discrepancies are caused by the relation of the riverine taxa with respect to the lacustrine clades. However, those relationships are often only weekly supported in both of the two comparative single-locus phylogenies.

### Genetic variability

Overall, a high genetic variability among the 15 loci was observed. Accordingly, the relative frequency of variable and parsimony-informative sites ranged between 23.3–33.2% (mean = 28.4%, median = 27.6%, COX1 = 26.7%) and 17.1–25.0% (mean = 21.6%, median = 21.3%, COX1 = 21.3%), respectively, with the lowest values observed for the rRNAs (Additional file 7: Table S3). On the intraspecific level, we here only compared some few individuals of the same species that were recovered as sister groups in the molecular phylogeny (i.e. *T. baskasti* 0827 vs. 6076, *T. helmuti* 0868 vs. 2716, *T. masapensis* 1335 vs. 1652, *Tylomelania* sp. ‘Patingko’ 0894 vs. 2494, *T. wesseli* 1630 vs. 1631, and *Tylomelania* sp. ‘Beau’ 2504 vs. 2584). Accordingly, the highest number of variable sites across all species pairs was found in ND5 (*N* = 30), followed by COX1 (*N* = 22), ND4 (*N* = 17), and ATP6 as well as COX3 (*N* = 13 each). In contrast, only low variation was found in ATP8 and ND4L (*N* = 3 each; Additional file 8: Table S4).

**Fig. 4:**
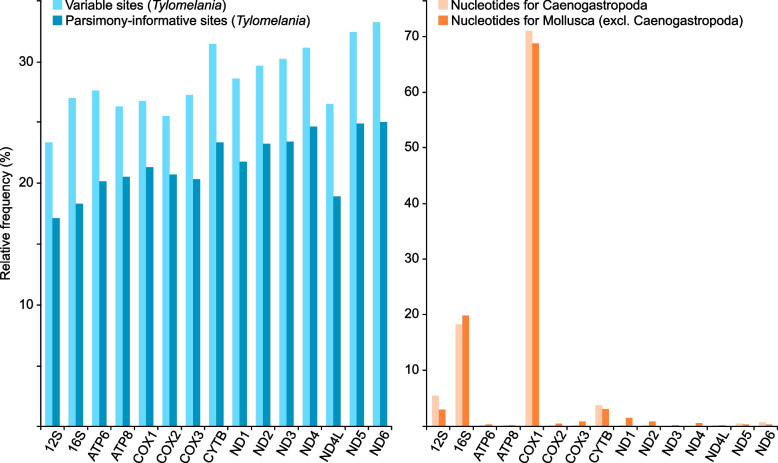
Mitochondrial gene representation: relative frequency of variable and parsimony-informative sites among the 77 *Tylomelania* mitogenomes finally analysed in the present study (left panel); relative frequency of caenogastropod nucleotides available in GenBank excluding complete mitogenomes (right panel)

Despite this considerable genetic variability, these mitochondrial markers are not used to the same extent as reflected by the number of nucleotides available in GenBank for both Caenogastropoda and Mollusca in general, with COX1 being the predominant locus, followed by 16S rRNA and 12S rRNA, and CYTB (Fig. 4 and Additional file 9: Table S5).

### Mitogenome arrangement

The arrangement of the 13 CDS and 2 rRNA genes is identical to the single published mitogenome of *T. sarasinorum* [54] but also to nine other published mitogenomes comprising six other caenogastropod families: Batillariidae, GenBank acc. no. NC_047187; Paludomidae, NC_045095; Potamididae, MZ168697 & NC_039951; Semisulcospiridae, NC_023364 & NC_037771; Thiaridae, MZ321058 & MZ662113; and Turritellidae, NC_029717.

## Discussion

In the present study, we provide, for the first time, mitogenomic information across a freshwater gastropod species flock at near-complete taxonomic level. By doing so, we were interested whether or not mitogenomes and their individual loci substantially improve the phylogenetic resolution of a comparatively young species assemblage endemic to Sulawesi’s ancient Malili lake system.

The present multi-locus phylogeny revealed several patterns already recognized in previous studies and, in large parts, also observed in other freshwater groups inhabiting this ecosystem. These include the presence of three Malili clades (M1–M3) plus a Lake Poso clade (P) and several cases of mitochondrial introgression between lacustrine species or lacustrine–riverine species (Fig. 2). Those findings are mainly congruent with a first comprehensive phylogeny based on COX1 and 16S rRNA [30] and a later 16S rRNA-derived phylogeny with a main focus on the lacustrine taxa [73]. Apparent major deviations in the position of one or a few specimens between gene trees (see e.g. the COX1–16S rRNA cophyloplot in Fig. 3) are, in fact, not supported. However, the present molecular phylogeny is substantially better supported than previous phylogenetic hypotheses (see [30, 73]) and thus at least, in parts, improved the resolution of interspecific relationships. This is probably not surprising given that the mitogenome behaves like a single locus (e.g. [2, 74]), and as such, nearly identical topologies were found among the different loci (see Fig. 3) that all contributed to the overall support. However, the higher number of characters did not affect the phylogeny in the same way, which is reflected in the poorly supported clades M2 and M3. We here argue that these short branches are related to rapid diversification events resulting in so-called ‘bottom-heavy’ clades typical for adaptive radiations ([75]; see also [76]).

In general, it is remarkable how many variable and parsimony-informative sites were identified among and within species across the remaining ‘non-traditional’ mitochondrial genes despite the comparatively young age of this species flock (Fig. 4, Additional file 6: Tables S2, and Additional file 7: Table S3). Mitogenomes therefore not only seem to be very powerful in a phylogenetic context at the family and superfamily level in molluscs (e.g. [2, 9–12]), but also further below. The nucleotide database of NCBI GenBank is very biased towards COX1, 12S rRNA and 16S rRNA and perhaps CYTB, whereas the remaining loci are only rarely used in both Caenogastropoda (to which the *Tylomelania* species flock belongs to) and Mollusca in general (Fig. 4). Obviously, this is not much of an issue, considering that all mitochondrial genes are part of a single locus (see above) and that the two genes applied previously to the *Tylomelania* system (COX1 and 16S rRNA) have essentially recovered the same topology as recovered by all other genes (see also above). However, the constant increase in WGS and lcWGS projects (see e.g. [5]) in combination with the suite of relatively easy-to-use mapping tools available nowadays (e.g. [60, 77–80]) will soon mitigate this bias and further provide a plethora of ‘non-traditional’ loci that will likely be relevant for a variety of biodiversity and phylogenetic studies beyond a classical DNA barcoding approach.

However, the very nature of such a young species assemblages as *Tylomelania*, in which interspecific, intralacustrine as well as lacustrine–riverine hybridisation and perhaps also incomplete lineage sorting play a major role, pushes mitogenome data to their limits (e.g. [1, 2] and references therein). Understanding the evolution of such a high biodiversity and morphological disparity (shells and radula forms) thus requires that the molecular phylogeny approximates the species tree (see e.g. [81]). In our study, the seemingly polyphyletic nature of most species and the pattern of lacustrine–riverine hybridisation found in the previous single-locus studies on *Tylomelania* have not been resolved by a mitogenome approach, indicating that our multi-locus gene tree is likely not a good approximation of the species tree. It might even be argued whether or not the only clear advantage we found in using near-complete mitogenomes rather than just two mitochondrial gene fragments on lacustrine *Tylomelania *is in fact a benefit. This means that a considerably better overall branch support actually becomes a disadvantage by giving a higher credibility to relationships that reflect the maternal inheritance of a single locus rather than introgression and ‘true’ species boundaries, particularly in these young groups (e.g. [82, 83]).

## Conclusions

Our analyses show that although mitochondrial data and even complete mitogenomes are essential for taxonomic studies and may provide a robust phylogenetic backbone, genome-wide data are key to shed light on the adaptive radiation and their underlying processes. This not only applies to the freshwater gastropods studied here, but also other non-model species flocks currently underrepresented in WGS studies. Future work should therefore also focus on these mainly invertebrate groups to identify potentially common factors that triggered diversification and speciation in those and other isolated ecosystems.

### Supplementary Information

**Additional file 1:** **Figure S1.** Map of Sulawesi showing Lake Poso (northwest) and the Malili lake system (southeast) including localities of the 78 individuals analysed. Inset maps show the location of Sulawesi in Indonesia and the study area in Sulawesi, respectively.

**Additional file 2: Figure S2.** Coverage plots among samples compared to the reference mitogenome of *T. sarasinorum* (NC_030263, top panel).

**Additional file 3: Figure S3.** Damage plots of unmerged mapped reads (before and after duplicate removal) among samples. Red and blue lines denote C to T and G to A misincorporations, respectively, towards the ends.

**Additional file 4: Figure S4.** Cophyloplots for the different mitochondrial loci analysed vs. the COX1 topology (left side of the plot).

**Additional file 5: Table S1.** Sample information for the 78 individuals processed including details on sequencing and mapping.

**Additional file 6: Table S2.** GenBank accession numbers for the samples finally analysed.

**Additional file 7: Table S3.** Details on interspecific variation among the 15 loci analysed using AMAS.

**Additional file 8: Table S4.** Details on intraspecific variation for selected taxa among the 15 loci analysed using AMAS.

**Additional file 9: Table S5.** Number of variable and parsimony-informative sites within *Tylomelania* among the 15 loci analysed using AMAS. Number of nucleotides available in GenBank for the 15 loci analysed.

## Data Availability

All data generated or analysed in this study are included in the article and its additional files. Newly generated sequences were submitted to the NCBI GenBank database under the accession numbers: PP525623–PP525699 (12S rRNA), PP525700–PP525776 (16S rRNA), PP531756–PP531832 (ATP6), PP531833–PP531909 (ATP8), PP515298–PP515374 (COX1), PP531910–PP531986 (COX2), PP531987–PP532063 (COX3), PP532064–PP532140 (CYTB), PP532141–PP532217 (ND1), PP532218–PP532294 (ND2), PP532295–PP532371 (ND3), PP532372–PP532448 (ND4), PP532449–PP532525 (ND4L), PP532526–PP532602 (ND5), and PP532603–PP532679 (ND6).

## References

[1] Blair C (2023). Organellar DNA continues to provide a rich source of information in the genomics era. Mol Ecol.

[2] Ghiselli F, Gomes-Dos-Santos A, Adema CM, Lopes-Lima M, Sharbrough J, Boore JL (2021). Molluscan mitochondrial genomes break the rules. Philos Trans R Soc London B.

[3] Bouchet P, Rocroi J-P, Hausdorf B, Kaim A, Kano Y, Nützel A (2017). Revised classification, nomenclator and typification of gastropod and monoplacophoran families. Malacologia.

[4] Sigwart JD, Lindberg DR, Chen C, Sun J (2021). Molluscan phylogenomics requires strategically selected genomes. Philos Trans R Soc London B.

[5] Gomes-dos-Santos A, Lopes-Lima M, Castro LFC, Froufe E (2020). Molluscan genomics: the road so far and the way forward. Hydrobiologia.

[6] Klein AH, Ballard KR, Storey KB, Motti CA, Zhao M, Cummins SF (2019). Multi-omics investigations within the Phylum Mollusca, Class Gastropoda: from ecological application to breakthrough phylogenomic studies. Brief Bioinform.

[7] Liu F, Li Y, Yu H, Zhang L, Hu J, Bao Z (2021). MolluscDB: an integrated functional and evolutionary genomics database for the hyper-diverse animal phylum Mollusca. Nucleic Acids Res.

[8] Yang Z, Zhang L, Hu J, Wang J, Bao Z, Wang S (2020). The evo-devo of molluscs: insights from a genomic perspective. Evol Dev.

[9] Grande C, Templado J, Zardoya R (2008). Evolution of gastropod mitochondrial genome arrangements. BMC Evol Biol.

[10] Stöger I, Schrödl M (2013). Mitogenomics does not resolve deep molluscan relationships (yet?). Mol Phylogenet Evol.

[11] Osca D, Templado J, Zardoya R (2015). Caenogastropod mitogenomics. Mol Phylogenet Evol.

[12] Varney RM, Brenzinger B, Malaquias MAE, Meyer CP, Schrödl M, Kocot KM (2021). Assessment of mitochondrial genomes for heterobranch gastropod phylogenetics. BMC Ecol Evol.

[13] Greenwood PH, Echelle AA, Kornfield I (1984). What is a species flock?. Evolution of fish species flocks.

[14] Brooks JL (1950). Speciation in ancient lakes. Q Rev Biol.

[15] Martens K, Goddeeris B, Coulter G (1994). Speciation in ancient lakes.

[16] Martens K (1997). Speciation in ancient lakes. Trends Ecol Evol.

[17] Sherbakov DY (1999). Molecular phylogenetic studies on the origin of biodiversity in Lake Baikal. Trends Ecol Evol.

[18] Cristescu ME, Adamowicz SJ, Vaillant JJ, Haffner GD (2010). Ancient lakes revisited: from the ecology to the genetics of speciation. Mol Ecol.

[19] von Rintelen T, von Rintelen K, Glaubrecht M, Schubart CD, Herder F, Gower DJ, Johnson KG, Richardson JE, Rosen BR, Rüber L, Williams ST (2012). Aquatic biodiversity hotspots in Wallacea: the species flocks in the ancient lakes of Sulawesi, Indonesia. Biotic evolution and environmental change in Southeast Asia.

[20] Salzburger W, Van Bocxlaer B, Cohen AS (2014). Ecology and evolution of the African Great Lakes and their faunas. Annu Rev Ecol Evol Syst.

[21] Wilke T, Hauffe T, Jovanovska E, Cvetkoska A, Donders T, Ekschmitt K (2020). Deep drilling reveals massive shifts in evolutionary dynamics after formation of ancient ecosystem. Sci Adv..

[22] Stelbrink B, Wilke T, Albrecht C (2020). Ecological opportunity enabled invertebrate radiations in ancient Lake Ohrid. J Great Lakes Res.

[23] Stroud JT, Losos JB (2016). Ecological opportunity and adaptive radiation. Annu Rev Ecol Evol Syst.

[24] Wagner CE, Harmon LJ, Seehausen O (2012). Ecological opportunity and sexual selection together predict adaptive radiation. Nature.

[25] Salzburger W, Meyer A (2004). The species flocks of East African cichlid fishes: recent advances in molecular phylogenetics and population genetics. Naturwissenschaften.

[26] Cohen AS, Stone JR, Beuning KRM, Park LE, Reinthal PN, Dettman D (2007). Ecological consequences of early Late Pleistocene megadroughts in tropial Africa. Proc Natl Acad Sci USA.

[27] Schultheiß R, Van Bocxlaer B, Wilke T, Albrecht C (2009). Old fossils – young species: evolutionary history of an endemic gastropod assemblage in Lake Malawi. Proc R Soc London B.

[28] Michel E. Why snails radiate: a review of gastropod evolution in long-lived lakes, both recent and fossil. In: Speciation in Ancient Lakes. Stuttgart: E. Schweitzerbart’sche Verlagsbuchhandlung; 1994:284–317.

[29] Rüber L, Verheyen E, Meyer A (1999). Replicated evolution of trophic specializations in an endemic cichlid fish lineage from Lake Tanganyika. Proc Natl Acad Sci USA.

[30] von Rintelen T, Wilson AB, Meyer A, Glaubrecht M (2004). Escalation and trophic specialization drive adaptive radiation of viviparous freshwater gastropods in the ancient lakes on Sulawesi, Indonesia. Proc R Soc London B.

[31] von Rintelen K, Glaubrecht M, Schubart CD, Wessel A, von Rintelen T (2010). Adaptive radiation and ecological diversification of Sulawesi’s ancient lake shrimps. Evolution.

[32] Pfaender J, Miesen FW, Hadiaty RK, Herder F (2011). Adaptive speciation and sexual dimorphism contribute to diversity in form and function in the adaptive radiation of Lake Matano’s sympatric roundfin sailfin silversides. J Evol Biol.

[33] Ronco F, Matschiner M, Böhne A, Boila A, Büscher HH, El Taher A (2021). Drivers and dynamics of a massive adaptive radiation in cichlid fishes. Nature.

[34] Herder F, Nolte AW, Pfaender J, Schwarzer J, Hadiaty RK, Schliewen UK (2006). Adaptive radiation and hybridization in Wallace’s dreamponds: evidence from sailfin silversides in the Malili lakes of Sulawesi. Proc R Soc London B.

[35] Seehausen O (2006). African cichlid fish: a model system in adaptive radiation research. Proc R Soc London B.

[36] Gante HF, Matschiner M, Malmstrøm M, Jakobsen KS, Jentoft S, Salzburger W (2016). Genomics of speciation and introgression in Princess cichlid fishes from Lake Tanganyika. Mol Ecol.

[37] Meier JI, Marques DA, Mwaiko S, Wagner CE, Excoffier L, Seehausen O (2017). Ancient hybridization fuels rapid cichlid fish adaptive radiations. Nat Commun.

[38] Malinsky M, Svardal H, Tyers AM, Miska EA, Genner MJ, Turner GF (2018). Whole genome sequences of Malawi cichlids reveal multiple radiations interconnected by gene flow. Nat Ecol Evol.

[39] Seehausen O (2004). Hybridization and adaptive radiation. Trends Ecol Evol.

[40] Abbott R, Albach D, Ansell S, Arntzen JW, Baird SJE, Bierne N (2013). Hybridization and speciation. J Evol Biol.

[41] Marques DA, Meier JI, Seehausen O (2019). A combinatorial view on speciation and adaptive radiation. Trends Ecol Evol.

[42] Salzburger W (2018). Understanding explosive diversification through cichlid fish genomics. Nat Rev Genet.

[43] Svardal H, Quah FX, Malinsky M, Ngatunga BP, Miska EA, Salzburger W (2020). Ancestral hybridization facilitated species diversification in the Lake Malawi cichlid fish adaptive radiation. Mol Biol Evol.

[44] Hampton SE, McGowan S, Ozersky T, Virdis SGP, Vu TT, Spanbauer TL (2018). Recent ecological change in ancient lakes. Limnol Oceanogr.

[45] von Rintelen T, von Rintelen K, Glaubrecht M, Glaubrecht M (2010). The species flocks of the viviparous freshwater gastropod *Tylomelania* (Mollusca: Cerithioidea: Pachychilidae) in the ancient lakes of Sulawesi, Indonesia: the role of geography, trophic morphology and color as driving forces in adaptive radiation. Evolution in Action.

[46] Schluter D (2000). The ecology of adaptive radiation.

[47] von Rintelen T, Stelbrink B, Marwoto RM, Glaubrecht M (2014). A snail perspective on the biogeography of Sulawesi, Indonesia: origin and intra-island dispersal of the viviparous freshwater gastropod * Tylomelania*. PLoS ONE.

[48] Albrecht C, Stelbrink B, Gauffre-Autelin P, Marwoto RM, von Rintelen T, Glaubrecht M (2020). Diversification of epizoic freshwater limpets in ancient lakes on Sulawesi, Indonesia: coincidence or coevolution?. J Great Lakes Res.

[49] Russell JM, Vogel H, Bijaksana S, Melles M, Deino A, Hafidz A (2020). The late Quaternary tectonic, biogeochemical, and environmental evolution of ferruginous Lake Towuti. Indonesia Palaeogeogr Palaeoclimatol Palaeoecol..

[50] Muschick M, Indermaur A, Salzburger W (2012). Convergent evolution within an adaptive radiation of cichlid fishes. Curr Biol.

[51] Verheyen E, Salzburger W, Snoeks J, Meyer A (2003). Origin of the superflock of cichlid fishes from Lake Victoria, East Africa. Science.

[52] Meier JI, McGee MD, Marques DA, Mwaiko S, Kishe M, Wandera S (2023). Cycles of fusion and fission enabled rapid parallel adaptive radiations in African cichlids. Science..

[53] Stelbrink B, Stöger I, Hadiaty RK, Schliewen UK, Herder F (2014). Age estimates for an adaptive lake fish radiation, its mitochondrial introgression, and an unexpected sister group: sailfin silversides of the Malili Lakes system in Sulawesi. BMC Evol Biol.

[54] Hilgers L, Grau JH, Pfaender J, von Rintelen T (2016). The complete mitochondrial genome of the viviparous freshwater snail *Tylomelania sarasinorum* (Caenogastropoda: Cerithioidea). Mitochondrial DNA Part B.

[55] Hilgers L, Hartmann S, Hofreiter M, von Rintelen T (2018). Novel genes, ancient genes and gene co-option contributed to the genetic basis of the radula, a molluscan innovation. Mol Biol Evol.

[56] Hilgers L, Hartmann S, Pfaender J, Lentge-Maaß N, Marwoto RM, von Rintelen T (2022). Evolutionary divergence and radula diversification in two ecomorphs from an adaptive radiation of freshwater snails. Genes.

[57] Winnepenninckx B, Backeljau T, De Wachter R (1993). Extraction of high molecular weight DNA from molluscs. Trends Genet.

[58] Bolger AM, Lohse M, Usadel B (2014). Trimmomatic: a flexible trimmer for Illumina sequence data. Bioinformatics.

[59] Li H, Durbin R (2009). Fast and accurate short read alignment with Burrows-Wheeler transform. Bioinformatics.

[60] Li H. Aligning sequence reads, clone sequences and assembly contigs with BWA-MEM. 2013.

[61] Li H, Handsaker B, Wysoker A, Fennell T, Ruan J, Homer N (2009). The sequence alignment/map format and SAMtools. Bioinformatics.

[62] McKenna A, Hanna M, Banks E, Sivachenko A, Cibulskis K, Kernytsky A (2010). The Genome Analysis Toolkit: a MapReduce framework for analyzing next-generation DNA sequencing data. Genome Res.

[63] Van der Auwera GA, O’Connor BD. Genomics in the cloud: using Docker, GATK, and WDL in Terra. O’Reilly; 2020.

[64] Katoh K, Standley DM (2013). MAFFT multiple sequence alignment software version 7: improvements in performance and usability. Mol Biol Evol.

[65] Neukamm J, Peltzer A, Nieselt K (2021). DamageProfiler: fast damage pattern calculation for ancient DNA. Bioinformatics.

[66] Gilbert MTP, Bandelt H-J, Hofreiter M, Barnes I (2005). Assessing ancient DNA studies. Trends Ecol Evol.

[67] Briggs AW, Stenzel U, Johnson PLF, Green RE, Kelso J, Prüfer K (2007). Patterns of damage in genomic DNA sequences from a Neandertal. Proc Natl Acad Sci USA.

[68] Borowiec ML (2016). AMAS: a fast tool for alignment manipulation and computing of summary statistics. PeerJ..

[69] Minh BQ, Schmidt HA, Chernomor O, Schrempf D, Woodhams MD, von Haeseler A (2020). IQ-TREE 2: new models and efficient methods for phylogenetic inference in the genomic era. Mol Biol Evol.

[70] Nguyen LT, Schmidt HA, Von Haeseler A, Minh BQ (2015). IQ-TREE: a fast and effective stochastic algorithm for estimating maximum-likelihood phylogenies. Mol Biol Evol.

[71] Revell LJ (2012). phytools: an R package for phylogenetic comparative biology (and other things). Methods Ecol Evol.

[72] R Core Team. R: a language and environment for statistical computing. 2021. Vienna, Austria: R Foundation for Statistical Computing.

[73] von Rintelen T, Glaubrecht M (2008). Three new species of the freshwater snail genus
* Tylomelania
* (Caenogastropoda: Pachychilidae) from the Malili lake system, Sulawesi, Indonesia. Zootaxa.

[74] Zink RM, Barrowclough GF (2008). Mitochondrial DNA under siege in avian phylogeography. Mol Ecol.

[75] Gavrilets S, Vose A (2005). Dynamic patterns of adaptive radiation. Proc Natl Acad Sci USA.

[76] Gould SJ, Gilinsky NL, German RZ (1987). Asymmetry of lineages and the direction of evolutionary time. Science.

[77] Al-Nakeeb K, Petersen TN, Sicheritz-Pontén T (2017). Norgal: extraction and *de novo* assembly of mitochondrial DNA from whole-genome sequencing data. BMC Bioinformatics.

[78] Meng G, Li Y, Yang C, Liu S (2019). MitoZ: a toolkit for animal mitochondrial genome assembly, annotation and visualization. Nucleic Acids Res..

[79] Hahn C, Bachmann L, Chevreux B (2013). Reconstructing mitochondrial genomes directly from genomic next-generation sequencing reads – a baiting and iterative mapping approach. Nucleic Acids Res.

[80] Song M-H, Yan C, Li J-T (2022). MEANGS: an efficient seed-free tool for *de novo* assembling animal mitochondrial genome using whole genome NGS data. Brief Bioinform.

[81] Meyer BS, Matschiner M, Salzburger W (2017). Disentangling incomplete lineage sorting and introgression to refine species-tree estimates for Lake Tanganyika cichlid fishes. Syst Biol.

[82] Moore WS (1995). Inferring phylogenies from mtDNA variation: mitochondrial-gene trees versus nuclear-gene trees. Evolution.

[83] Sloan DB, Havird JC, Sharbrough J (2017). The on-again, off-again relationship between mitochondrial genomes and species boundaries. Mol Ecol.

