# Supplementary figures and images for: Mitogenomes do not substantially improve phylogenetic resolution in a young non-model adaptive radiation of freshwater gastropods
Source: BMC Ecol Evol. 2024 Apr 8;24:42. doi: 10.1186/s12862-024-02235-0 (PMC11000327; doi:10.1186/s12862-024-02235-0)

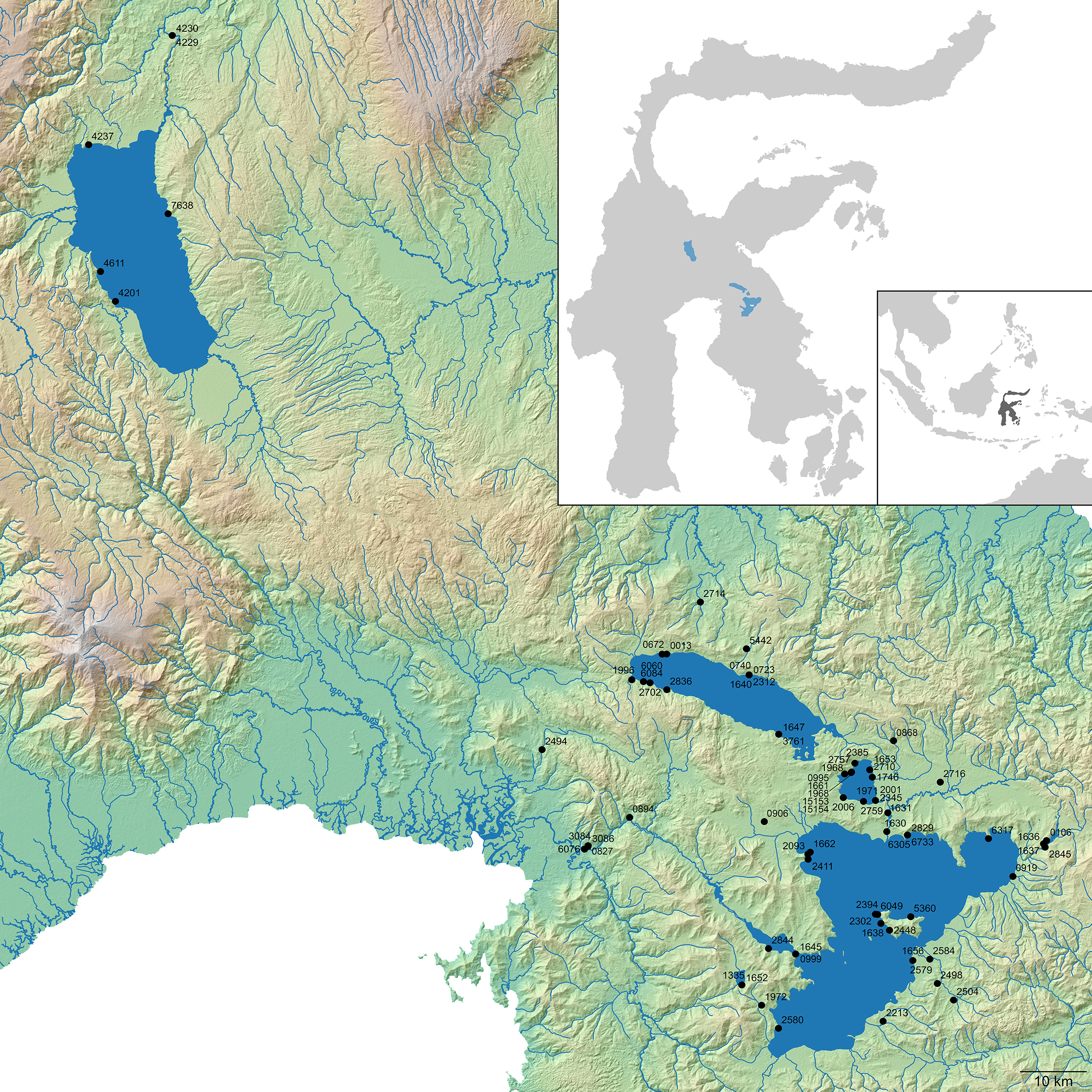

Supplement: Supplementary file 1 — Additional file 1: Figure S1. Map of Sulawesi showing Lake Poso (northwest) and the Malili lake system (southeast) including localities of the 78 individuals analysed. Inset maps show the location of Sulawesi in Indonesia and the study area in Sulawesi, respectively. [file 12862_2024_2235_MOESM1_ESM.jpg]

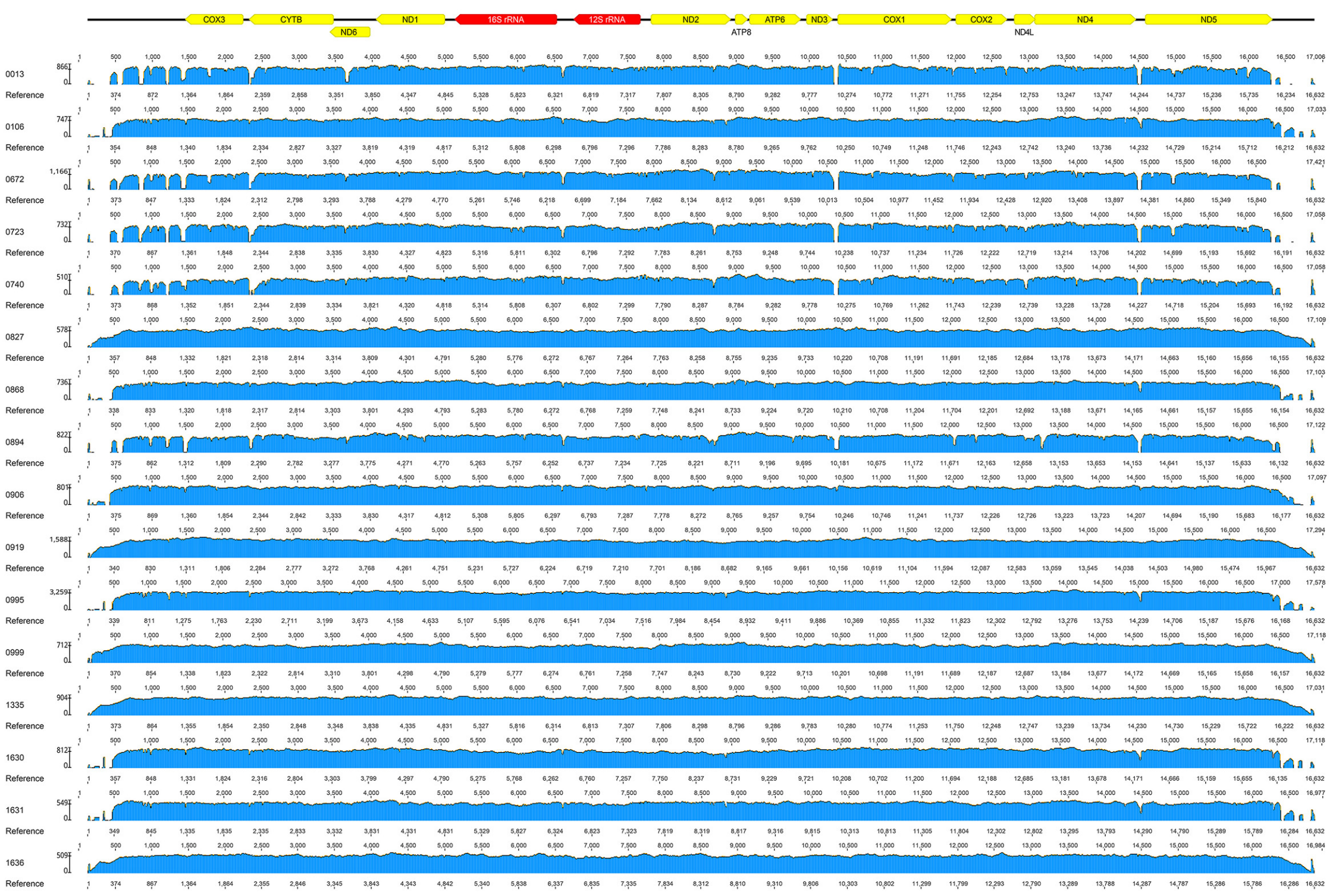

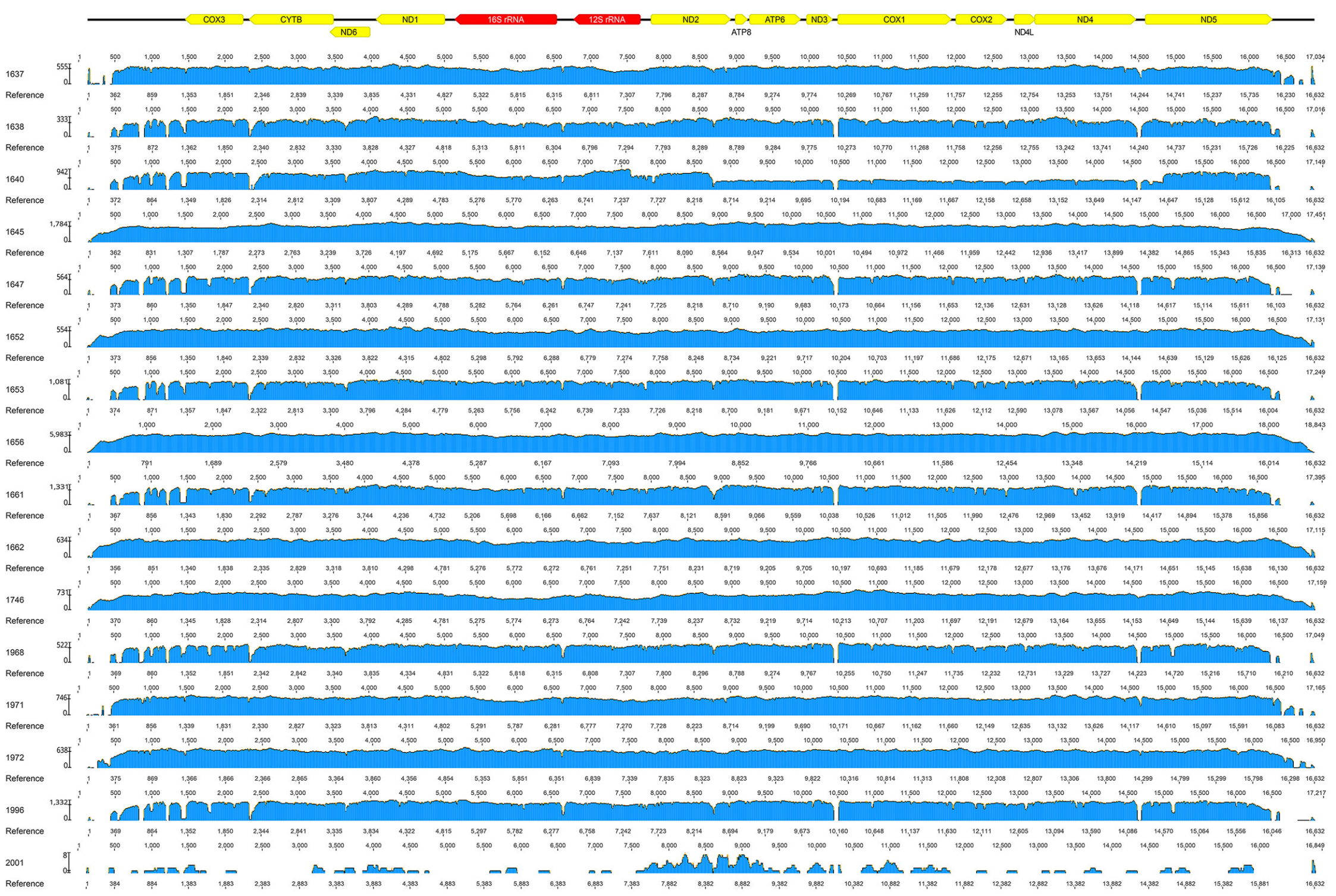

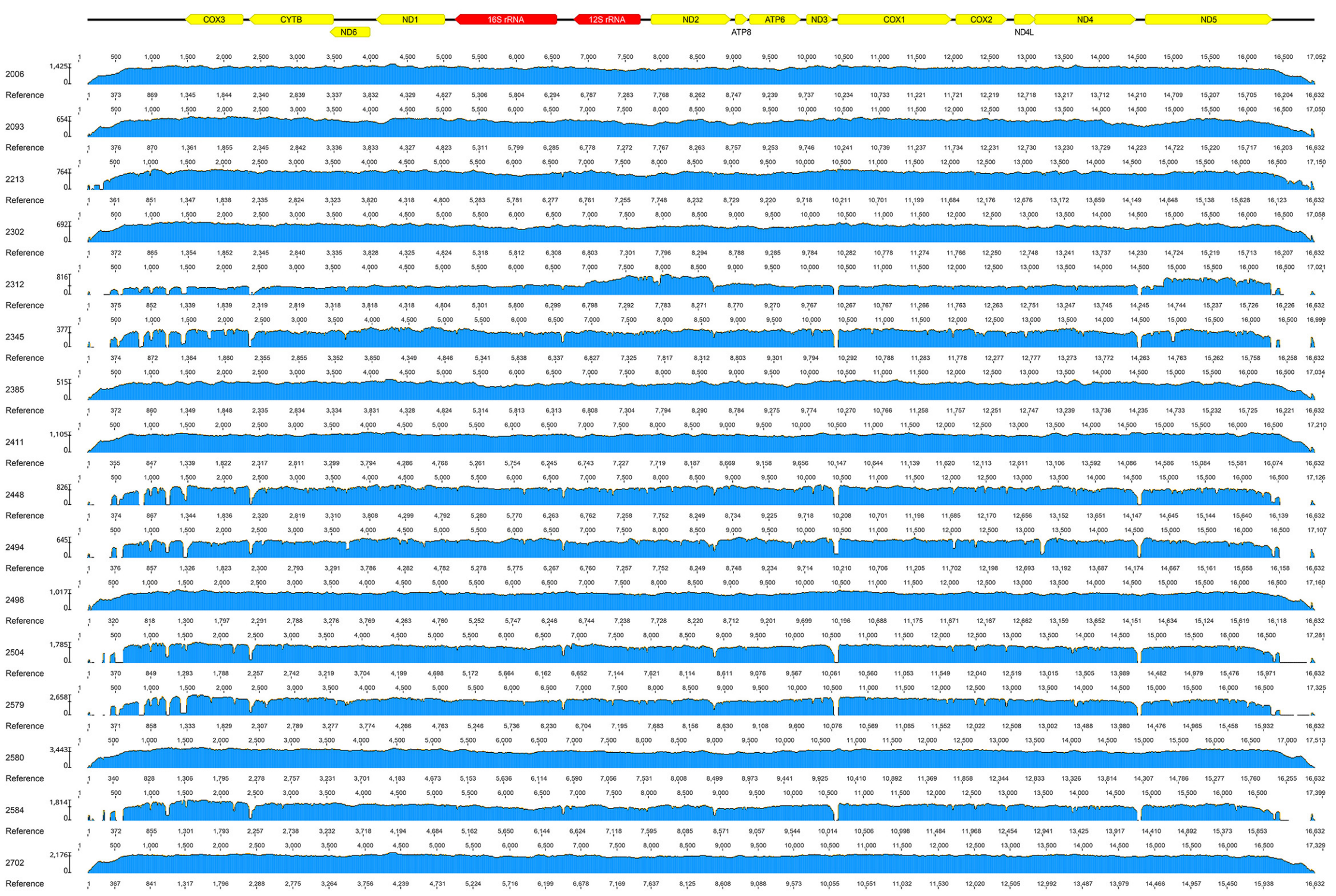

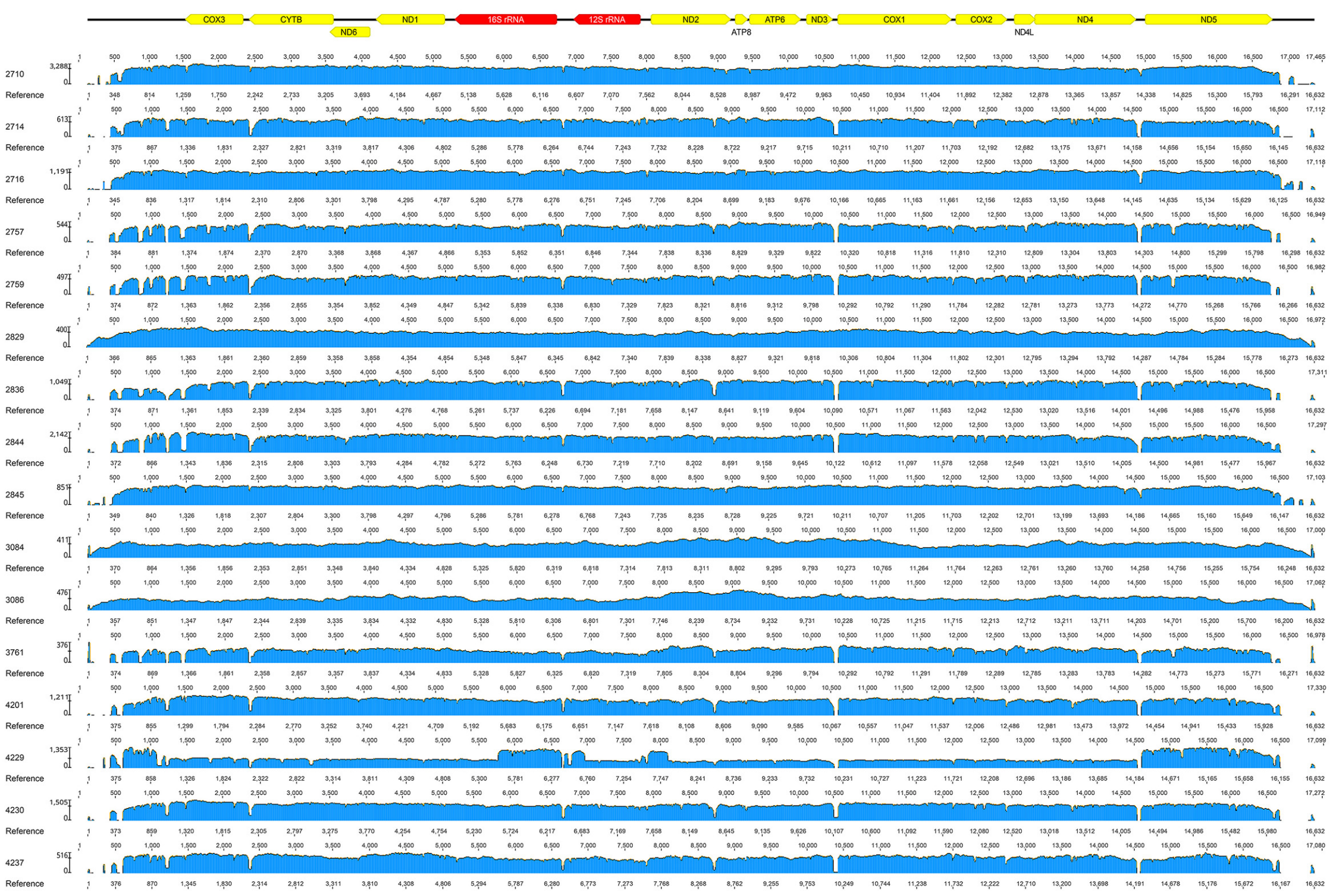

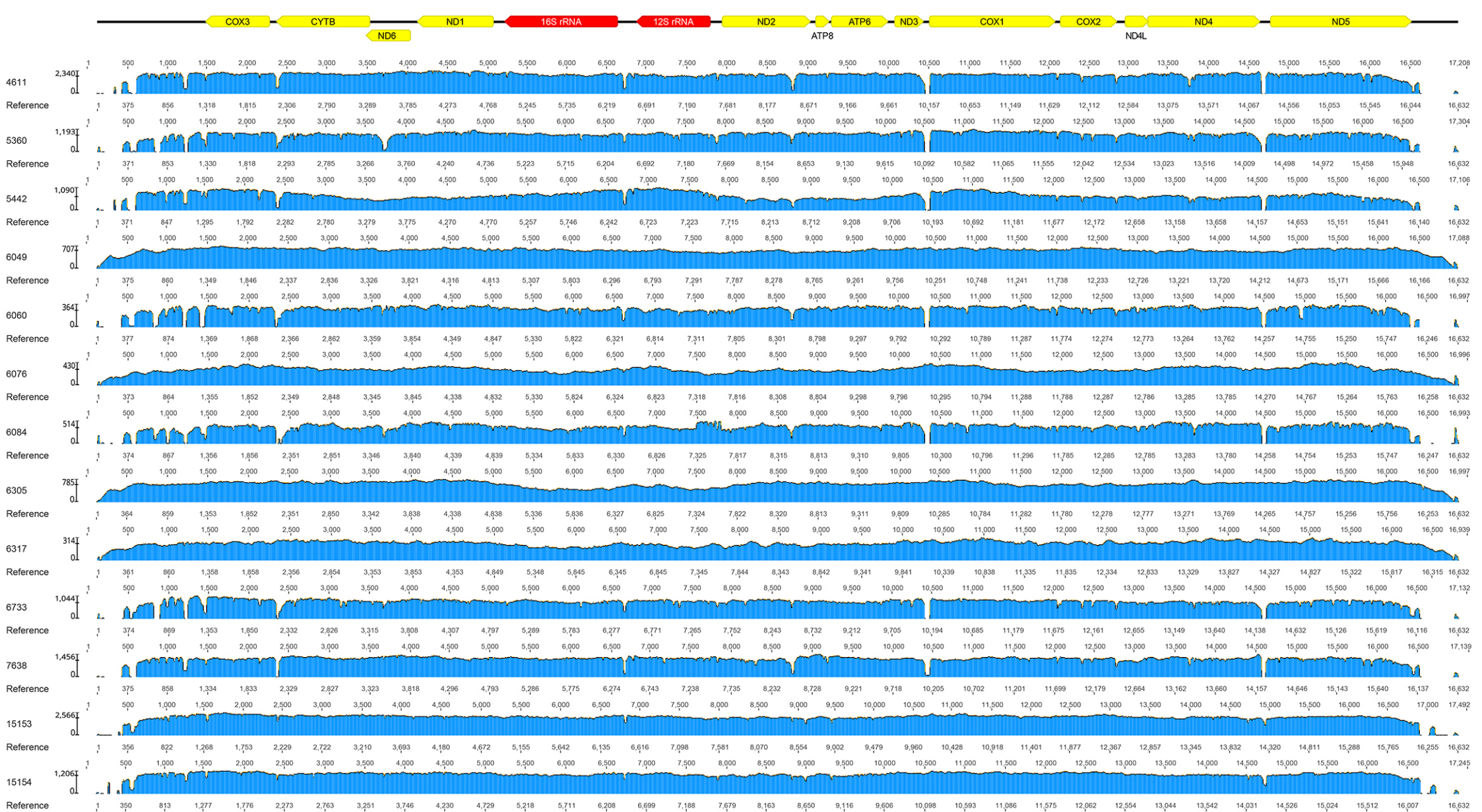

Supplement: Supplementary file 2 — Additional file 2: Figure S2. Coverage plots among samples compared to the reference mitogenome of T. sarasinorum (NC_030263, top panel). [file 12862_2024_2235_MOESM2_ESM.pdf]

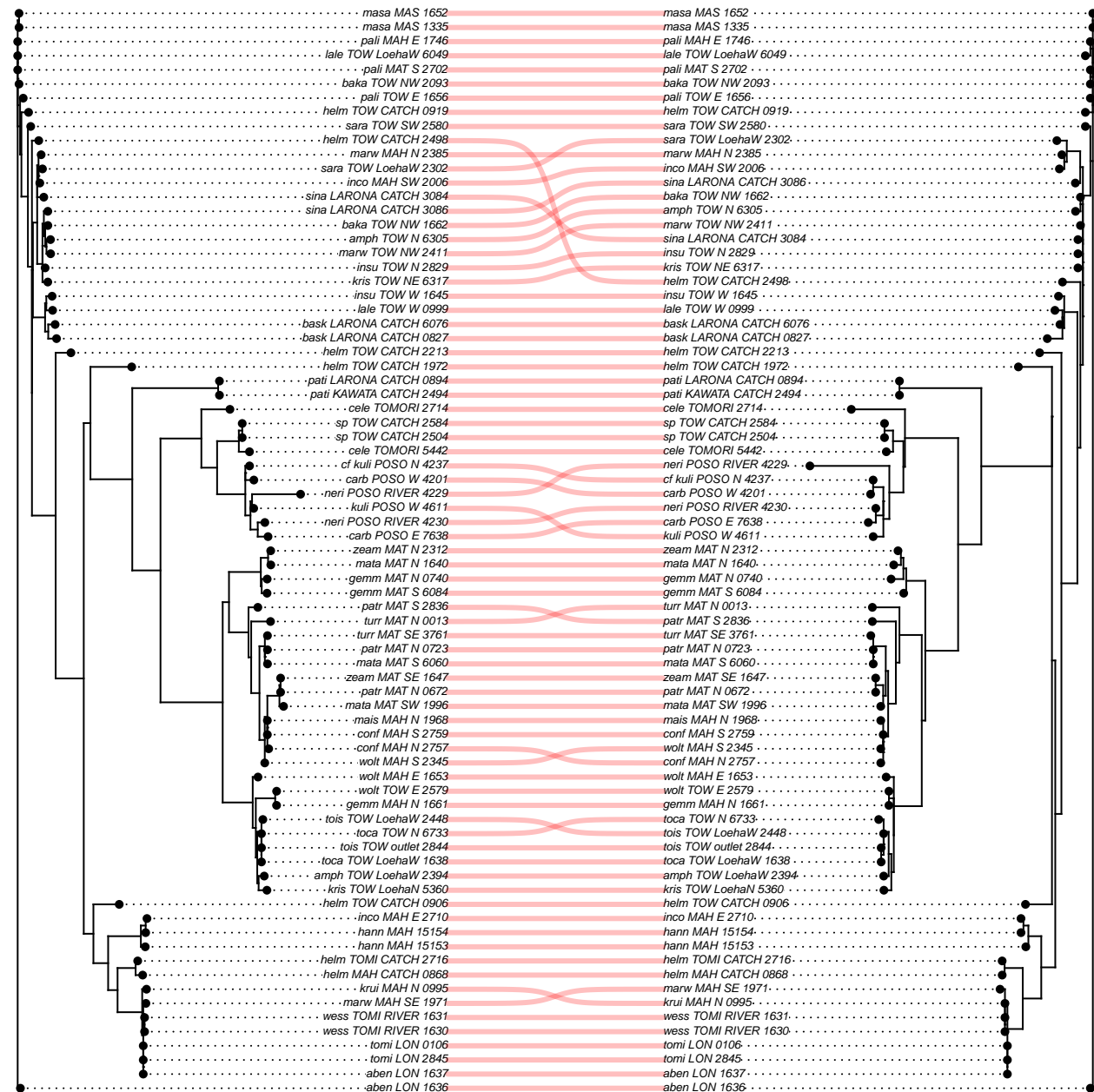

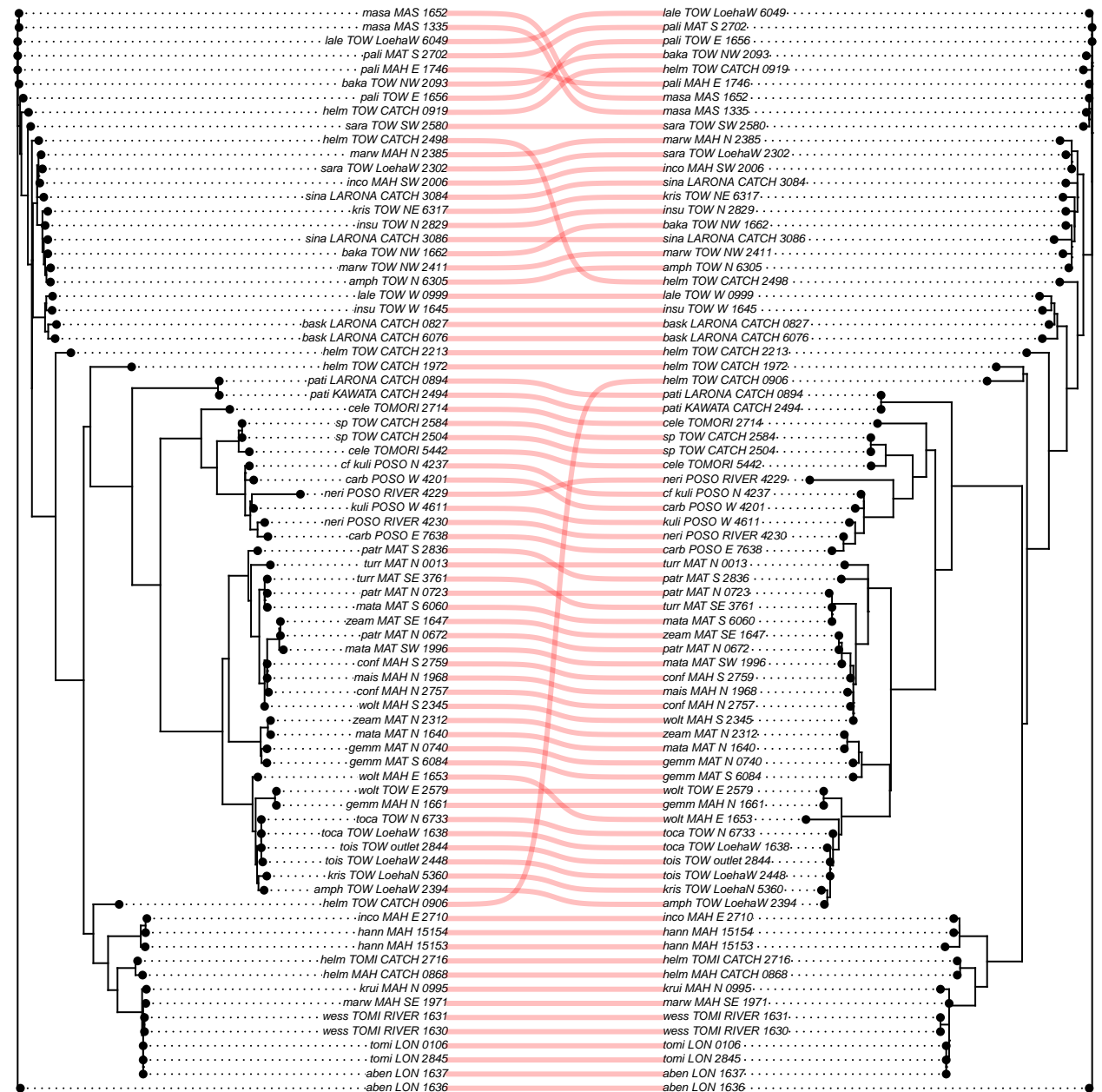

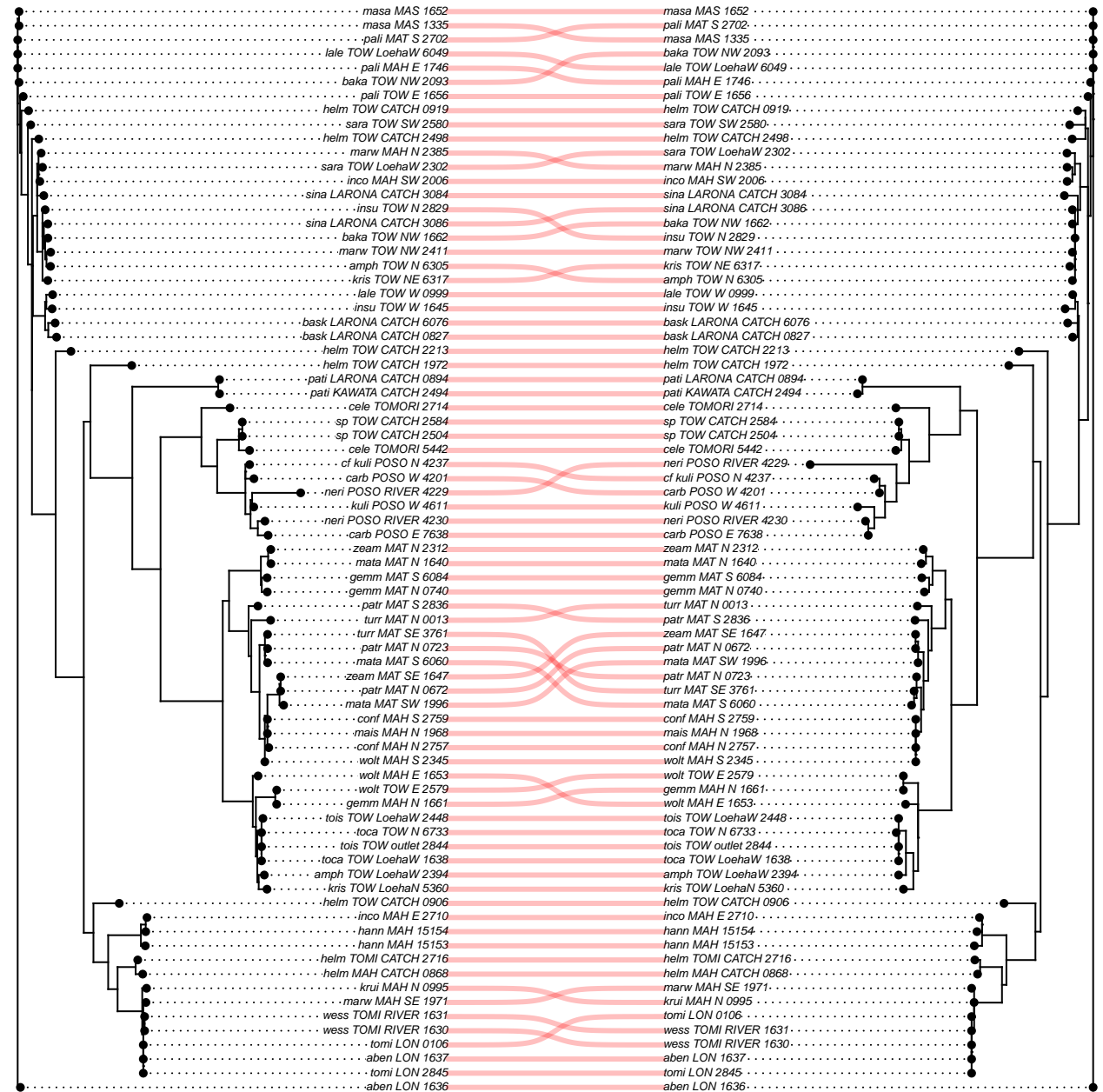

COX1

ATP8

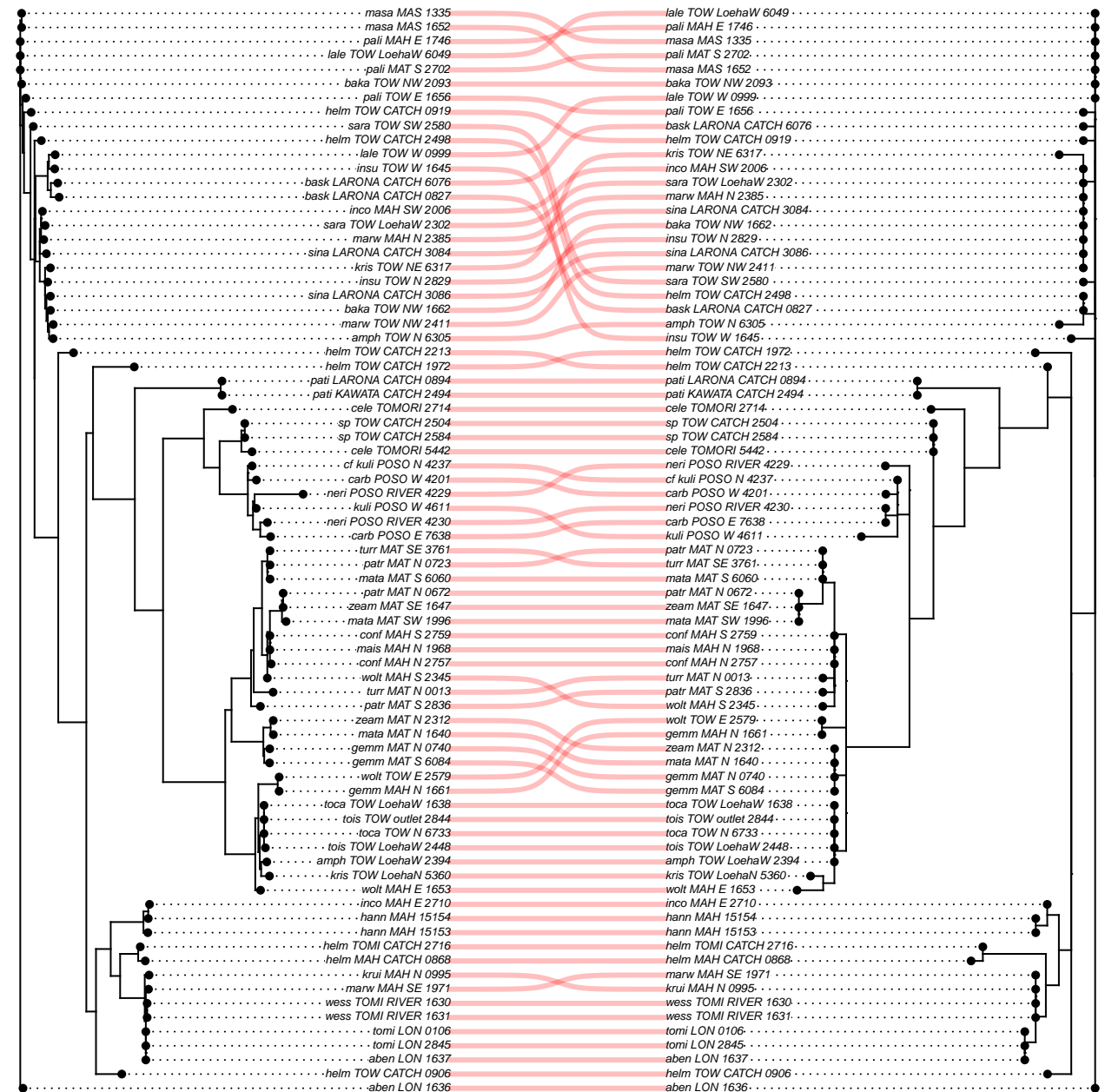

COX1

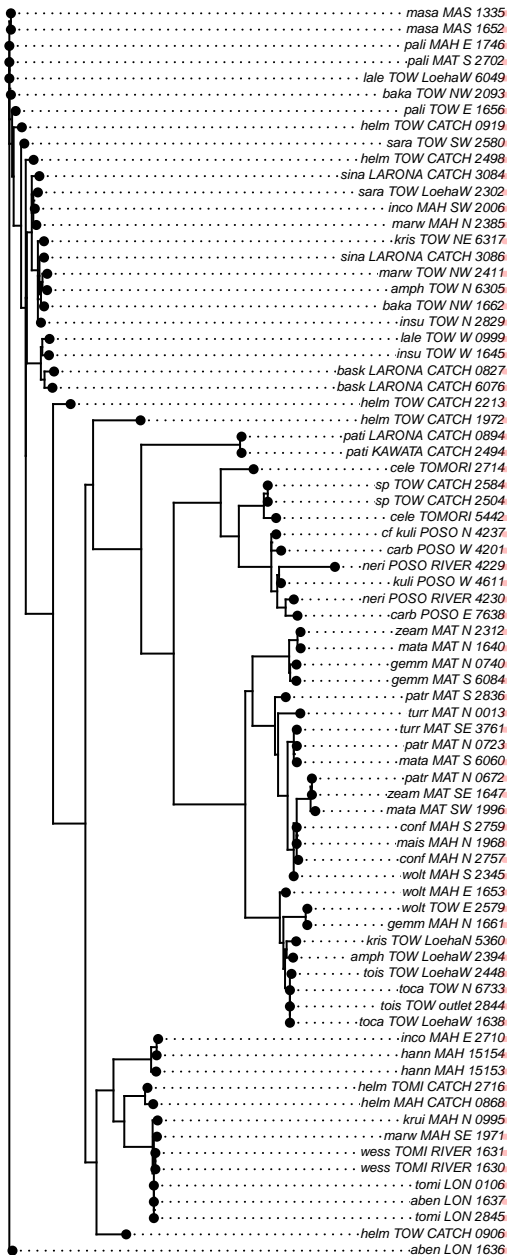

COX2

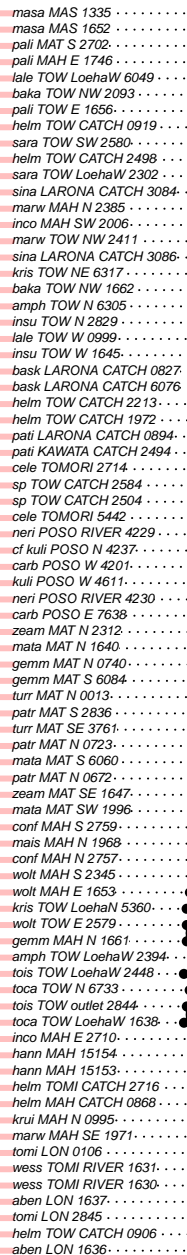

COX1

COX3

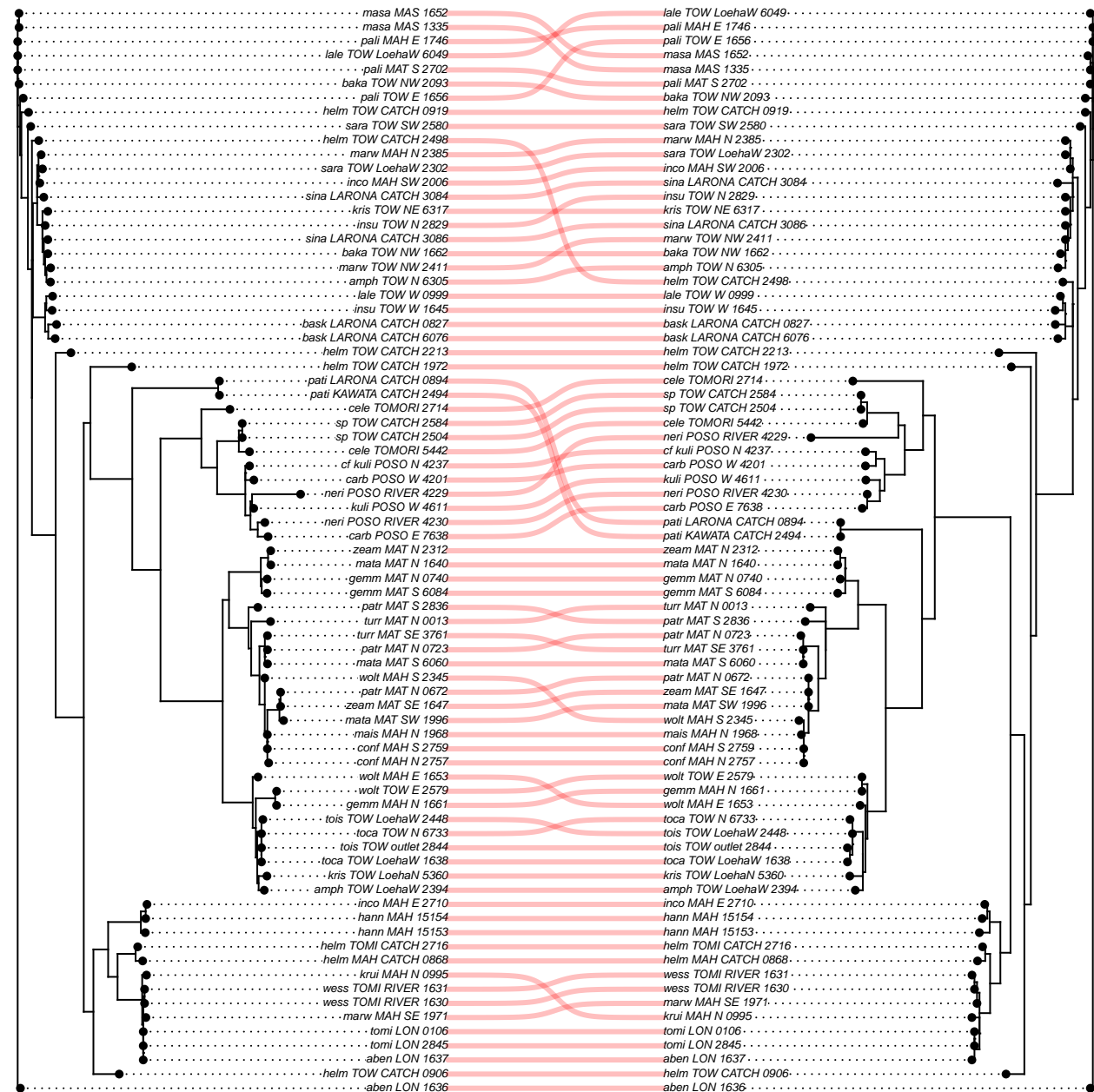

COX1

CYTB

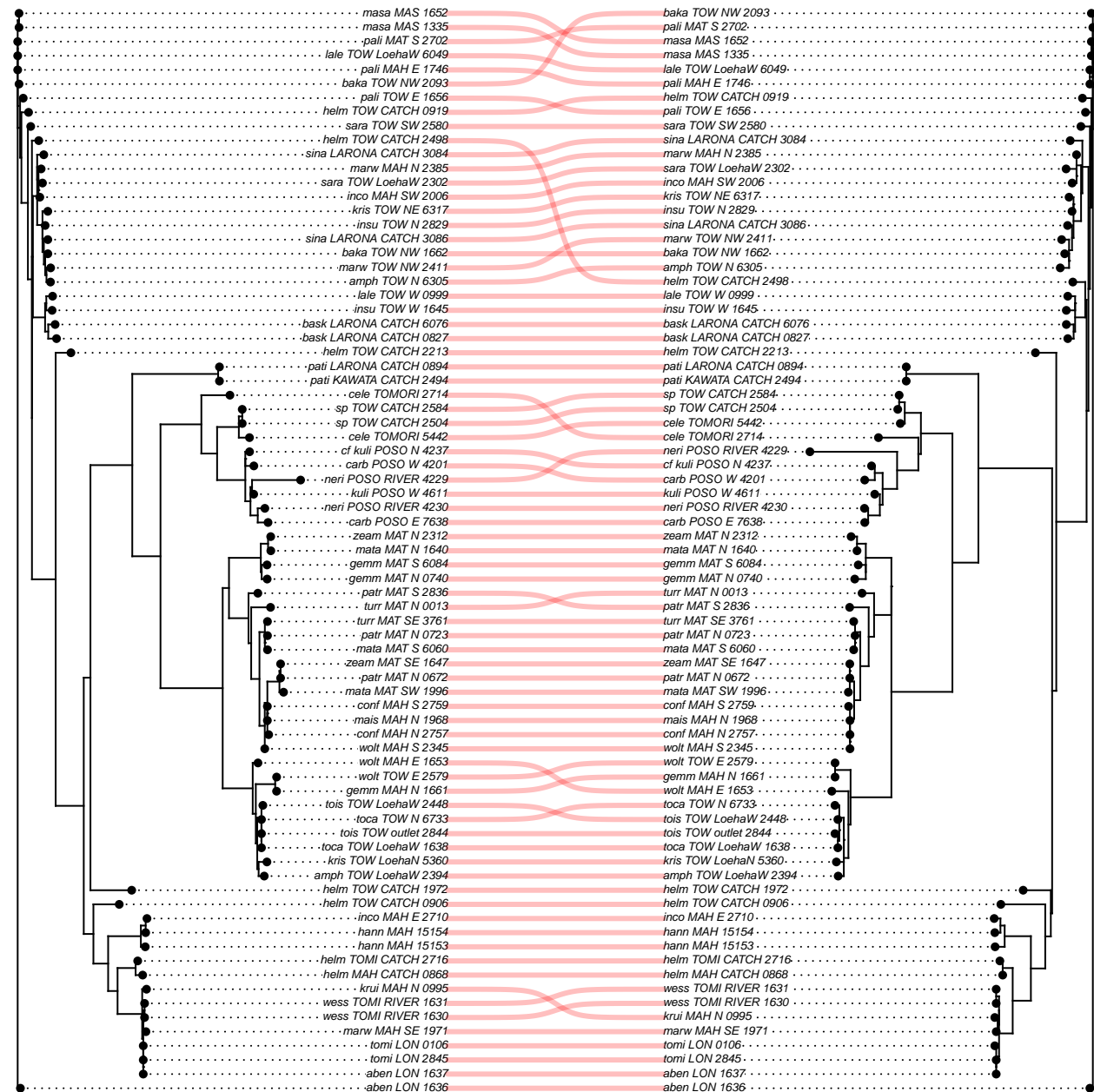

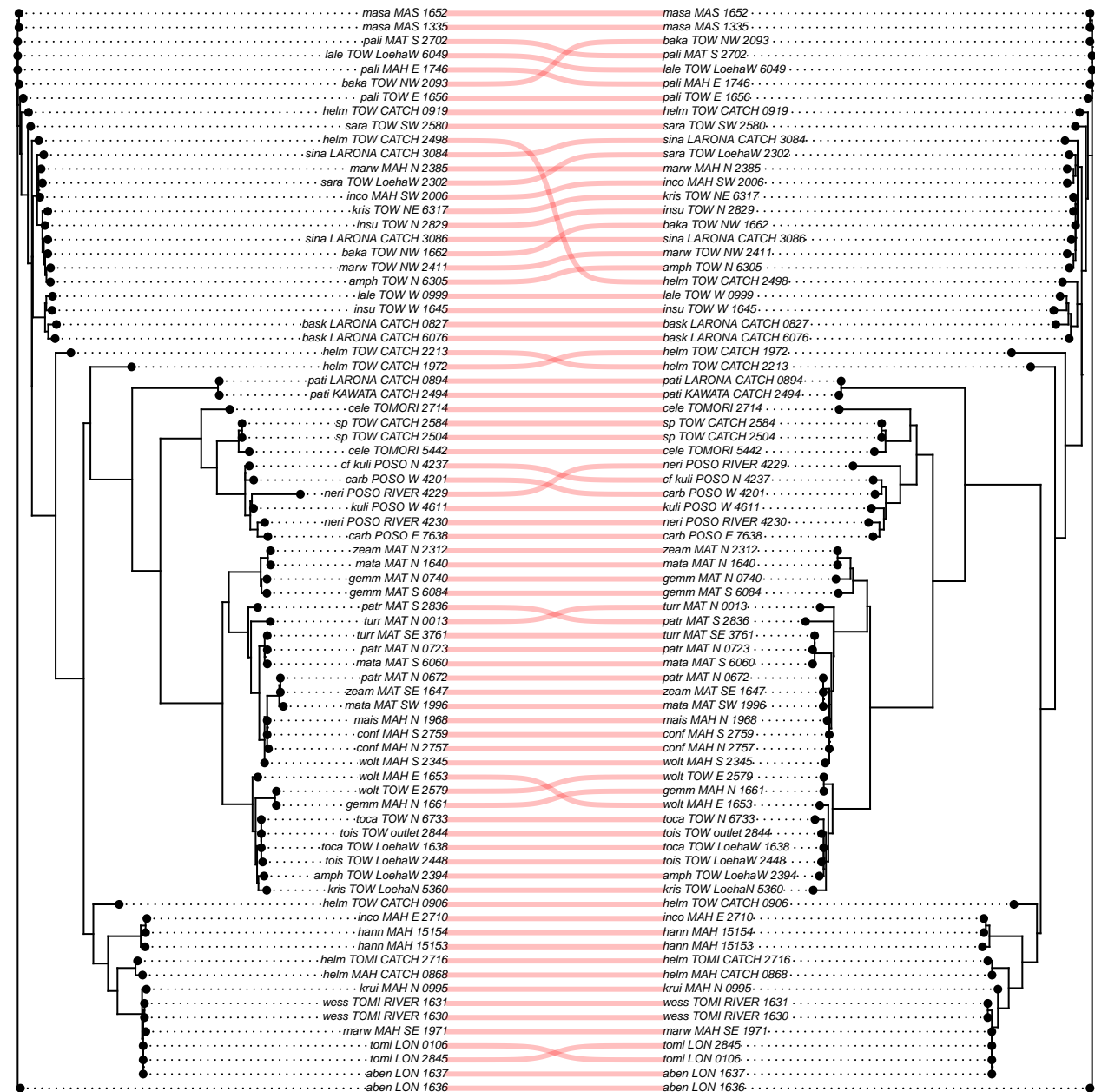

COX1

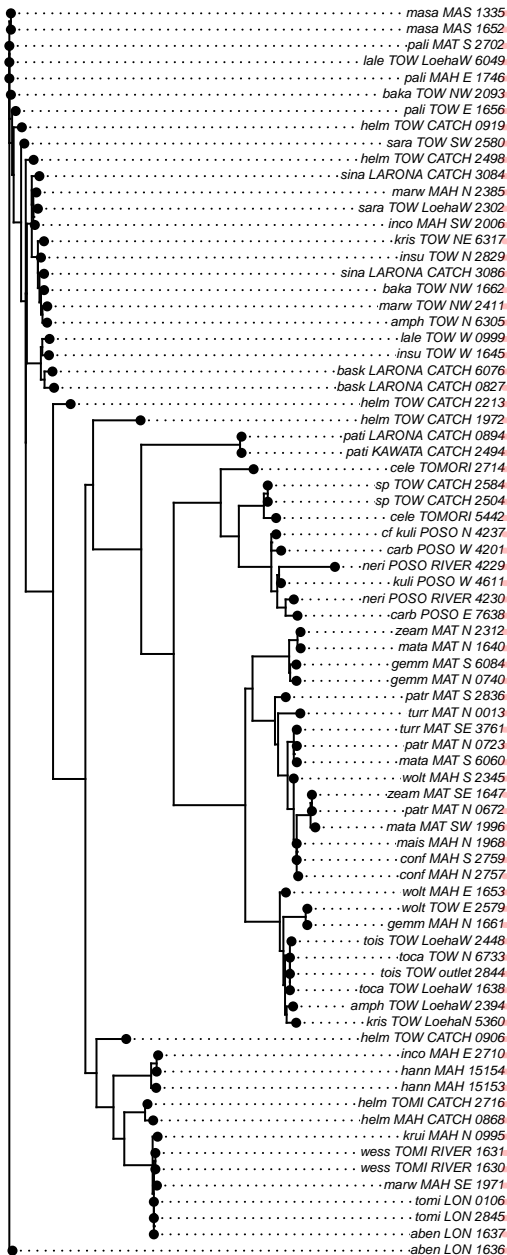

ND2

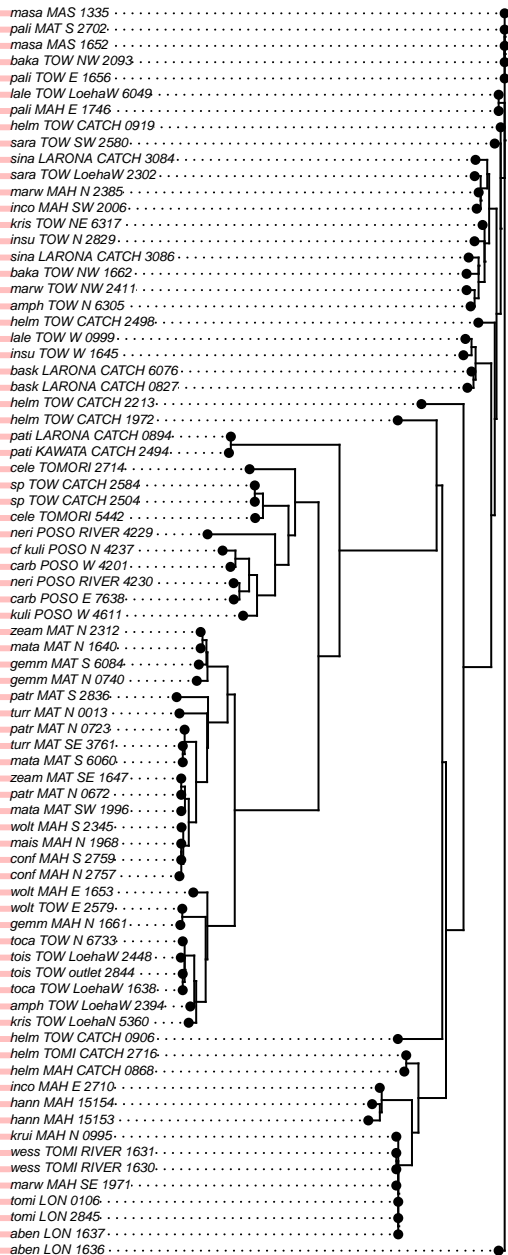

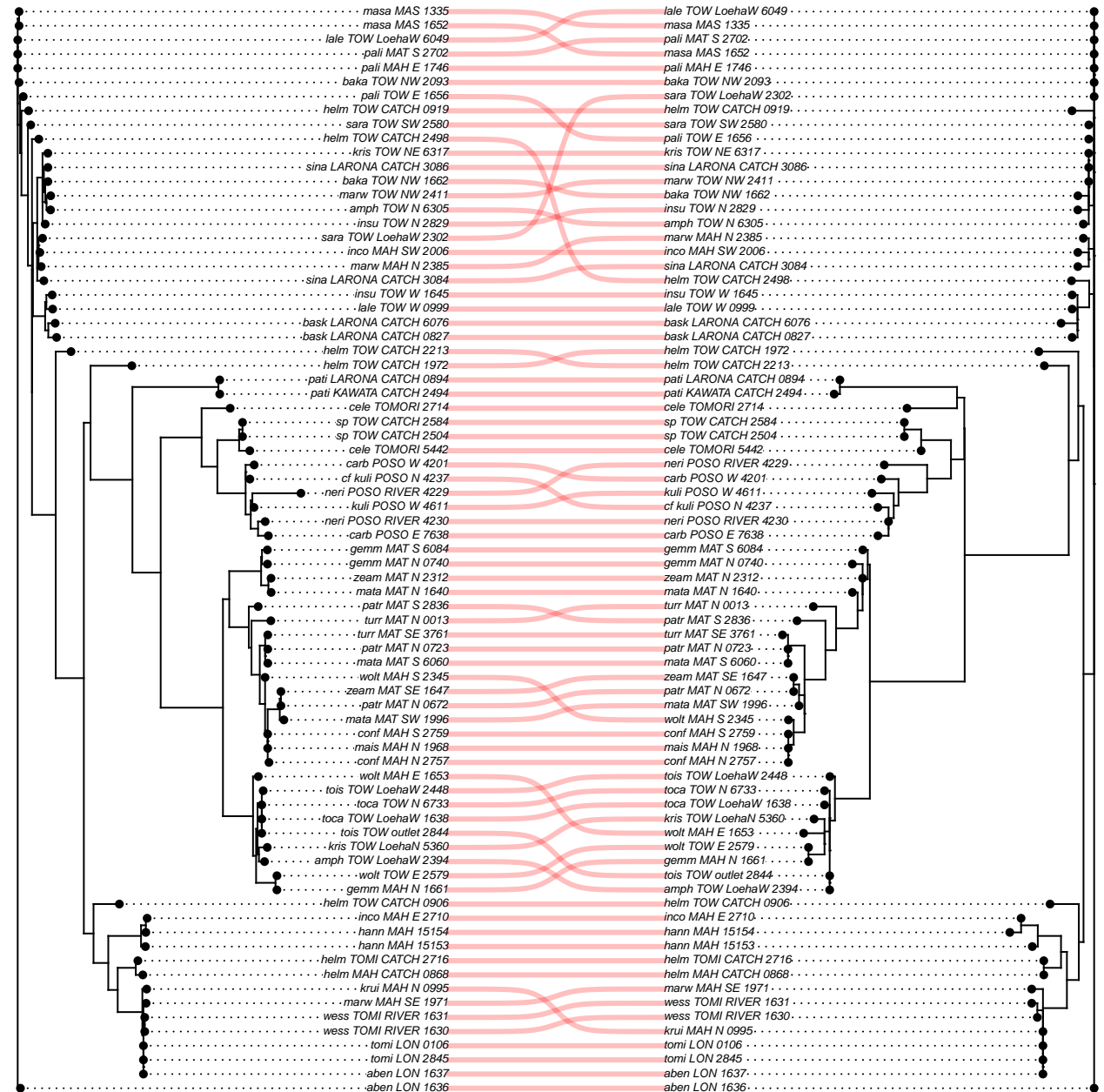

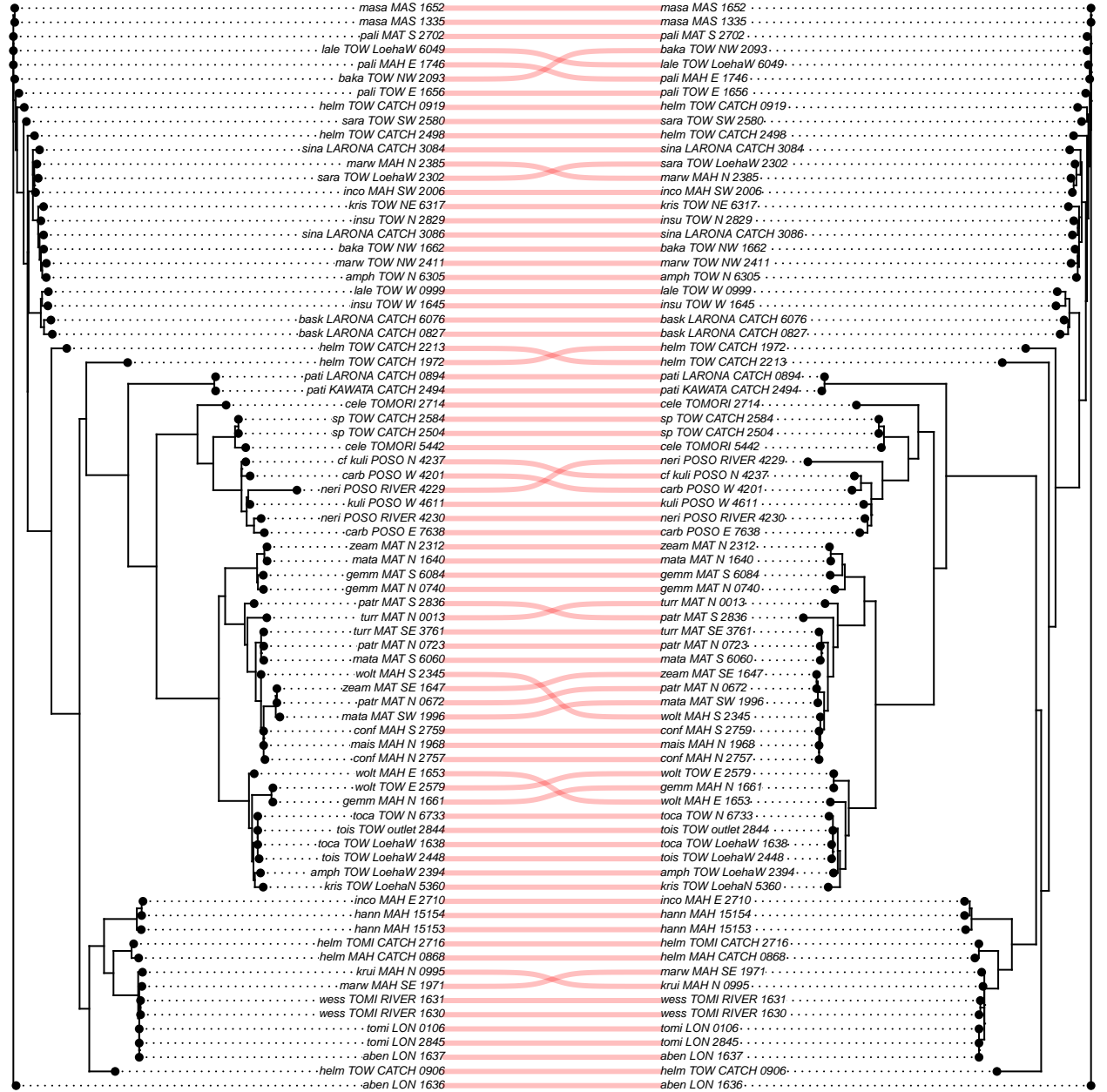

COX1

ND4L

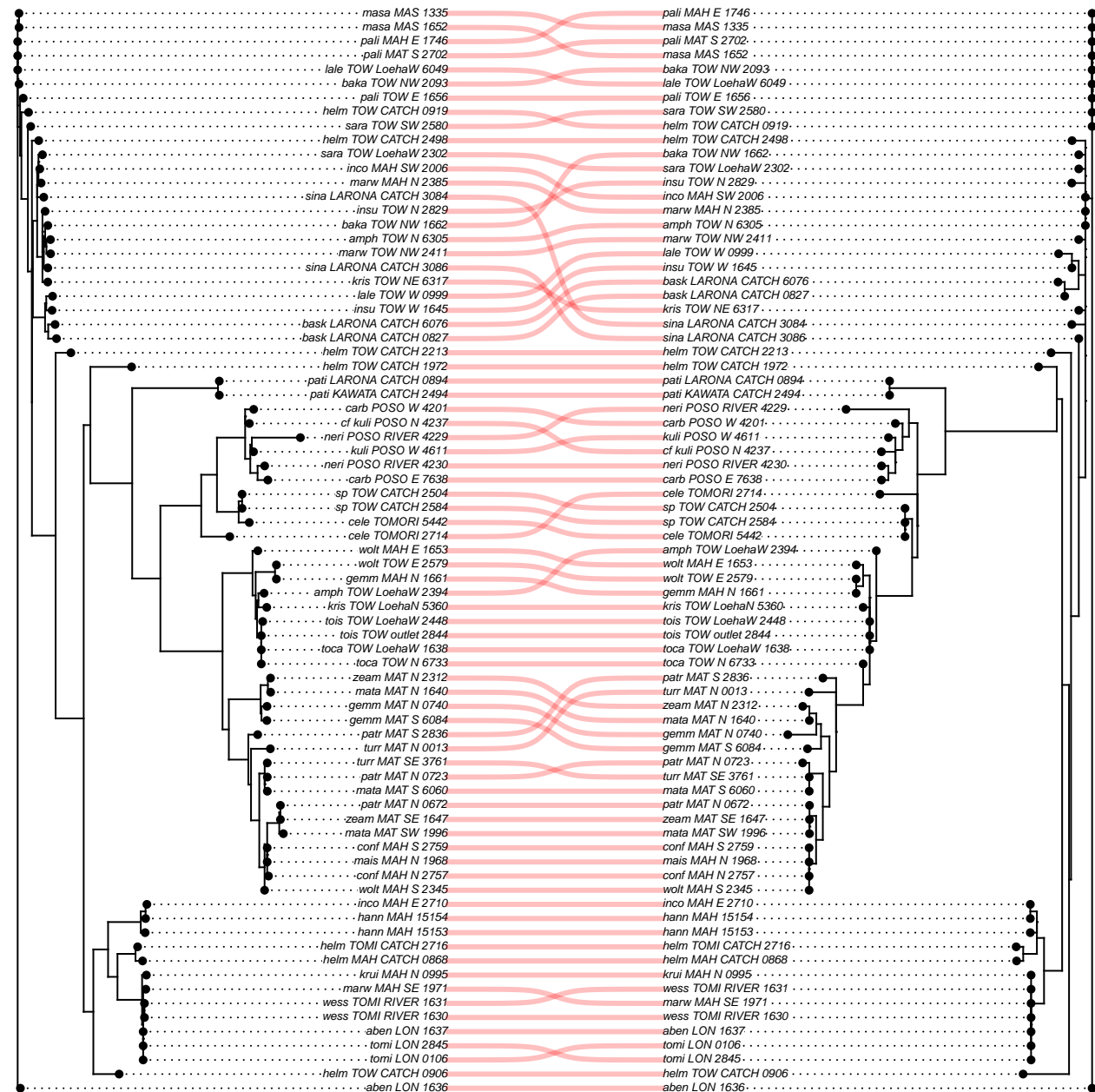

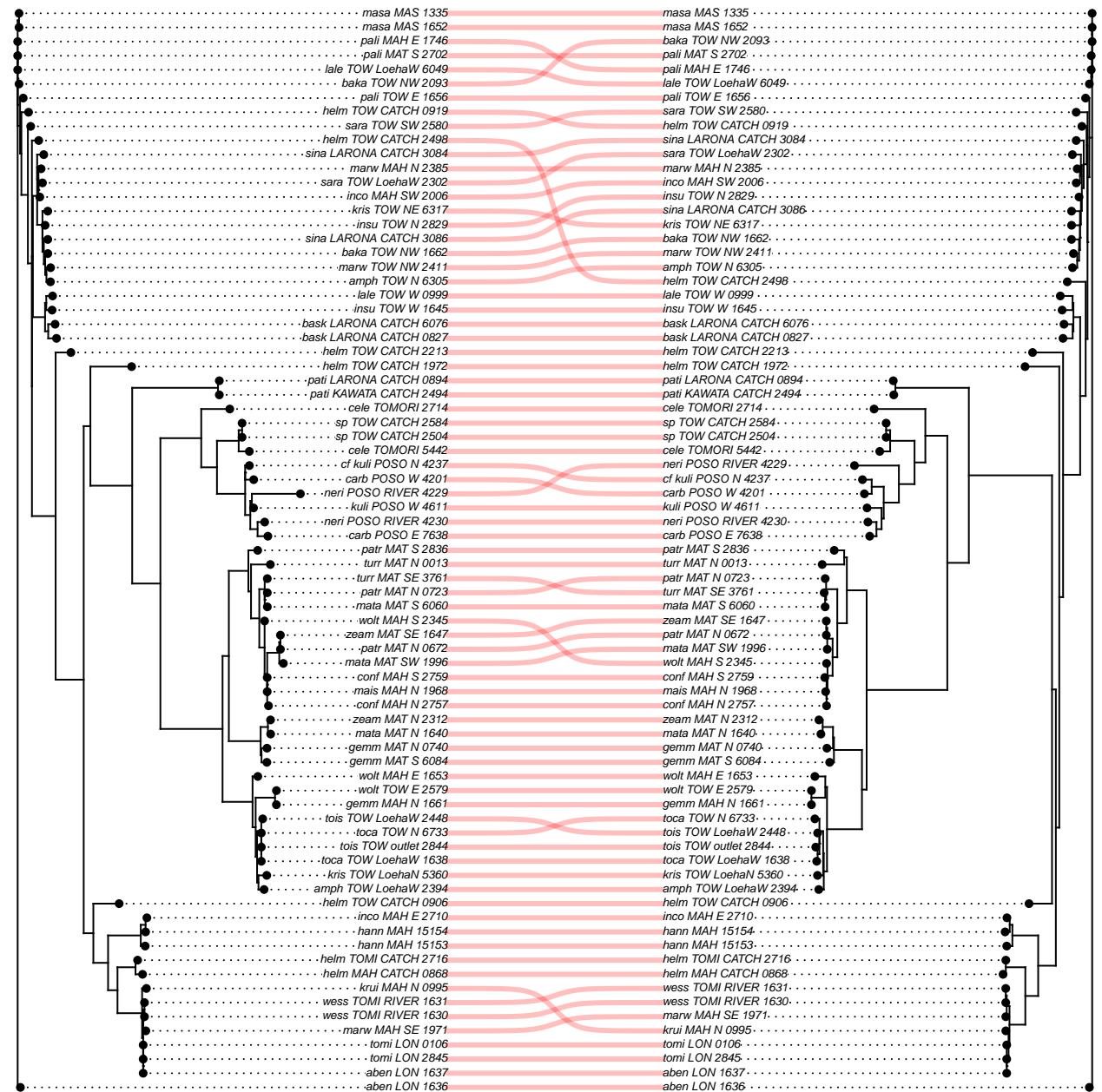

COX1

ND6

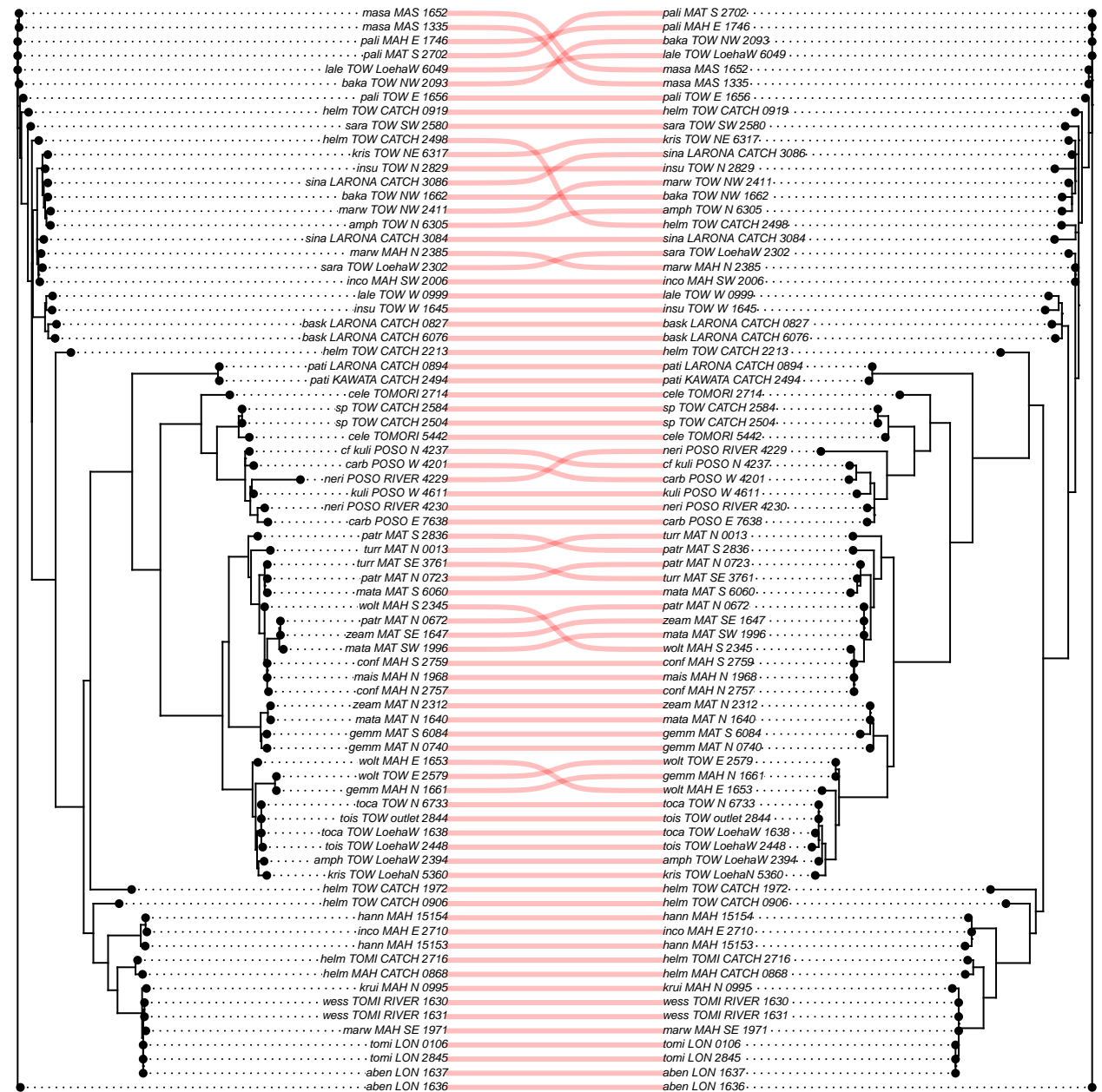

Supplement: Supplementary file 4 — Additional file 4: Figure S4. Cophyloplots for the different mitochondrial loci analysed vs. the COX1 topology (left side of the plot). [file 12862_2024_2235_MOESM4_ESM.pdf]
