# Supplementary material for: Mitogenomes do not substantially improve phylogenetic resolution in a young non-model adaptive radiation of freshwater gastropods
Source: BMC Ecol Evol. 2024 Apr 8;24:42. doi: 10.1186/s12862-024-02235-0 (PMC11000327; doi:10.1186/s12862-024-02235-0)

0013_aln

Number of used reads: 169,336 (100.0% of all input reads)

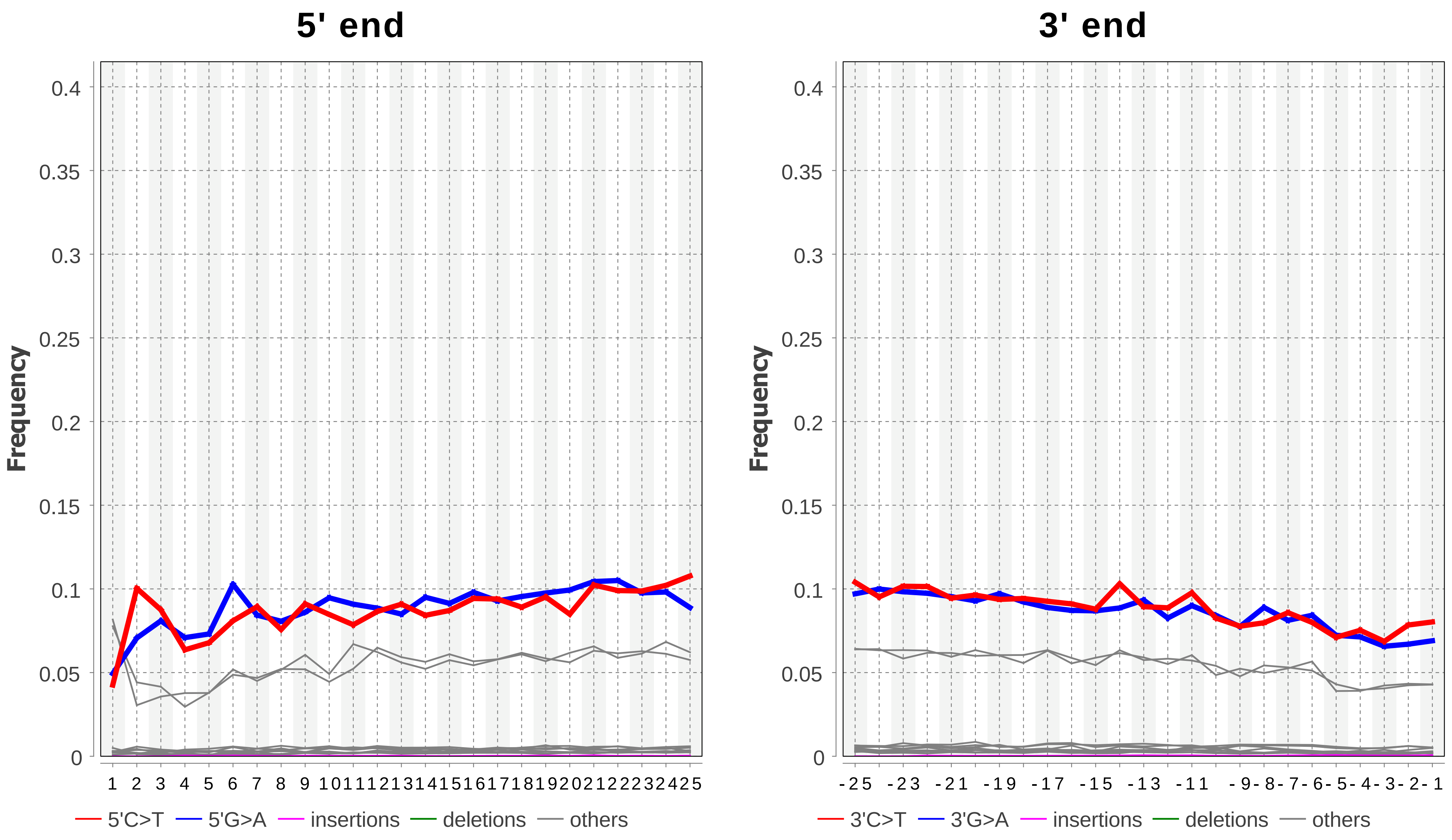

0013_MarkDuplicates

Number of used reads: 75,858 (100.0% of all input reads)

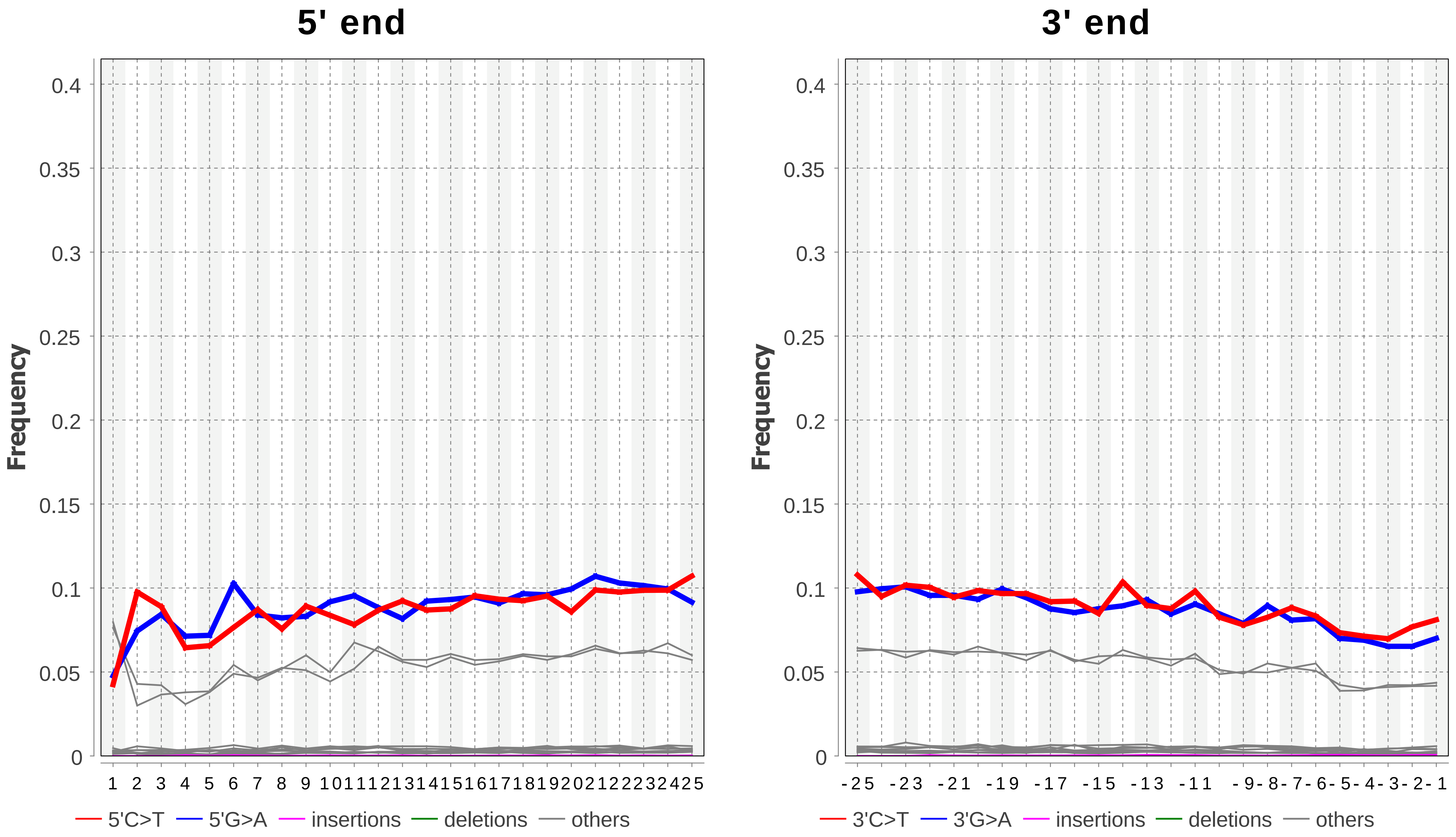

0106_aln
Number of used reads: 88,891 (100.0% of all input reads)

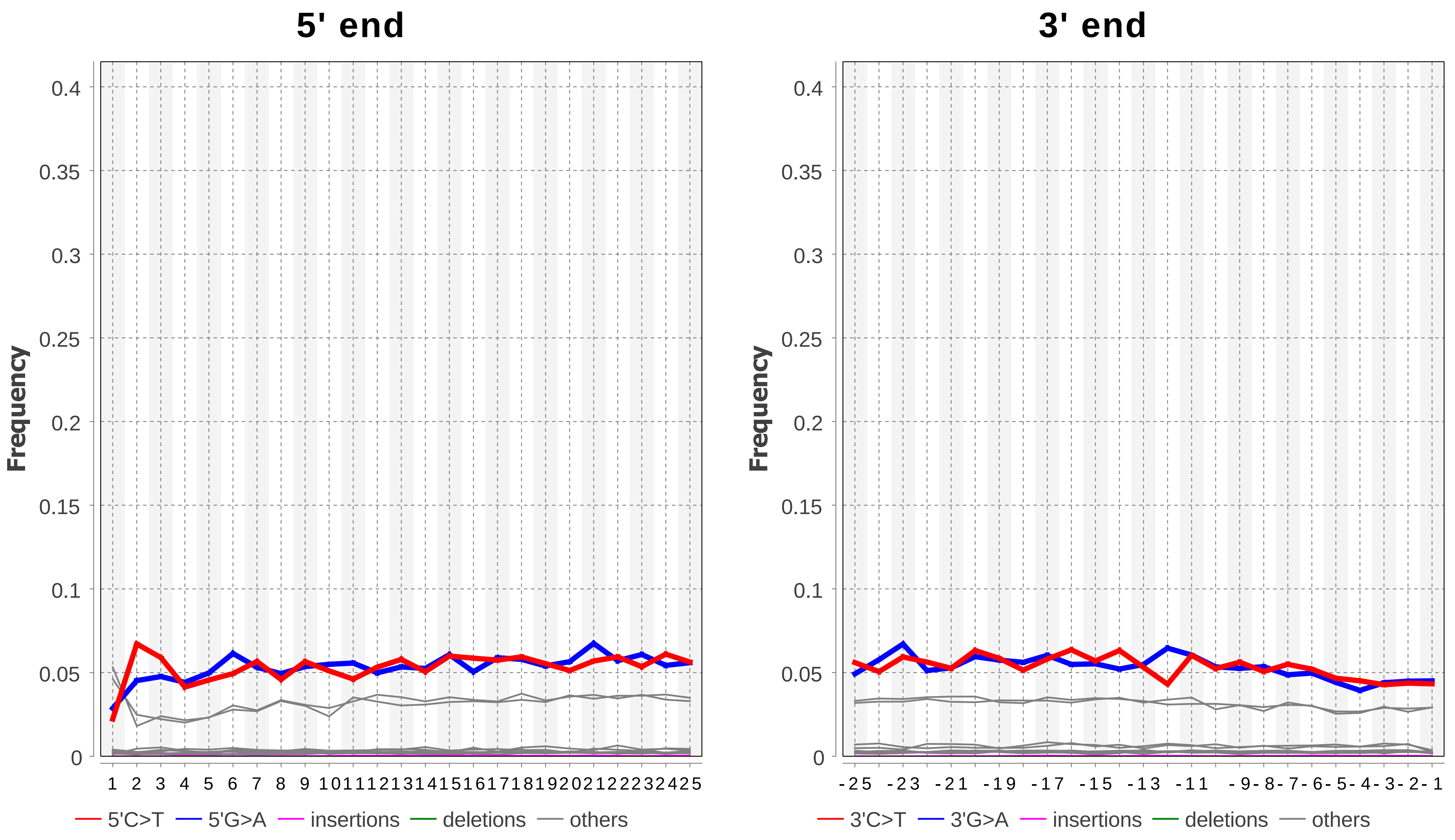

0106_MarkDuplicates
Number of used reads: 67,324 (100.0% of all input reads)

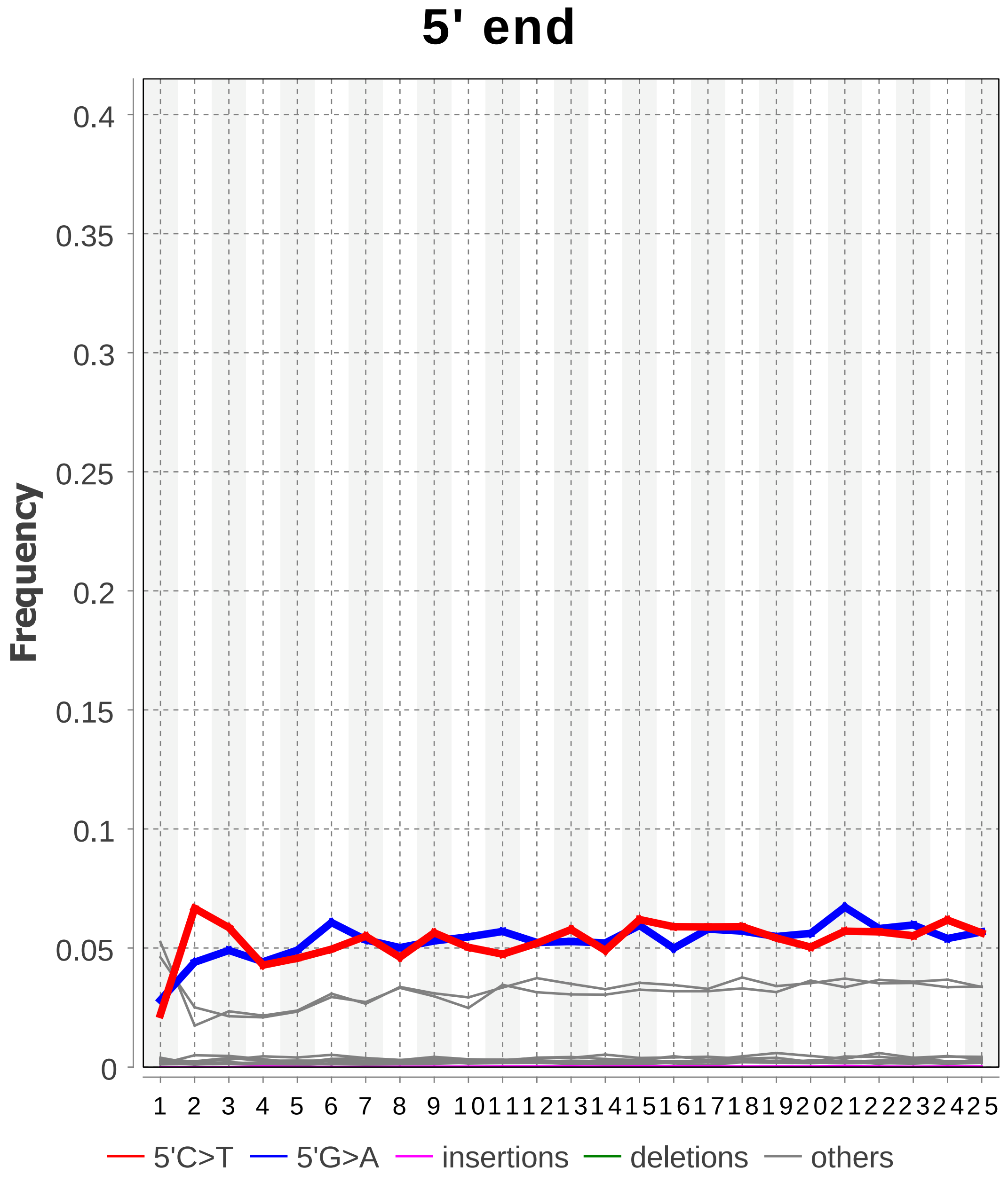

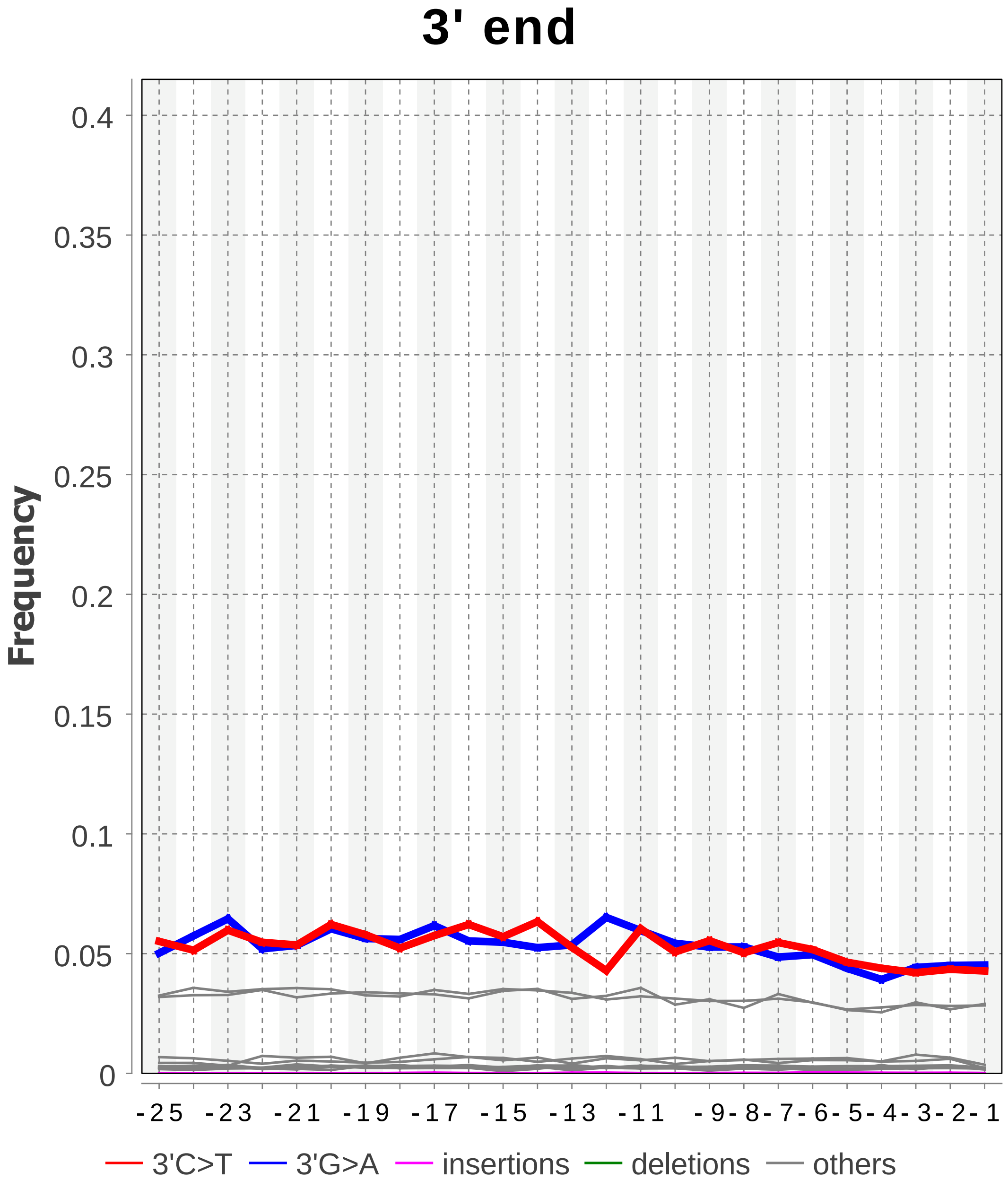

0672_aln

Number of used reads: 138,802 (100.0% of all input reads)

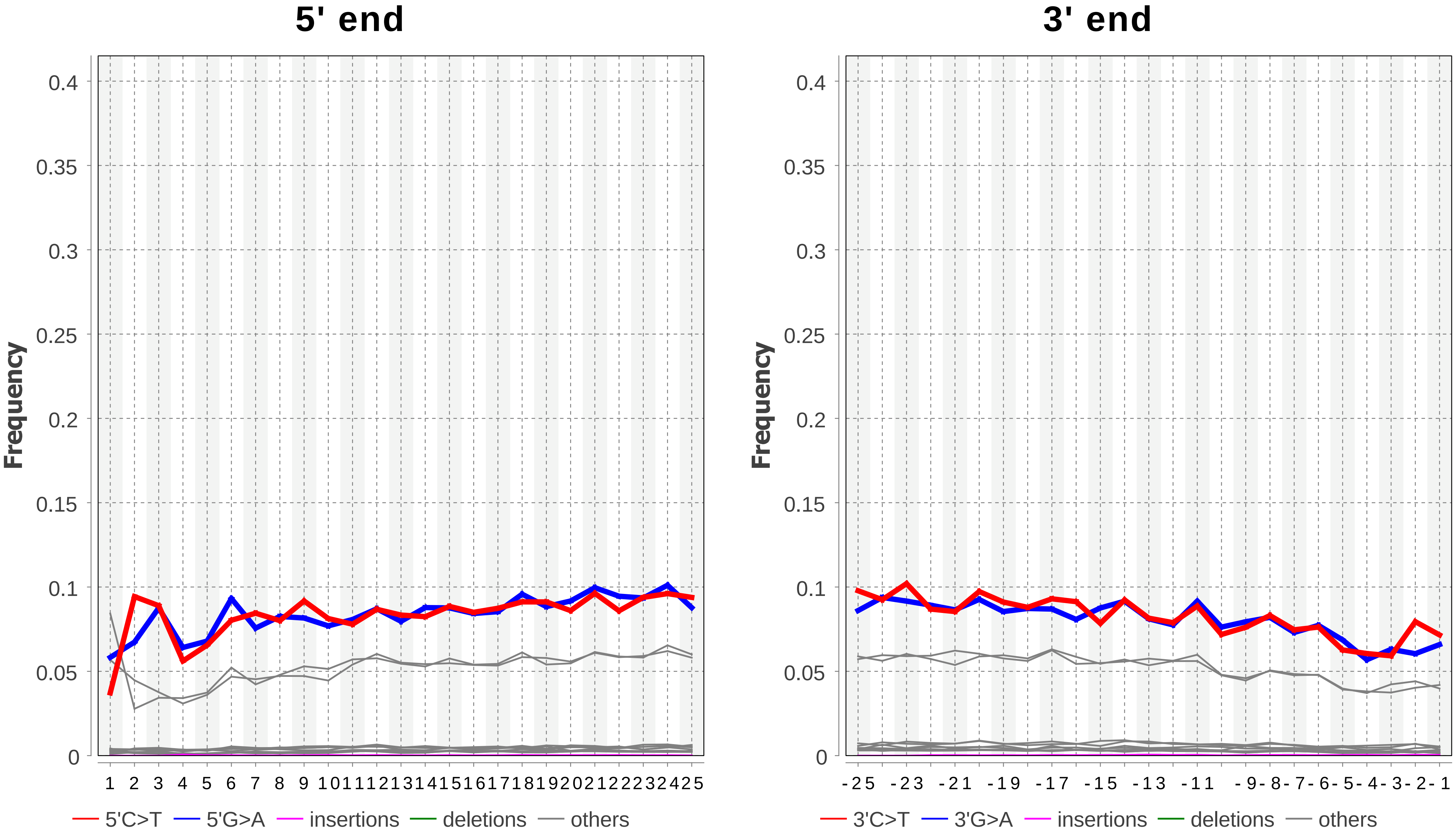

0672_MarkDuplicates
Number of used reads: 100,894 (100.0% of all input reads)

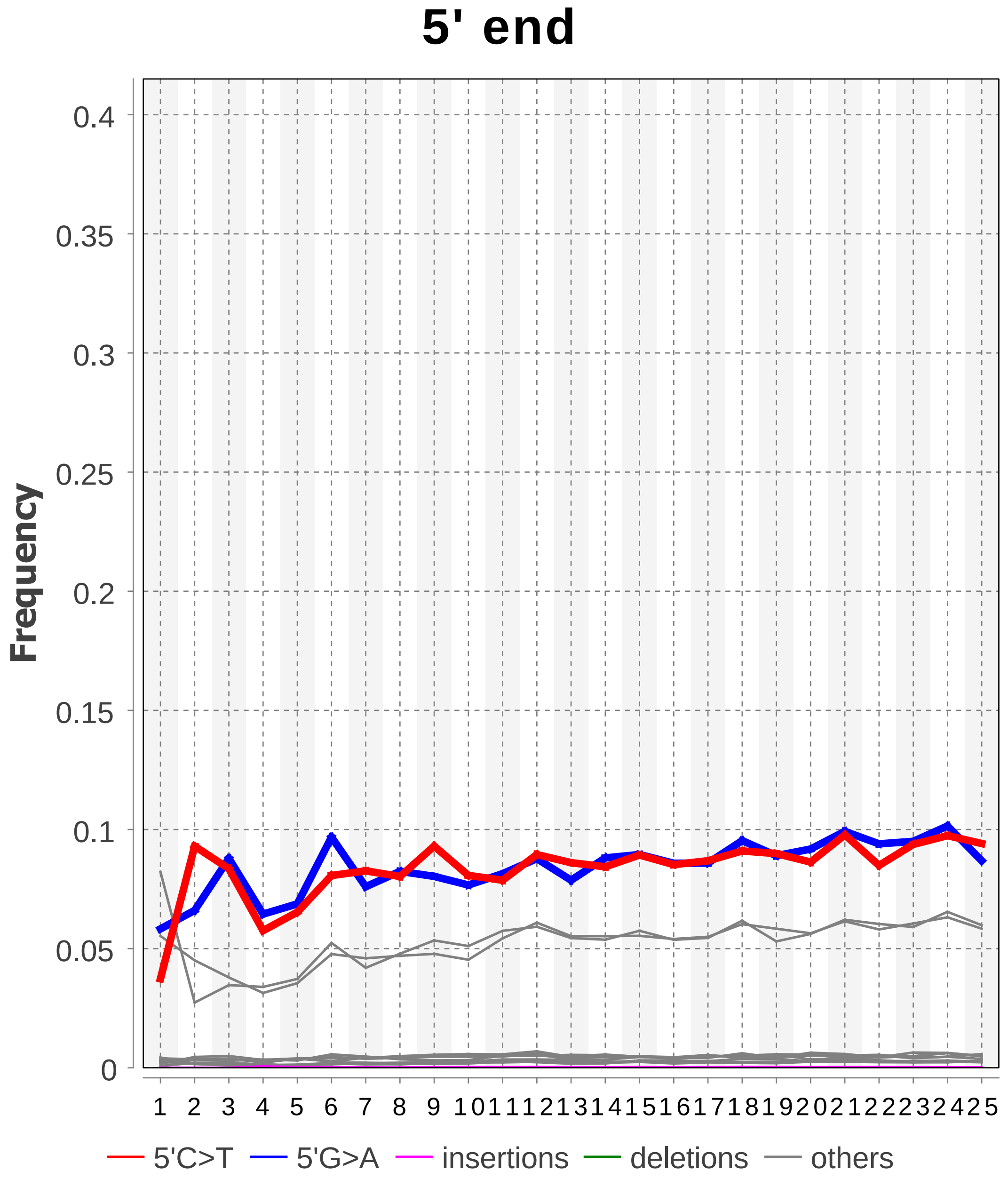

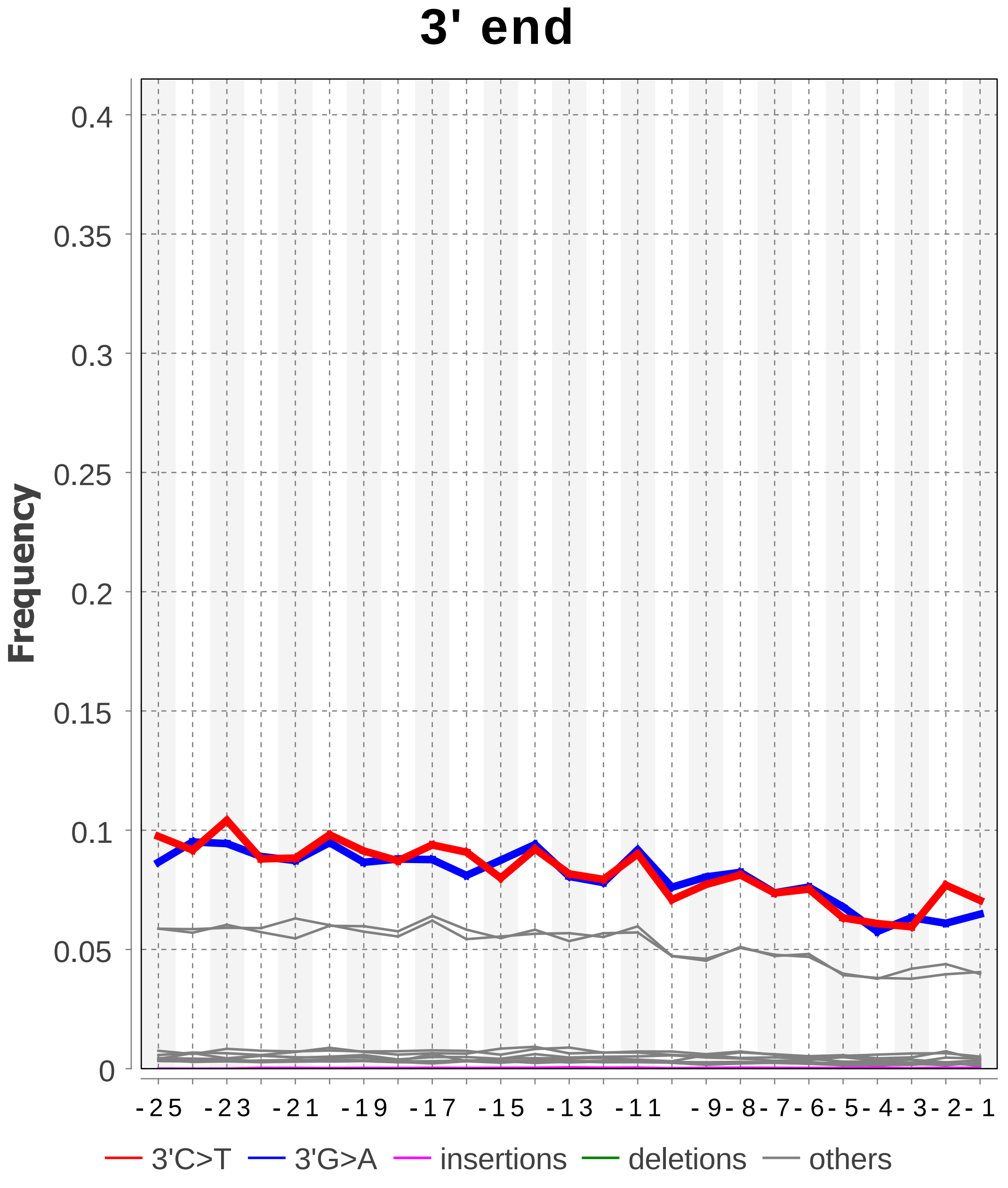

0723_aln
Number of used reads: 77,655 (100.0% of all input reads)

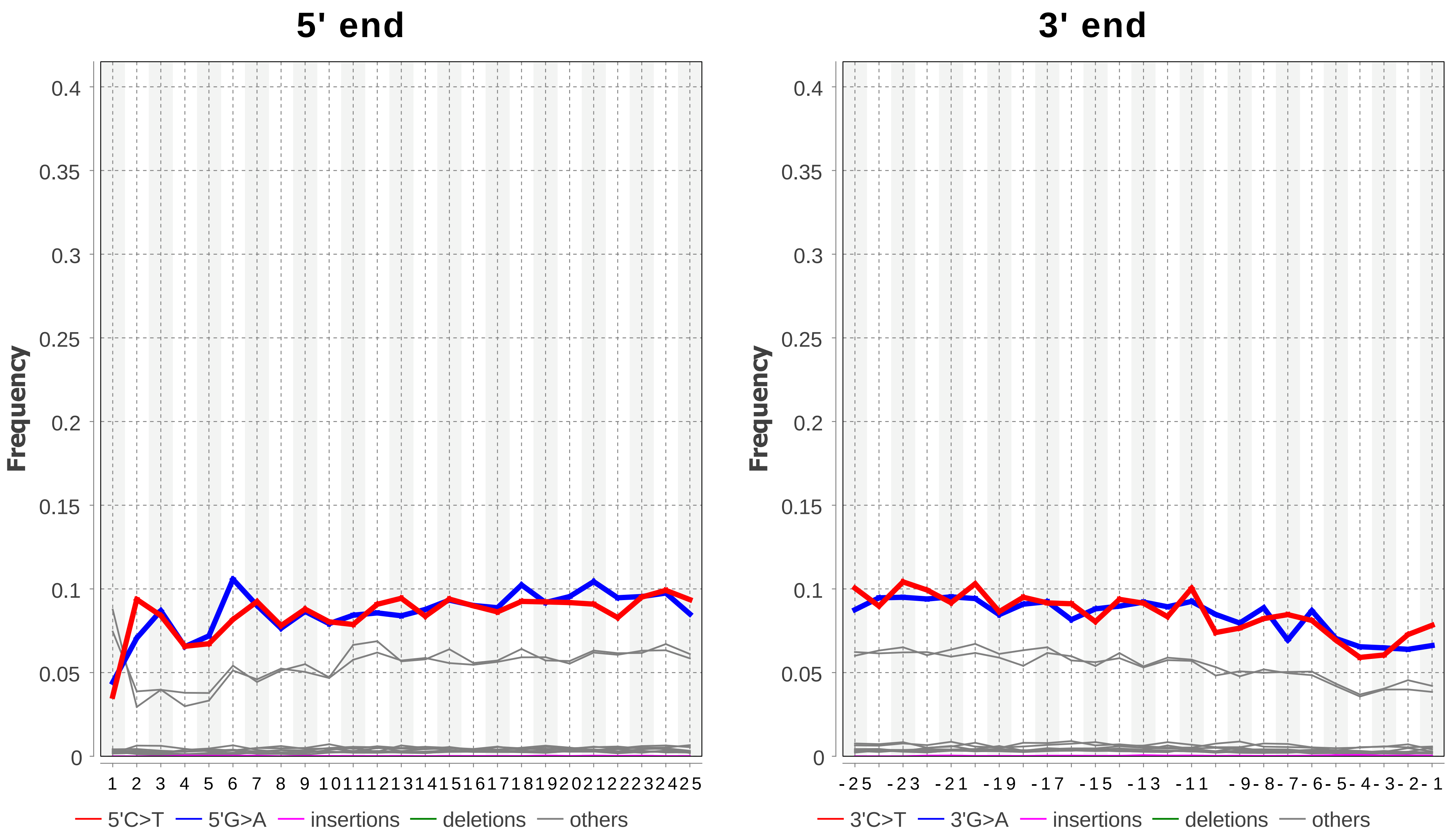

0723_MarkDuplicates
Number of used reads: 63,245 (100.0% of all input reads)

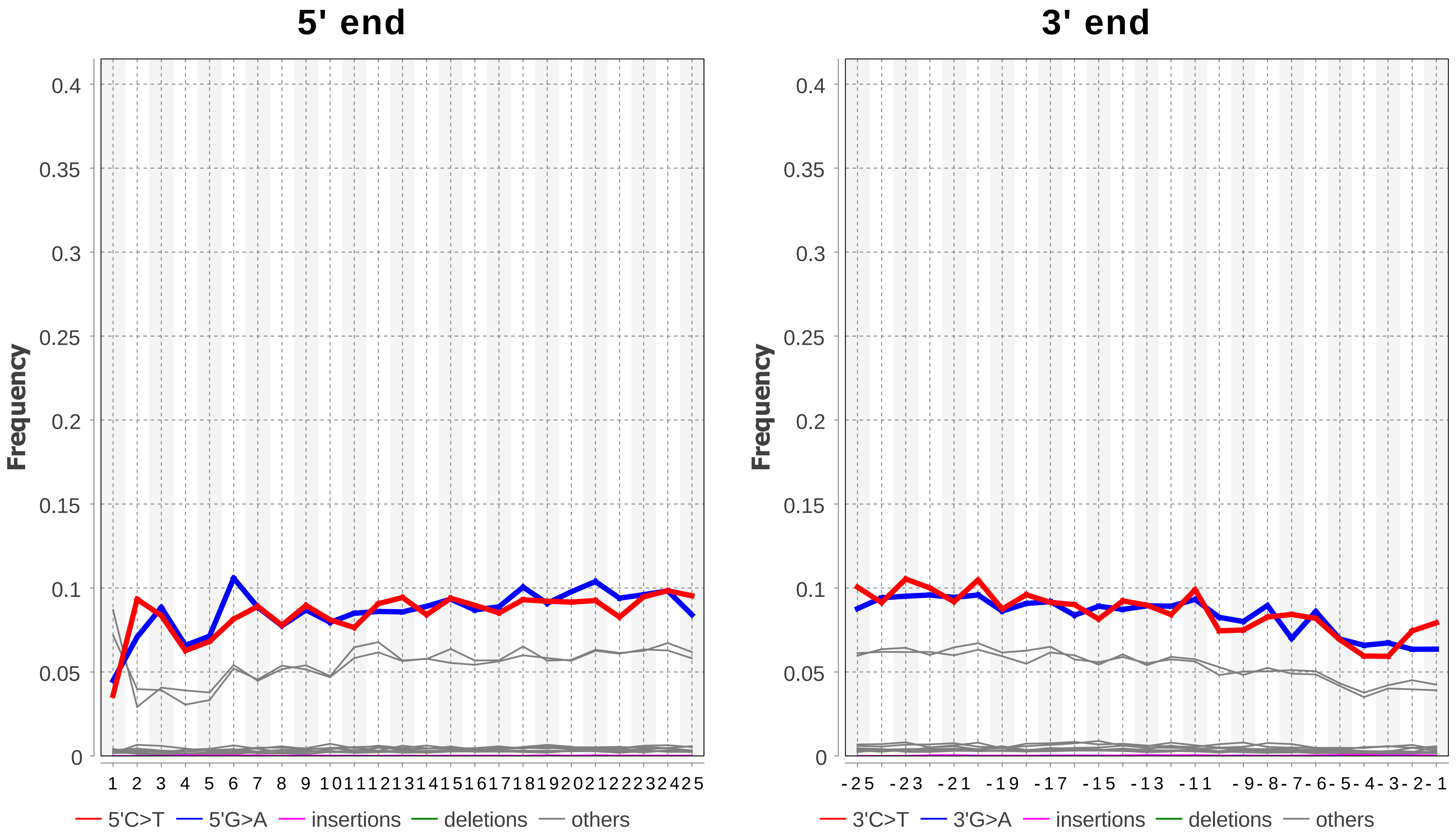

0740_aln

Number of used reads: 52,644 (100.0% of all input reads)

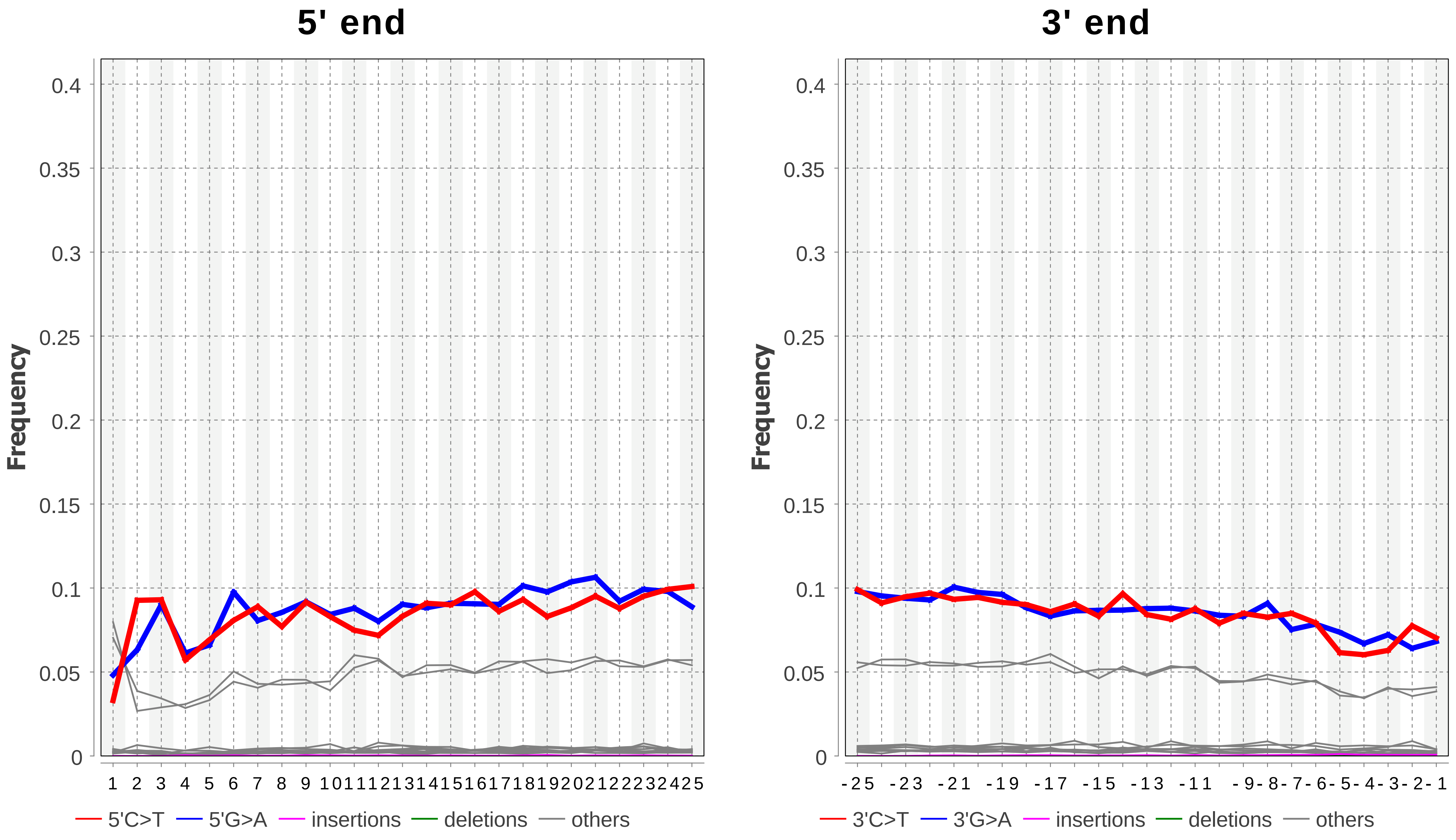

0740_MarkDuplicates
Number of used reads: 41,795 (100.0% of all input reads)

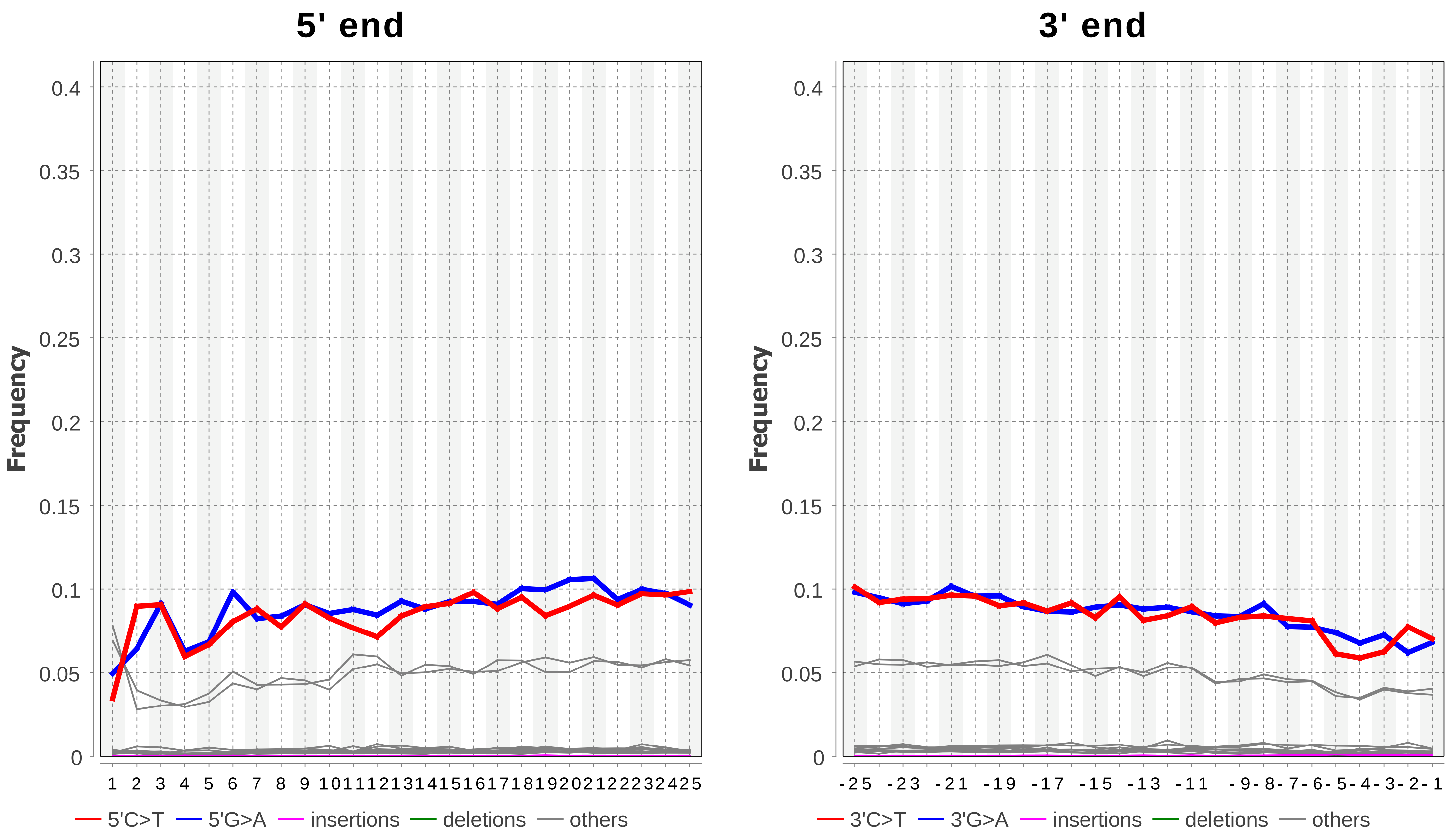

0827_aln

Number of used reads: 61,415 (100.0% of all input reads)

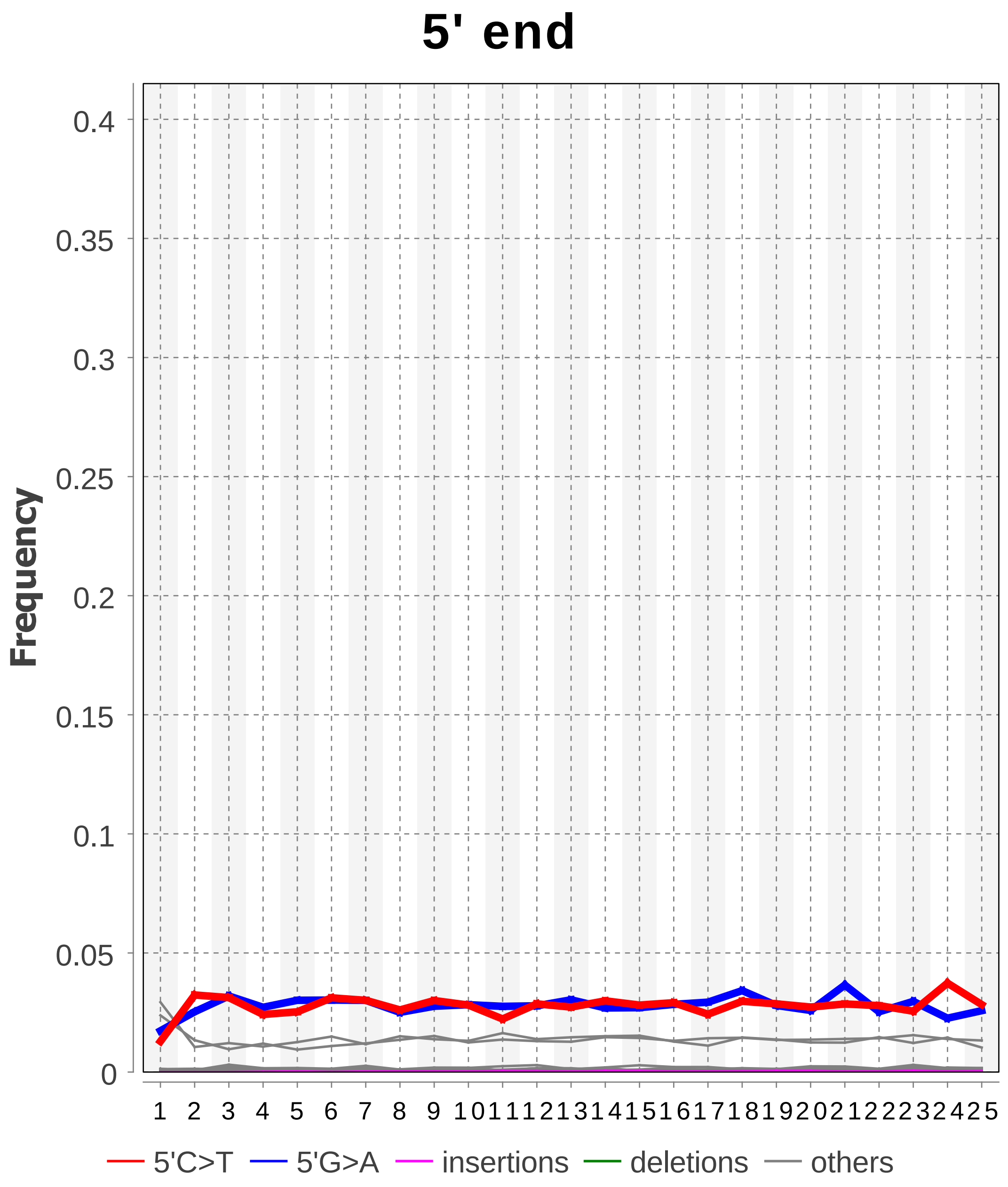

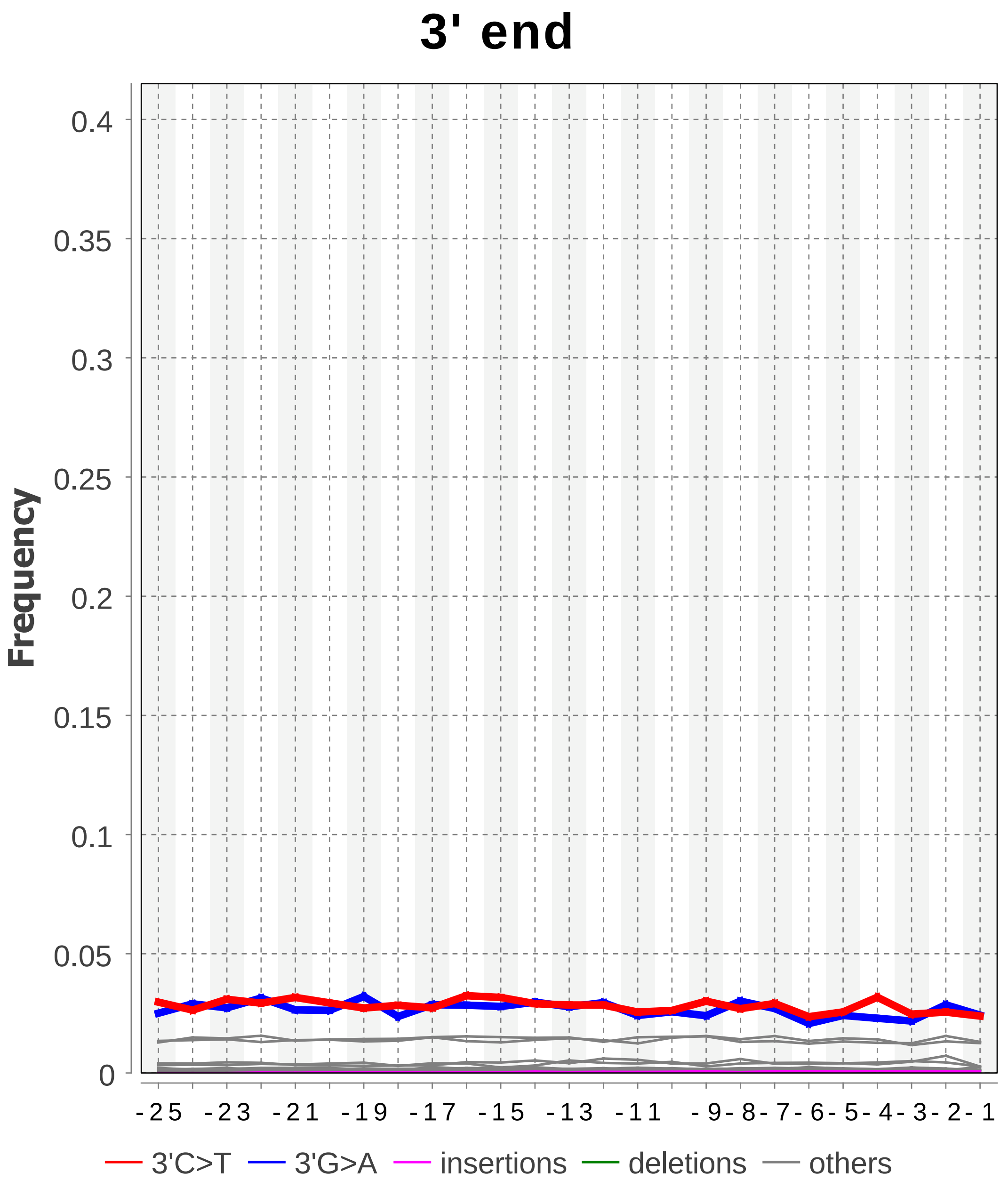

0827_MarkDuplicates

Number of used reads: 51,852 (100.0% of all input reads)

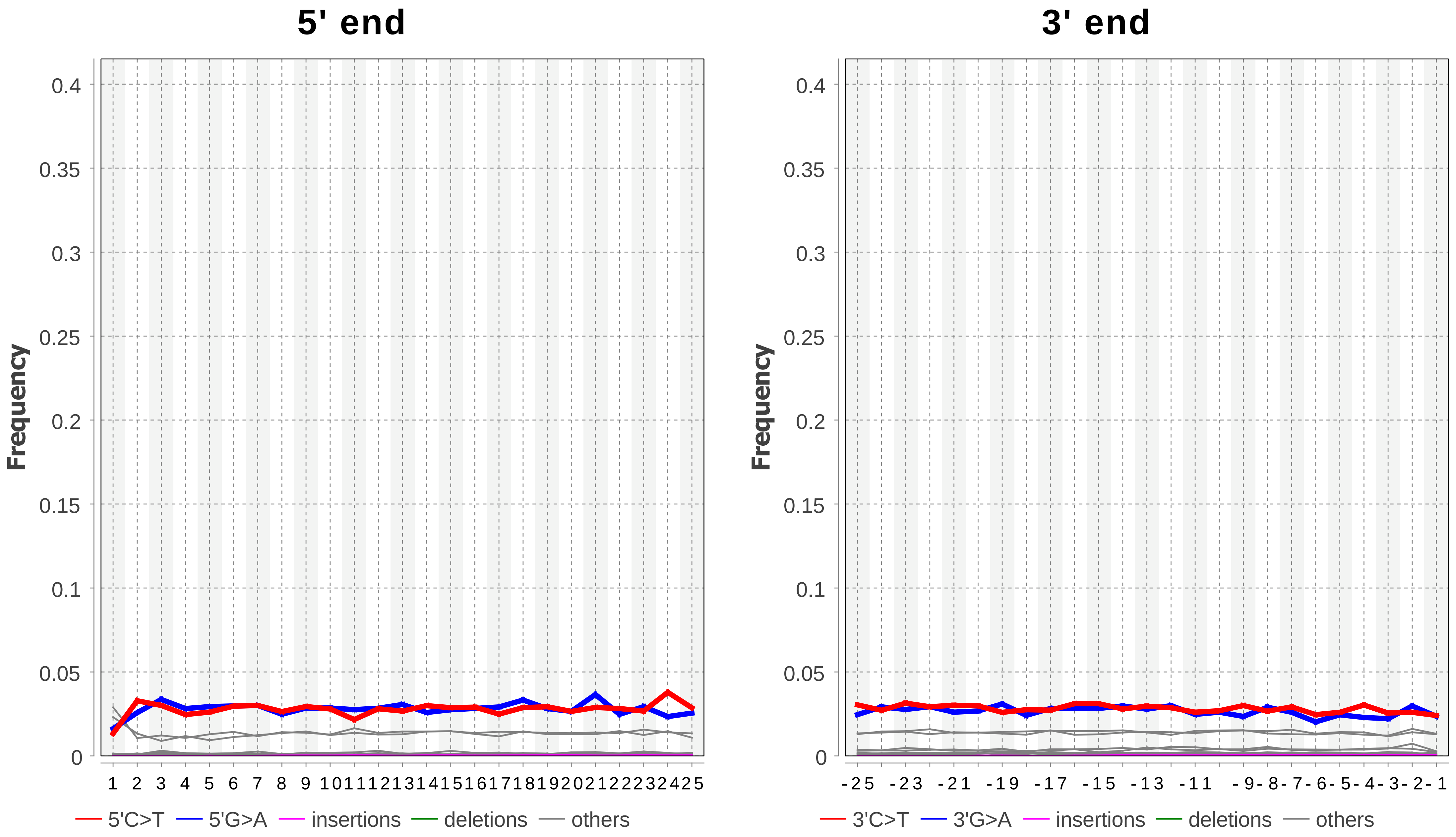

0868_aln

Number of used reads: 77,898 (100.0% of all input reads)

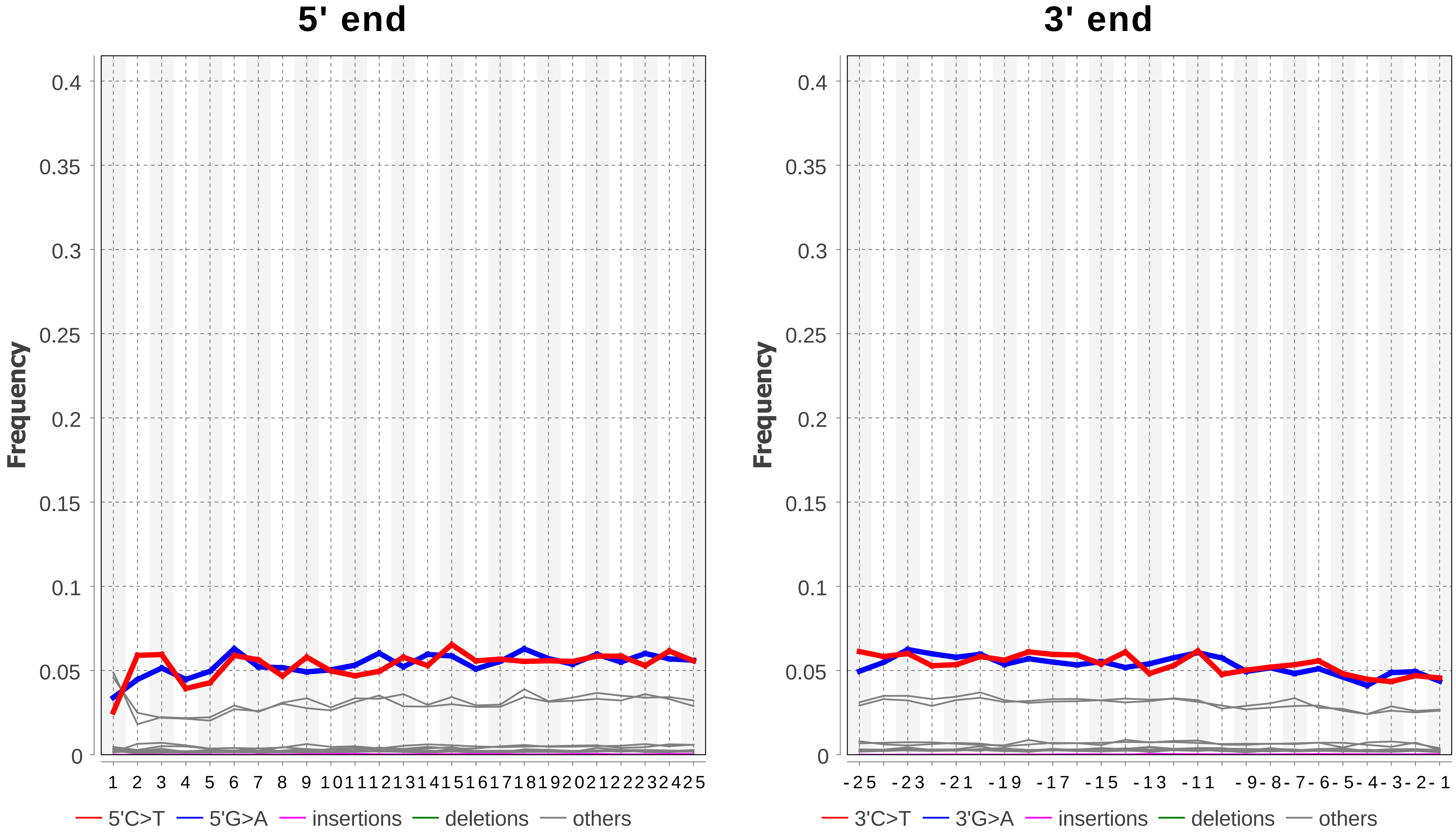

0868_MarkDuplicates
Number of used reads: 64,923 (100.0% of all input reads)

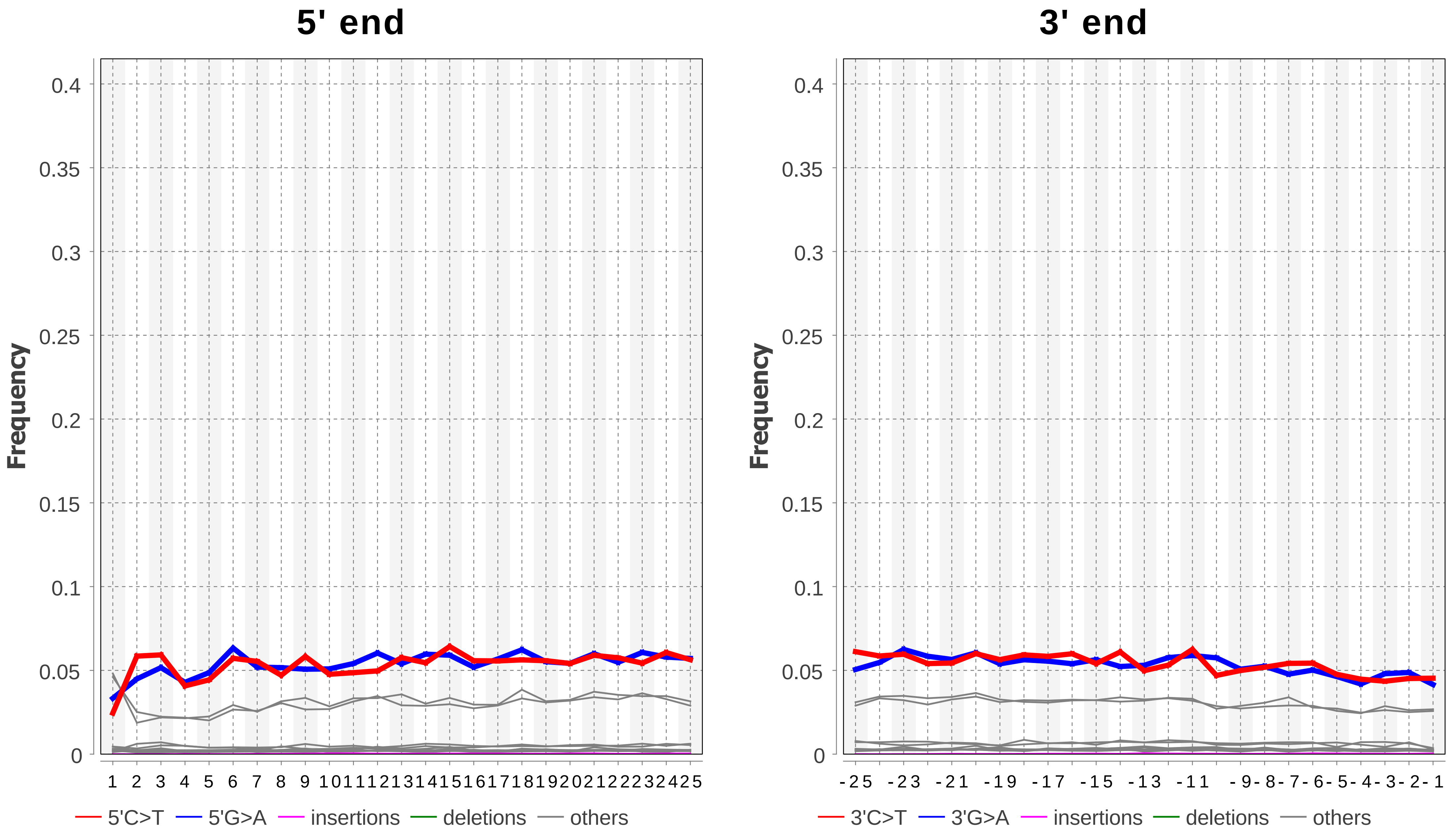

0894_aln
Number of used reads: 92,626 (100.0% of all input reads)

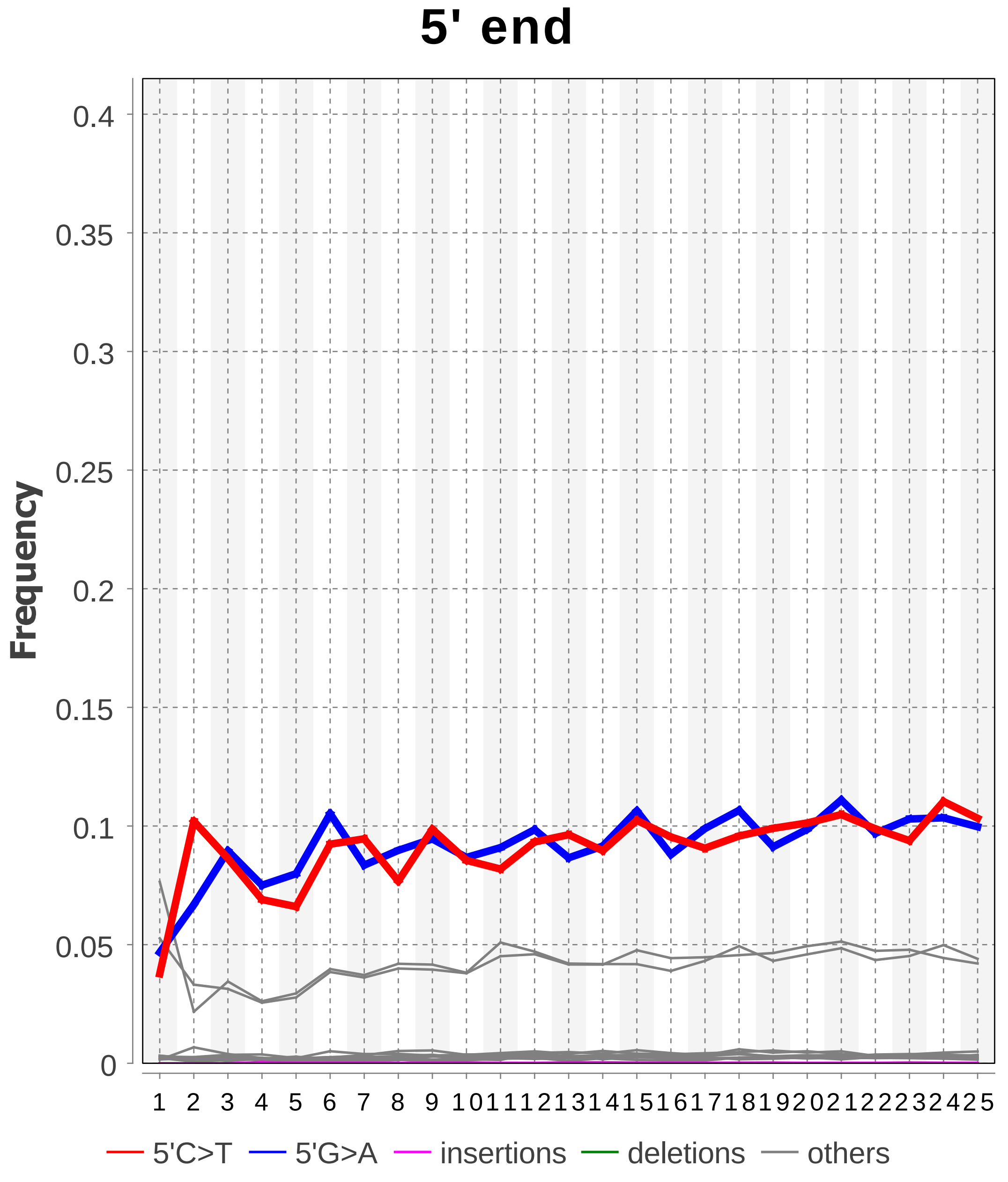

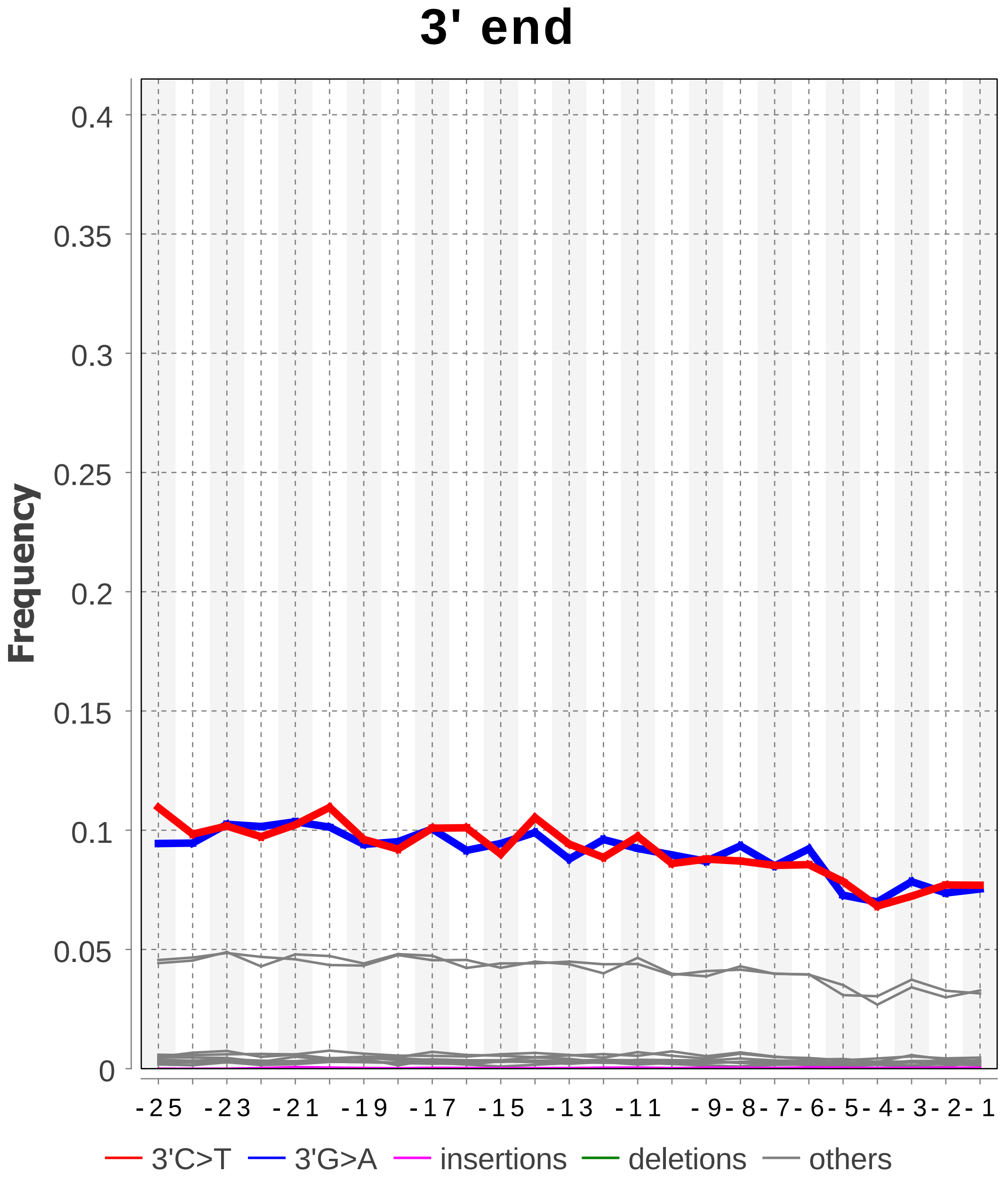

0894_MarkDuplicates

Number of used reads: 70,006 (100.0% of all input reads)

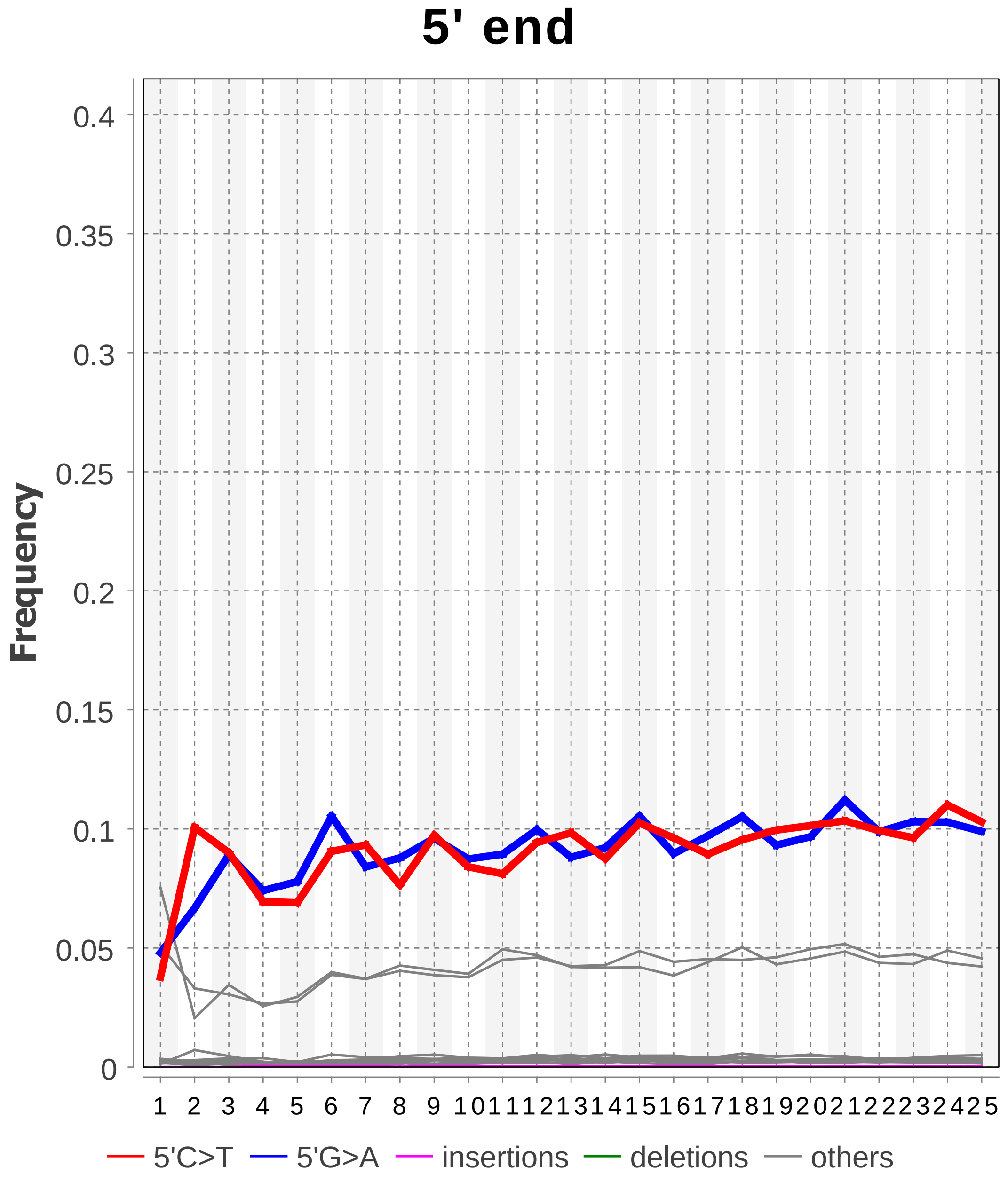

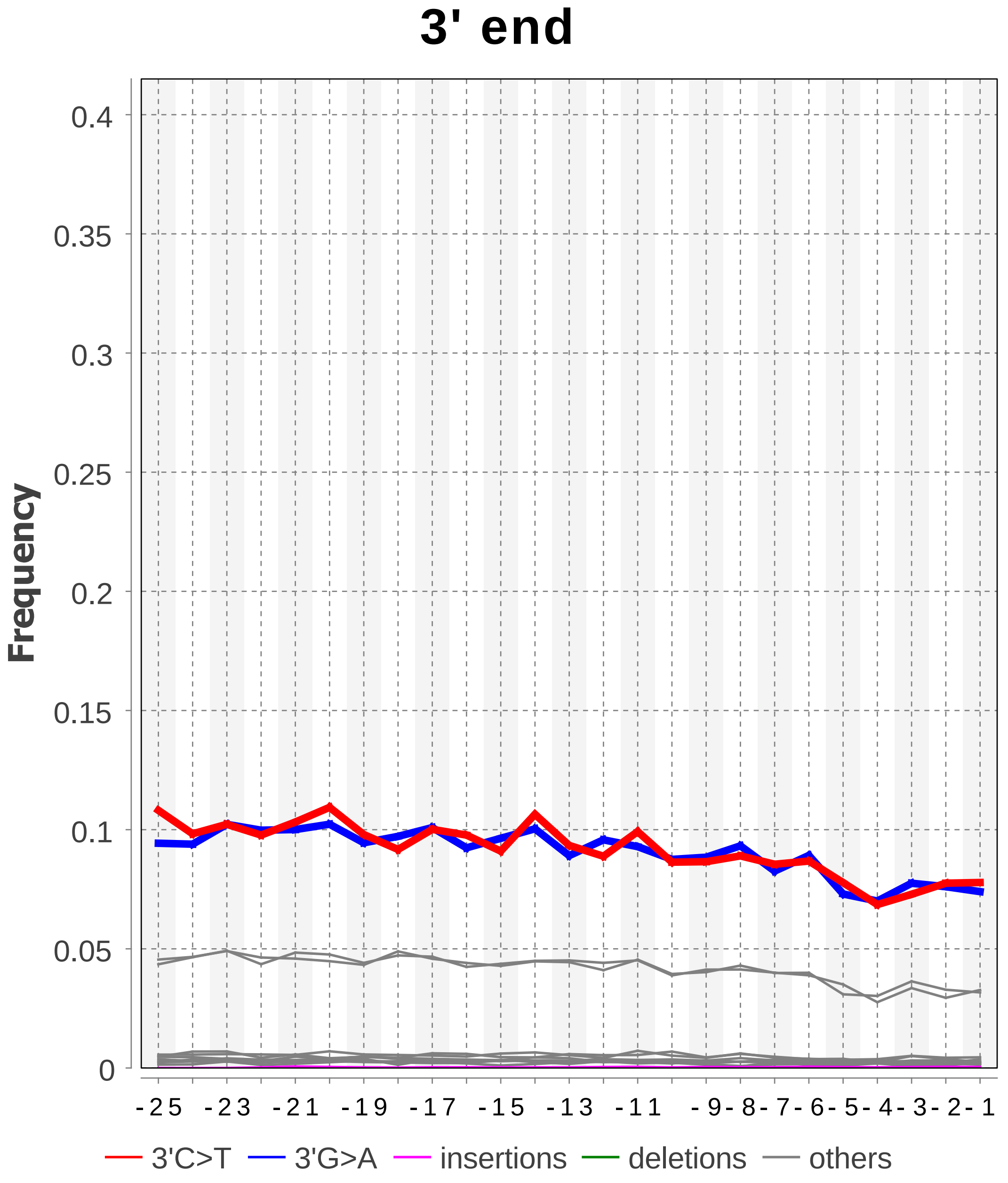

0906_aln

Number of used reads: 96,145 (100.0% of all input reads)

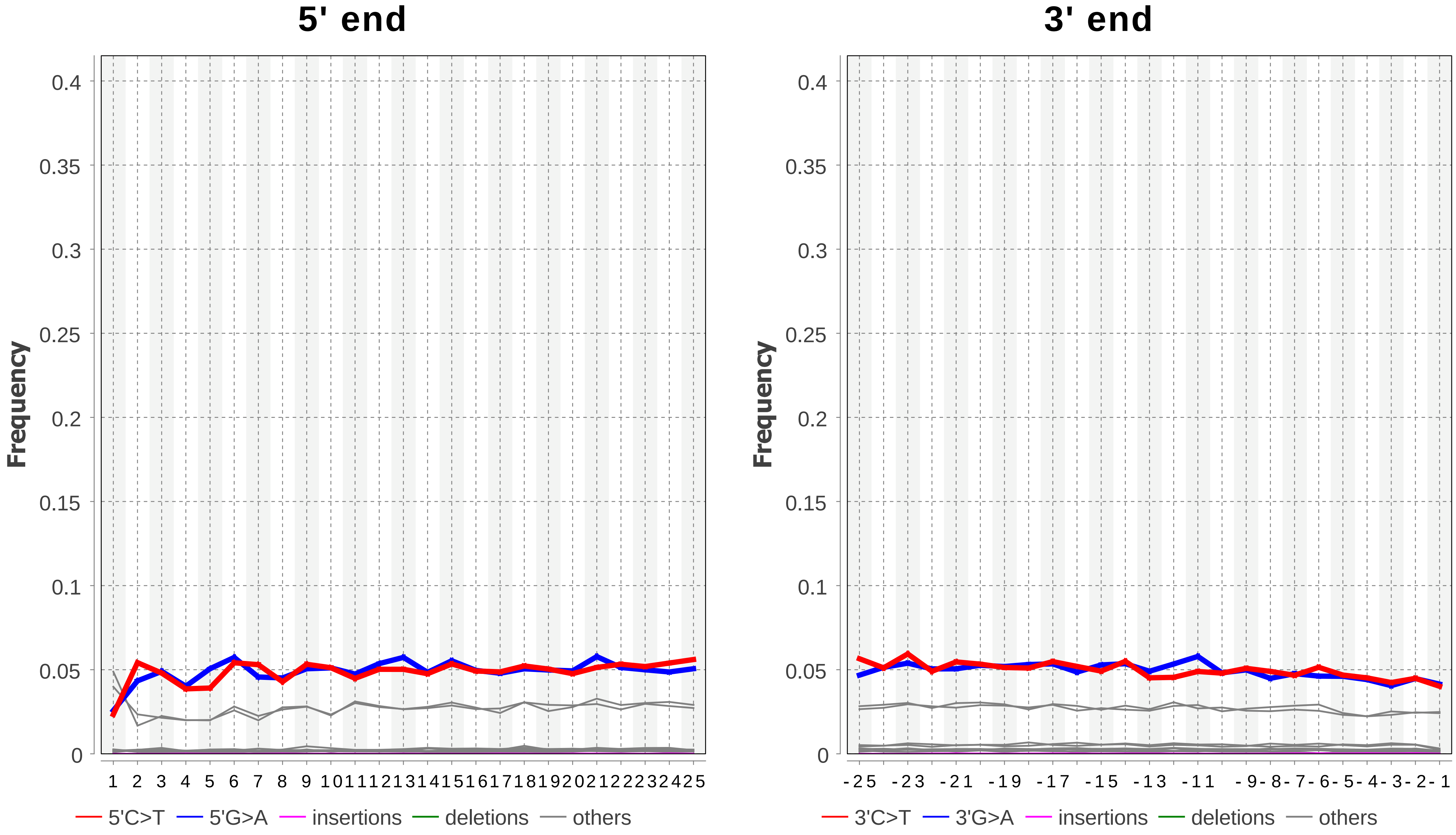

0906_MarkDuplicates
Number of used reads: 75,498 (100.0% of all input reads)

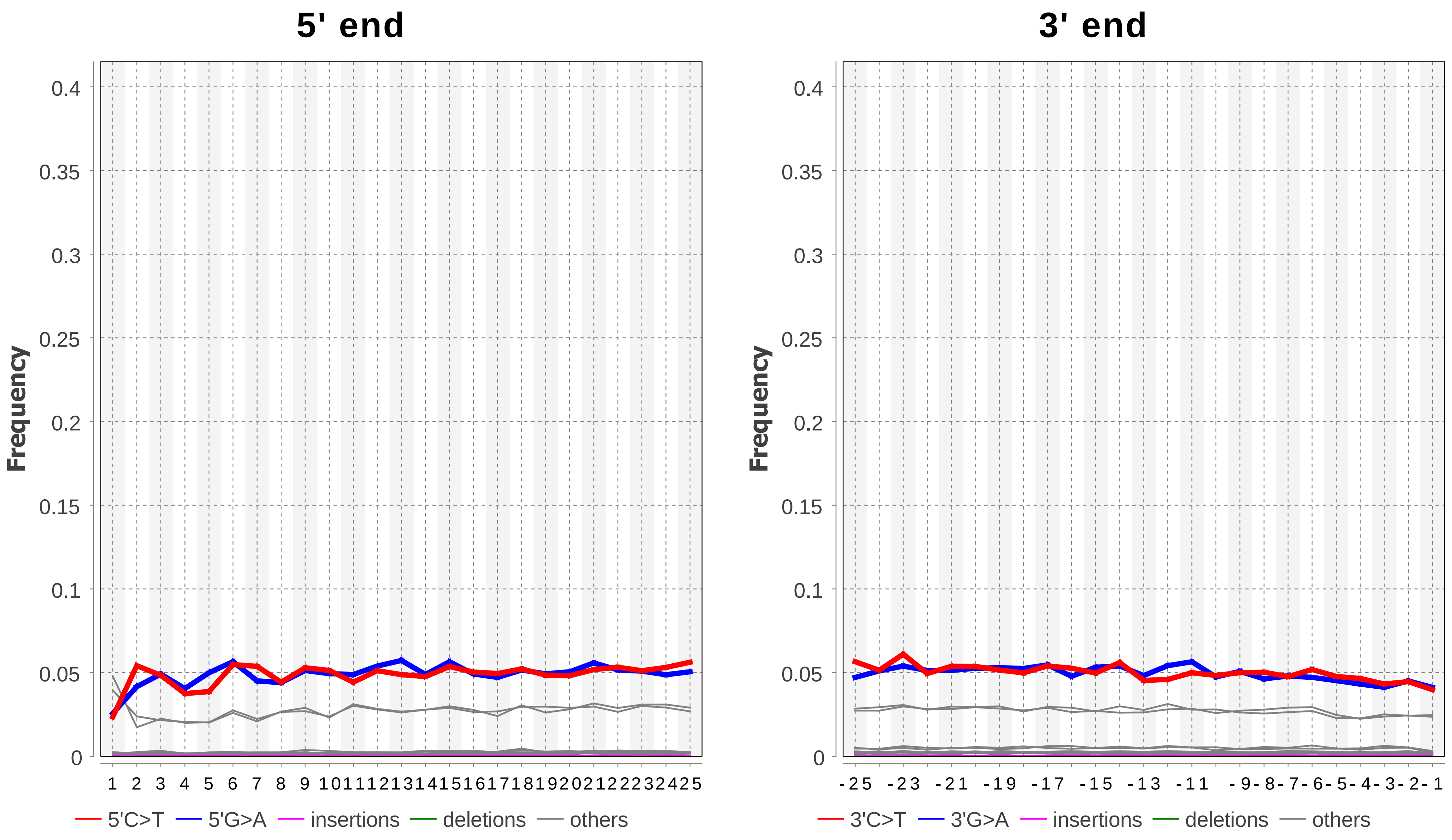

0919_aln

Number of used reads: 169,893 (100.0% of all input reads)

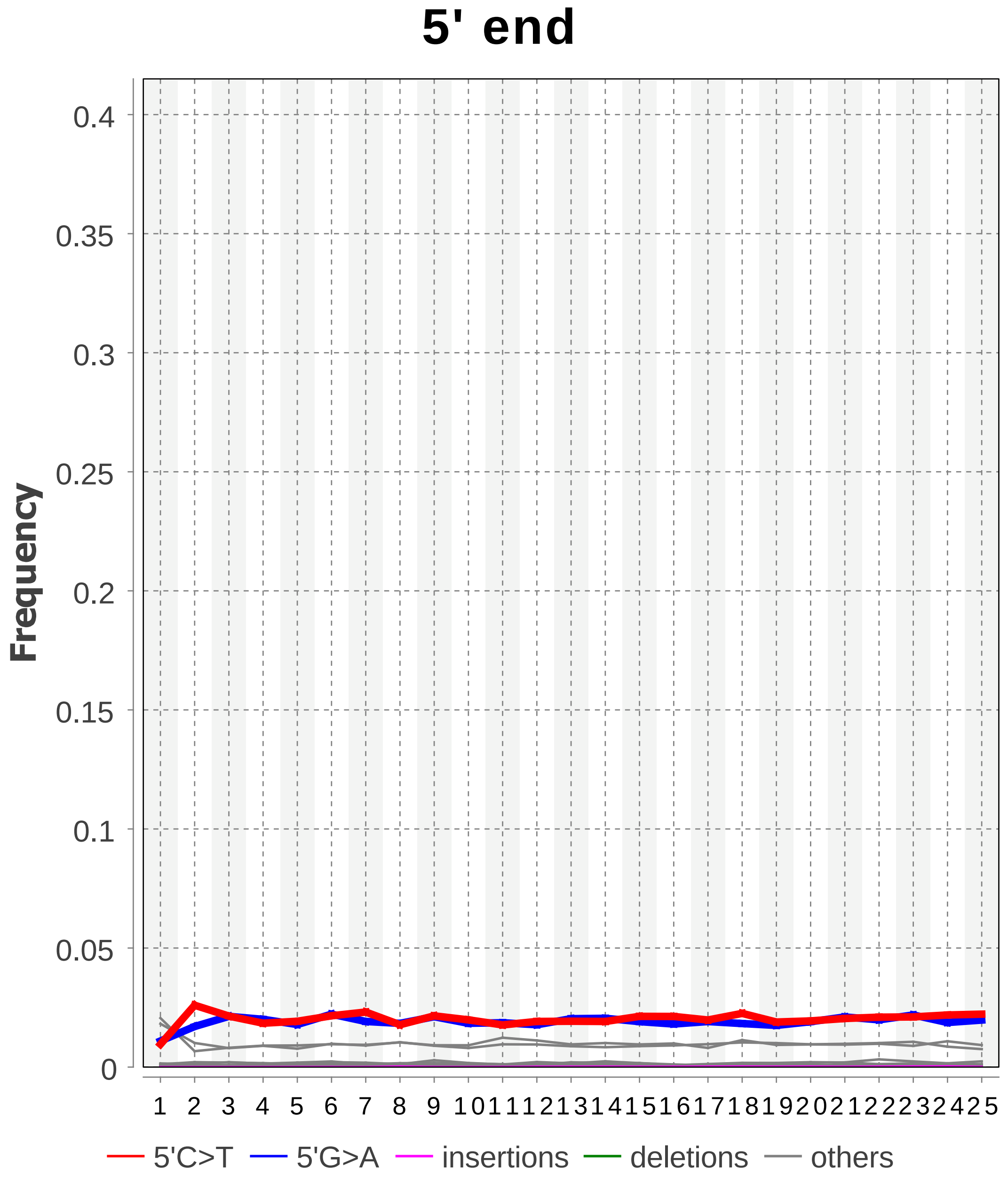

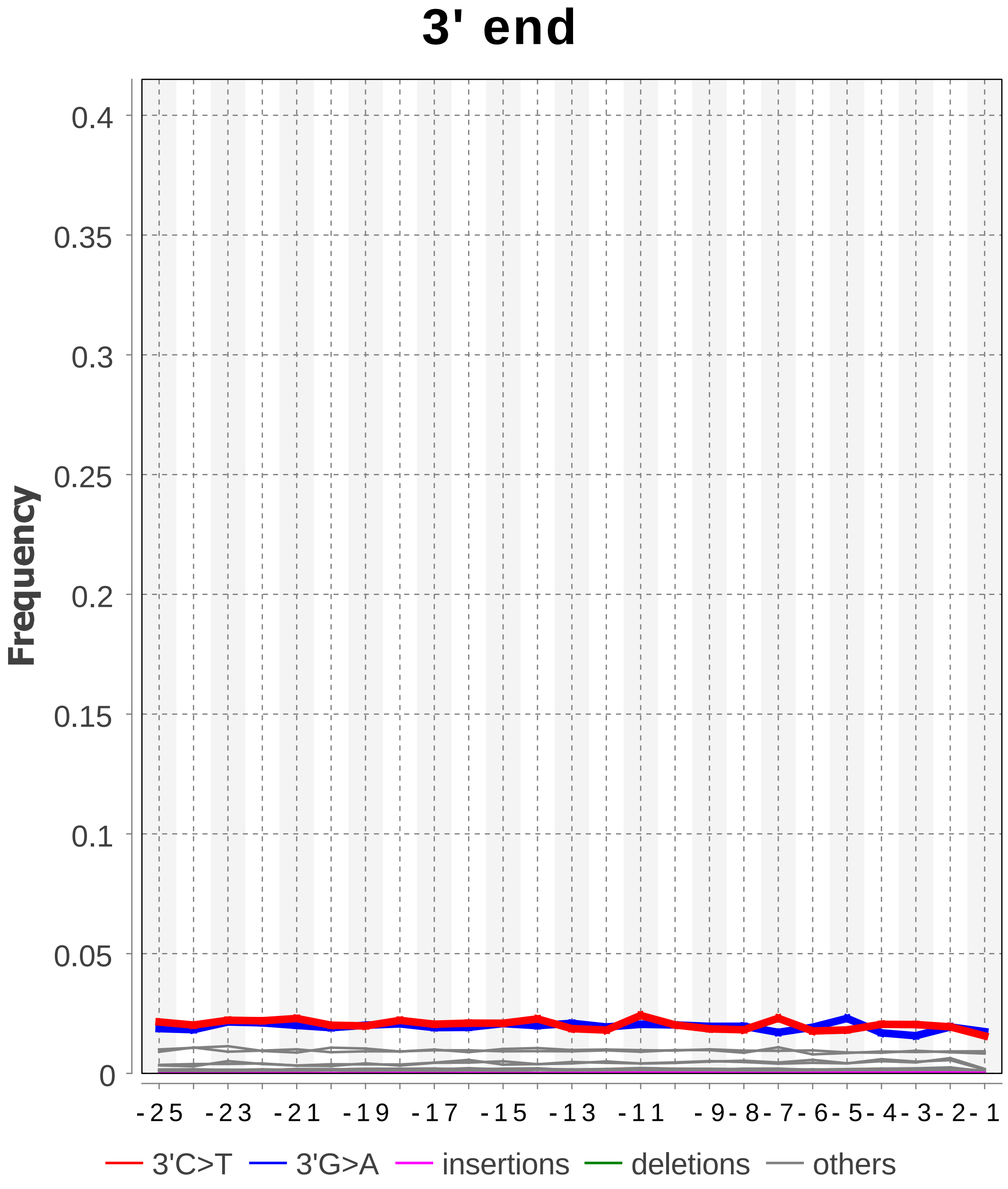

0919_MarkDuplicates

Number of used reads: 142,407 (100.0% of all input reads)

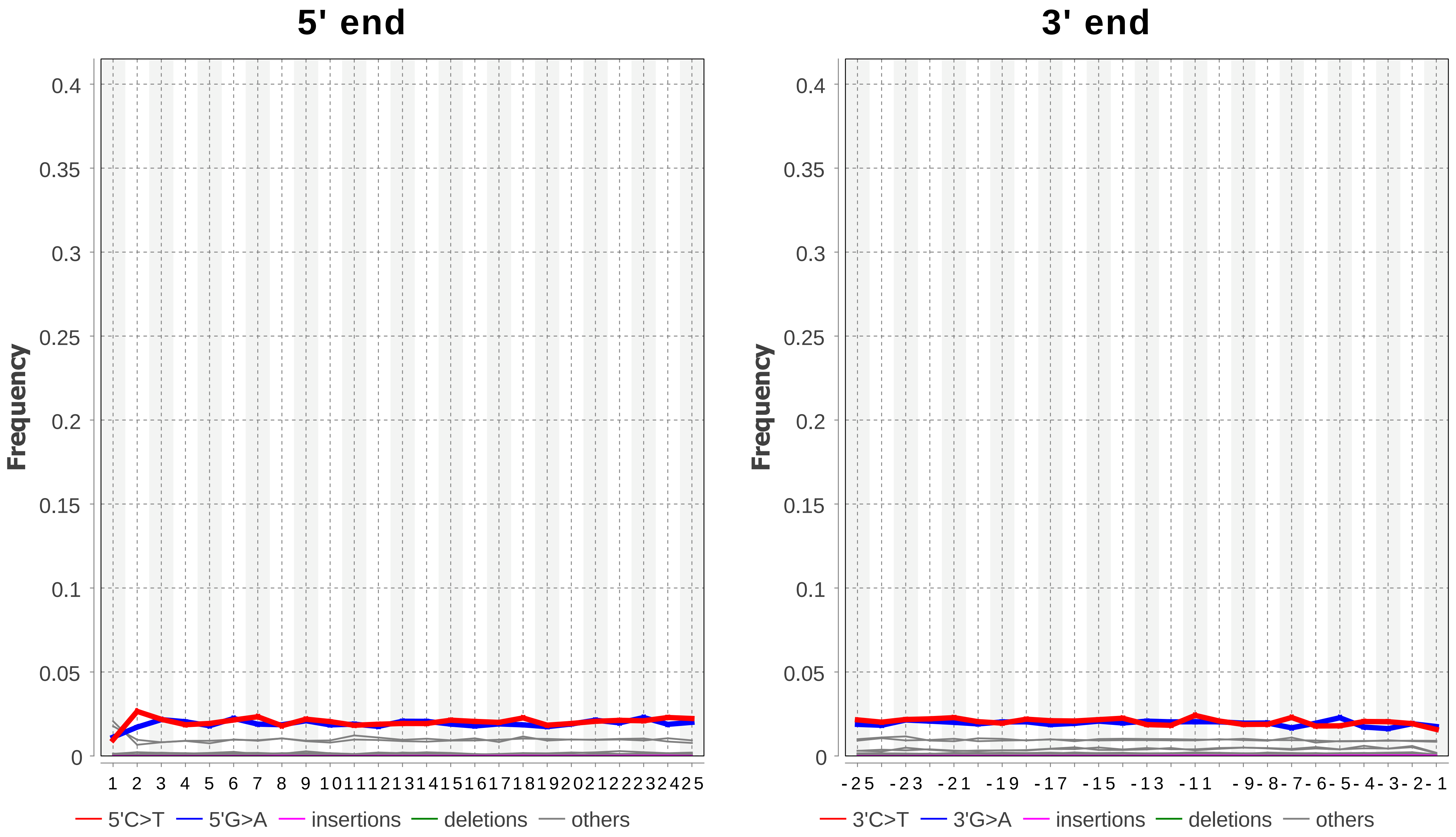

0995_aln
Number of used reads: 368,725 (100.0% of all input reads)

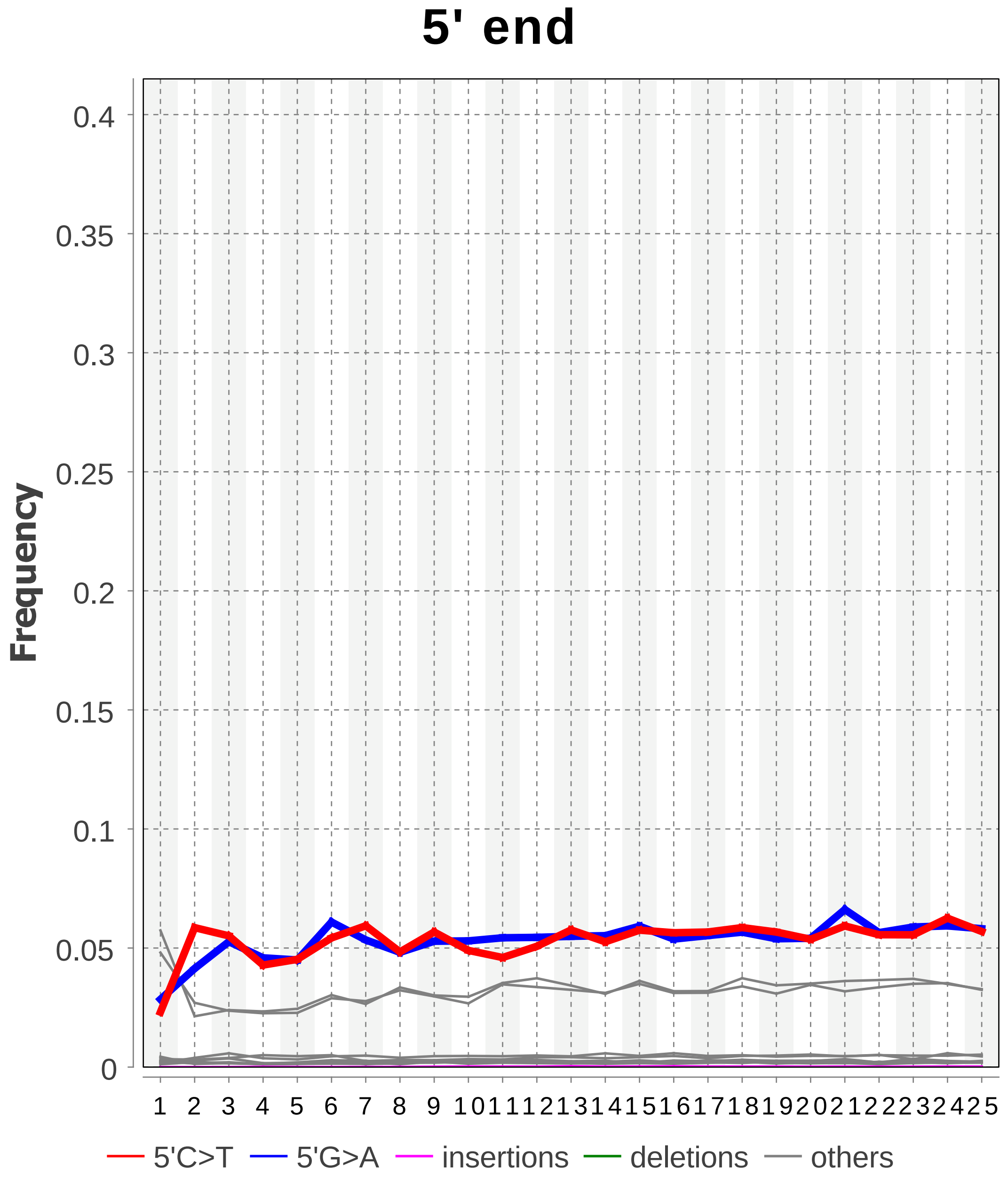

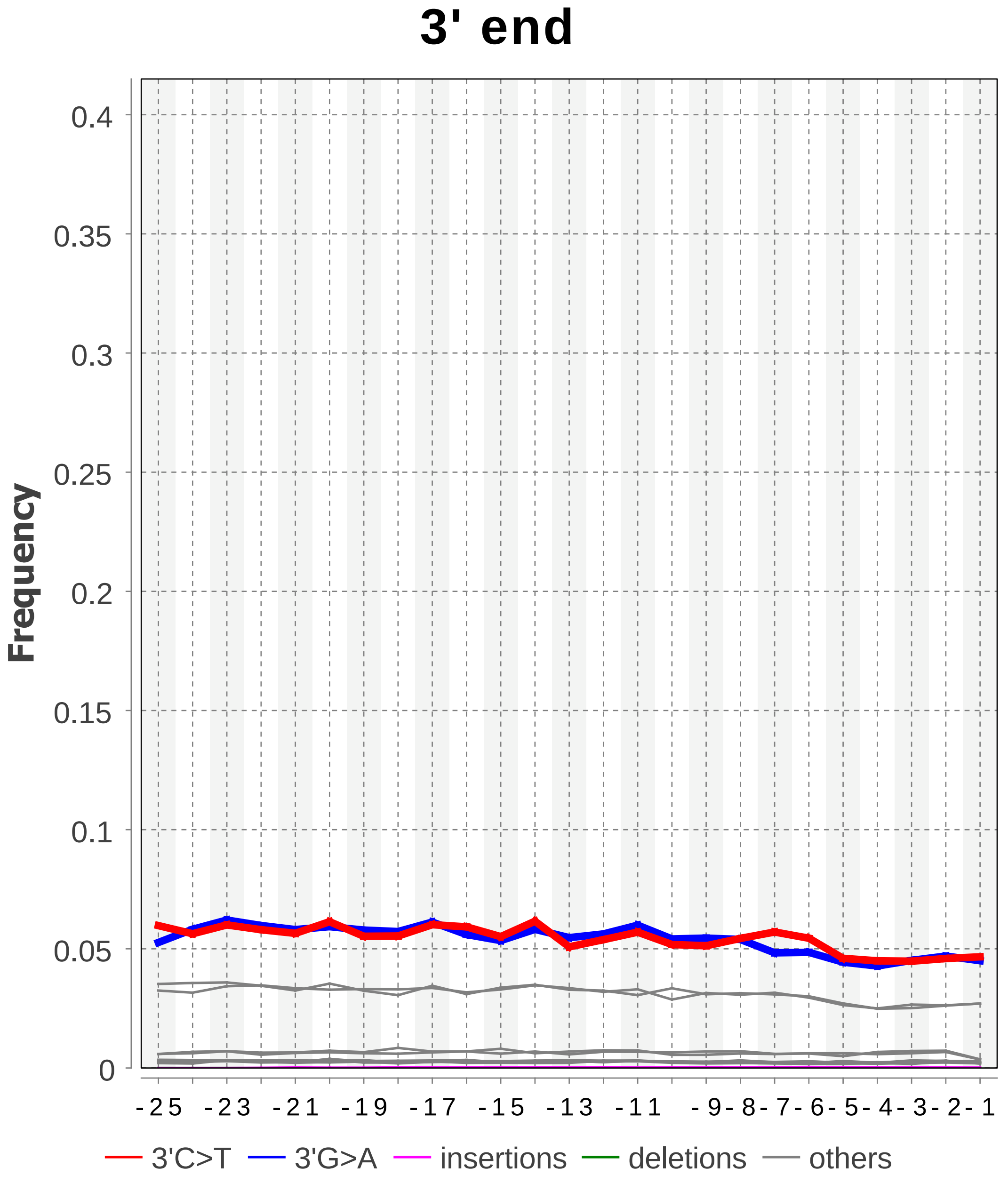

0995_MarkDuplicates

Number of used reads: 303,482 (100.0% of all input reads)

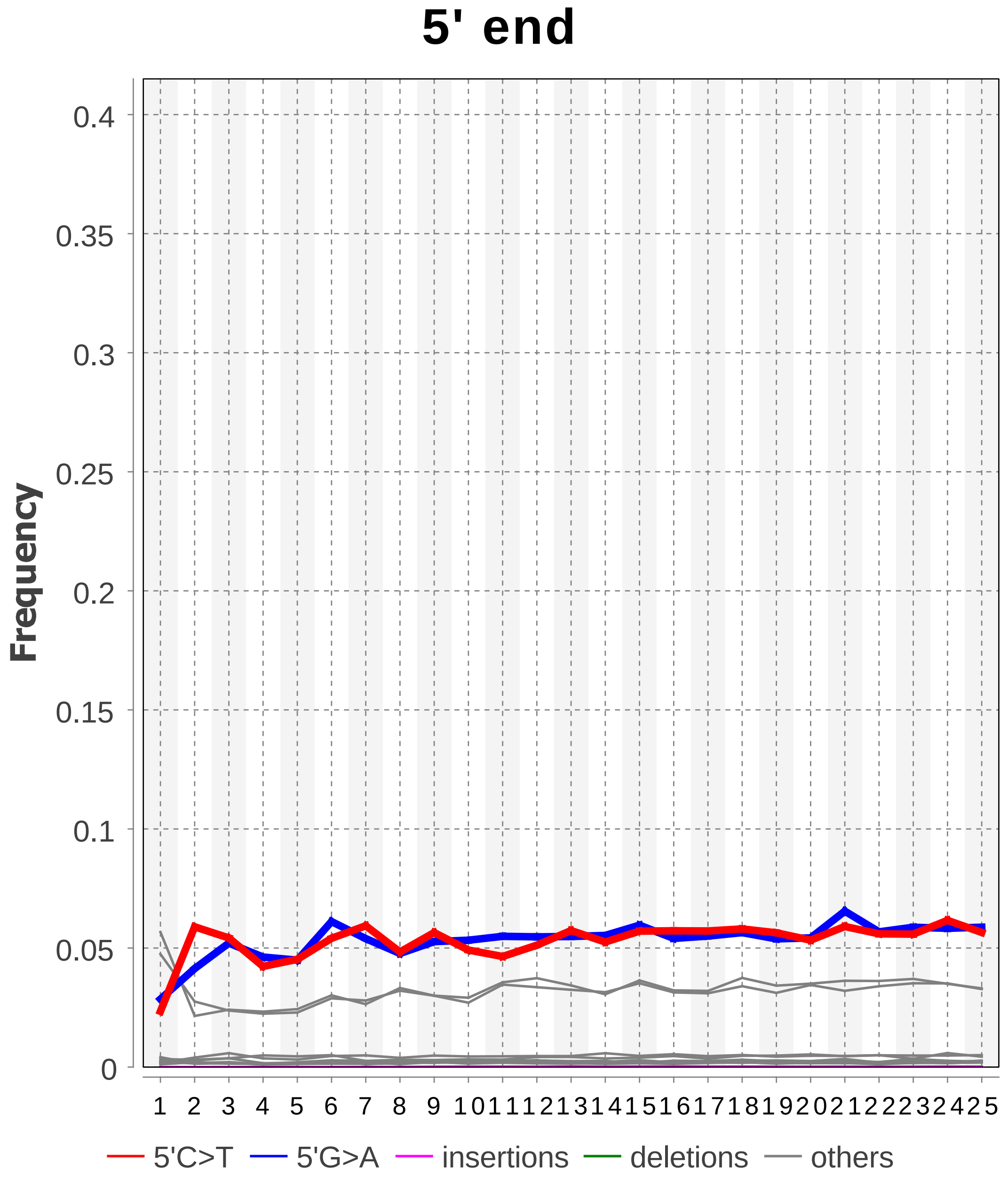

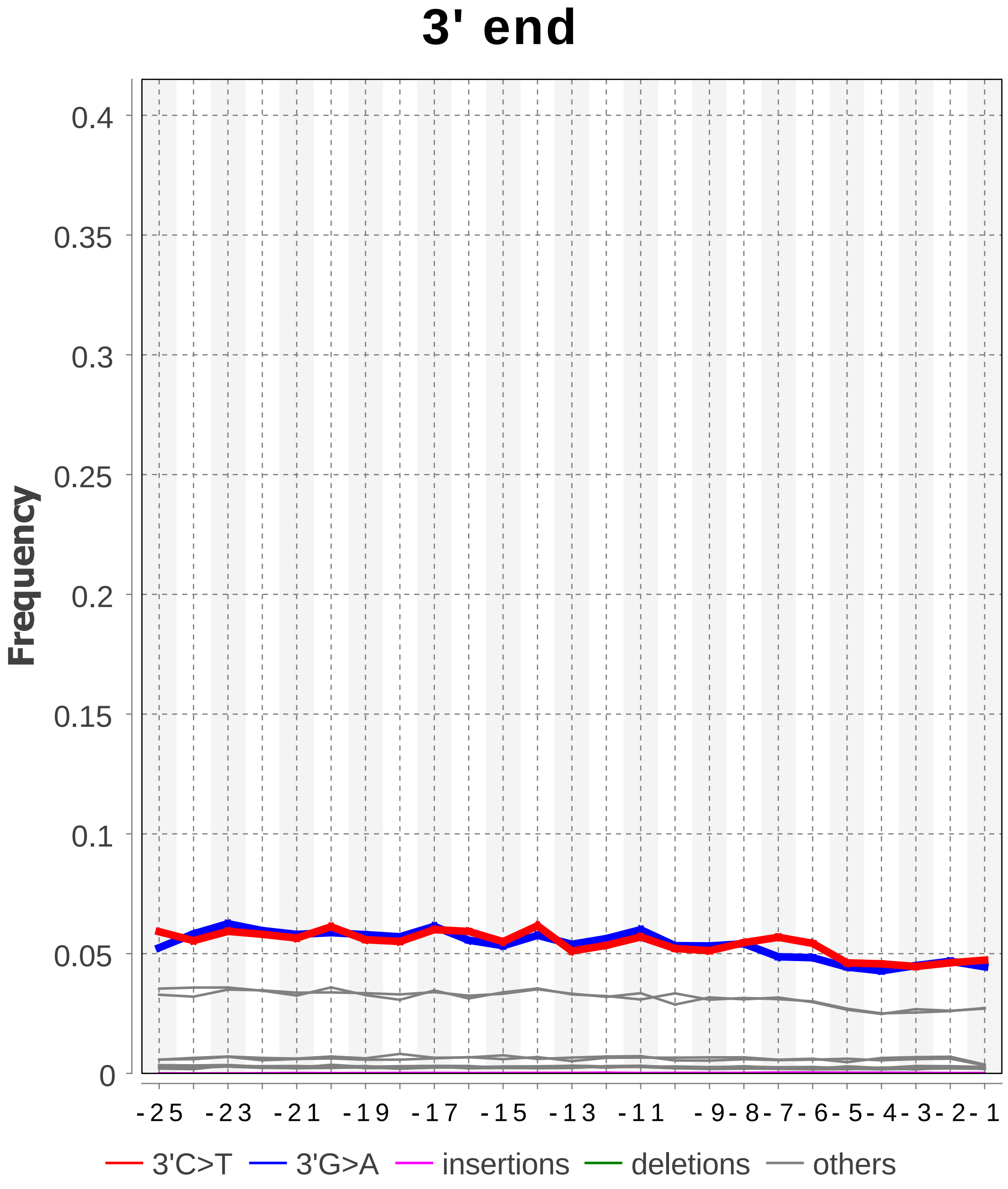

0999_aln

Number of used reads: 82,913 (100.0% of all input reads)

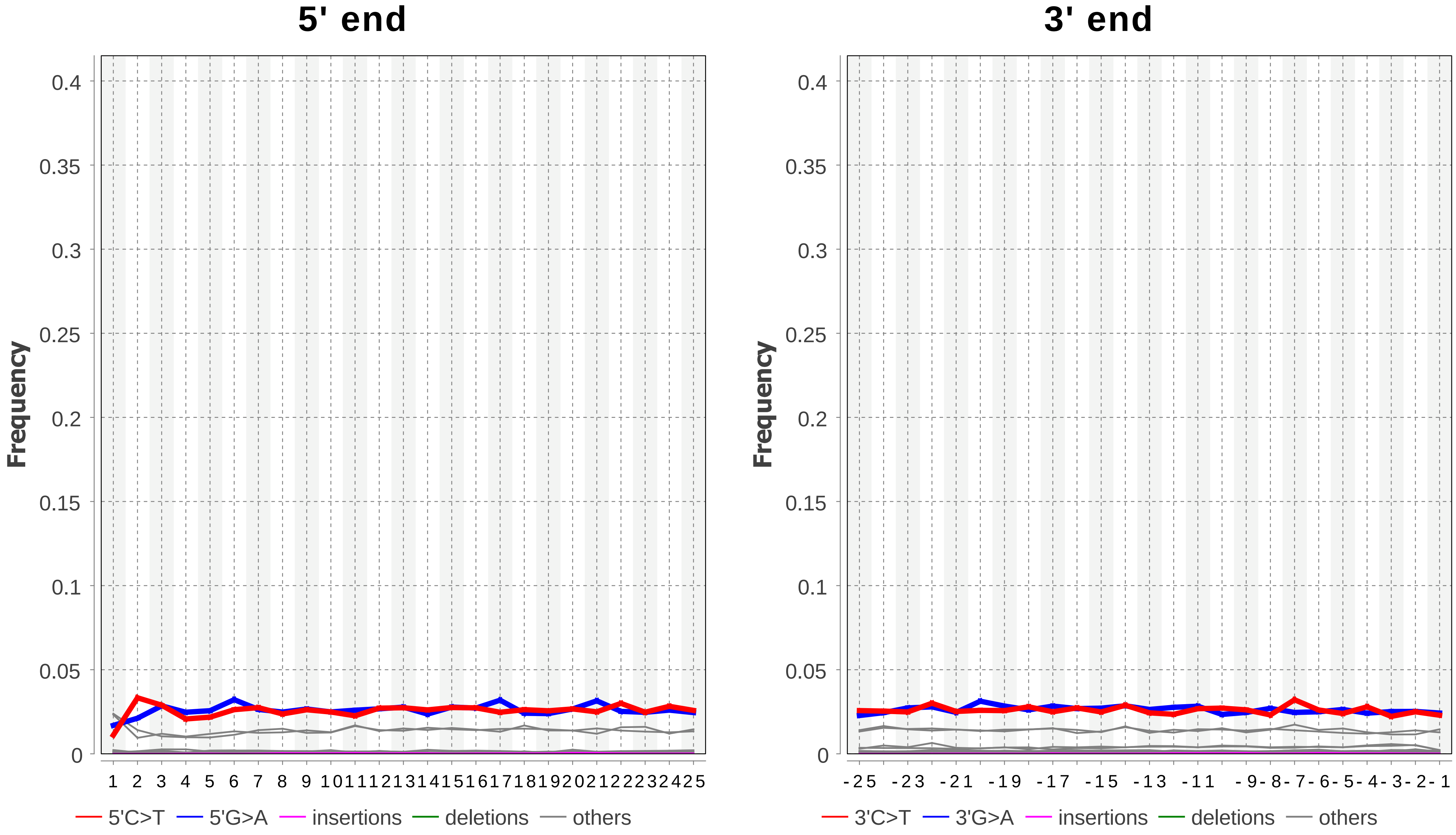

0999_MarkDuplicates
Number of used reads: 66,020 (100.0% of all input reads)

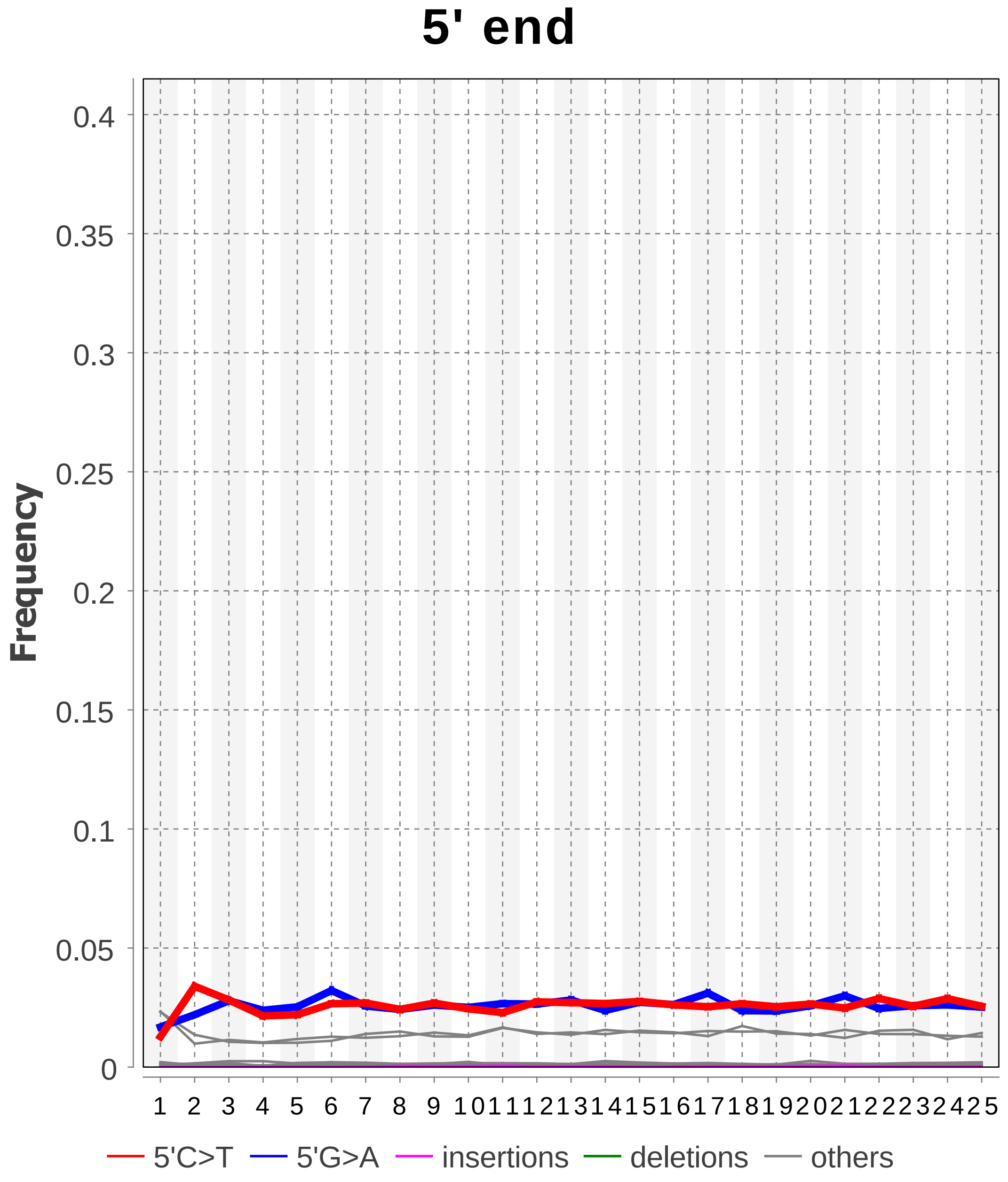

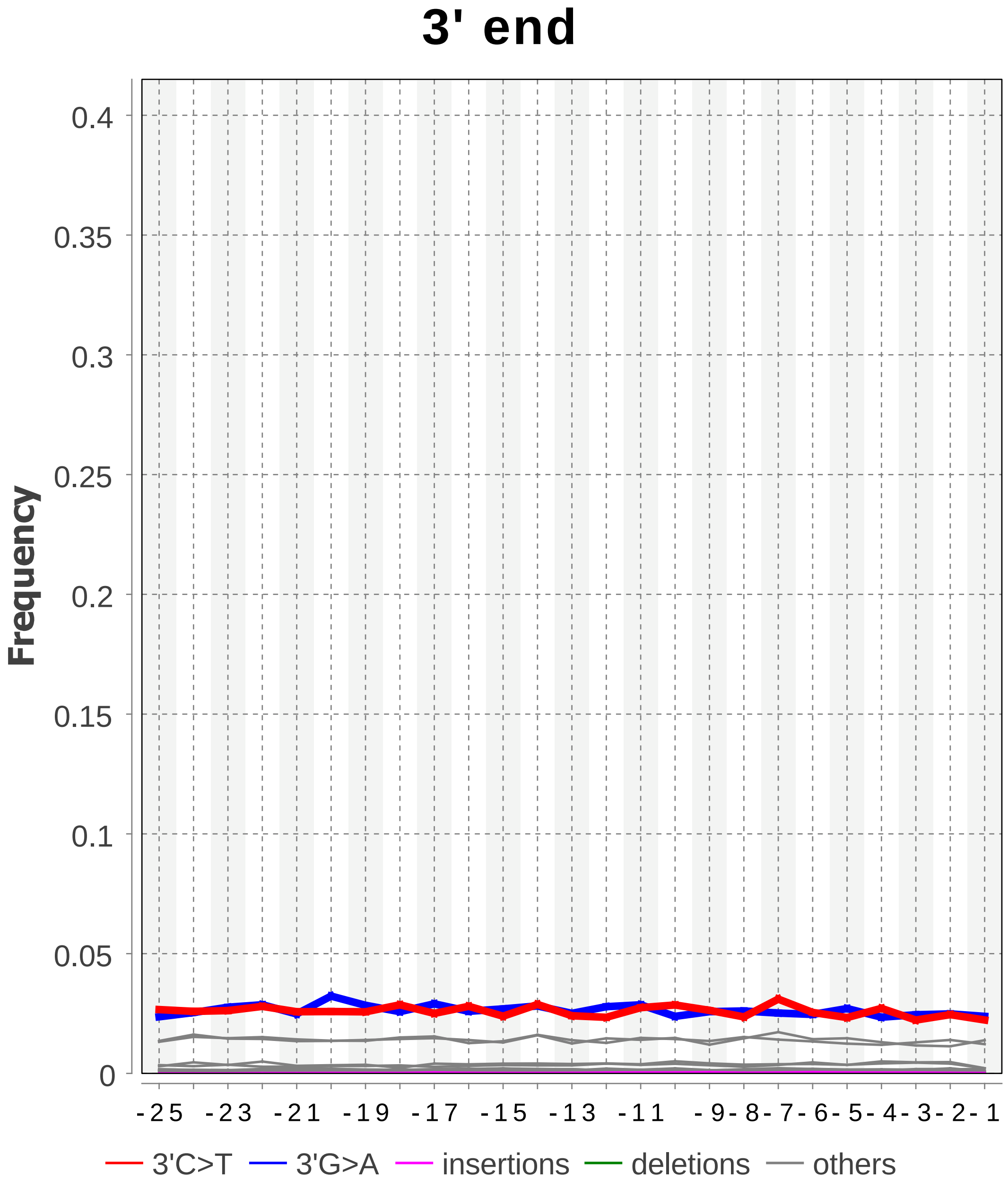

1335_aln
Number of used reads: 98,716 (100.0% of all input reads)

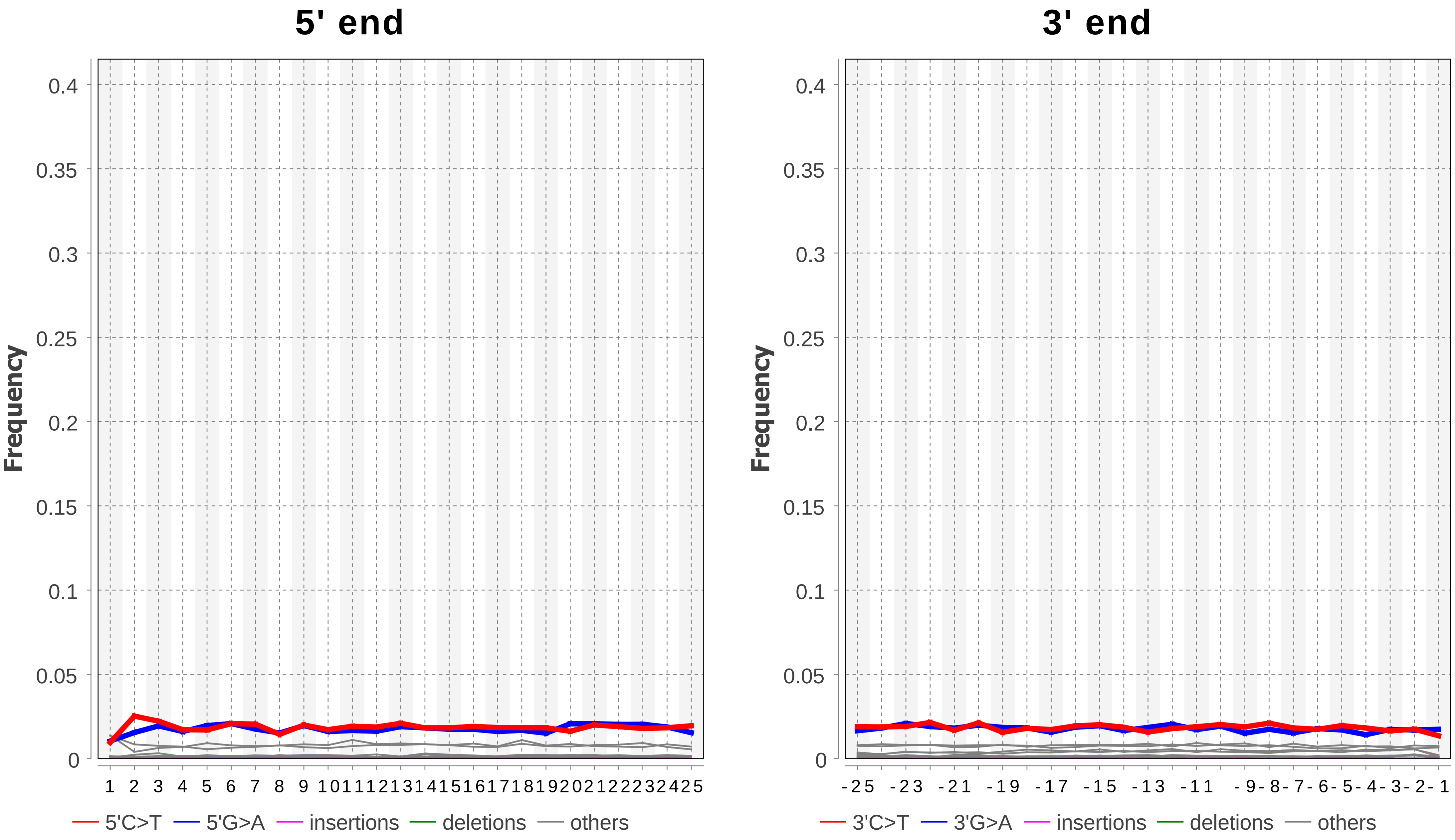

1335_MarkDuplicates

Number of used reads: 83,038 (100.0% of all input reads)

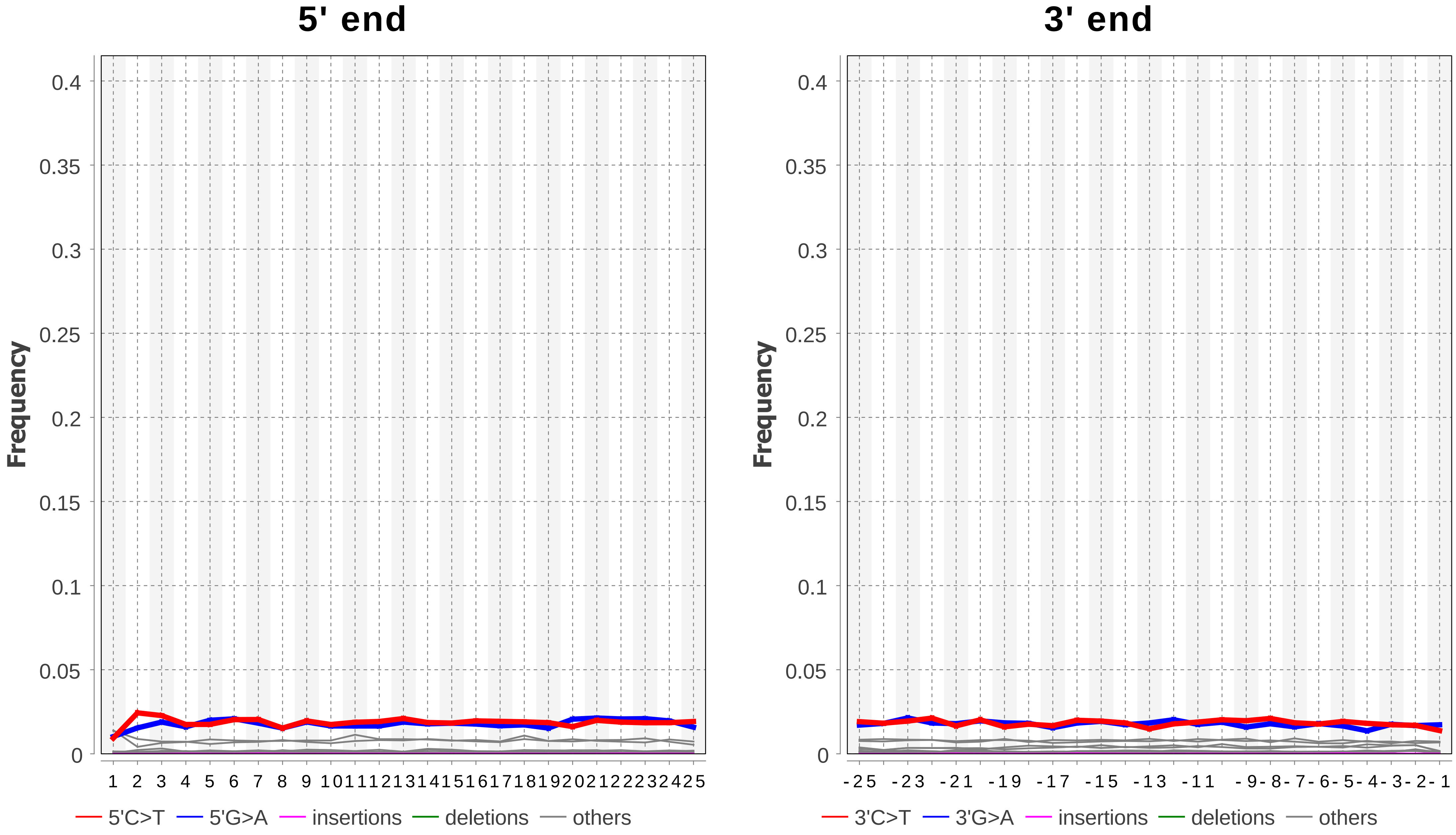

1630_aln
Number of used reads: 88,206 (100.0% of all input reads)

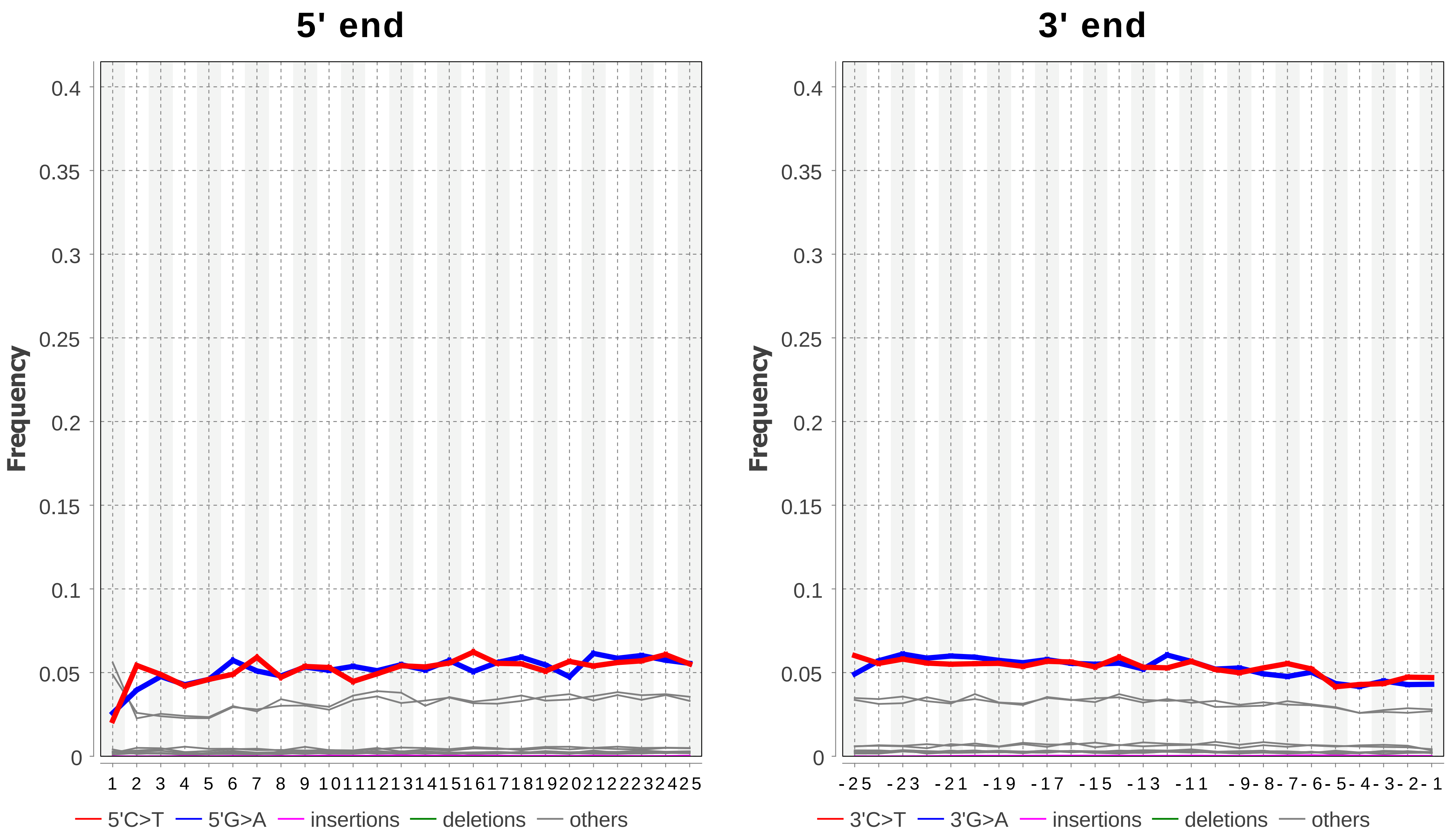

1630_MarkDuplicates
Number of used reads: 75,051 (100.0% of all input reads)

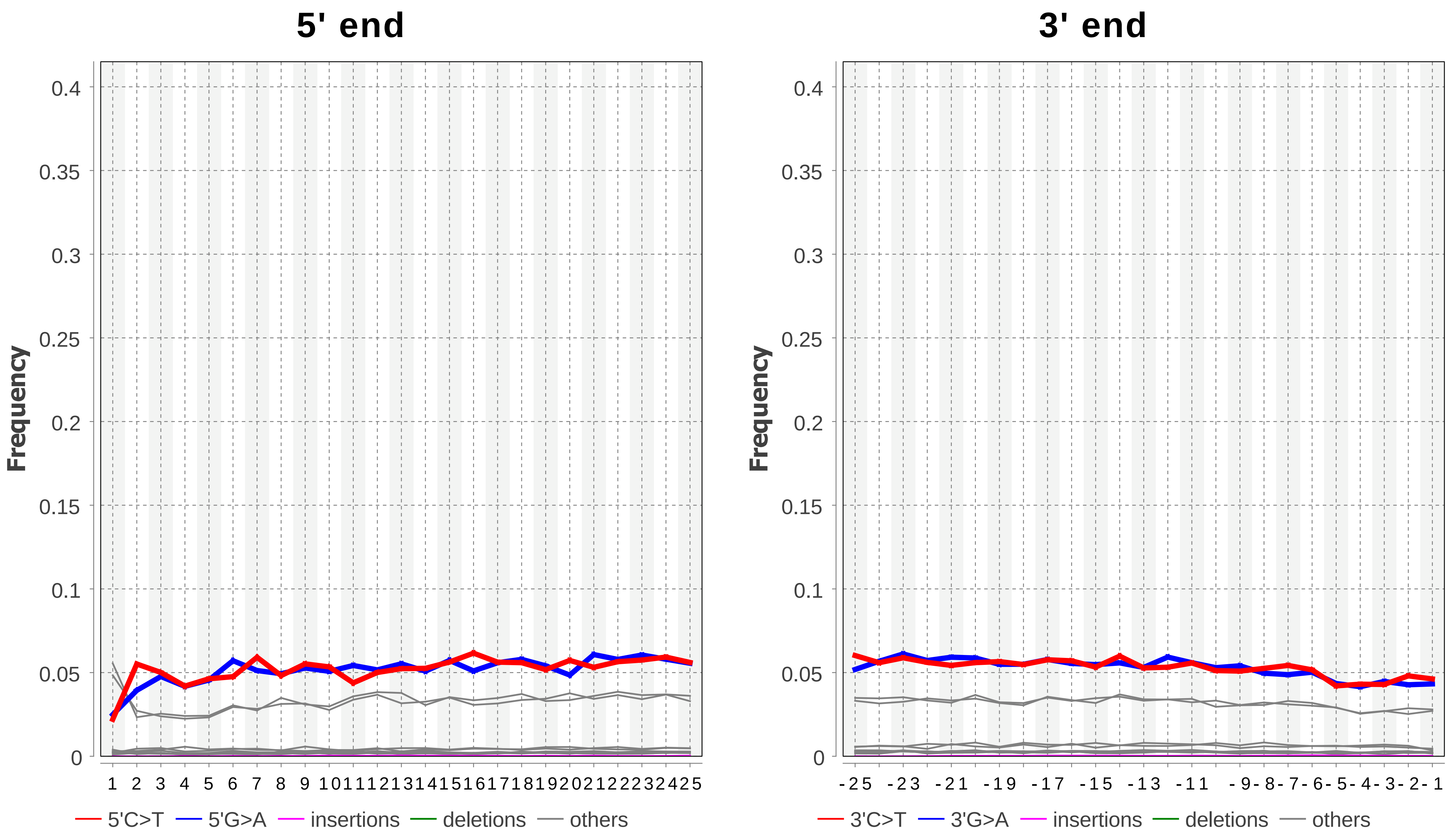

1631_aln

Number of used reads: 120,801 (100.0% of all input reads)

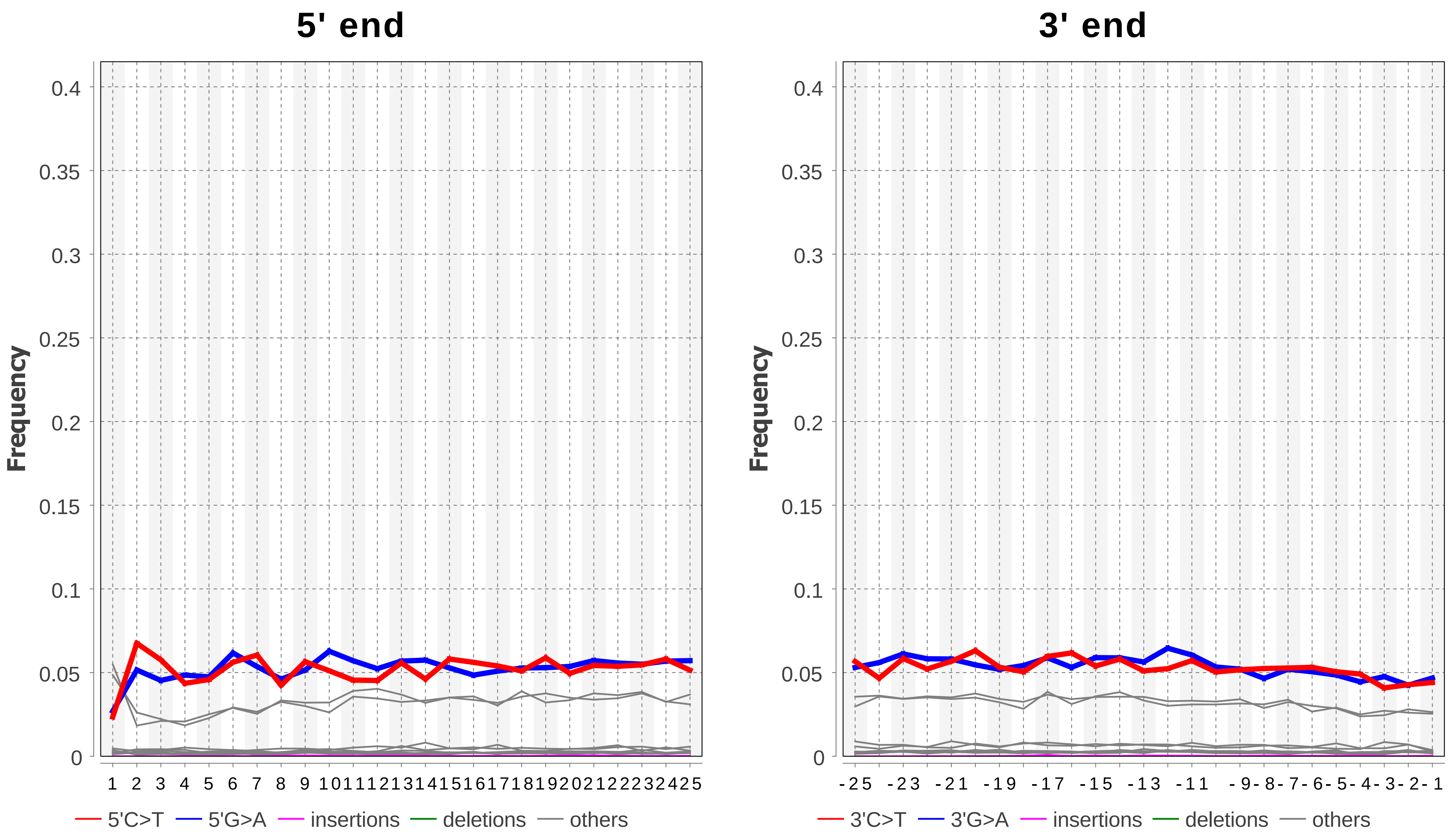

1631_MarkDuplicates

Number of used reads: 50,808 (100.0% of all input reads)

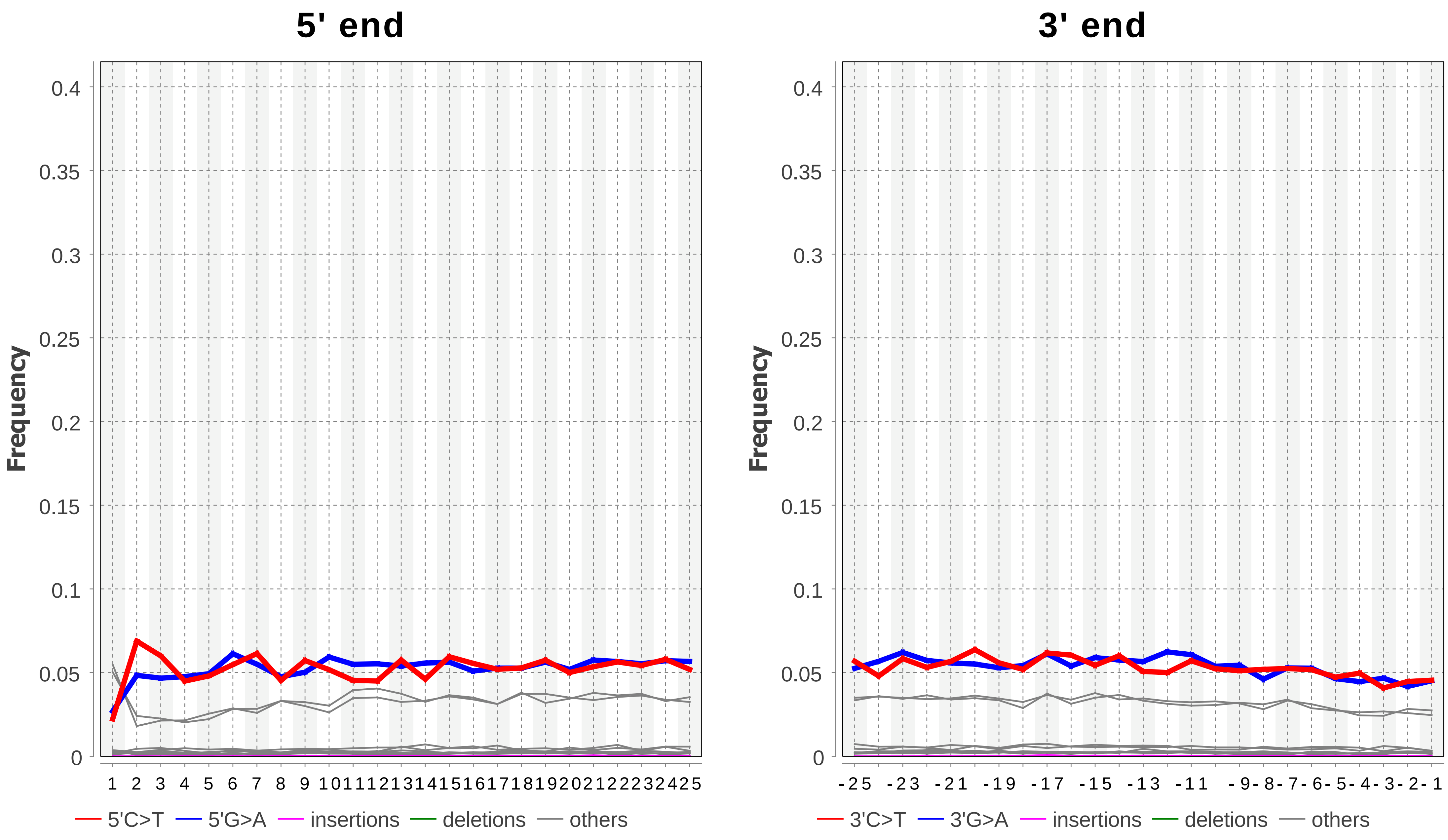

1636_aln

Number of used reads: 57,780 (100.0% of all input reads)

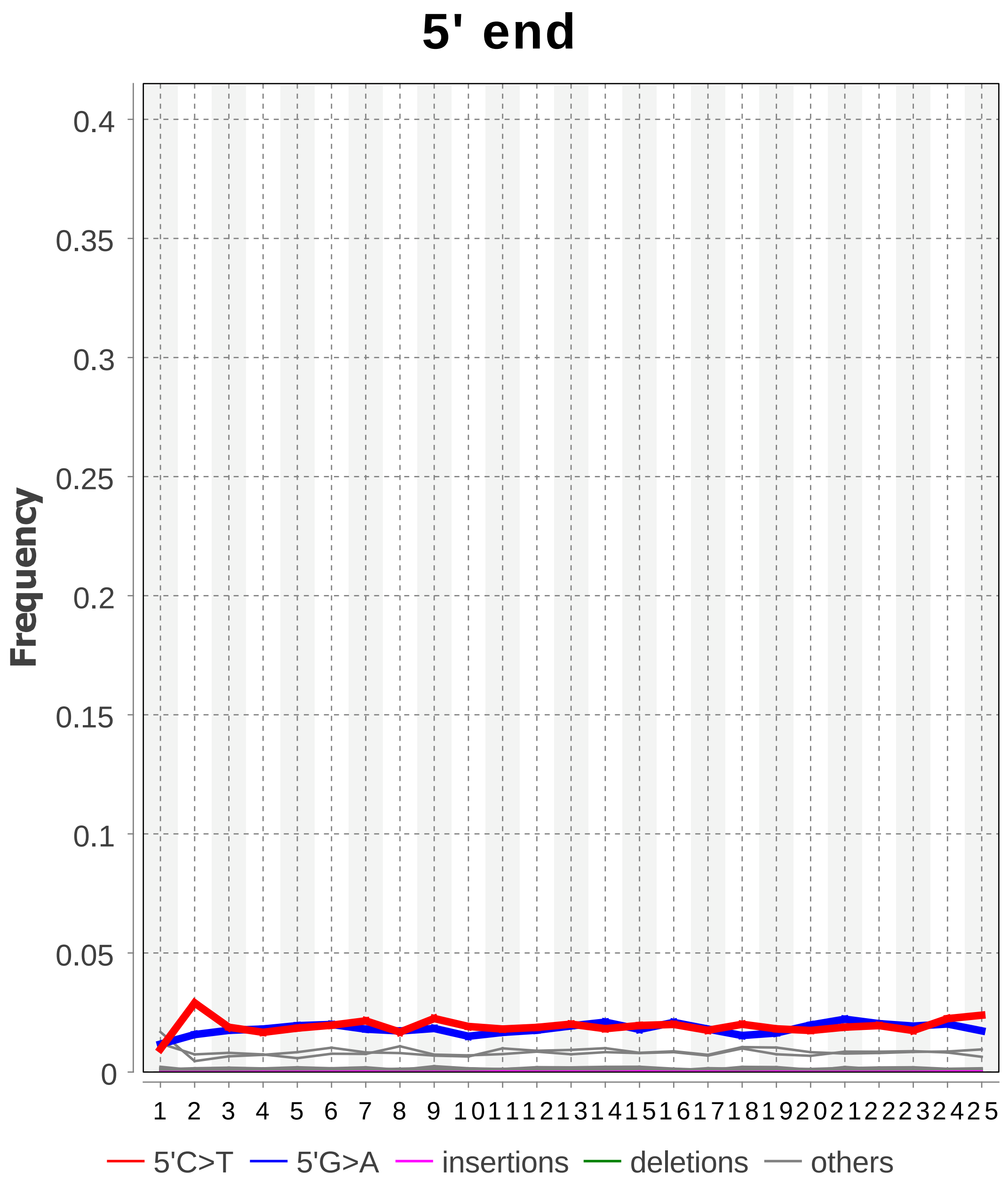

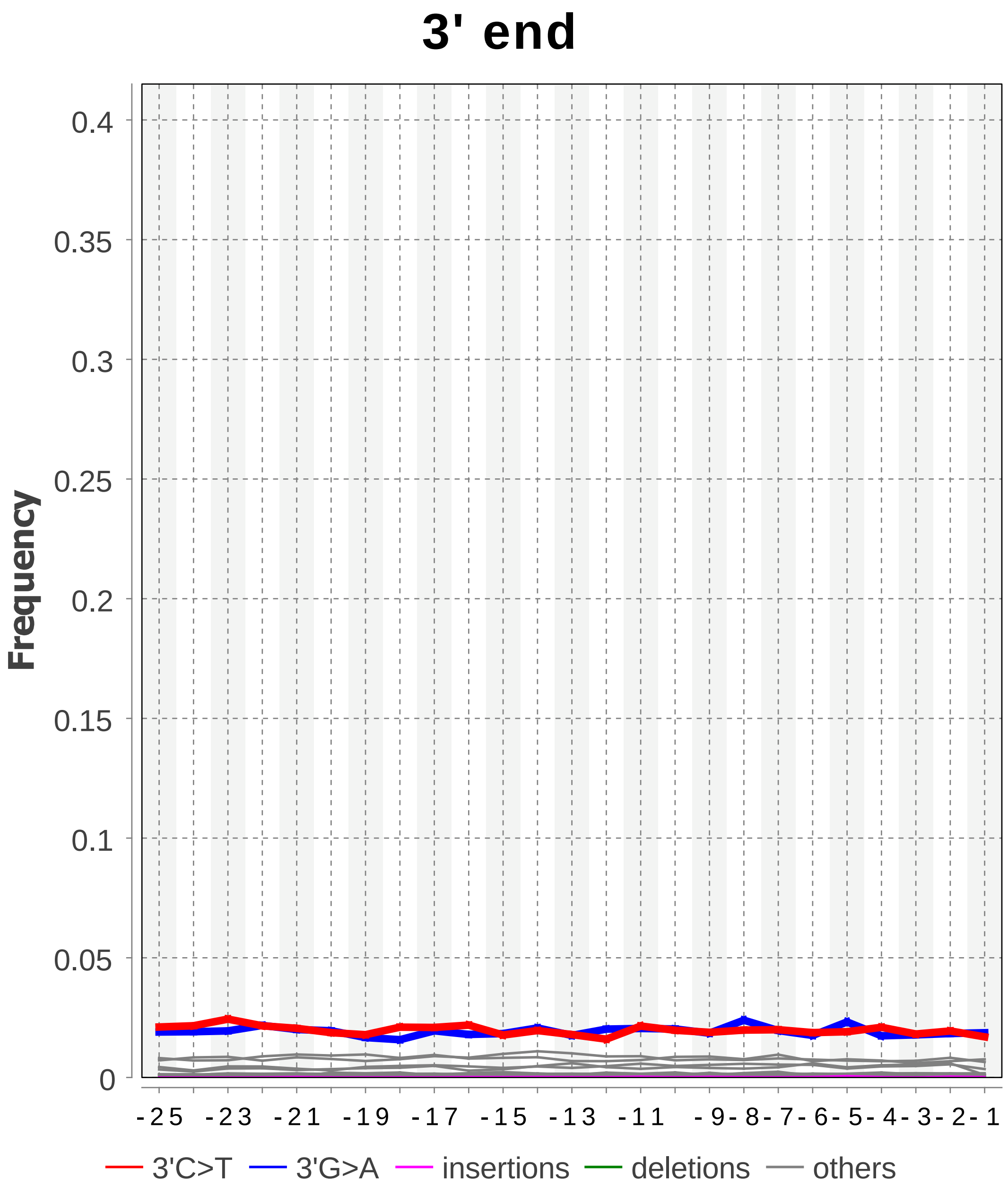

1636_MarkDuplicates
Number of used reads: 46,926 (100.0% of all input reads)

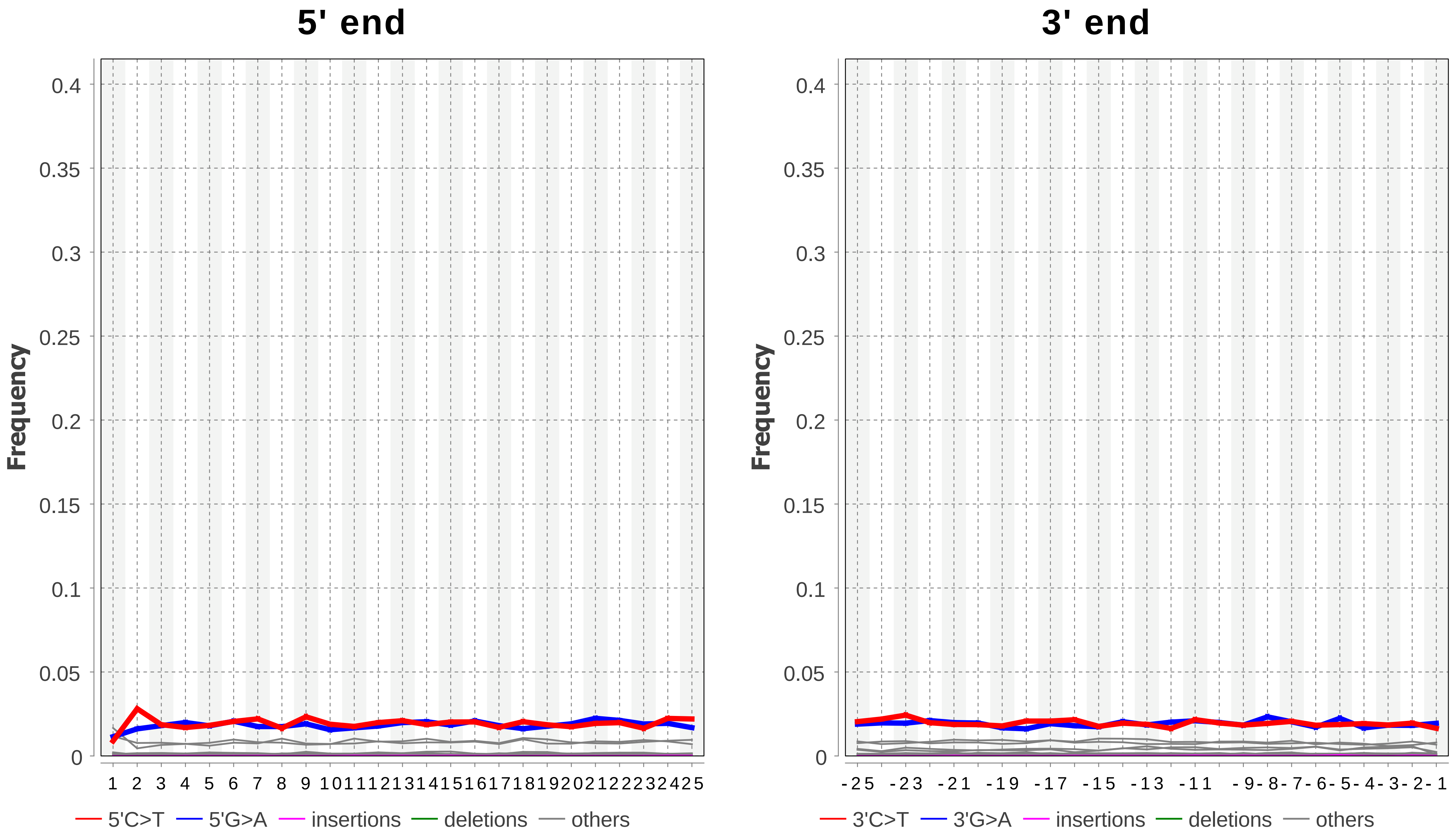

1637_aln
Number of used reads: 64,051 (100.0% of all input reads)

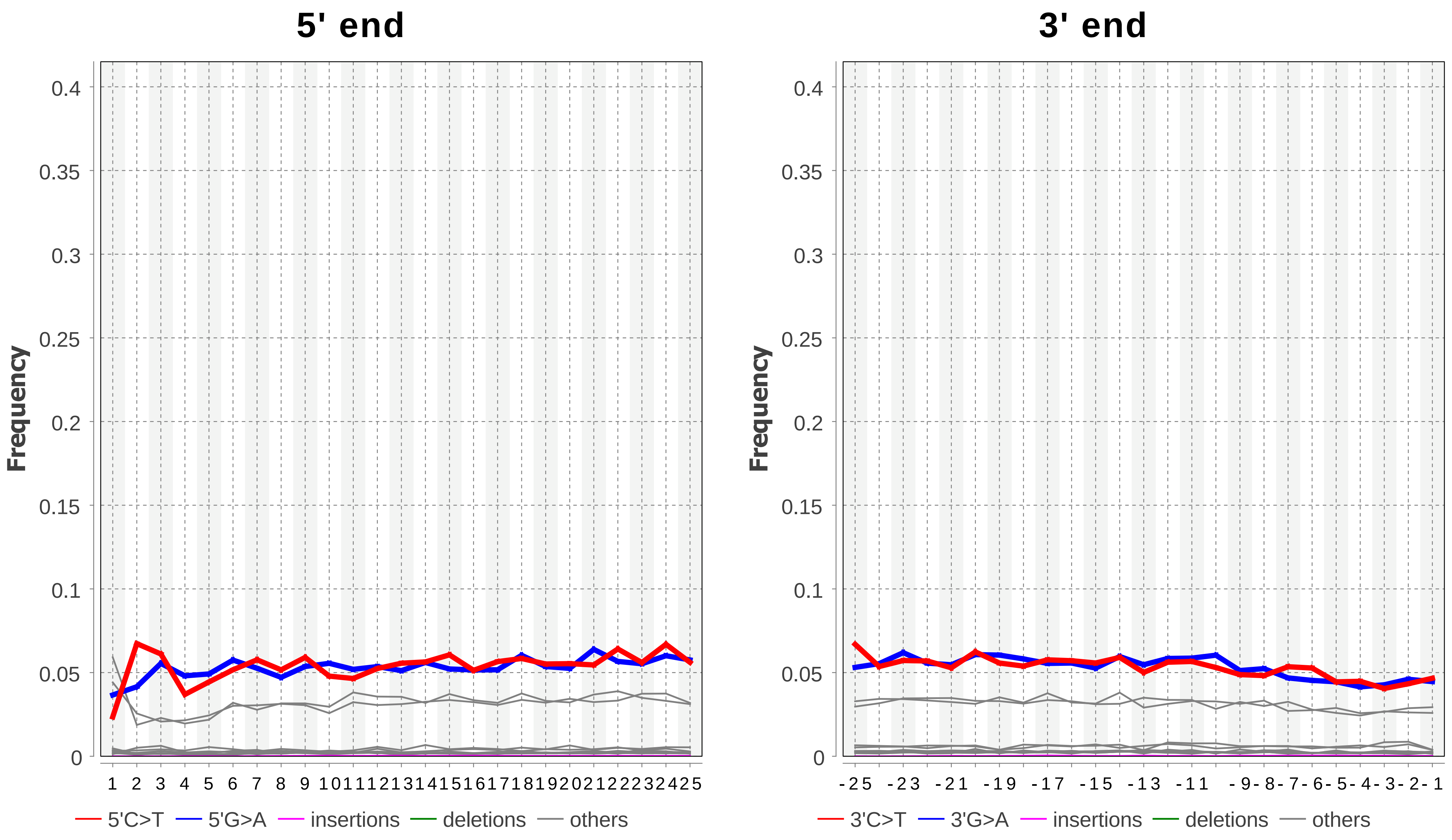

1637_MarkDuplicates

Number of used reads: 49,577 (100.0% of all input reads)

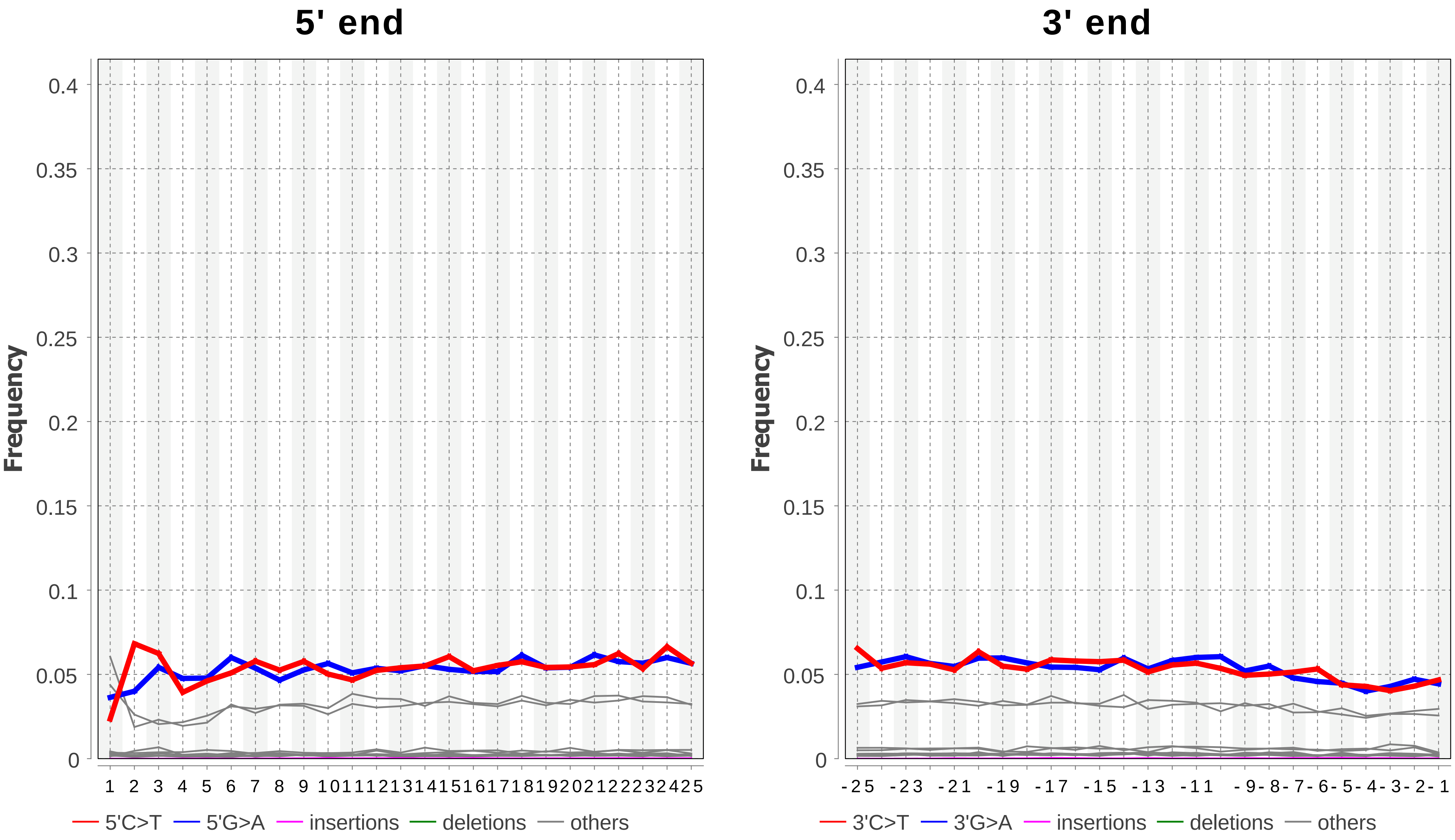

1638_aln

Number of used reads: 31,929 (100.0% of all input reads)

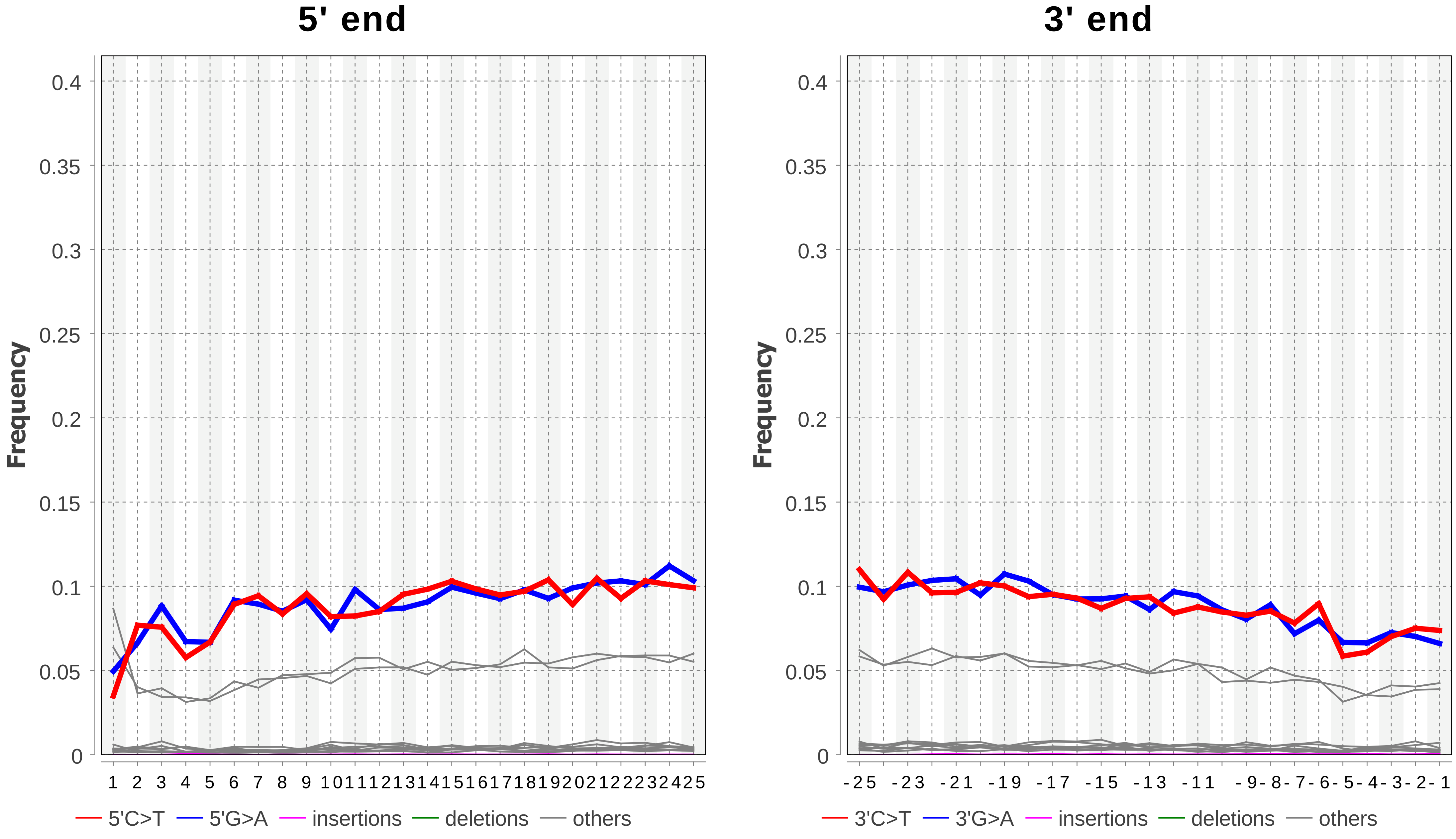

1638_MarkDuplicates
Number of used reads: 27,286 (100.0% of all input reads)

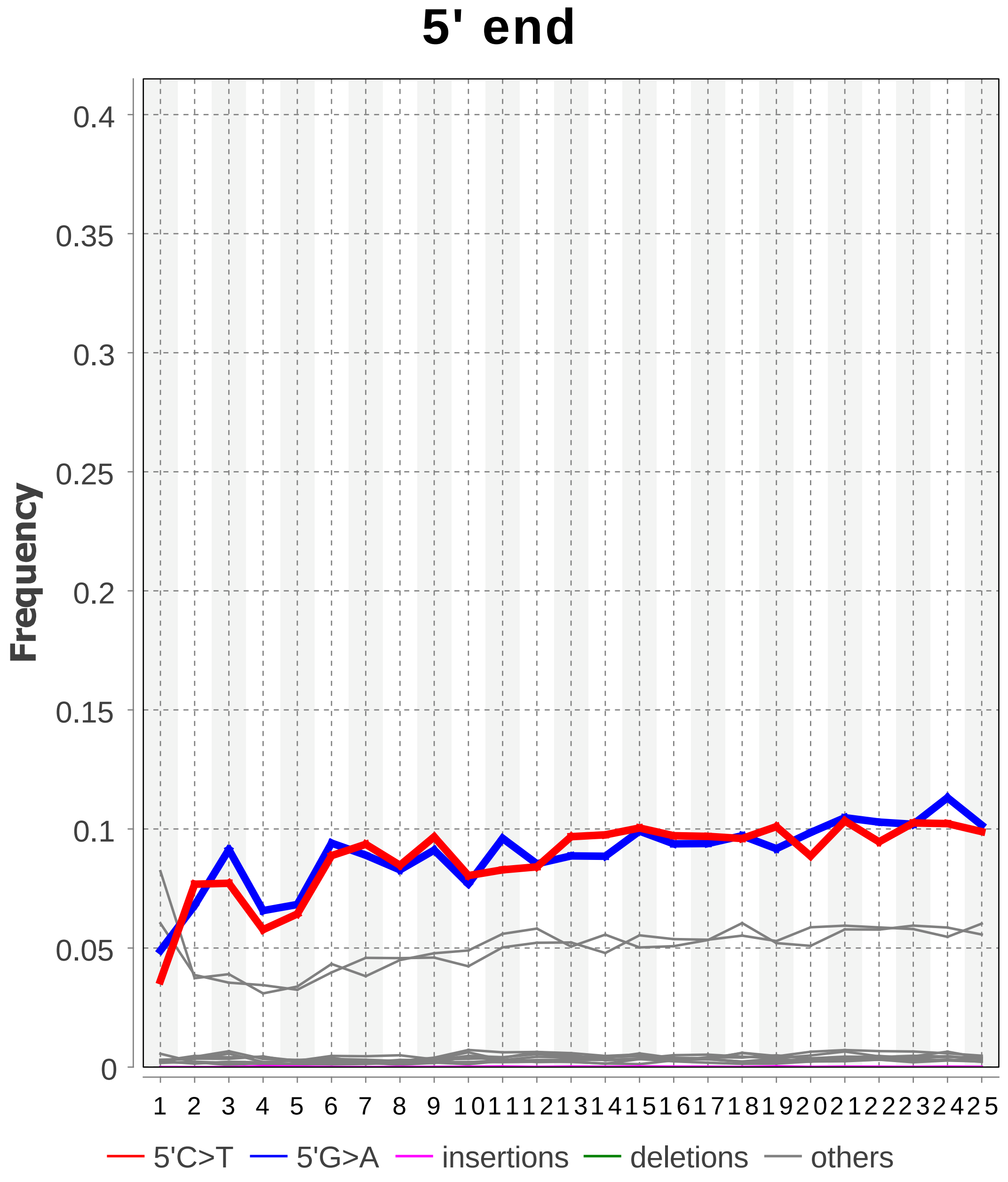

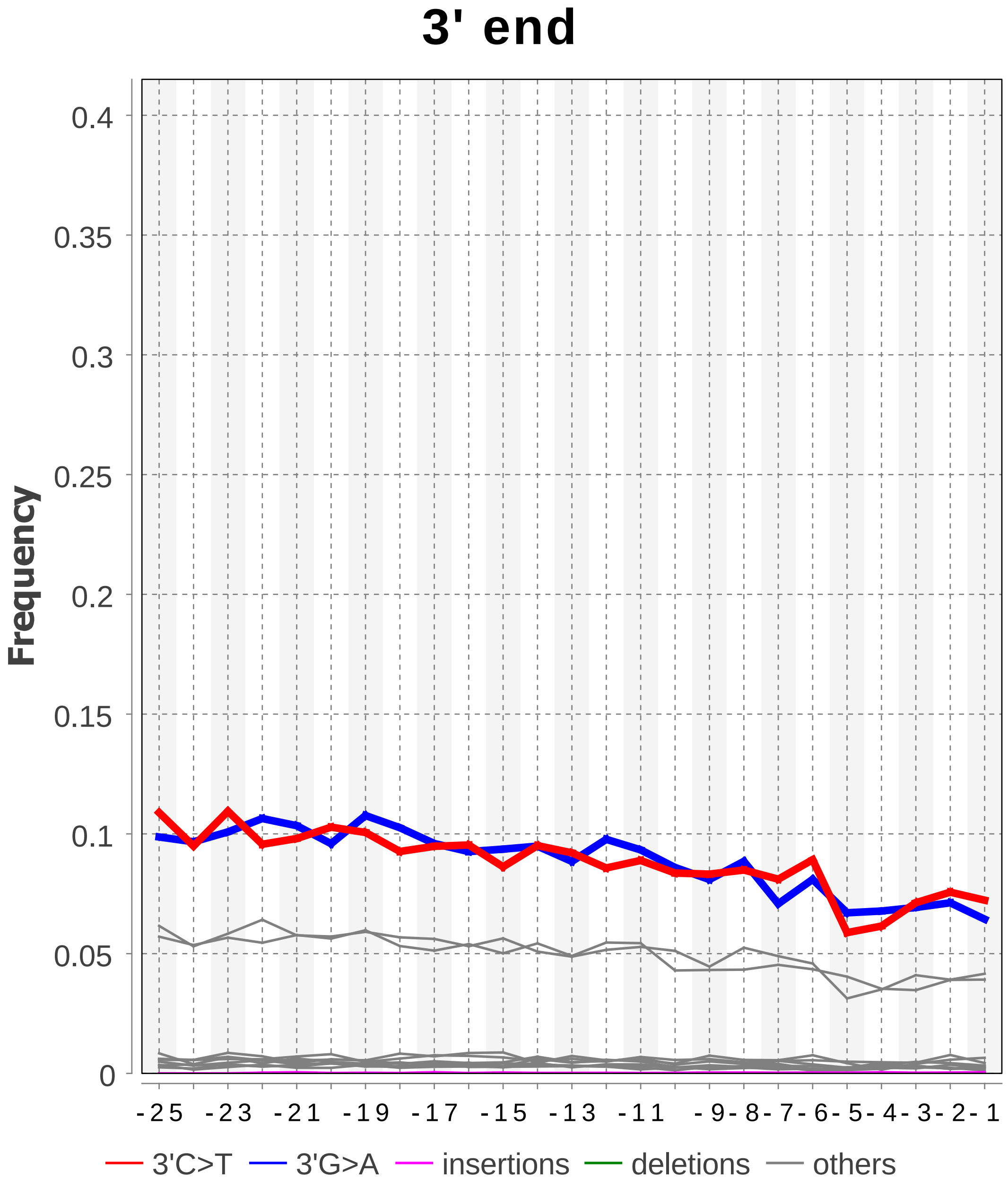

1640_aln

Number of used reads: 77,402 (100.0% of all input reads)

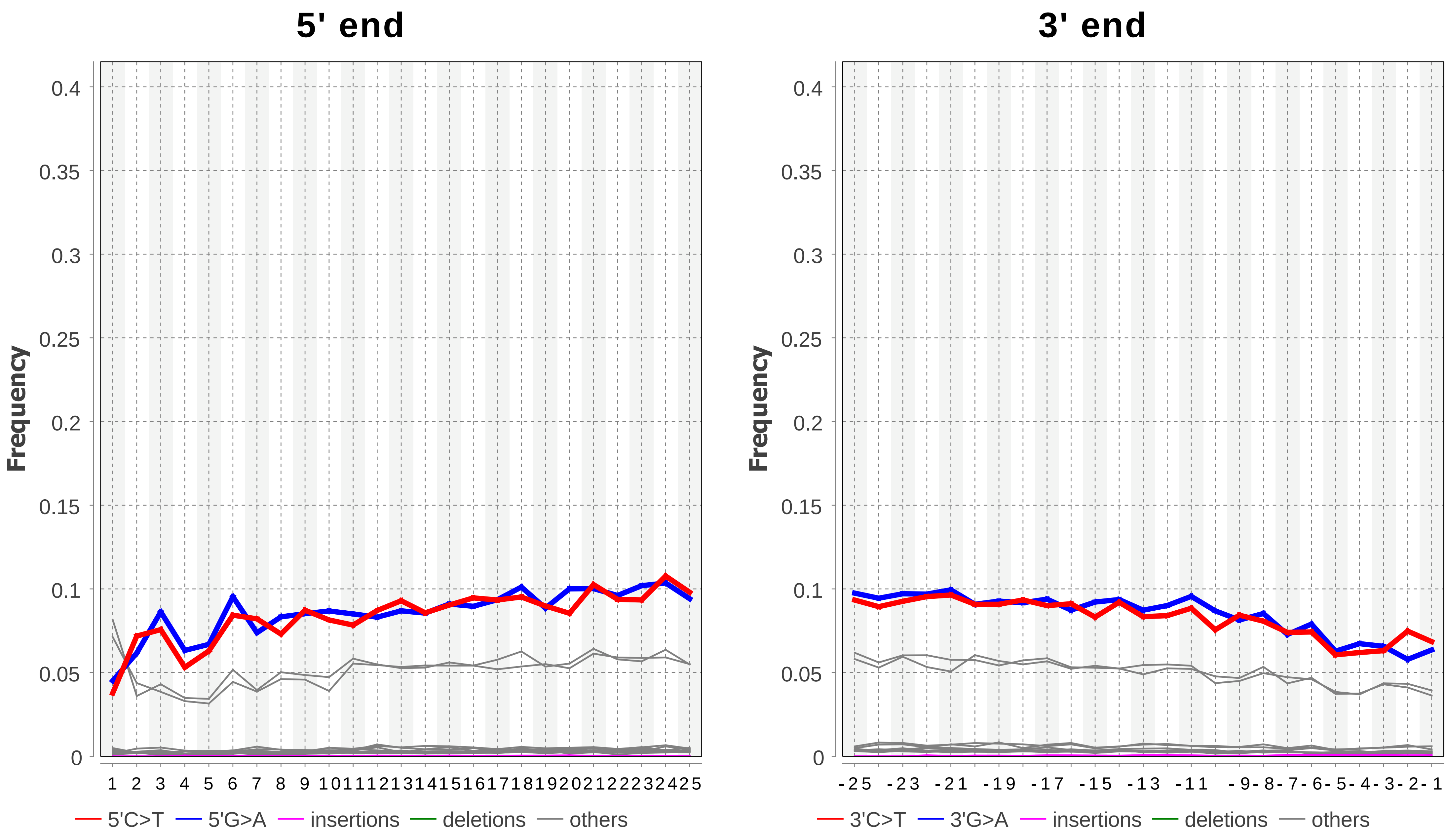

1640_MarkDuplicates
Number of used reads: 63,954 (100.0% of all input reads)

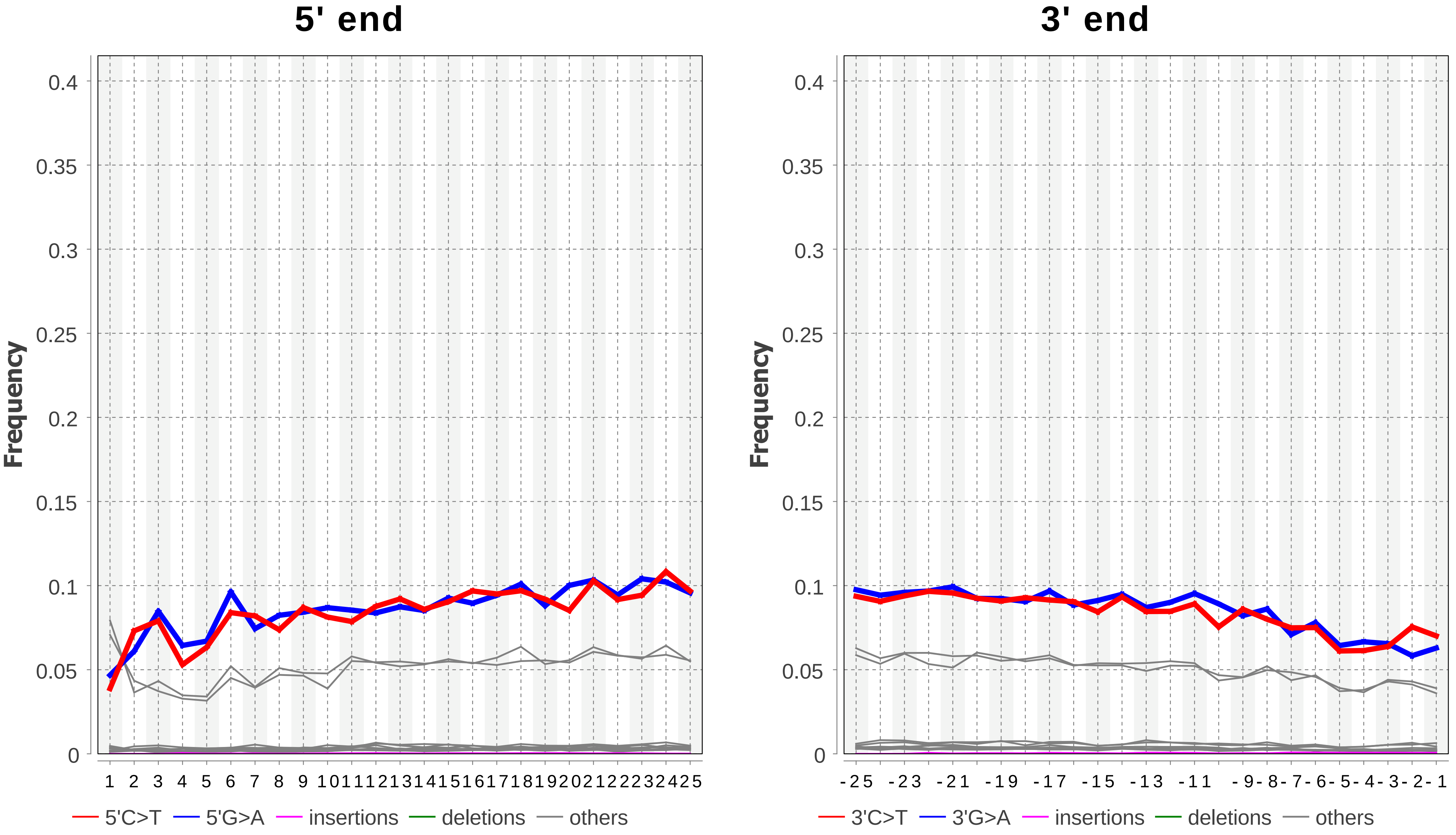

1645_aln
Number of used reads: 200,889 (100.0% of all input reads)

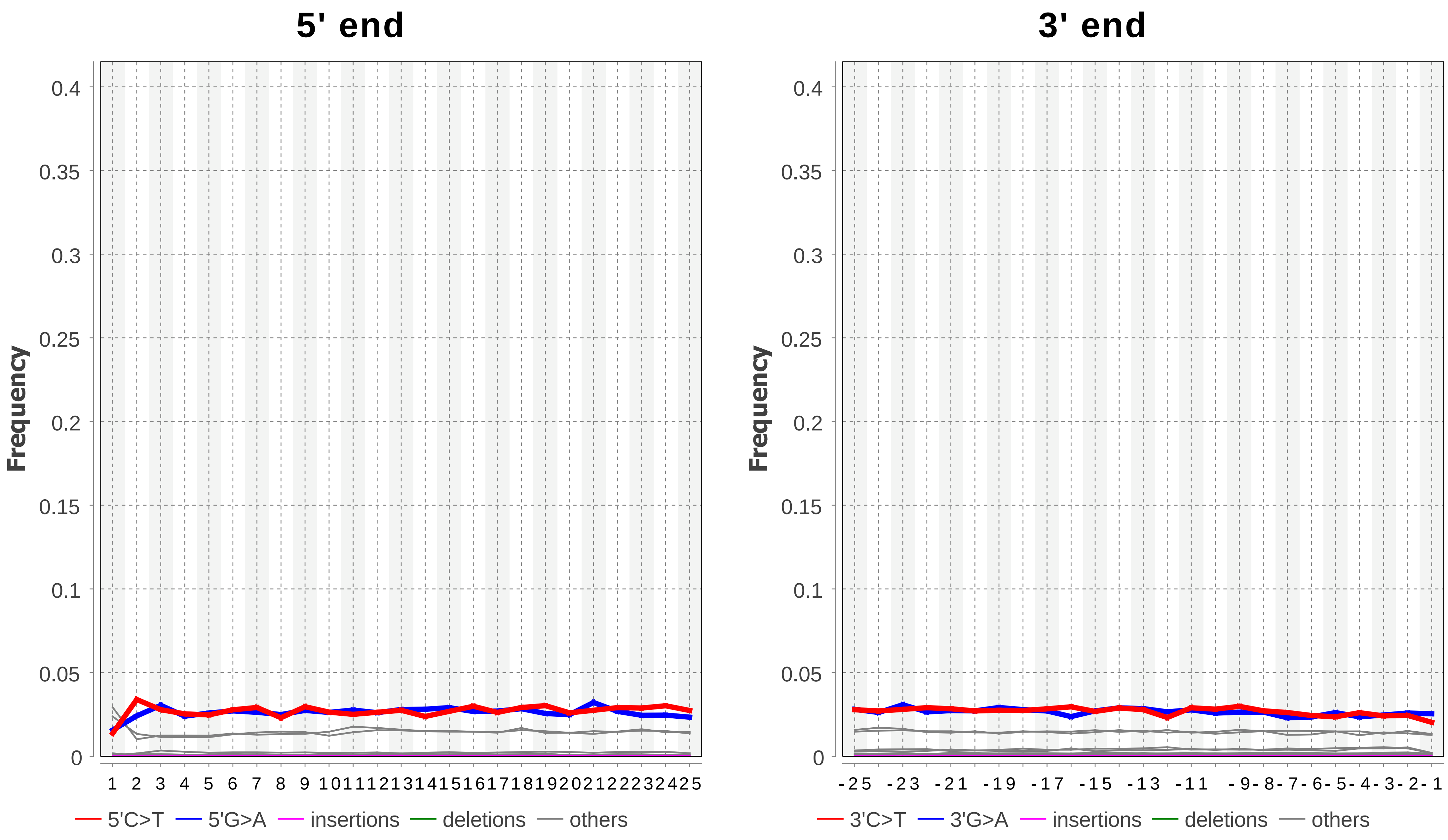

1645_MarkDuplicates
Number of used reads: 154,197 (100.0% of all input reads)

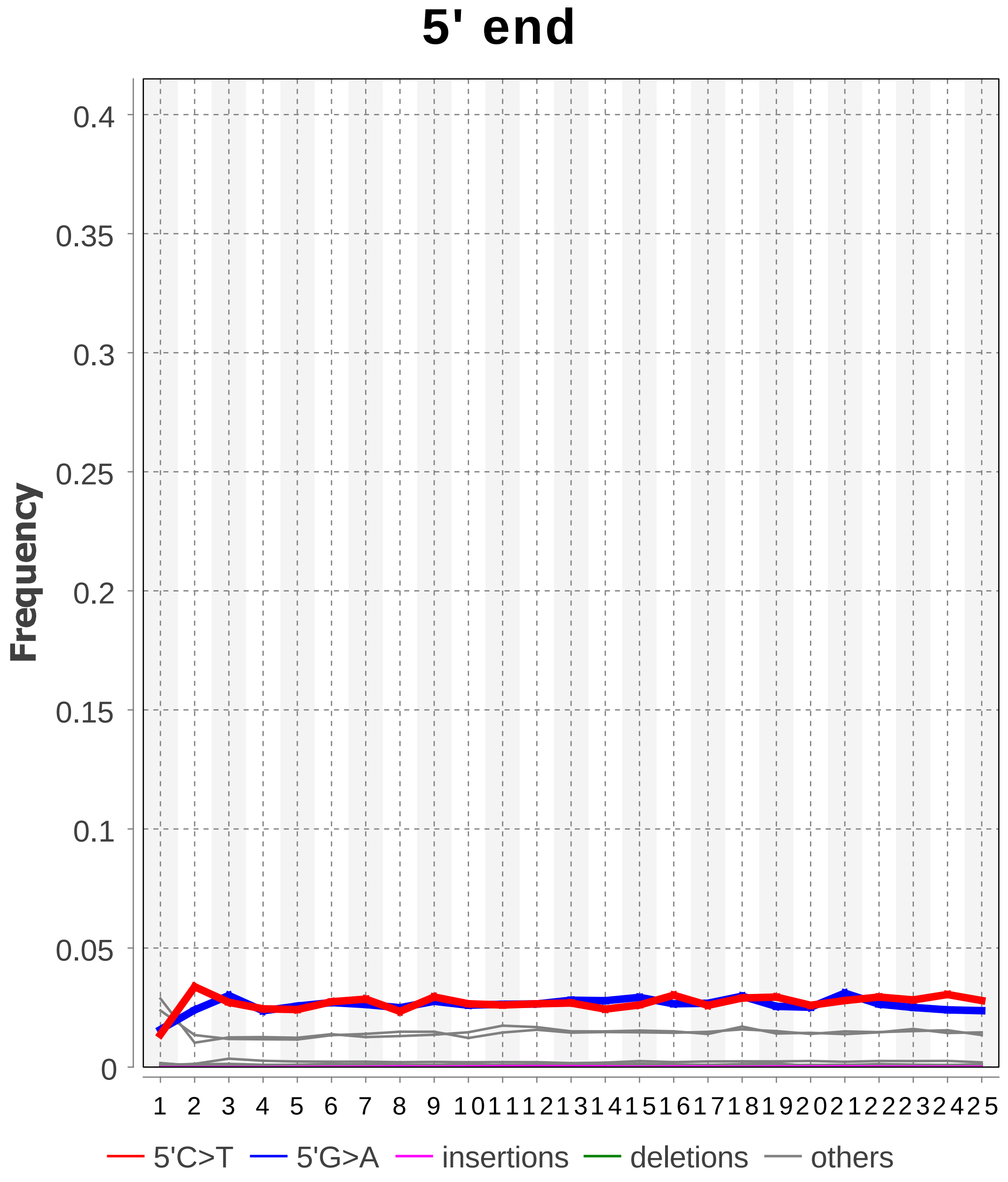

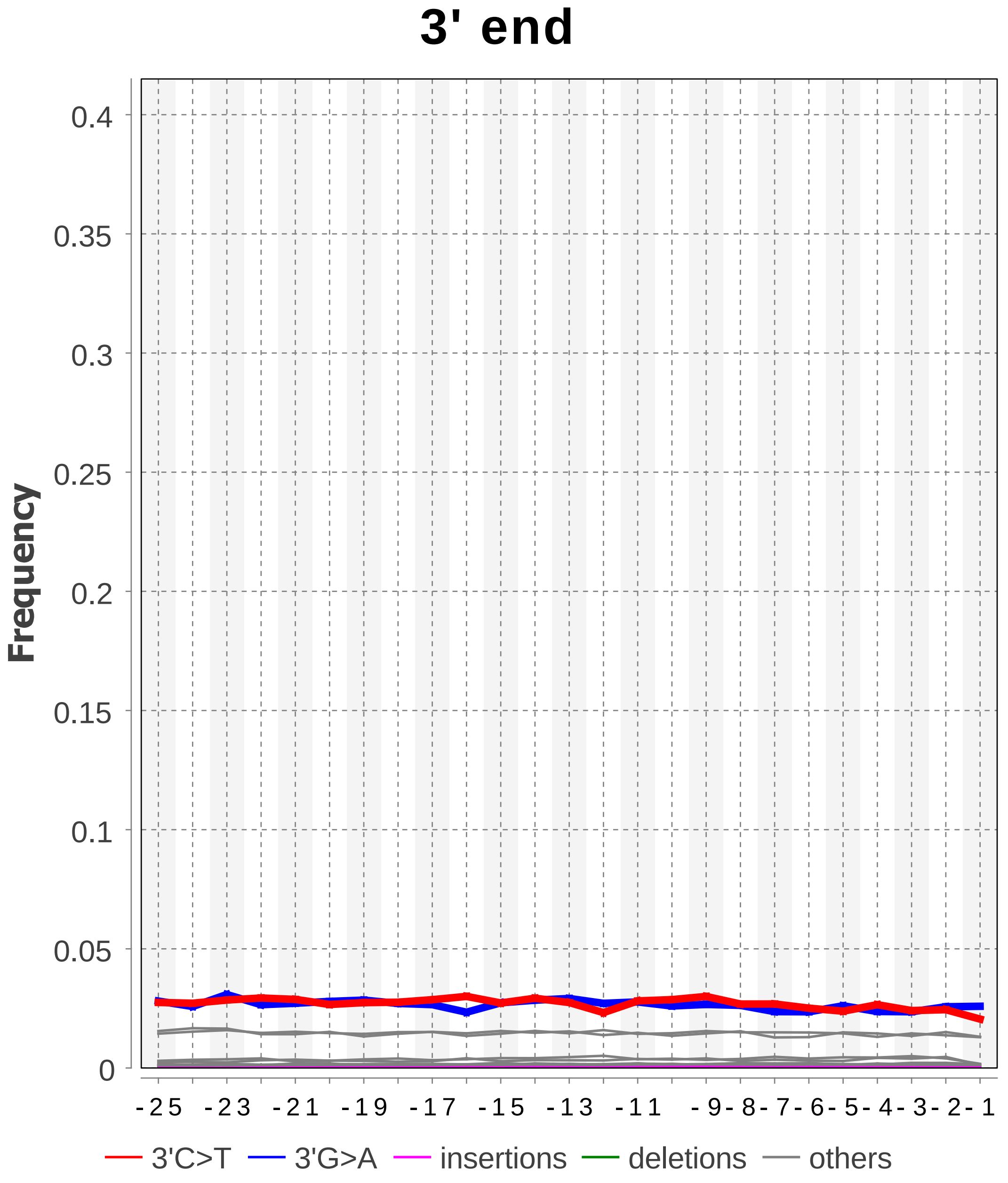

1647_aln
Number of used reads: 59,079 (100.0% of all input reads)

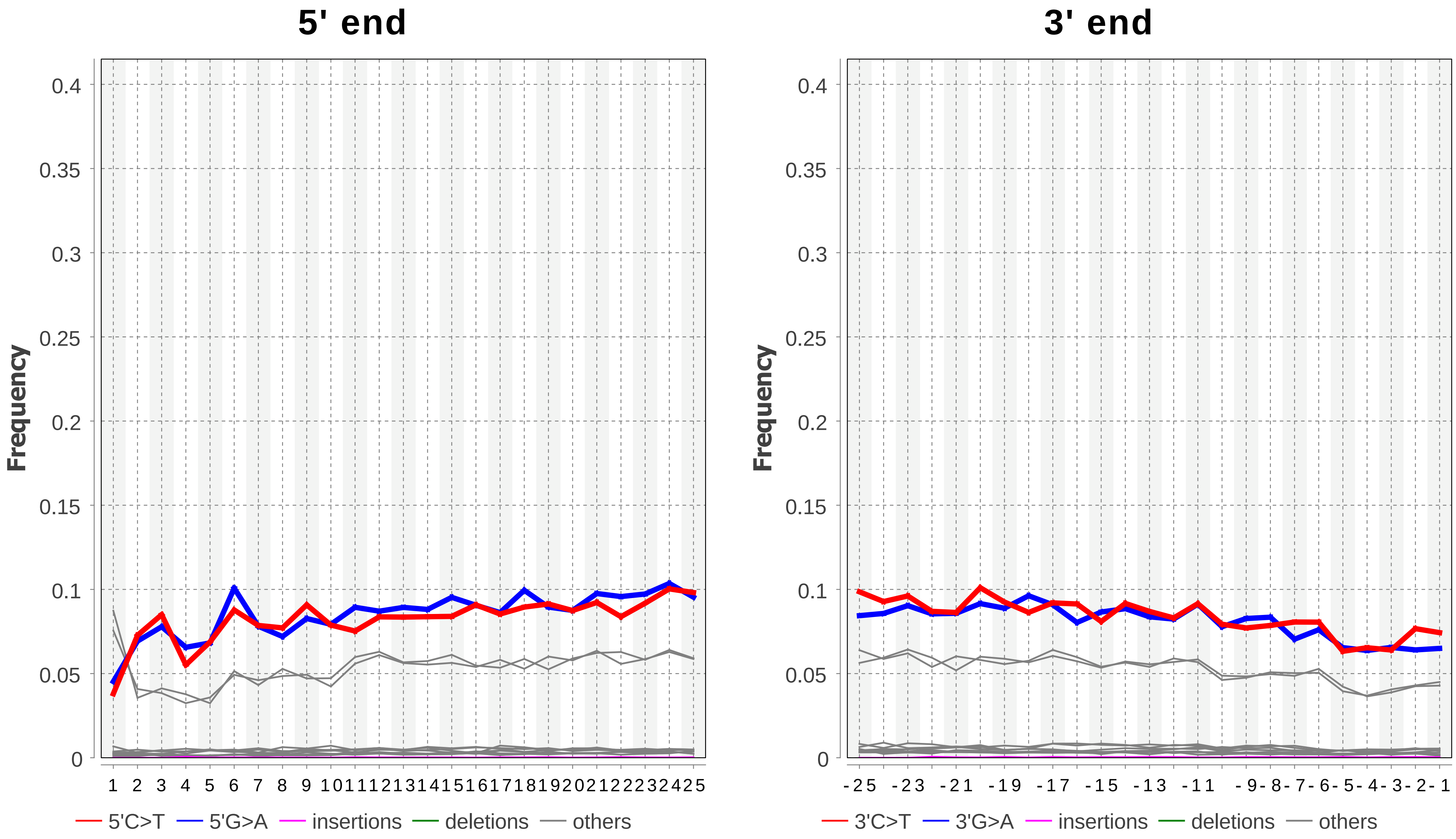

1647_MarkDuplicates
Number of used reads: 48,904 (100.0% of all input reads)

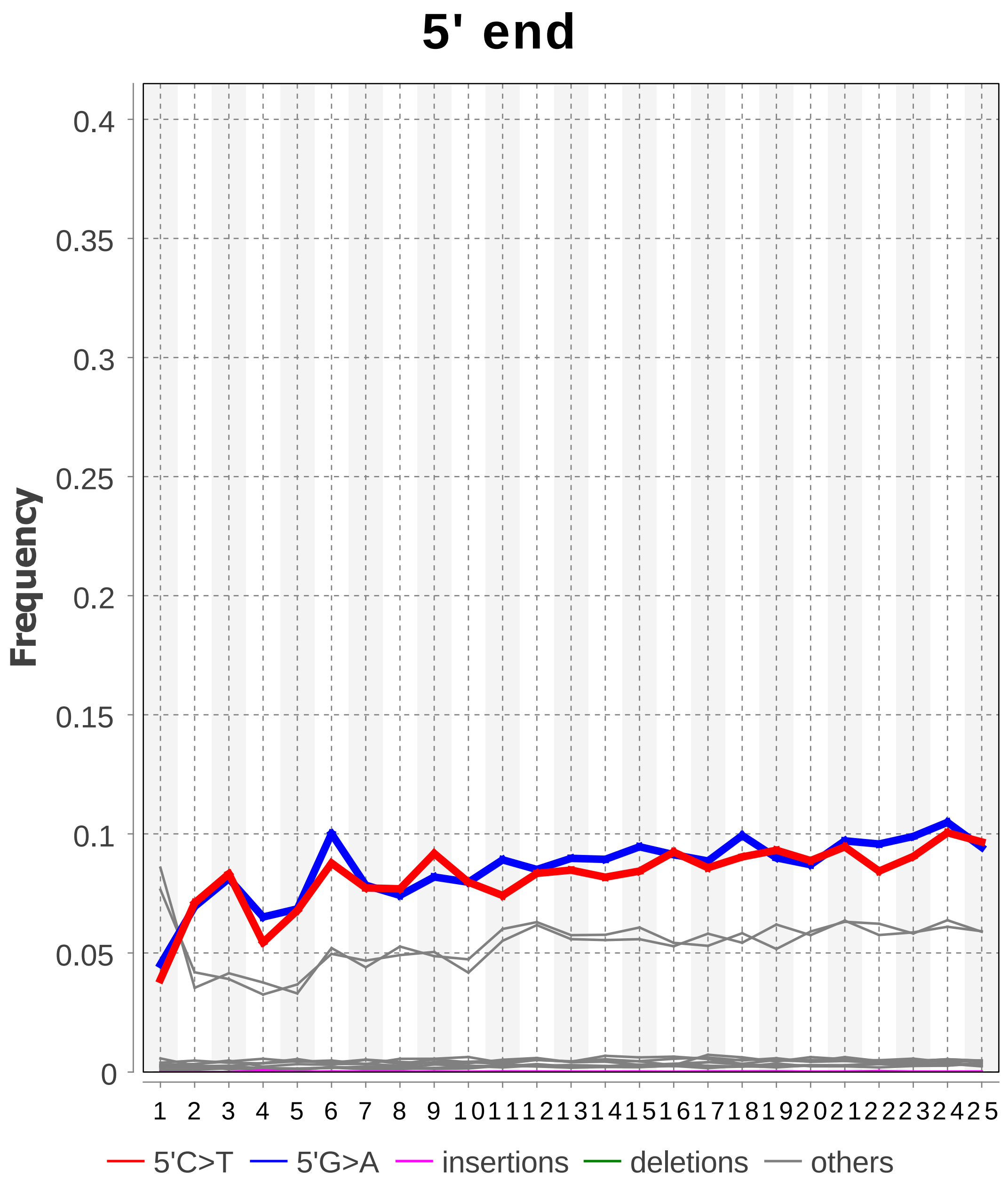

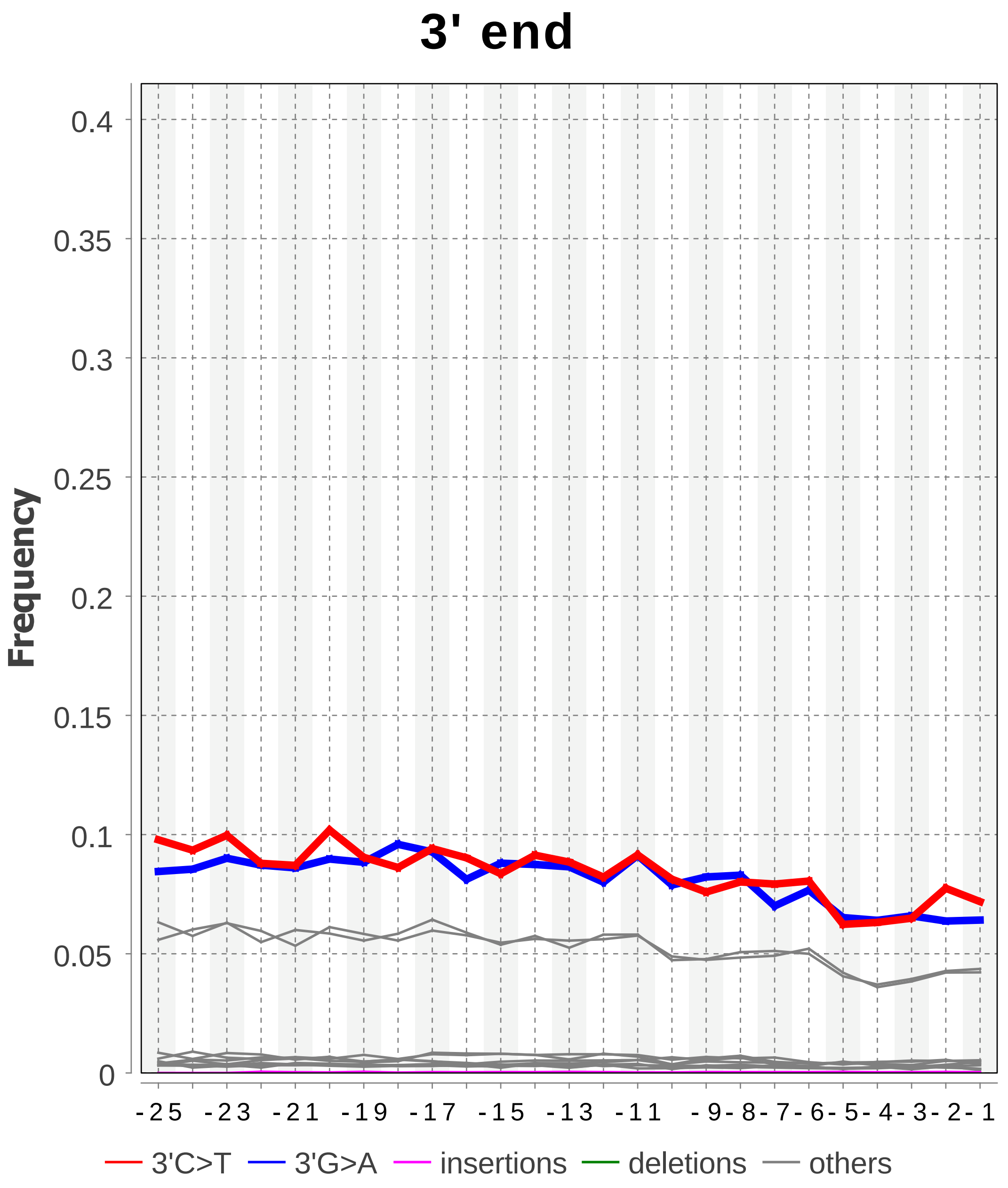

1652_aln
Number of used reads: 58,235 (100.0% of all input reads)

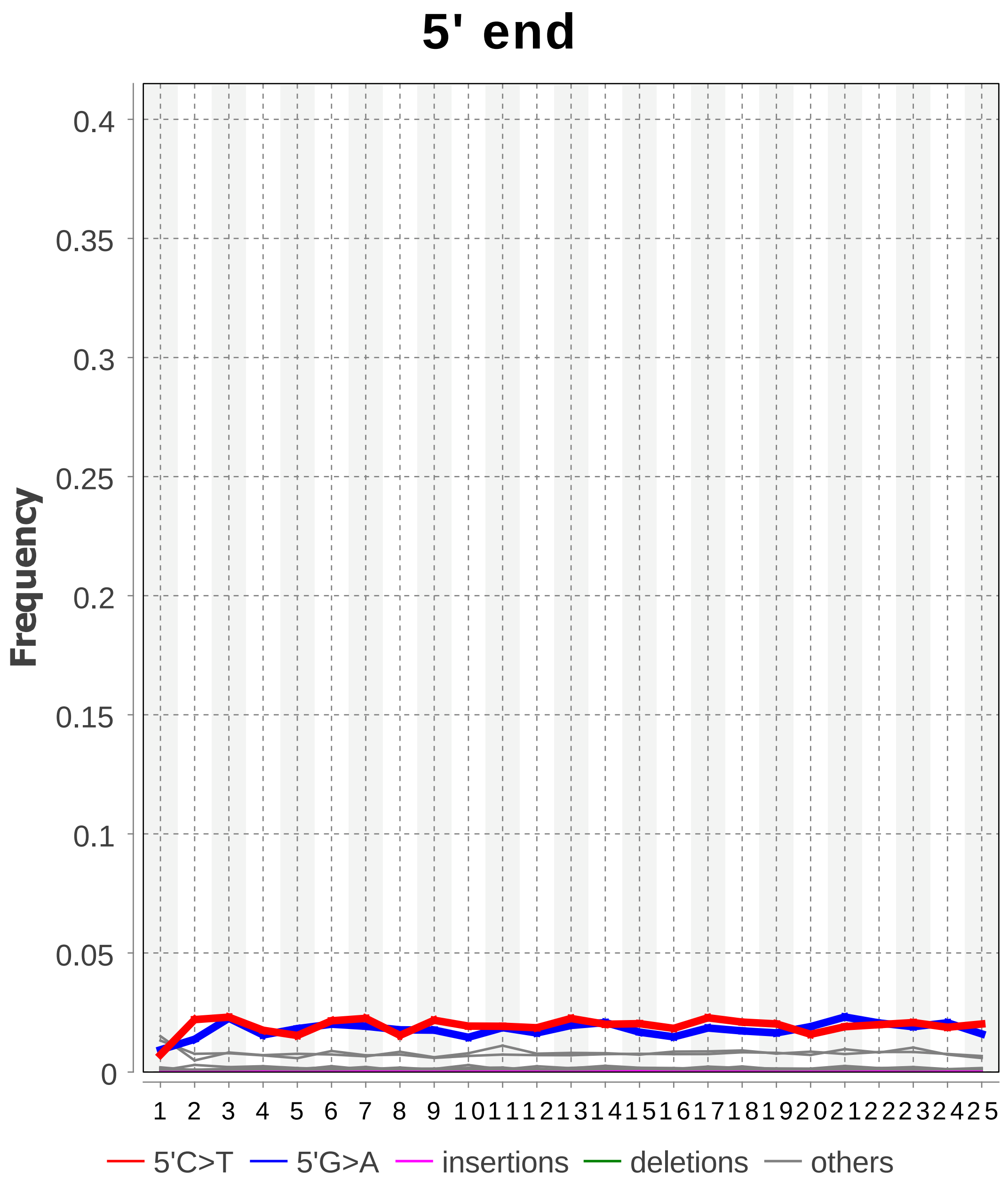

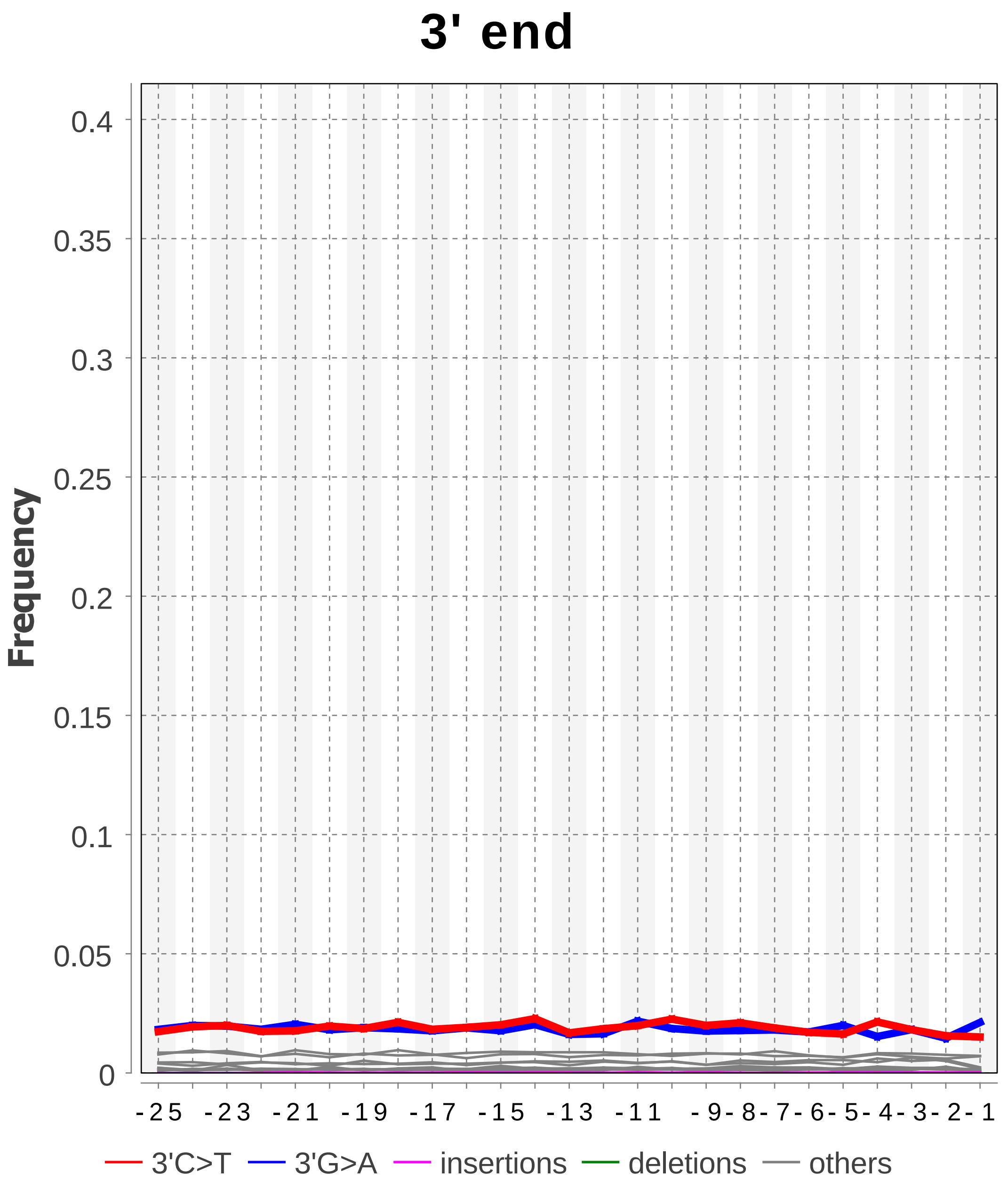

1652_MarkDuplicates

Number of used reads: 48,748 (100.0% of all input reads)

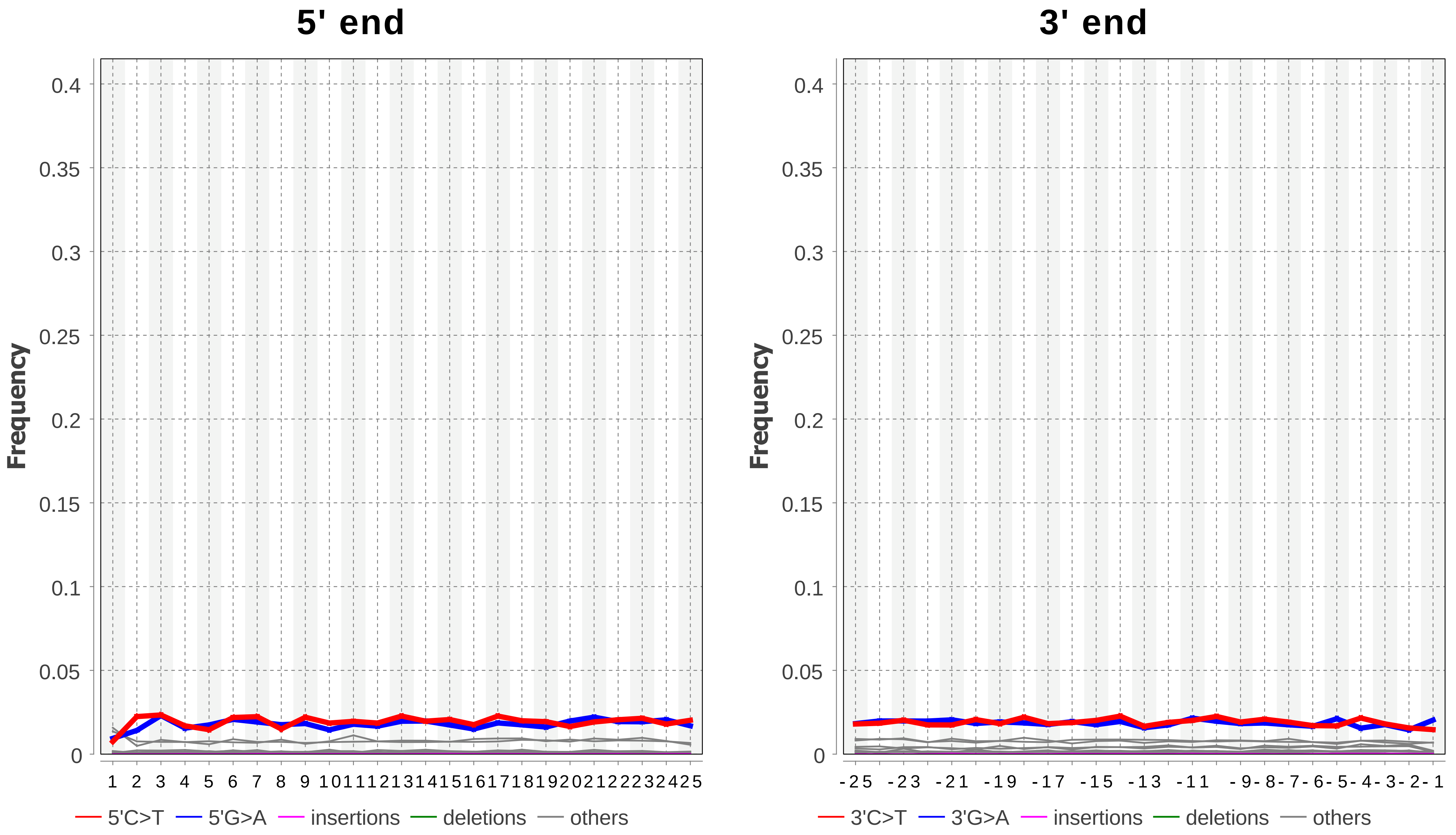

1653_aln
Number of used reads: 122,313 (100.0% of all input reads)

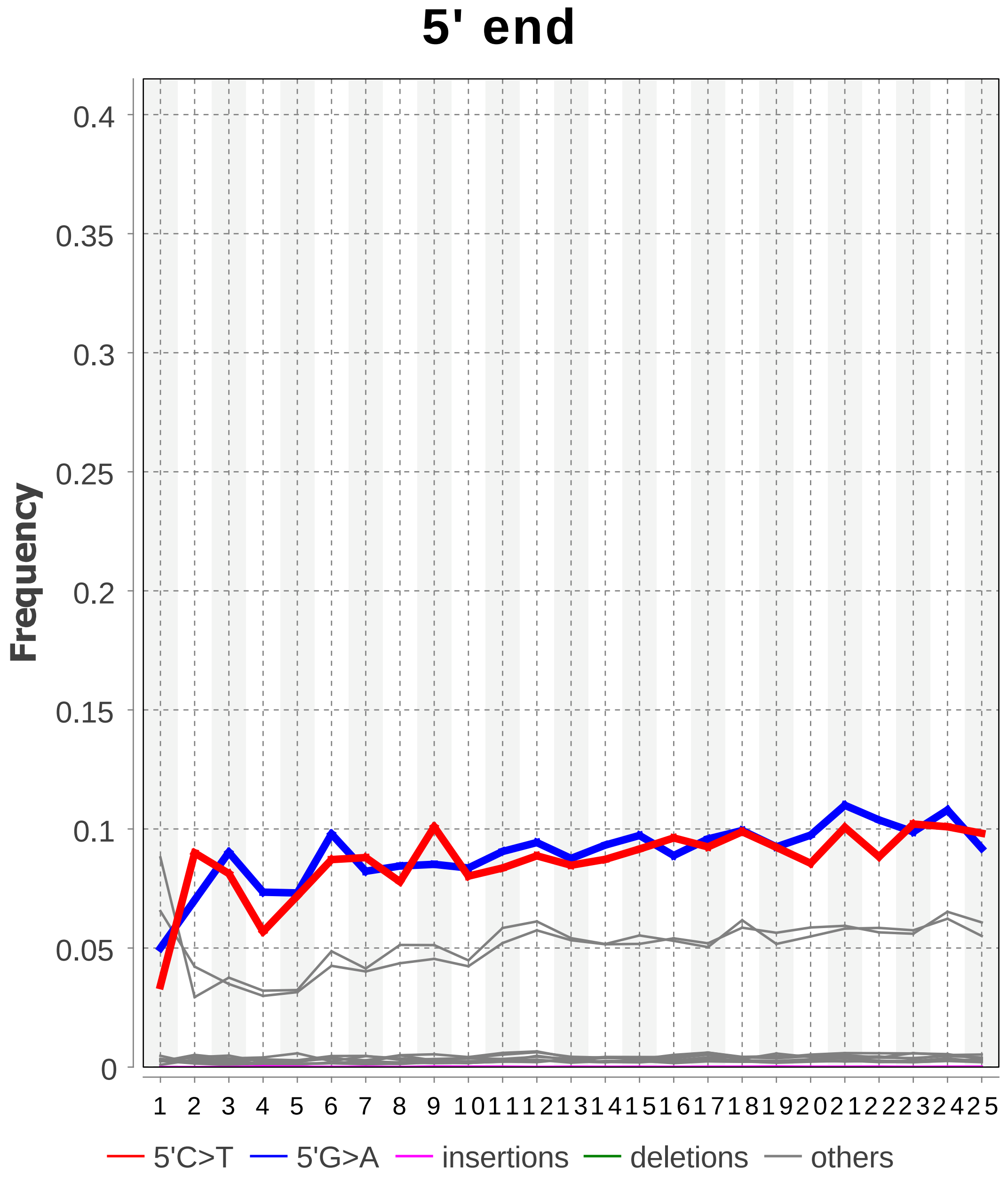

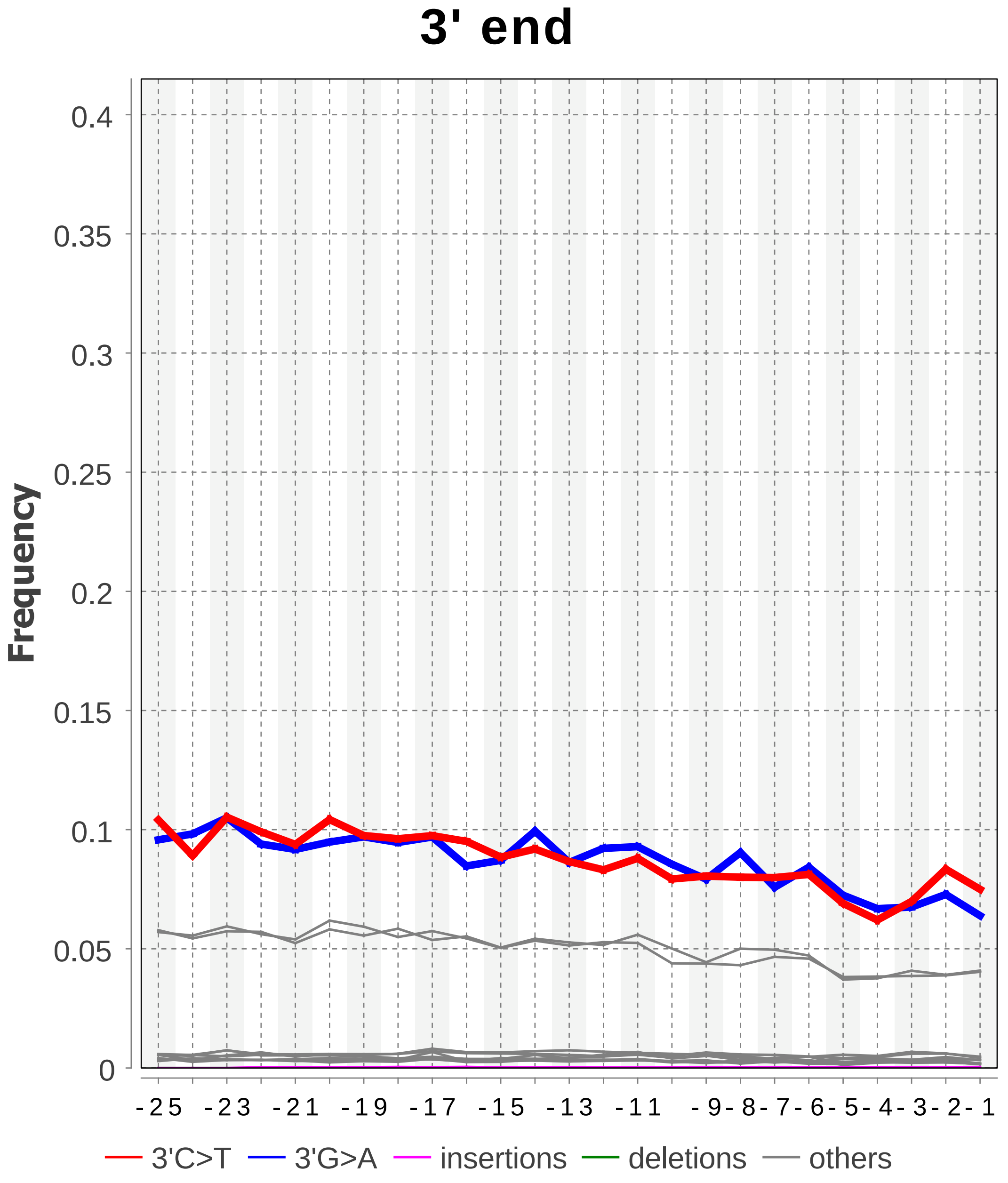

1653_MarkDuplicates
Number of used reads: 99,755 (100.0% of all input reads)

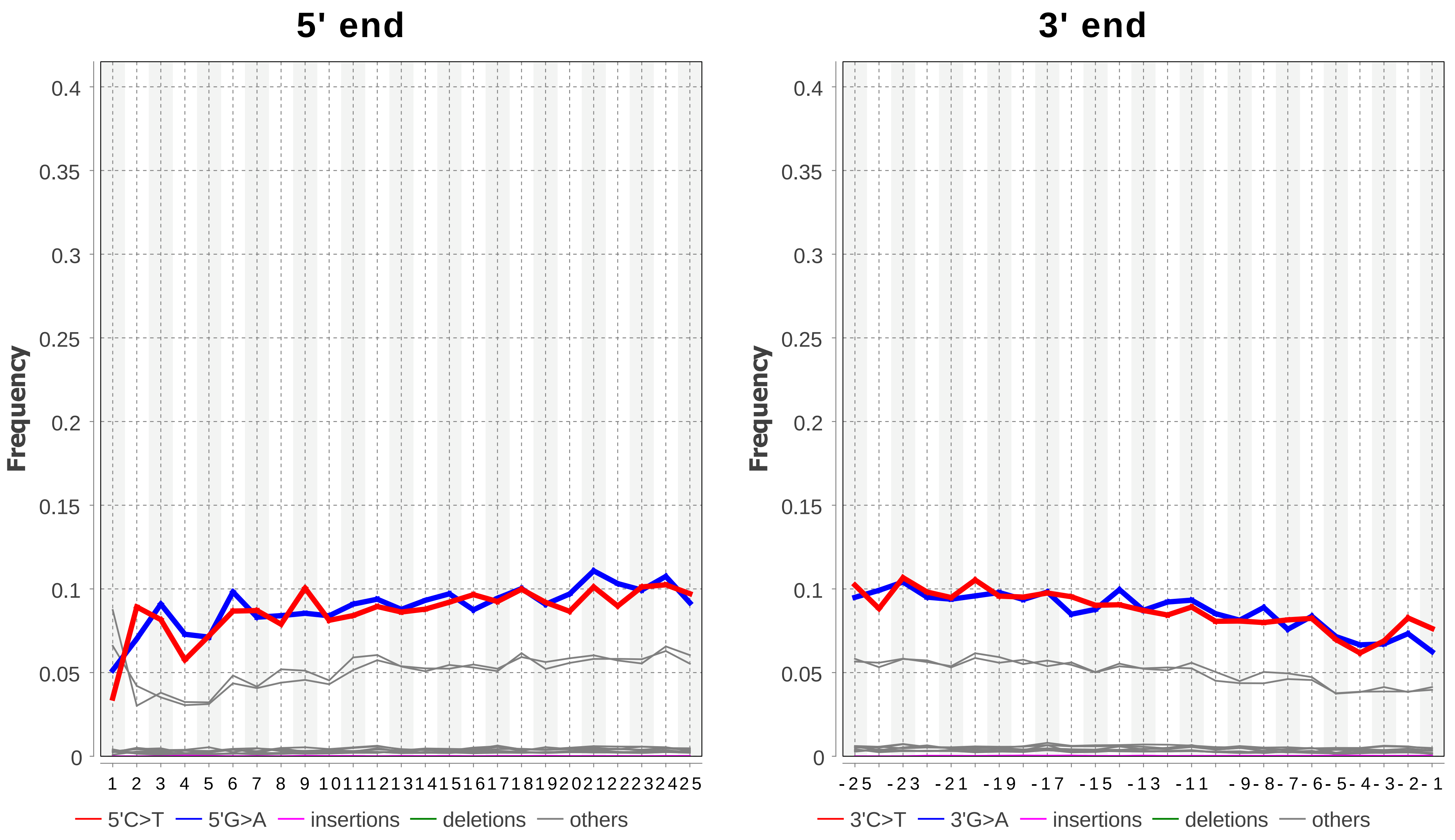

1656_aln

Number of used reads: 816,368 (100.0% of all input reads)

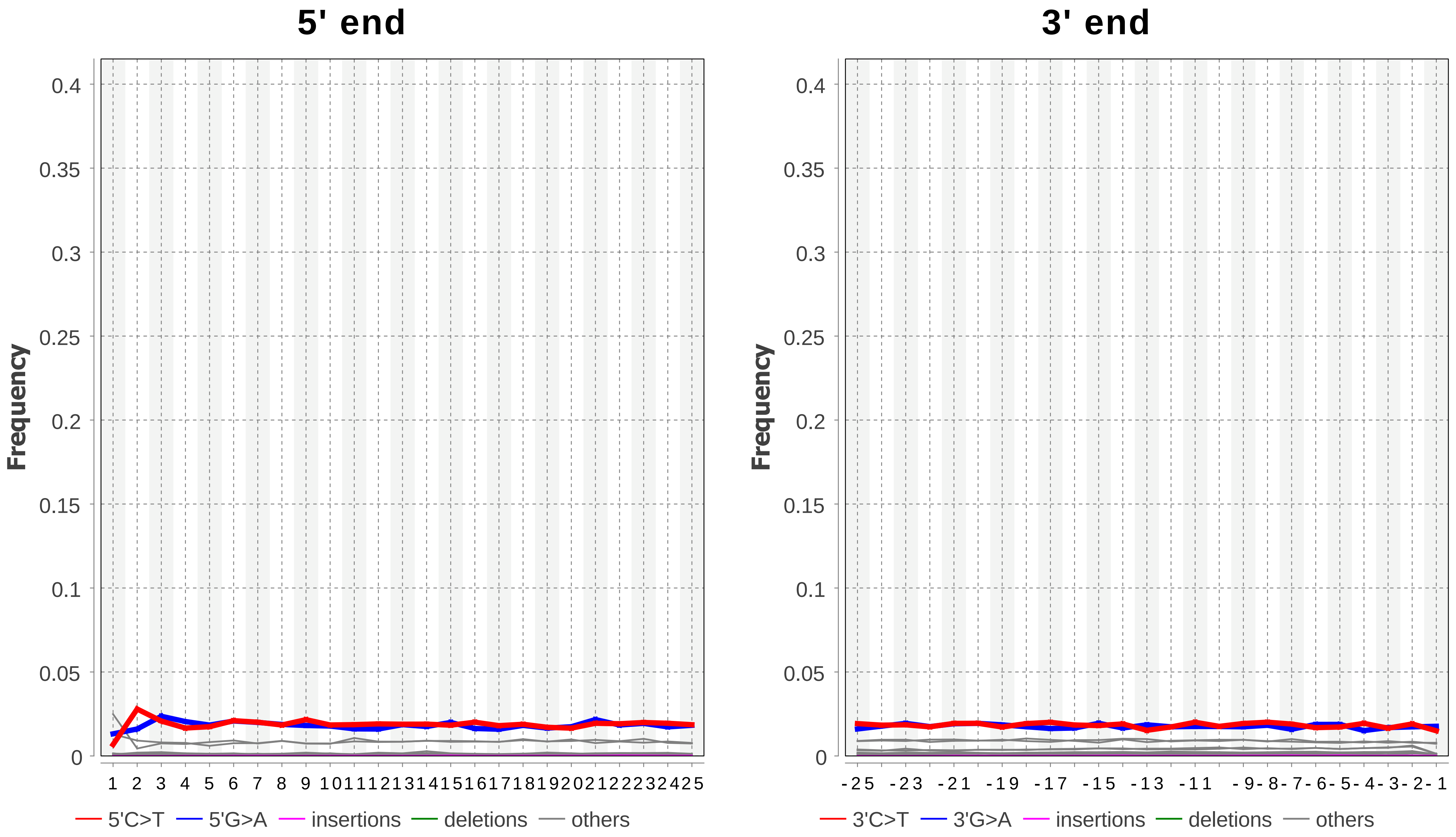

1656_MarkDuplicates
Number of used reads: 561,130 (100.0% of all input reads)

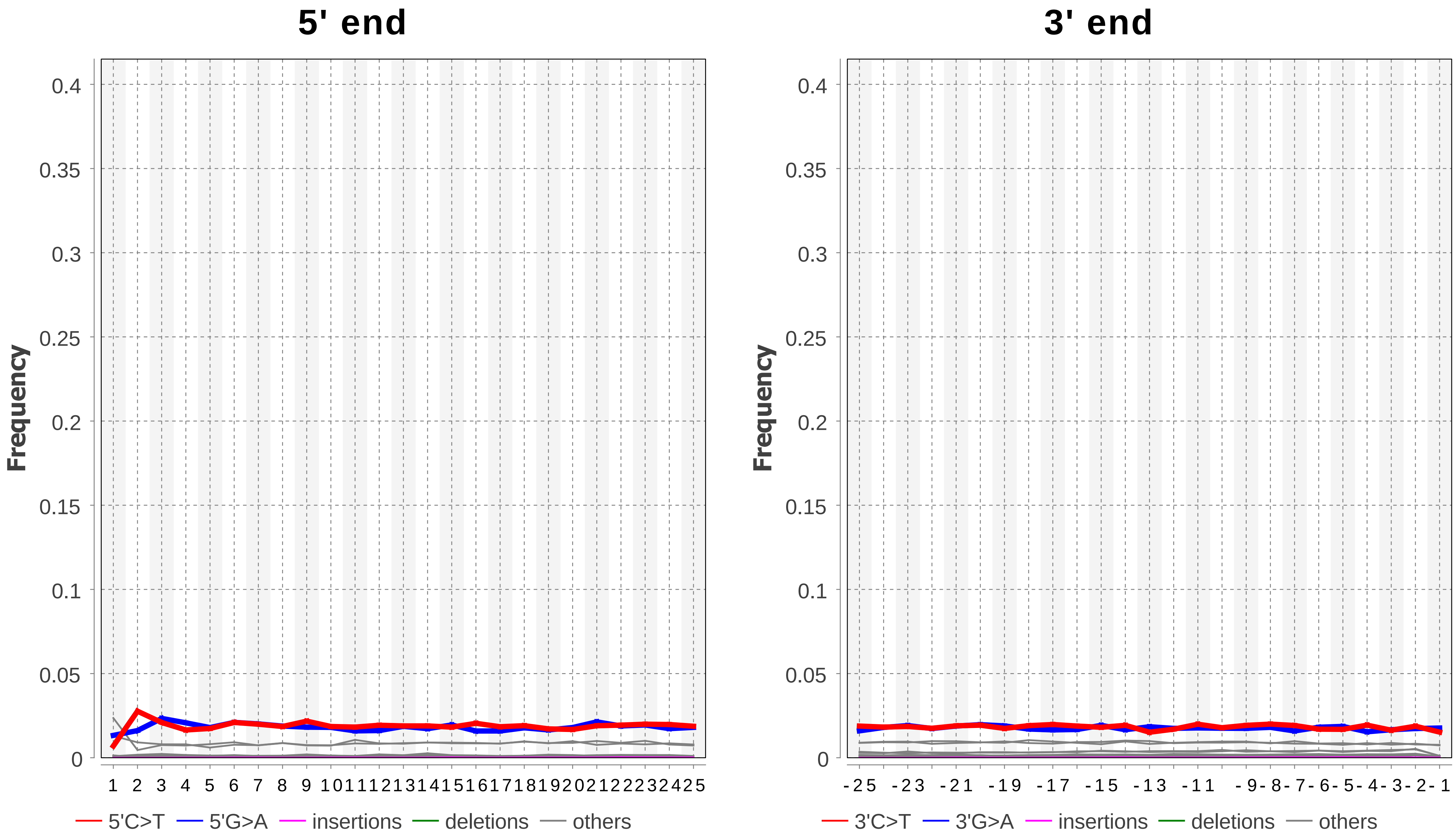

1661_aln
Number of used reads: 144,482 (100.0% of all input reads)

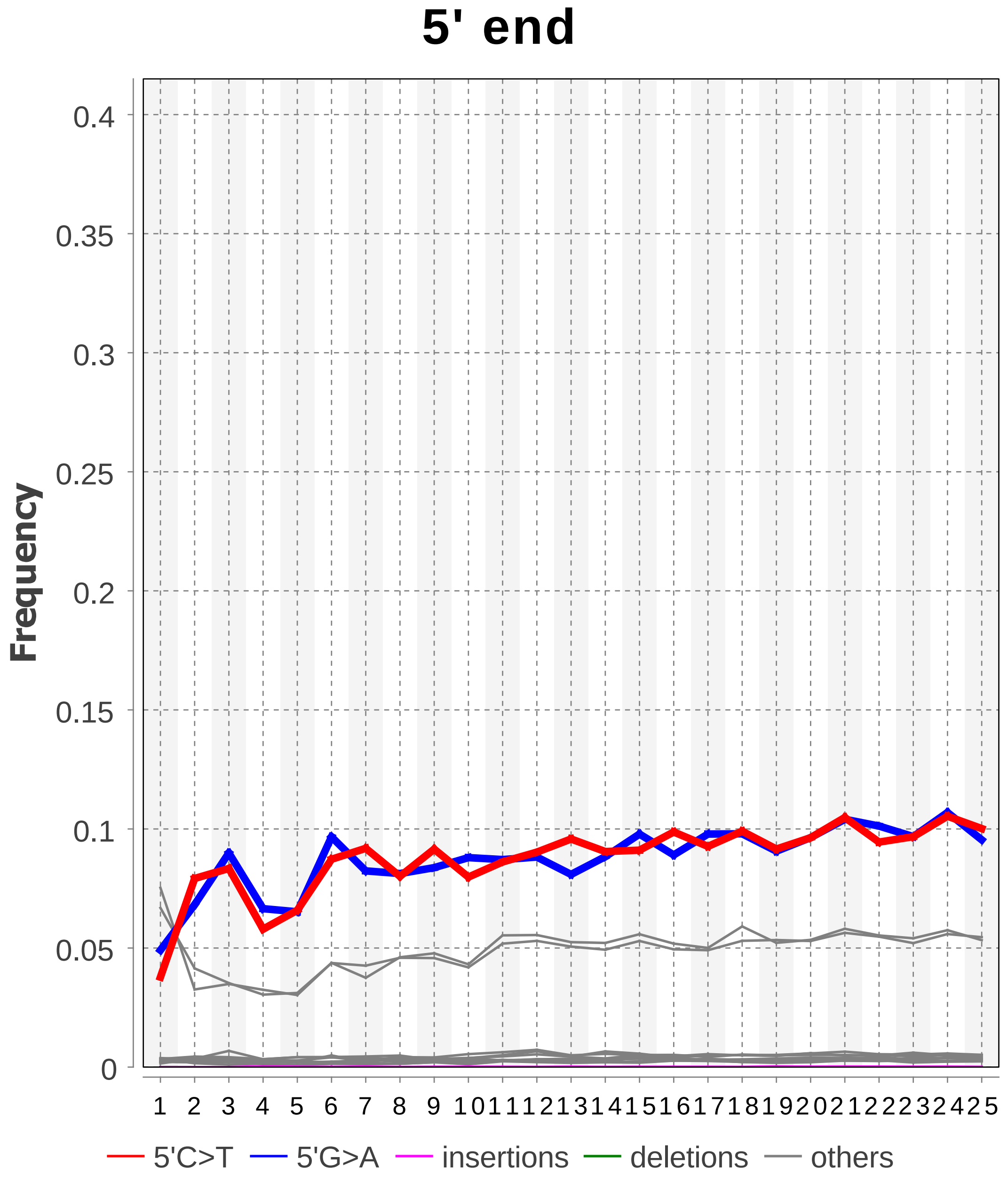

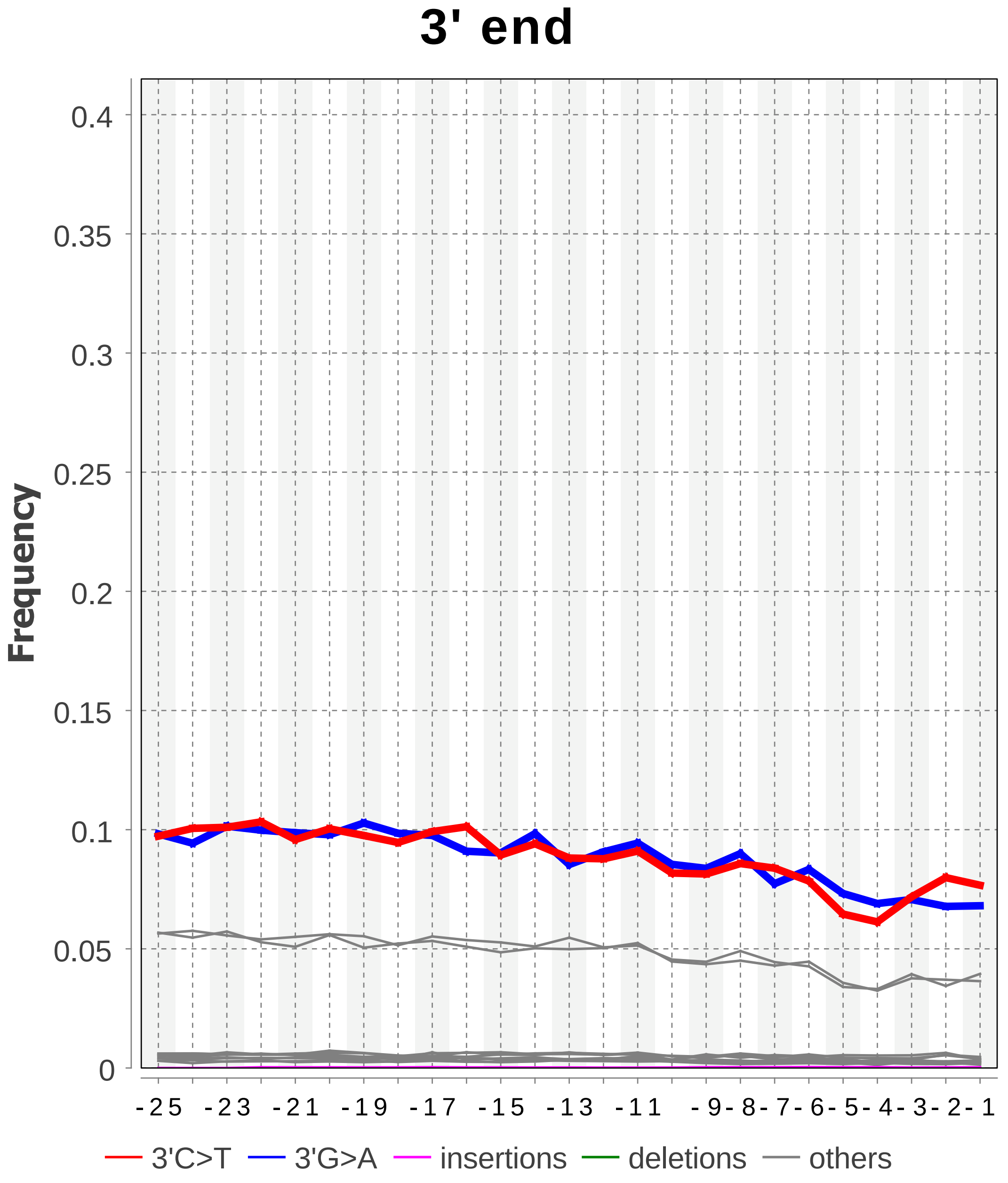

1661_MarkDuplicates

Number of used reads: 116,116 (100.0% of all input reads)

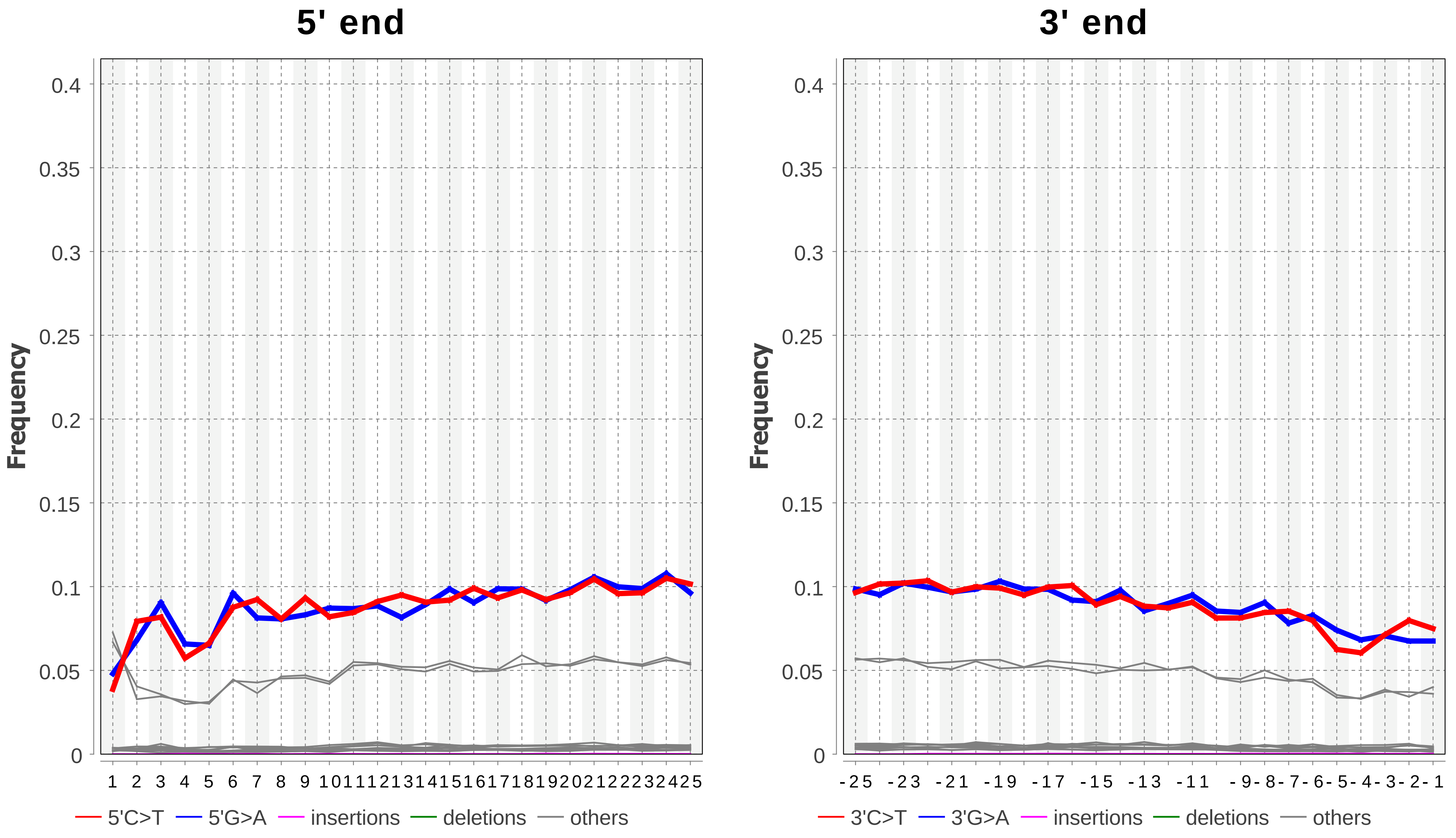

1662_aln

Number of used reads: 69,621 (100.0% of all input reads)

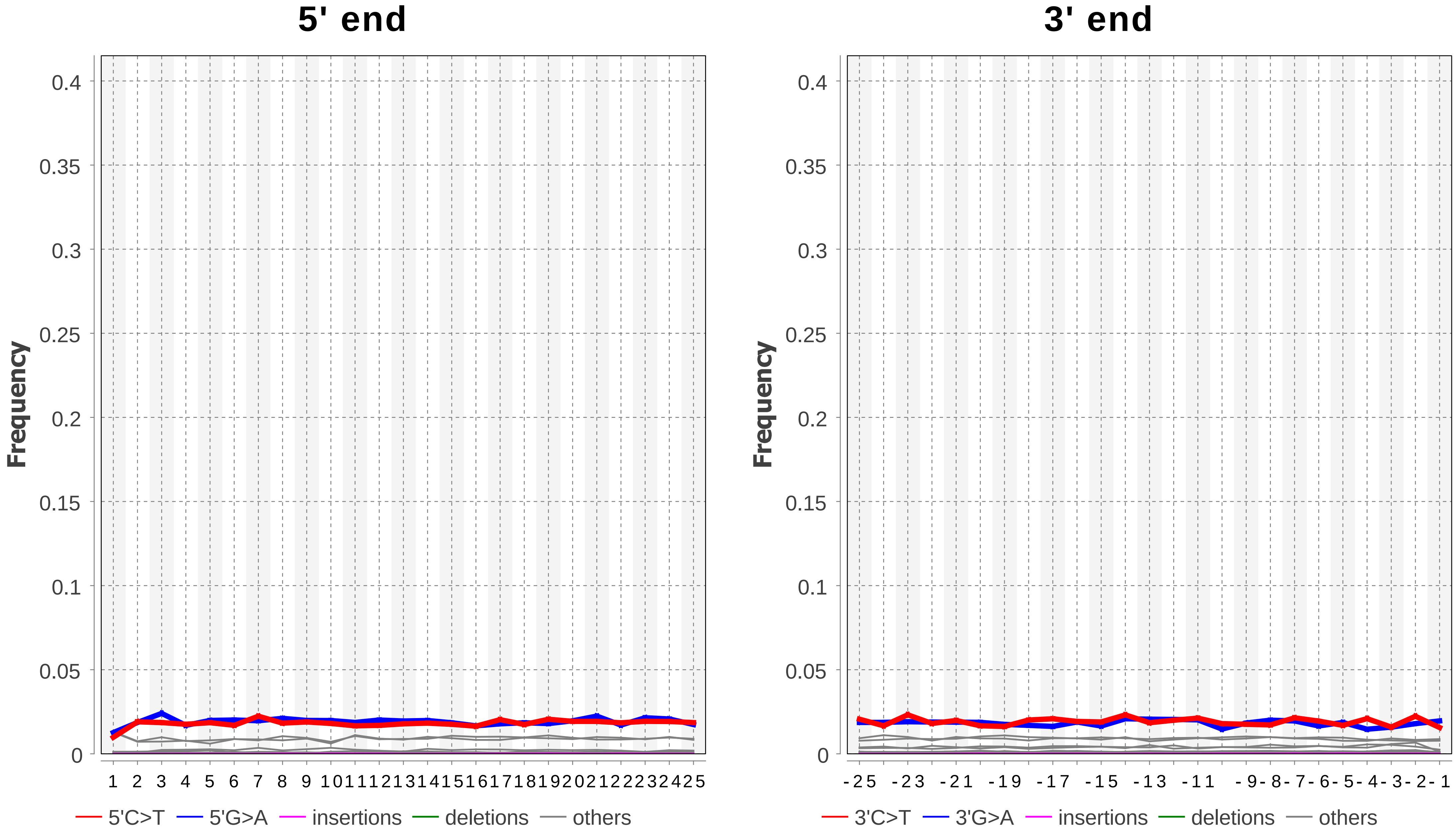

1662_MarkDuplicates
Number of used reads: 57,594 (100.0% of all input reads)

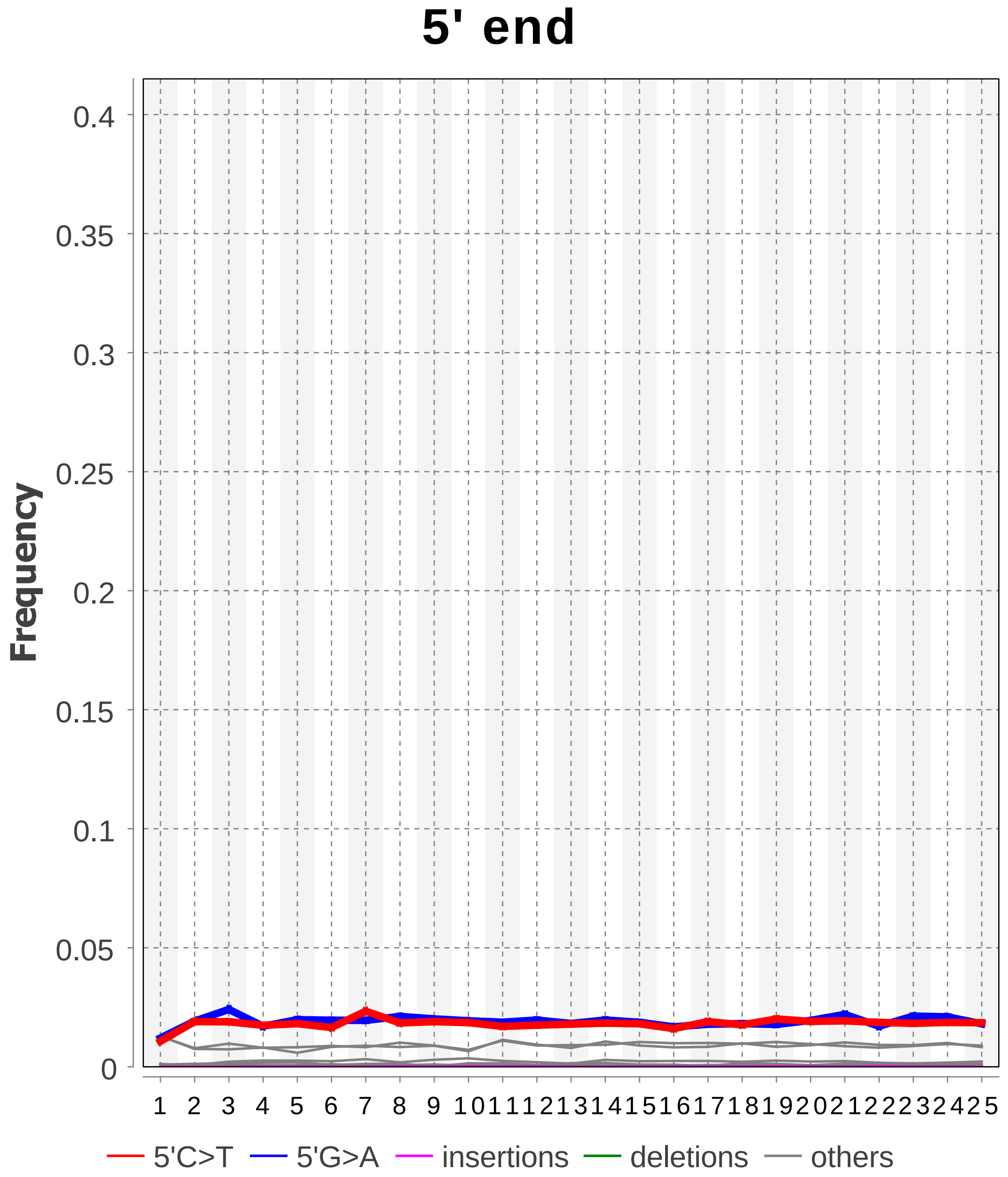

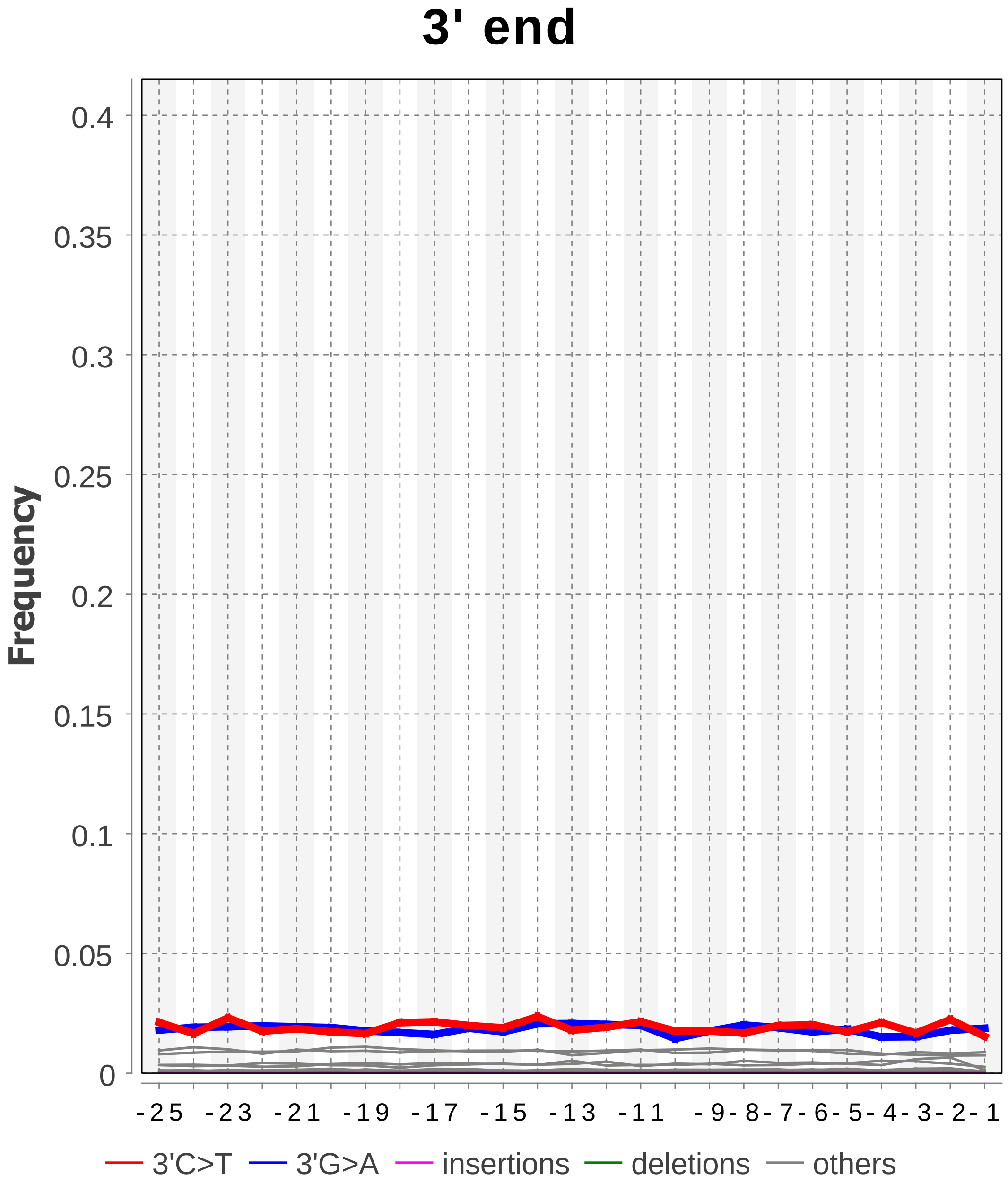

1746_aln
Number of used reads: 76,248 (100.0% of all input reads)

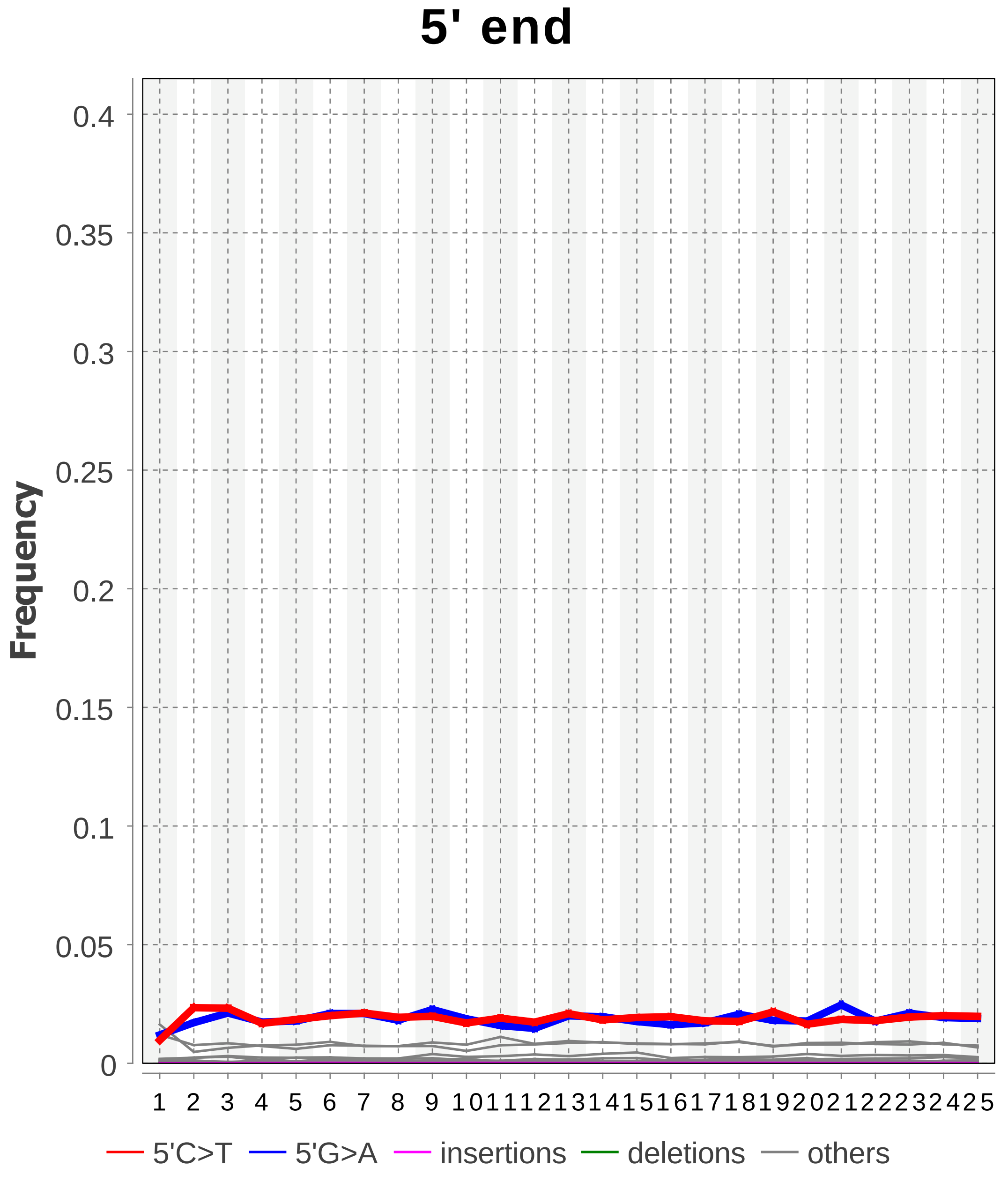

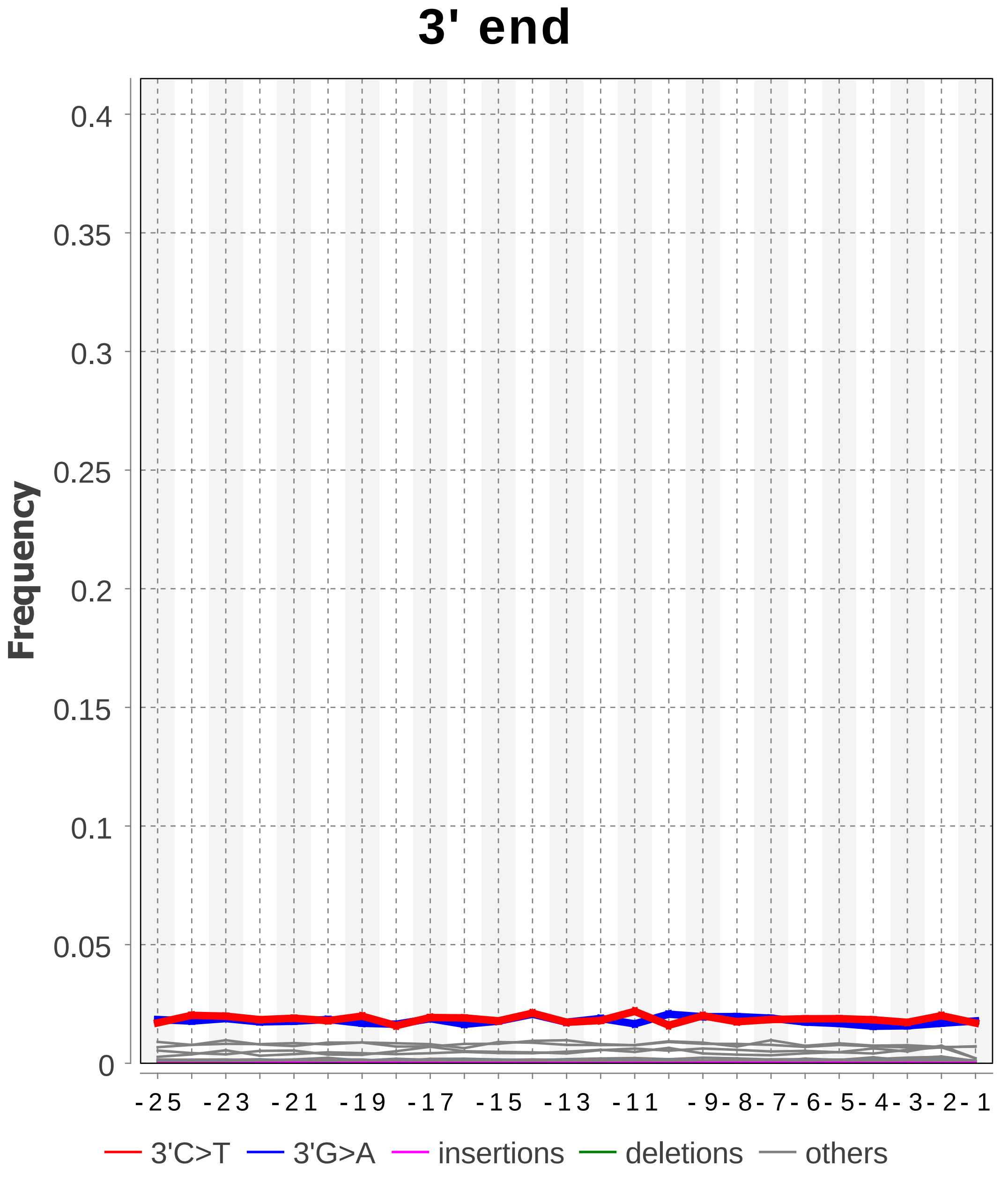

1746_MarkDuplicates
Number of used reads: 61,881 (100.0% of all input reads)

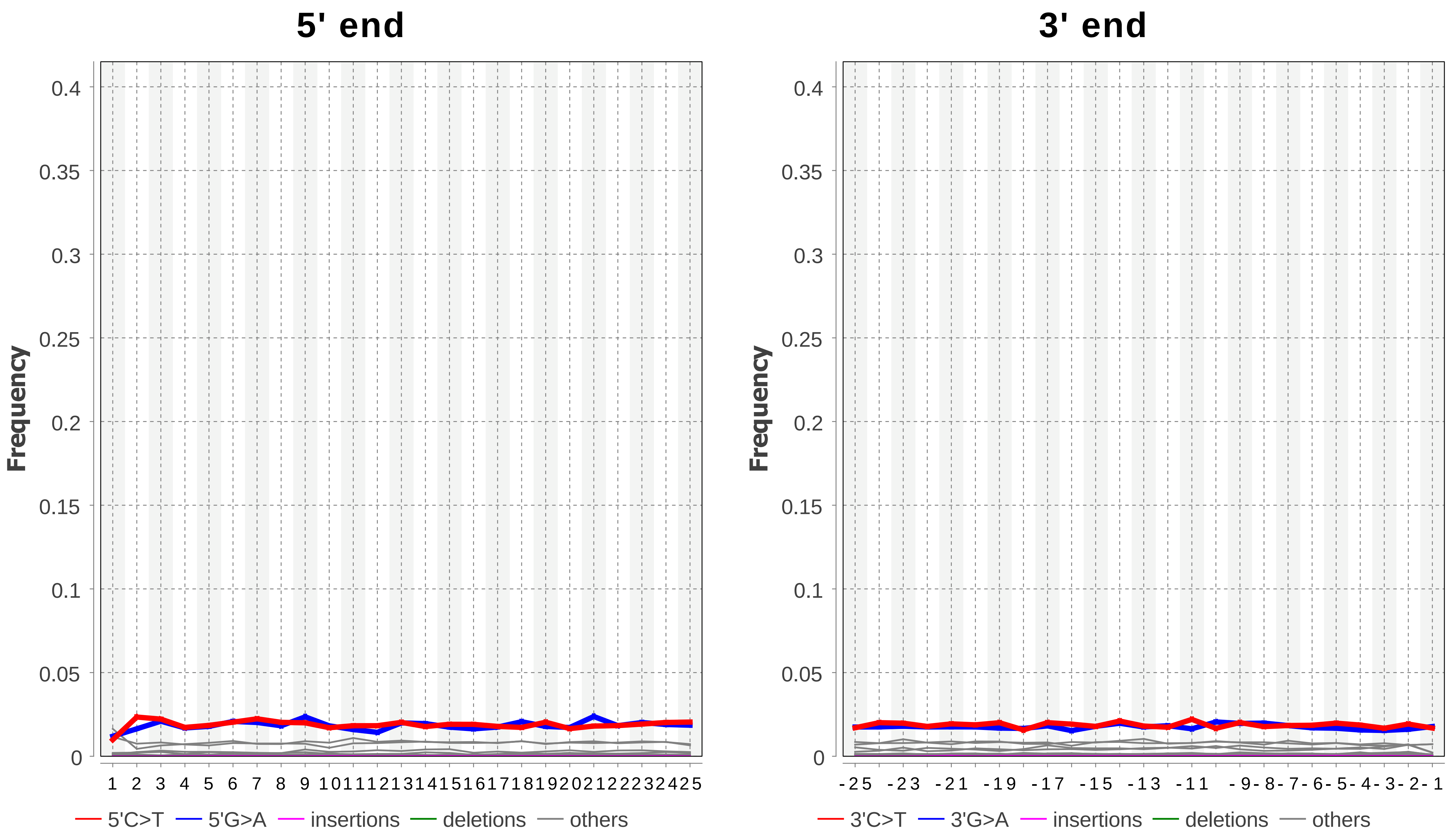

1968_aln

Number of used reads: 55,207 (100.0% of all input reads)

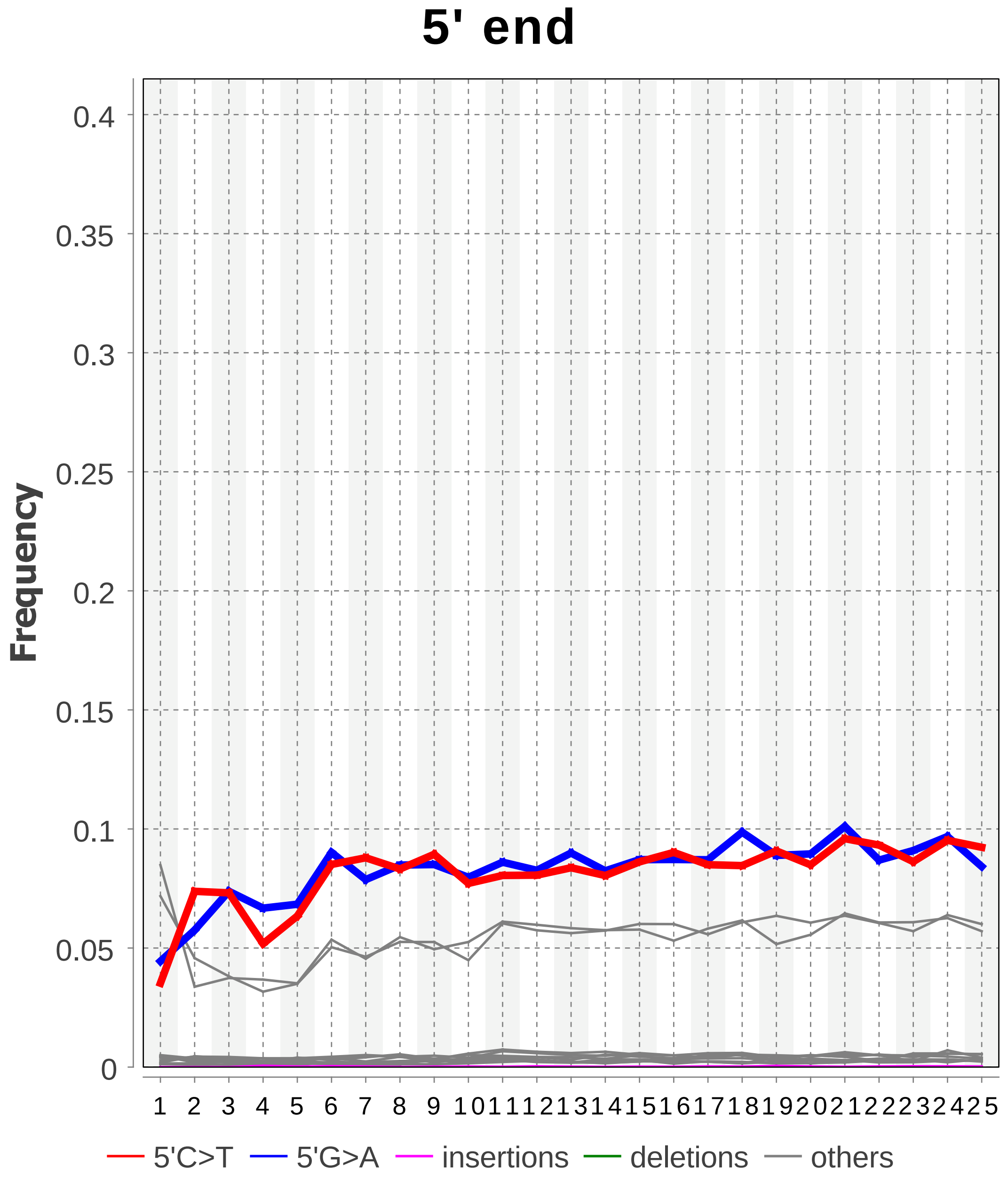

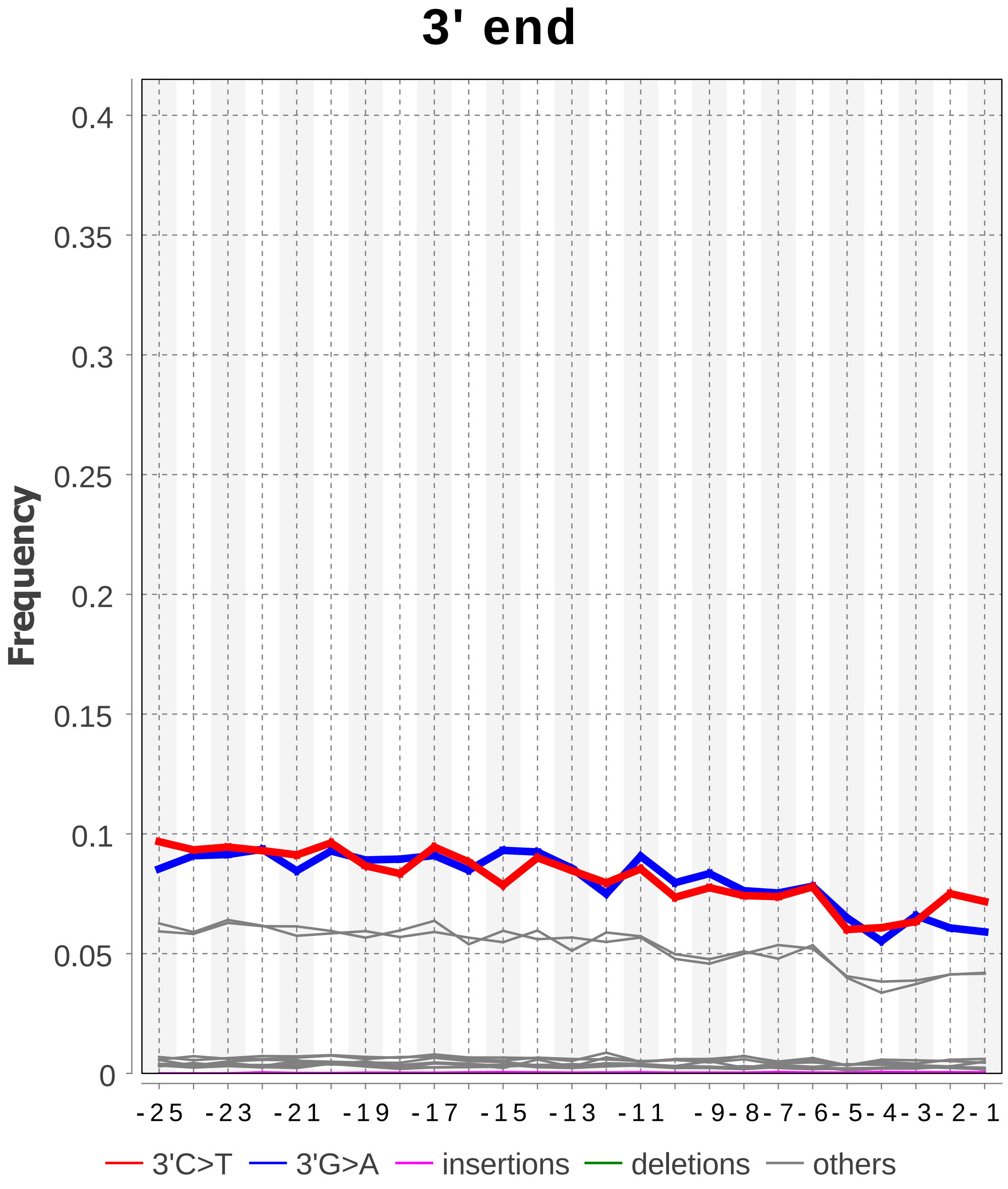

1968_MarkDuplicates

Number of used reads: 46,473 (100.0% of all input reads)

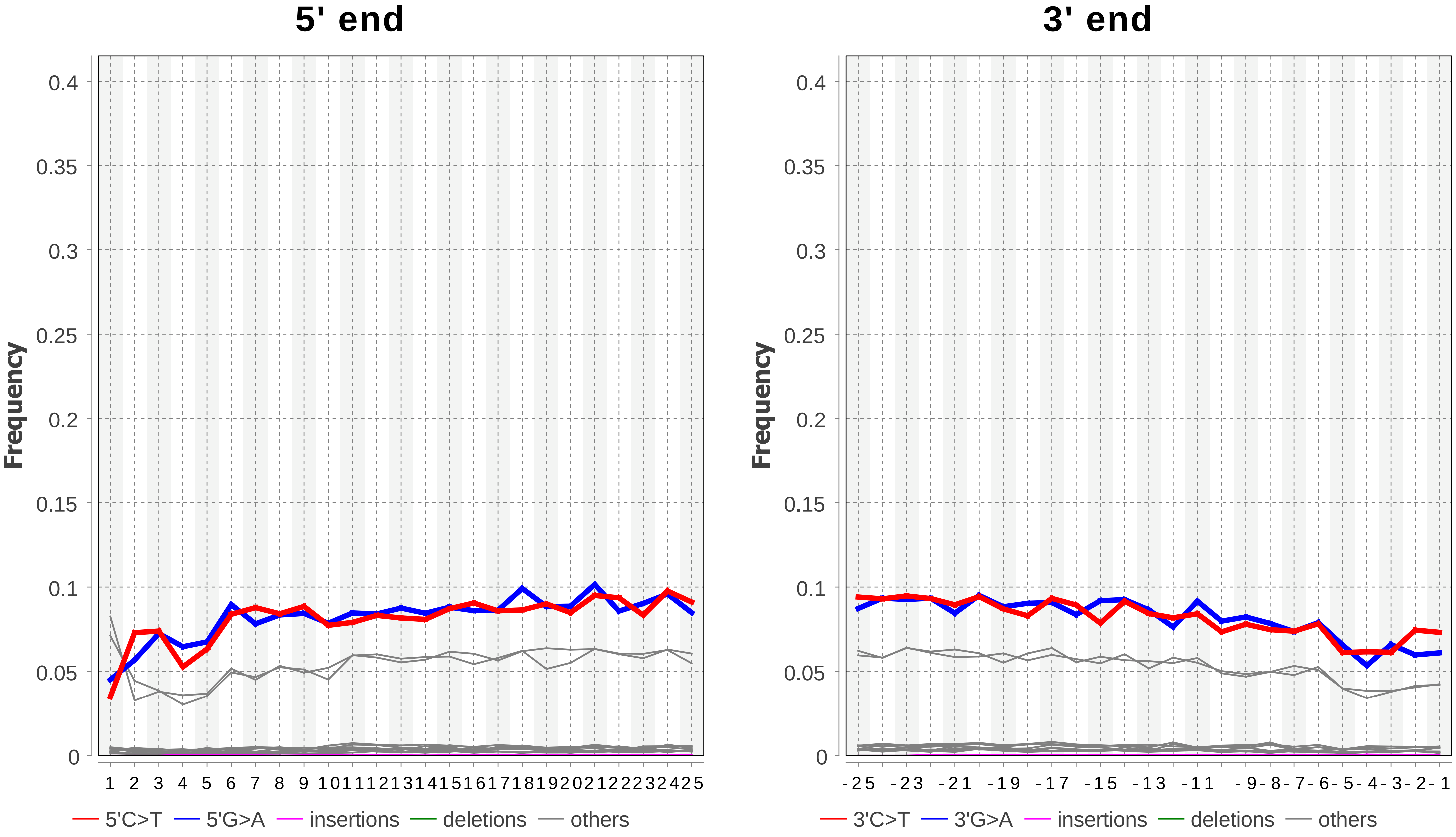

1971_aln

Number of used reads: 79,097 (100.0% of all input reads)

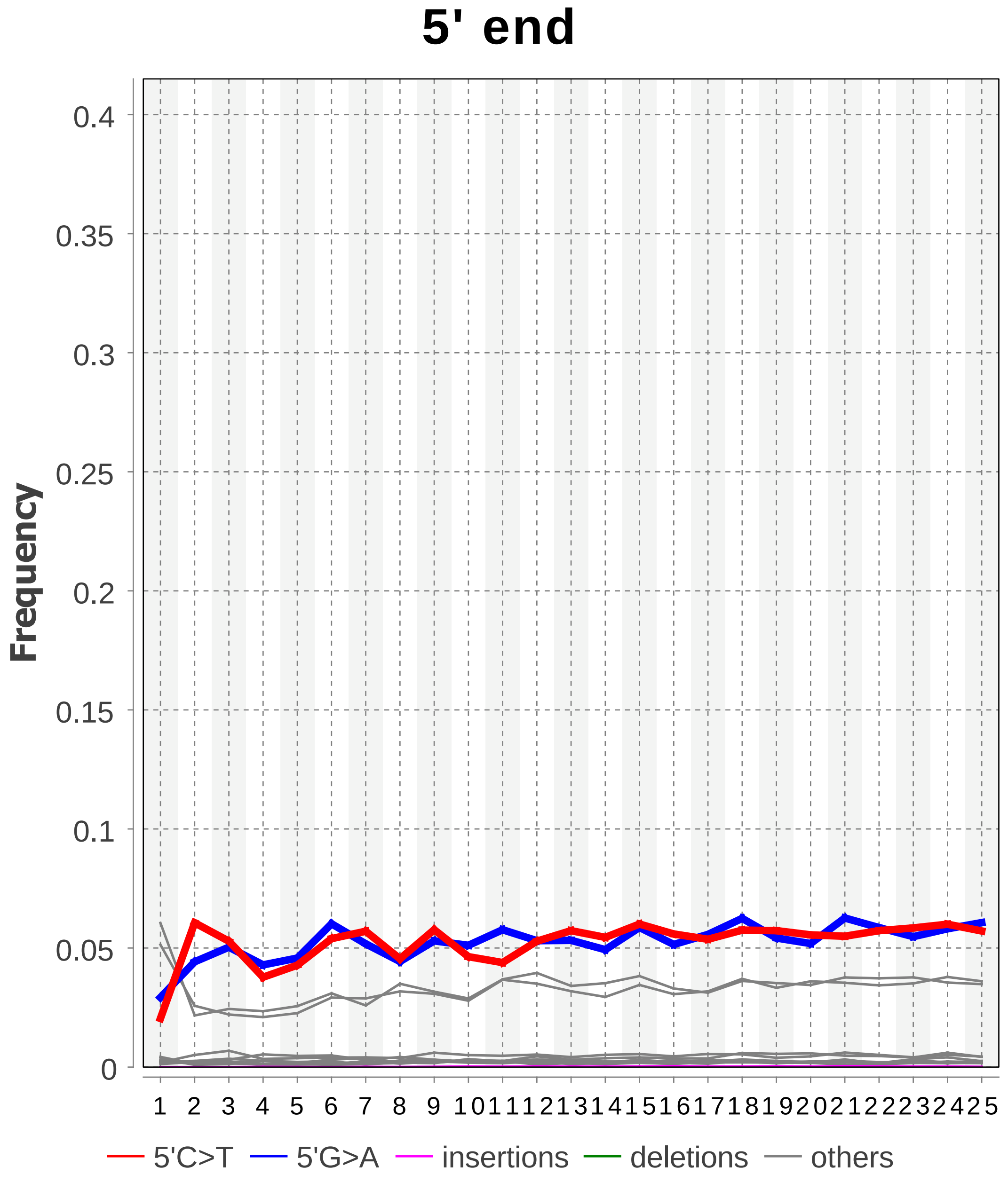

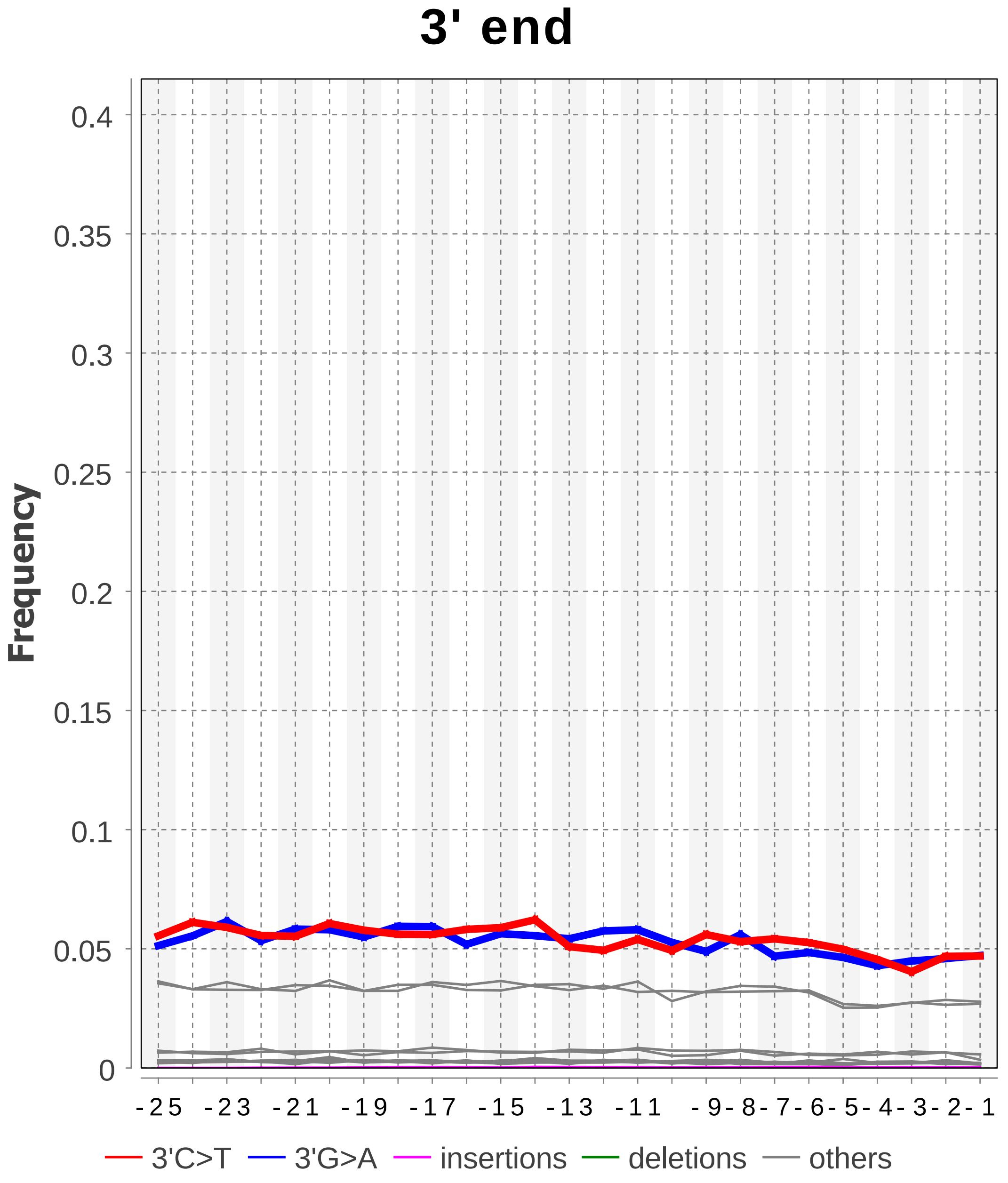

1971_MarkDuplicates

Number of used reads: 66,934 (100.0% of all input reads)

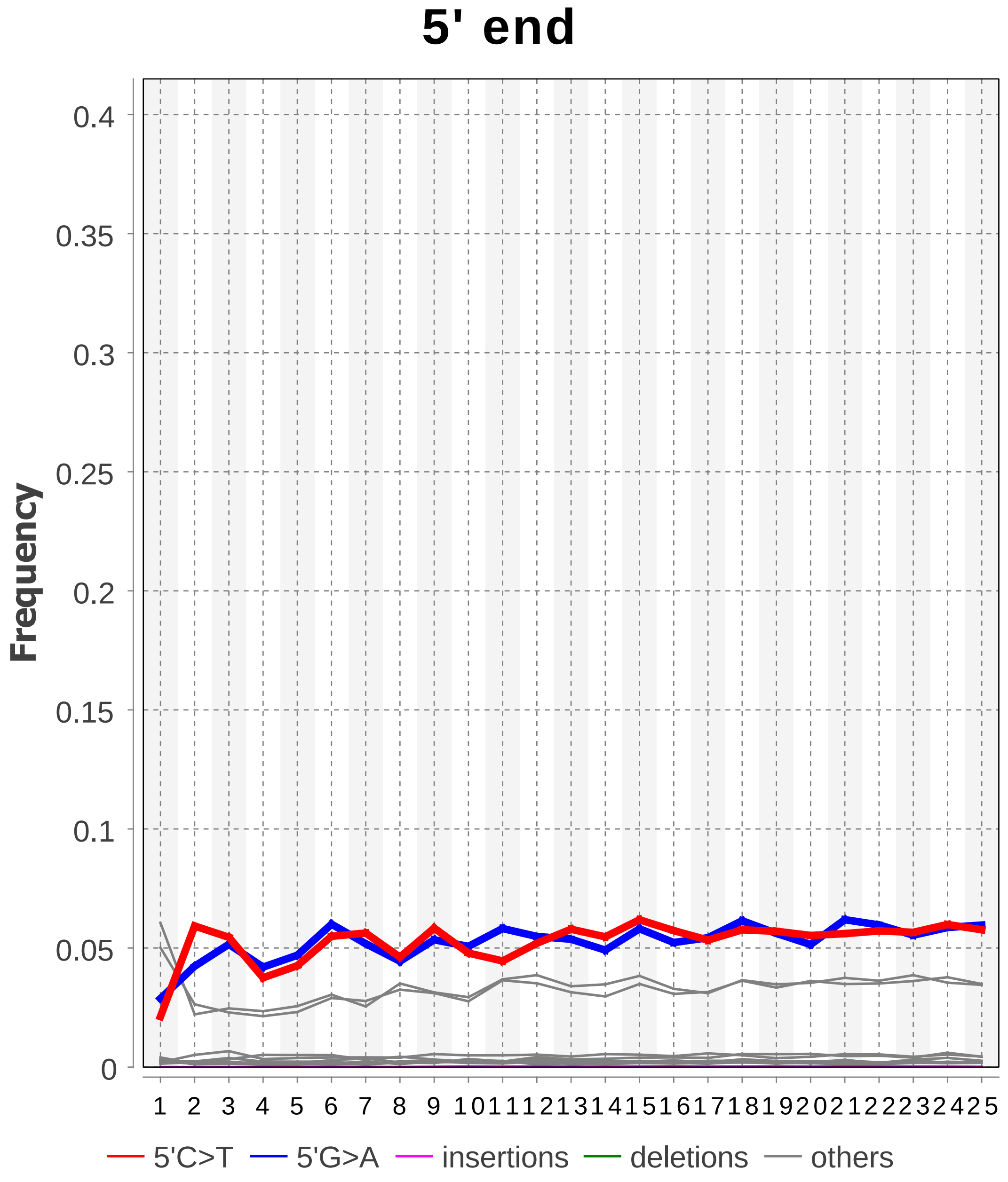

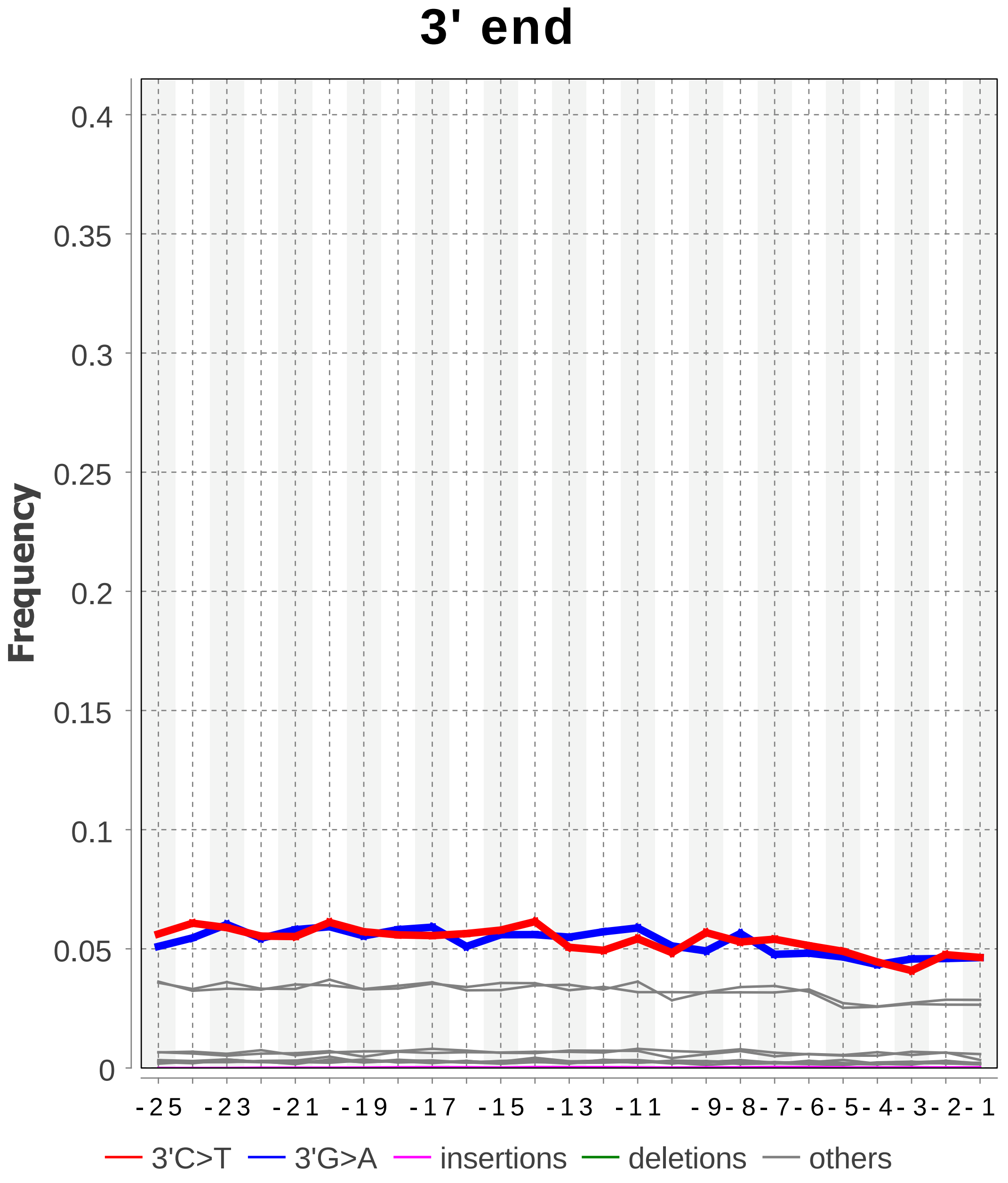

1972_aln

Number of used reads: 64,808 (100.0% of all input reads)

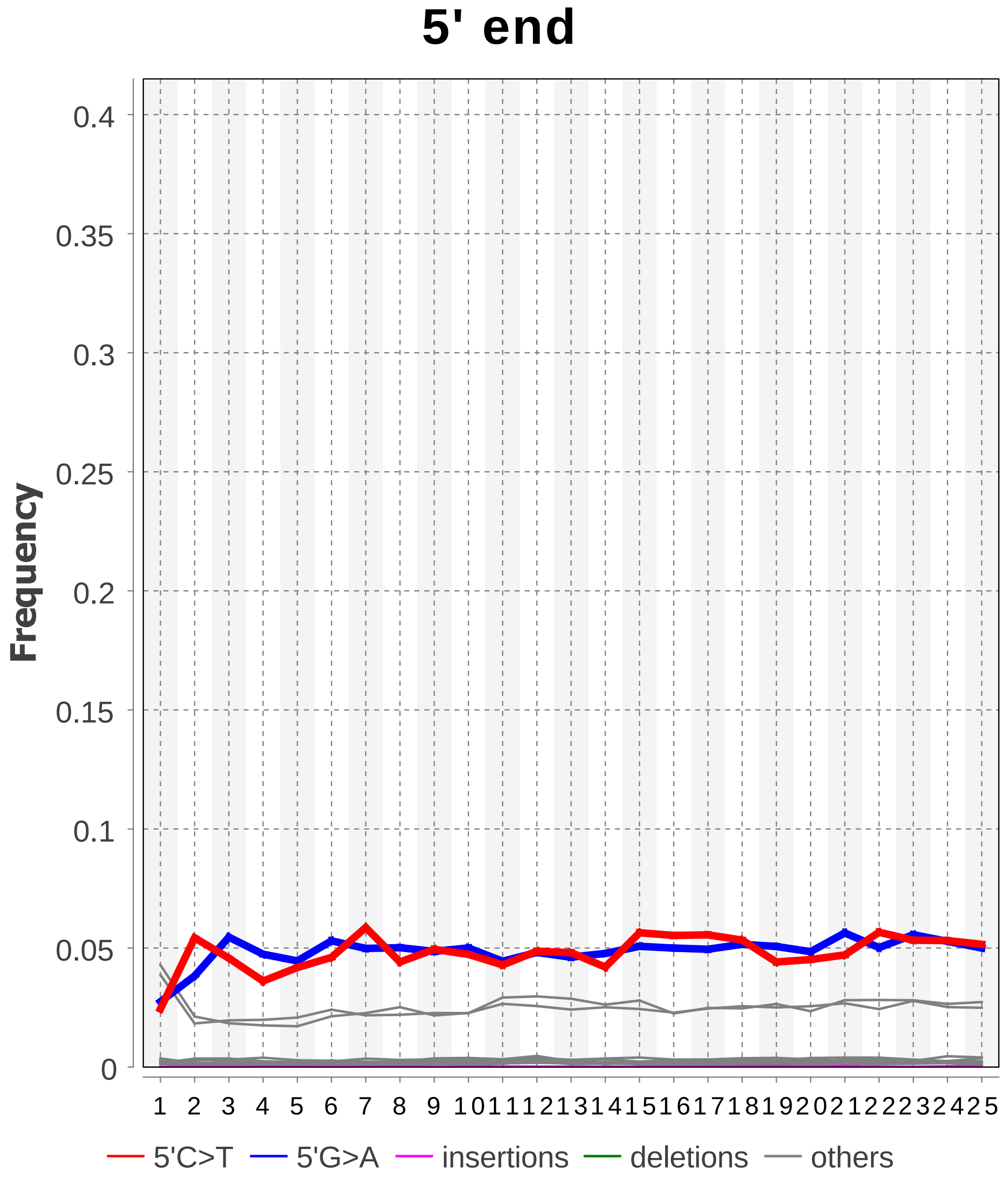

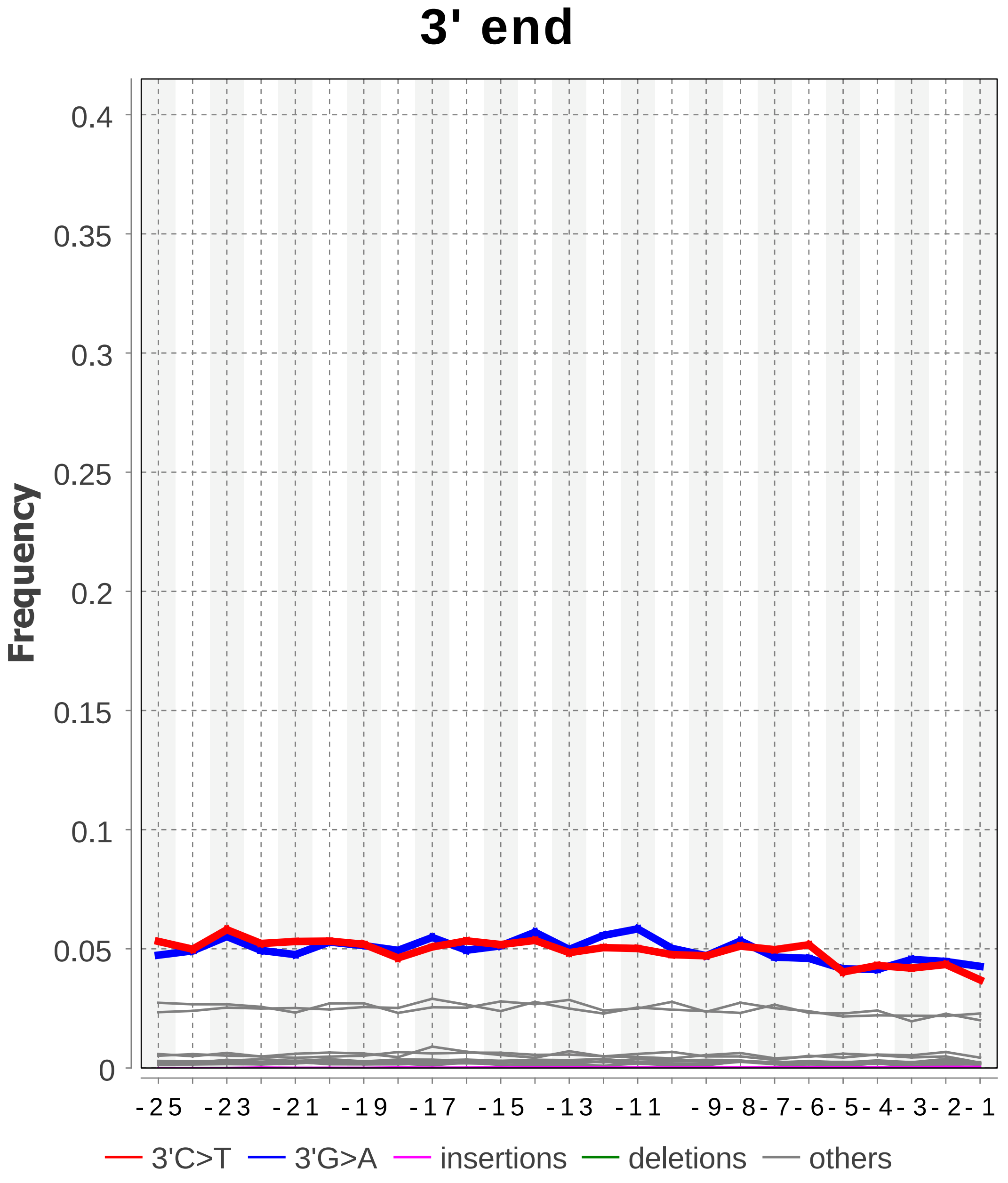

1972_MarkDuplicates
Number of used reads: 56,794 (100.0% of all input reads)

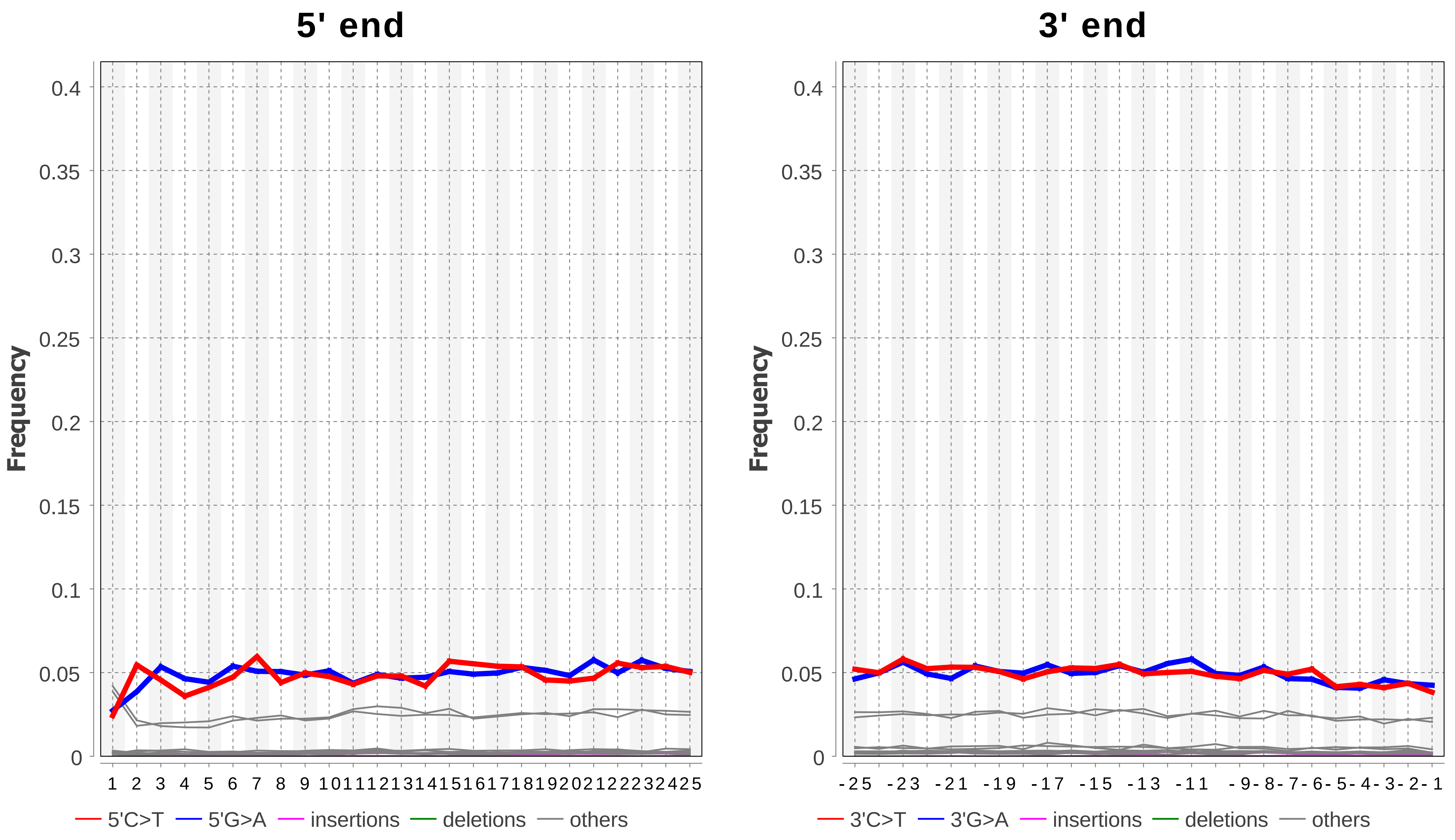

1996_aln
Number of used reads: 151,584 (100.0% of all input reads)

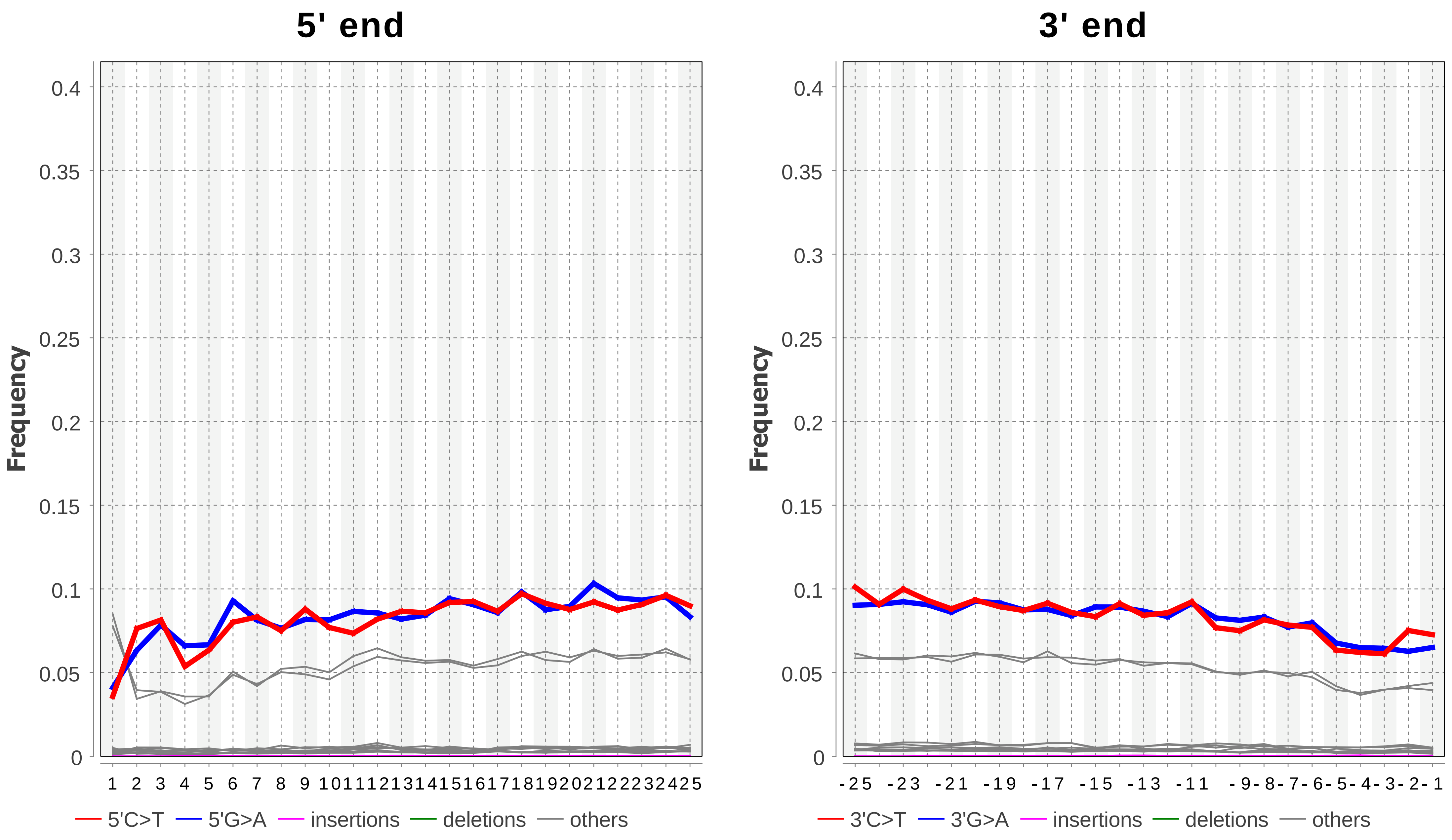

1996_MarkDuplicates
Number of used reads: 121,471 (100.0% of all input reads)

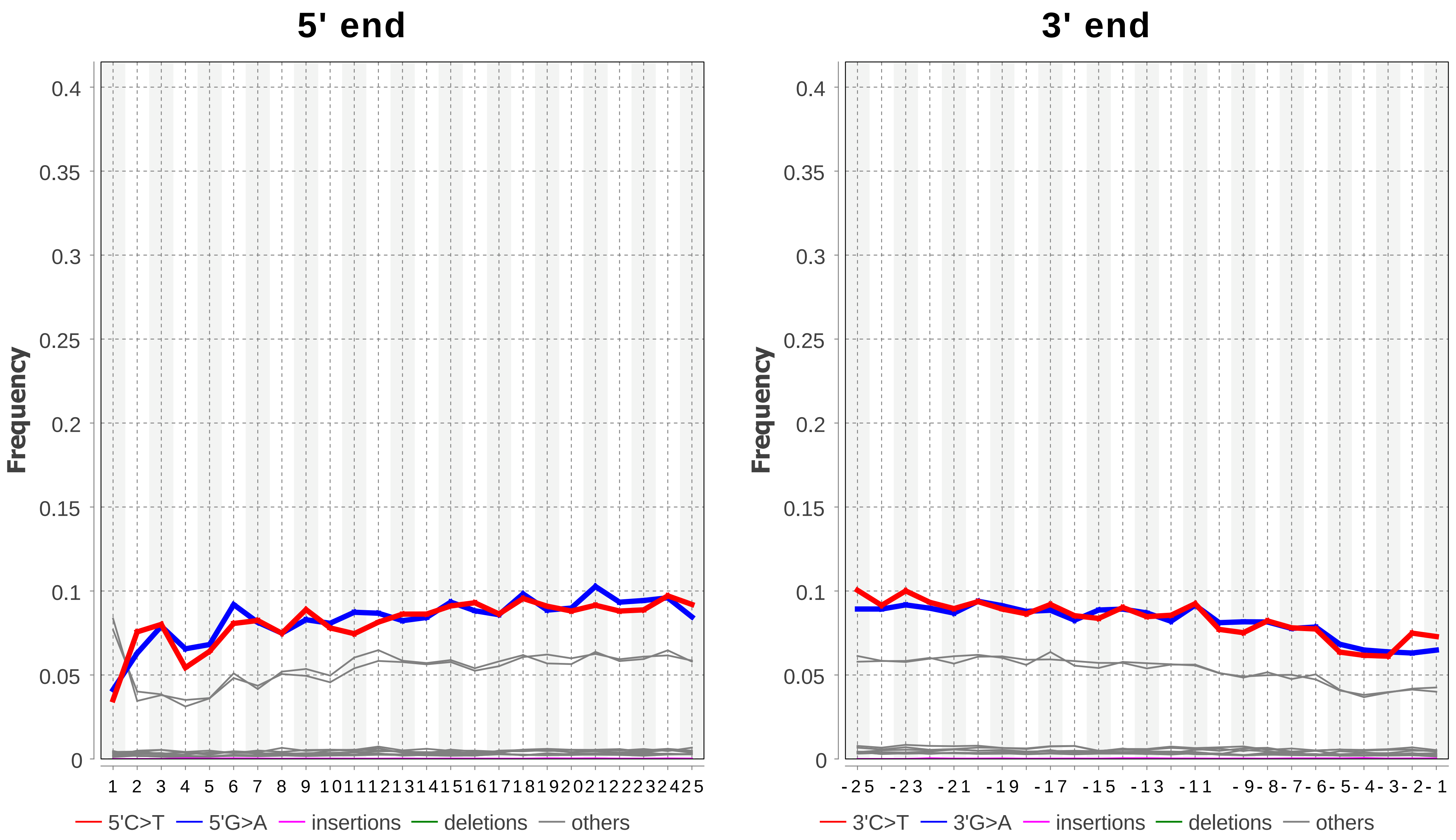

2001_aln

Number of used reads: 325 (100.0% of all input reads)

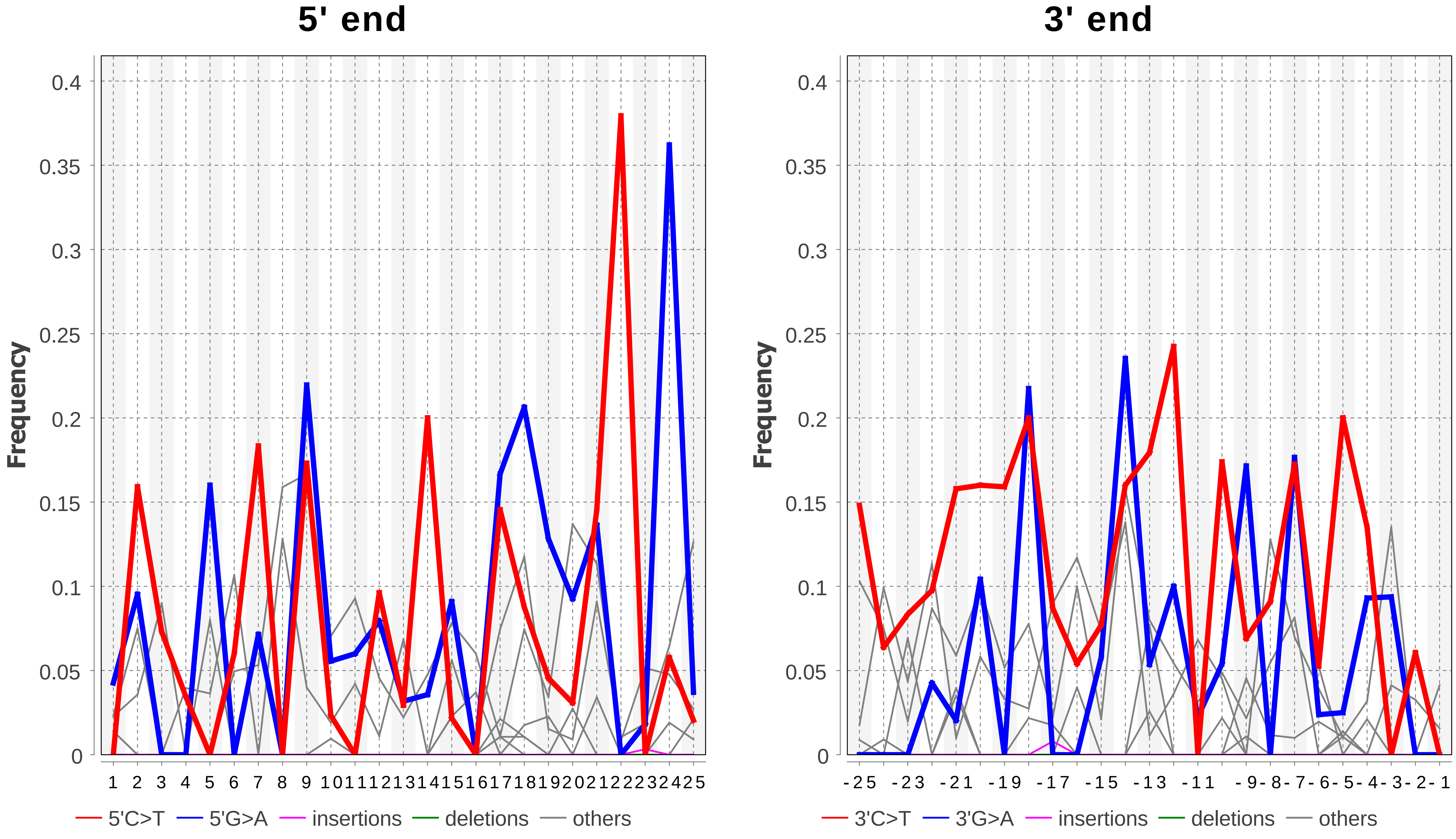

2001_MarkDuplicates
Number of used reads: 136 (100.0% of all input reads)

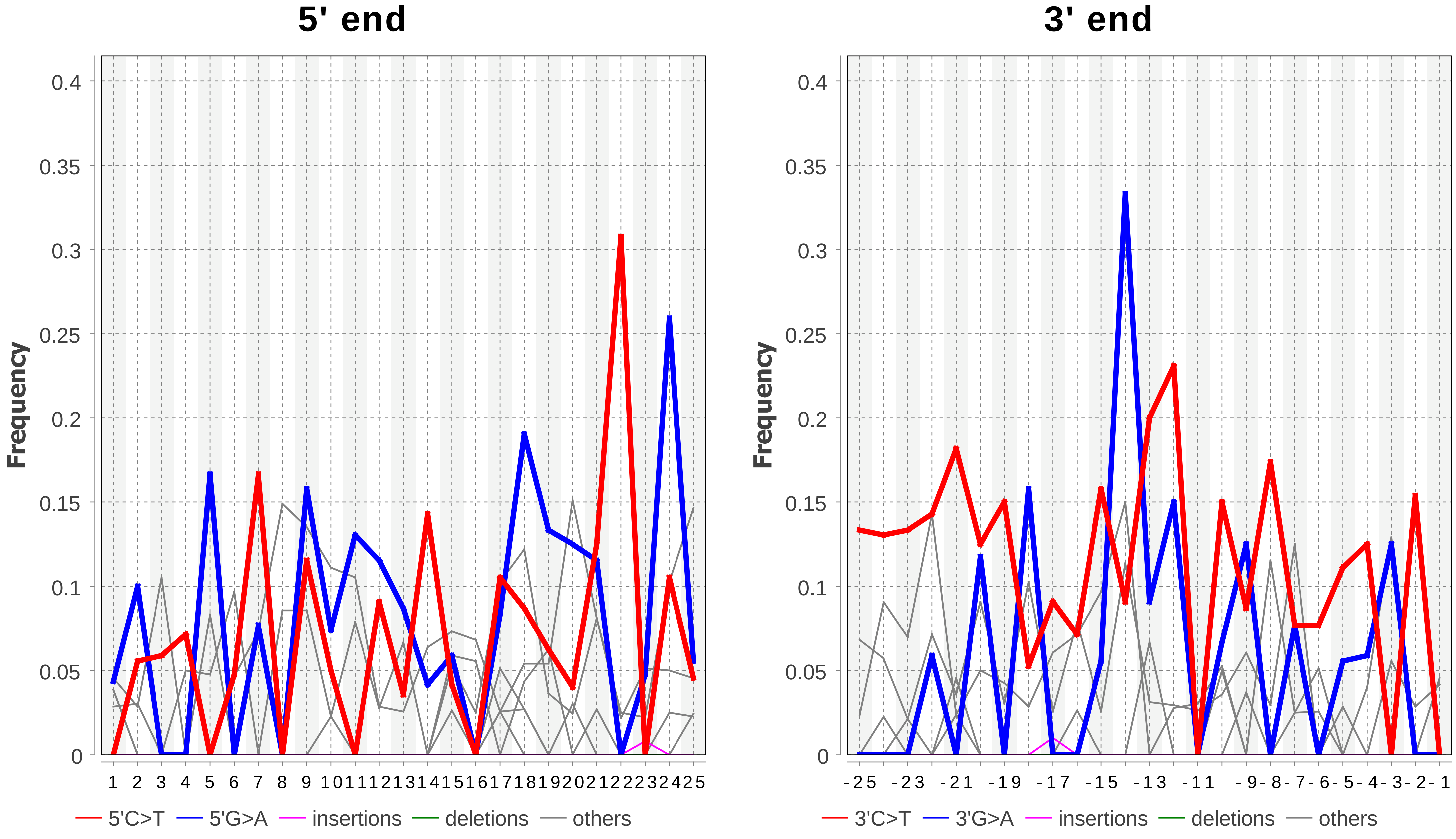

2006_aln

Number of used reads: 153,647 (100.0% of all input reads)

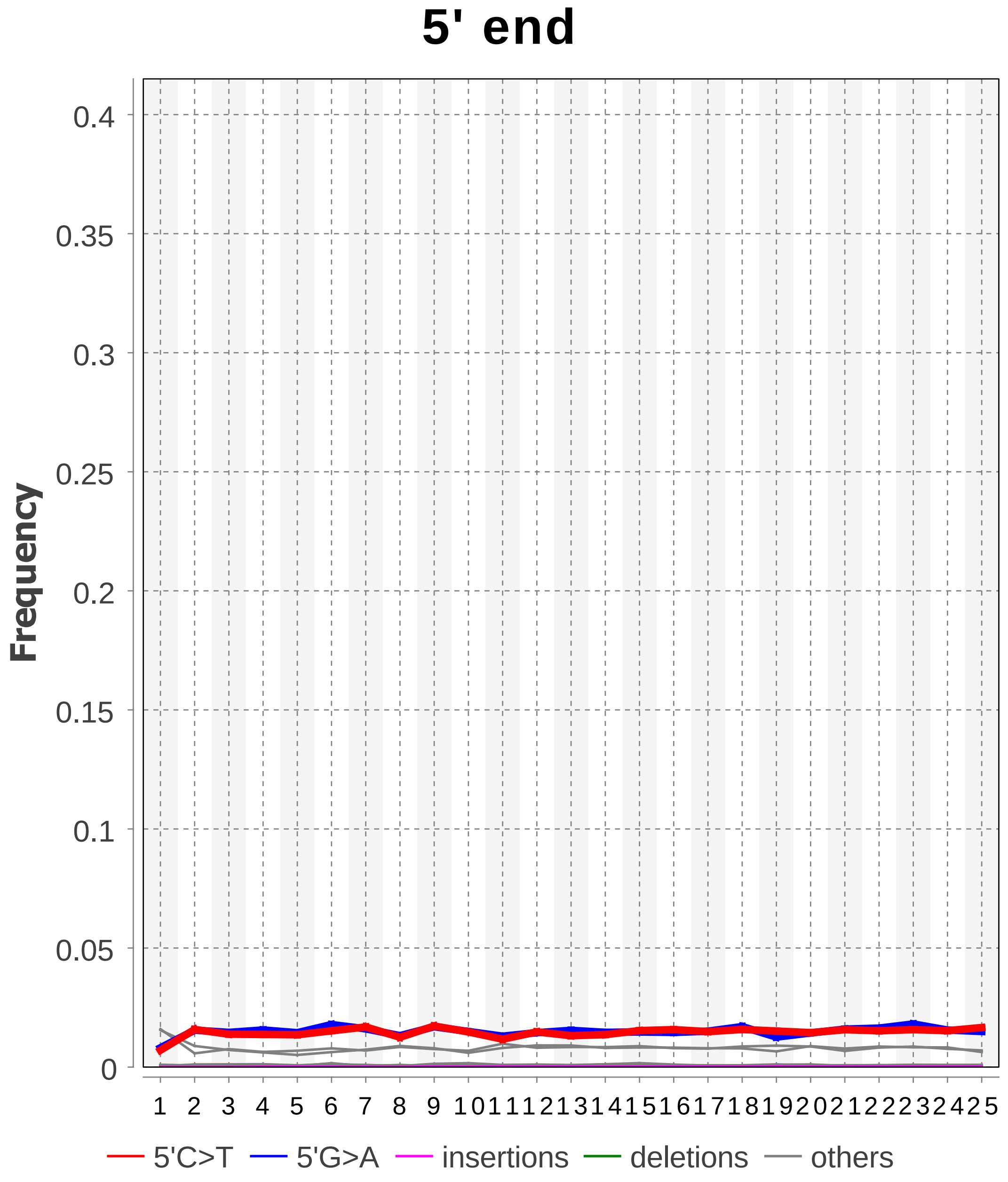

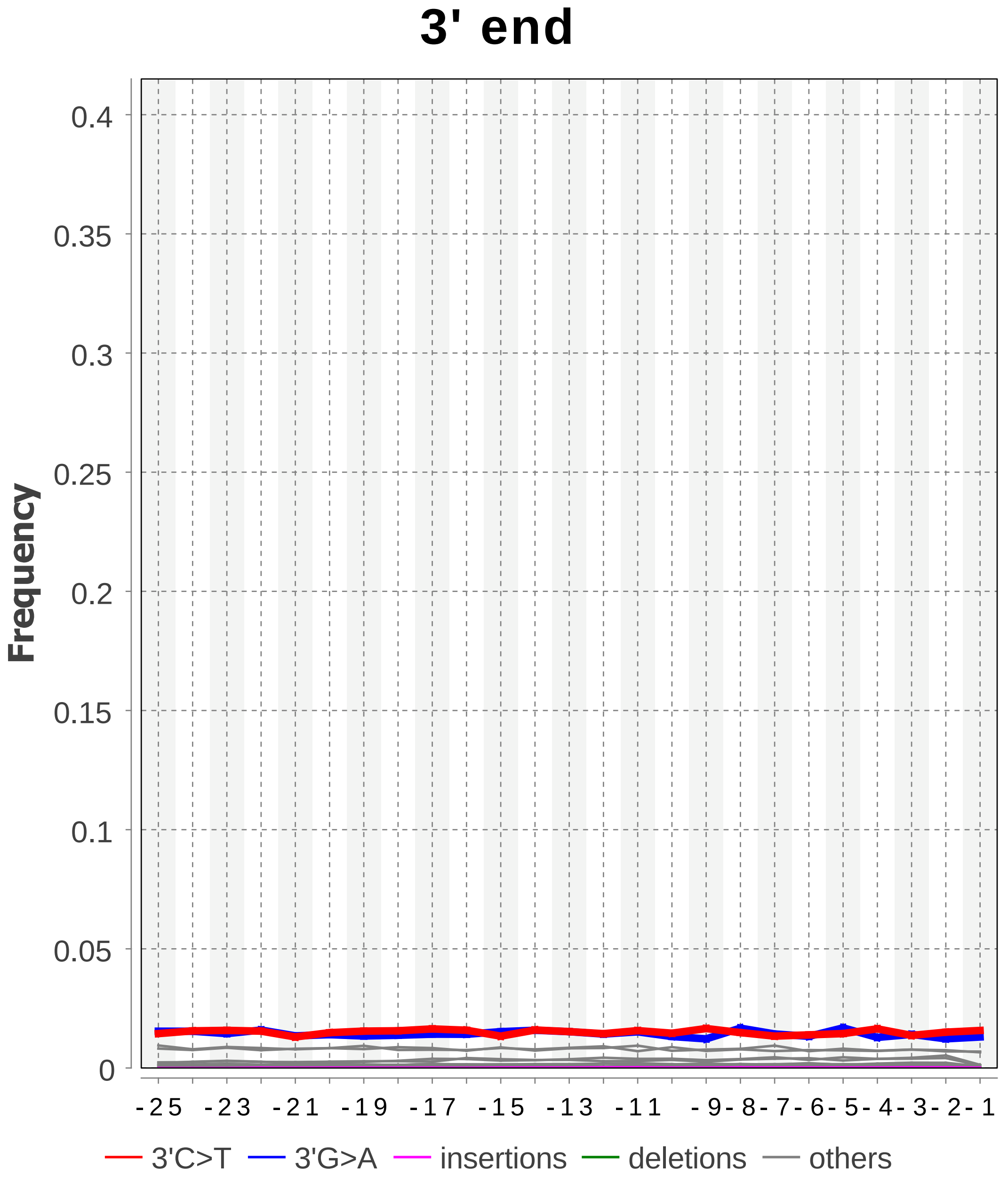

2006_MarkDuplicates

Number of used reads: 126,760 (100.0% of all input reads)

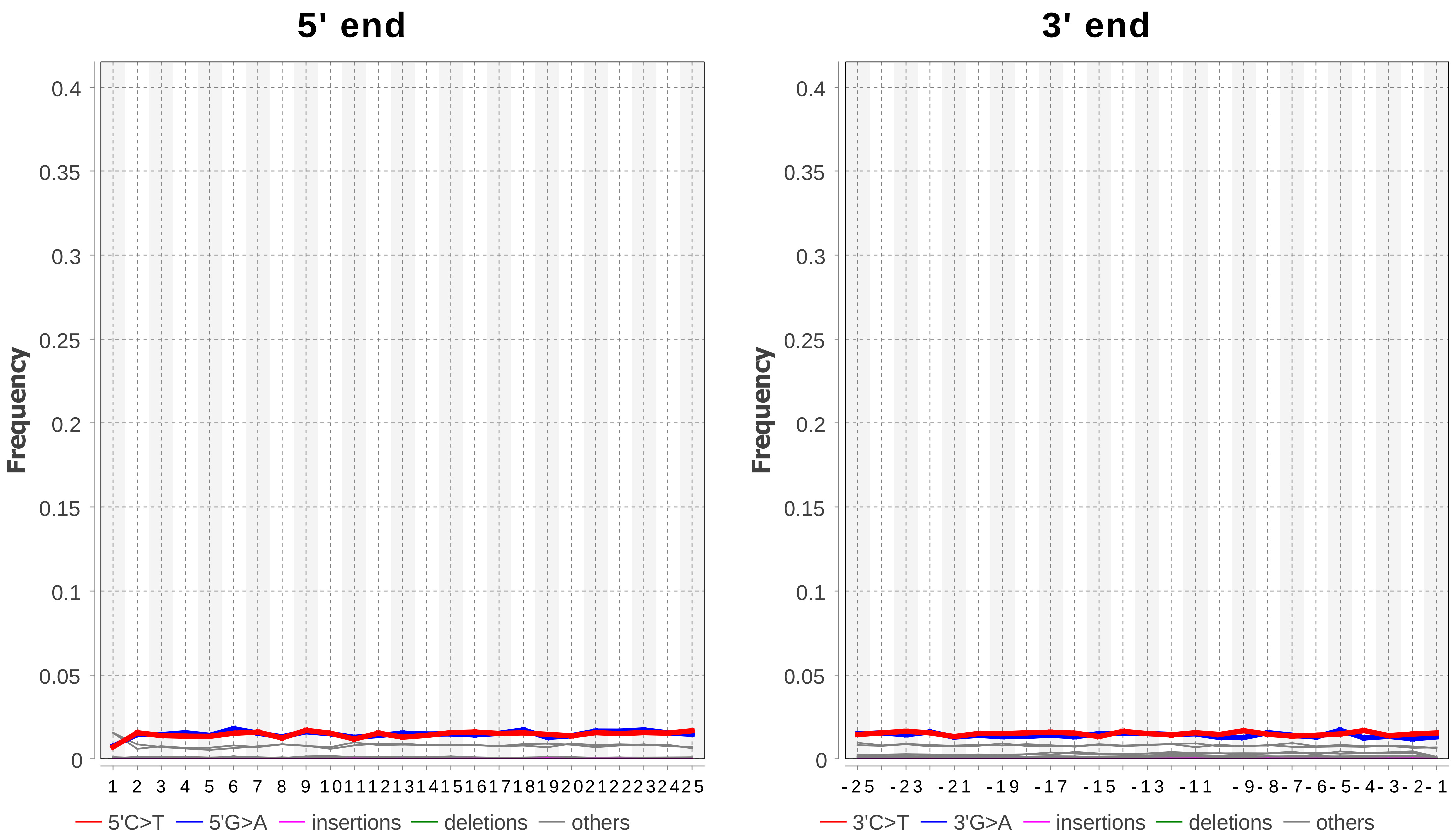

2093_aln
Number of used reads: 70,551 (100.0% of all input reads)

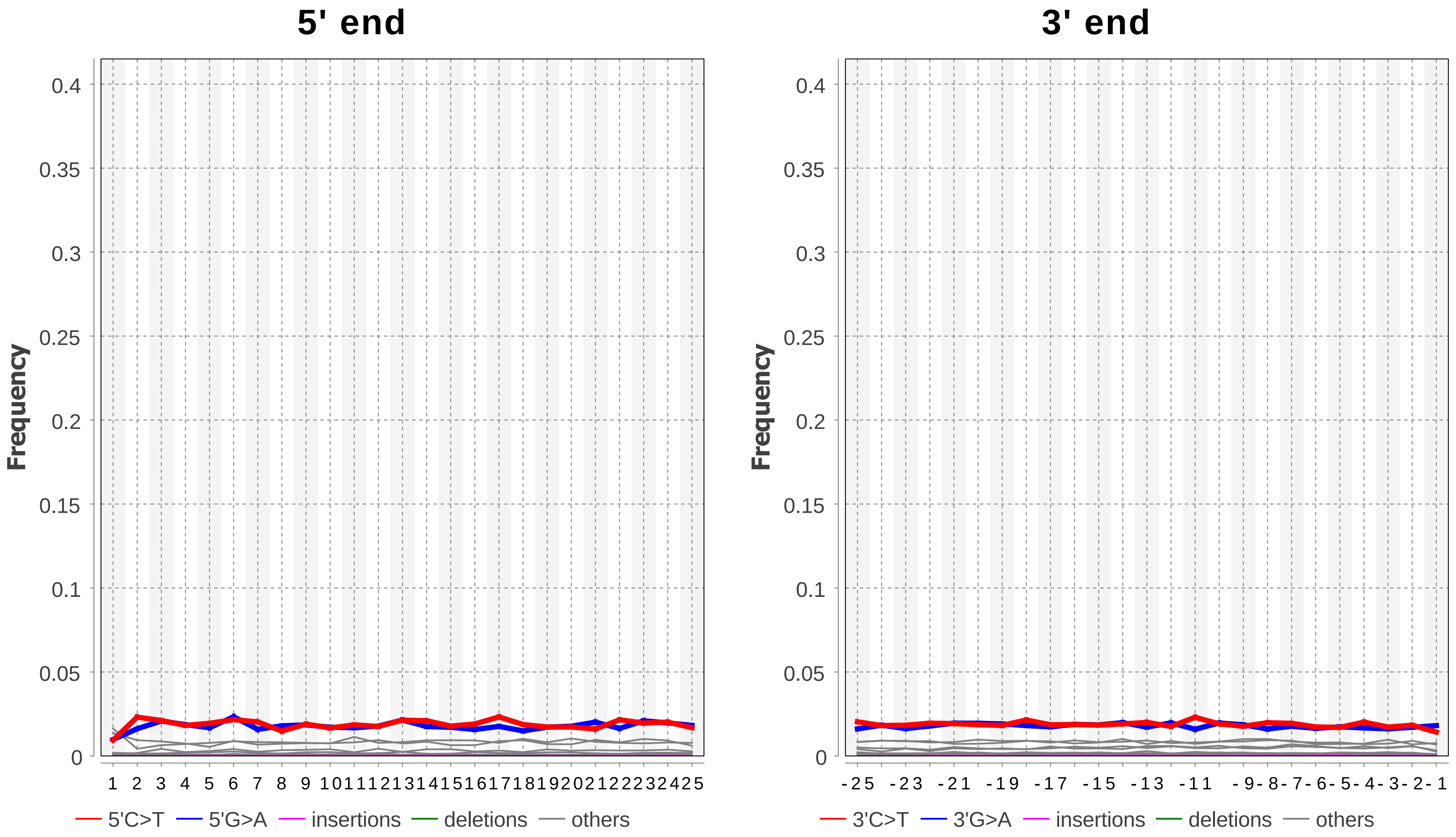

2093_MarkDuplicates
Number of used reads: 56,593 (100.0% of all input reads)

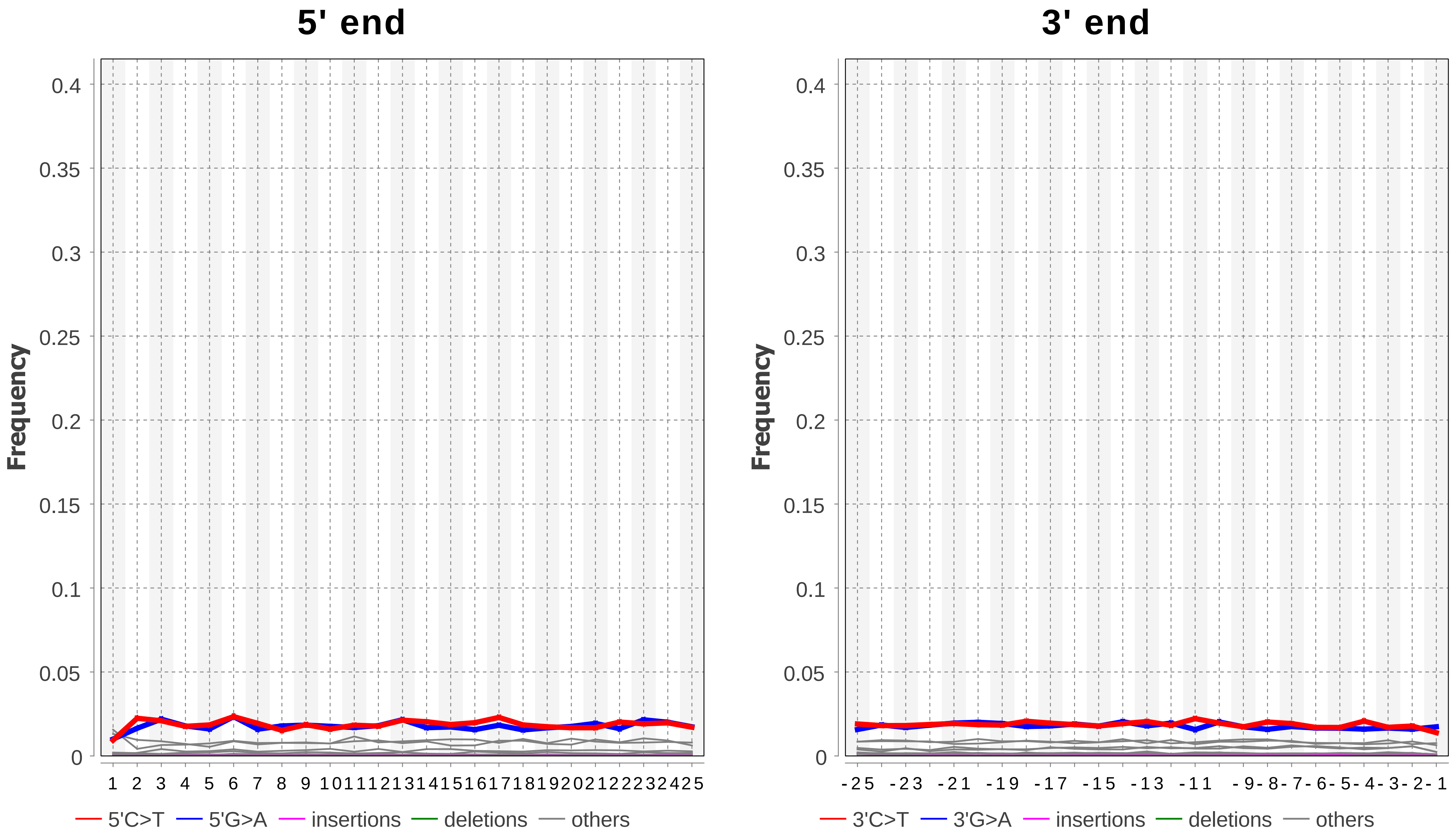

2213_aln

Number of used reads: 83,704 (100.0% of all input reads)

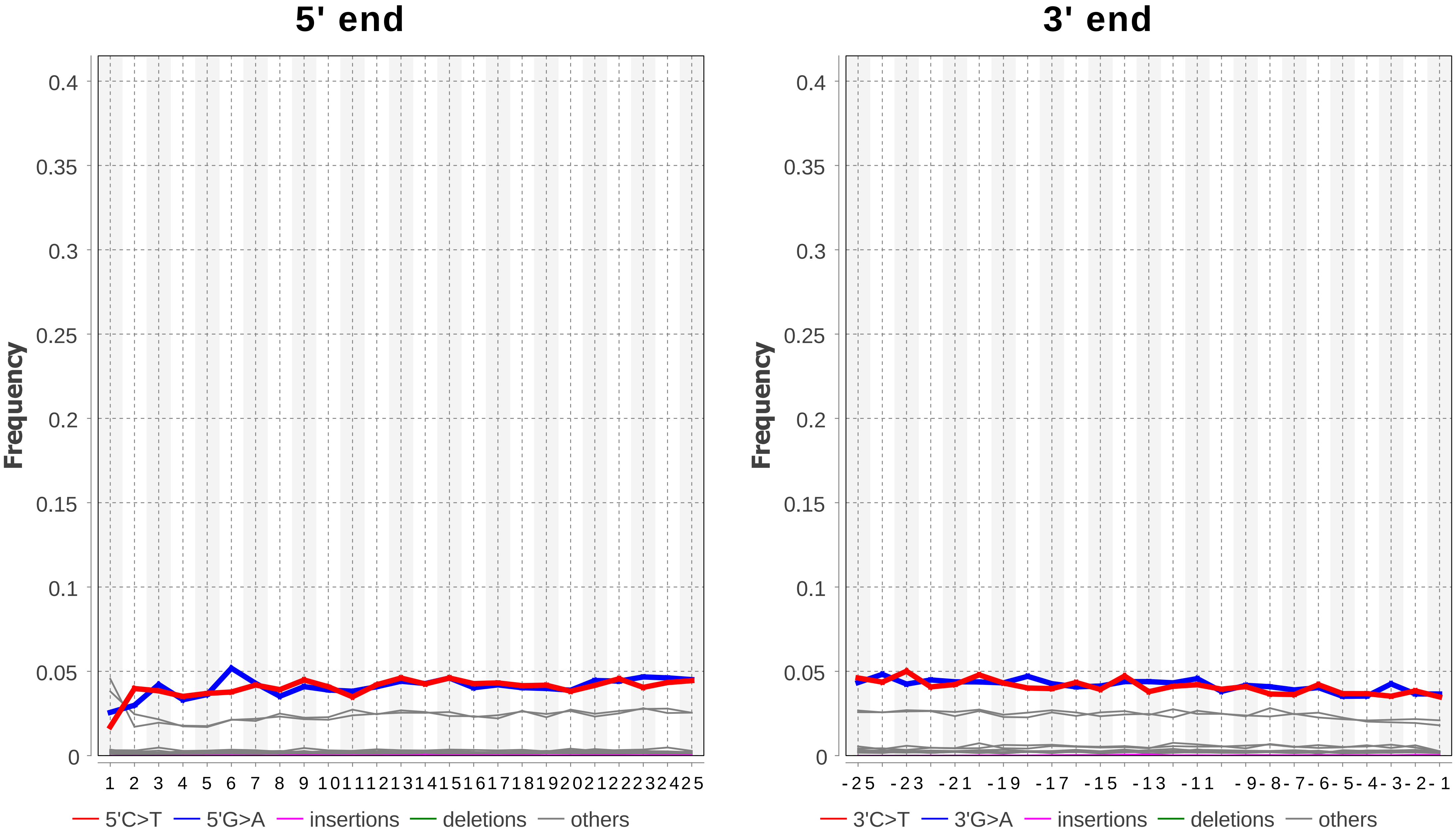

2213_MarkDuplicates

Number of used reads: 68,837 (100.0% of all input reads)

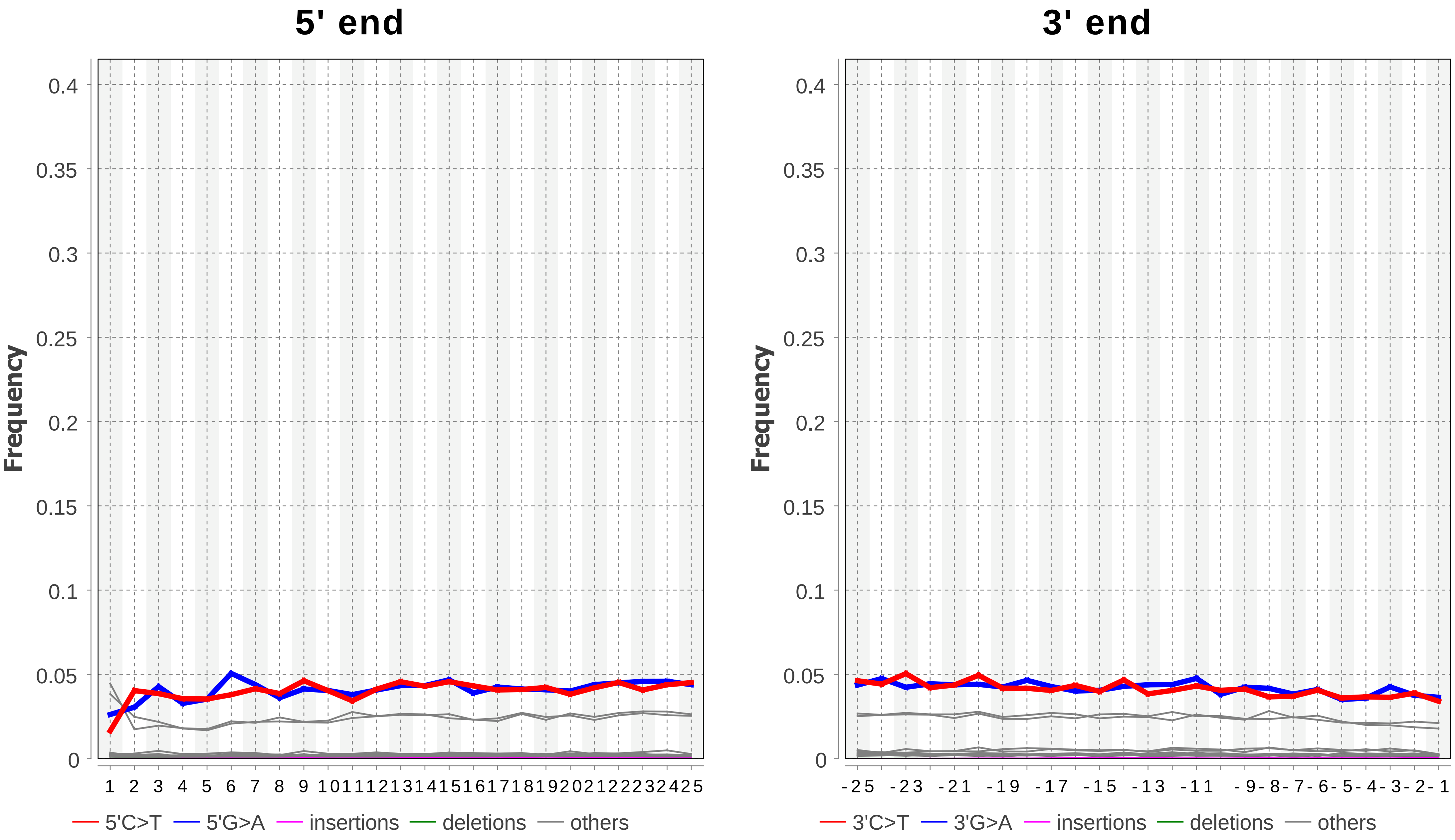

2302_aln
Number of used reads: 69,482 (100.0% of all input reads)

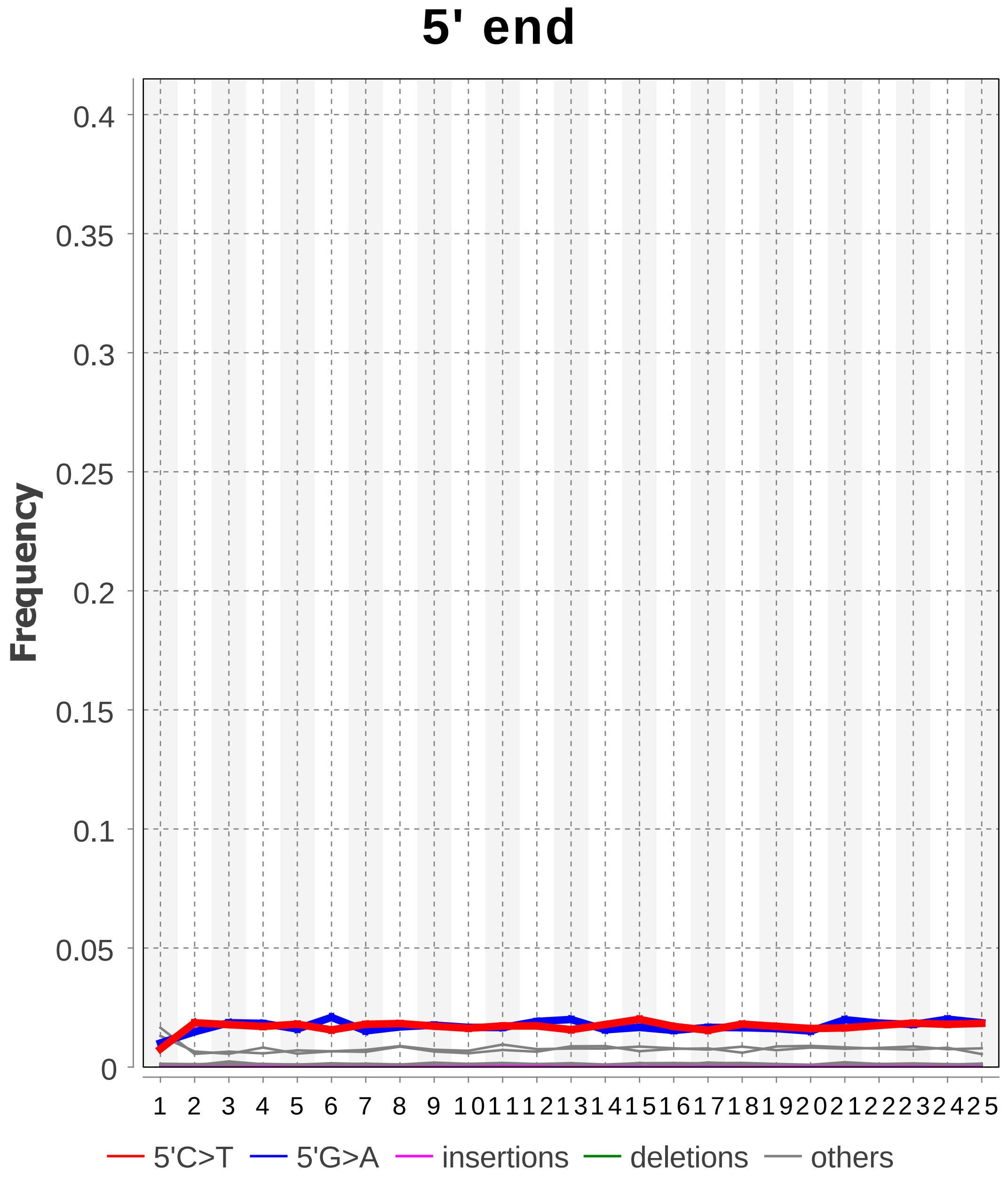

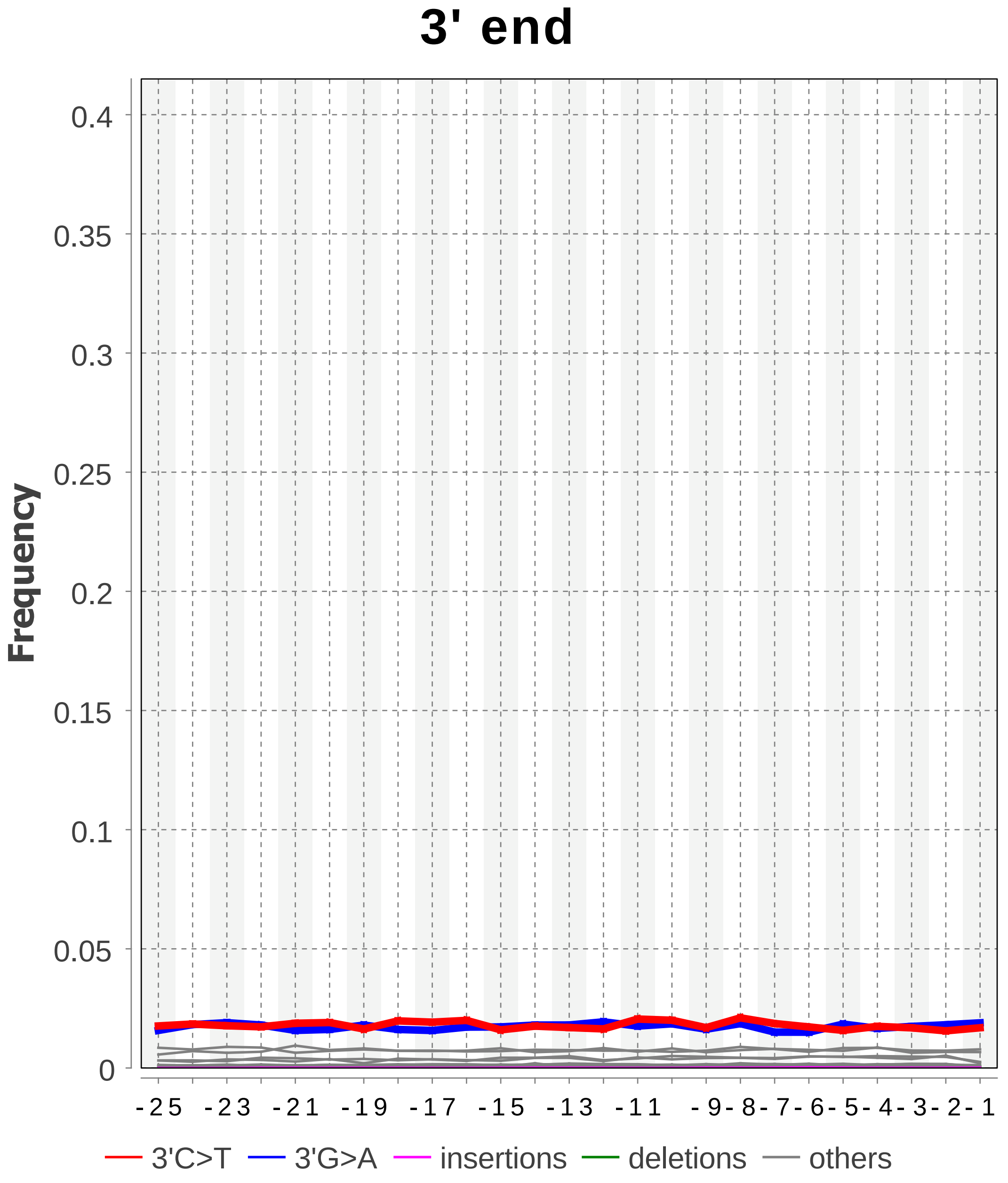

2302_MarkDuplicates

Number of used reads: 59,916 (100.0% of all input reads)

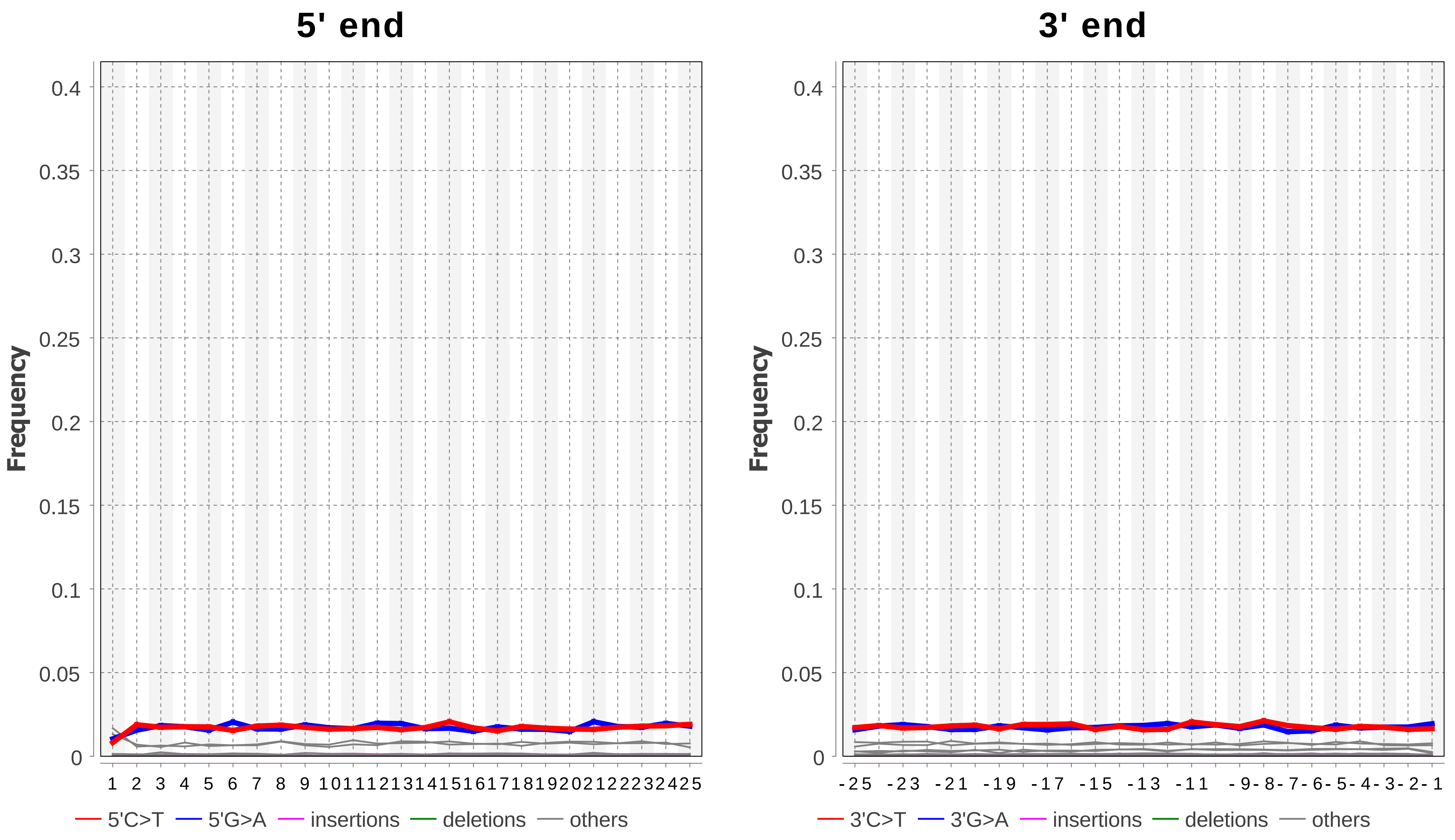

2312_aln

Number of used reads: 52,476 (100.0% of all input reads)

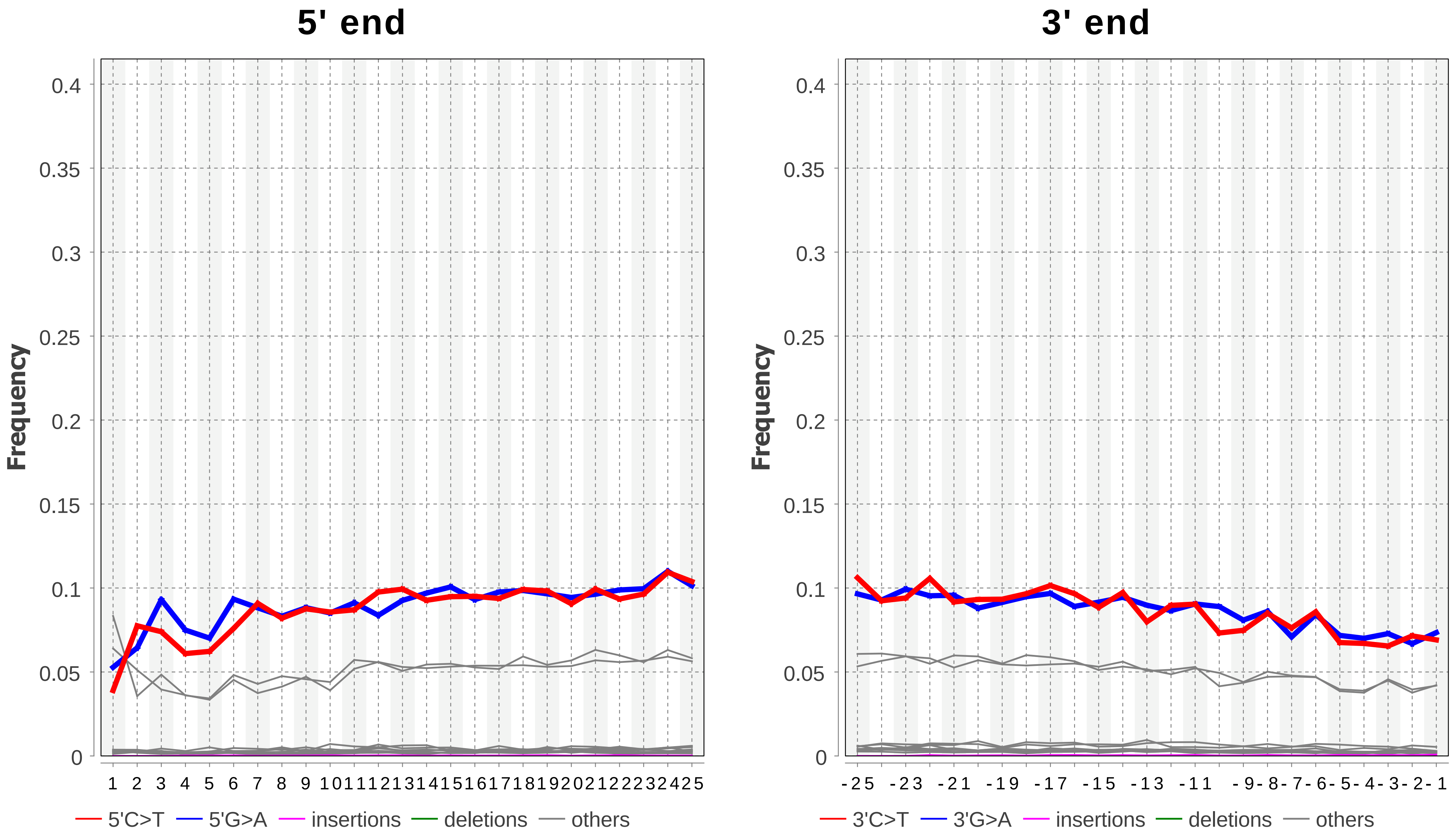

2312_MarkDuplicates
Number of used reads: 42,300 (100.0% of all input reads)

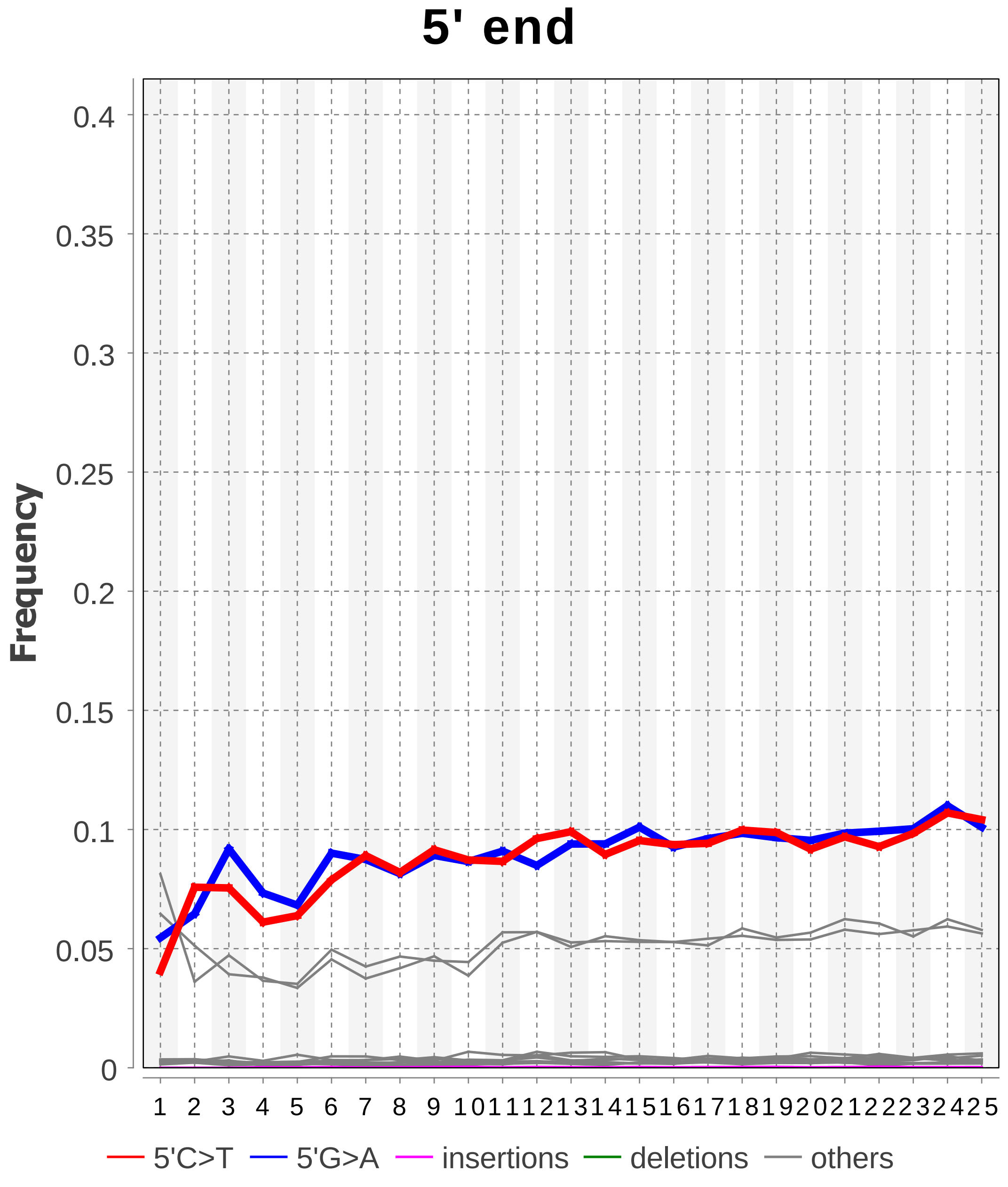

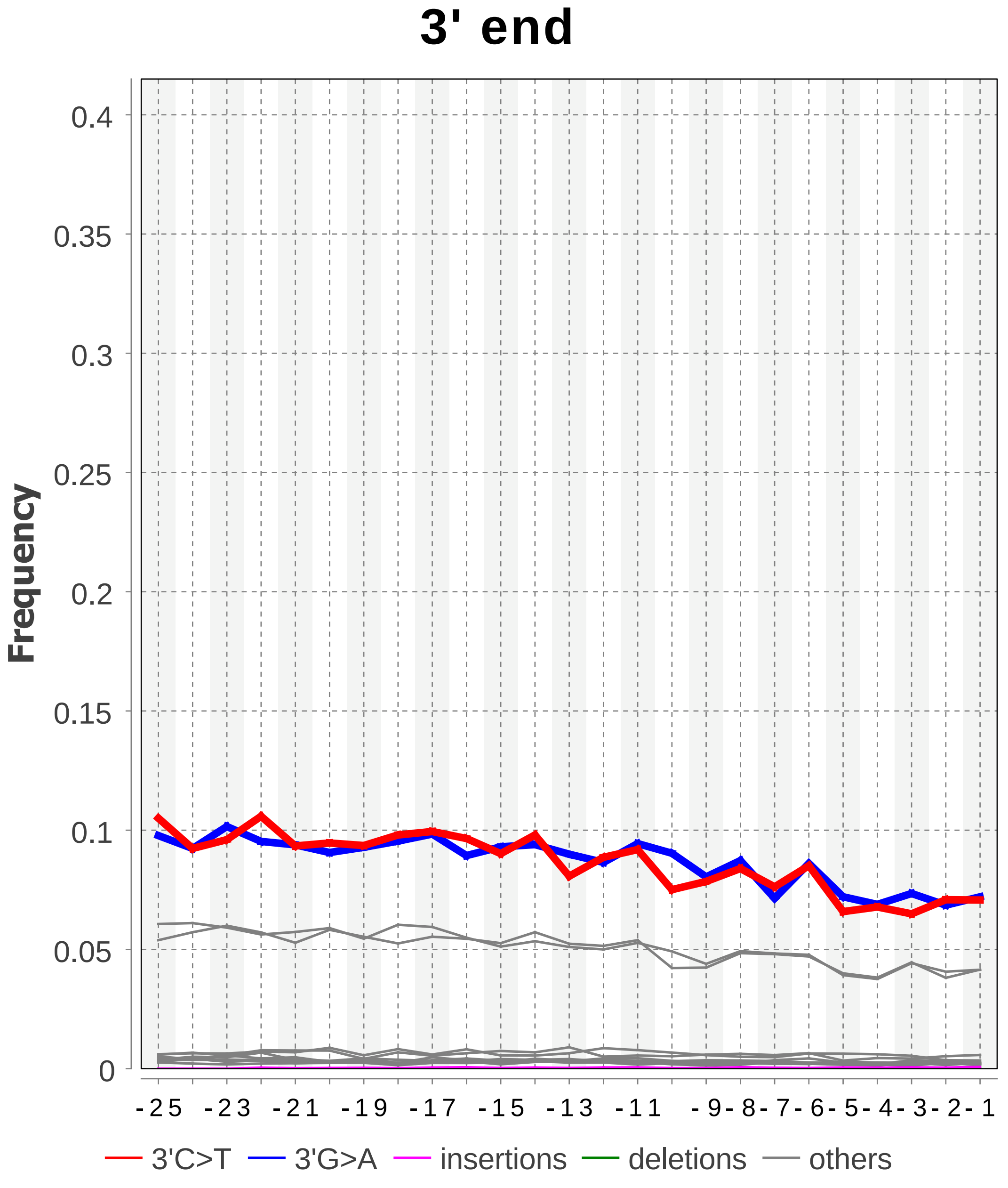

2345_aln
Number of used reads: 38,553 (100.0% of all input reads)

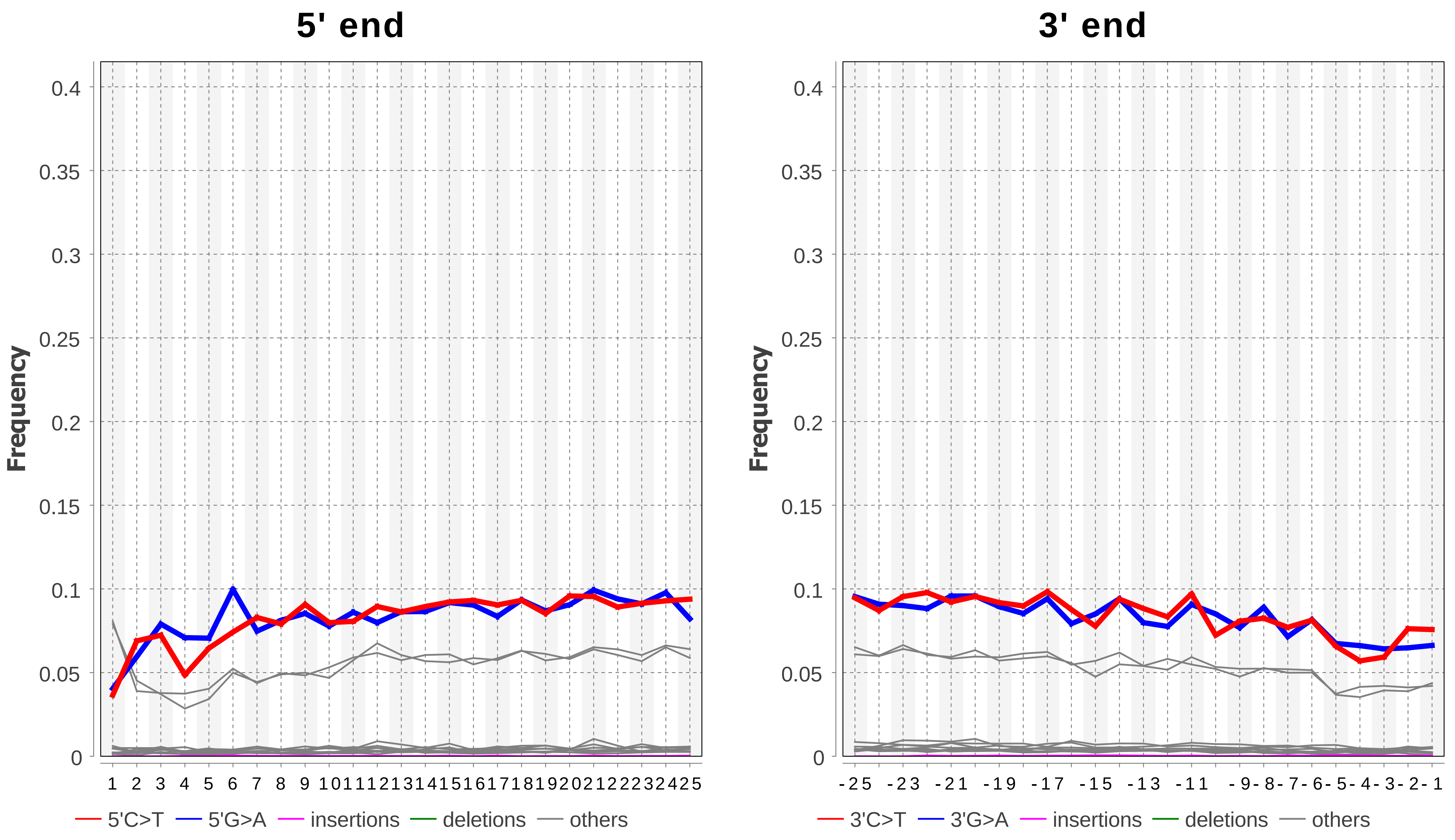

2345_MarkDuplicates

Number of used reads: 31,719 (100.0% of all input reads)

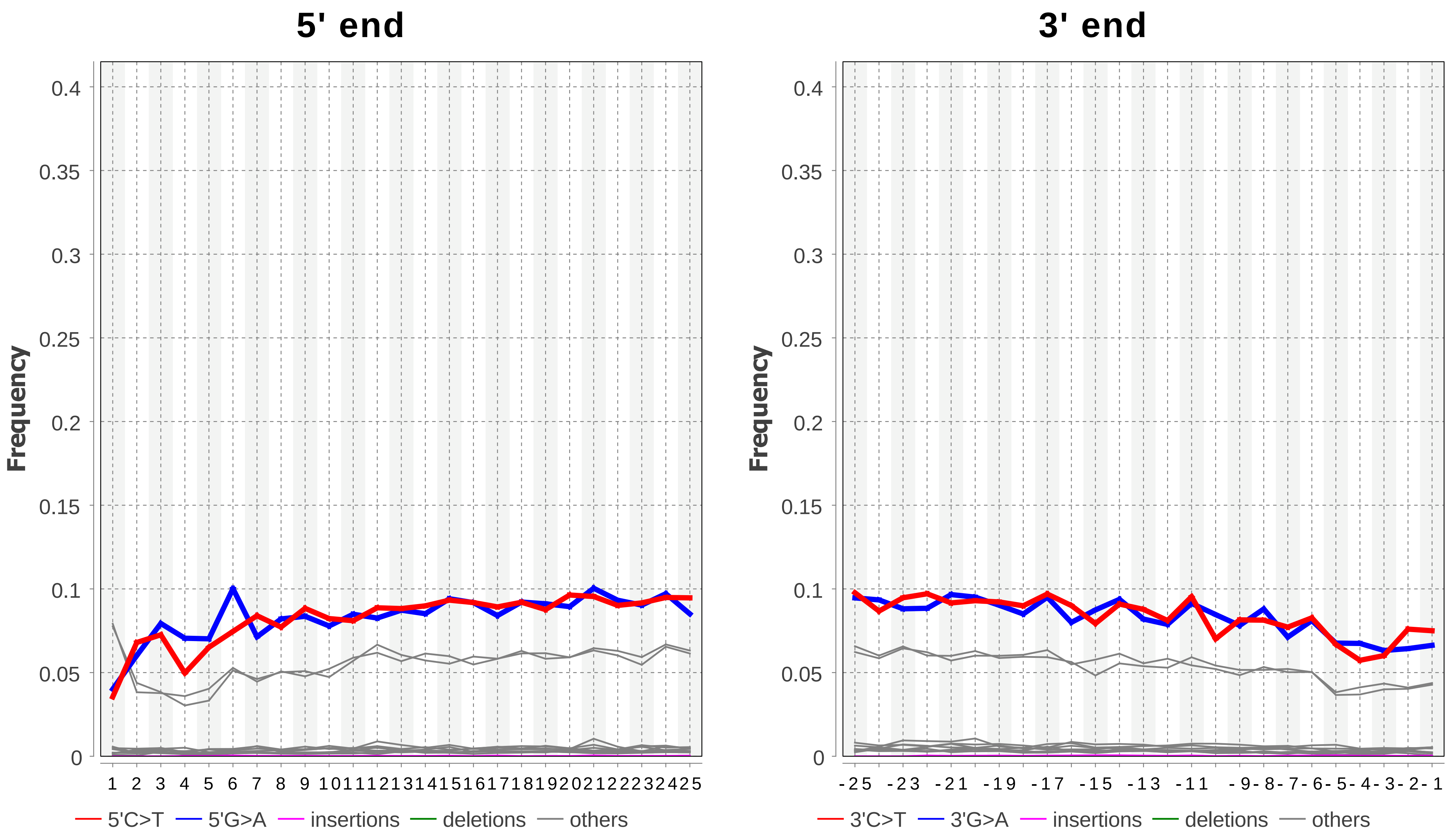

2385_aln

Number of used reads: 53,429 (100.0% of all input reads)

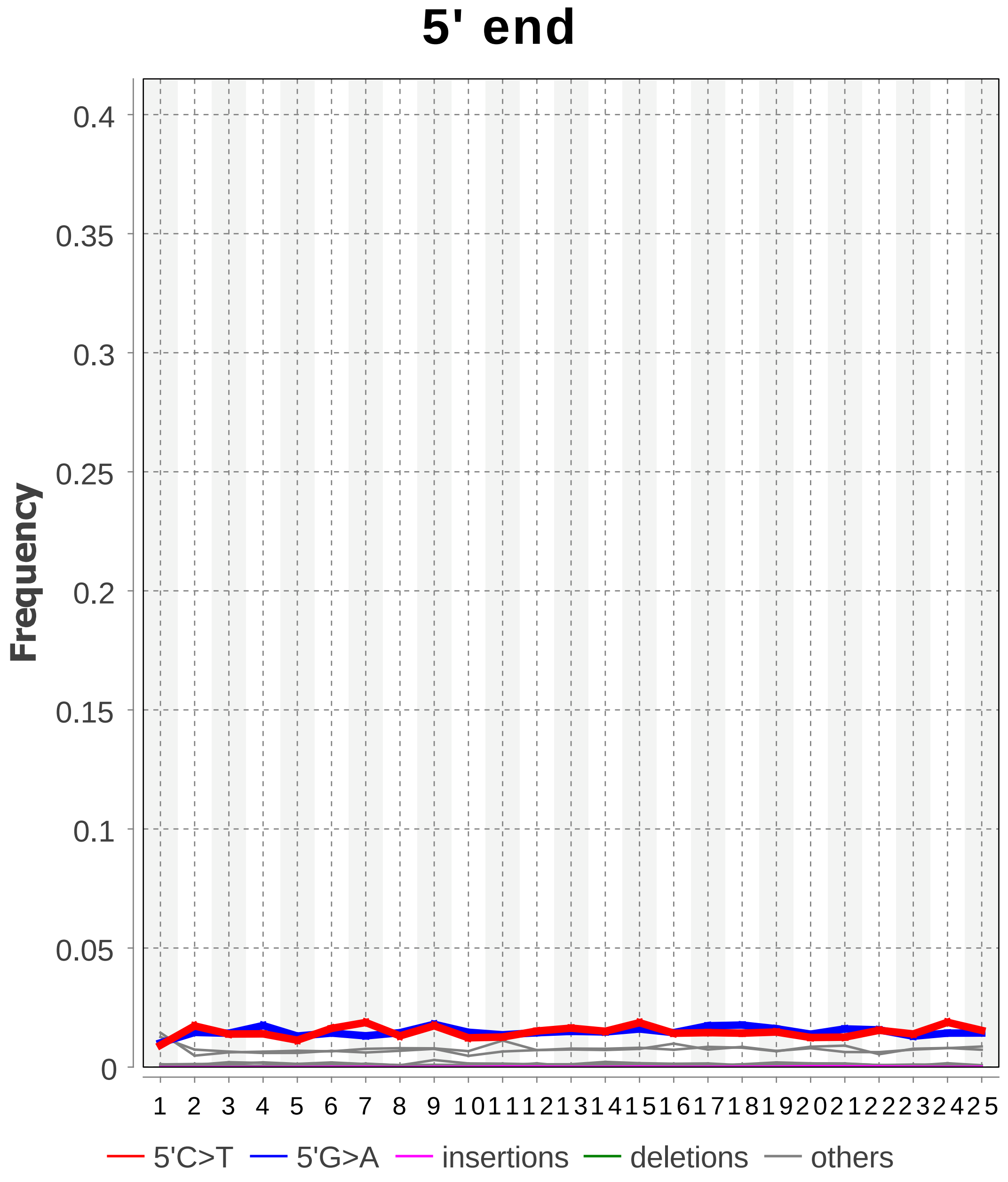

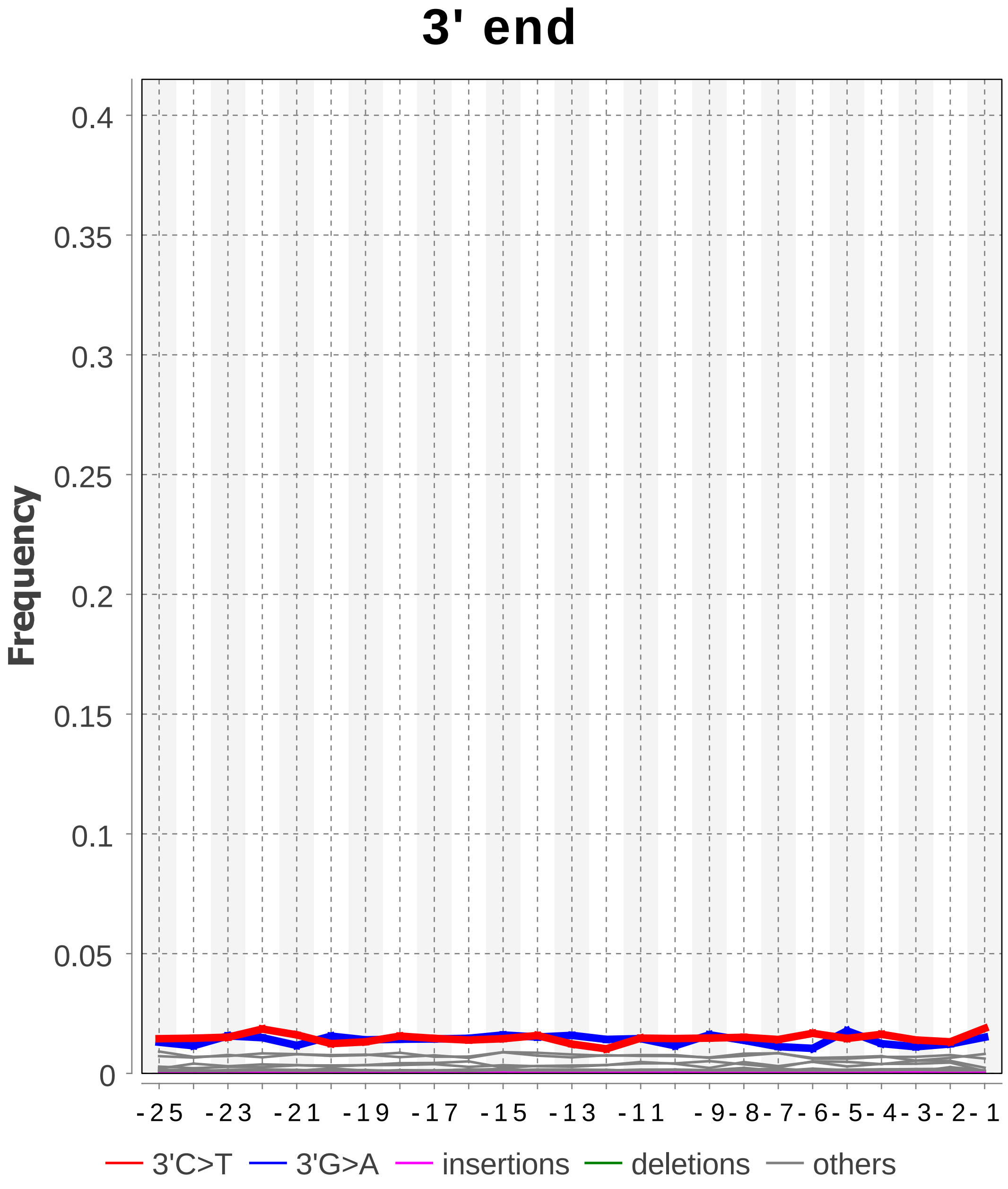

2385_MarkDuplicates

Number of used reads: 45,475 (100.0% of all input reads)

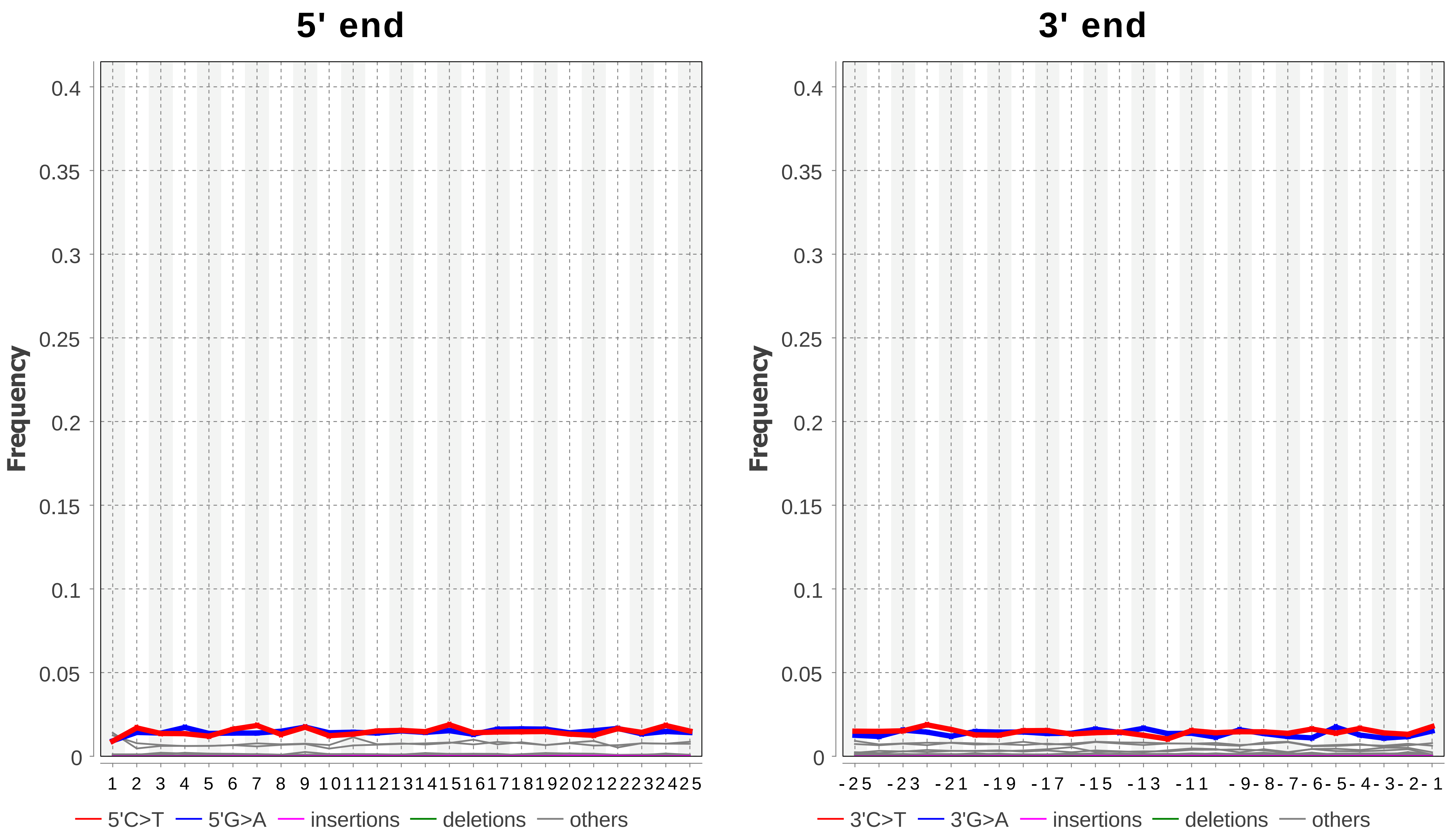

2394_aln
Number of used reads: 180,800 (100.0% of all input reads)

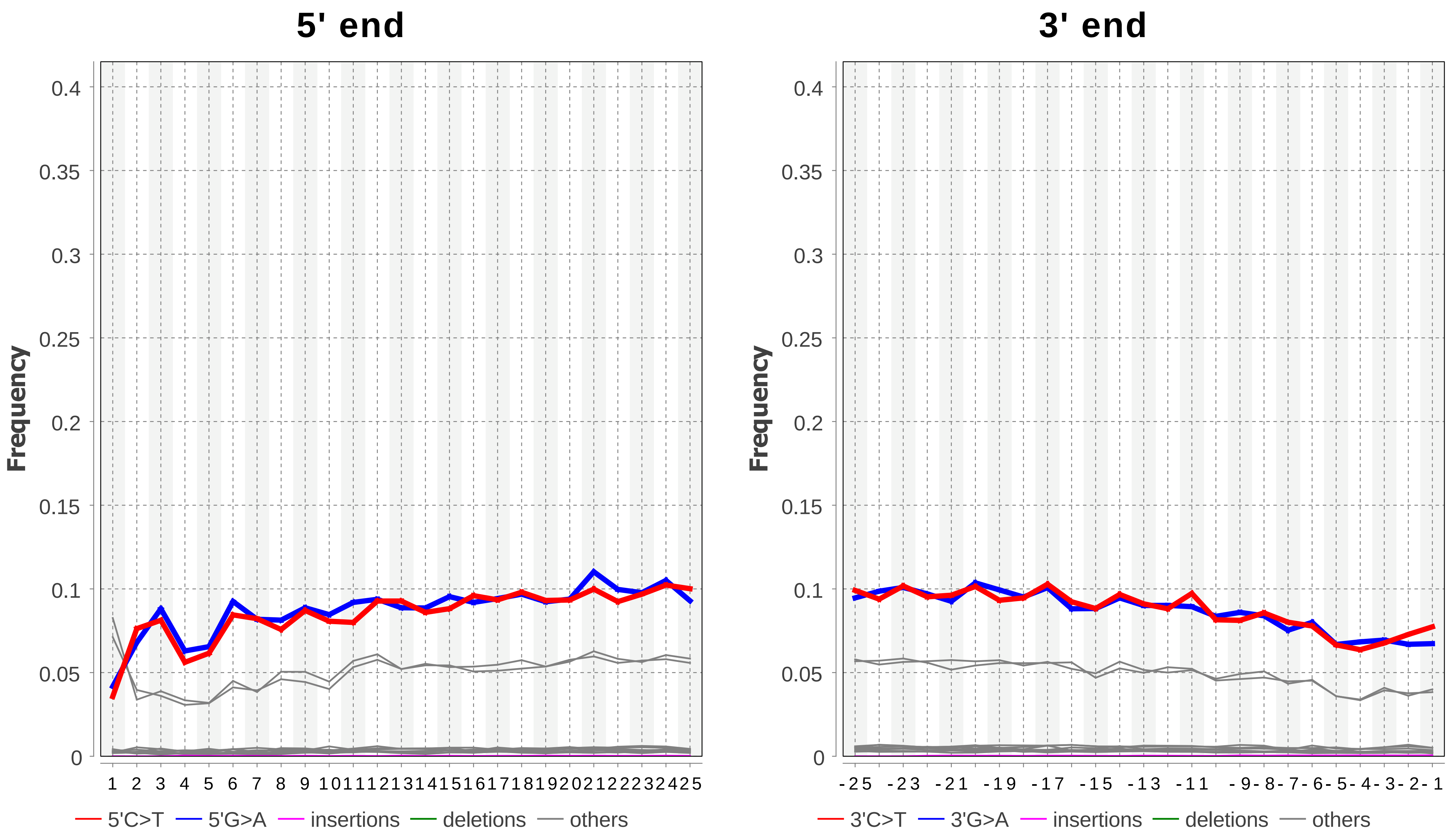

2394_MarkDuplicates

Number of used reads: 143,408 (100.0% of all input reads)

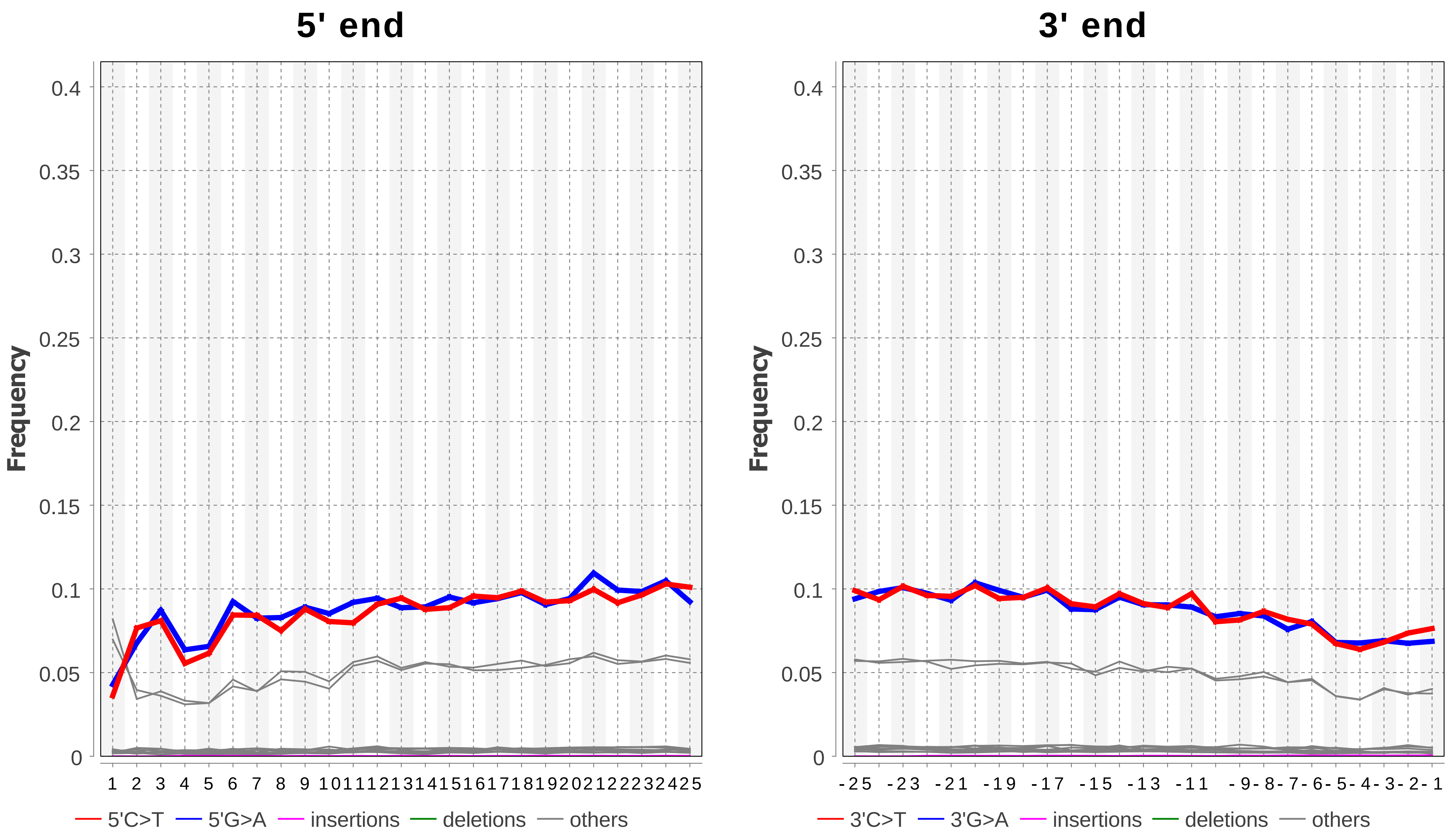

2411_aln
Number of used reads: 134,479 (100.0% of all input reads)

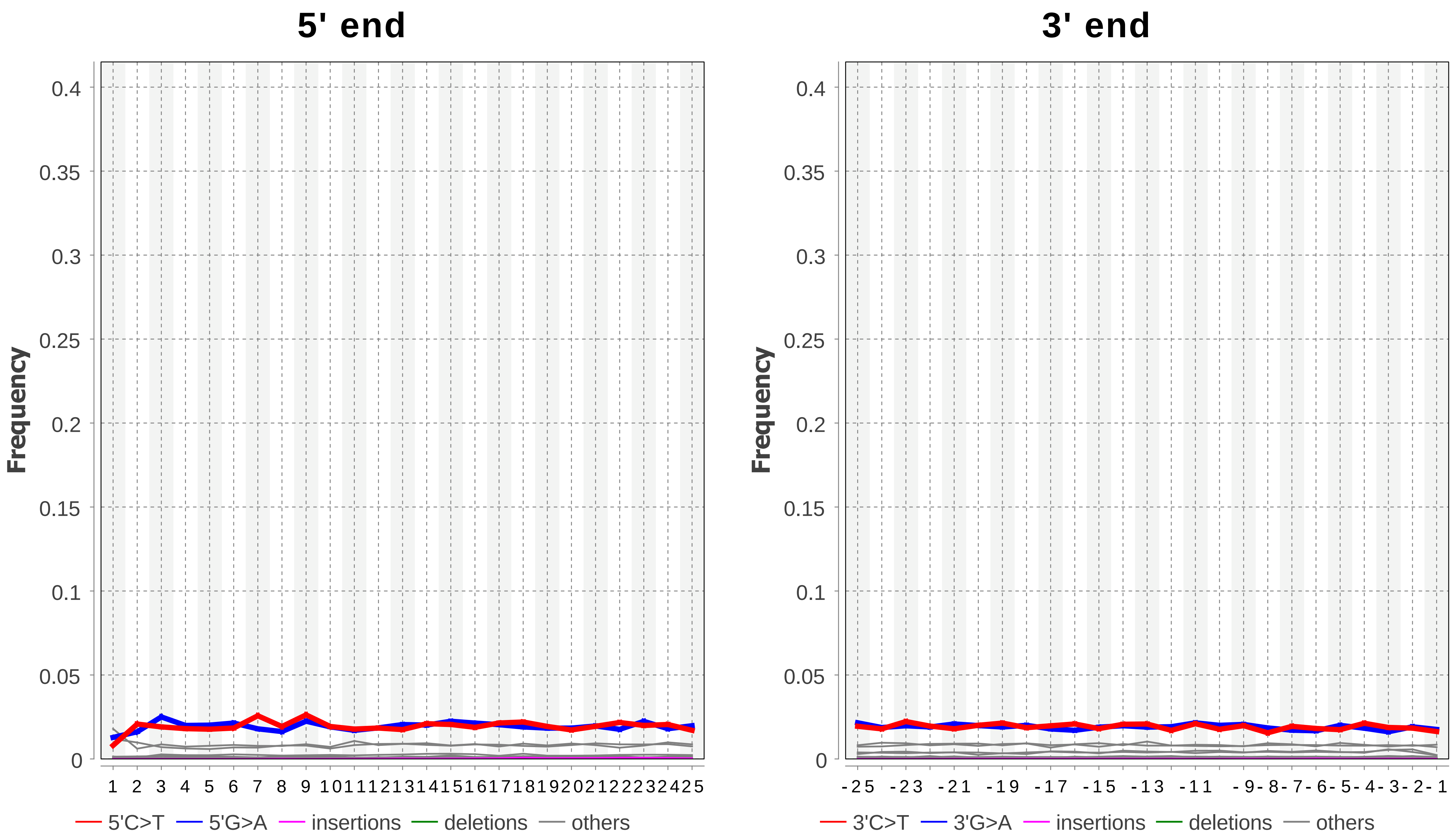

2411_MarkDuplicates
Number of used reads: 102,650 (100.0% of all input reads)

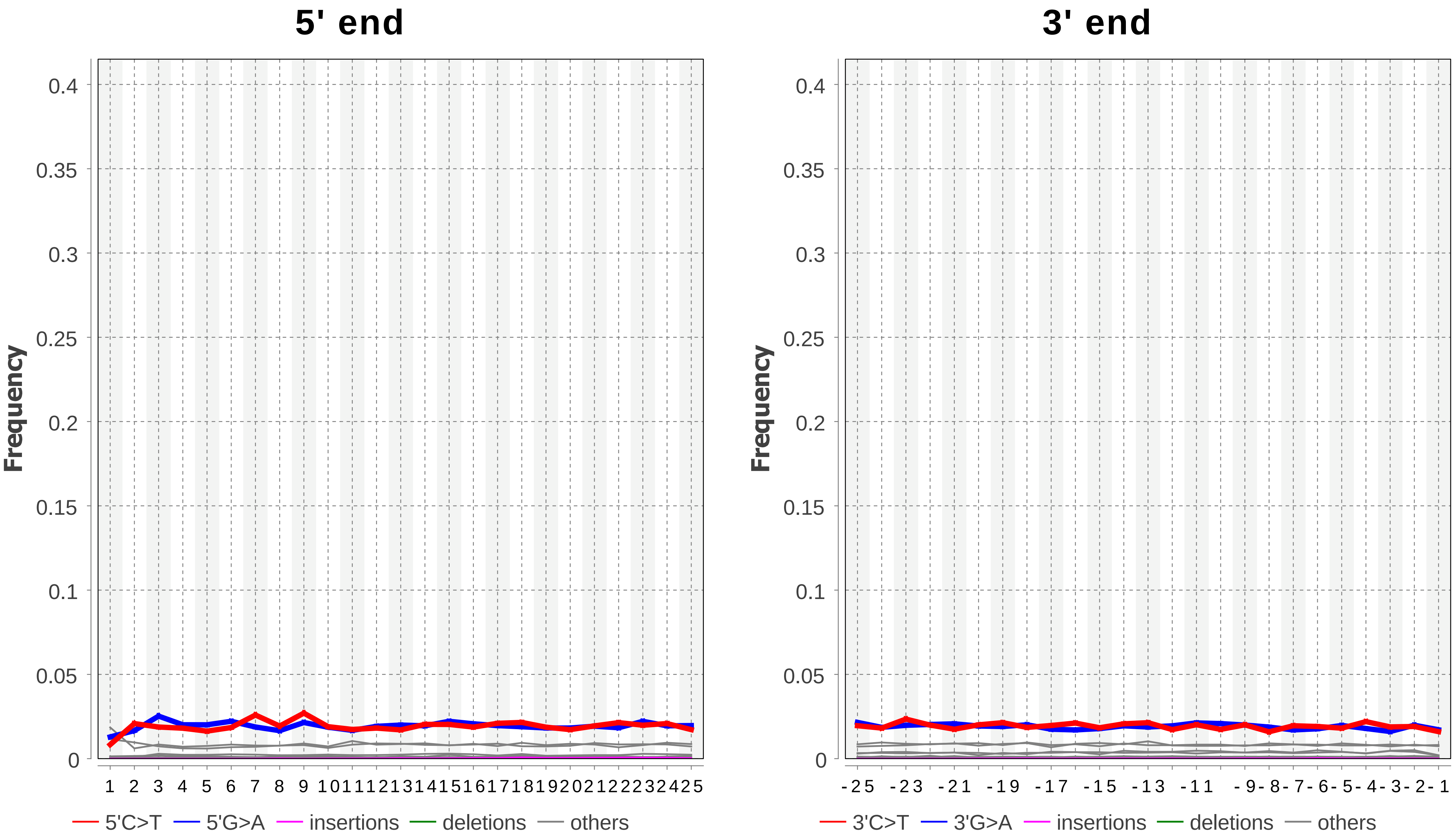

2448_aln

Number of used reads: 89,356 (100.0% of all input reads)

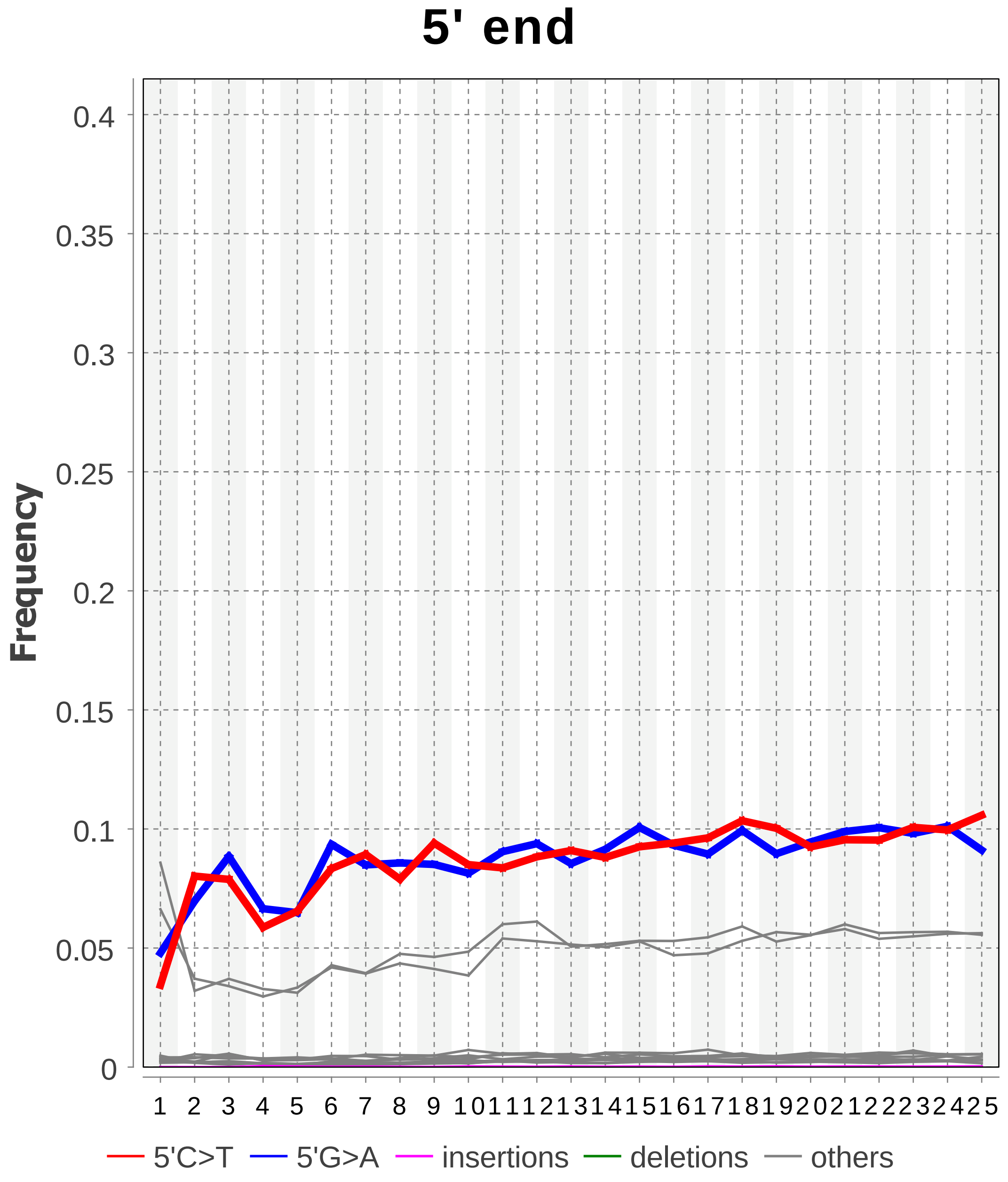

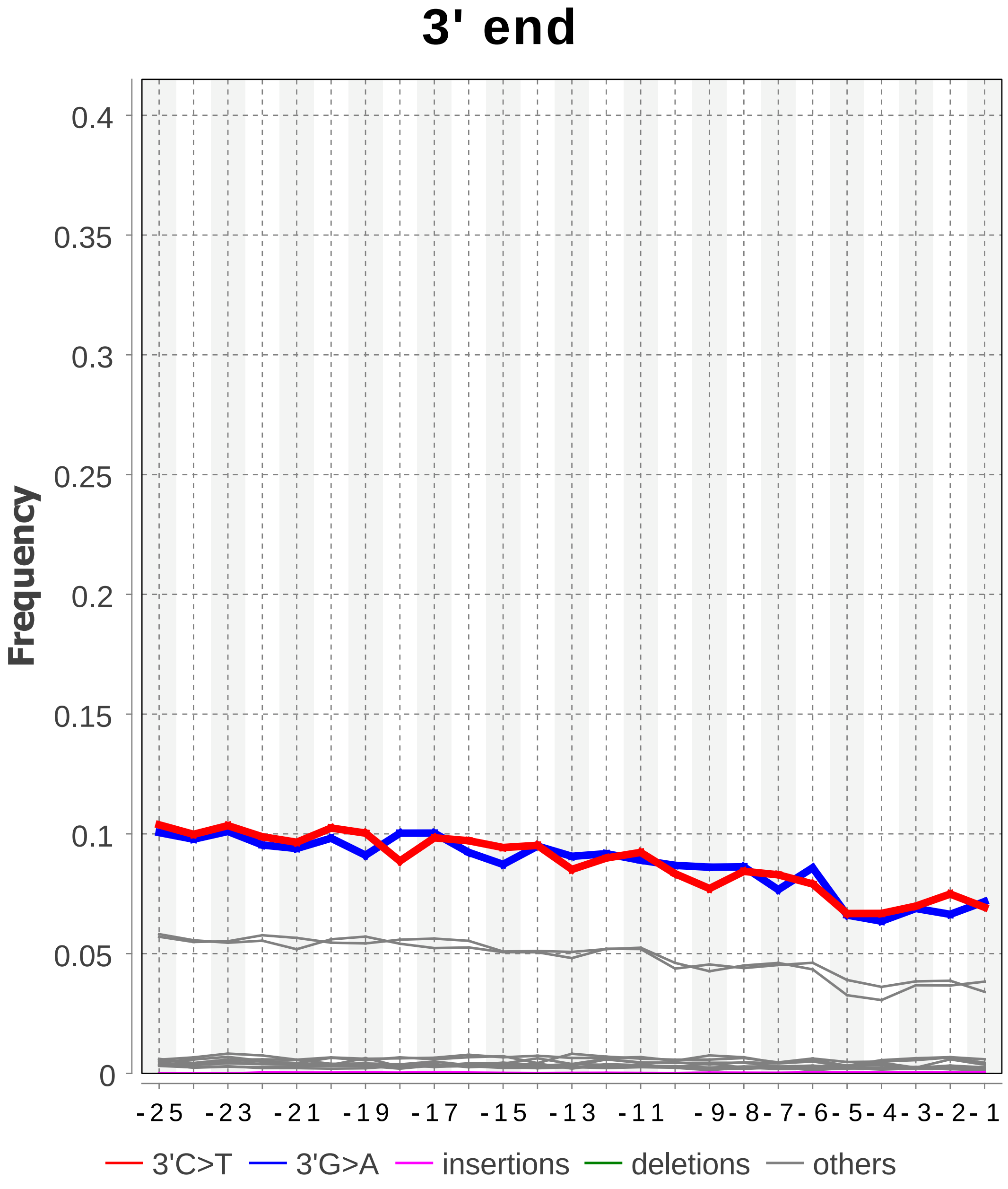

2448_MarkDuplicates

Number of used reads: 72,268 (100.0% of all input reads)

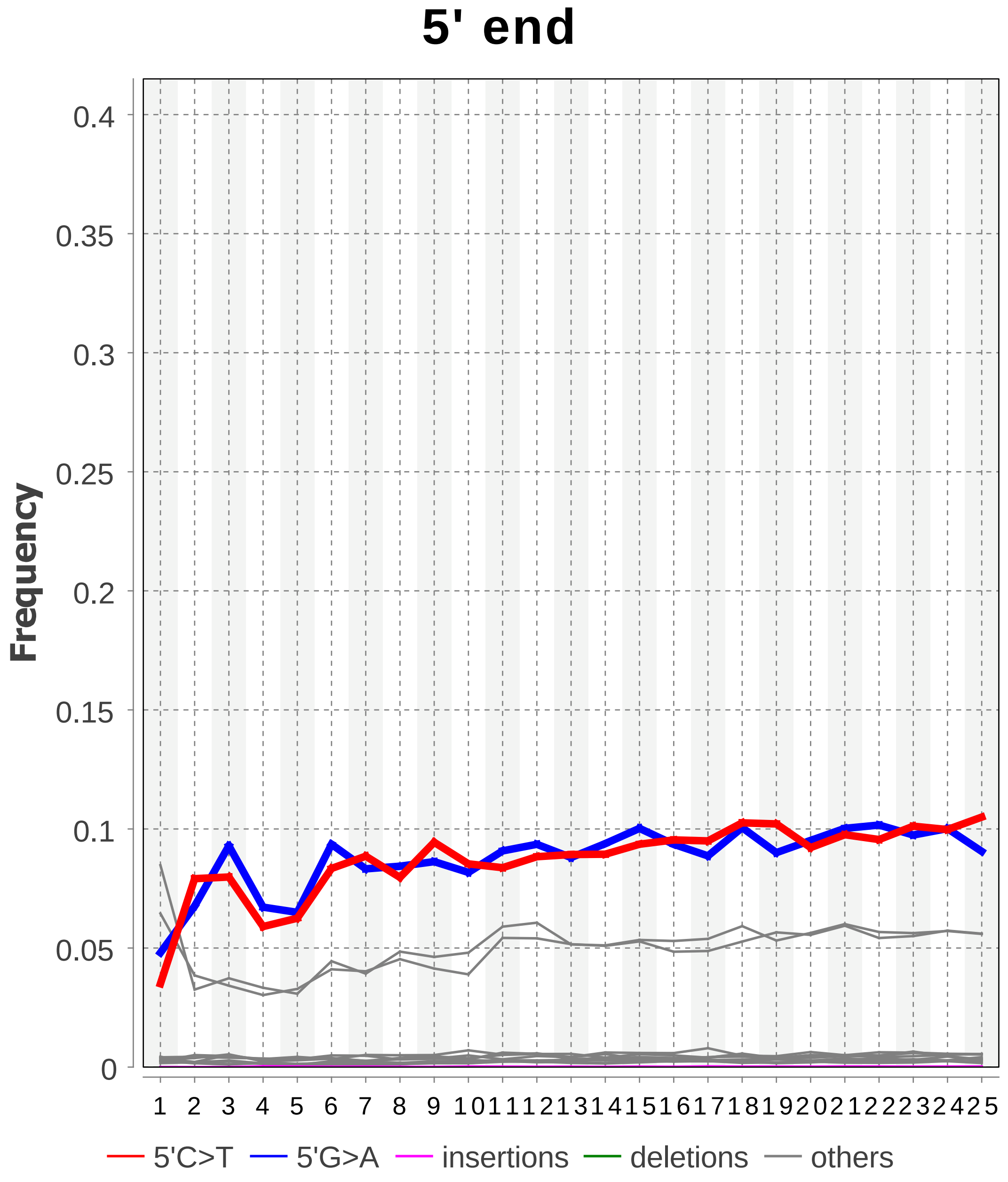

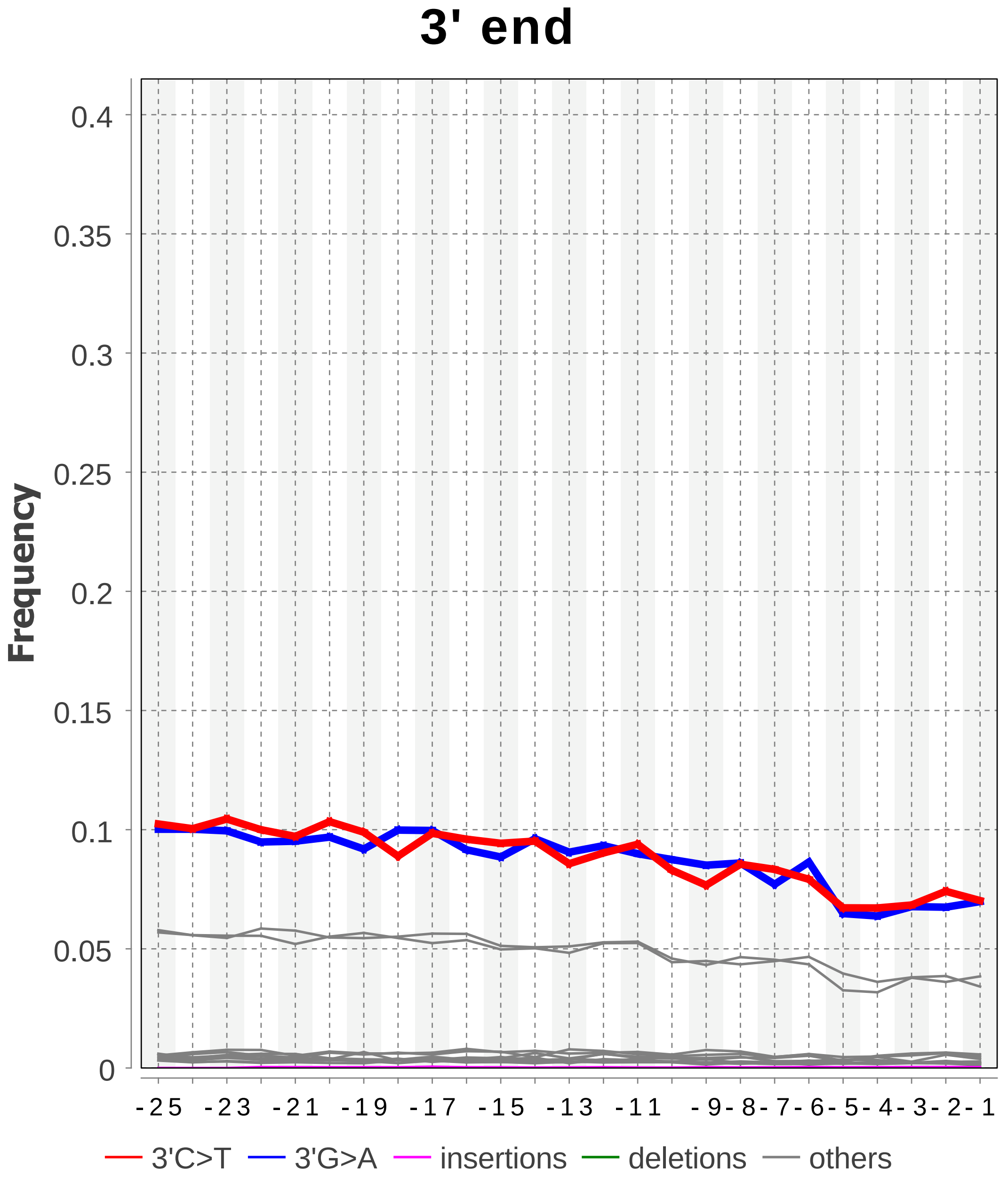

2494_aln
Number of used reads: 68,458 (100.0% of all input reads)

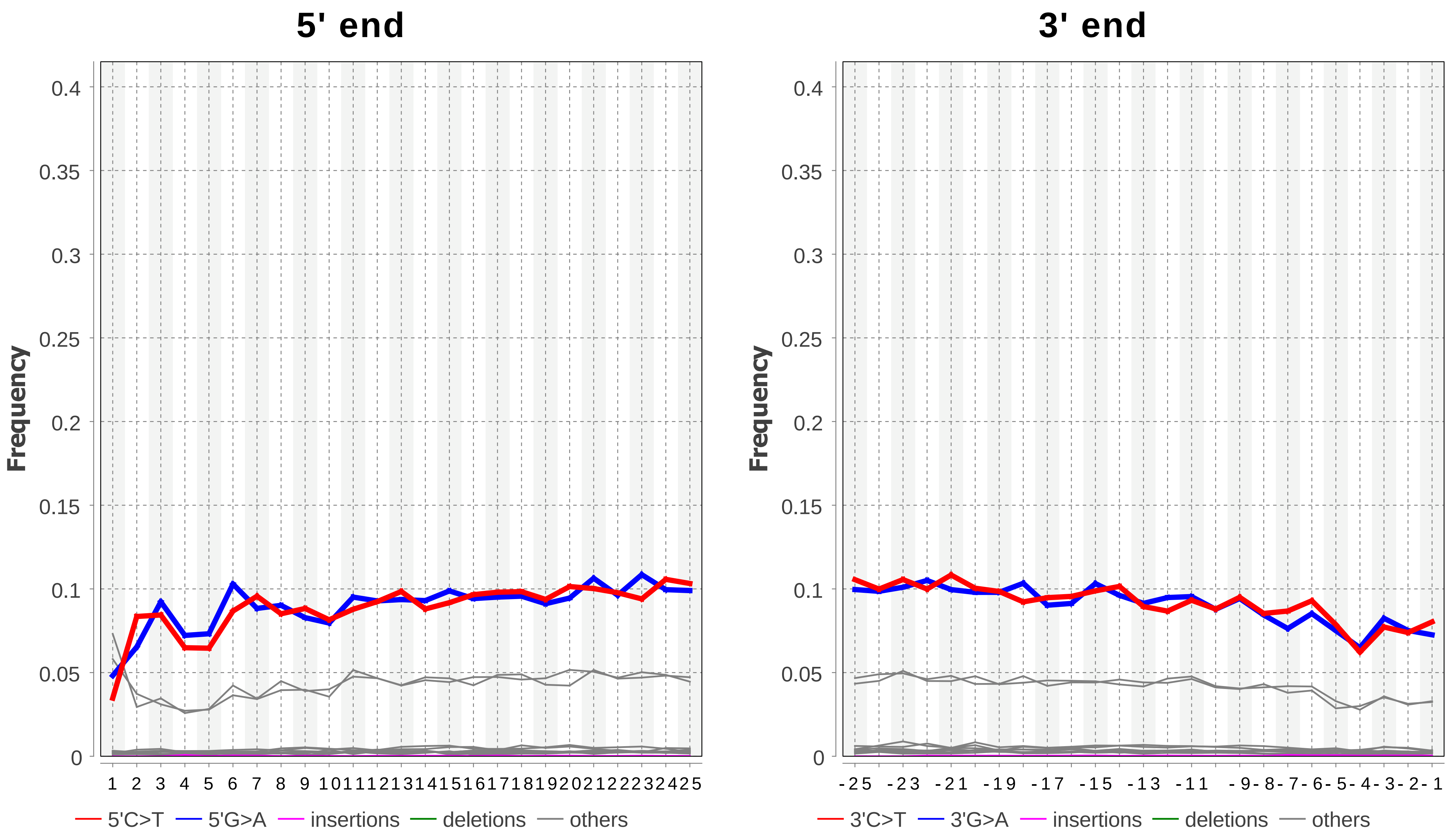

2494_MarkDuplicates
Number of used reads: 55,689 (100.0% of all input reads)

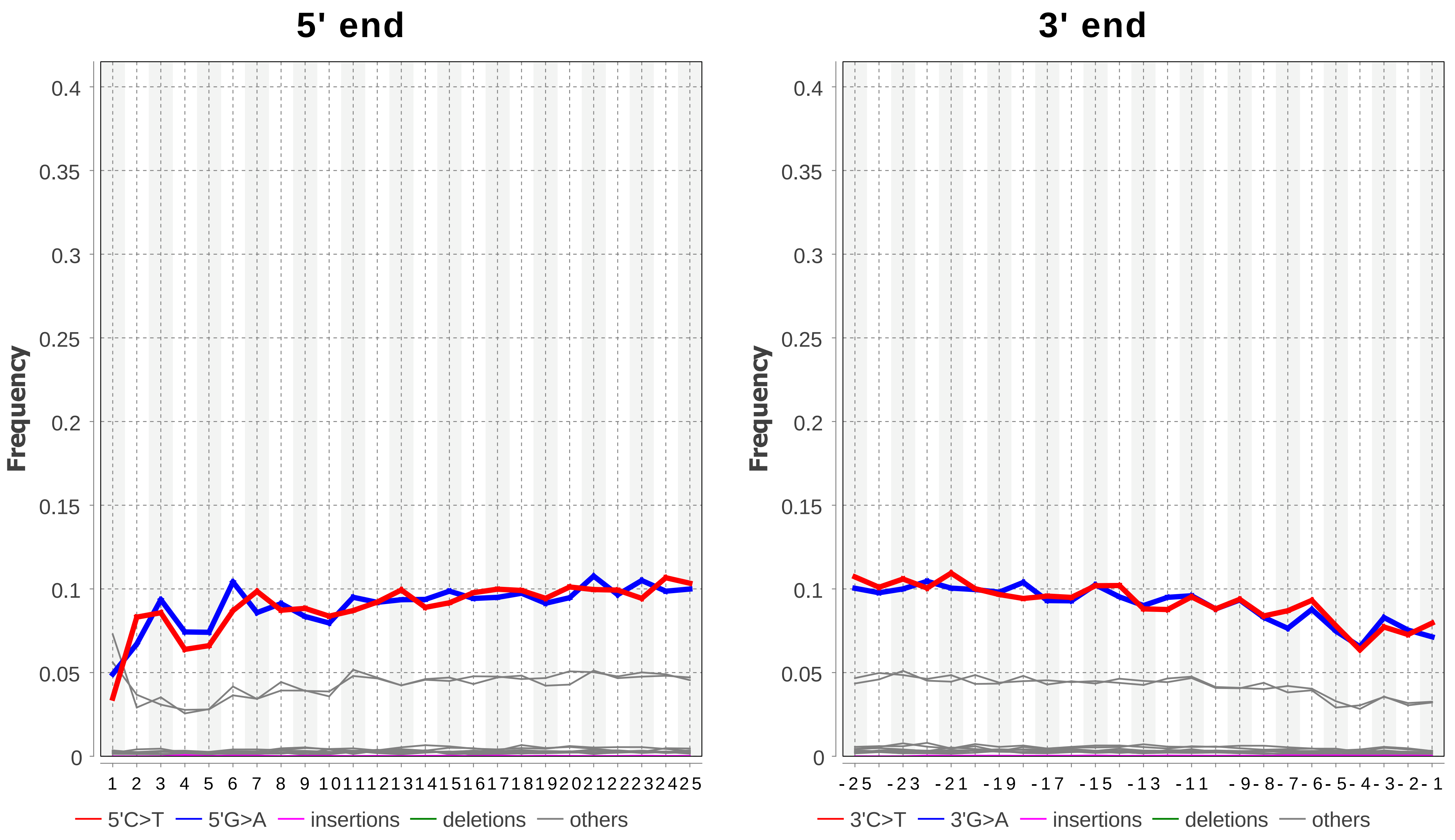

2498_aln

Number of used reads: 109,608 (100.0% of all input reads)

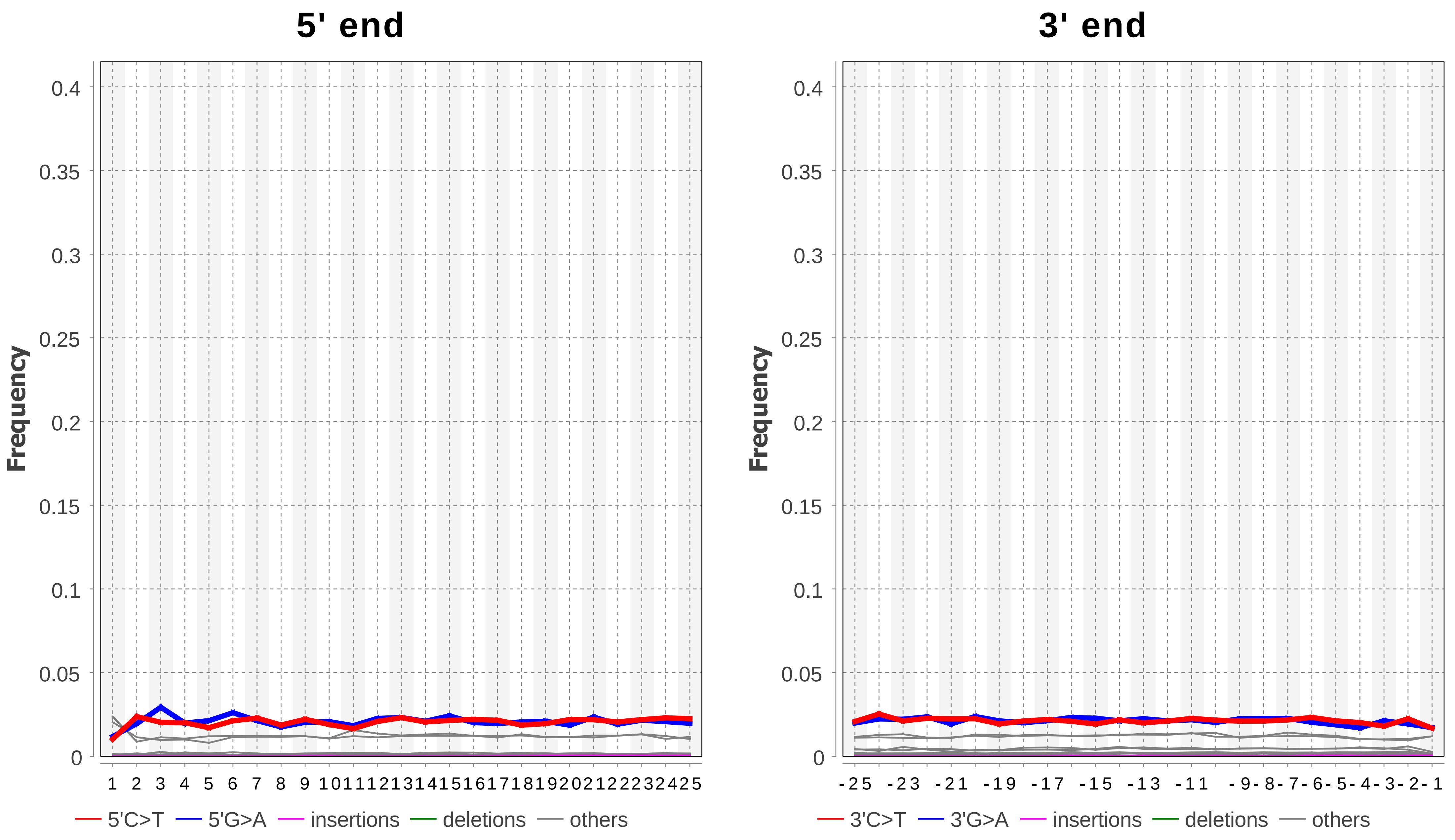

2498_MarkDuplicates
Number of used reads: 91,513 (100.0% of all input reads)

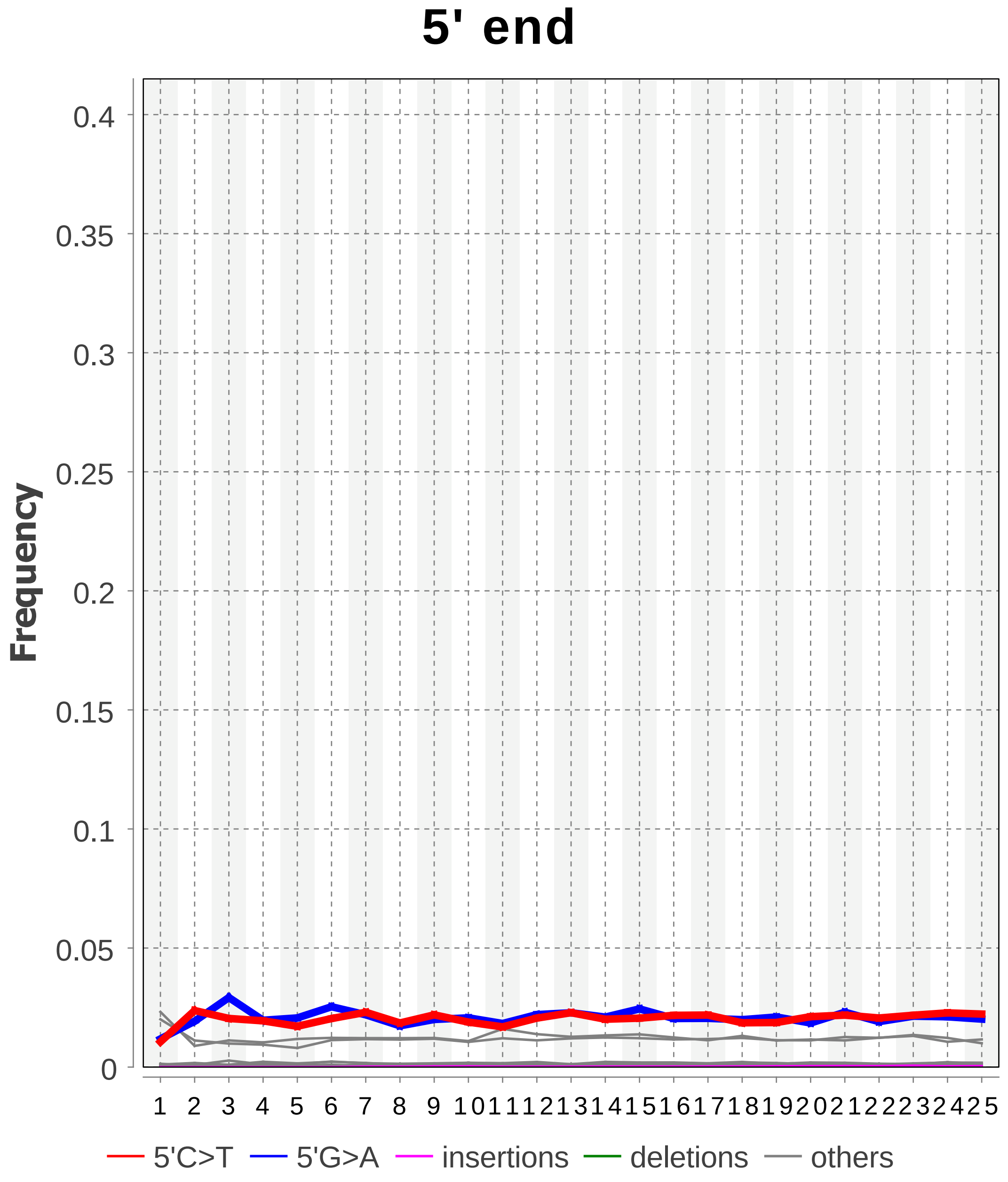

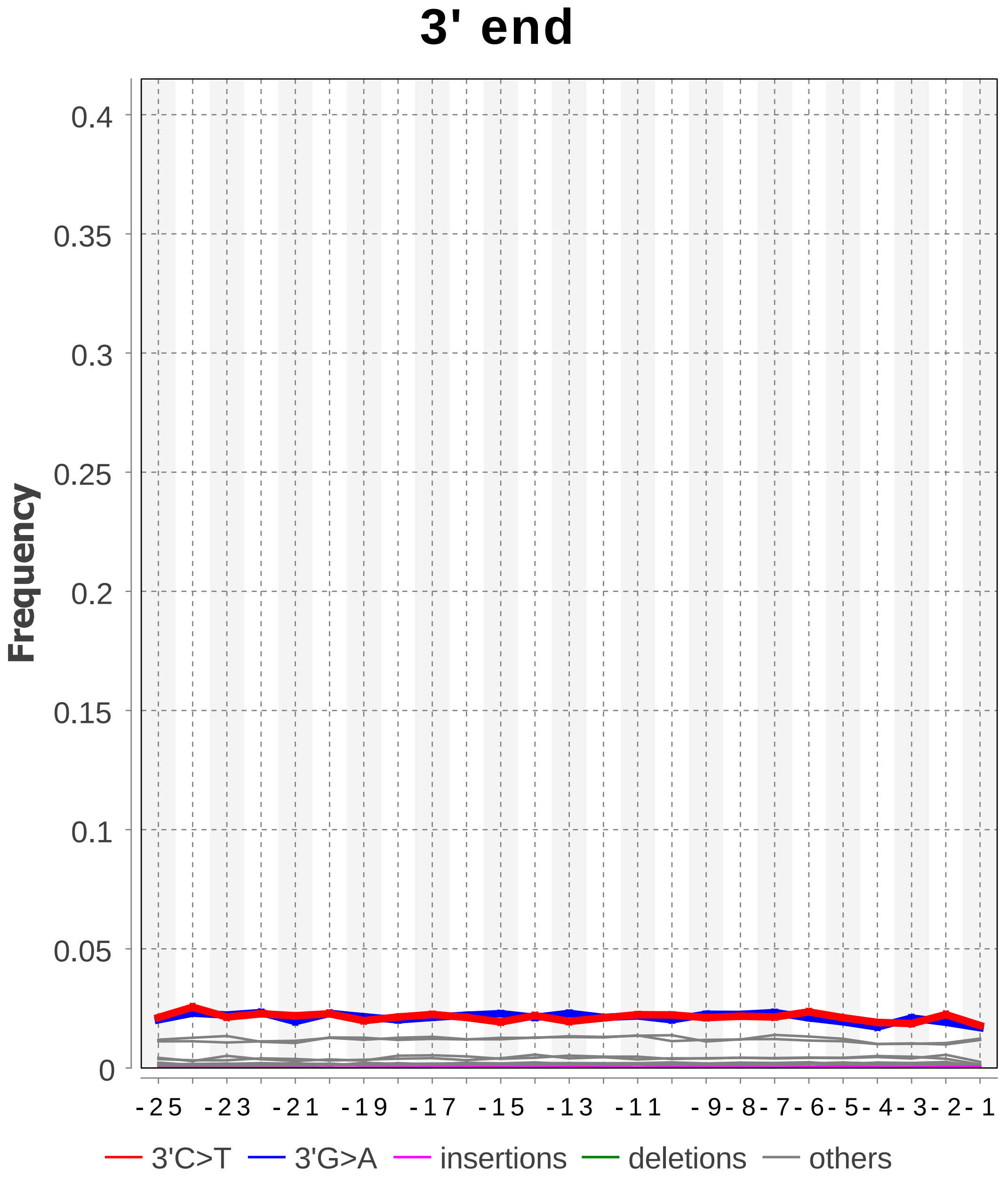

2504_aln

Number of used reads: 186,029 (100.0% of all input reads)

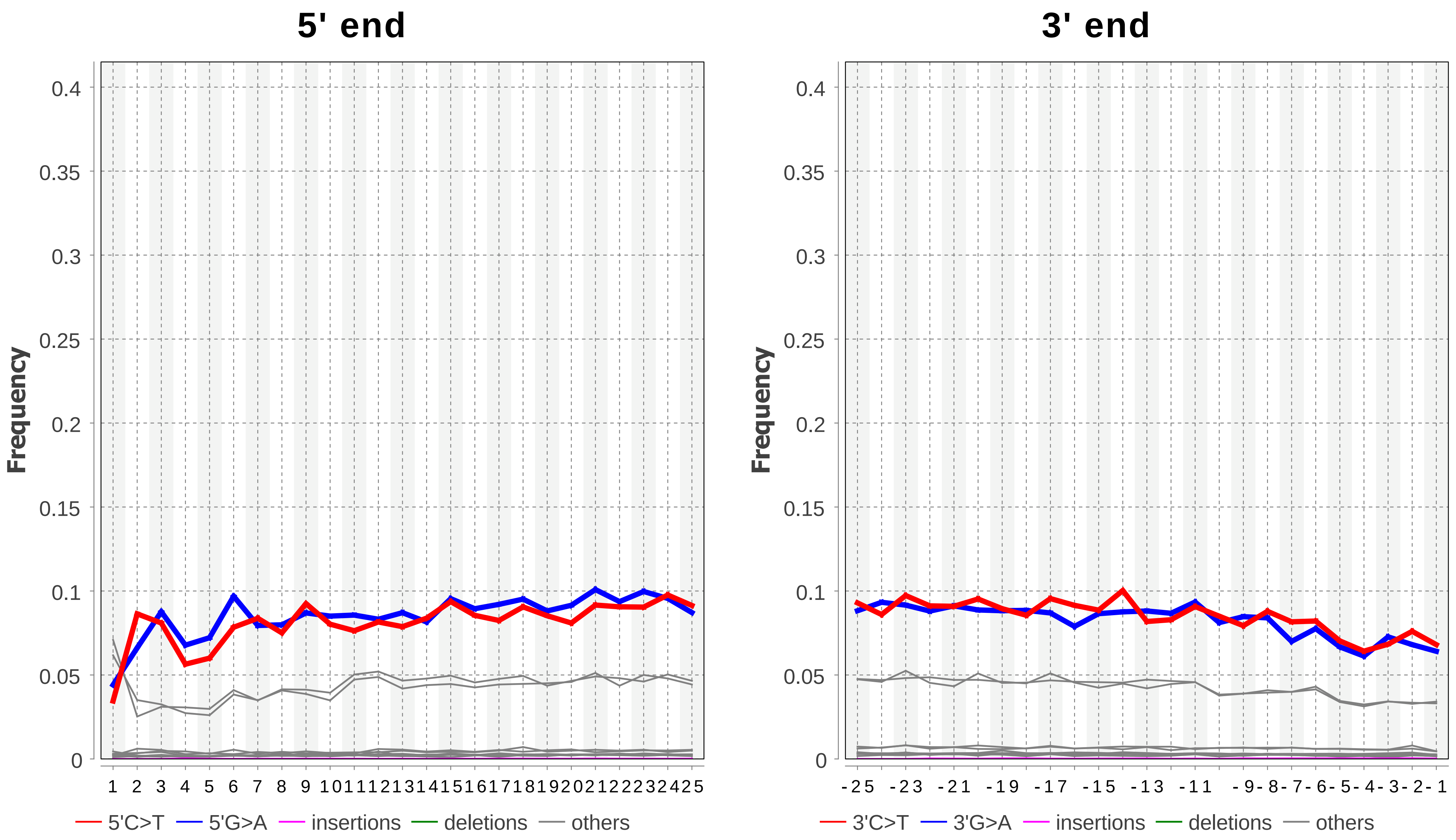

2504_MarkDuplicates

Number of used reads: 152,329 (100.0% of all input reads)

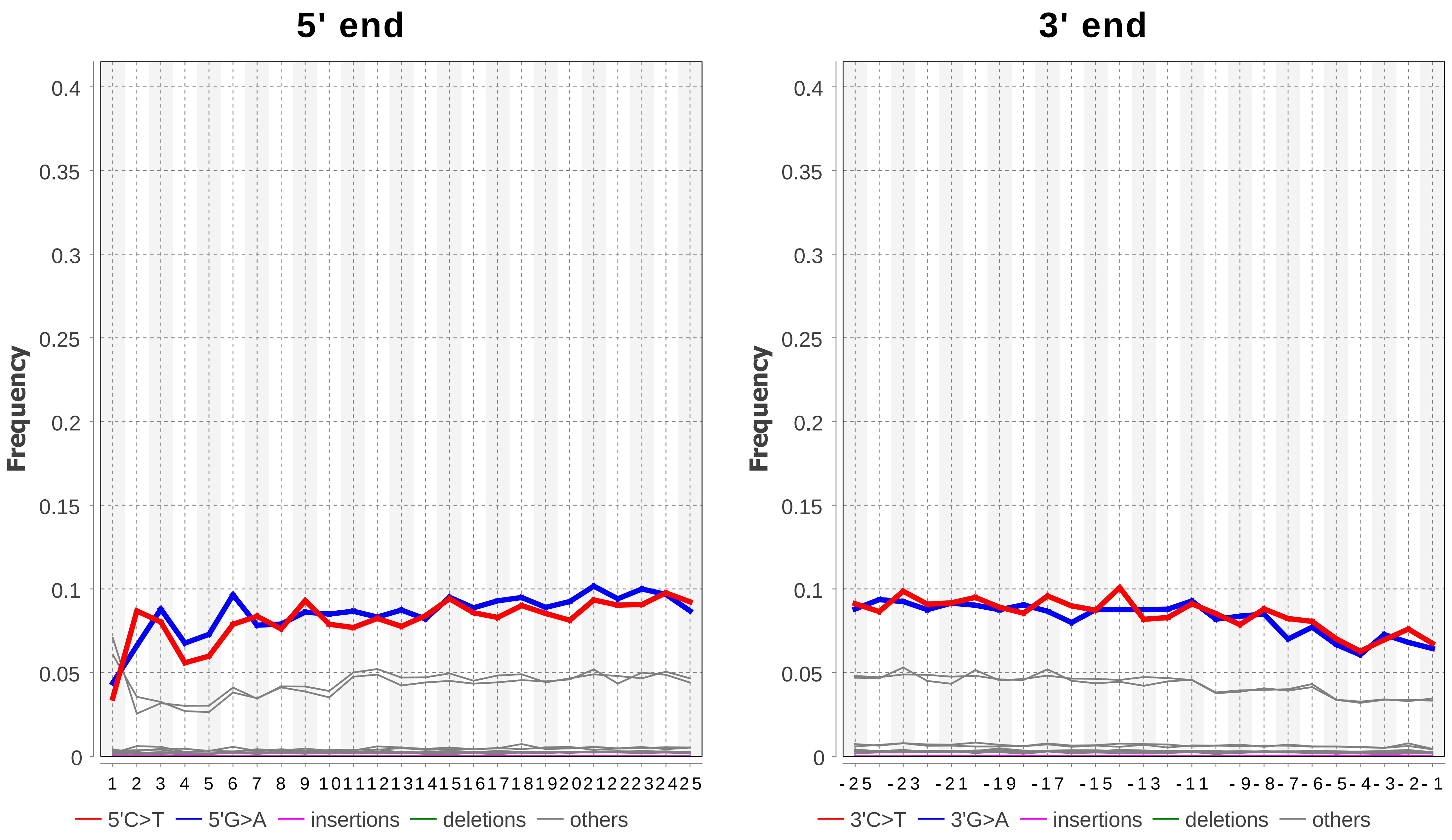

2579_aln
Number of used reads: 259,095 (100.0% of all input reads)

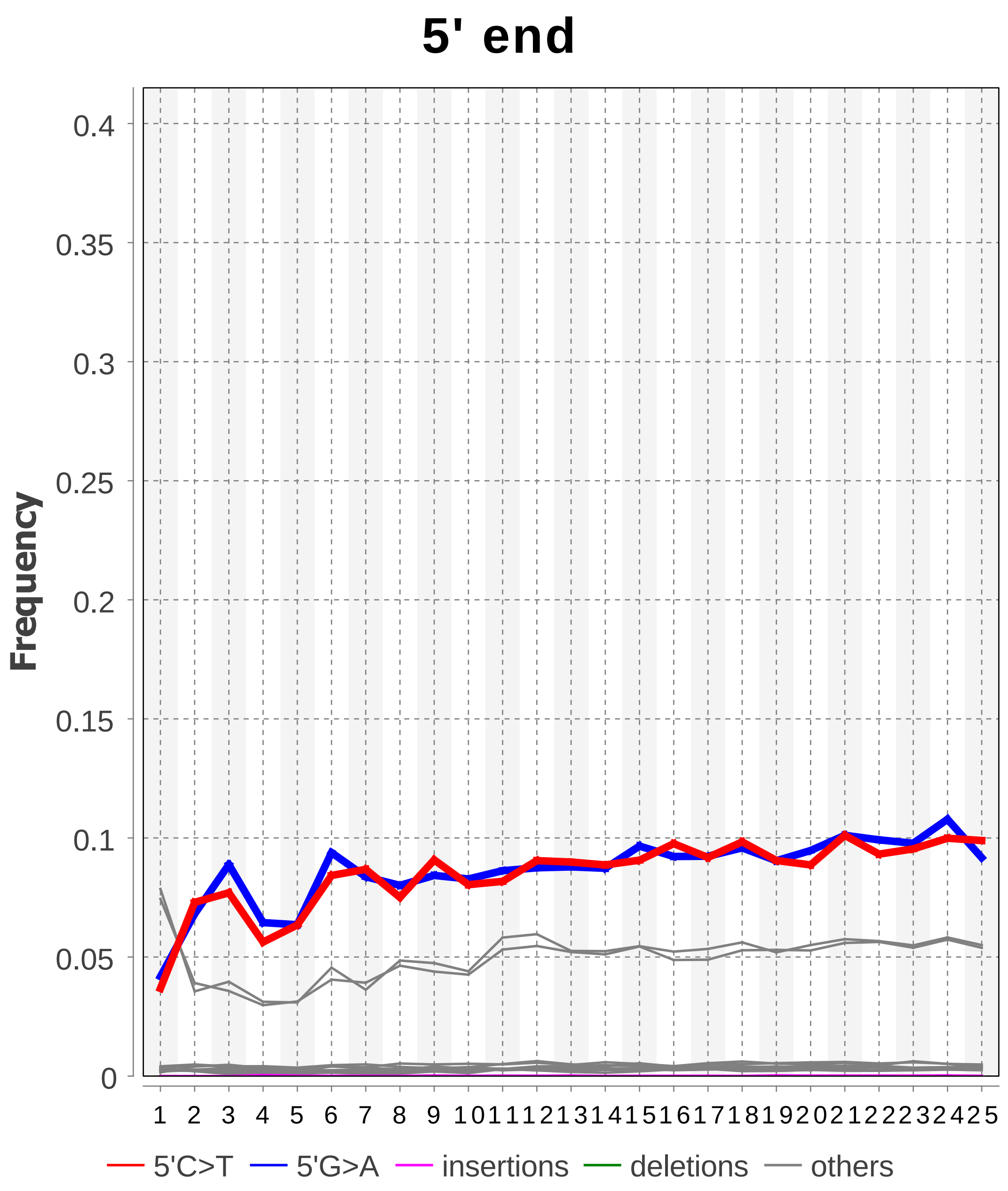

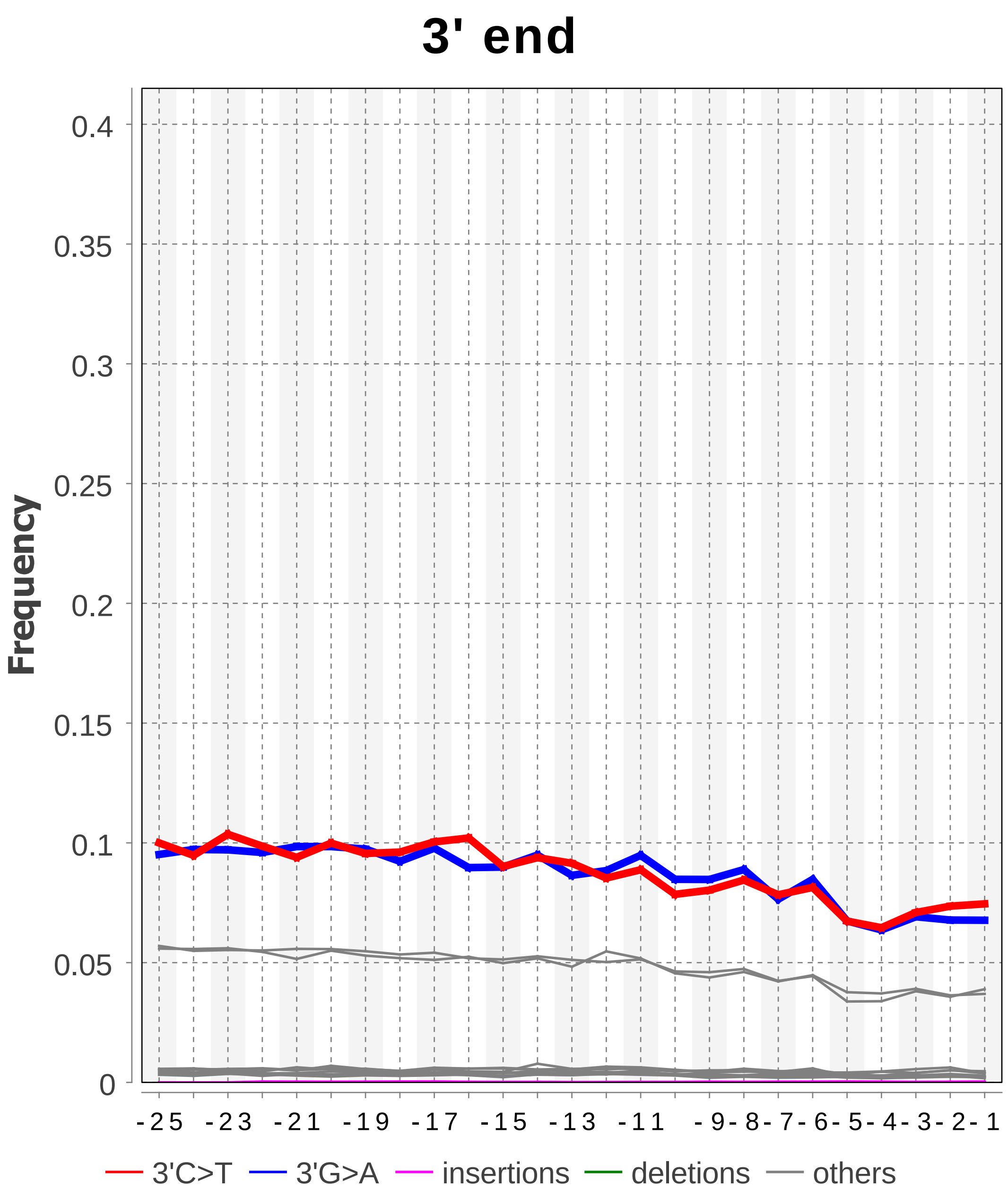

2579_MarkDuplicates
Number of used reads: 212,980 (100.0% of all input reads)

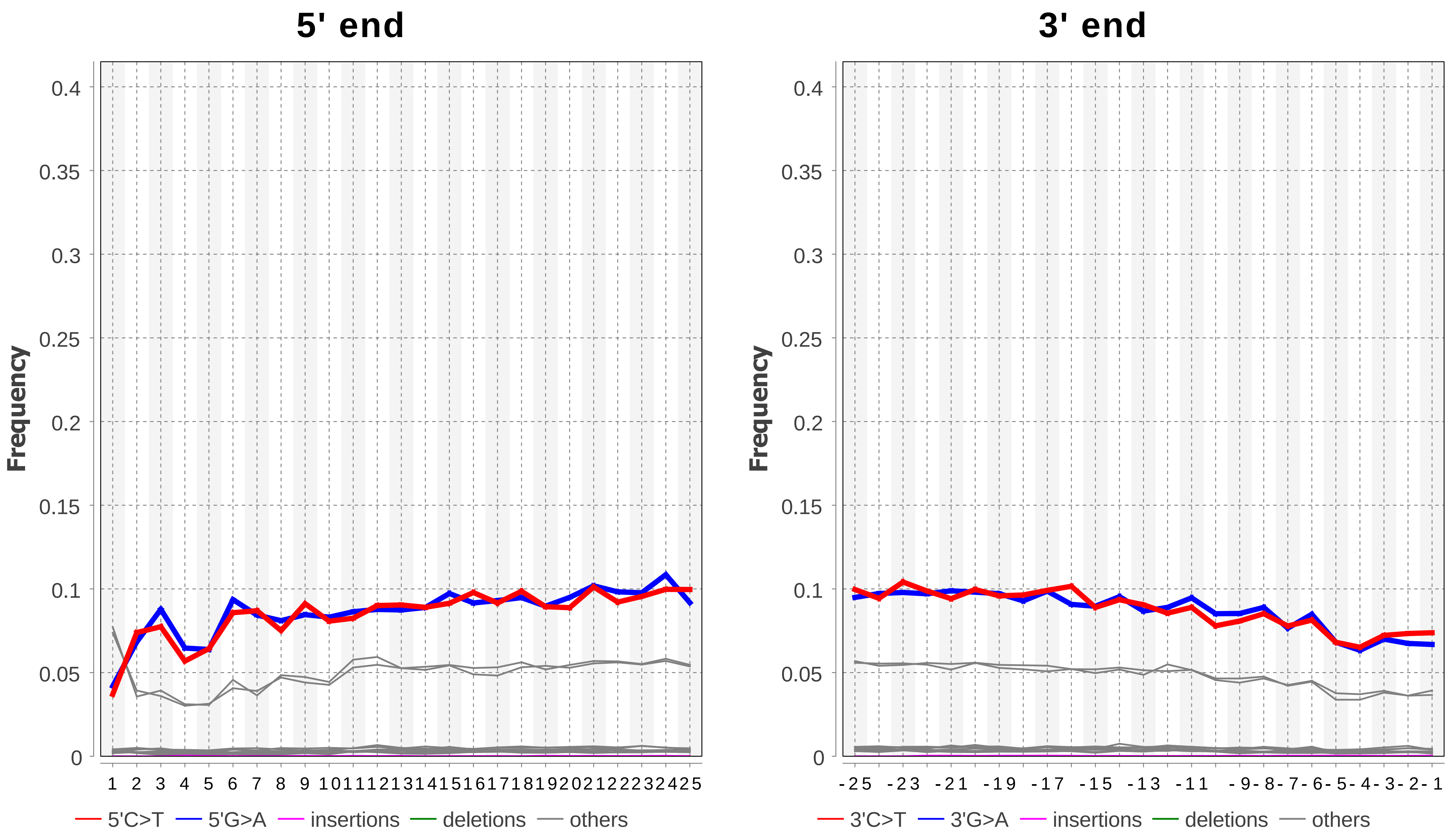

2580_aln

Number of used reads: 370,333 (100.0% of all input reads)

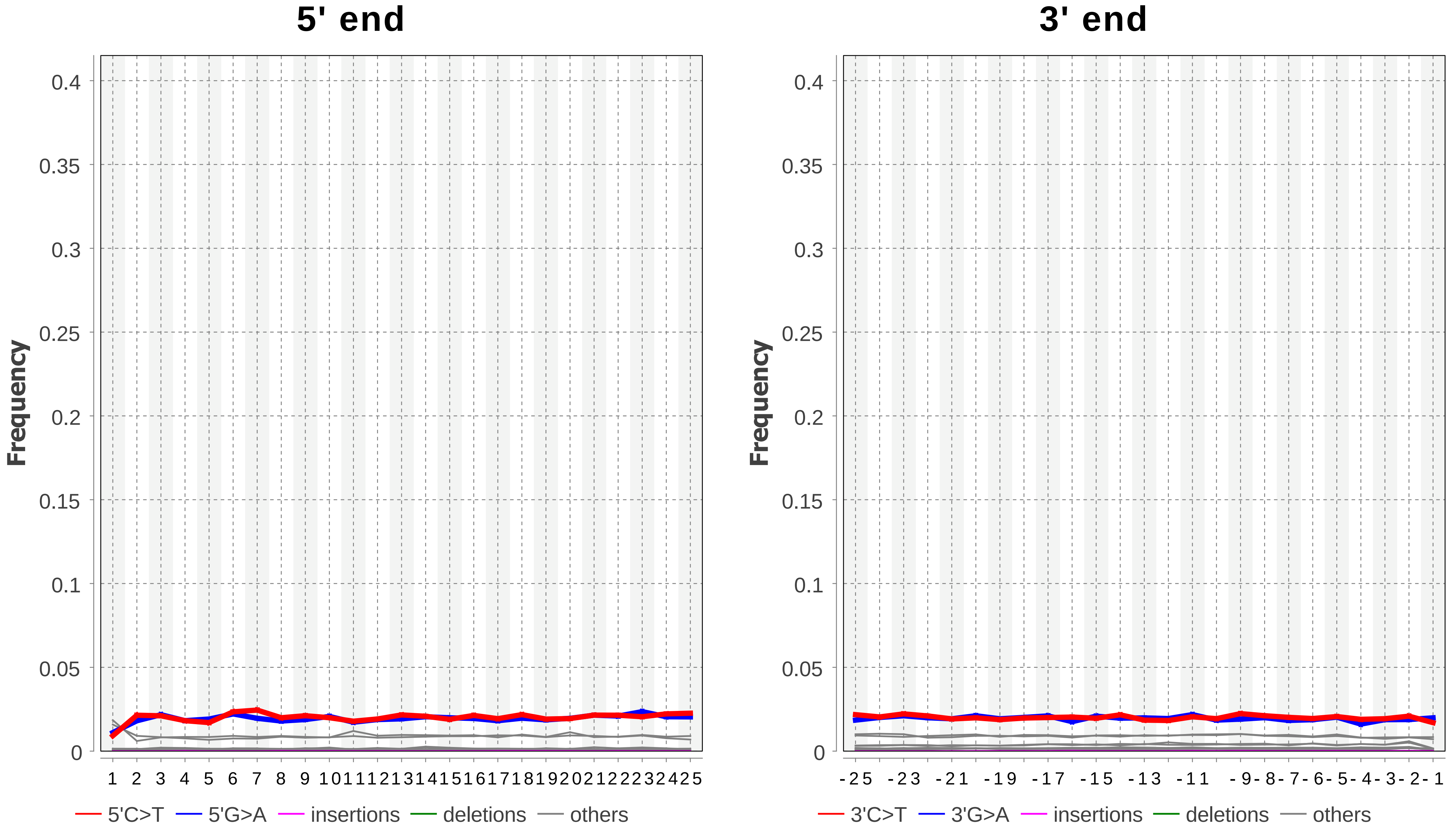

2580_MarkDuplicates
Number of used reads: 308,234 (100.0% of all input reads)

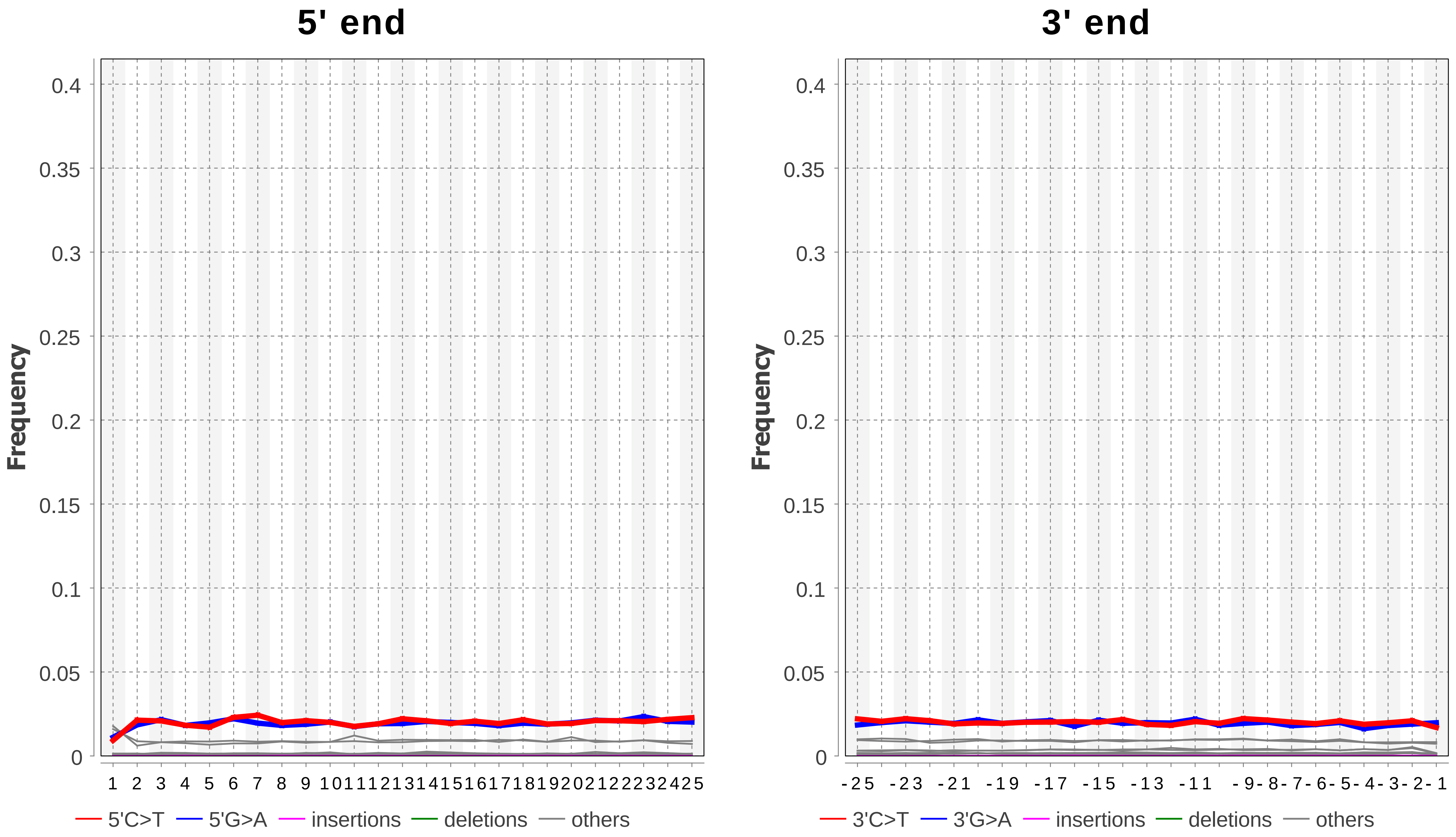

2584_aln
Number of used reads: 172,824 (100.0% of all input reads)

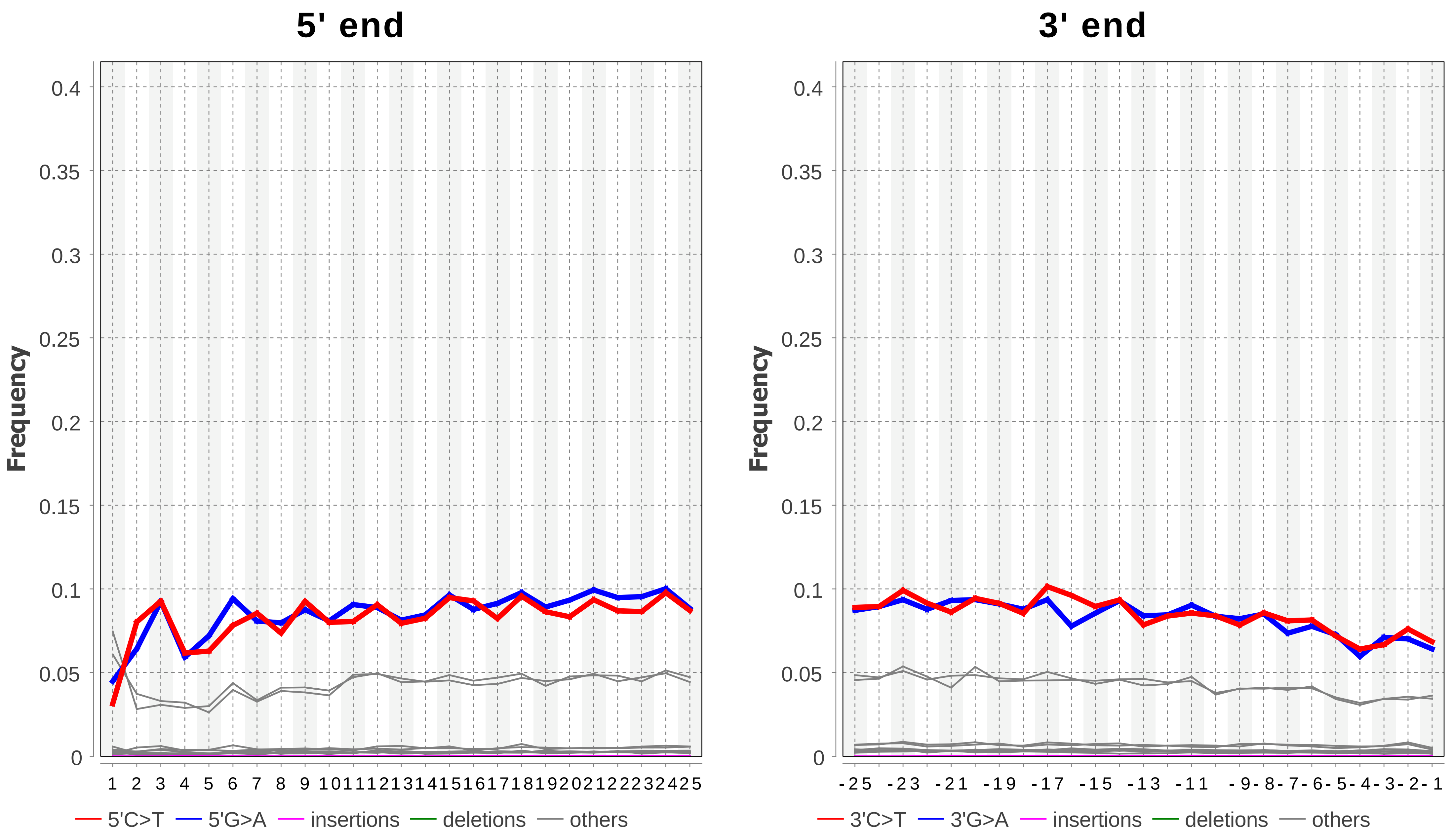

2584_MarkDuplicates
Number of used reads: 141,952 (100.0% of all input reads)

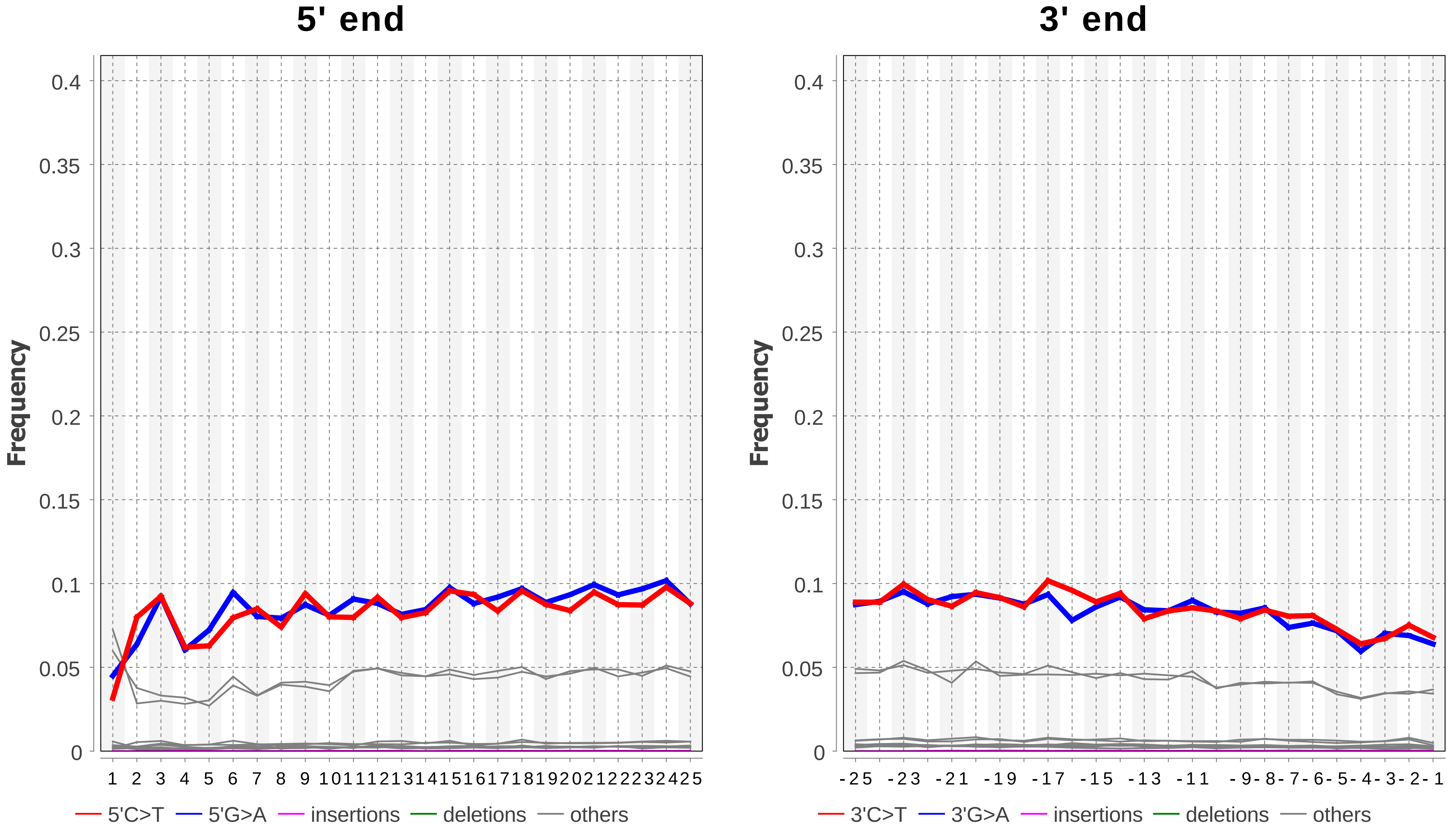

2702_aln
Number of used reads: 265,633 (100.0% of all input reads)

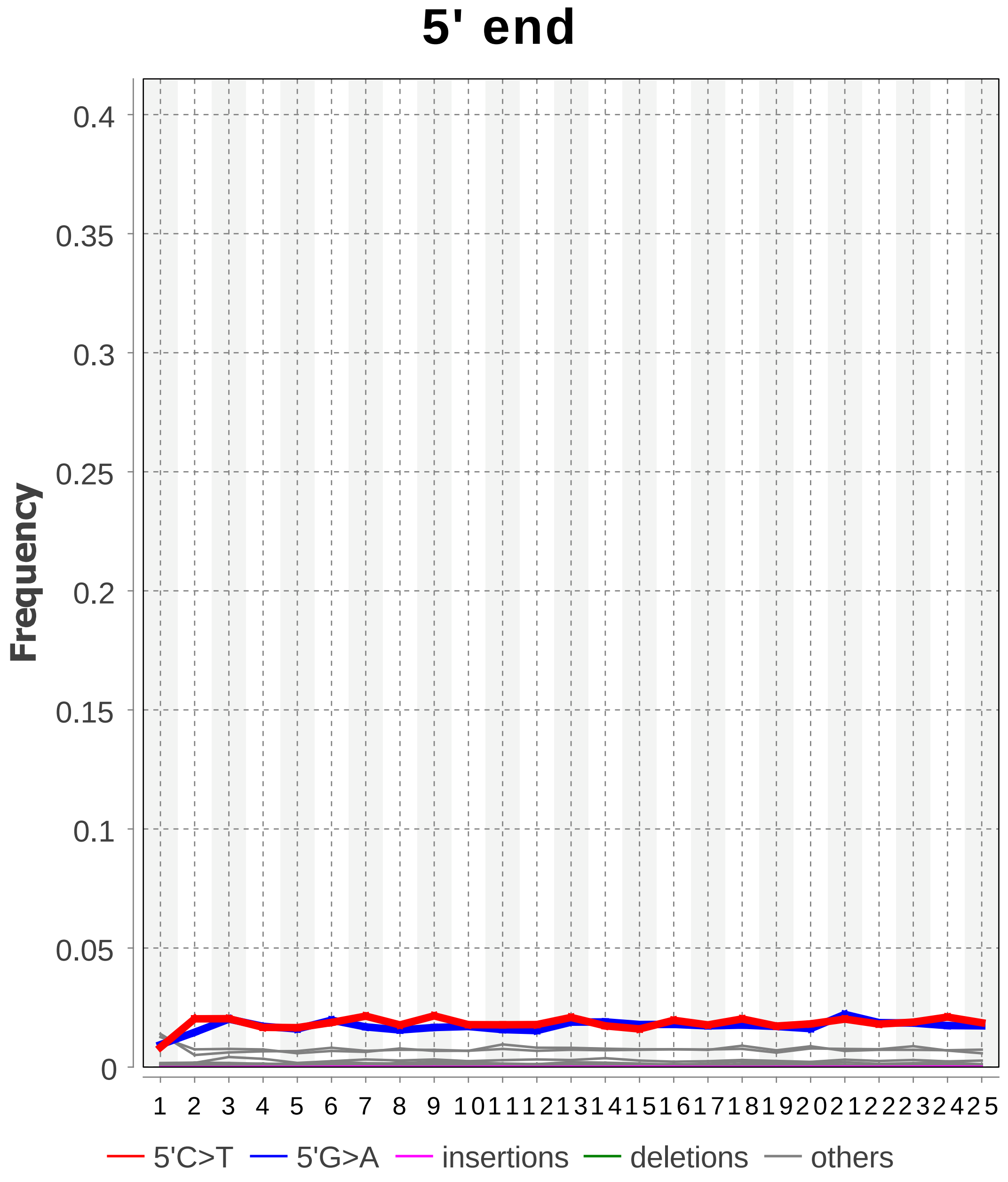

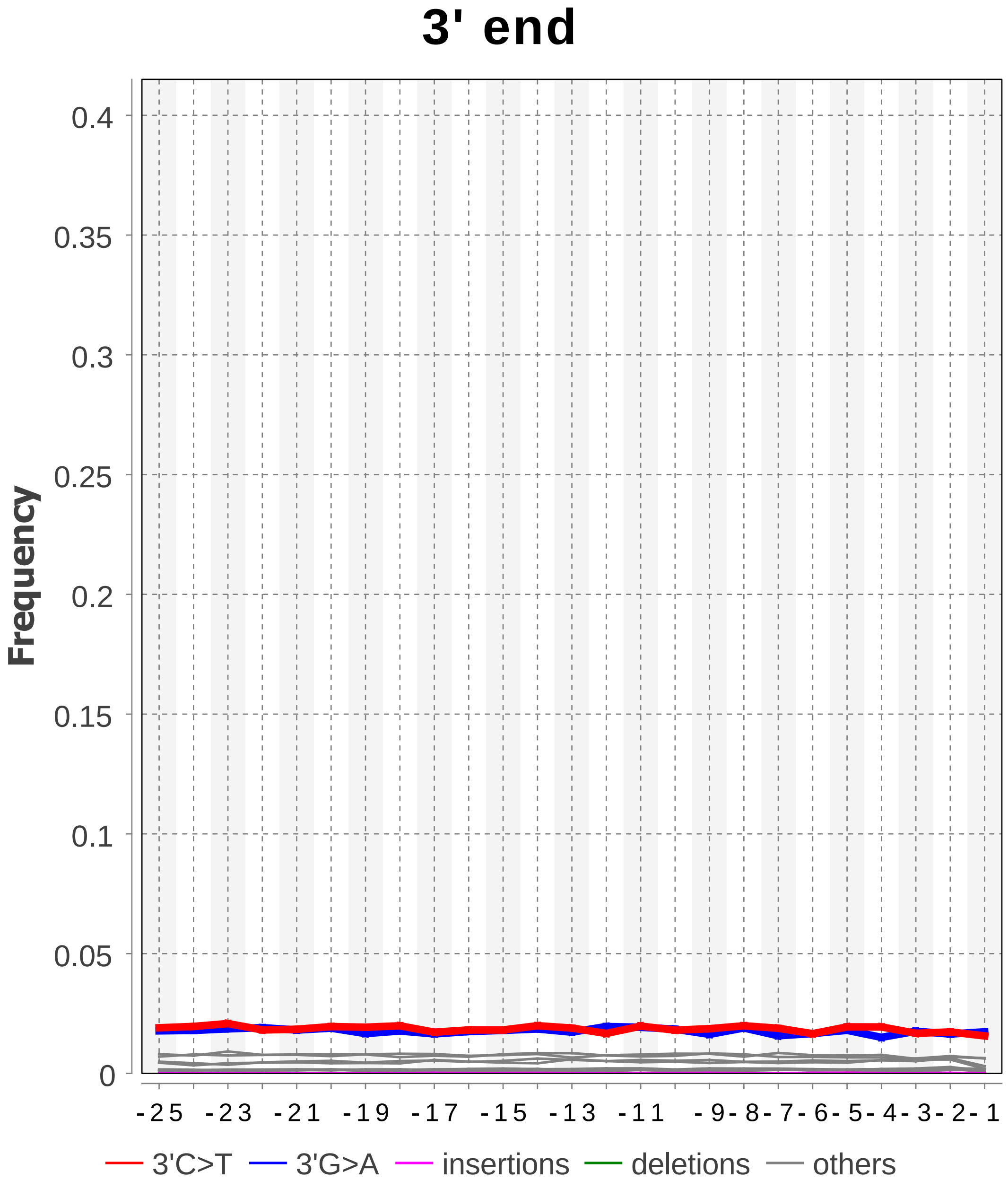

2702_MarkDuplicates

Number of used reads: 201,726 (100.0% of all input reads)

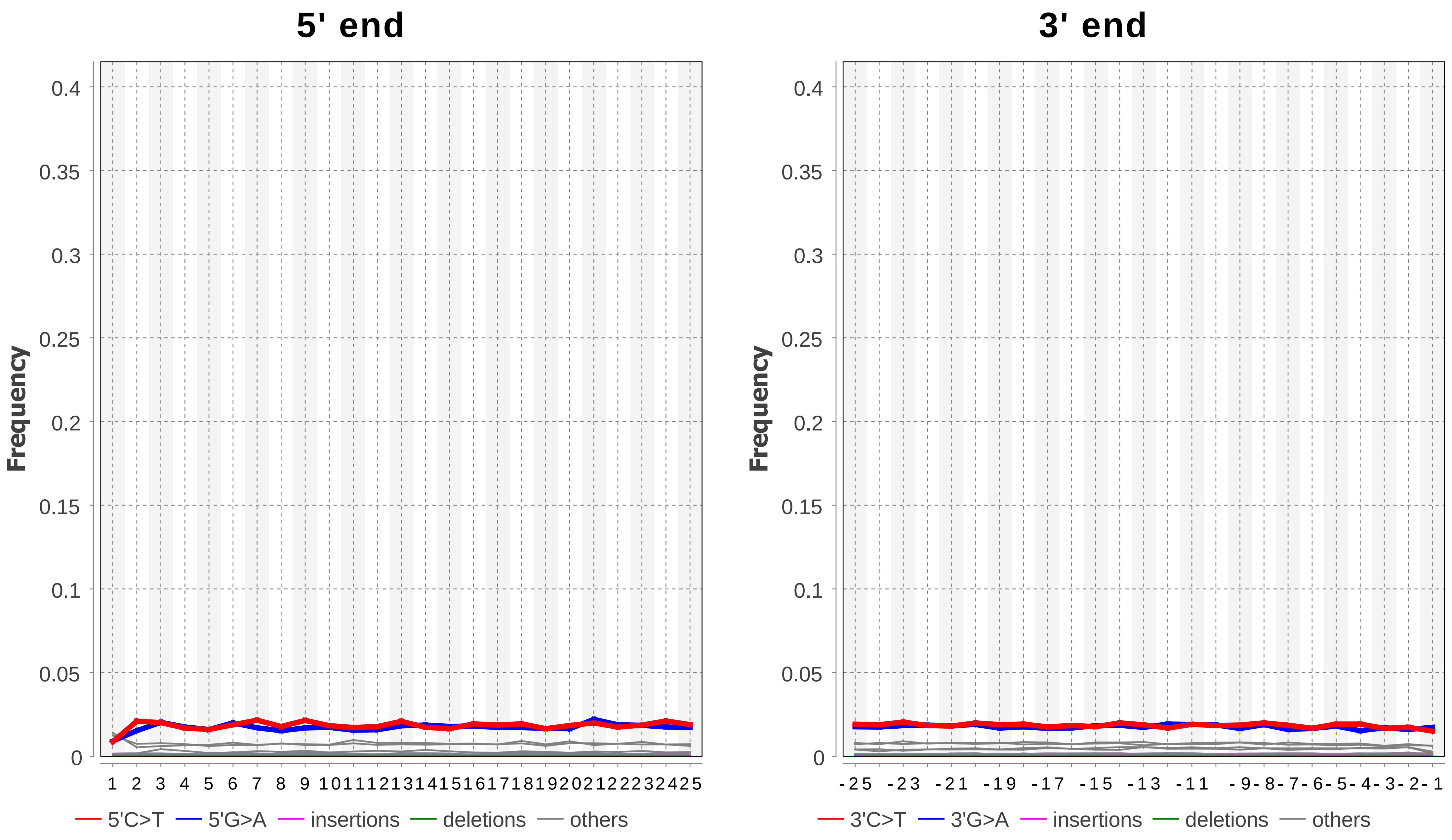

2710_aln

Number of used reads: 354,307 (100.0% of all input reads)

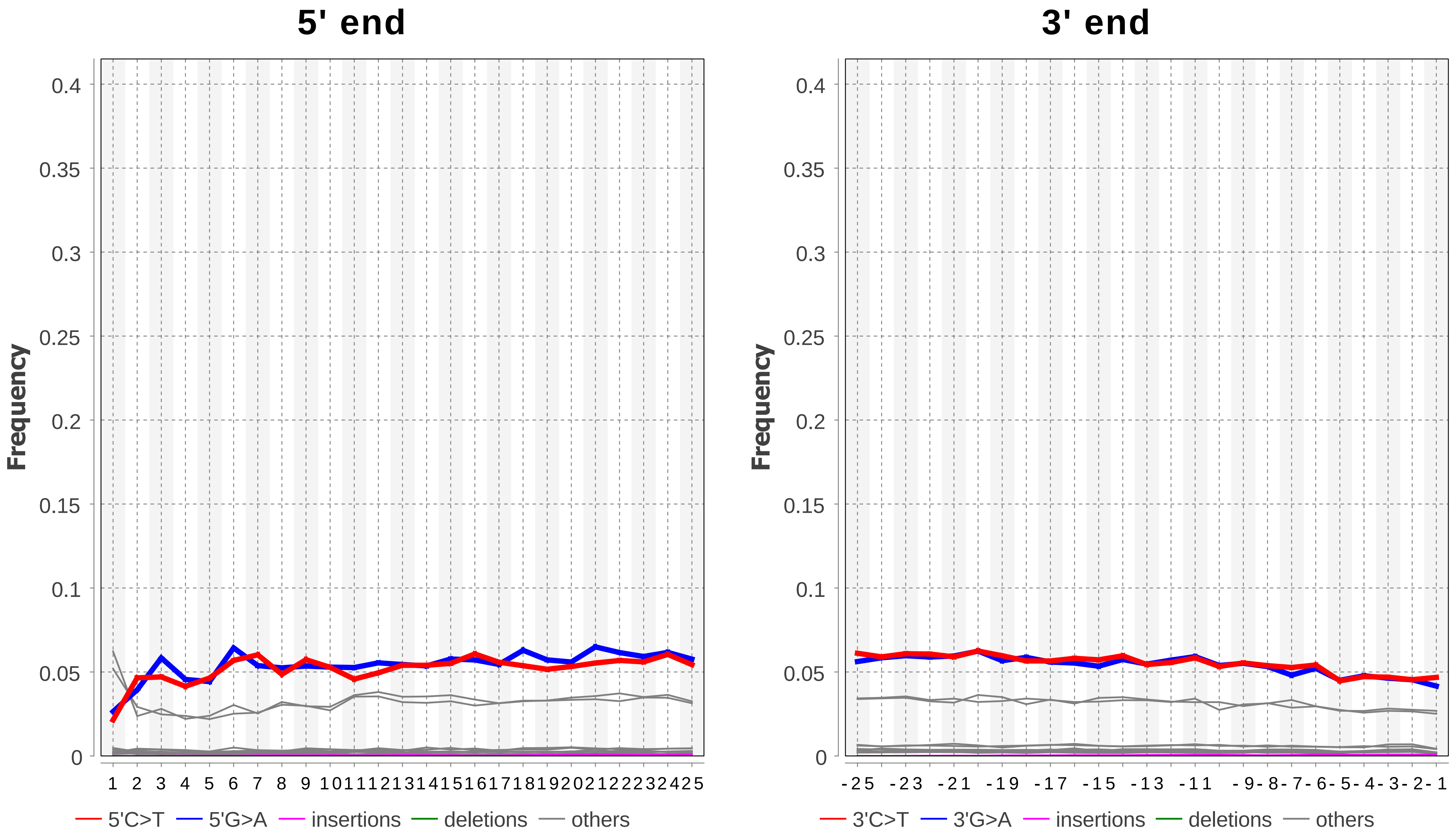

2710_MarkDuplicates
Number of used reads: 290,562 (100.0% of all input reads)

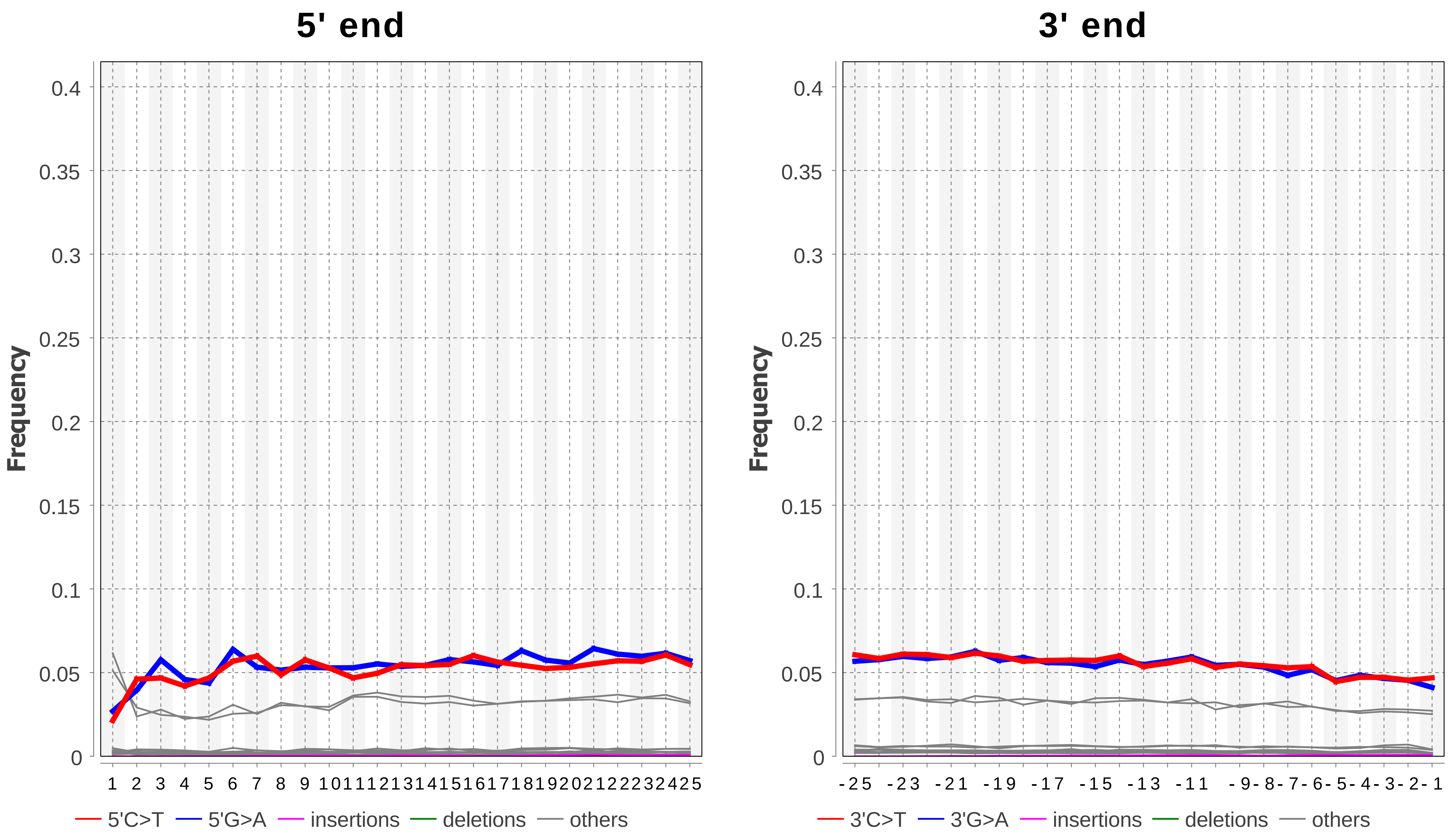

2714_aln

Number of used reads: 68,329 (100.0% of all input reads)

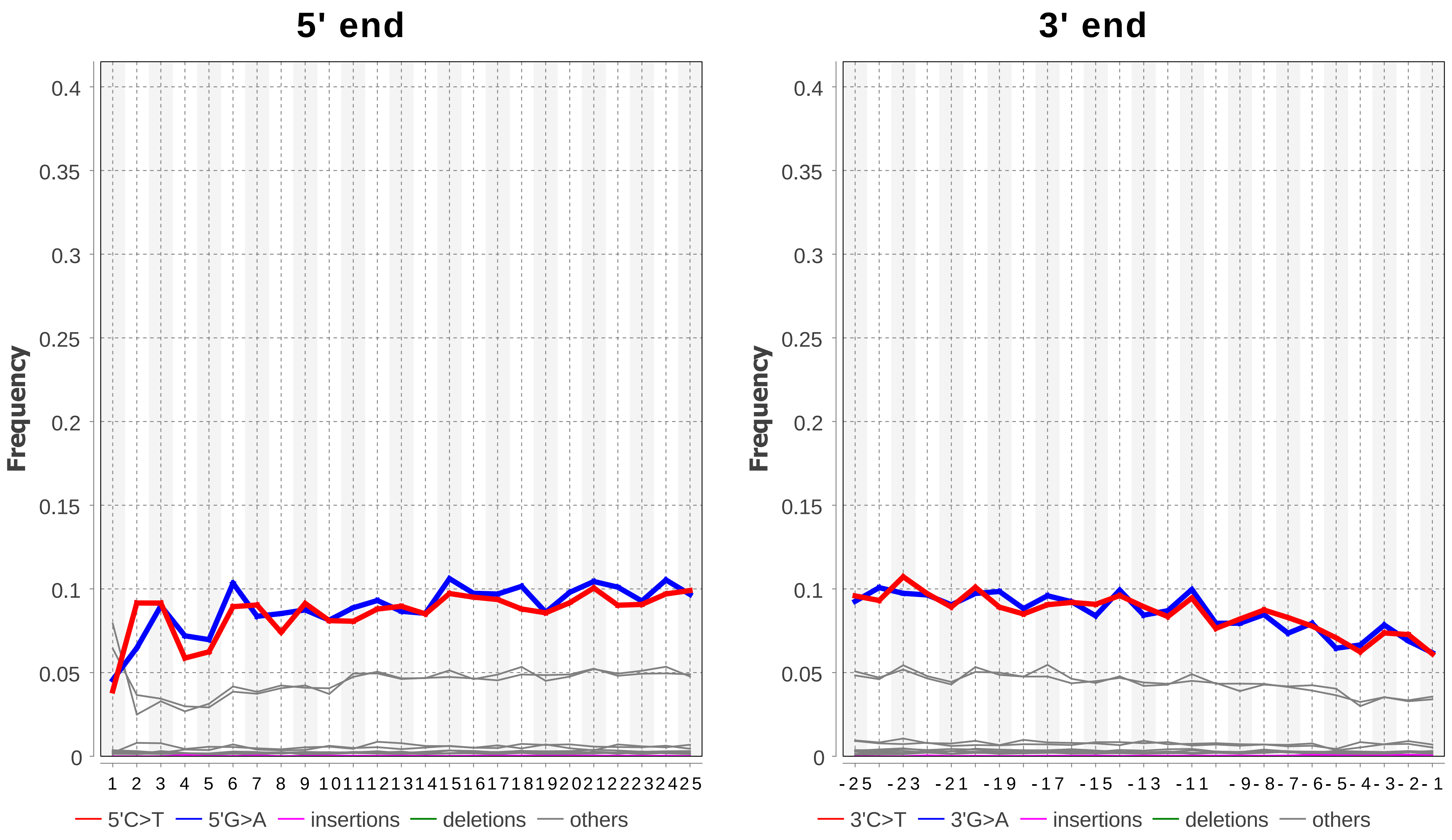

2714_MarkDuplicates

Number of used reads: 53,299 (100.0% of all input reads)

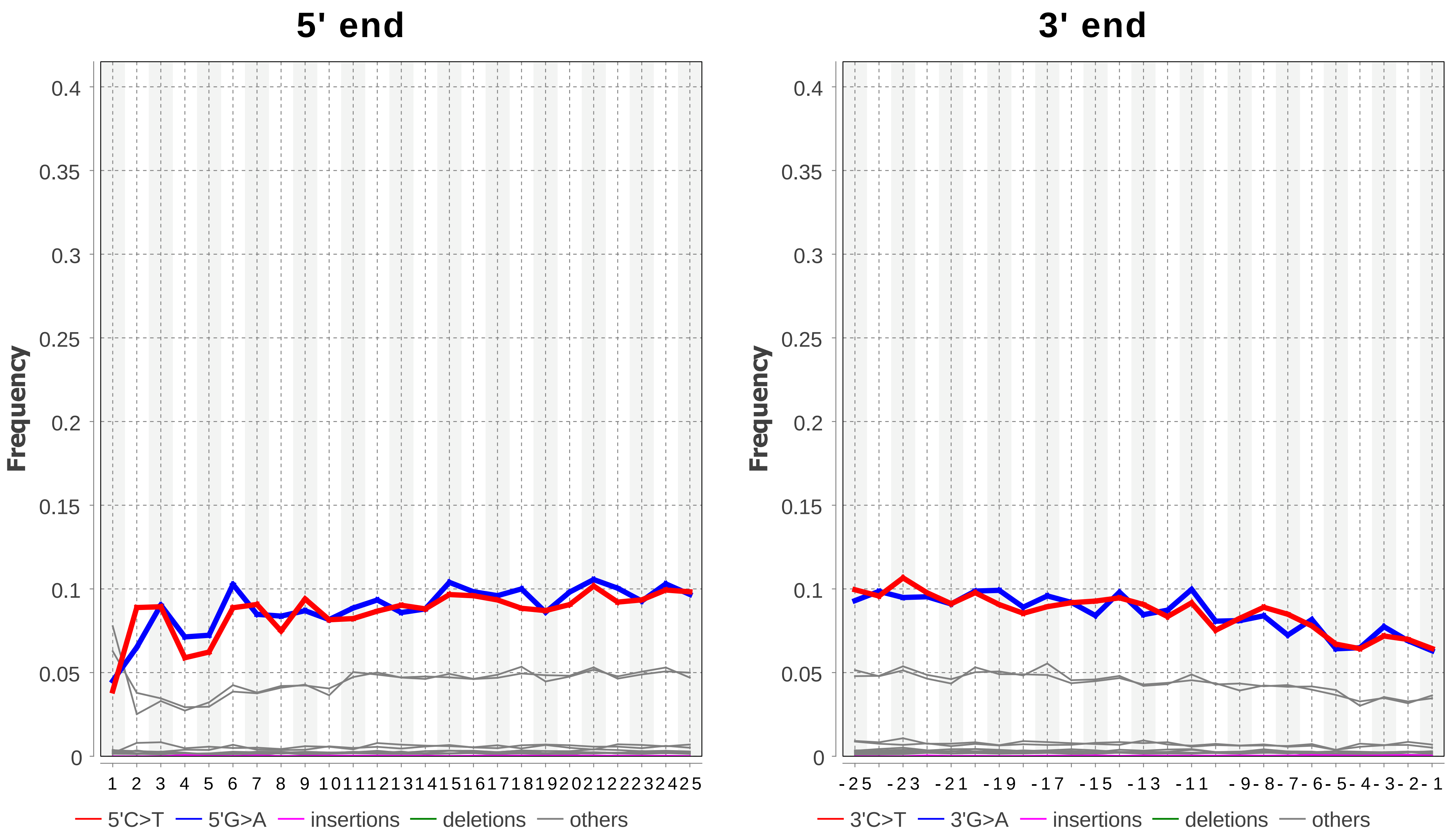

2716_aln

Number of used reads: 133,393 (100.0% of all input reads)

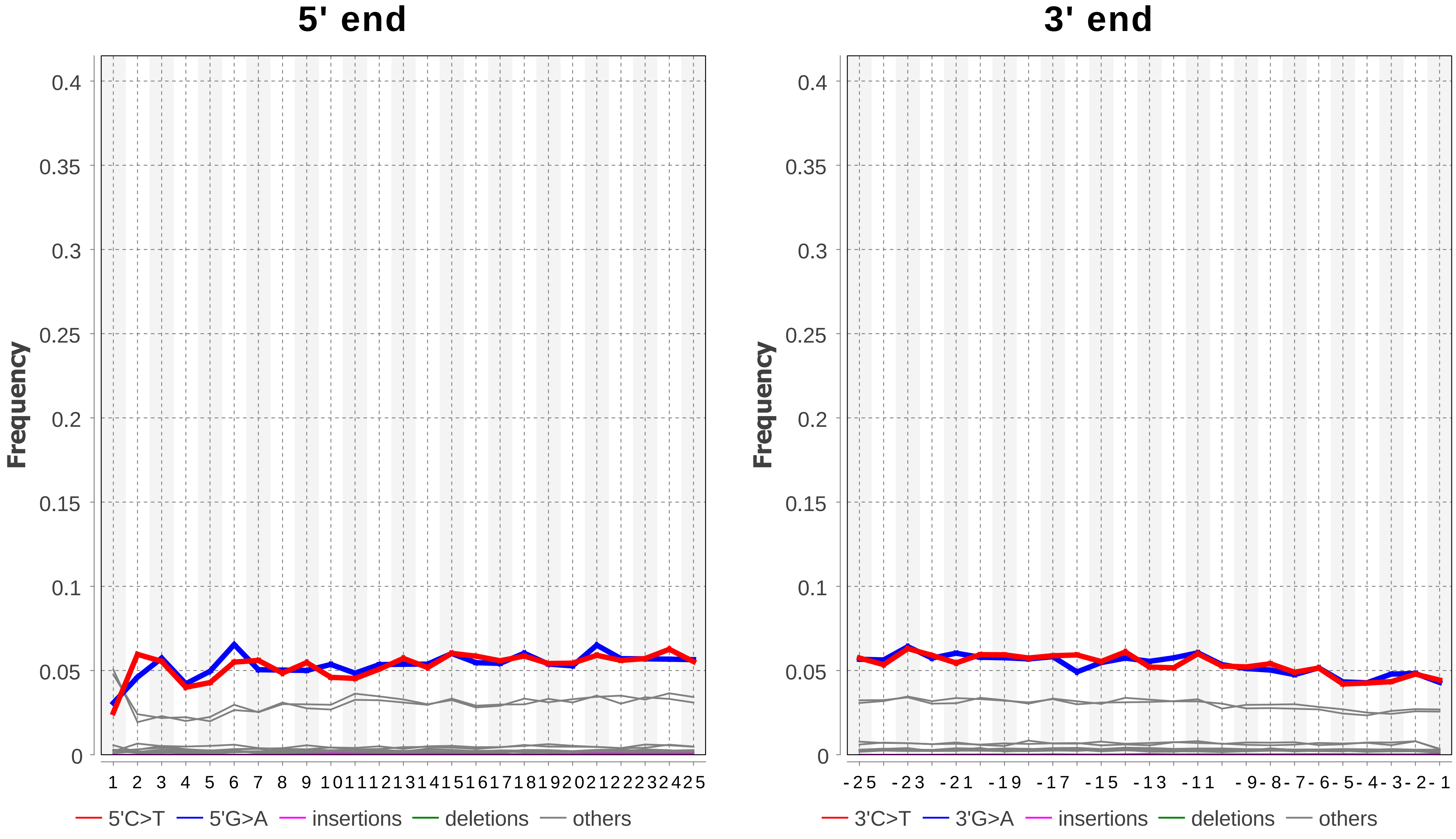

2716_MarkDuplicates
Number of used reads: 110,972 (100.0% of all input reads)

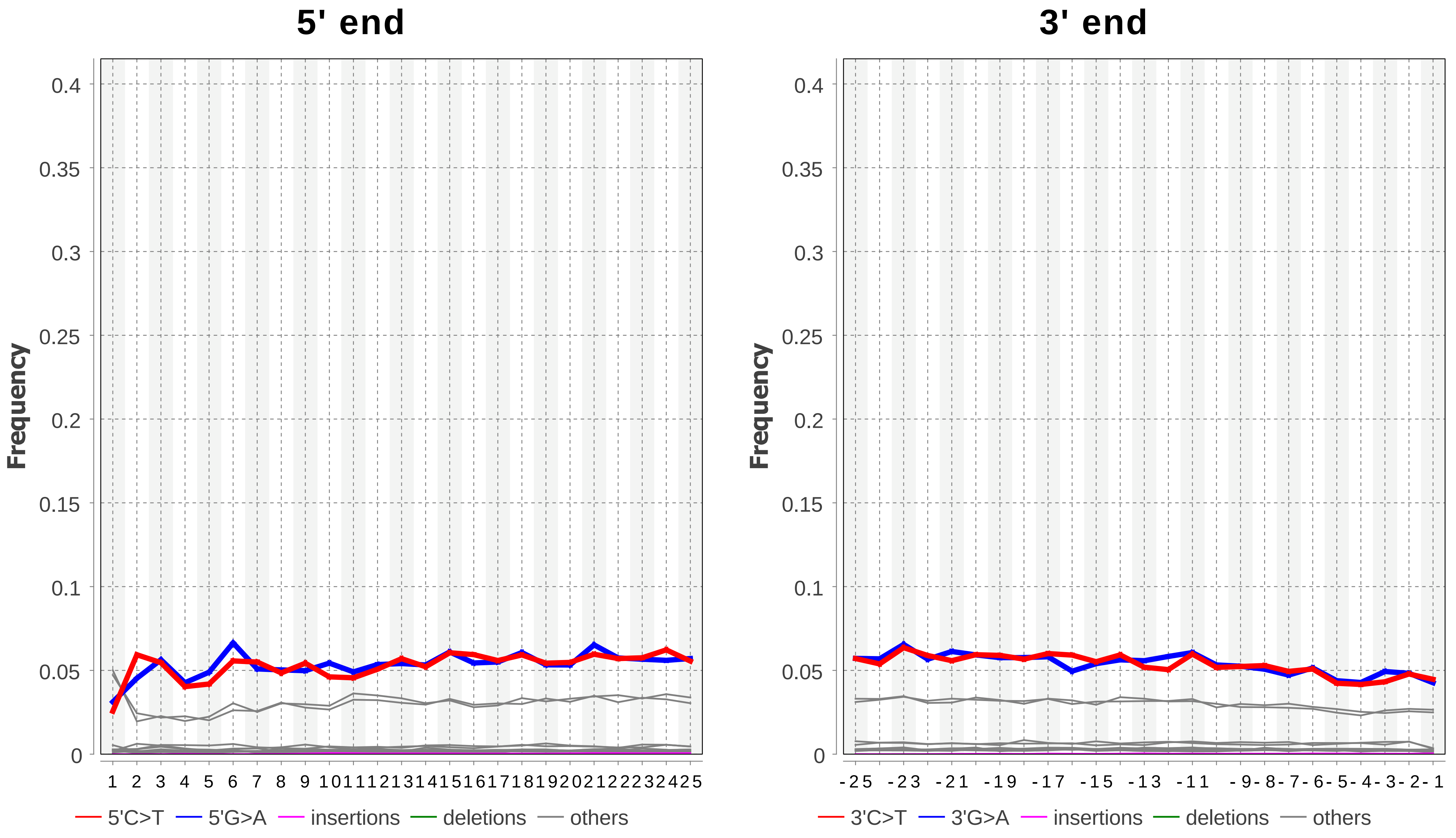

2757_aln

Number of used reads: 60,733 (100.0% of all input reads)

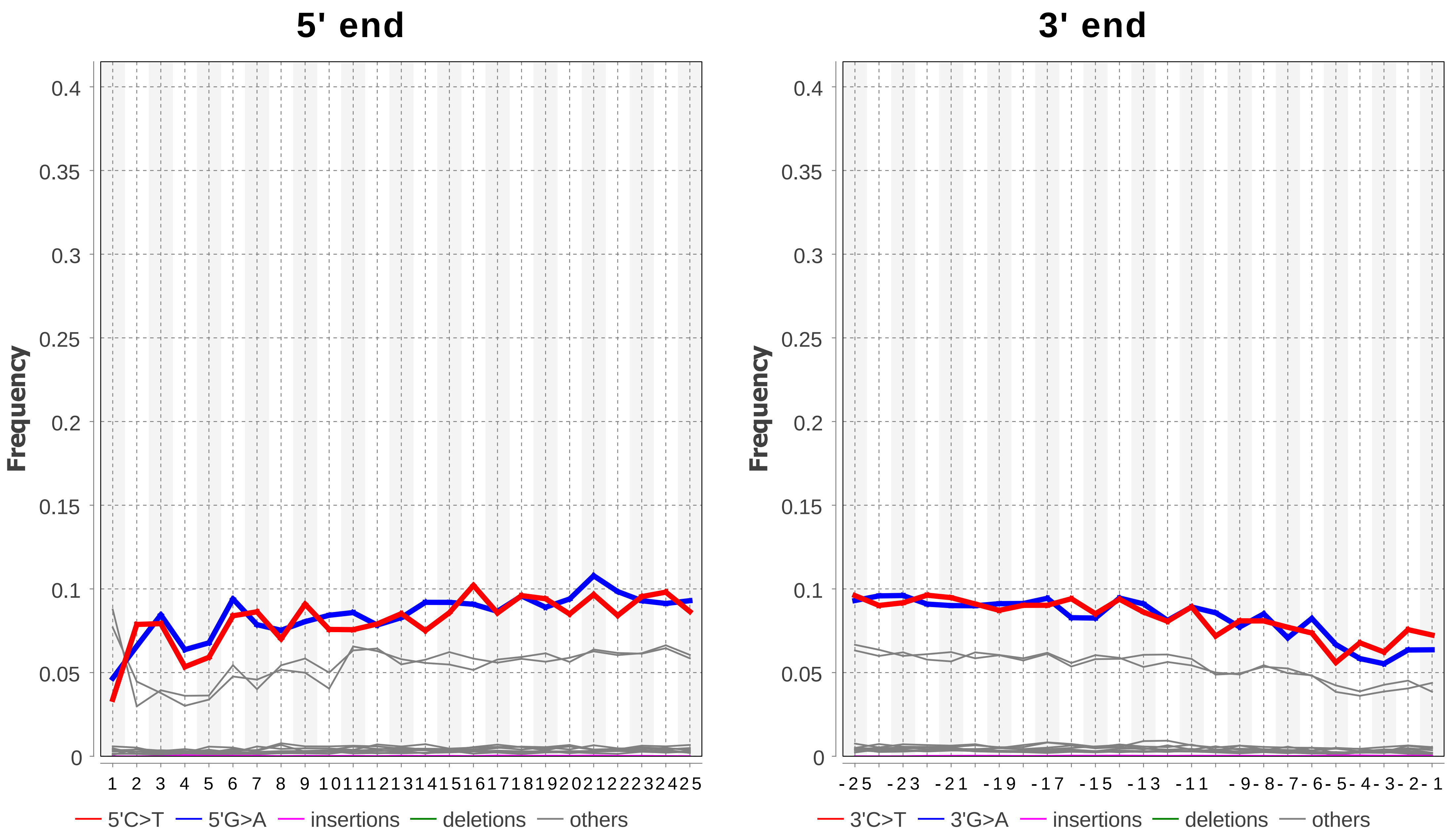

2757_MarkDuplicates
Number of used reads: 47,095 (100.0% of all input reads)

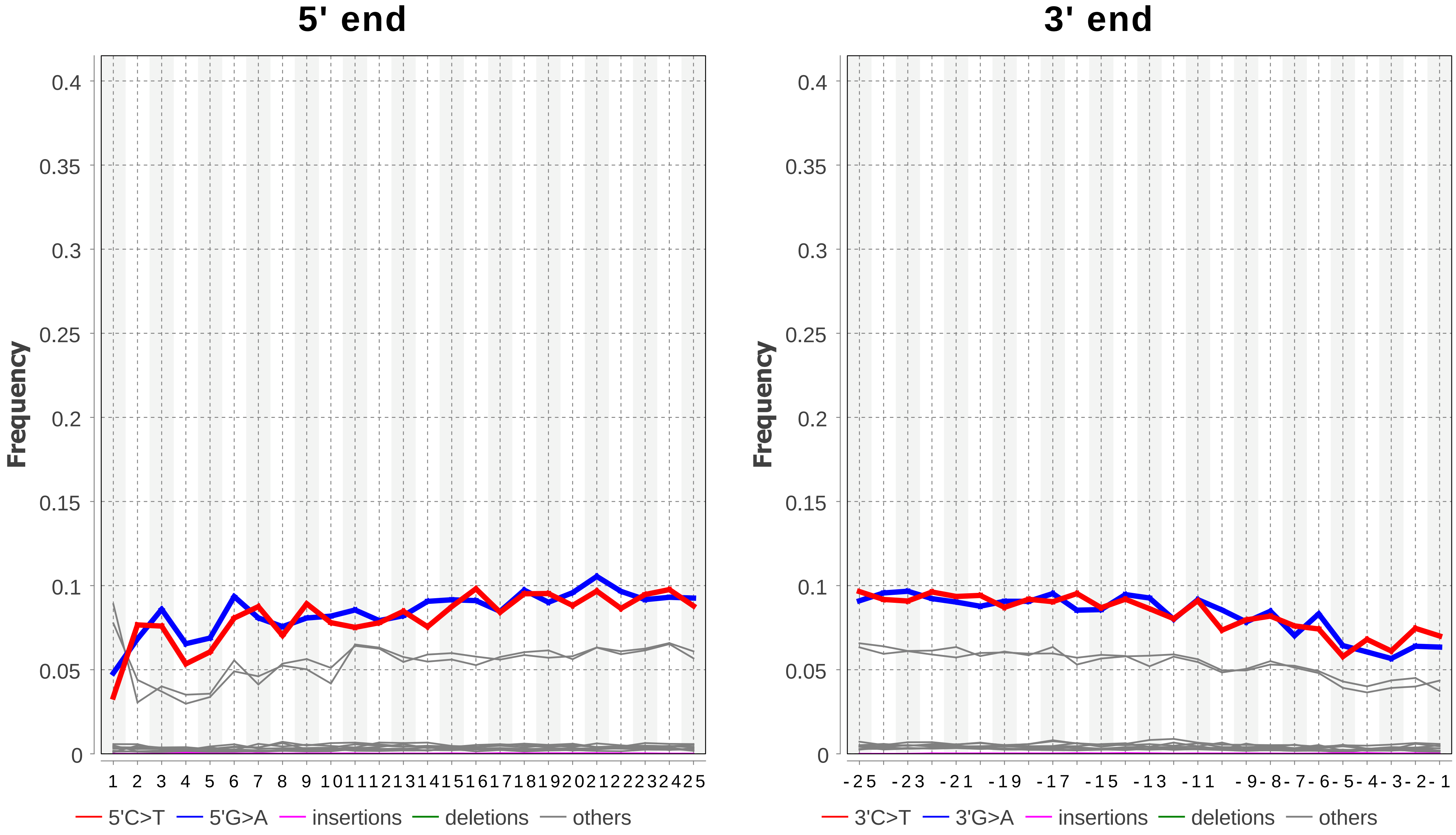

2759_aln

Number of used reads: 61,154 (100.0% of all input reads)

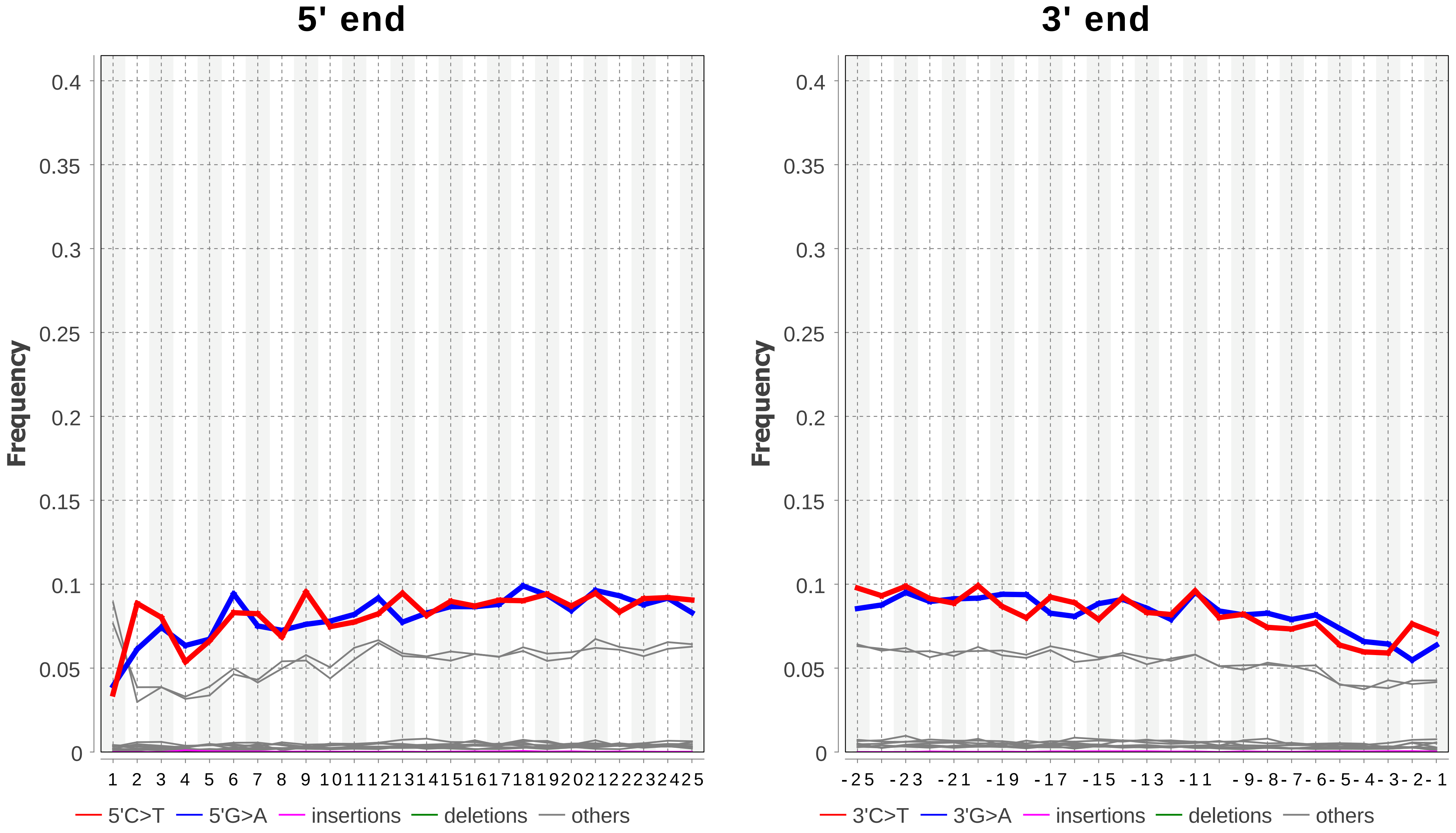

2759_MarkDuplicates
Number of used reads: 45,660 (100.0% of all input reads)

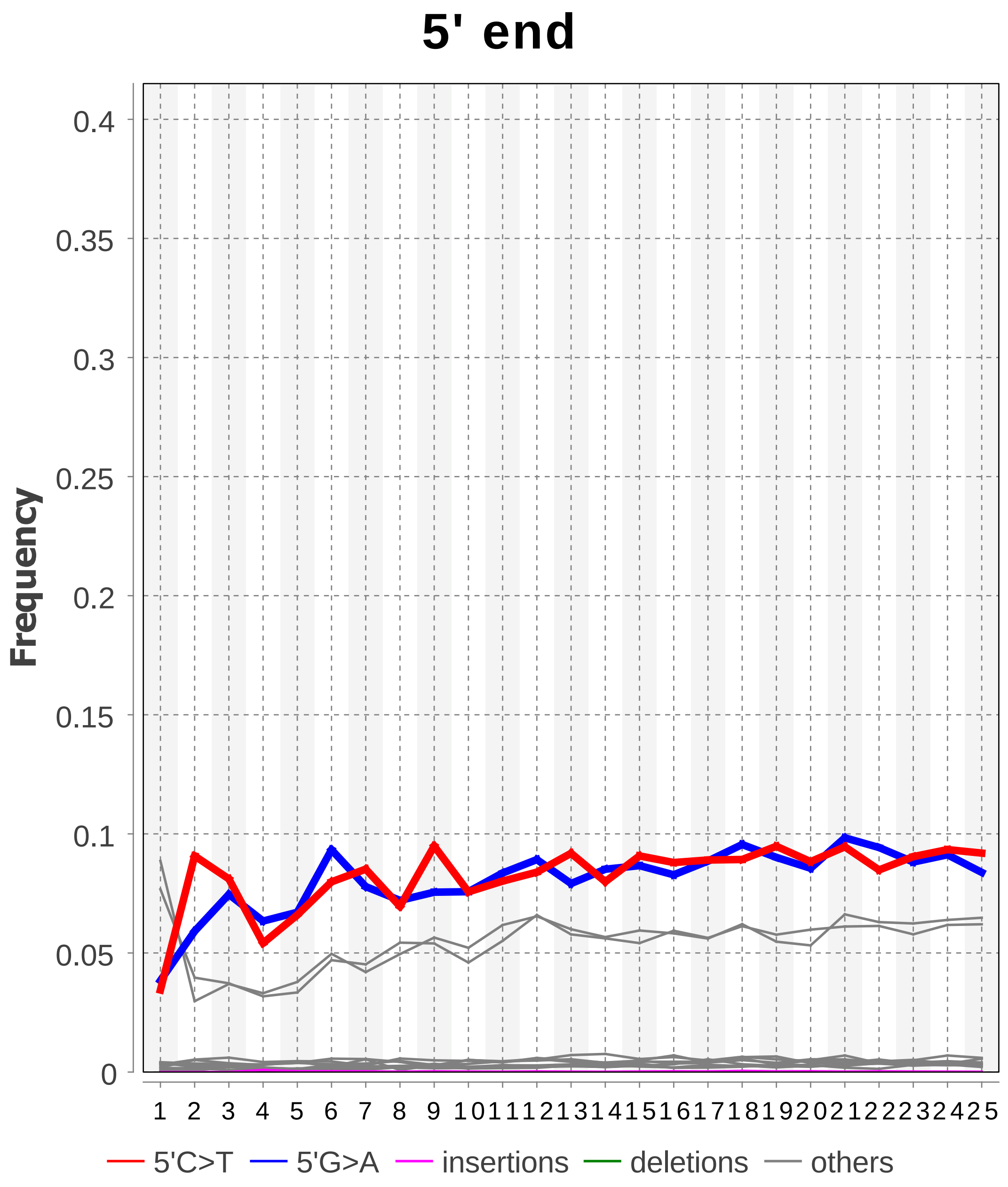

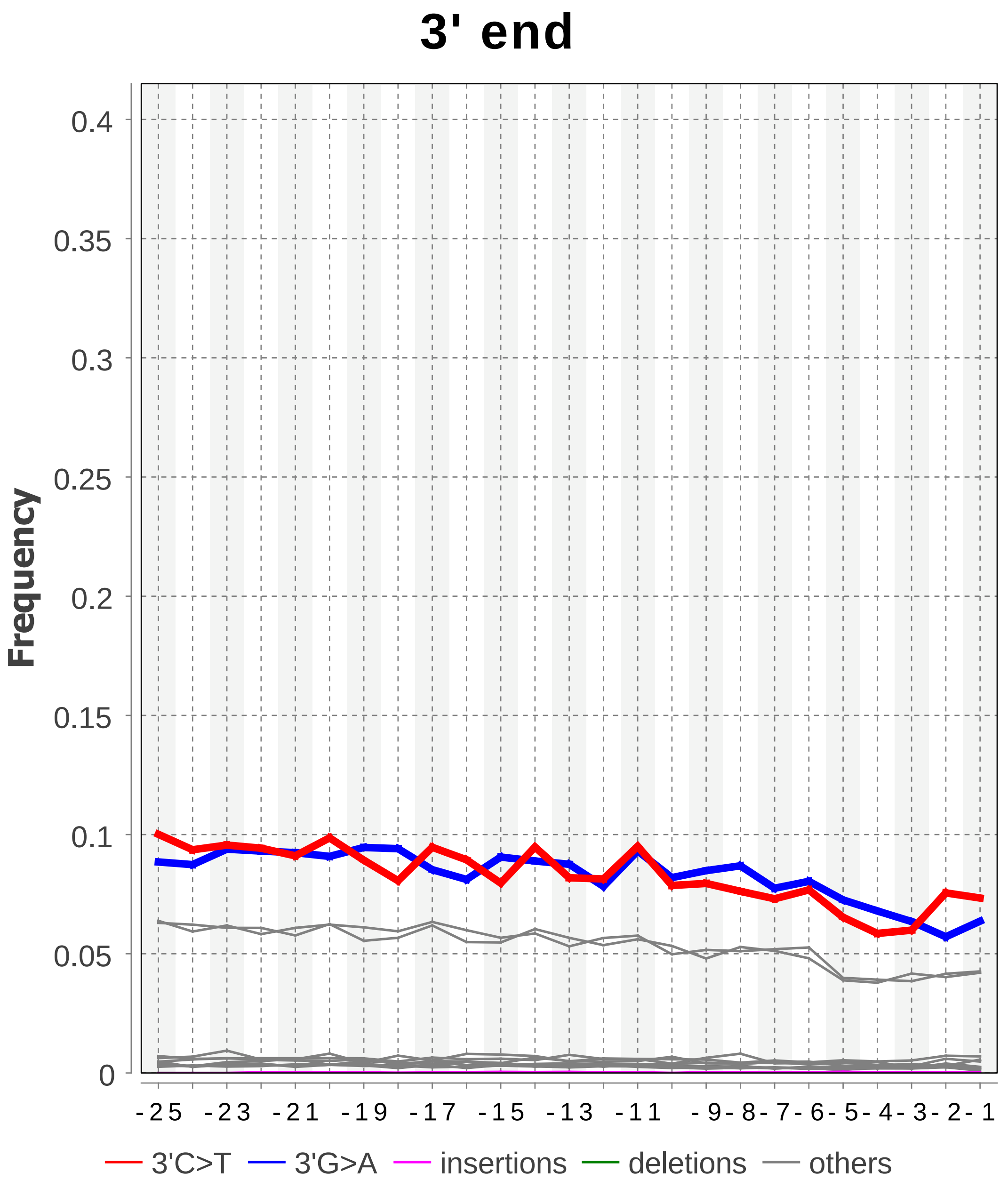

2829_aln
Number of used reads: 36,493 (100.0% of all input reads)

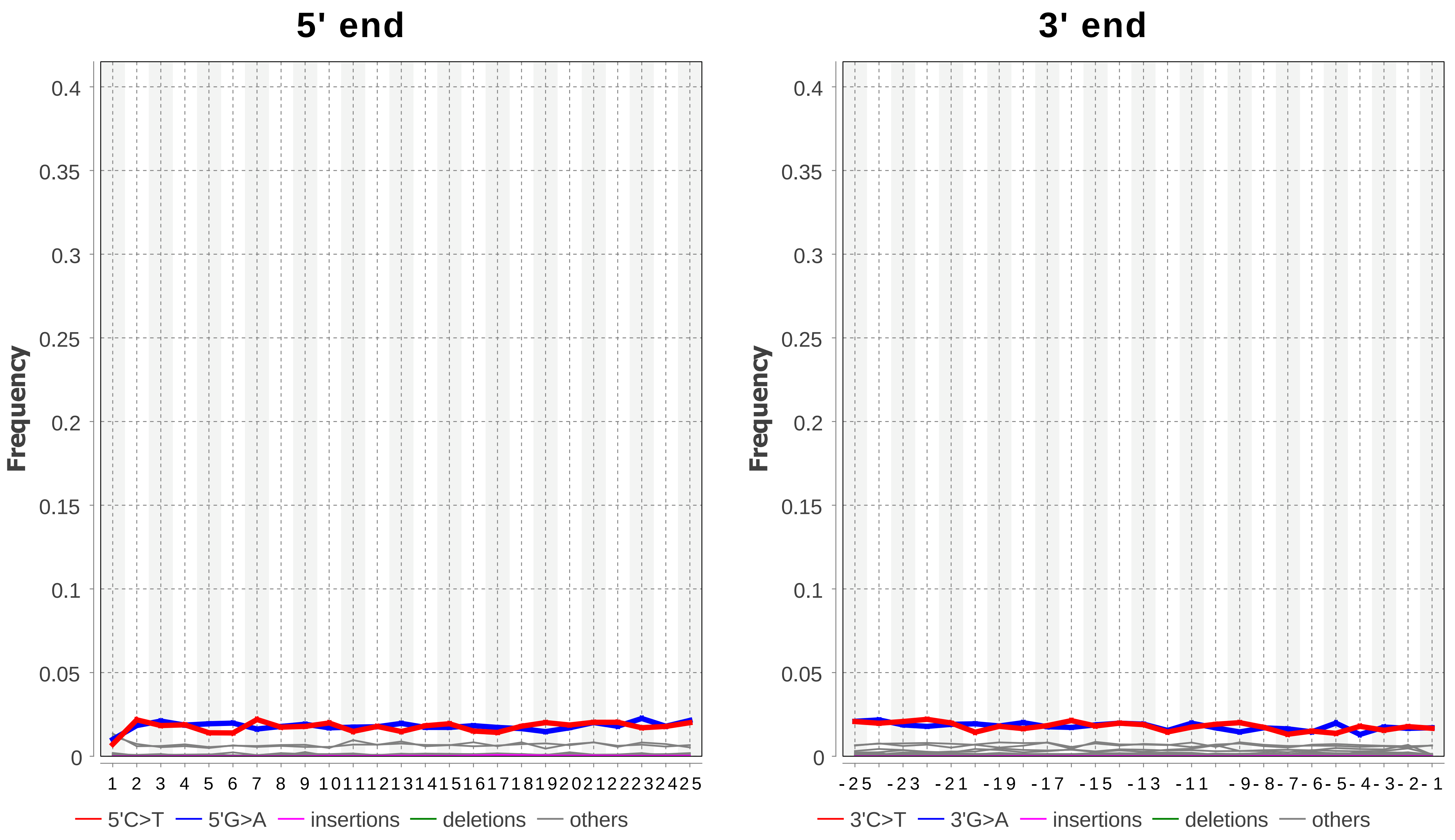

2829_MarkDuplicates
Number of used reads: 32,093 (100.0% of all input reads)

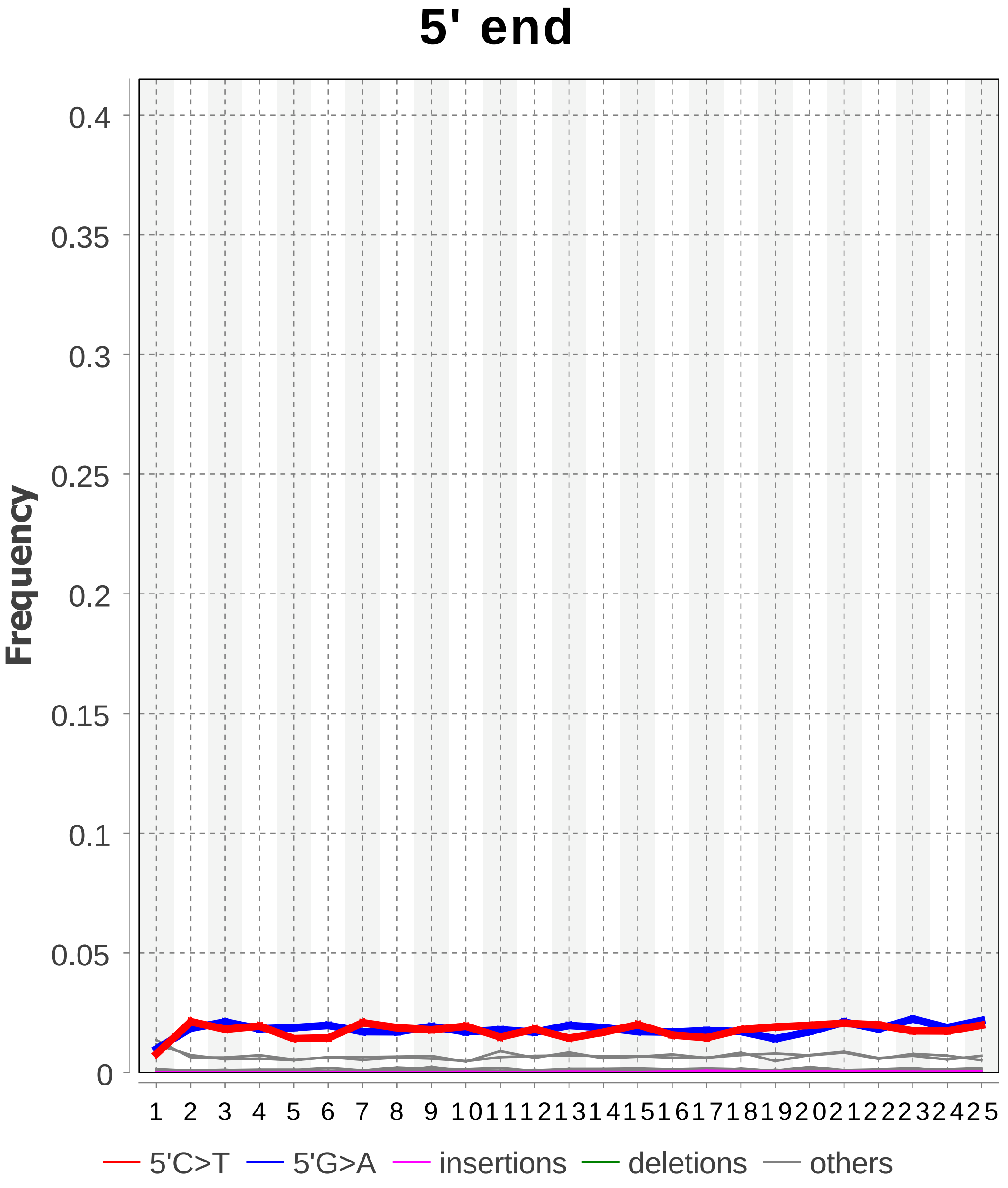

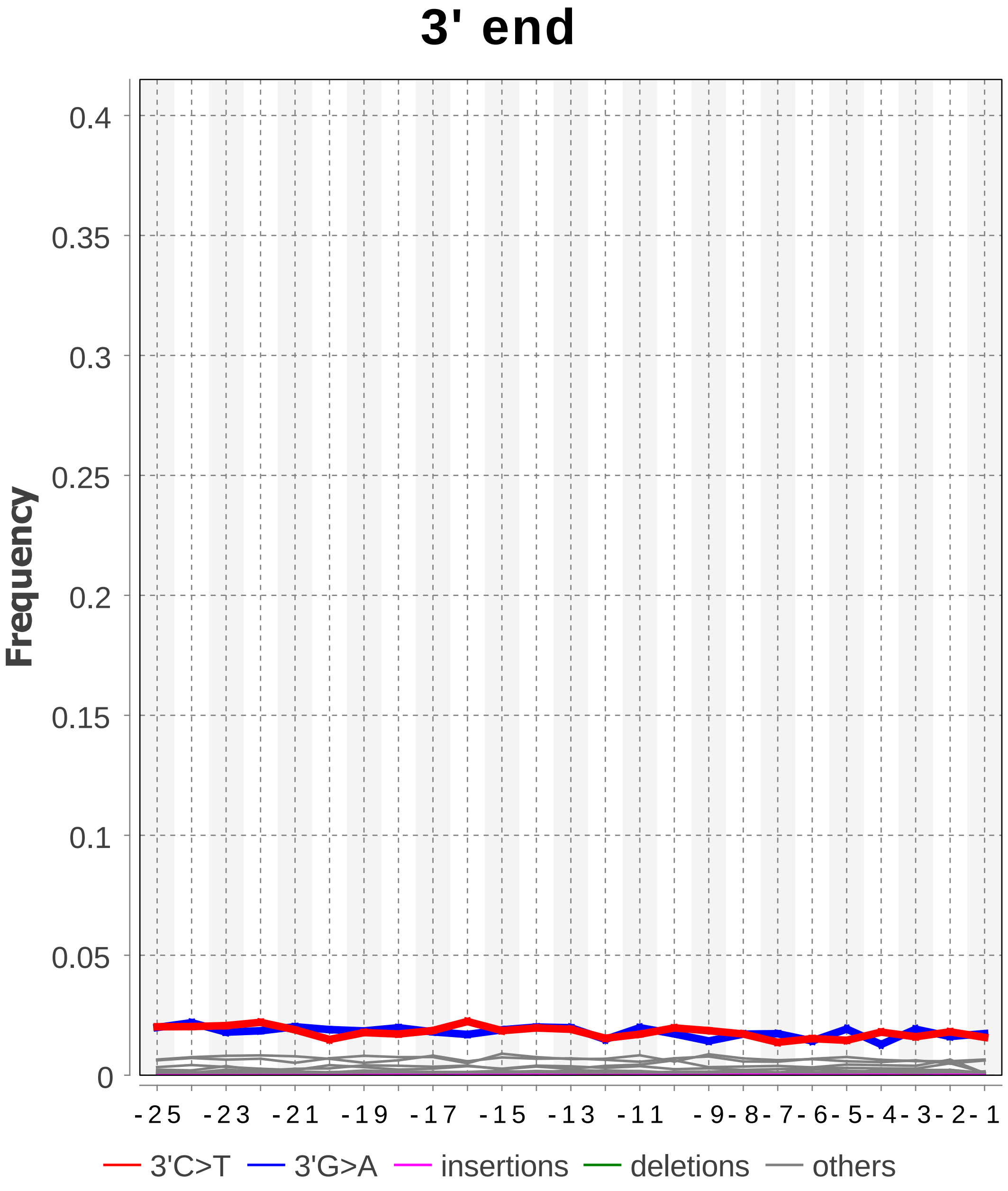

2836_aln

Number of used reads: 114,816 (100.0% of all input reads)

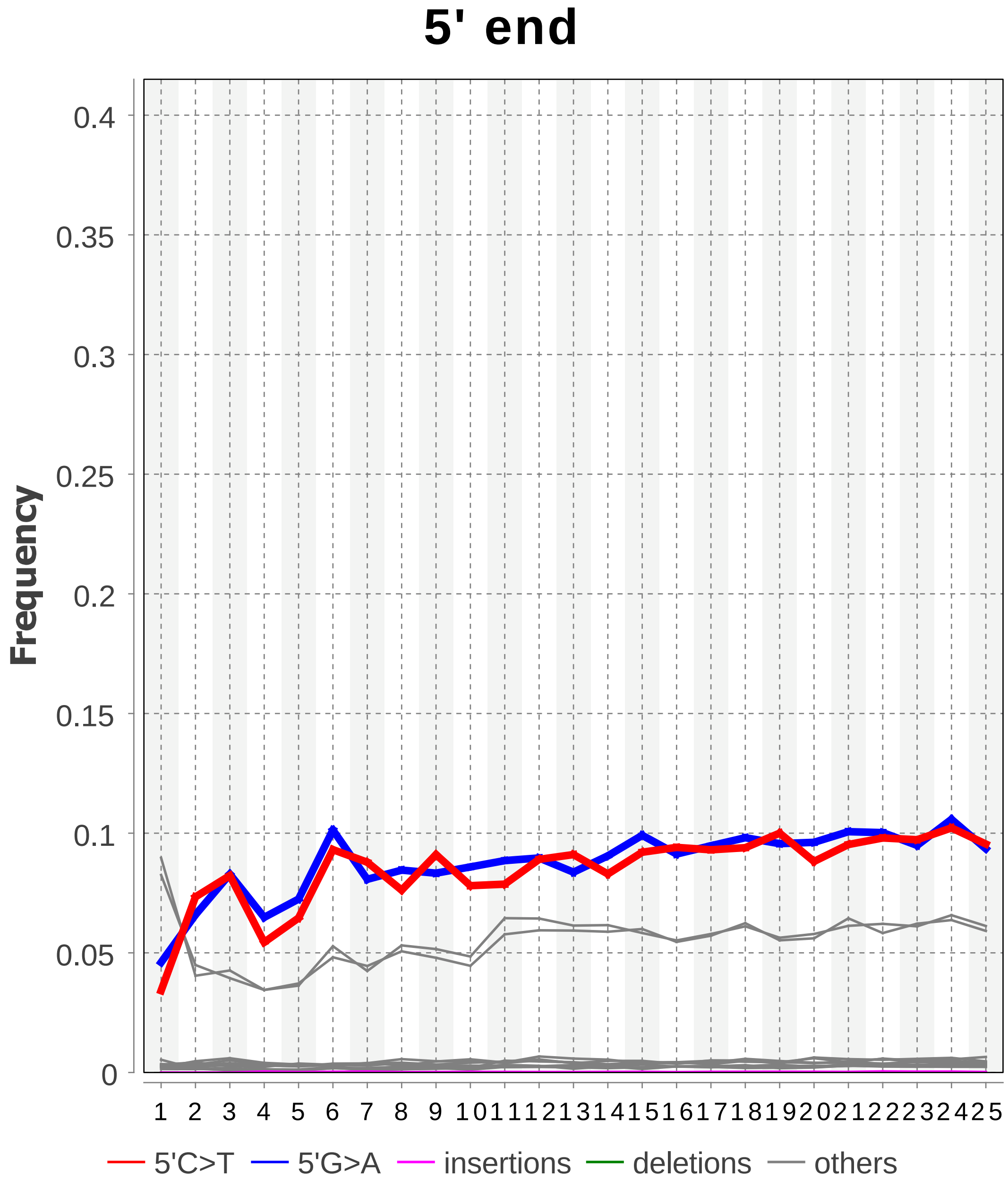

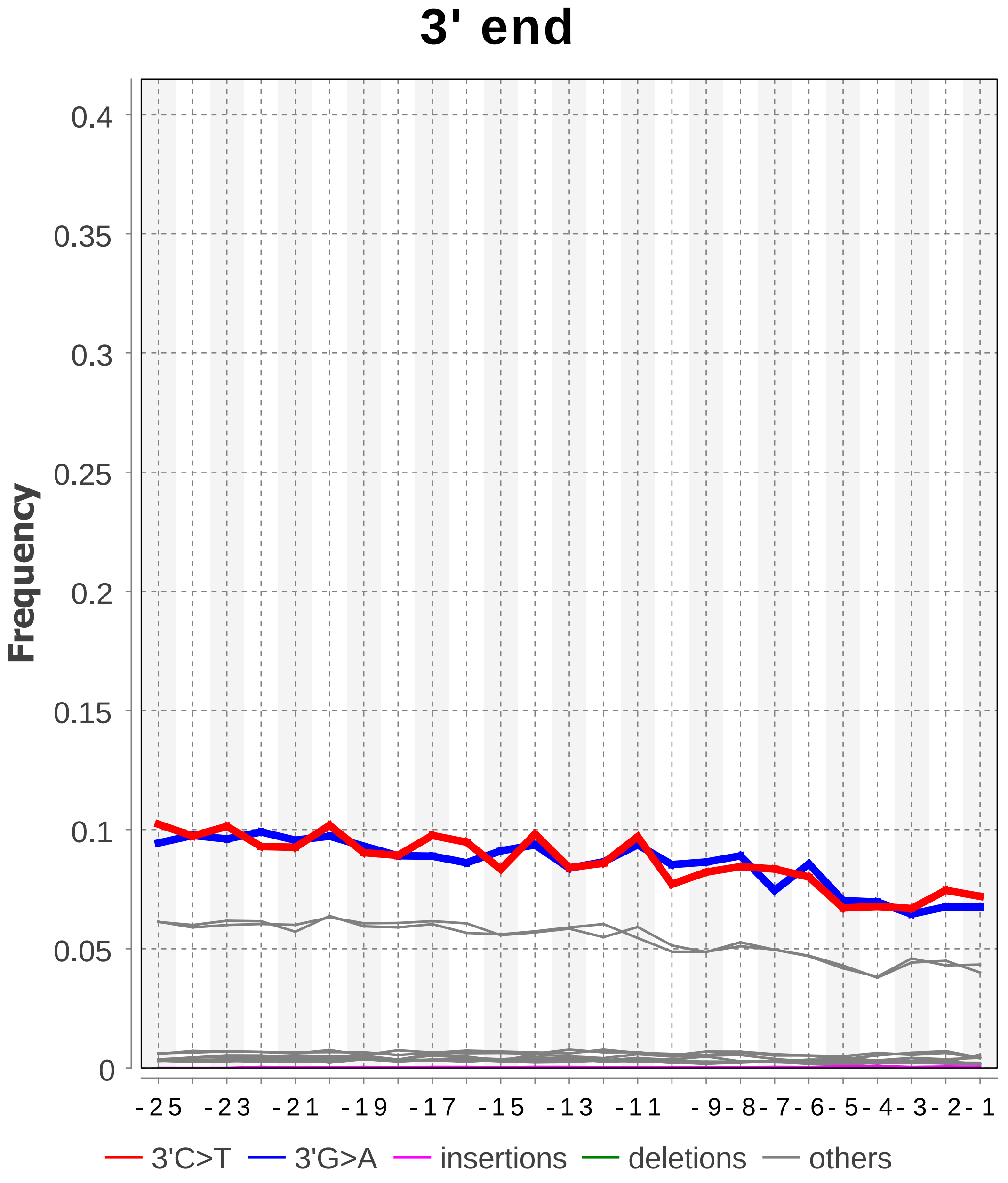

2836_MarkDuplicates
Number of used reads: 93,615 (100.0% of all input reads)

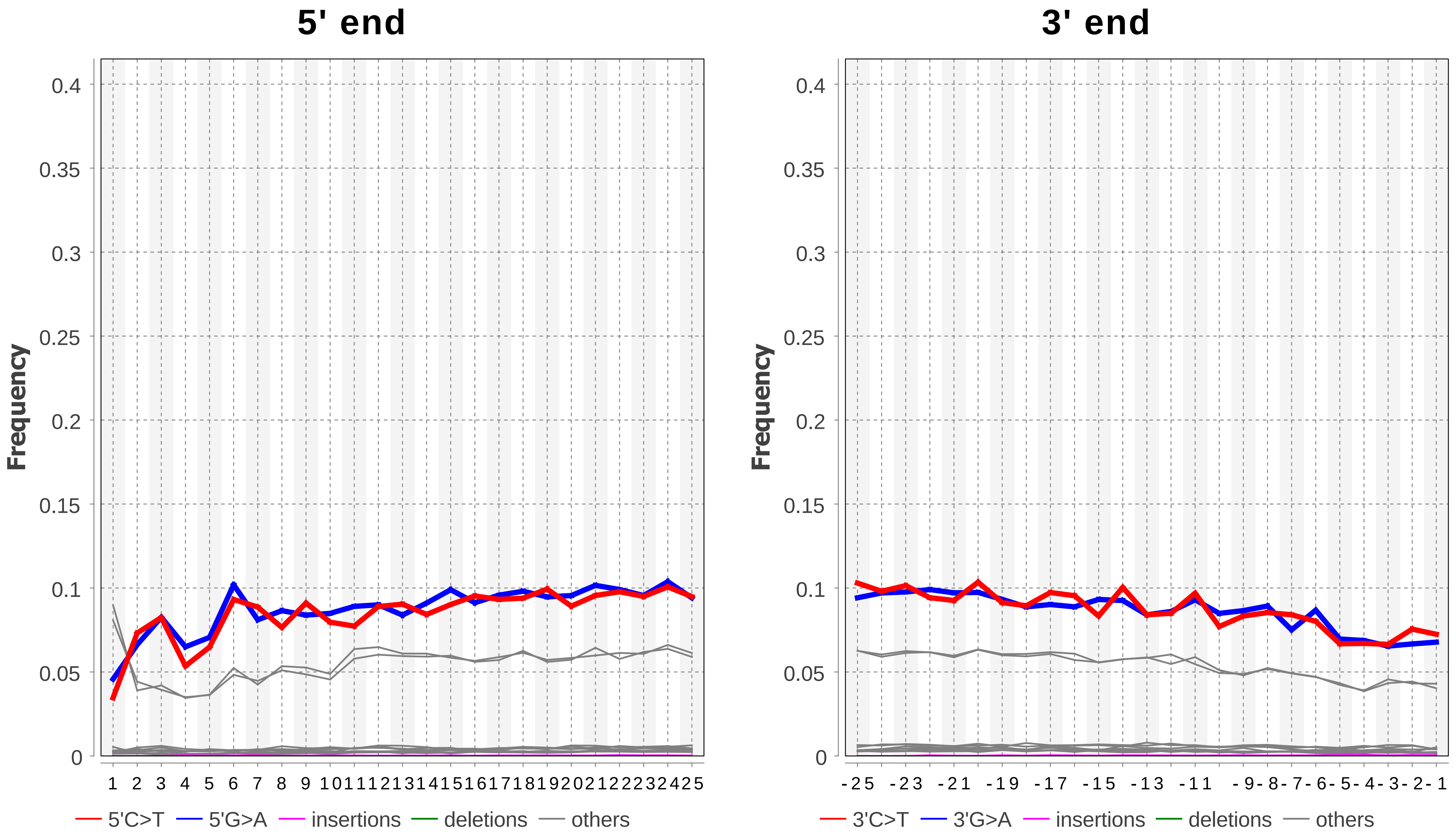

2844_aln

Number of used reads: 226,299 (100.0% of all input reads)

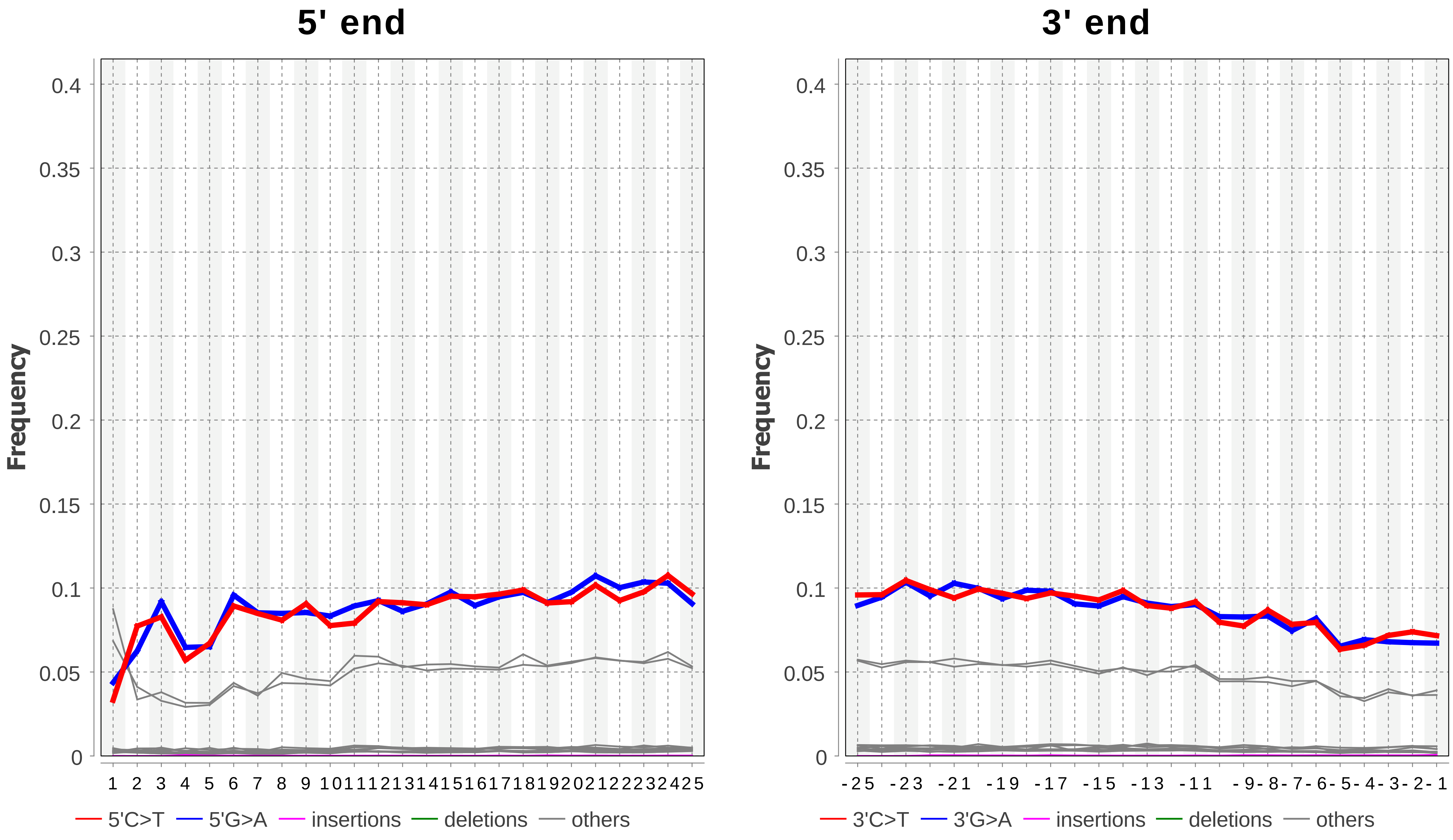

2844_MarkDuplicates
Number of used reads: 181,473 (100.0% of all input reads)

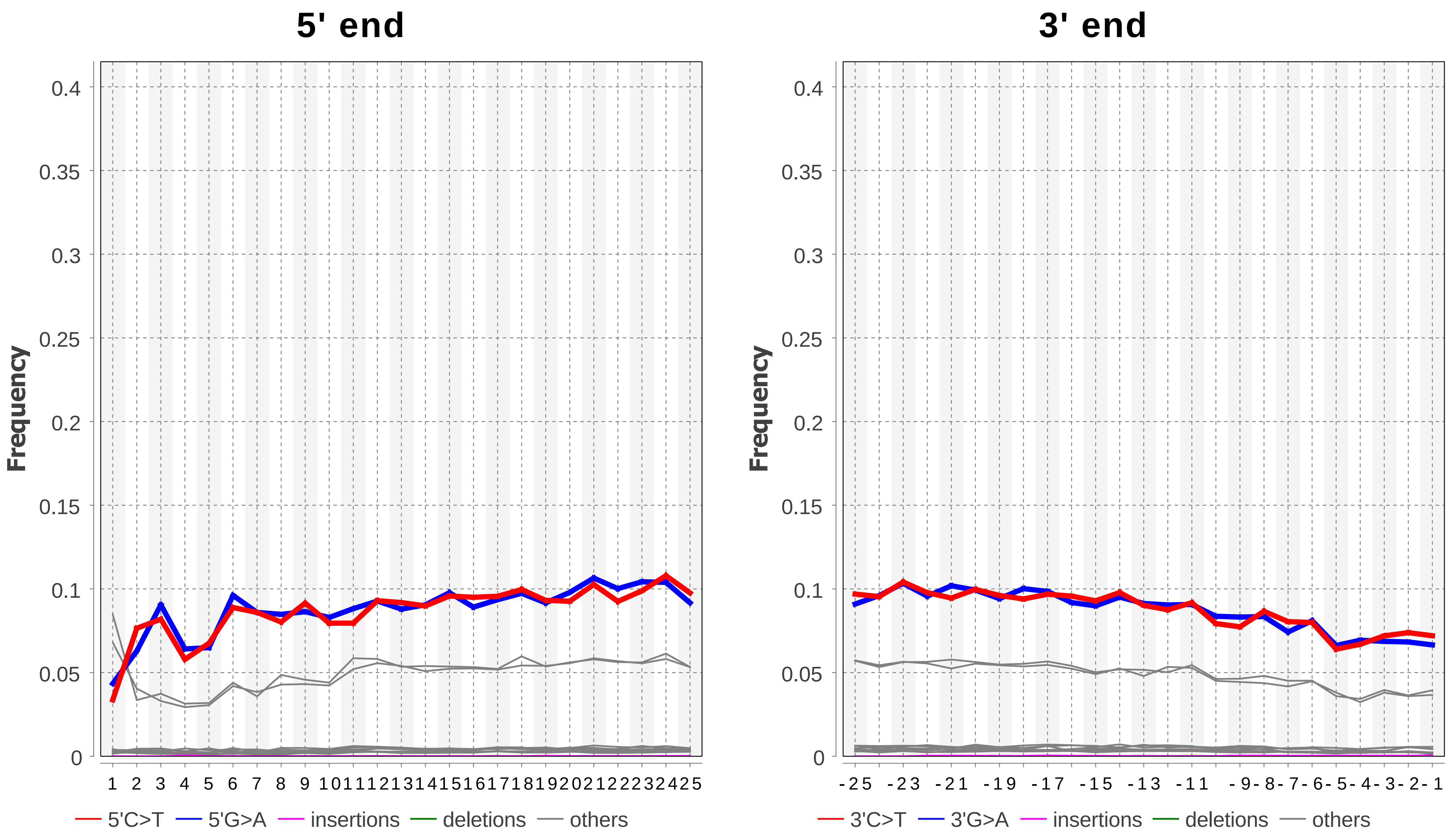

2845_aln

Number of used reads: 100,567 (100.0% of all input reads)

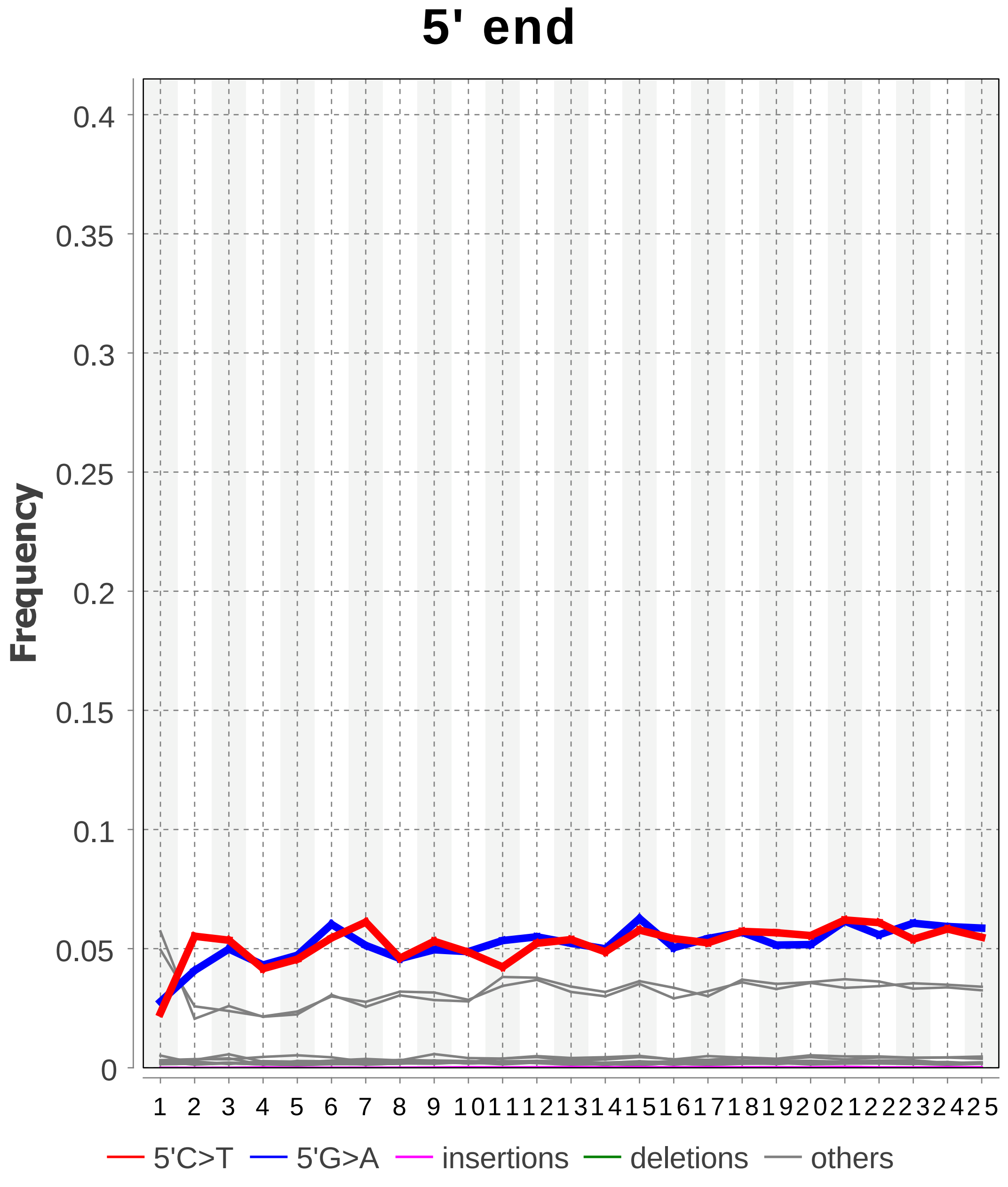

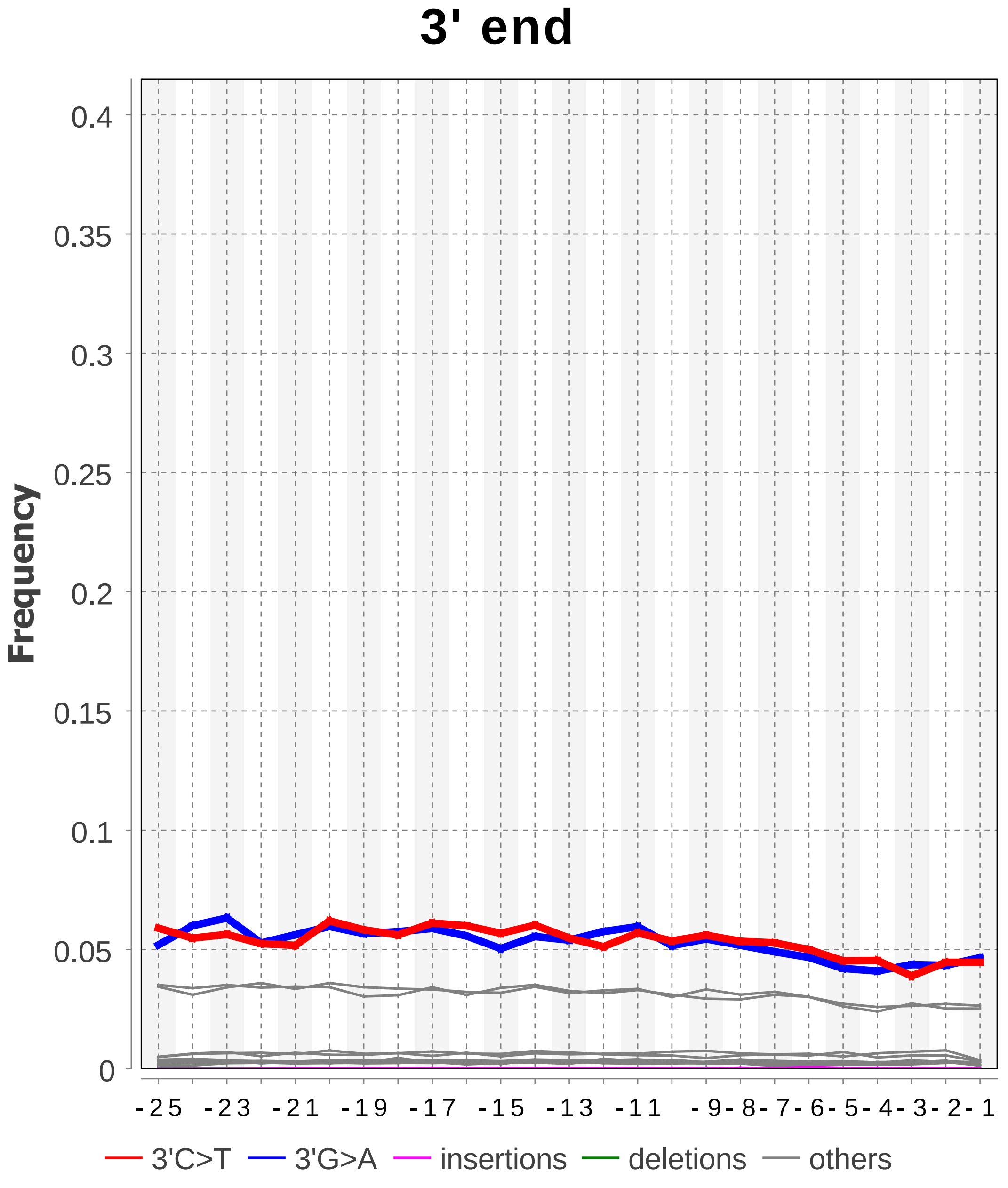

2845_MarkDuplicates

Number of used reads: 79,588 (100.0% of all input reads)

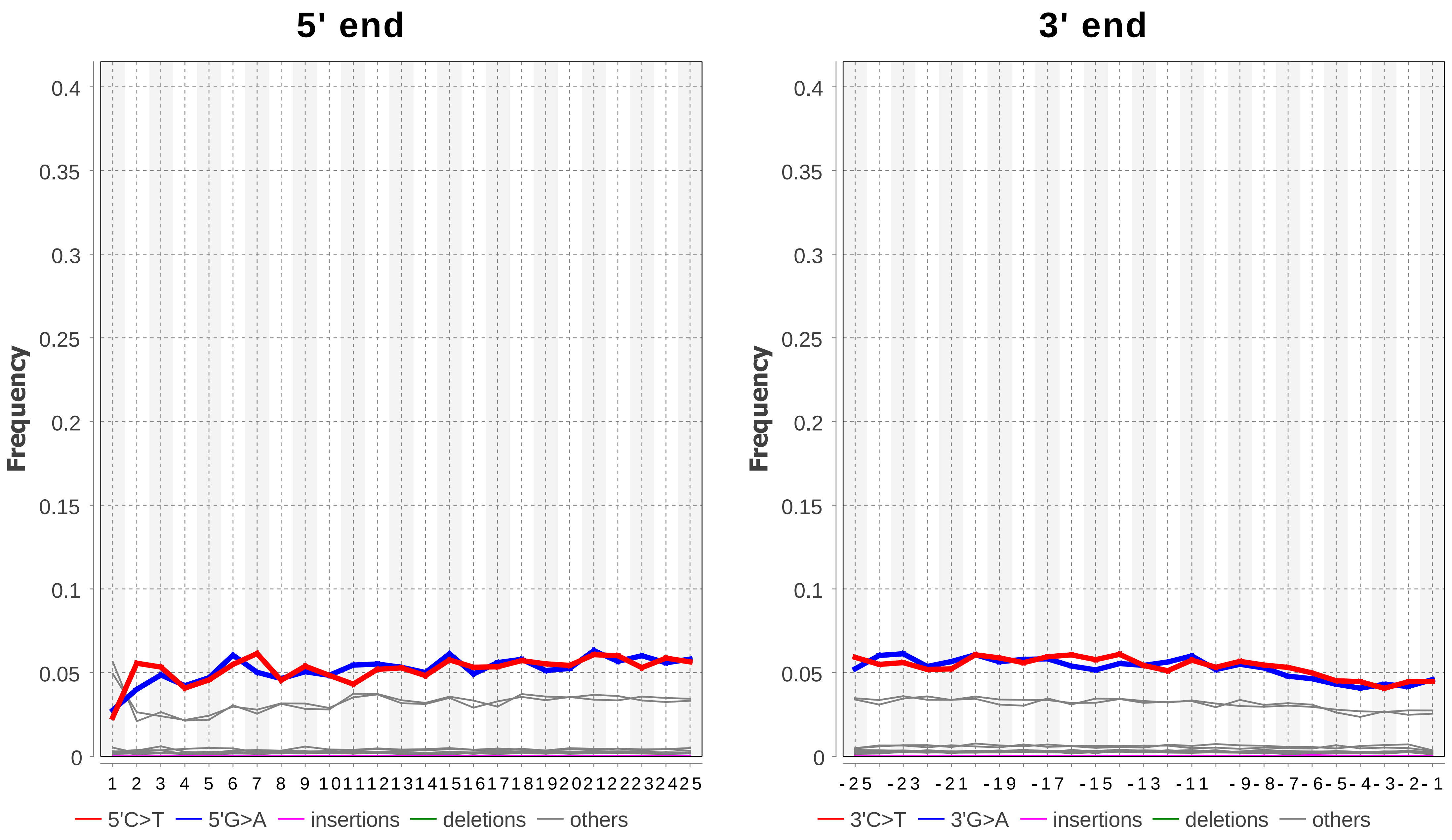

3084_aln

Number of used reads: 44,974 (100.0% of all input reads)

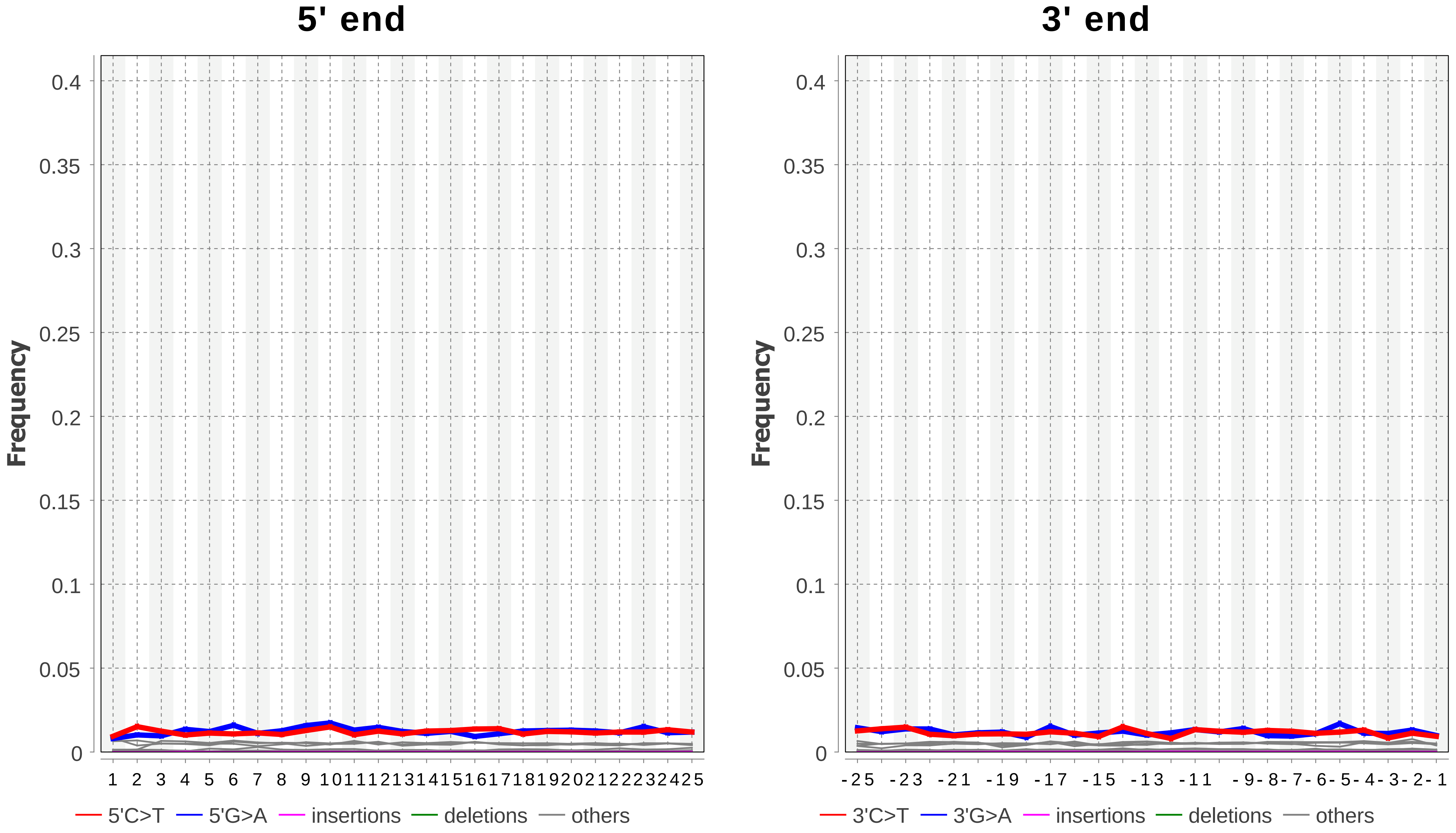

3084_MarkDuplicates

Number of used reads: 33,334 (100.0% of all input reads)

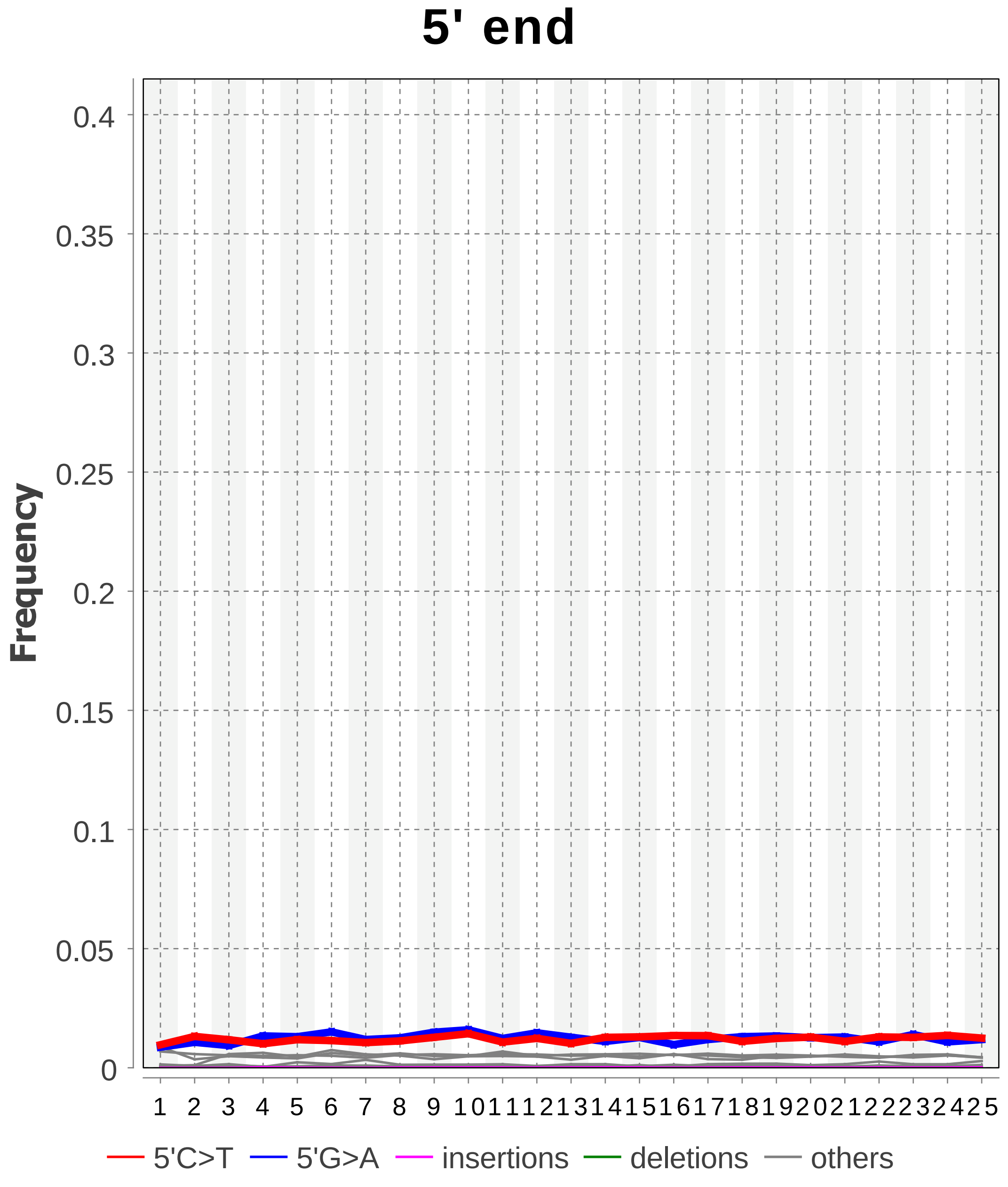

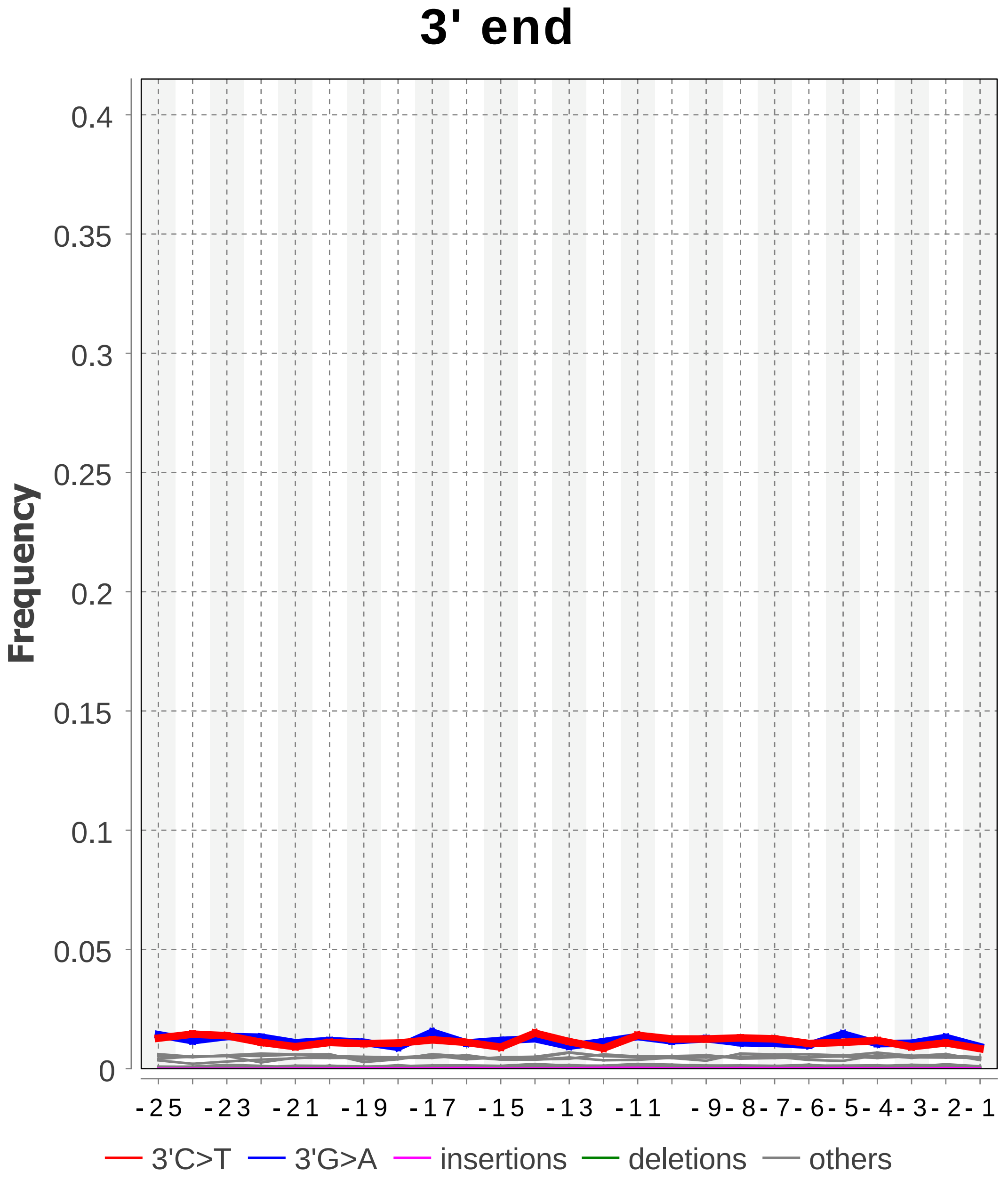

3086_aln

Number of used reads: 46,191 (100.0% of all input reads)

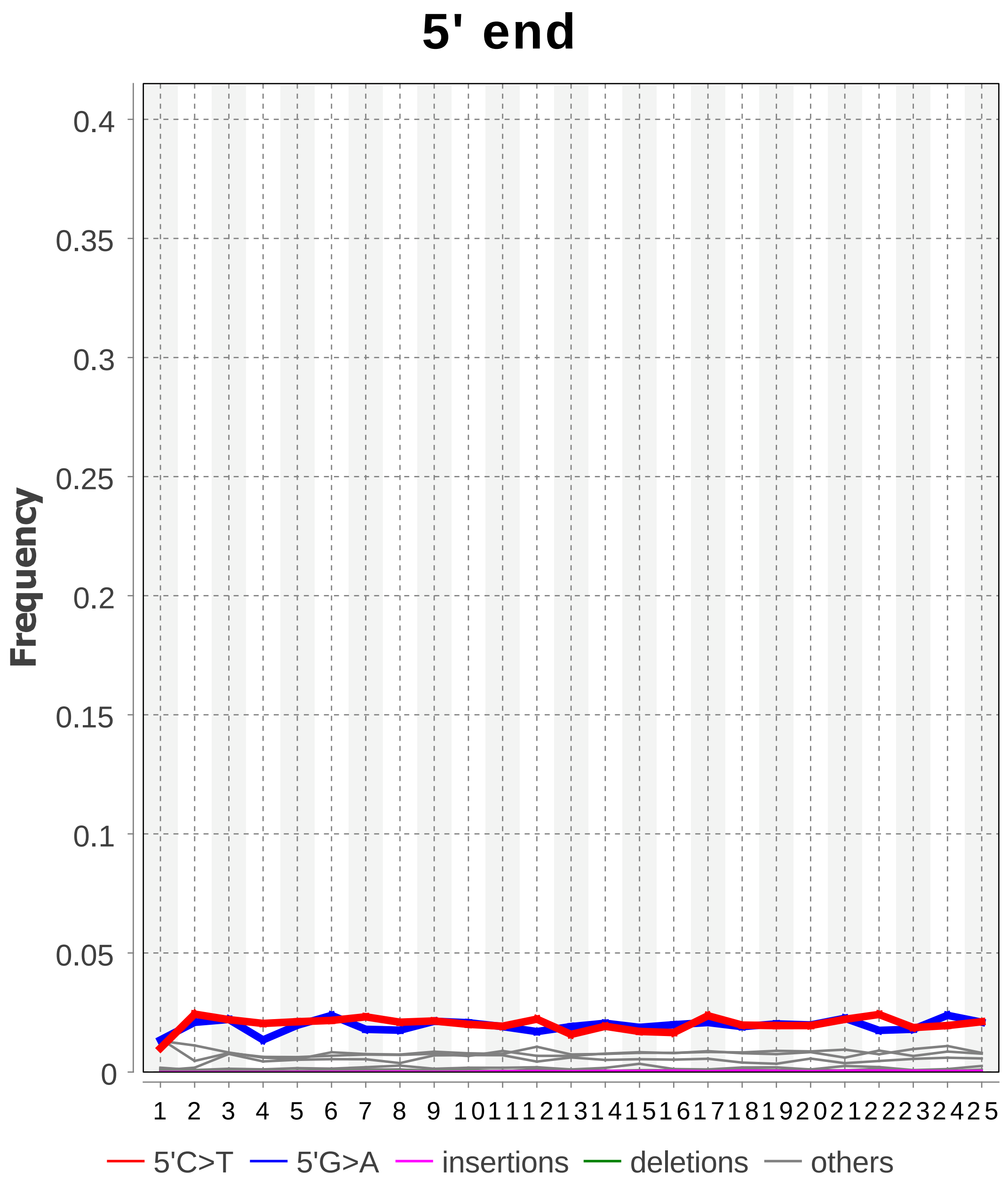

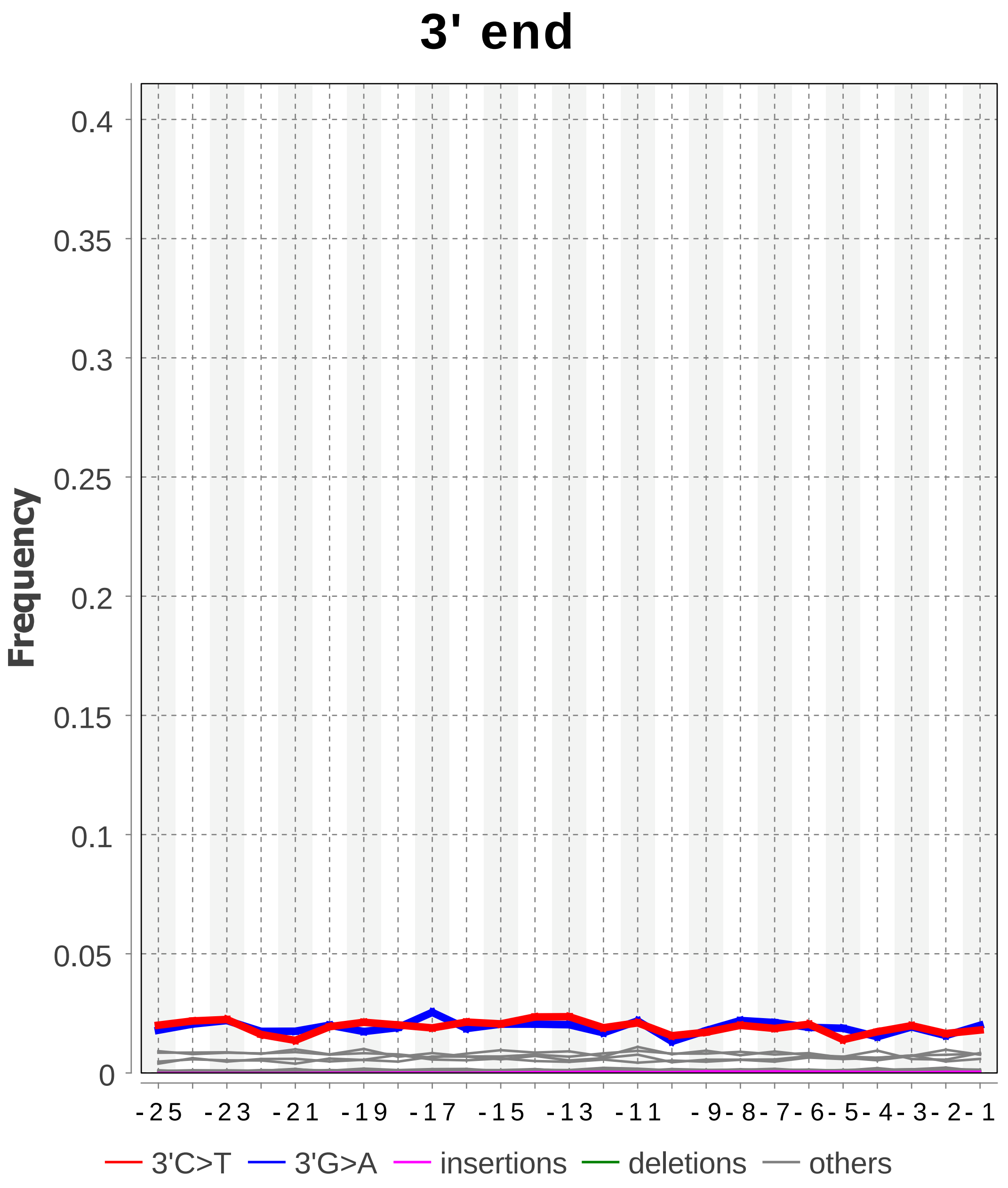

3086_MarkDuplicates

Number of used reads: 33,662 (100.0% of all input reads)

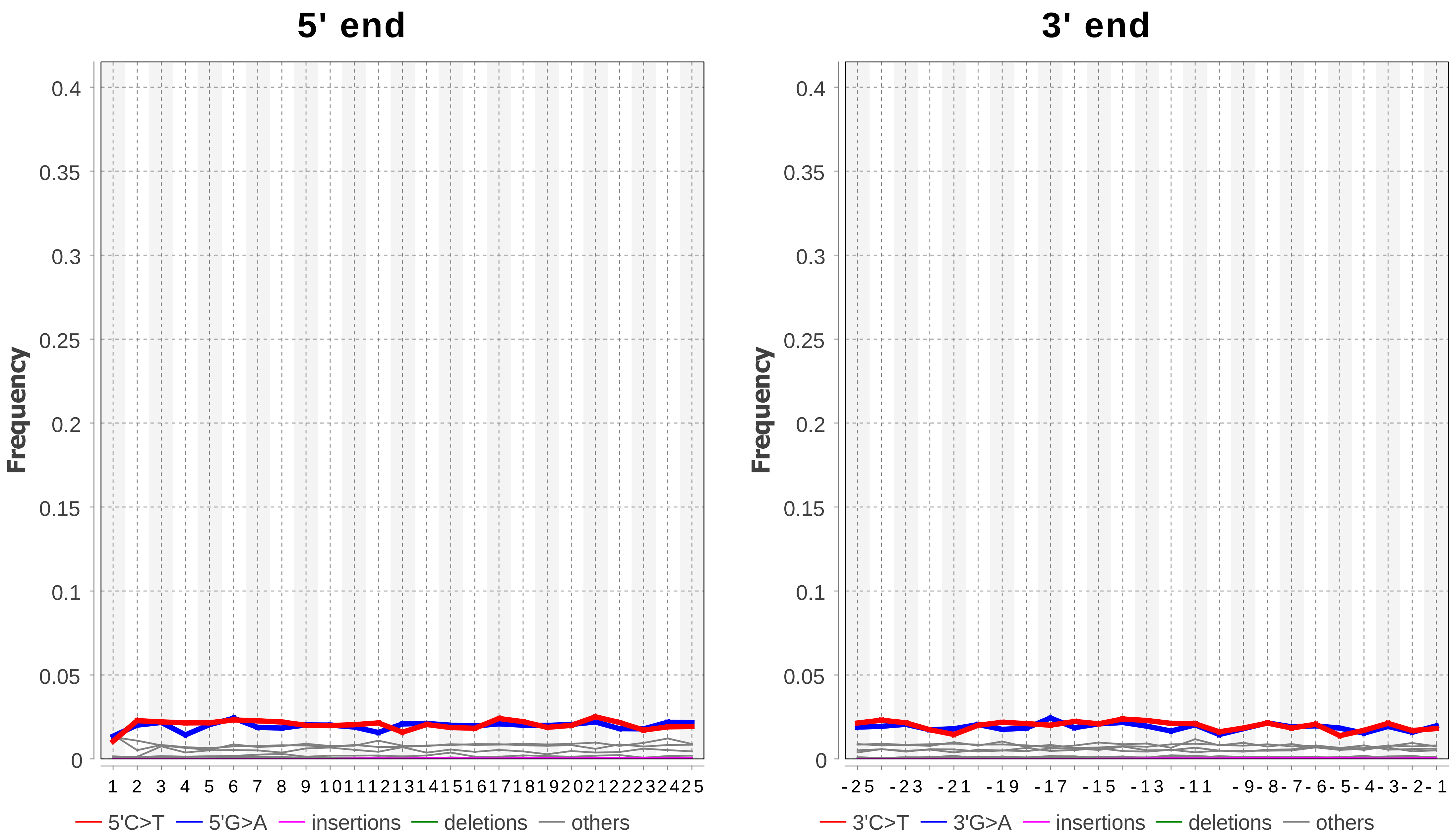

3761_aln

Number of used reads: 34,100 (100.0% of all input reads)

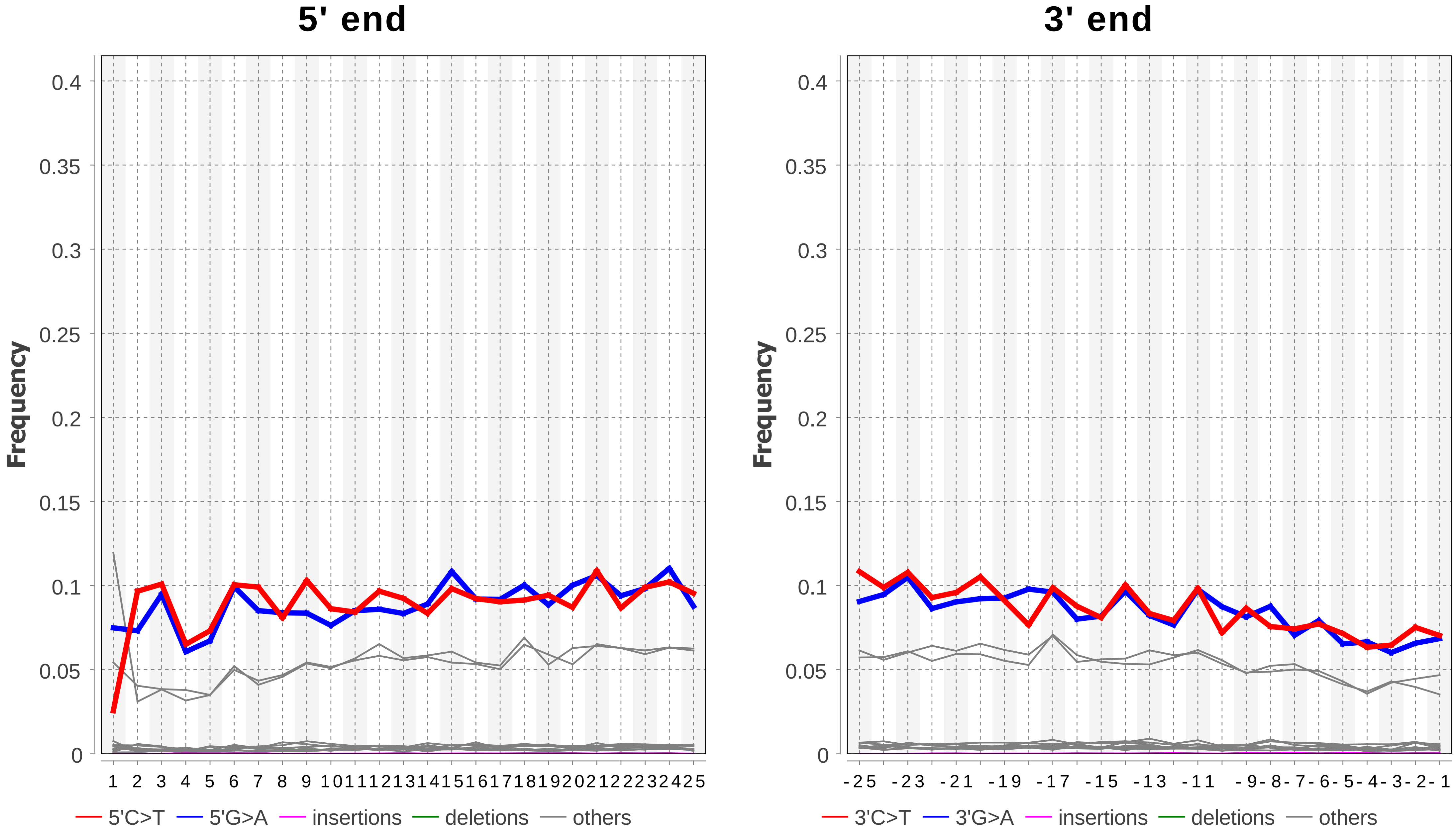

3761_MarkDuplicates
Number of used reads: 26,480 (100.0% of all input reads)

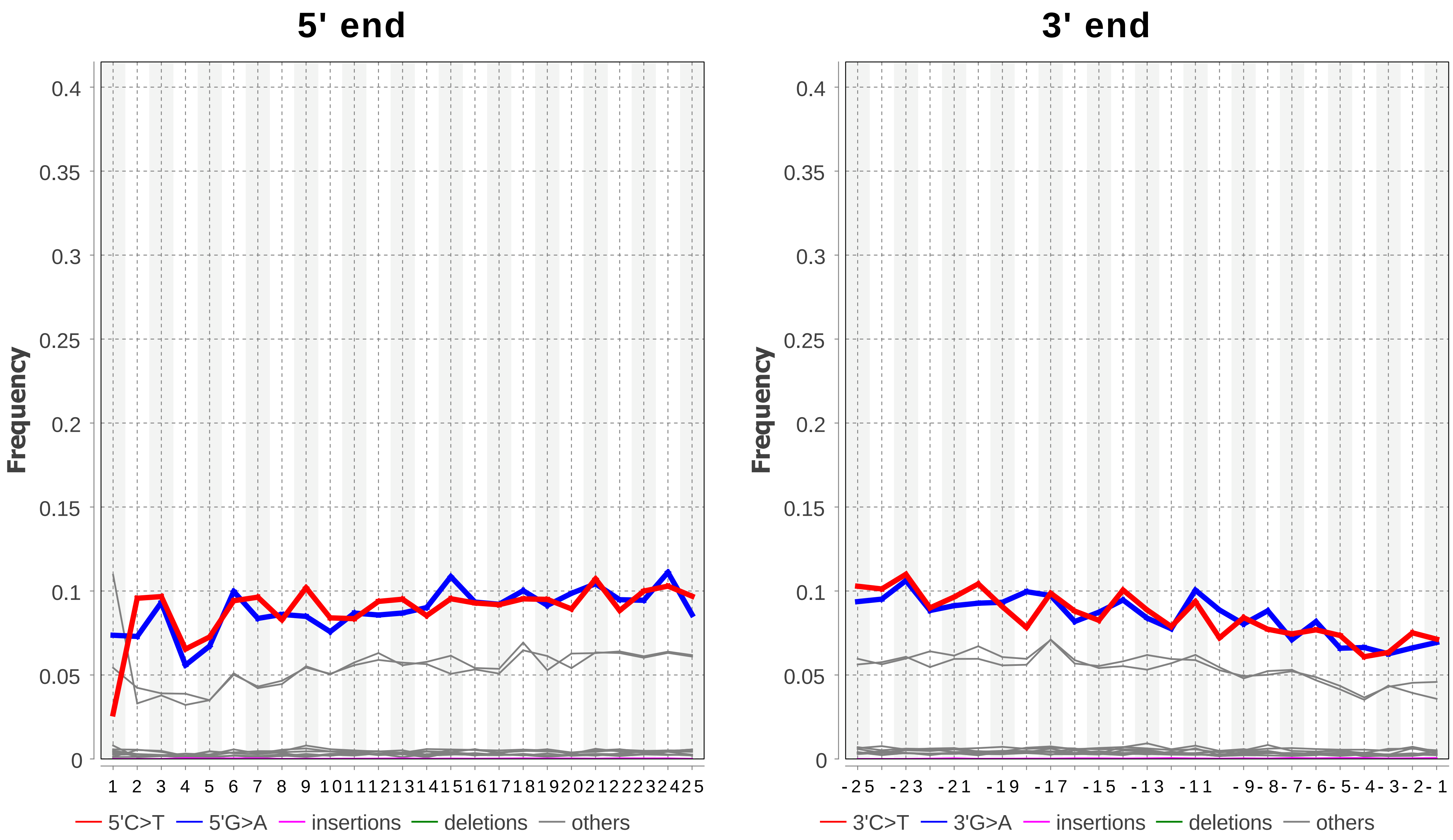

4201_aln

Number of used reads: 121,046 (100.0% of all input reads)

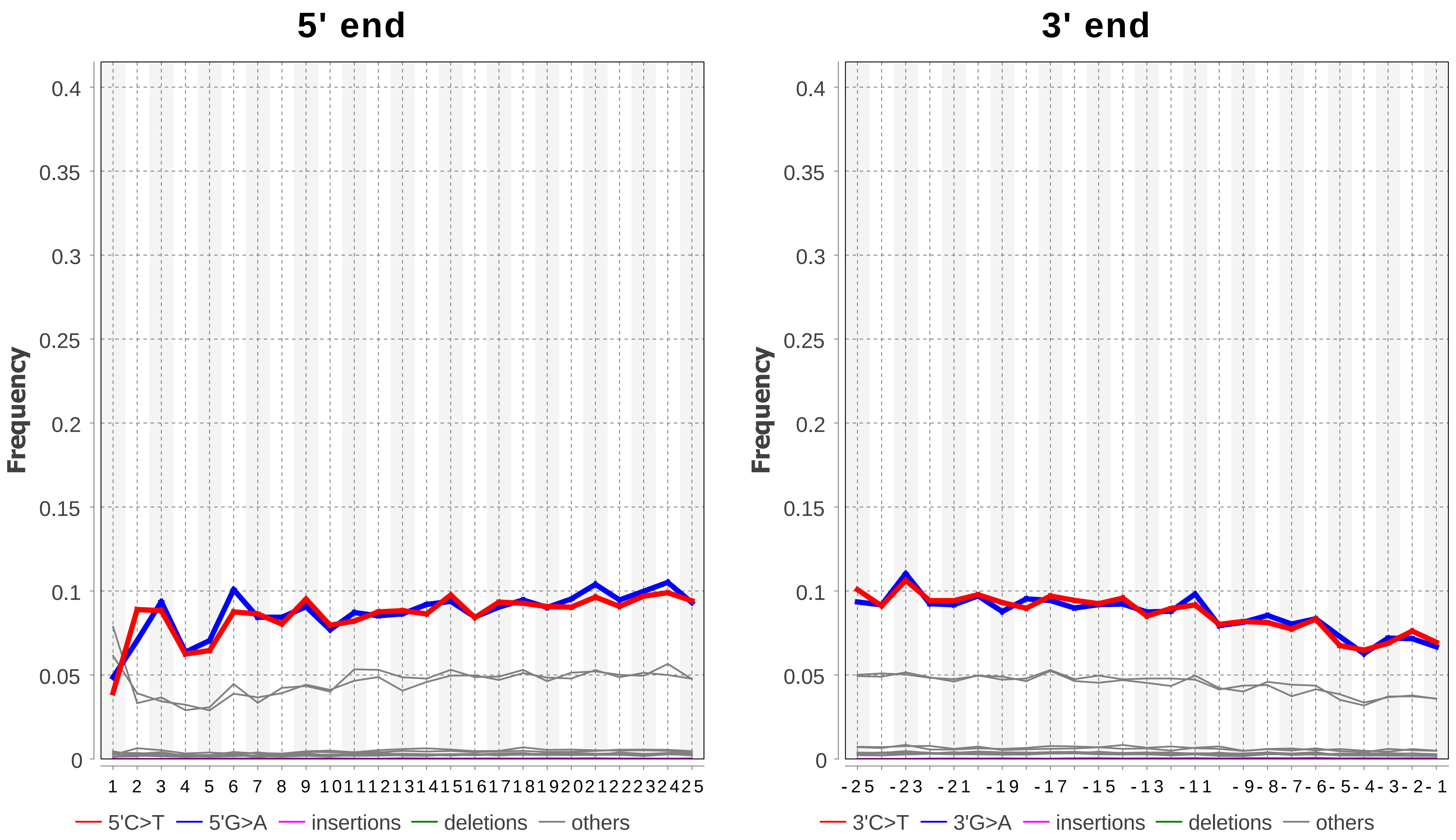

4201_MarkDuplicates
Number of used reads: 98,354 (100.0% of all input reads)

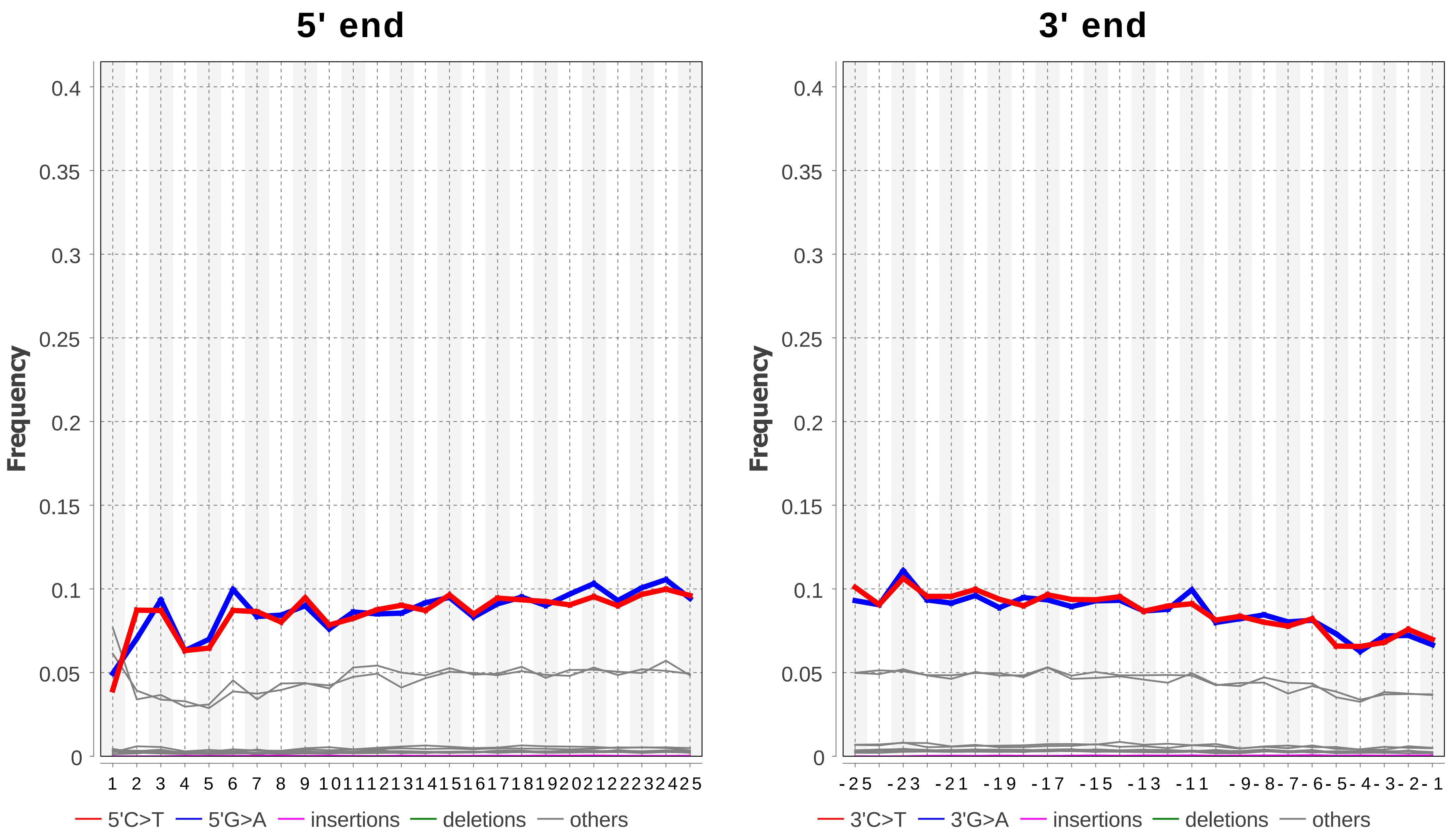

4229_aln

Number of used reads: 93,107 (100.0% of all input reads)

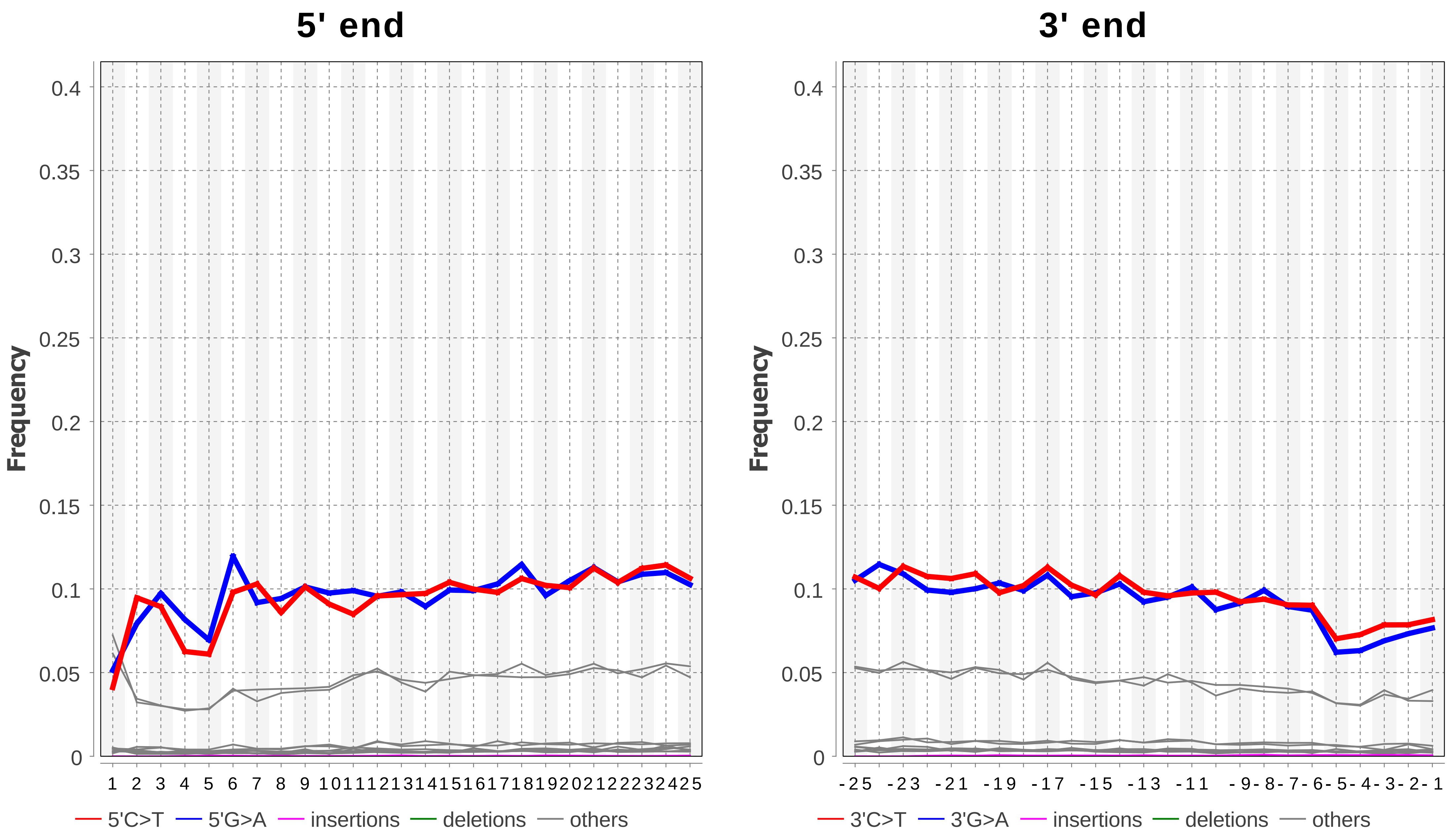

4229_MarkDuplicates
Number of used reads: 75,549 (100.0% of all input reads)

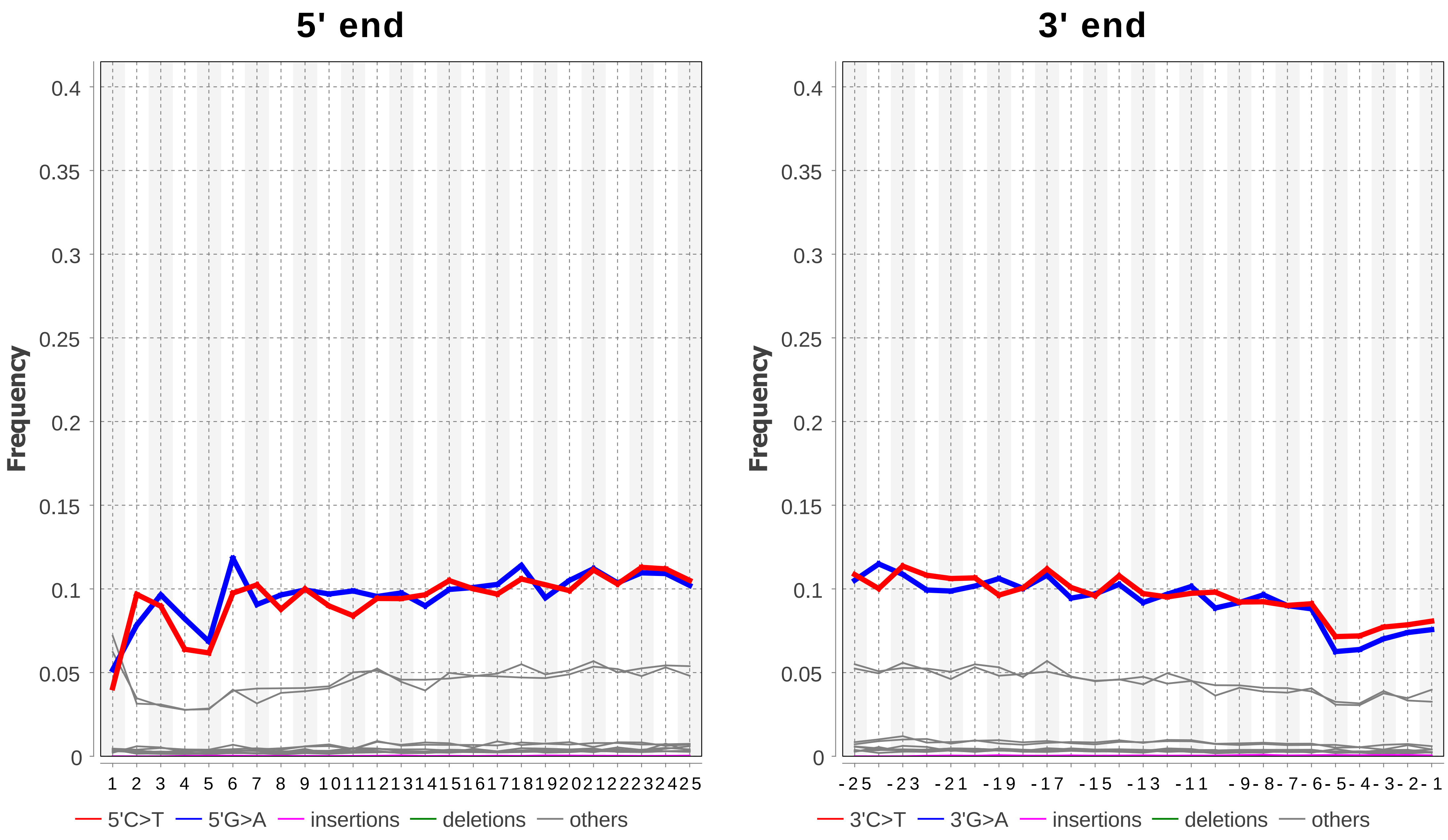

4230_aln

Number of used reads: 146,646 (100.0% of all input reads)

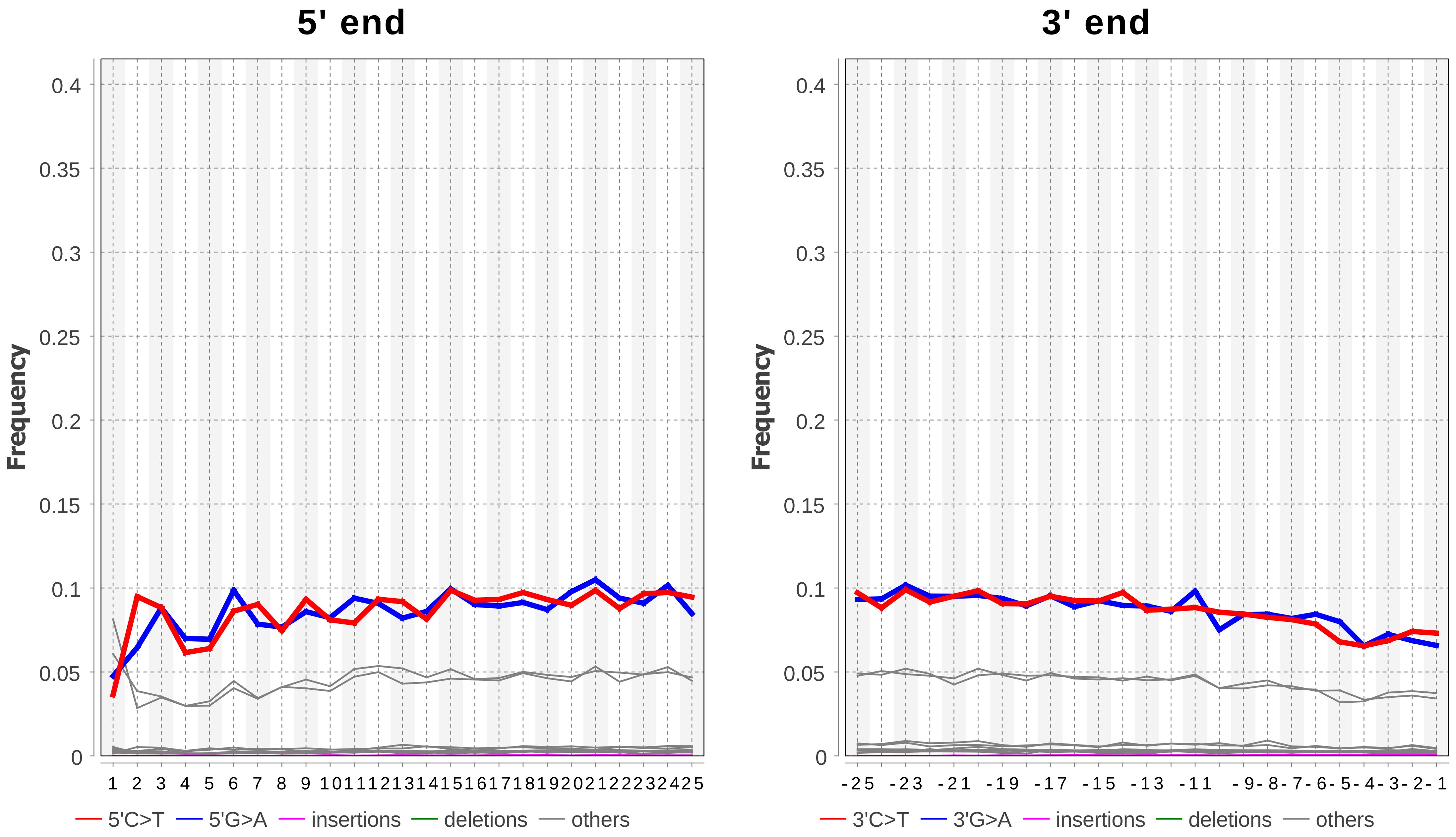

4230_MarkDuplicates
Number of used reads: 124,107 (100.0% of all input reads)

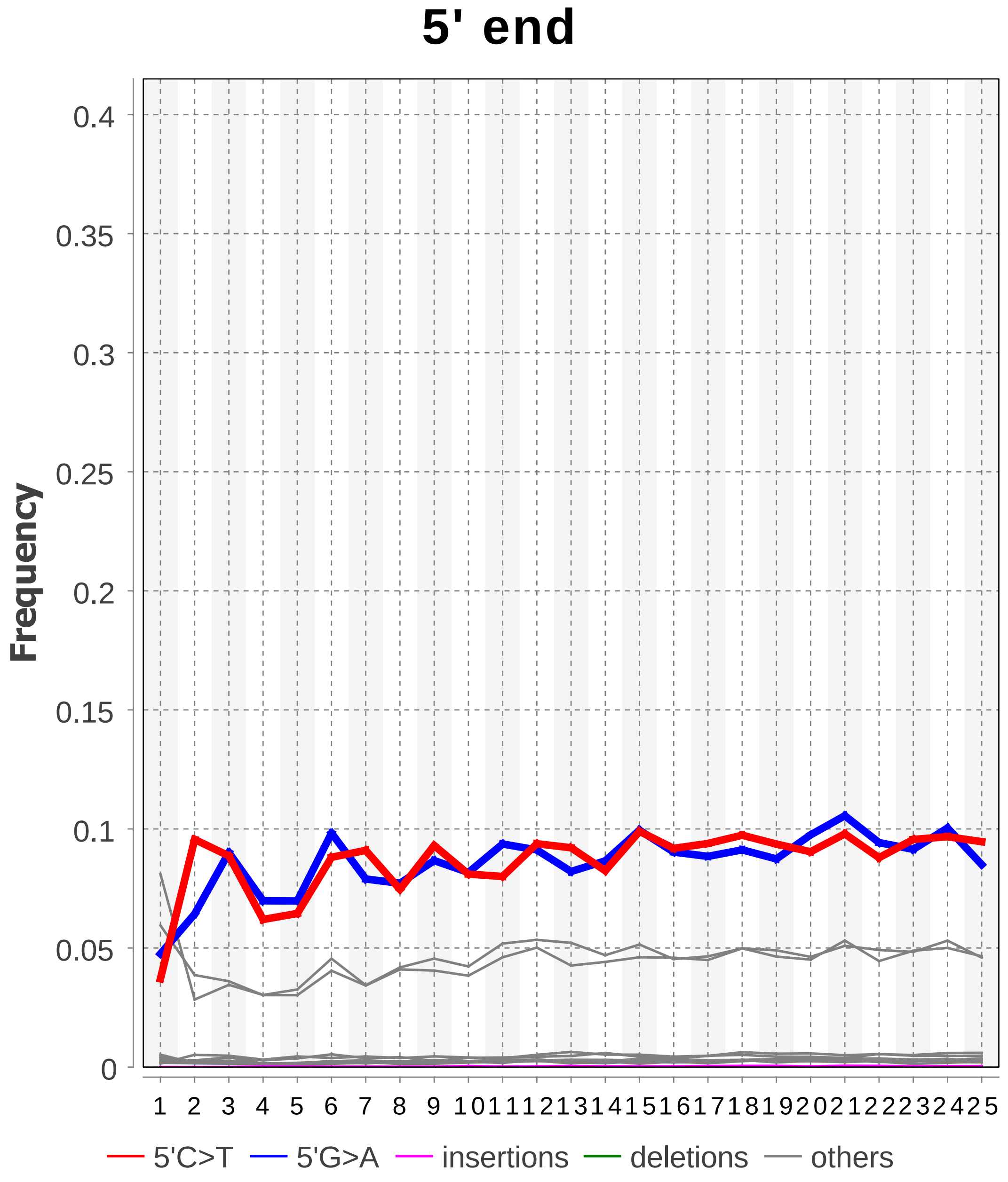

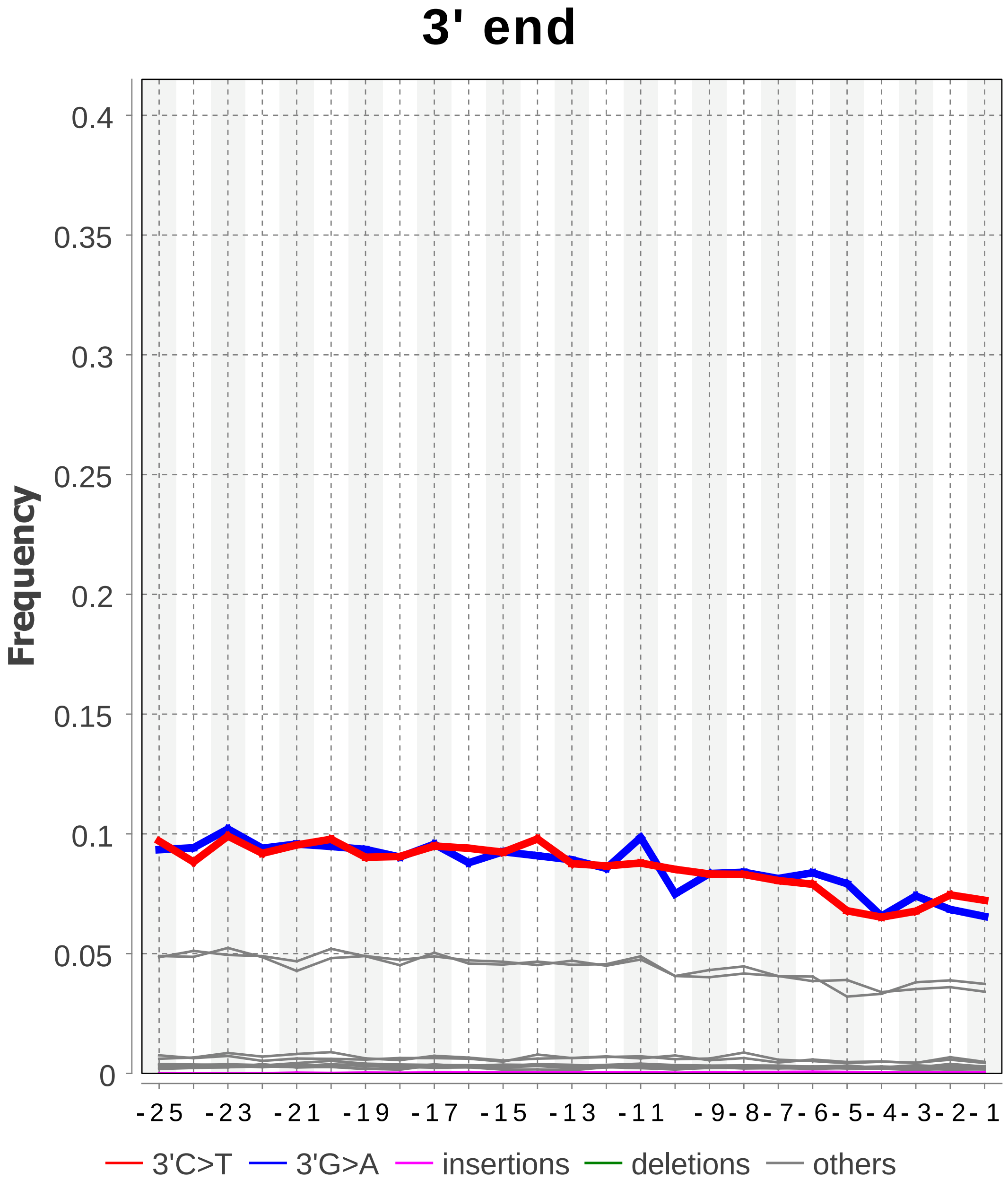

4237_aln

Number of used reads: 52,418 (100.0% of all input reads)

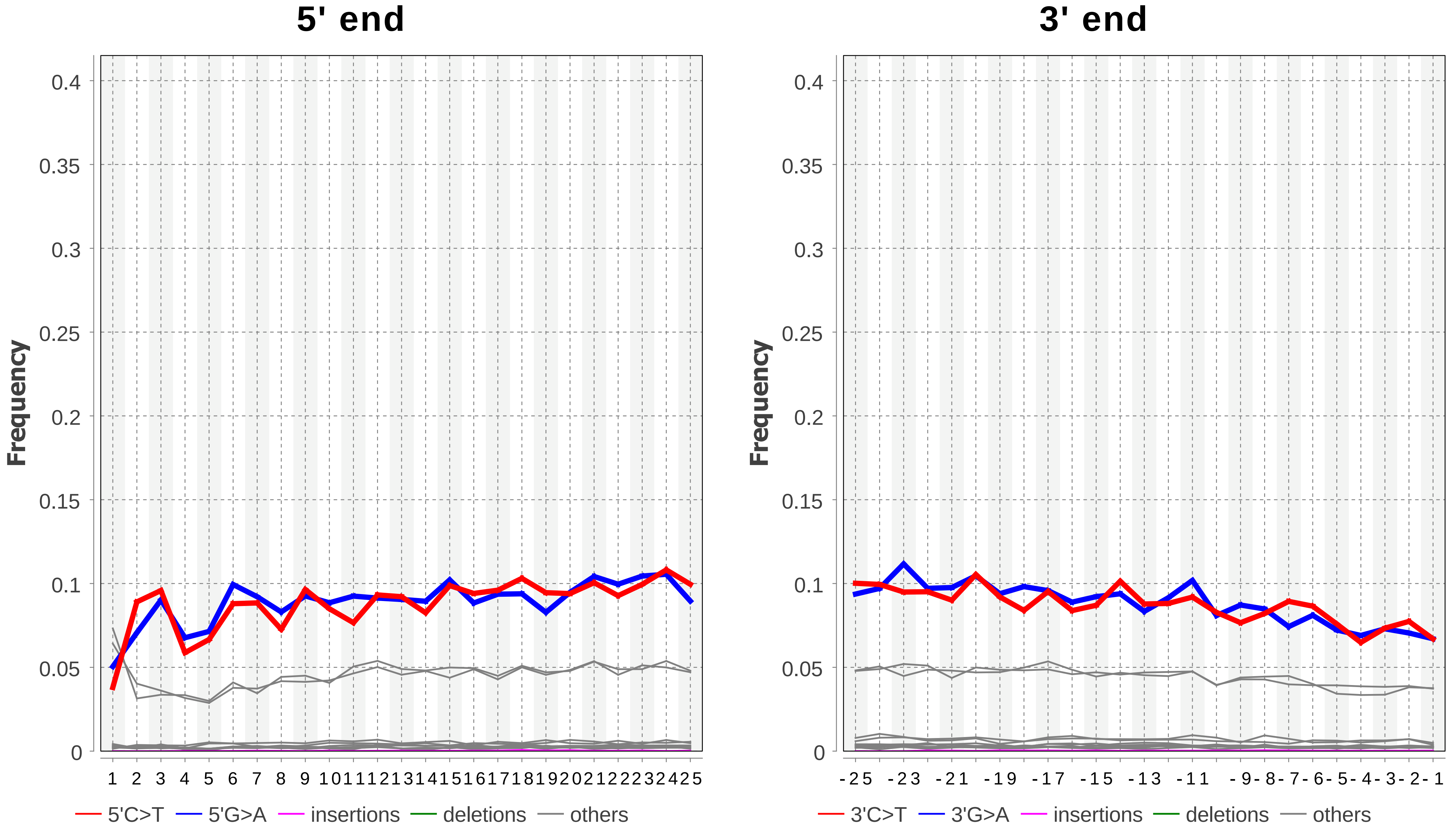

4237_MarkDuplicates

Number of used reads: 44,586 (100.0% of all input reads)

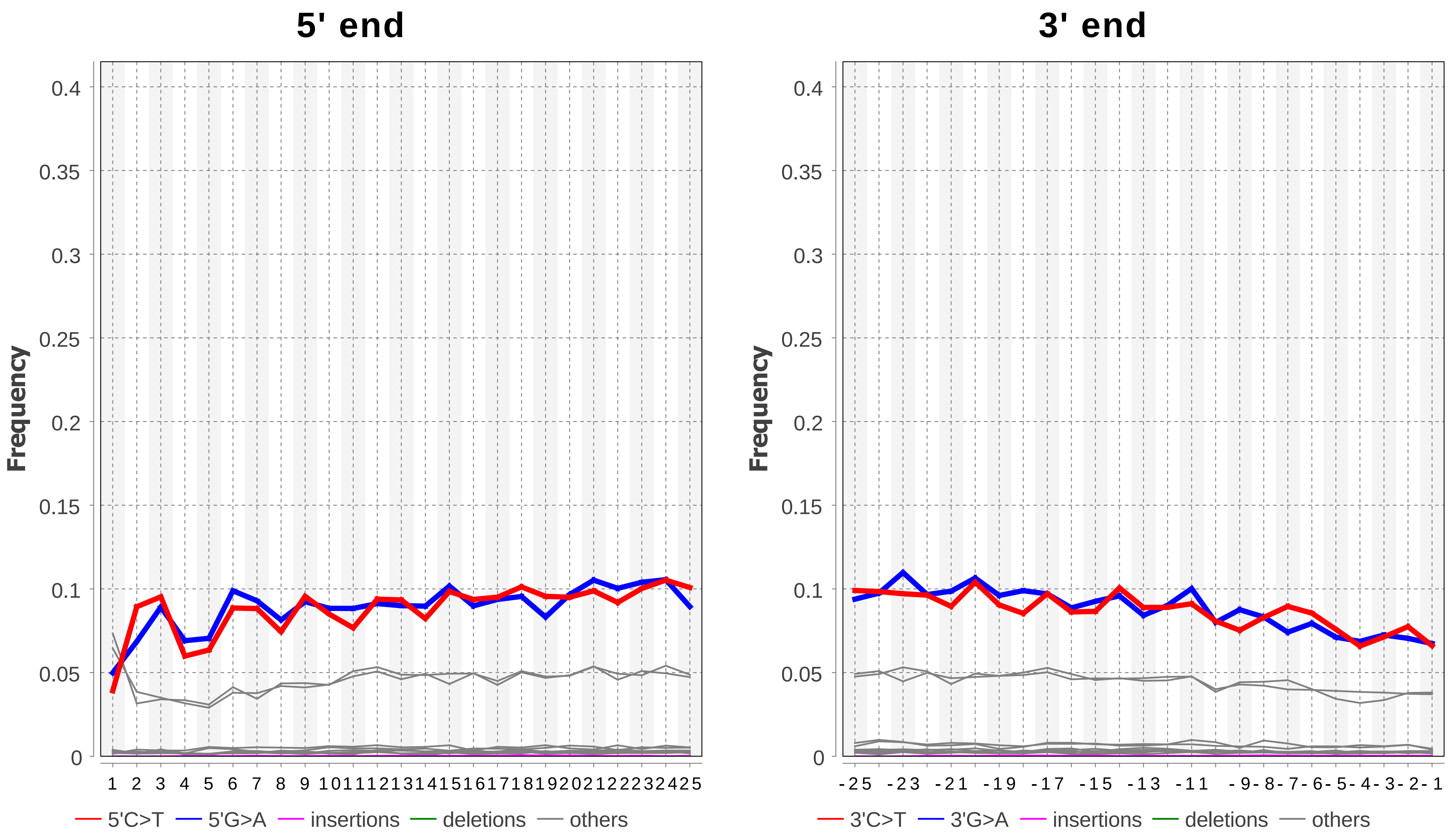

4611_aln

Number of used reads: 299,029 (100.0% of all input reads)

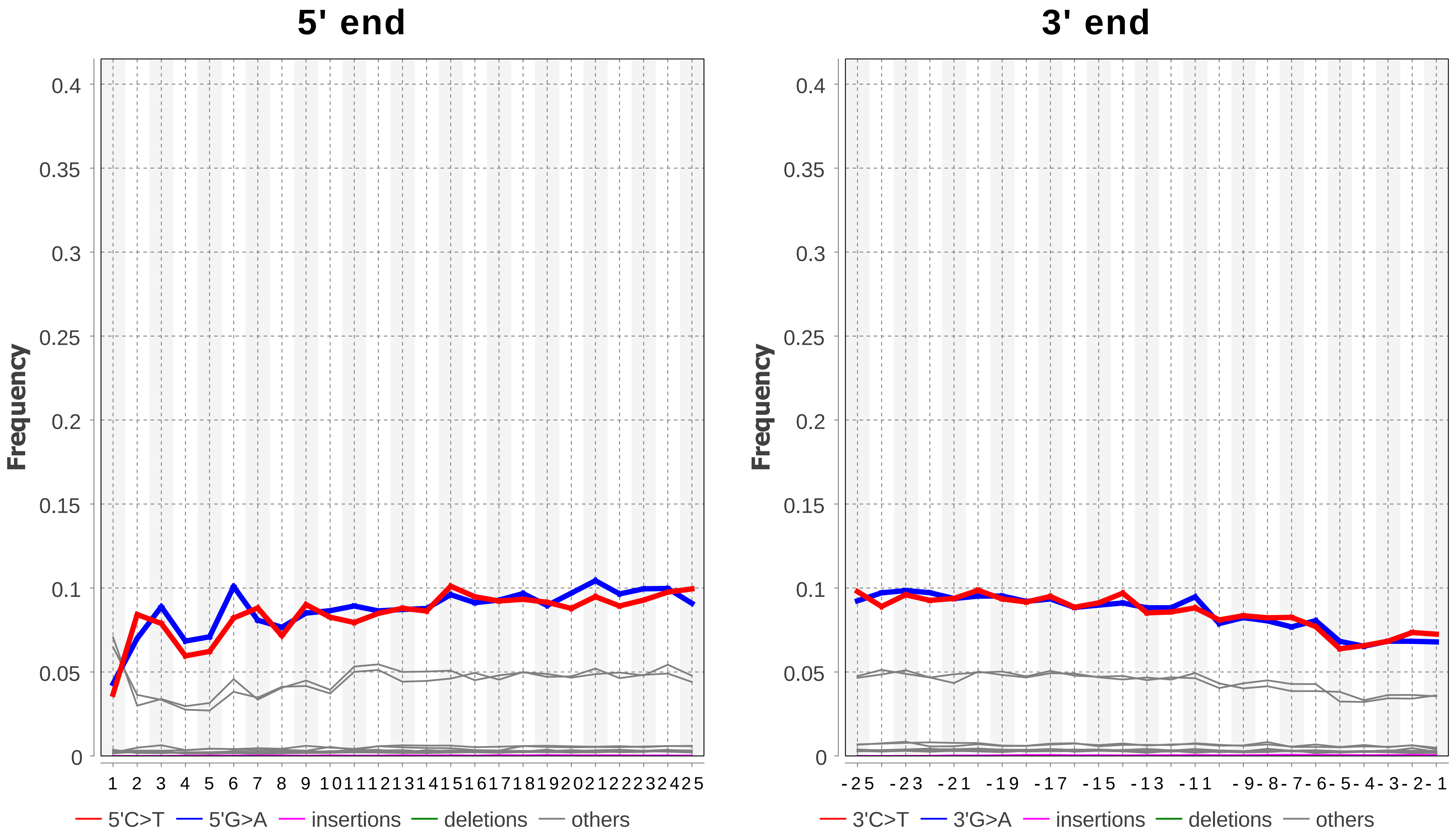

4611_MarkDuplicates
Number of used reads: 216,084 (100.0% of all input reads)

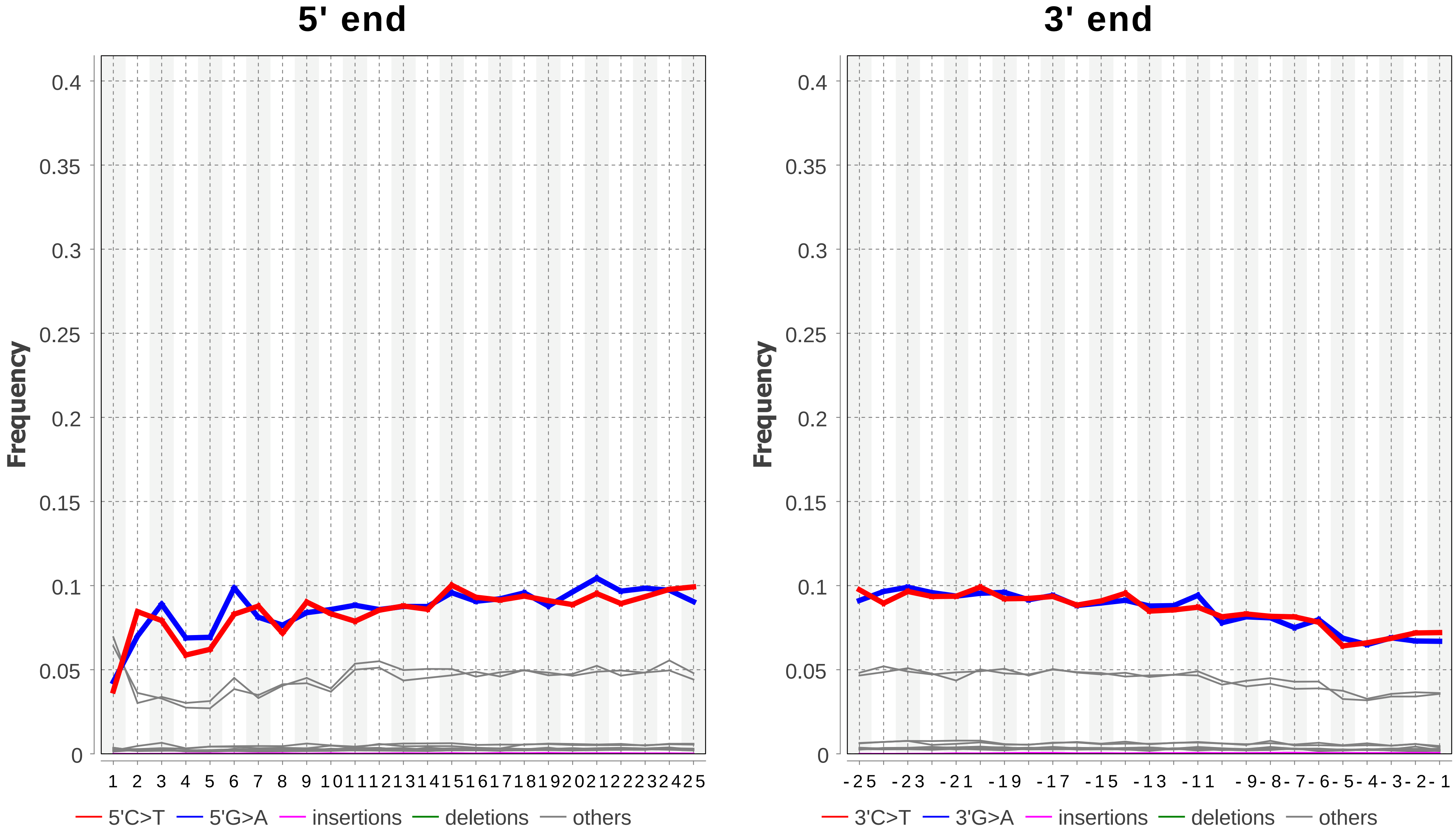

5360_aln
Number of used reads: 124,462 (100.0% of all input reads)

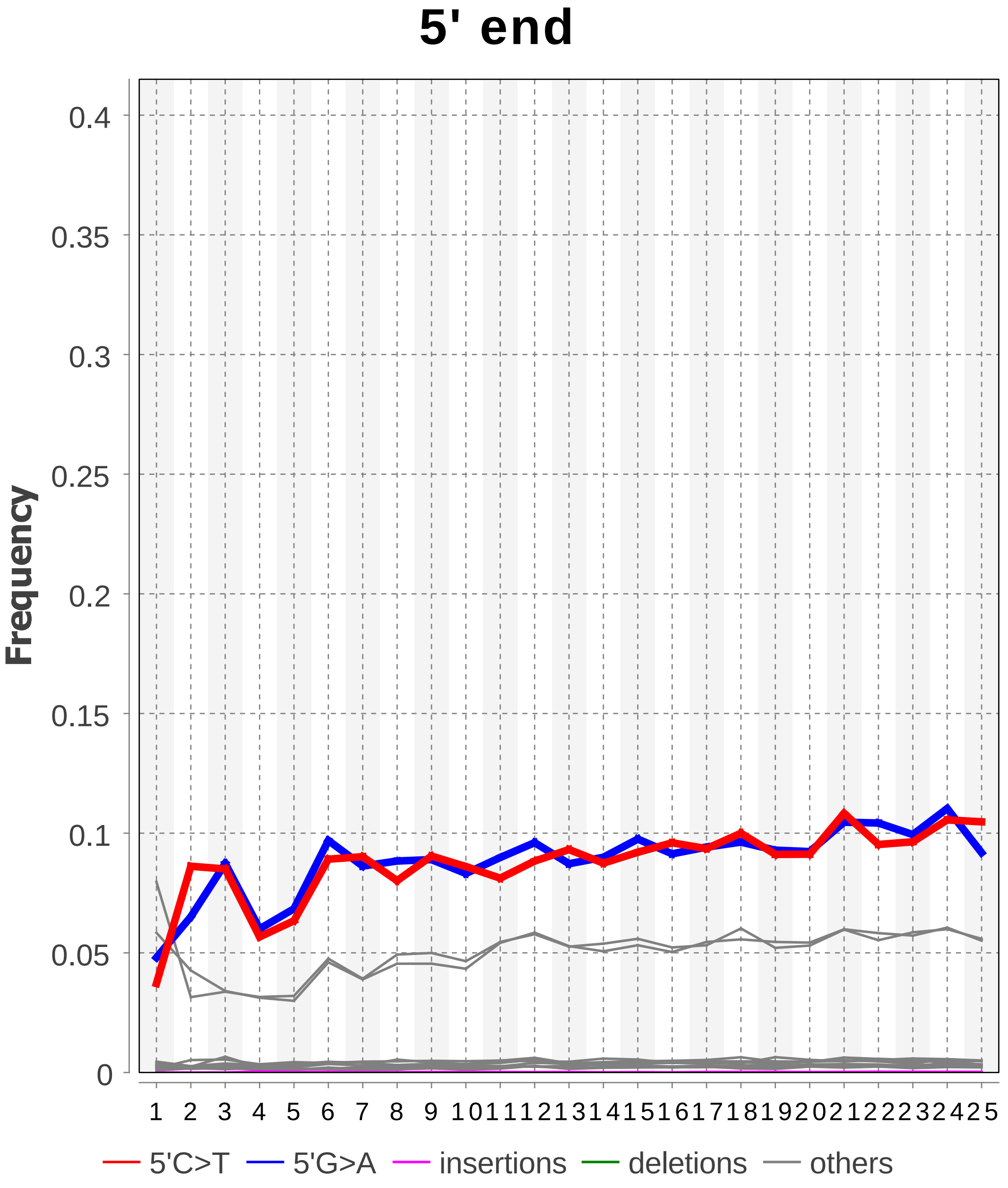

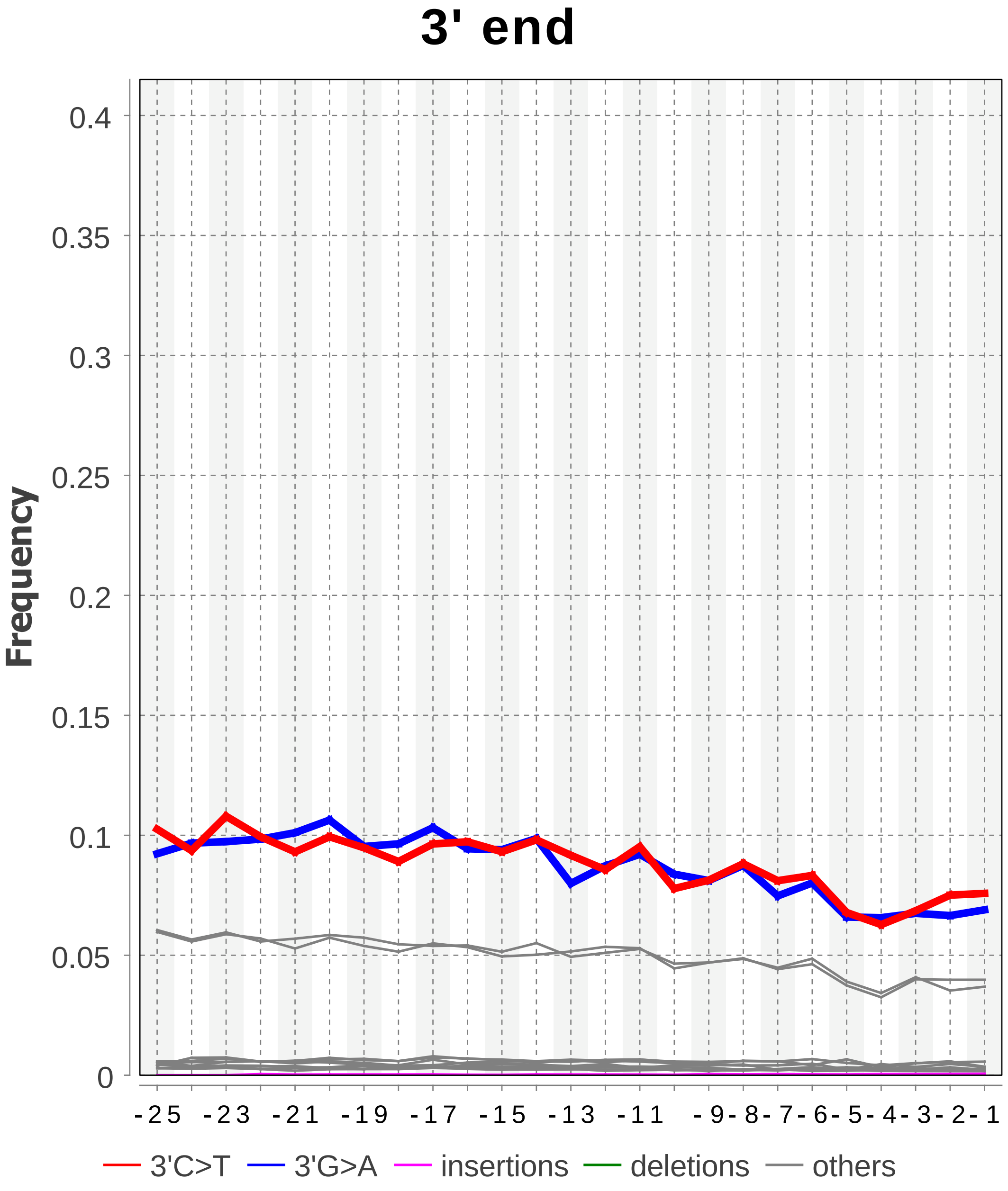

5360_MarkDuplicates
Number of used reads: 101,204 (100.0% of all input reads)

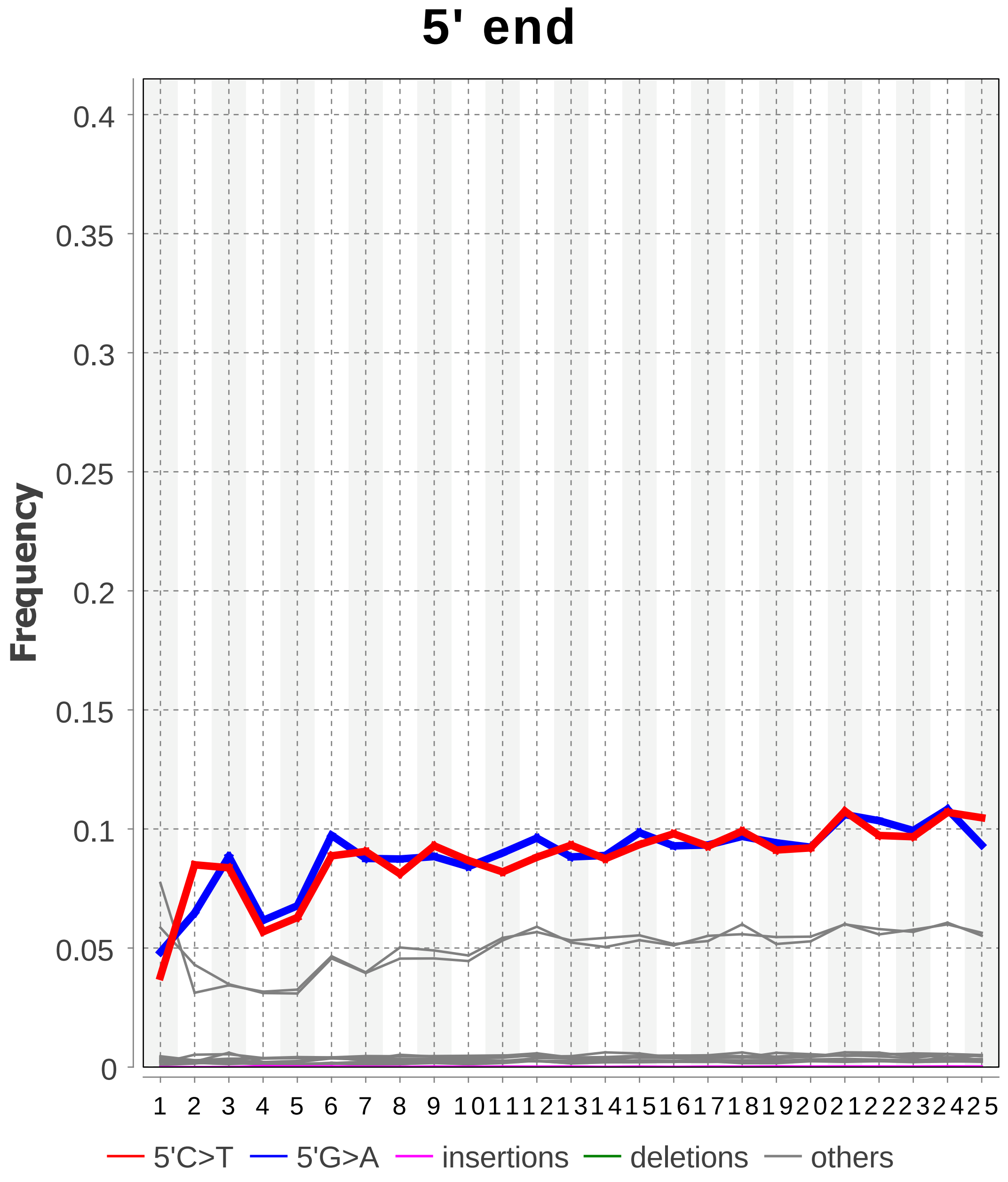

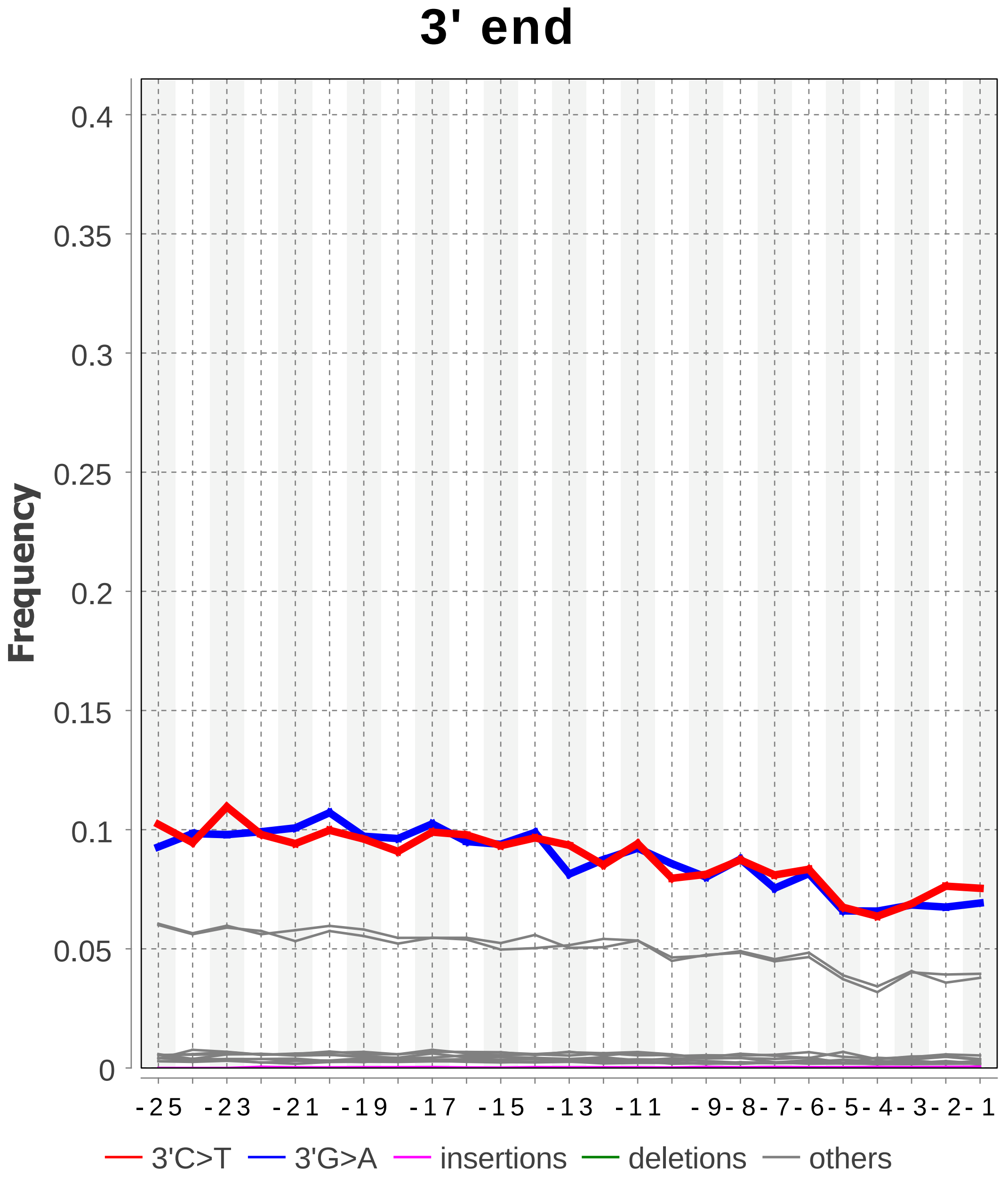

5442_aln

Number of used reads: 100,589 (100.0% of all input reads)

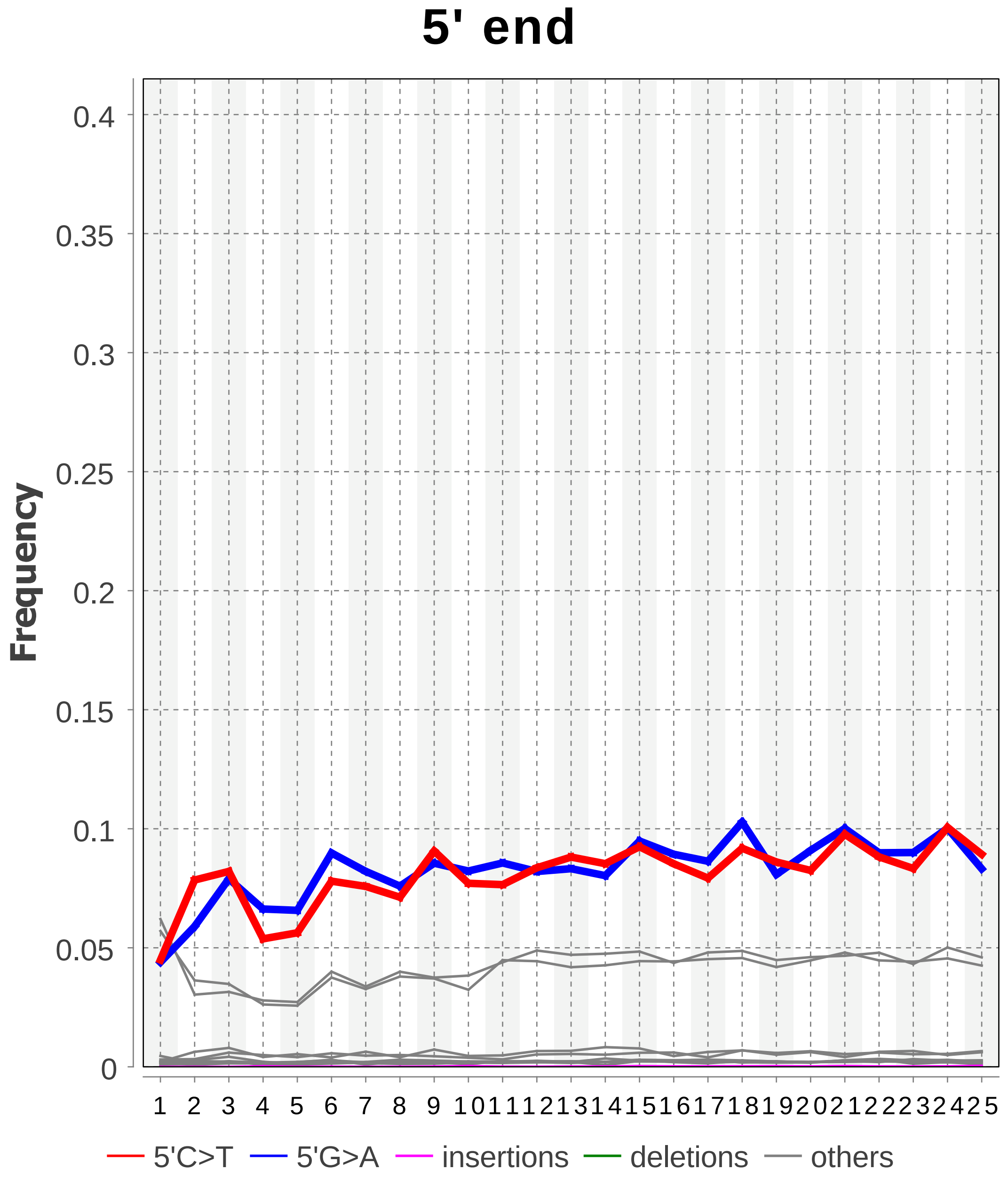

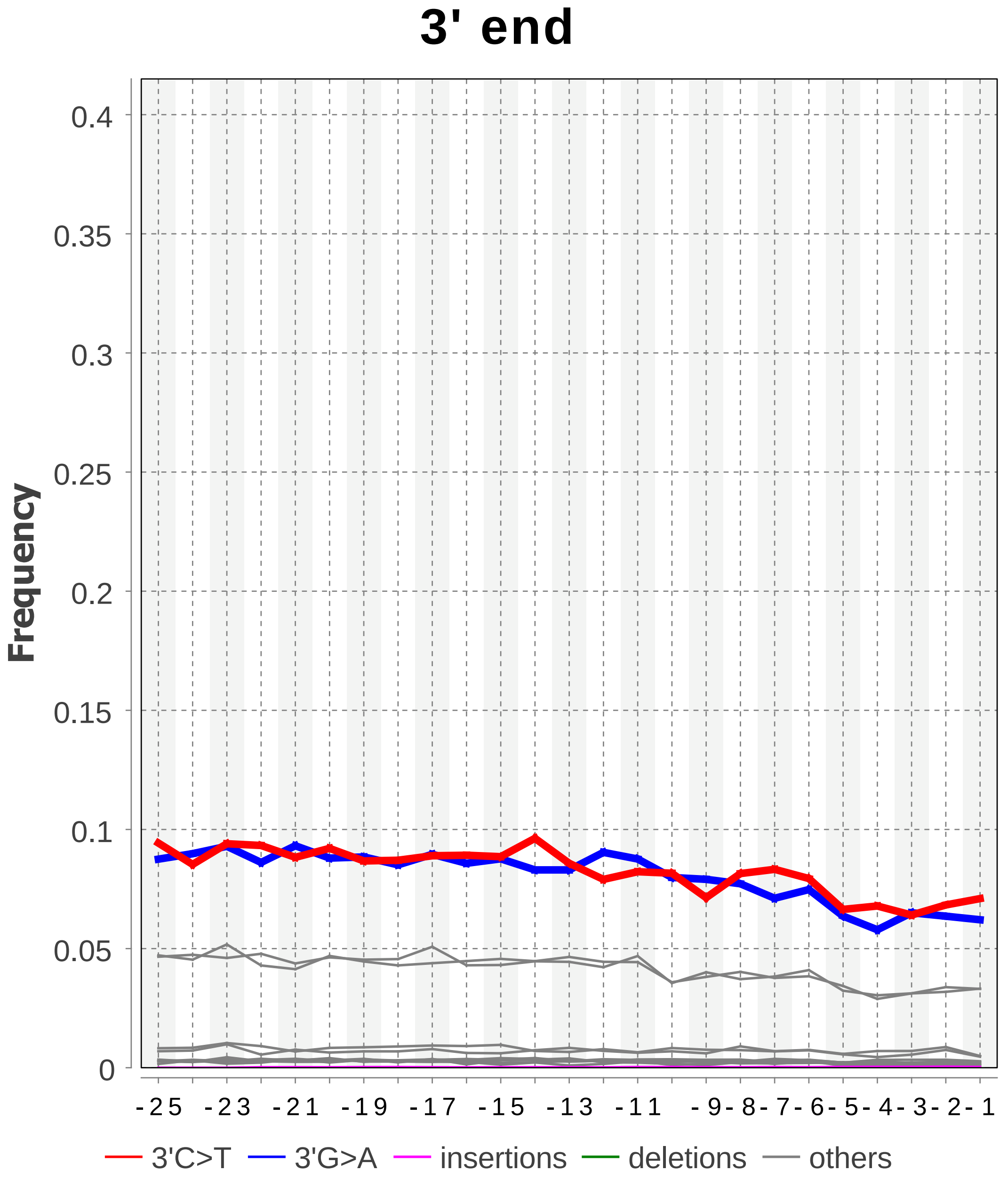

5442_MarkDuplicates
Number of used reads: 82,992 (100.0% of all input reads)

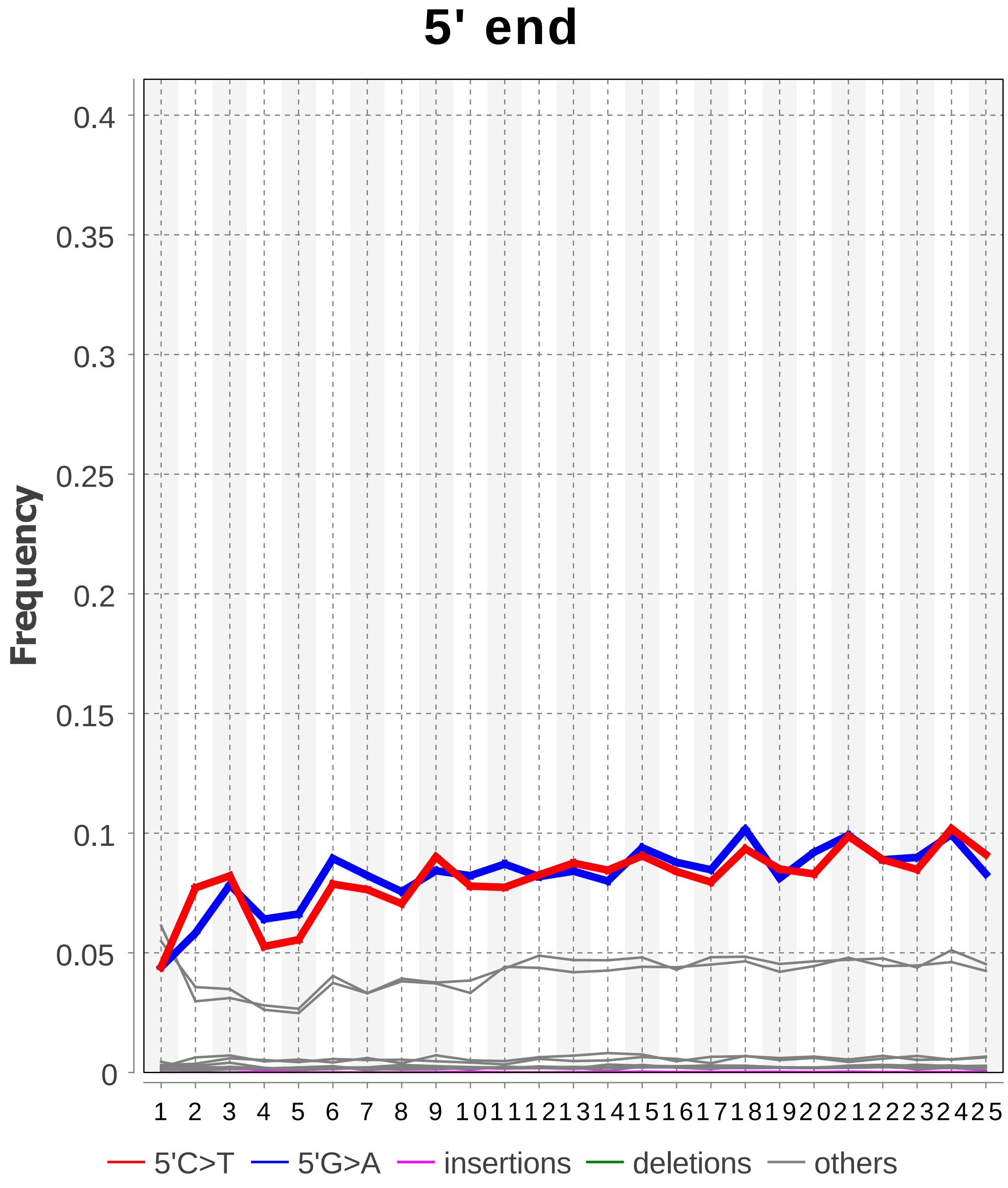

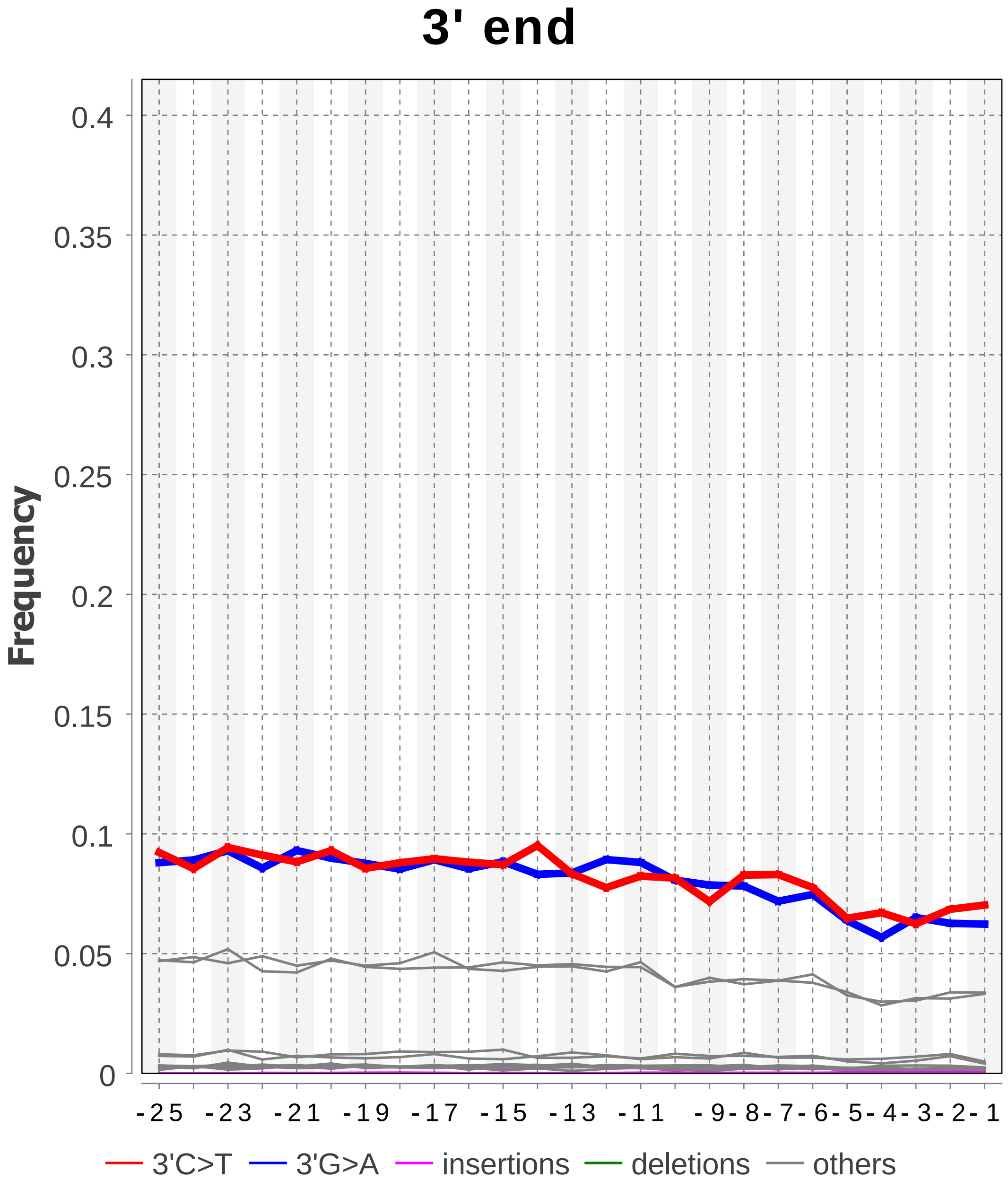

6049_aln

Number of used reads: 72,573 (100.0% of all input reads)

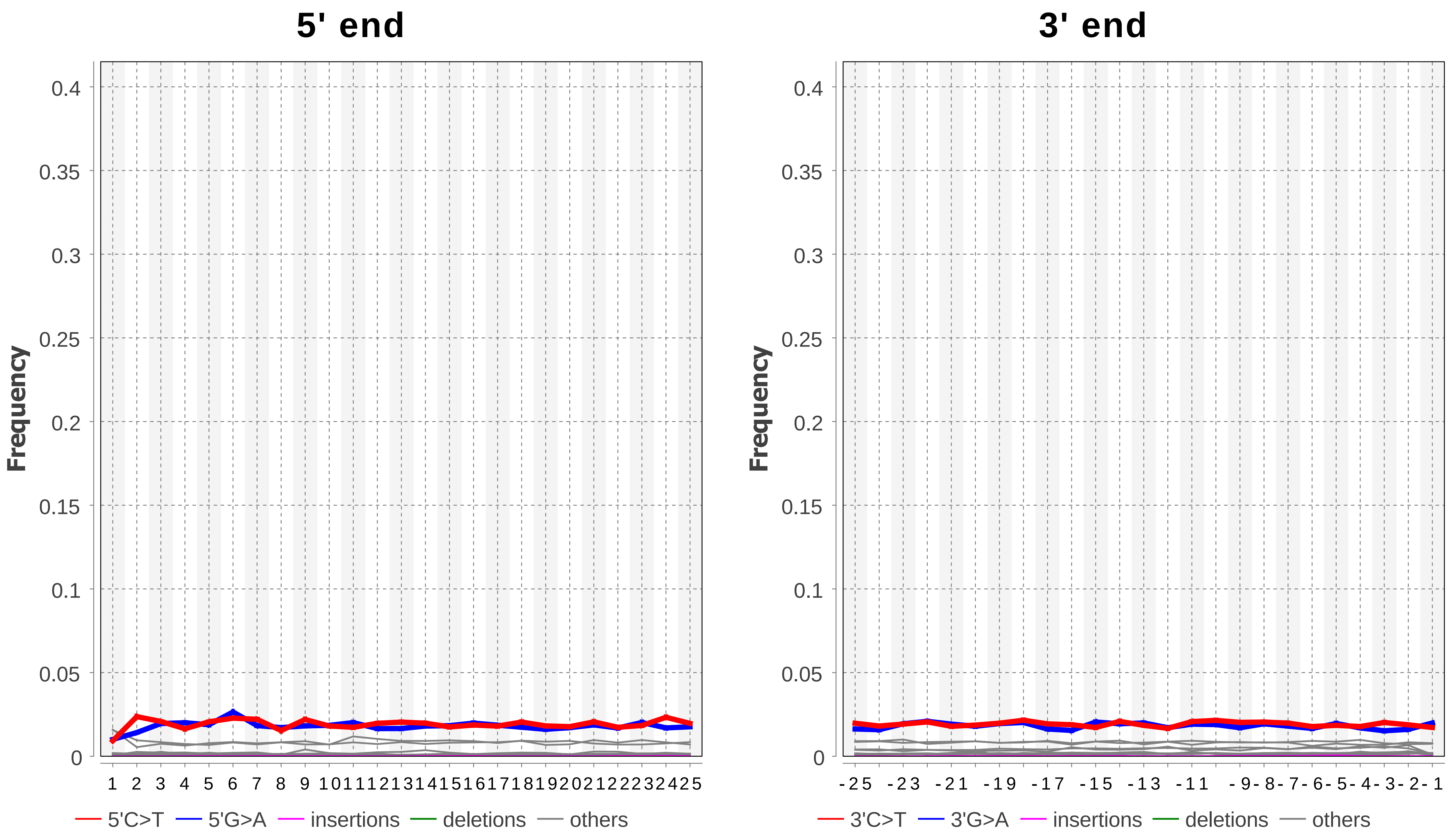

6049_MarkDuplicates
Number of used reads: 62,956 (100.0% of all input reads)

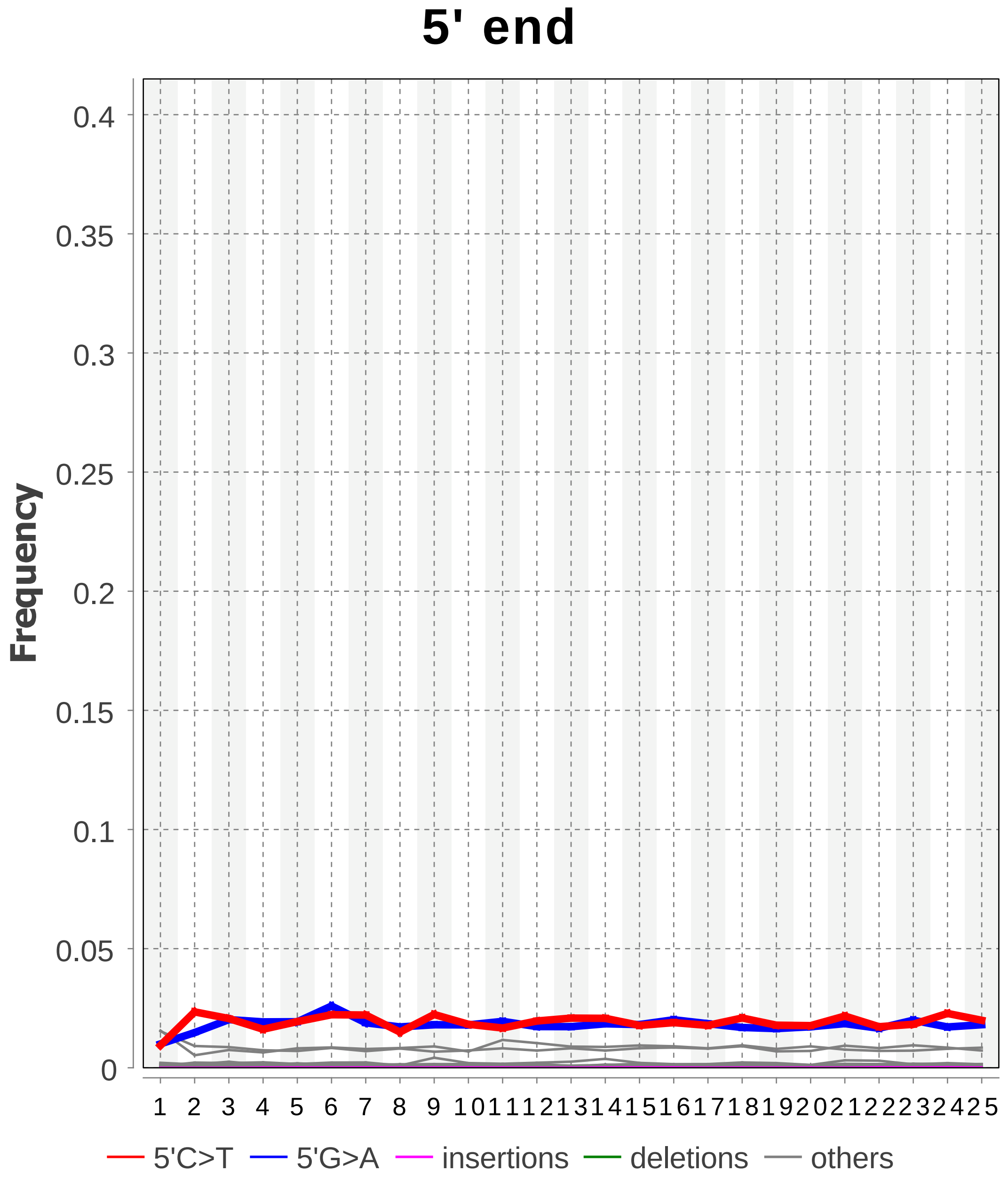

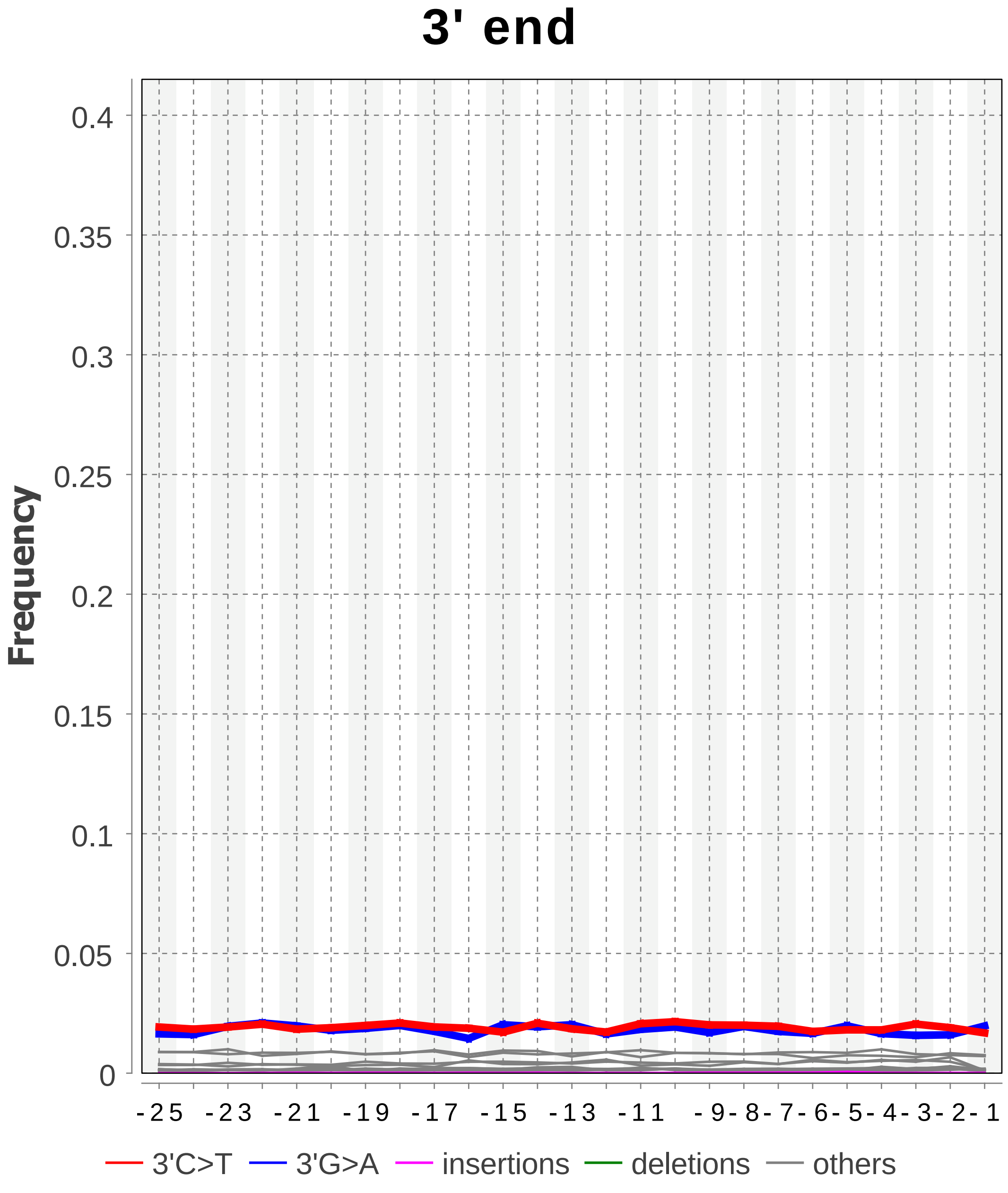

6060_aln

Number of used reads: 37,735 (100.0% of all input reads)

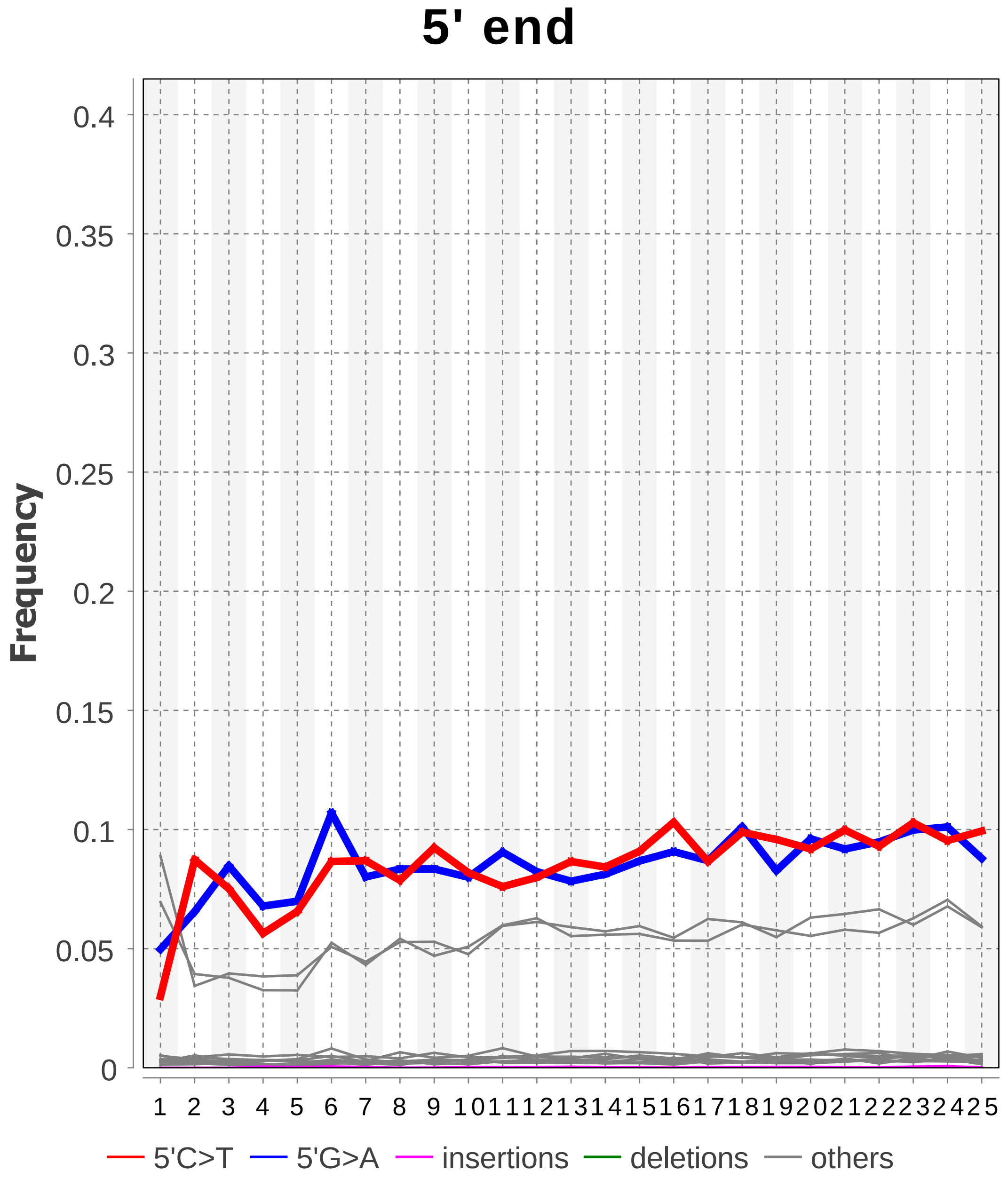

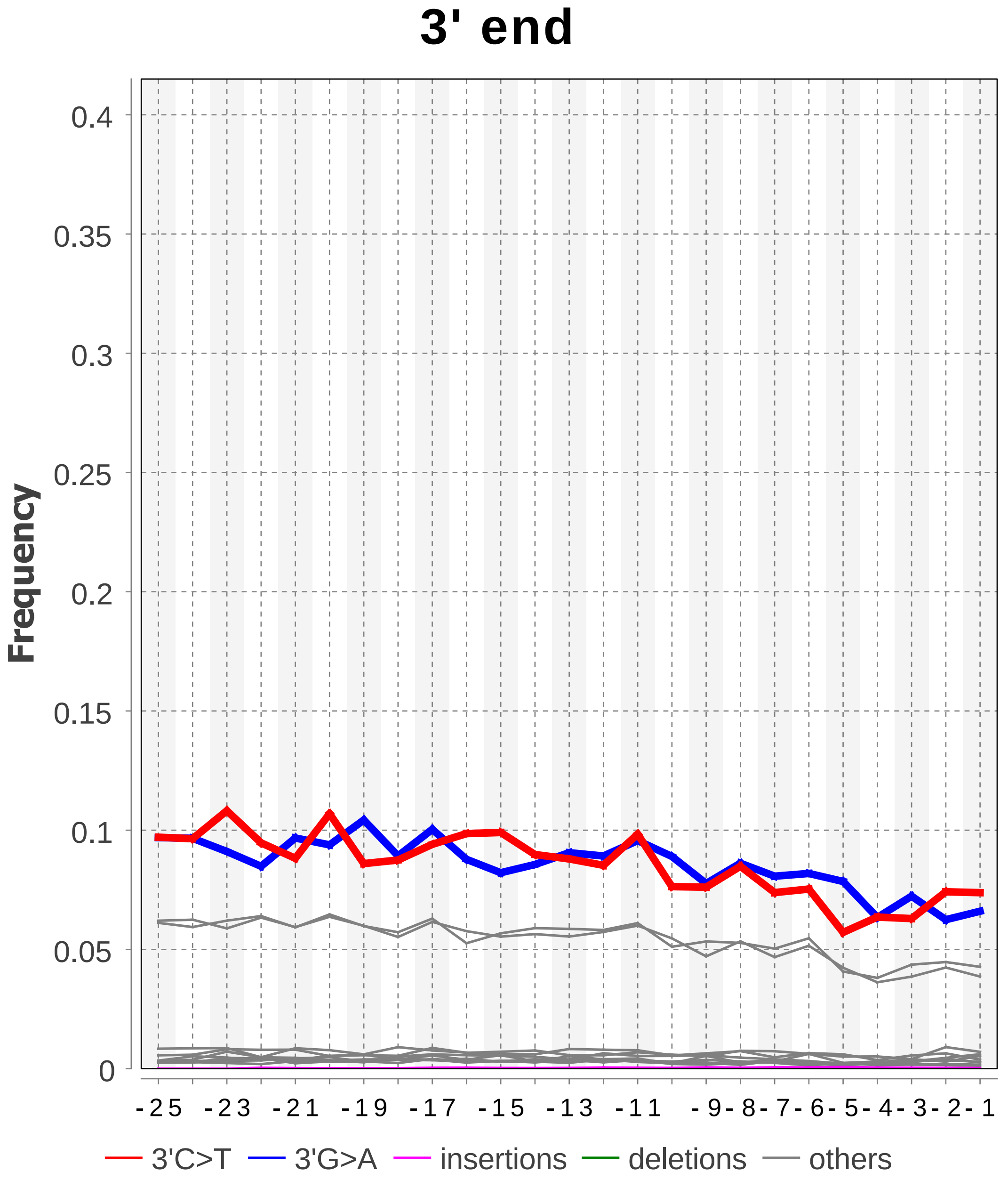

6060_MarkDuplicates
Number of used reads: 32,180 (100.0% of all input reads)

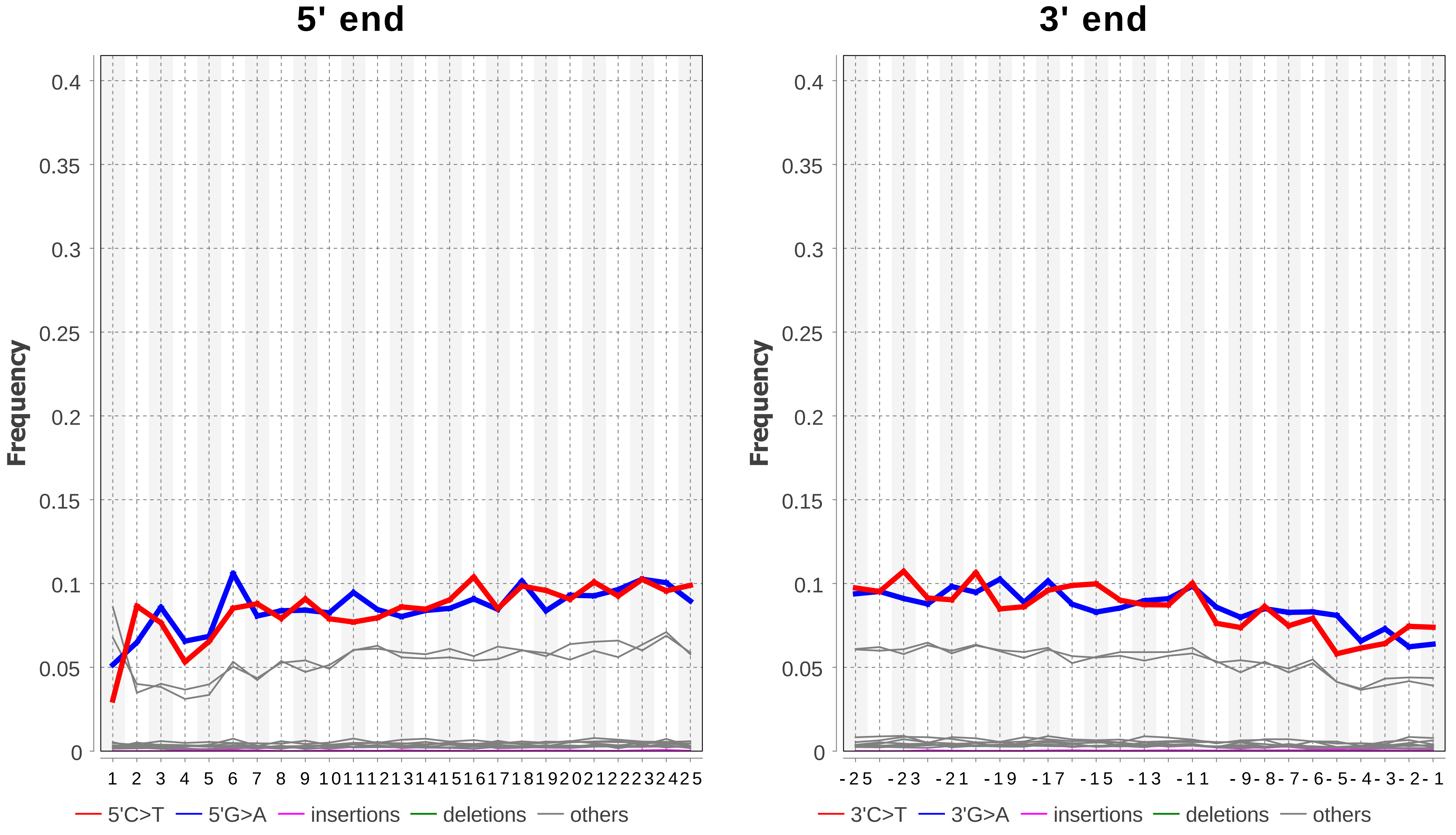

6076_aln
Number of used reads: 38,889 (100.0% of all input reads)

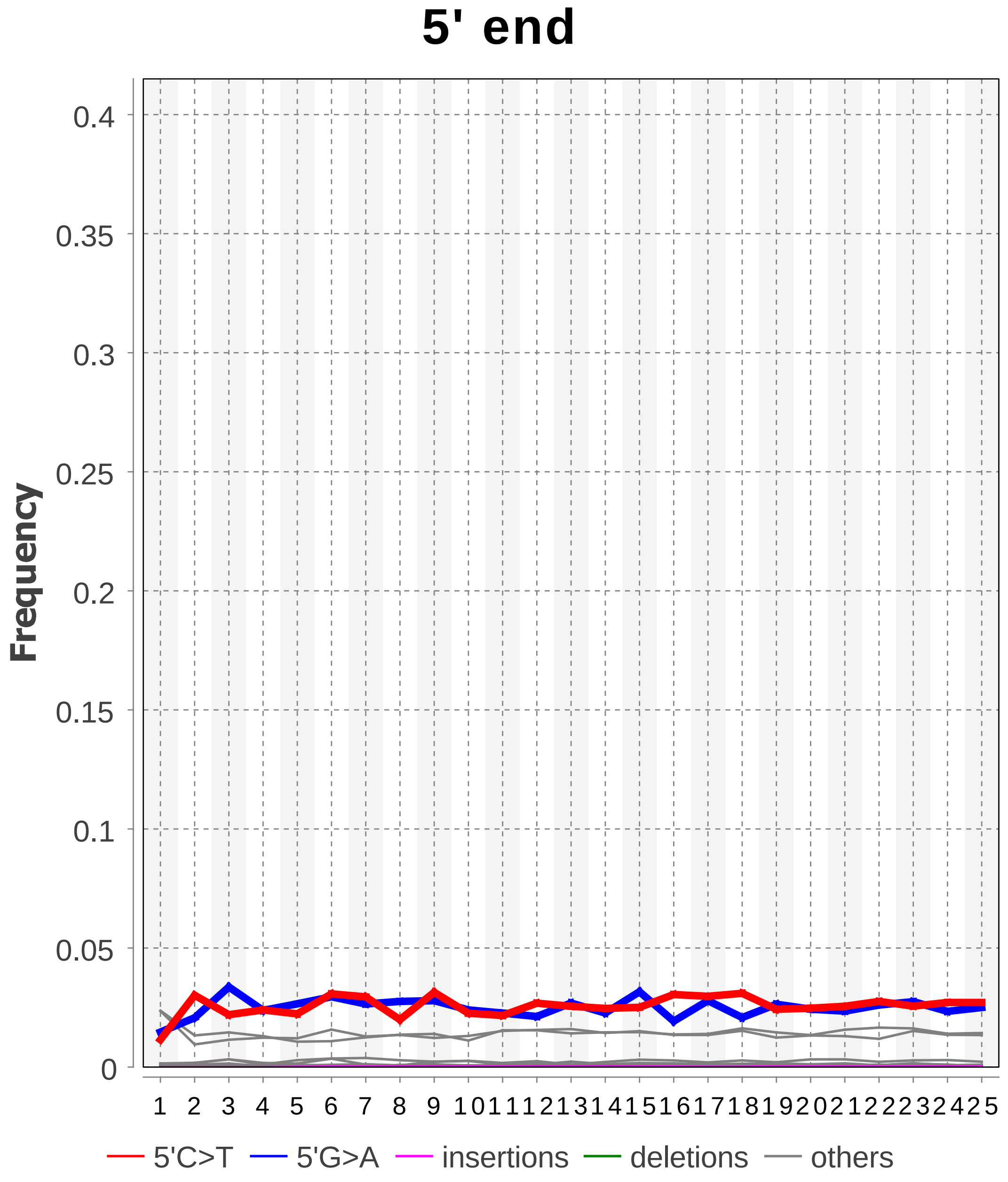

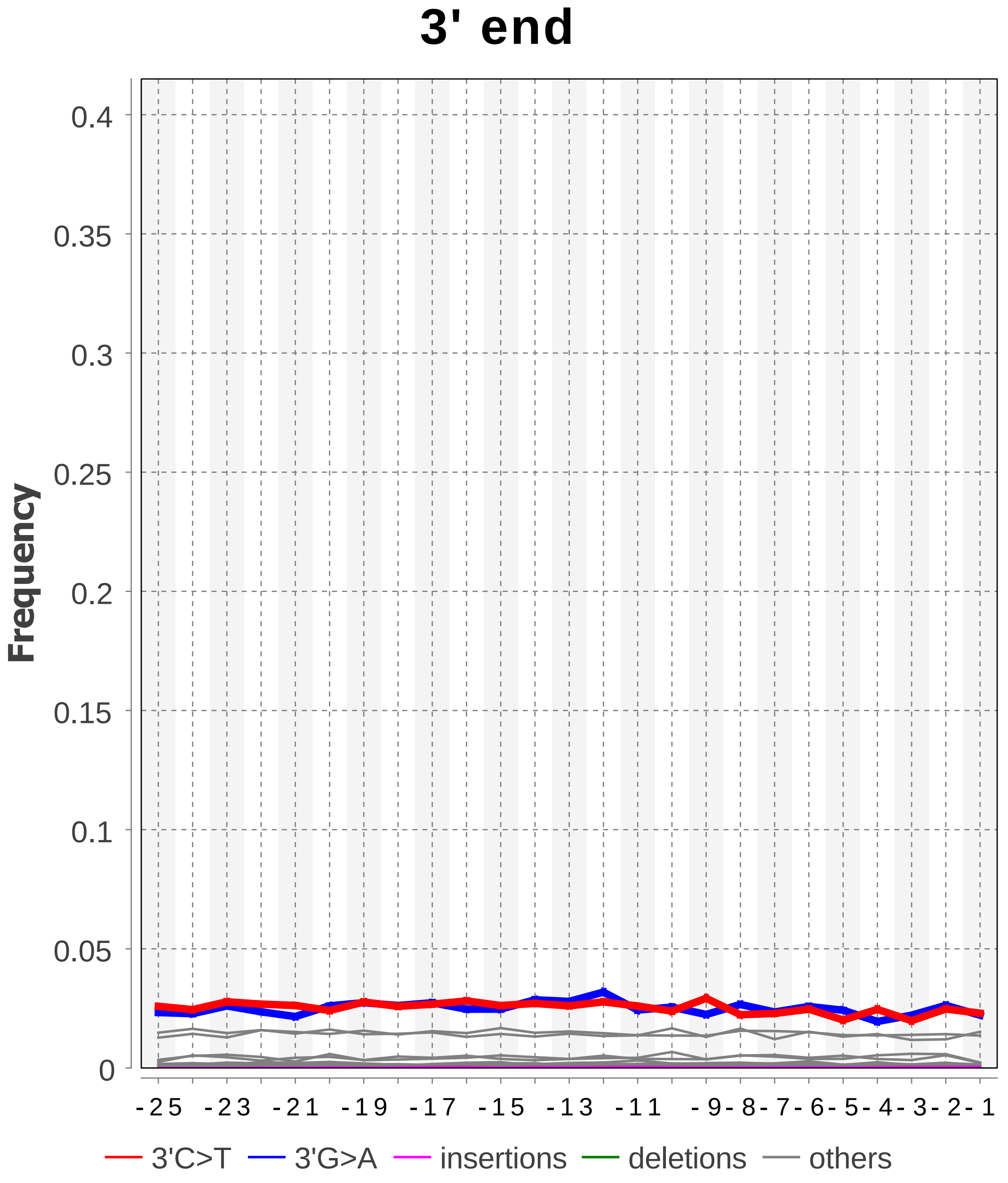

6076_MarkDuplicates
Number of used reads: 33,987 (100.0% of all input reads)

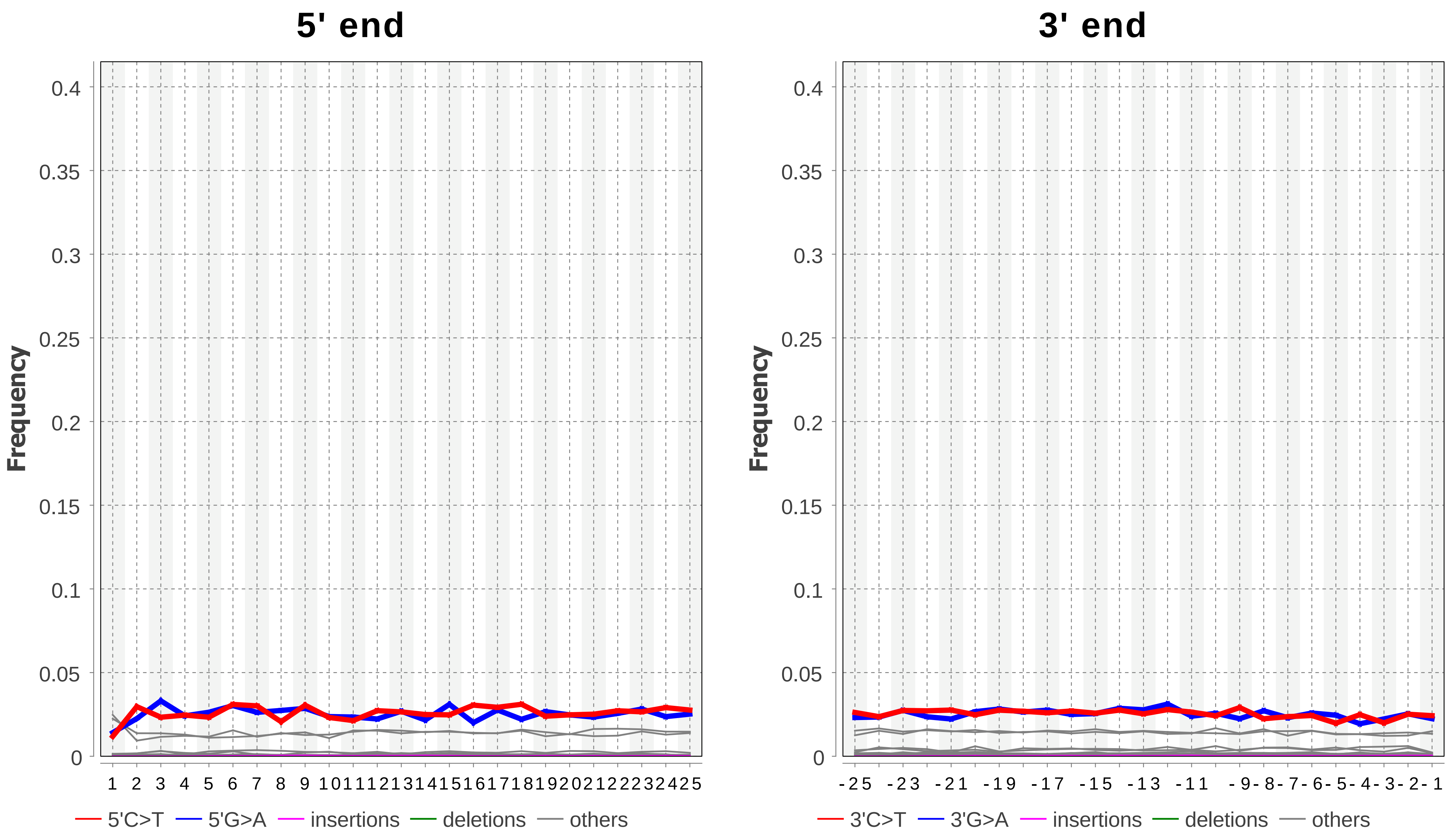

6084_aln

Number of used reads: 91,682 (100.0% of all input reads)

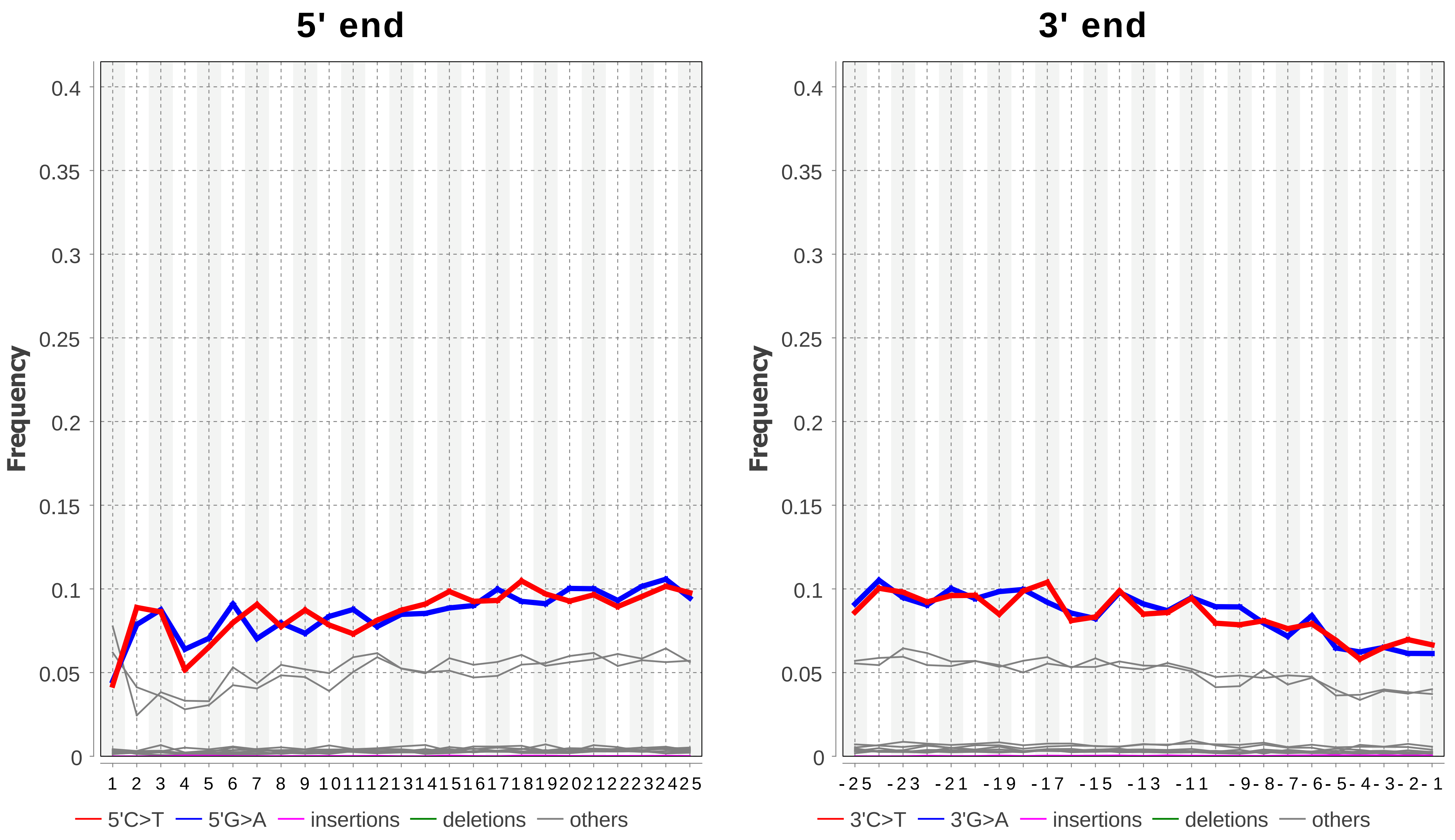

6084_MarkDuplicates

Number of used reads: 44,634 (100.0% of all input reads)

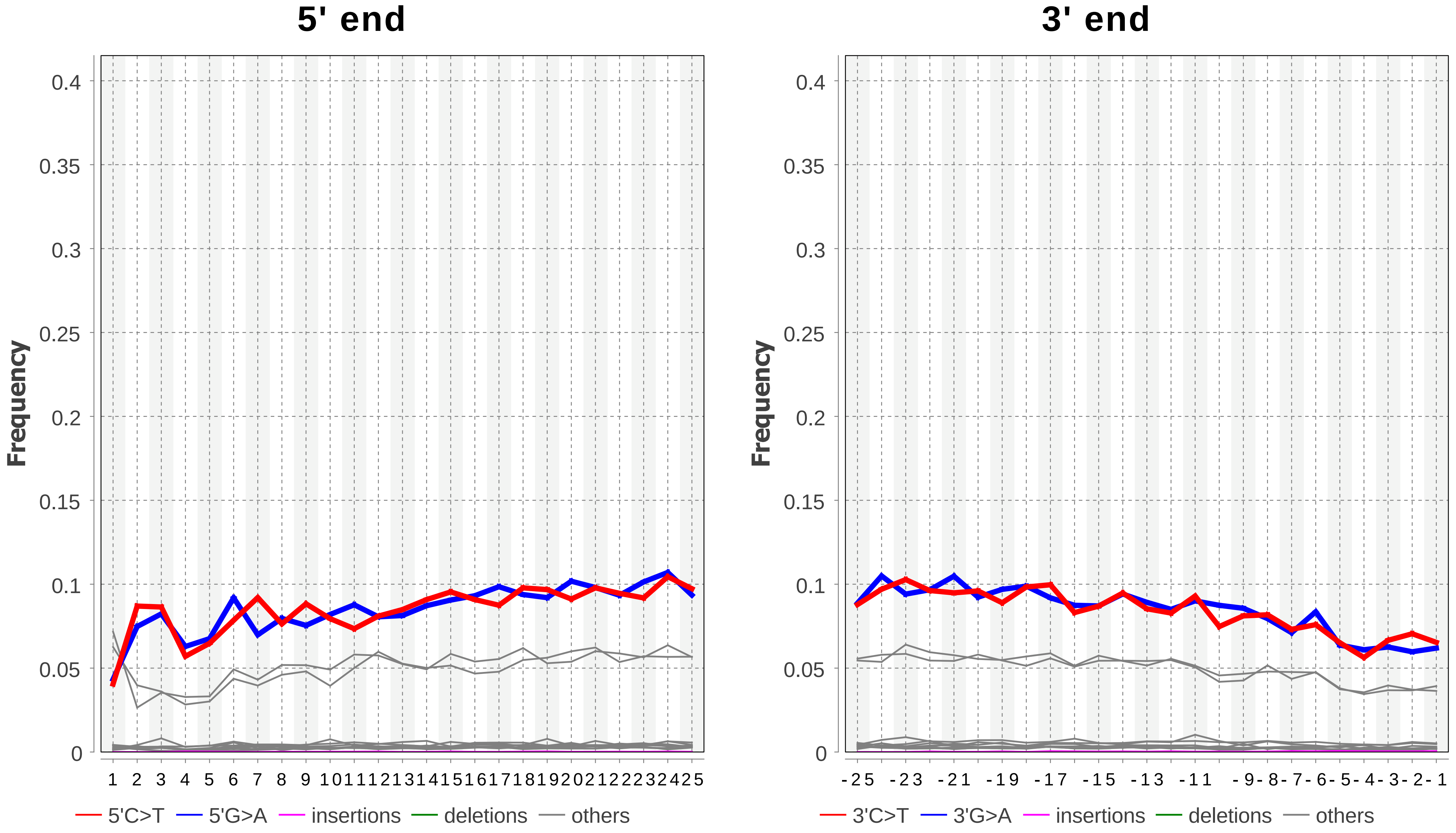

6305_aln
Number of used reads: 86,133 (100.0% of all input reads)

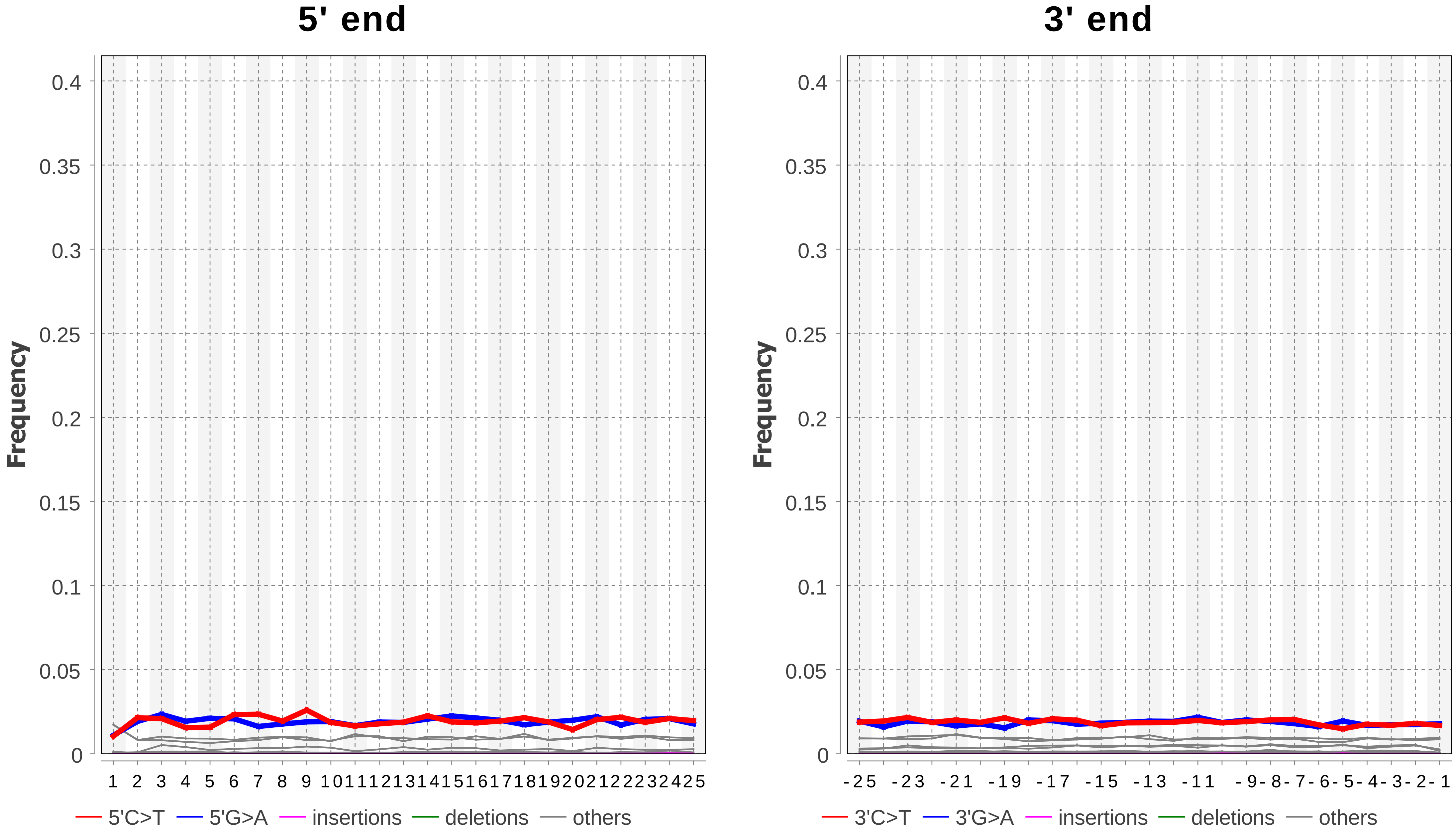

6305_MarkDuplicates

Number of used reads: 68,964 (100.0% of all input reads)

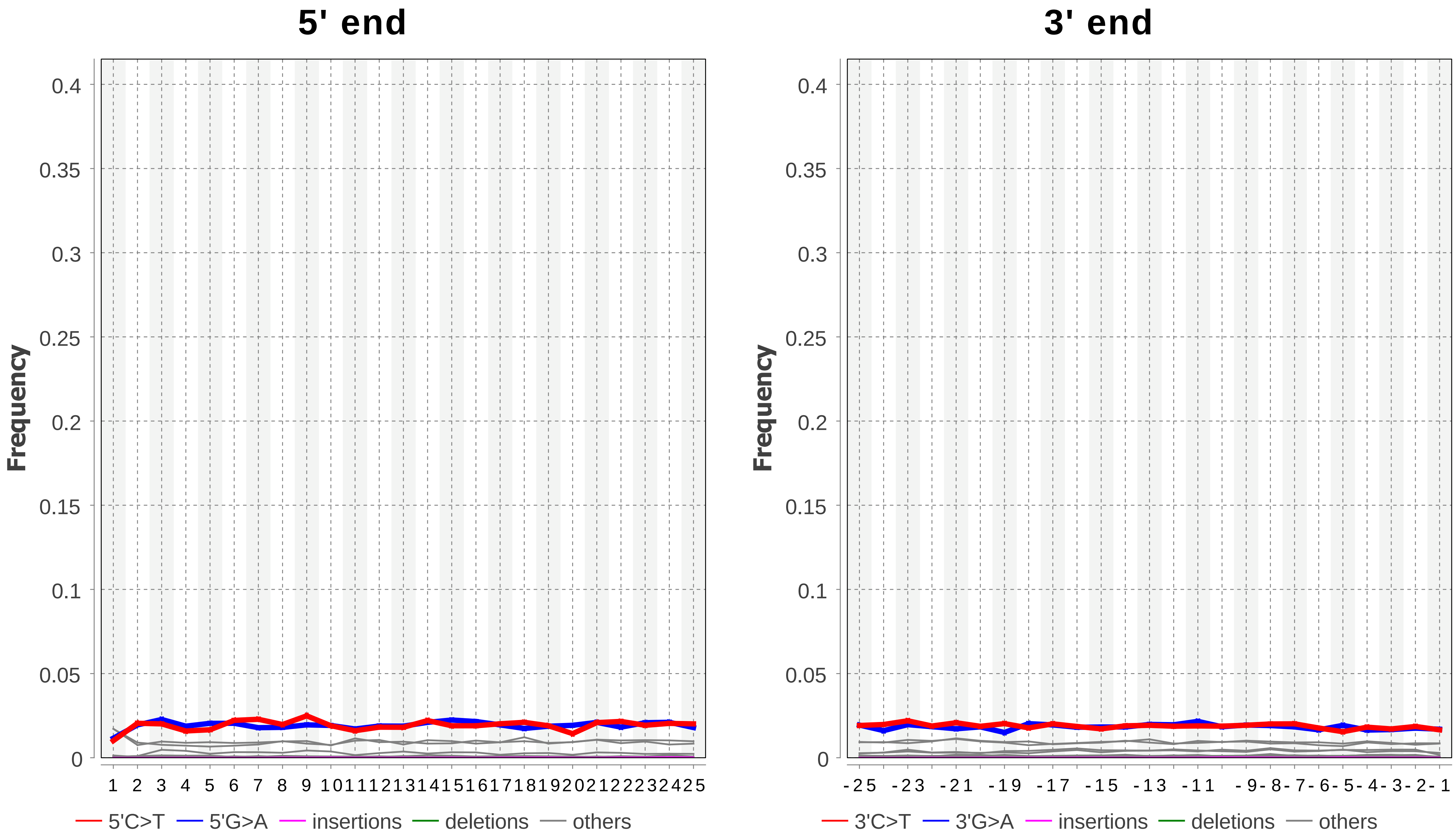

6317_aln
Number of used reads: 33,408 (100.0% of all input reads)

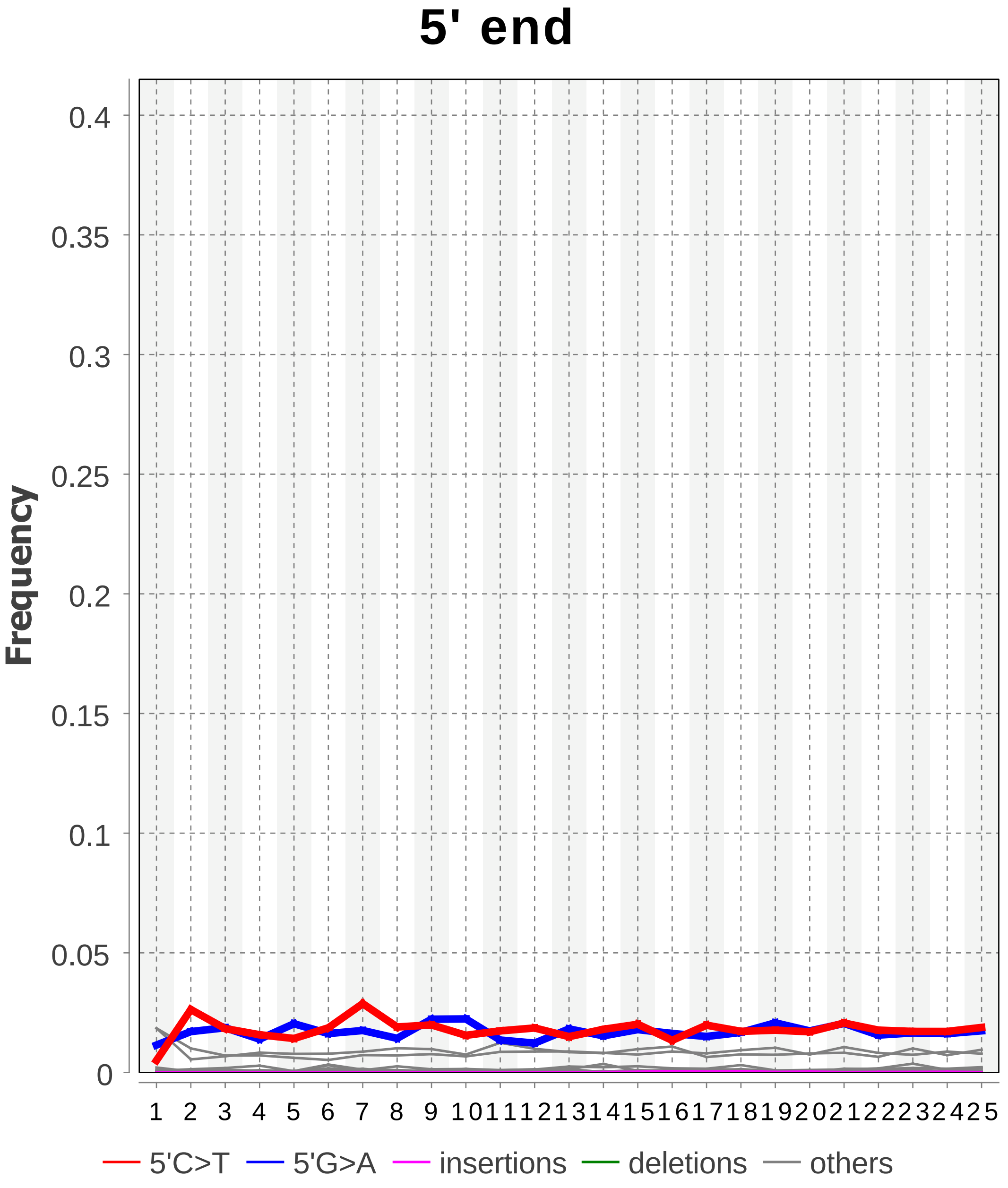

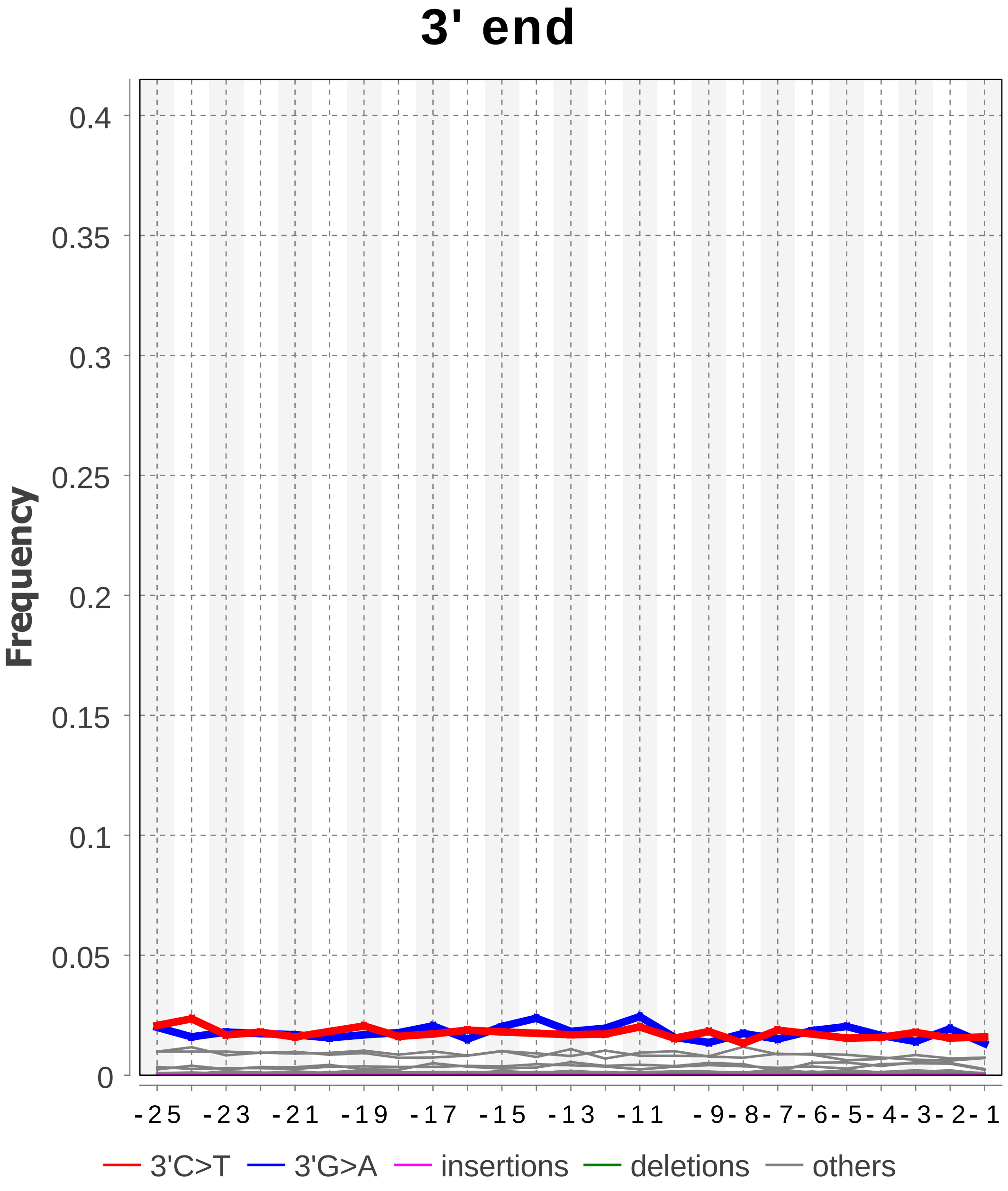

6317_MarkDuplicates
Number of used reads: 26,718 (100.0% of all input reads)

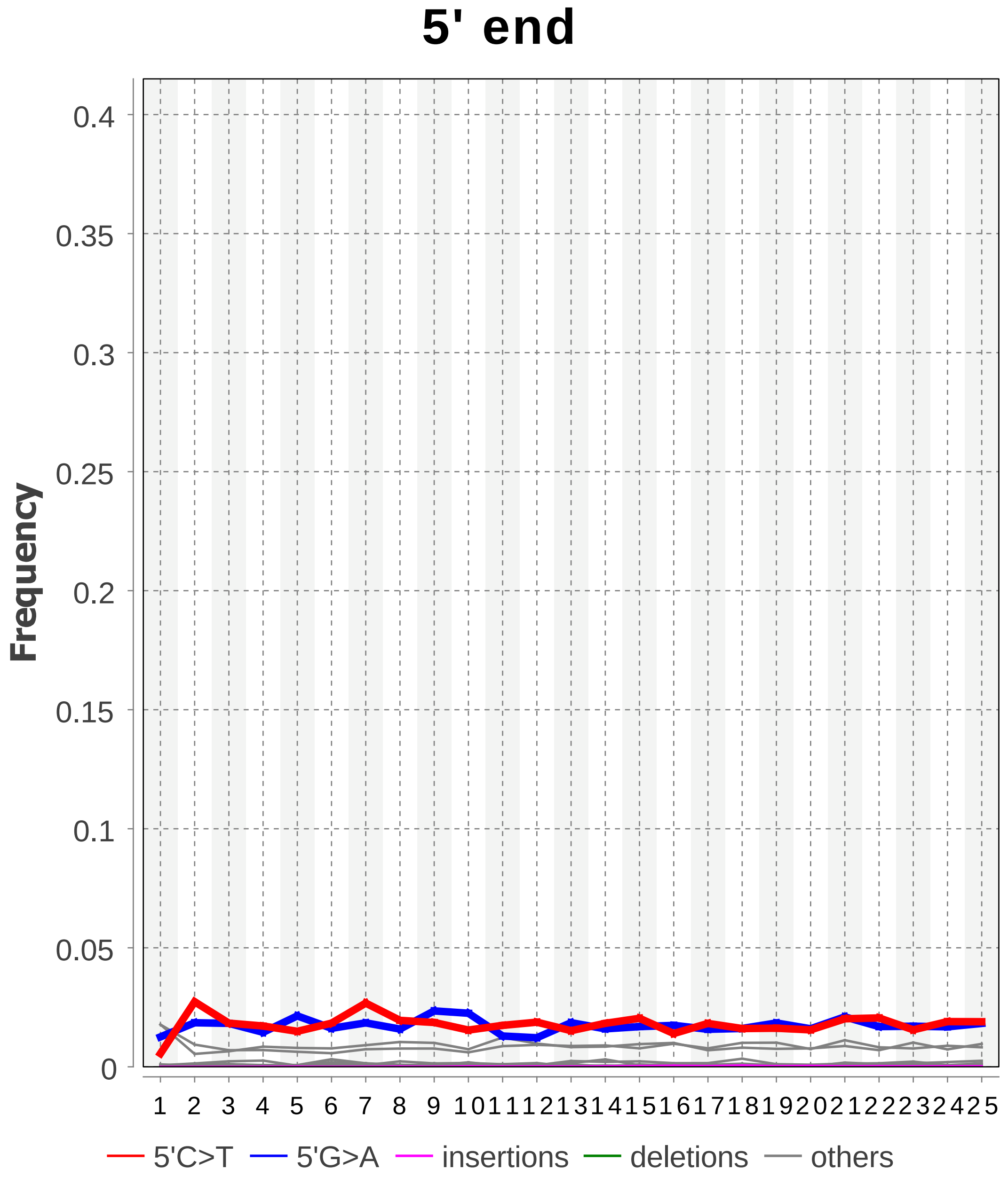

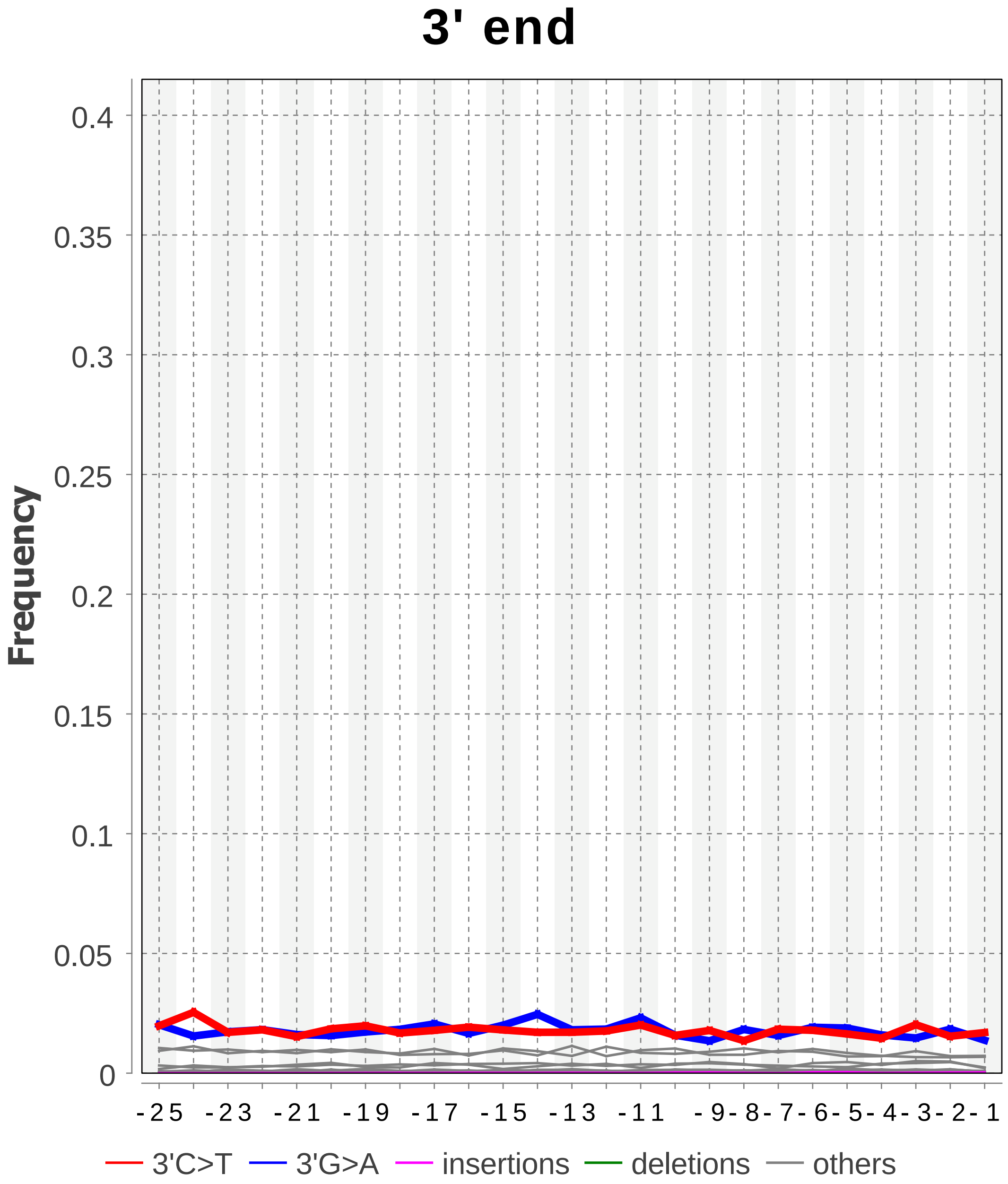

6733_aln

Number of used reads: 106,615 (100.0% of all input reads)

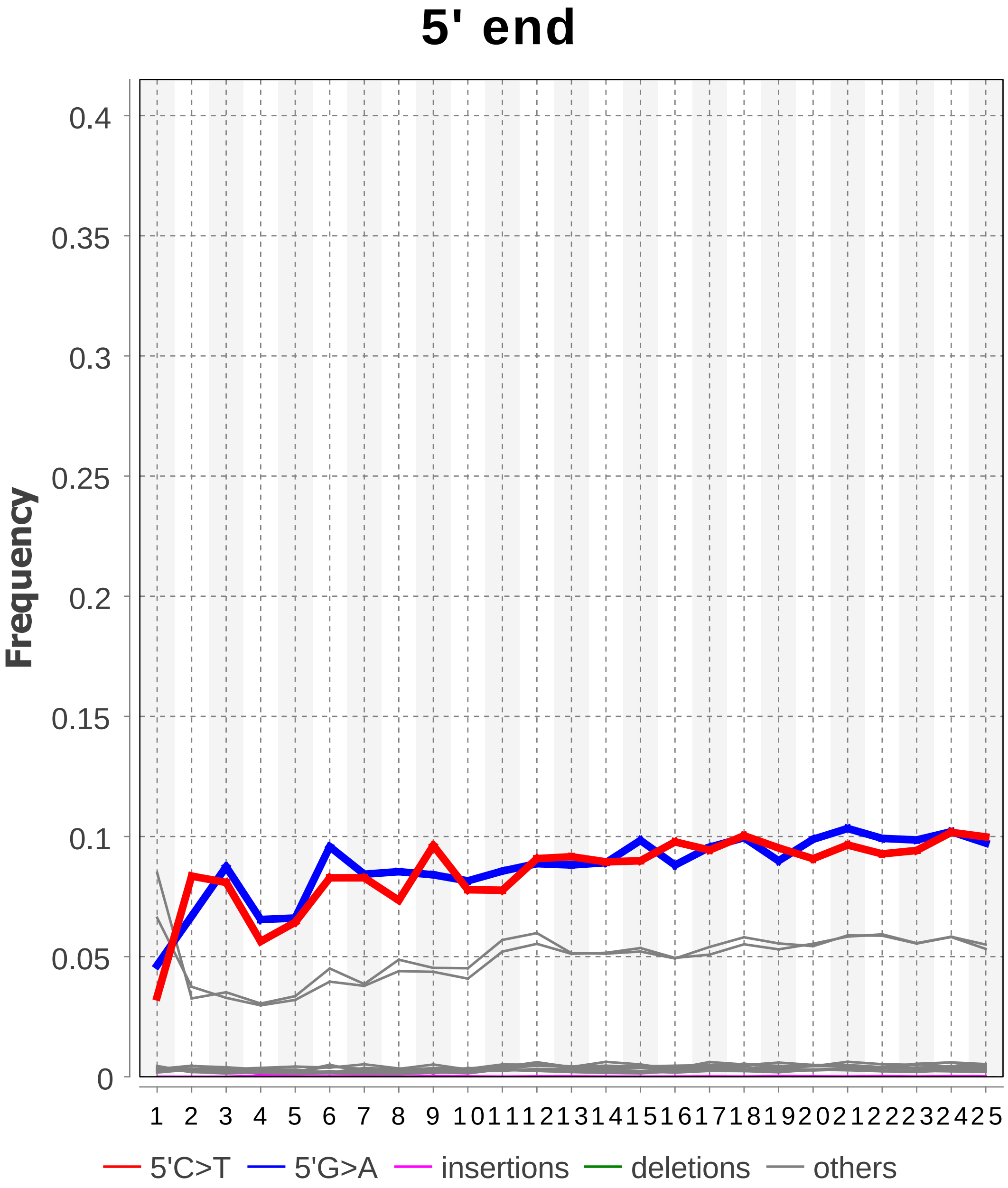

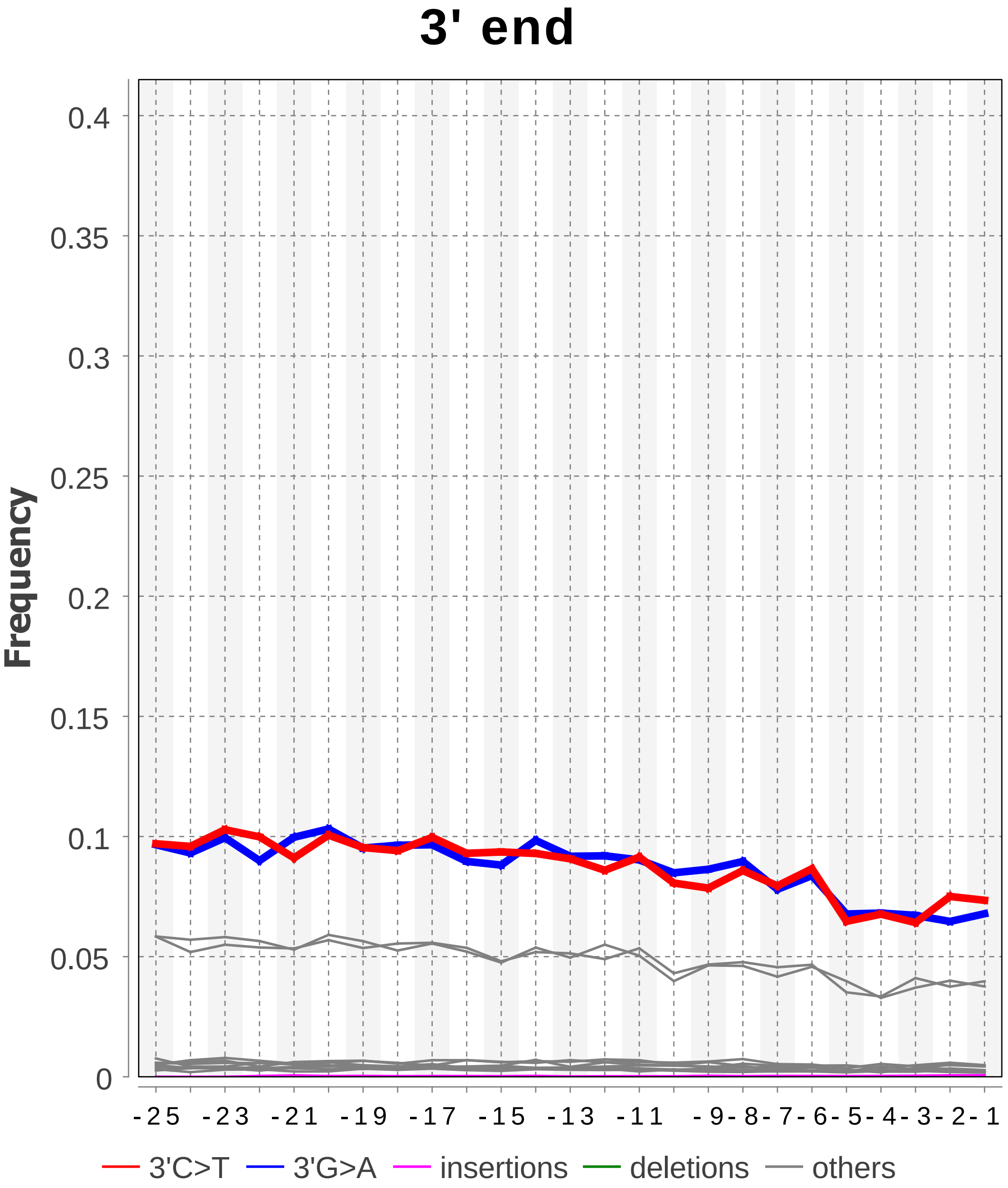

6733_MarkDuplicates

Number of used reads: 86,157 (100.0% of all input reads)

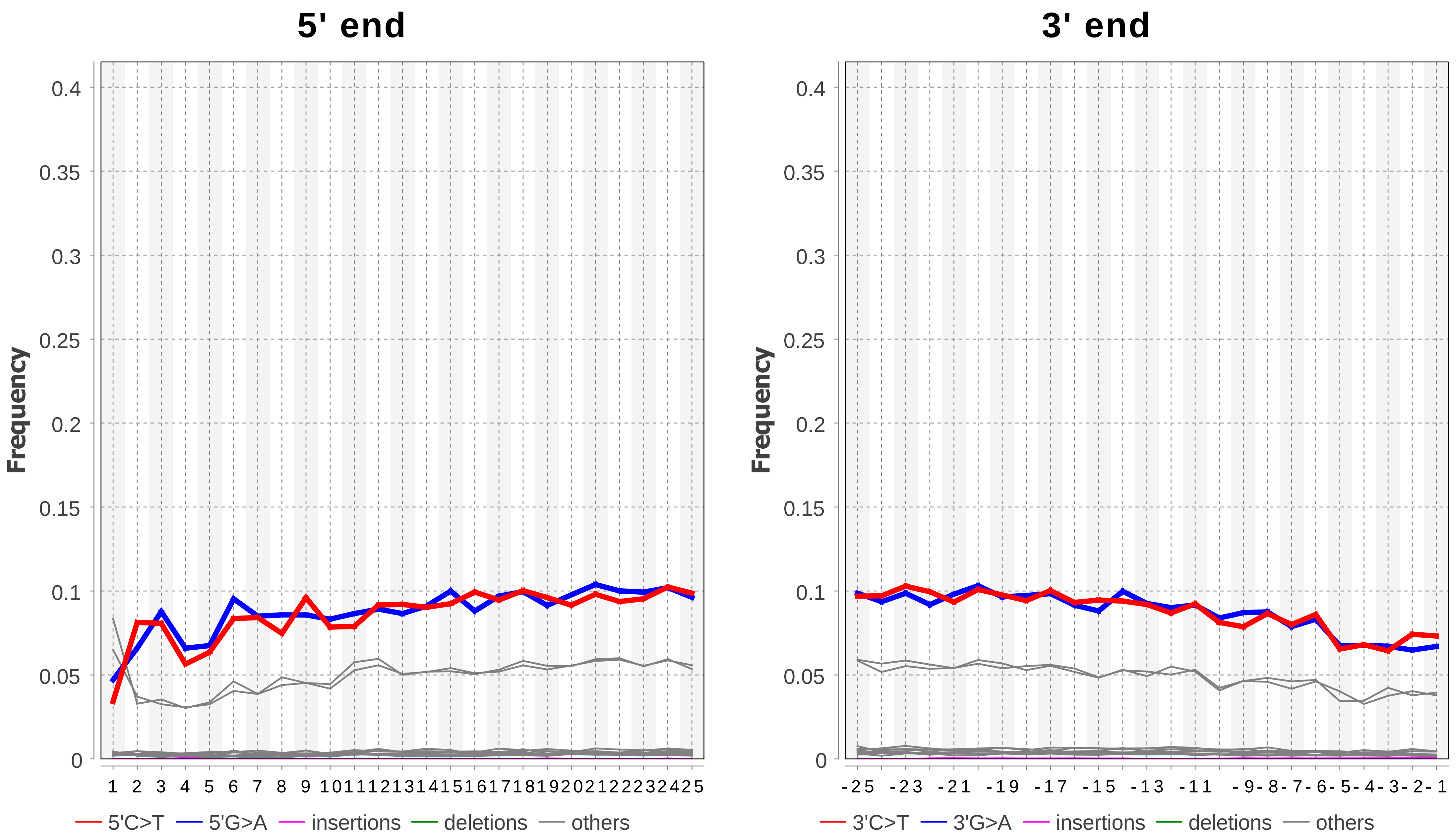

7638_aln

Number of used reads: 171,638 (100.0% of all input reads)

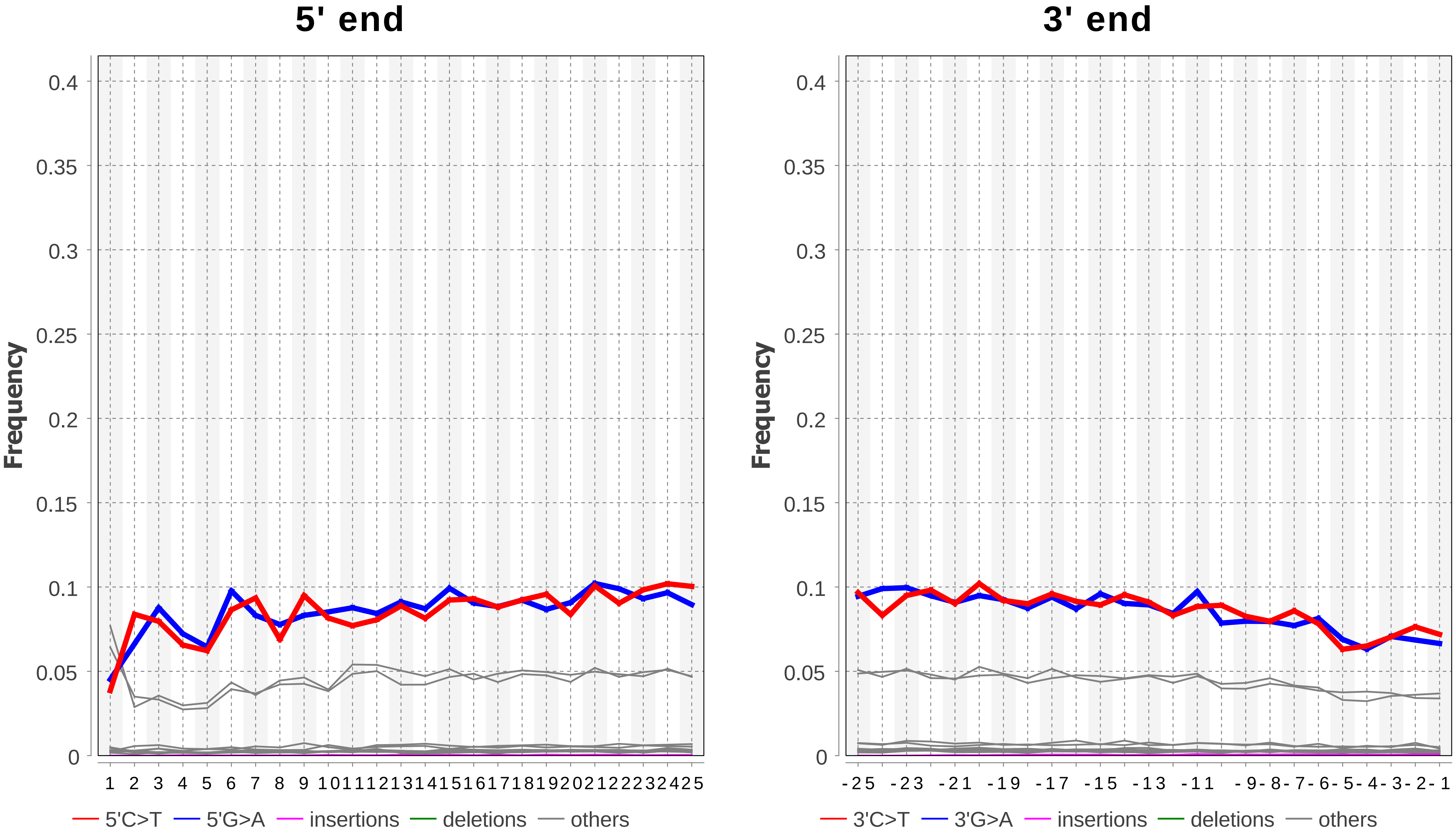

7638_MarkDuplicates
Number of used reads: 126,358 (100.0% of all input reads)

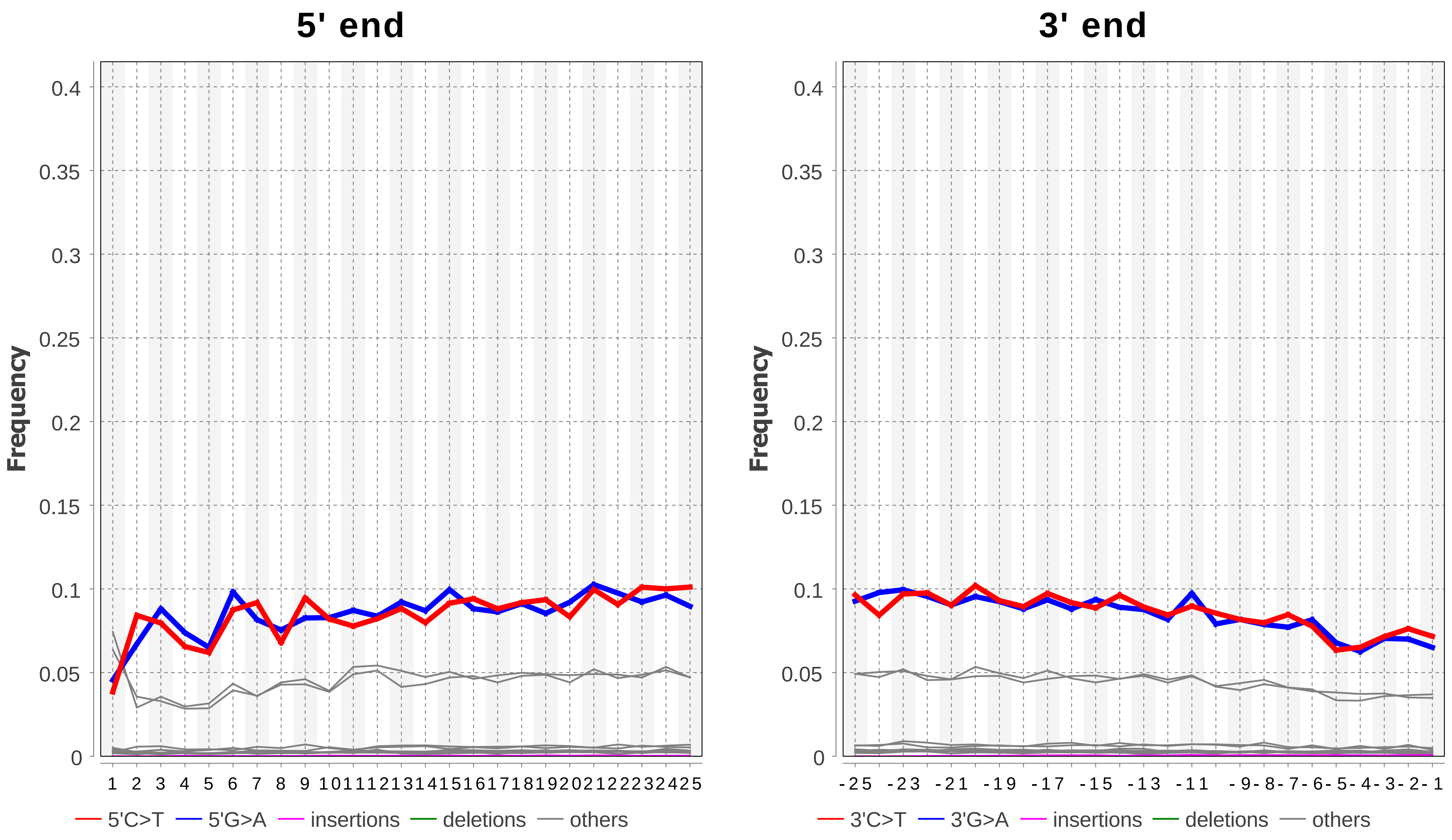

15153_aln

Number of used reads: 265,623 (100.0% of all input reads)

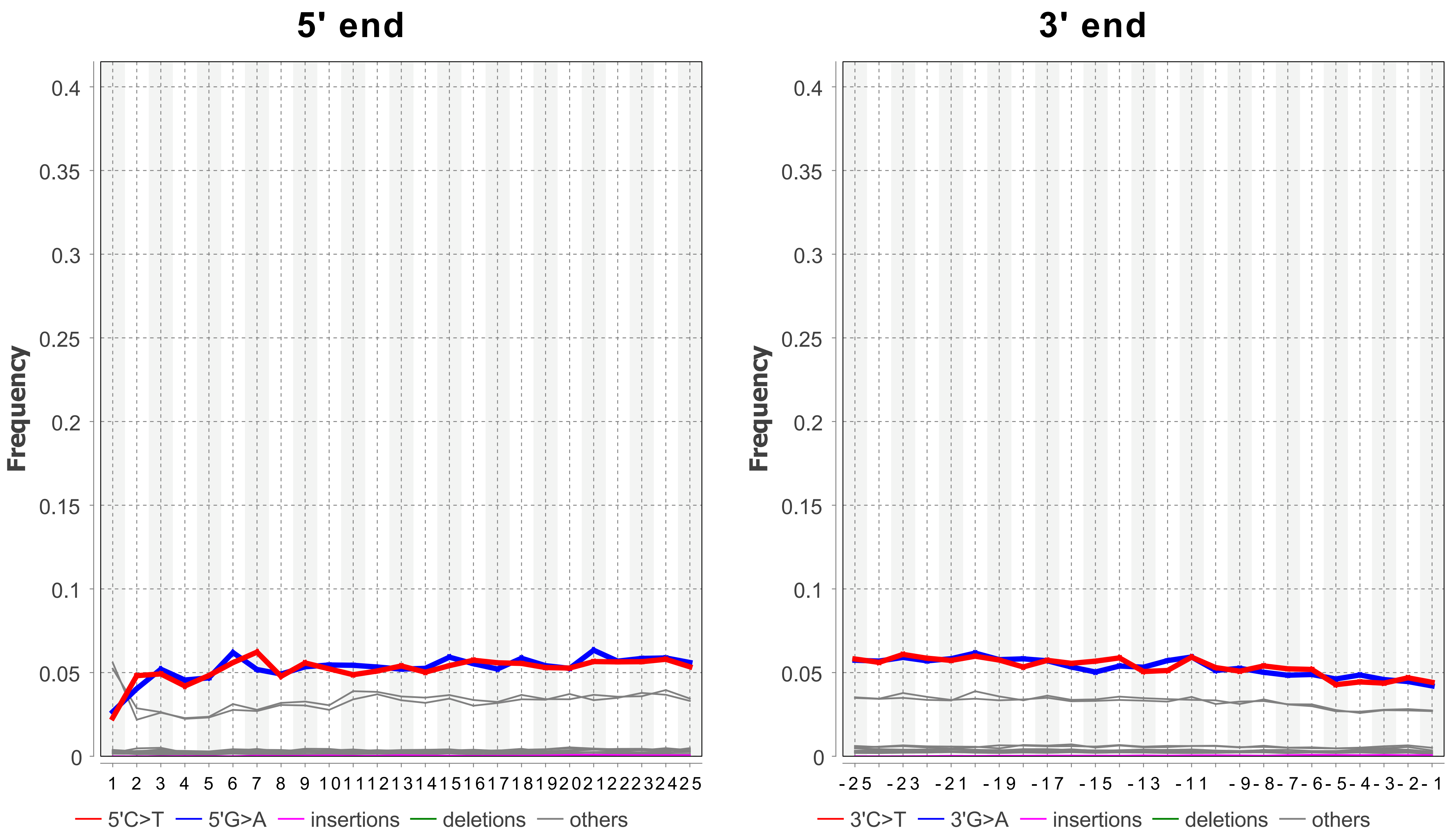

15153_MarkDuplicates
Number of used reads: 223,553 (100.0% of all input reads)

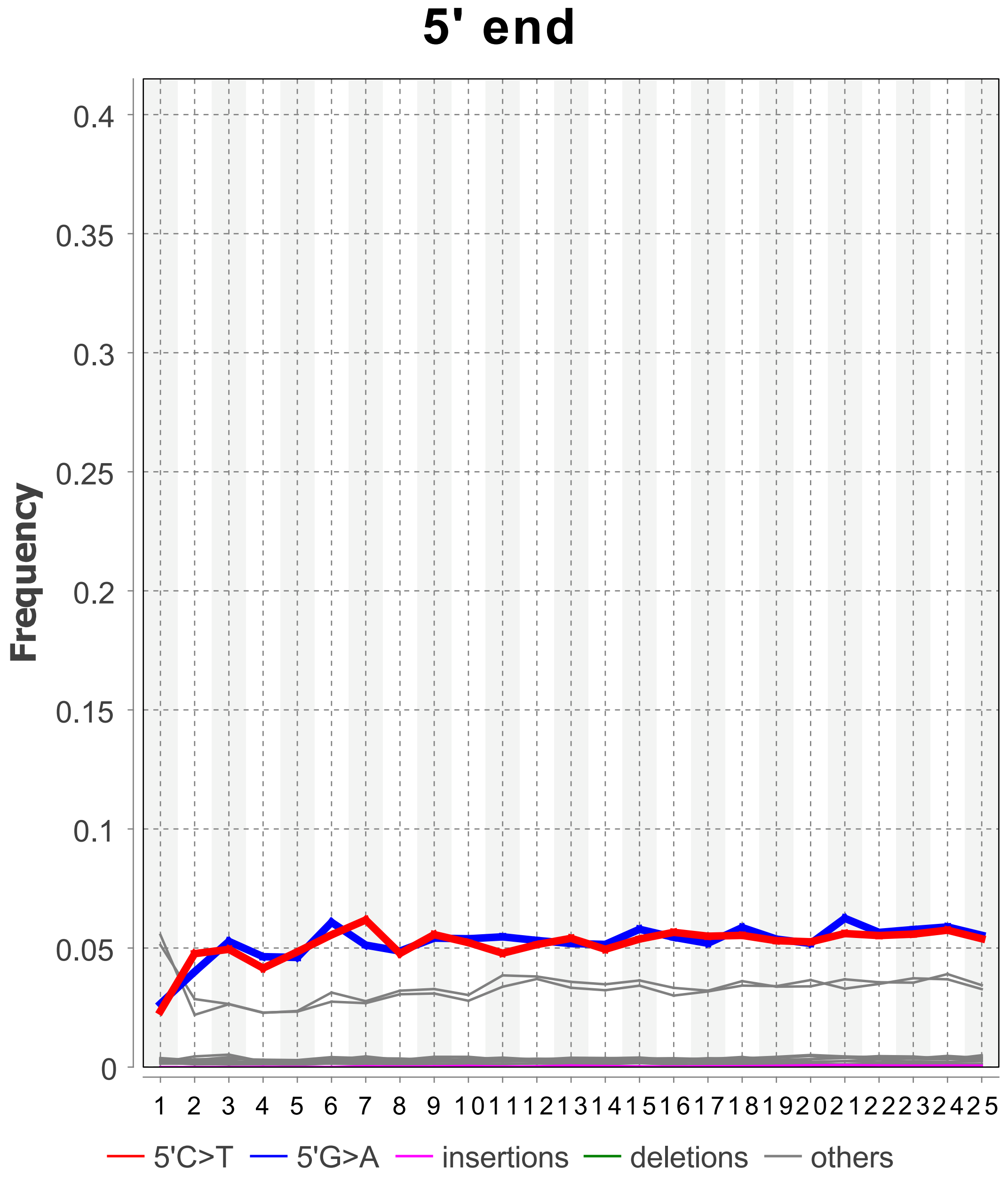

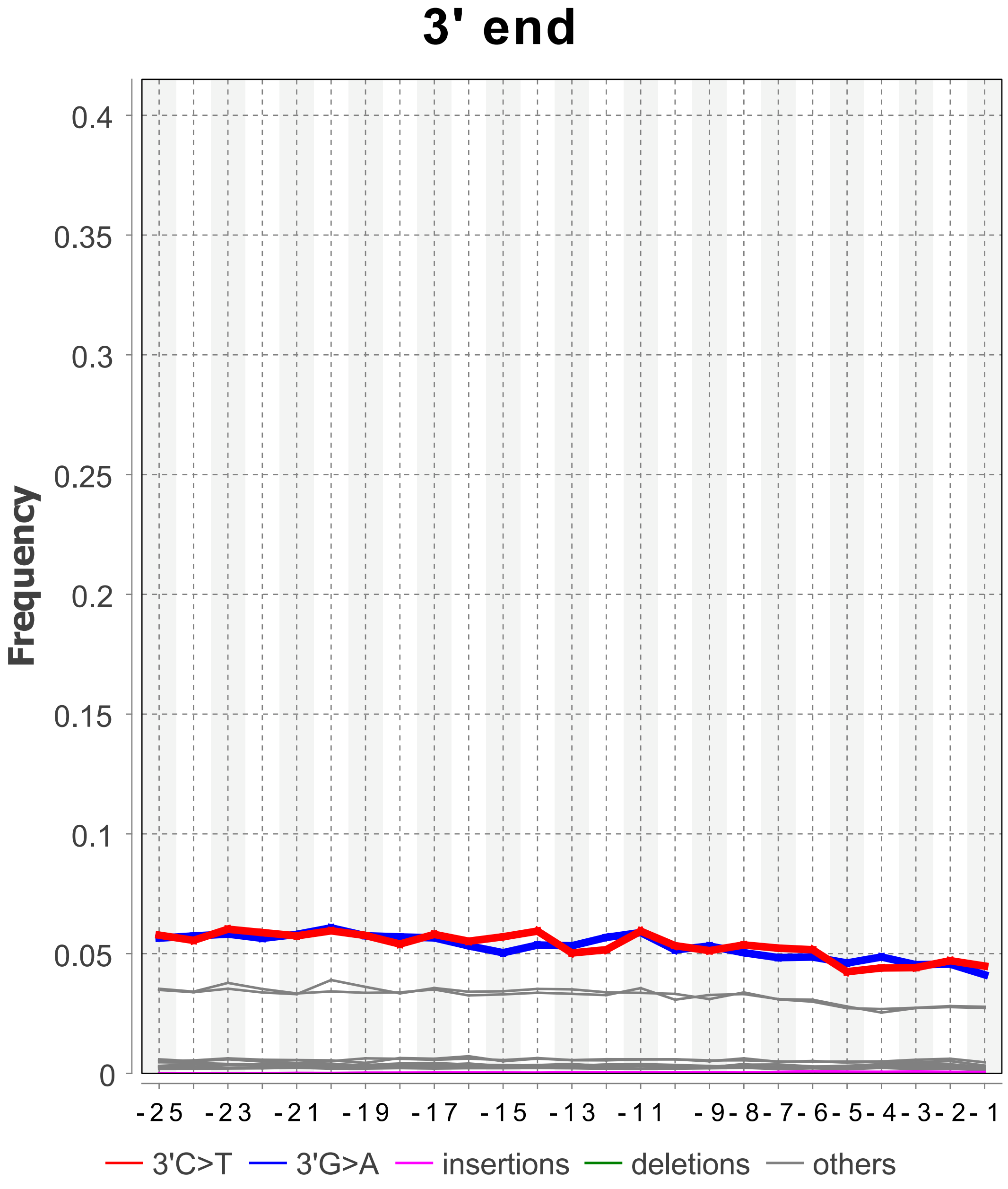

15154_aln

Number of used reads: 131,215 (100.0% of all input reads)

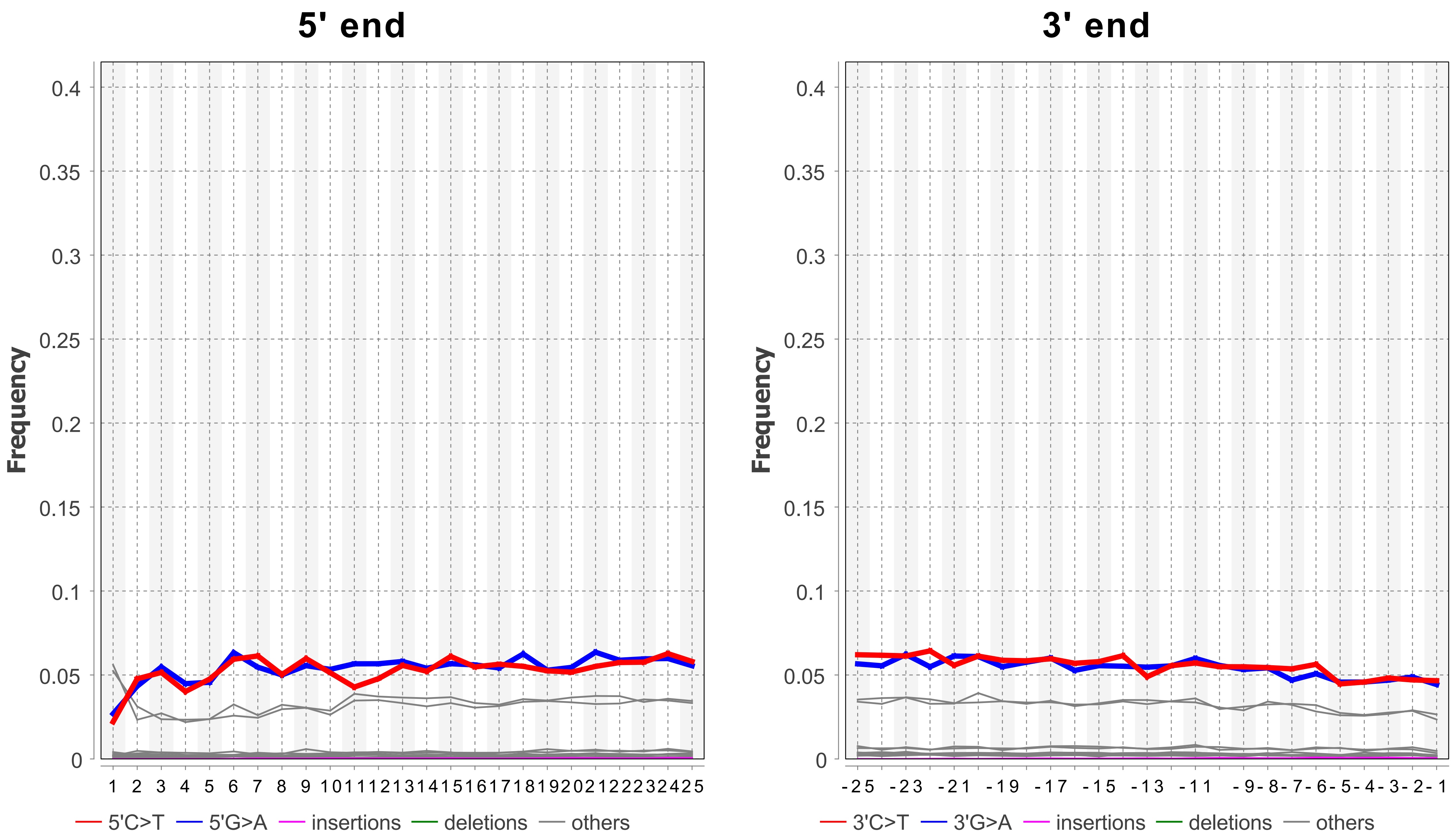

15154_MarkDuplicates

Number of used reads: 111,842 (100.0% of all input reads)

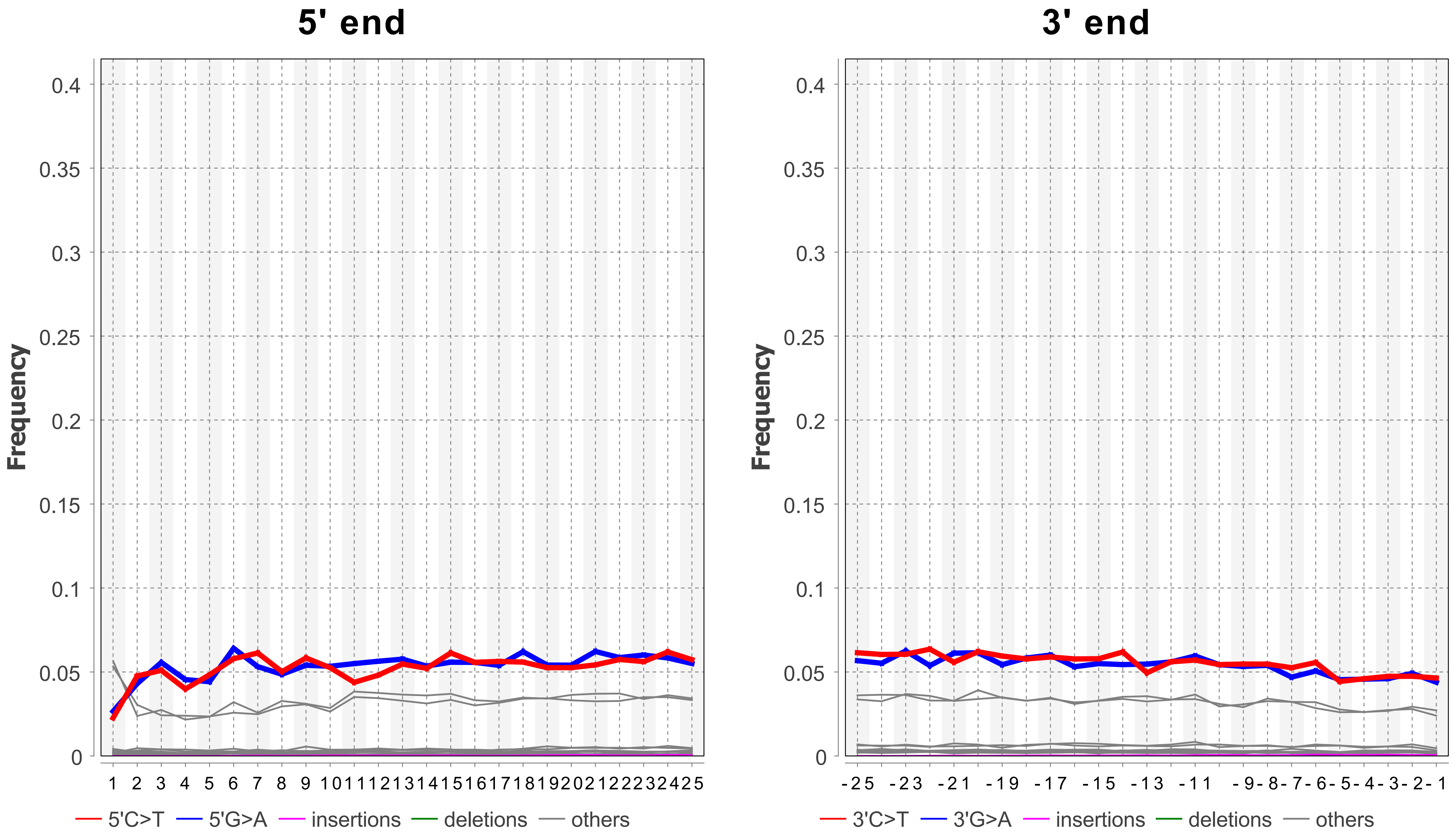

Supplement: Supplementary file 3 — Additional file 3: Figure S3. Damage plots of unmerged mapped reads (before and after duplicate removal) among samples. Red and blue lines denote C to T and G to A misincorporations, respectively, towards the ends. [file 12862_2024_2235_MOESM3_ESM.pdf]
